# A Supervised Network Analysis on Gene Expression Profiles of Breast Tumors Predicts a 41-Gene Prognostic Signature of the Transcription Factor *MYB* across Molecular Subtypes

**DOI:** 10.1155/2014/813067

**Published:** 2014-02-03

**Authors:** Li-Yu D. Liu, Li-Yun Chang, Wen-Hung Kuo, Hsiao-Lin Hwa, King-Jen Chang, Fon-Jou Hsieh

**Affiliations:** ^1^Biometry Division, Department of Agronomy, National Taiwan University, Taipei 106, Taiwan; ^2^Department of Obstetrics and Gynecology, College of Medicine, National Taiwan University, Taipei 100, Taiwan; ^3^Department of Surgery, College of Medicine, National Taiwan University, Taipei 100, Taiwan; ^4^Cheng Ching General Hospital, Taichung 400, Taiwan; ^5^Research Center for Developmental Biology and Regenerative Medicine, National Taiwan University, Taipei 100, Taiwan

## Abstract

*Background. MYB* is predicted to be a favorable prognostic predictor in a breast cancer population. We proposed to find the inferred mechanism(s) relevant to the prognostic features of *MYB* via a supervised network analysis. *Methods*. Both coefficient of intrinsic dependence (CID) and Galton Pierson's correlation coefficient (GPCC) were combined and designated as CIDUGPCC. It is for the univariate network analysis. Multivariate CID is for the multivariate network analysis. Other analyses using bioinformatic tools and statistical methods are included. *Results. ARNT2* is predicted to be the essential gene partner of *MYB*. We classified four prognostic relevant gene subpools in three breast cancer cohorts as feature types I–IV. Only the probes in feature type II are the potential prognostic feature of *MYB*. Moreover, we further validated 41 prognosis relevant probes to be the favorable prognostic signature. Surprisingly, two additional family members of *MYB* are elevated to promote poor prognosis when both levels of *MYB* and *ARNT2* decline. Both *MYBL1* and *MYBL2* may partially decrease the tumor suppressive activities that are predicted to be up-regulated by *MYB* and *ARNT2*. *Conclusions*. The major prognostic feature of *MYB* is predicted to be determined by the *MYB* subnetwork (41 probes) that is relevant across subtypes.

## 1. Introduction

Breast cancer (BC) has become a global health problem among women in recent years. Finding more cost effective strategies to better control this problem is highly desirable. It should be noted that the nature of heterogeneity for breast cancer at molecular and clinical level [1] still remains challenging to breast cancer care and prevention.

We selected* MYB* for the global network study because it is essential for mammary gland development and tumorigenesis [2]. However, the most important reason was that our preliminary data suggested *MYB* to be a good prognostic predictor among 181 infiltrating ductal breast carcinomas based on Kaplan-Meier survival analysis.

Miao et al. described a transient defect in mammary gland development in the mouse model with the genetic deletion of *MYB*. They suggested that *MYB* is critical for tumor growth and mammary carcinogenesis [2]. *MYB* transcription factors (TFs) in the *MYB* family are widely distributed in eukaryotic organisms [3, 4]. *MYB* family members consist of *A*-, *B*-, and *C*-*MYB*s in diverse vertebrates. *A-MYB *(*MYBL1*) plays a critical role in mammary gland development. In female mouse model, *MYBL1* is expressed in breast ductal epithelium, mainly during pregnancy-induced ductal branching and alveolar development [5]. *B-MYB* (*MYBL2*), a mitotic regulator, could be implicated in breast tumorigenesis because it is detected in a wide variety of cancer cells and plays an essential role during cell cycle progression [6–8]. It has been documented that *C-MYB *(*MYB*) plays different roles in normal and cancer cells [9]. The findings of Thorner et al. [10] indicated that *C-MYB* may not be behaving as an oncogene in estrogen receptor positive (ER(+)) luminal breast tumors and suggested that it may be behaving as a tumor suppressor in this disease. All these findings described above indicate the important roles of *MYB* family members in relation to mammary gland and breast cancer development in the model systems. However, more studies in a genome-wide scale for finding the roles of *MYB* in breast cancers are essential to fill in the gaps for the current findings in the field.

This study aimed at reassessing the developmentally important transcription factor *MYB* mediated transcriptional regulatory networks in relation to breast cancer development and clinical outcome.

## 2. Materials and Methods

### 2.1. Features of Surgical Specimens for Generating the Dataset of Gene Expression Profiles

We used immunohistochemical (IHC) statuses for three biomarkers (i.e., estrogen receptor *α* (ER), progesterone receptor A (PR), and HER-2/neu (HER)) as the classifiers to identify eight intrinsic subtypes. However, for ERBB2 (IHC score: 2+), determination of Her-2/neu gene copy number by chromogenic *in situ* hybridization (CISH) was performed [12]. As such, IHC/CISH status was used for determining HER status.

Ninety specimens of primary infiltrating ductal breast carcinomas (IDCs) consist of group IE (i.e., ER(+)PR(+)) (61/90) and group IIE (i.e., ER(+)PR(−)) (29/90). Ninety-one samples of IDCs consist of triple negatives (TN) (i.e., ER(−)PR(−)HER(−)) (48/91), ERBB2+ (i.e., ER(−)PR(−)HER(+)) (29/91), ER(−)PR(+)HER(−) (5/91), ER(−)PR(+)HER(+) (6/91), and ER(−)PR(+)HER(?) (3/91). Those samples were obtained from patients who underwent surgery at National Taiwan University Hospital (NTUH) between 1995 and 2007. The tumor samples for this study were the remaining frozen samples from diagnostic purpose. All patients provided informed consent according to the guidelines approved by the Institutional Review Board (IRB) at NTUH (IRB number: 200706039R, Research Ethics Committee at National Taiwan University Hospital, Taipei, Taiwan). The survival status of this cohort was derived from the recent medical recording collected in 2011 (by WHK). Other medical records of the patients were obtained from the great assistance from the office of medical record (Cancer Registry, Medical Information Management Office, NTUH). At the time of this study, the record of cancer treatments for these cancer patients was only partially complete.

The microarray data for this study (181 gene expression profiles) have been submitted to the NCBI Gene Expression Omnibus (GEO; http://www.ncbi.nlm.nih.gov/geo) under accession number GSE24124. In this study, we designated 90A as the gene expression microarray dataset for 90 ER(+) breast tumors. It consists of two subsets that are group IE (61A) and group IIE (29A). The definition for 91A is the gene expression microarray dataset of 91 ER(−) breast tumors. It consists of subsets for TN (48A), ERBB2+(29A), ER(−)PR(+)HER(−) (5A), ER(−)PR(+)HER(+) (6A), and ER(−)PR(+)HER(?) (3A). In addition, we designated the cohort of Groups IE and IIE containing ninety gene expression profiles as 90A cohort. The cohort from subcohorts for TN, ERBB2+, ER(−)PR(+)HER(−), ER(−)PR(+)HER(+), and ER(−)PR(+)HER(?) to make 91 gene expression profiles was designated as 91A cohort. For 181A cohort, it includes 90A cohort and 91A cohort.

### 2.2. Microarray Data Analyses

A global view of a gene profile per breast tumor specimen was analyzed using Human 1A (version 2) oligonucleotide microarray (half a genome size: 22 k) (Agilent technologies, USA). The heatmaps were displayed after unsupervised hierarchical clustering. For unsupervised hierarchical clustering, the log2 ratio for each gene was first centered by subtracting the median across all samples to discriminate the subclass of the dataset. The “hcluster” function in “stats” package was utilized to perform the unsupervised clustering. We used the Euclidean distance and the complete linkage as the default settings. Then, the selected gene expression profiles were fed into the software R2.15.1 for displaying gene list (*Y* axis) that is derived from hierarchical clustering analysis on the gene profiles of selected arrays (*X* axis) to generate the heatmaps. The heatmap was produced by “rect” function to make the customized view of the subcohorts. In addition, we used Gene Spring GX7.3.1 (Agilent Technologies, USA) for generating Venn diagrams and for retrieving updated gene annotation. ANOVA has the advantage of performing both dichotomous and multichotomous analyses. ANOVA test for the relationship between mRNA levels of *MYB *and the statuses of a clinical index of interest in a given population as well as the statistical methods for establishing *MYB* transcriptional regulatory network were described previously [11–14]. We used the same data analyses described above for analyzing other transcription factors of interest. We performed Kaplan-Meier survival analyses [15] using “survival” package in R (version 2.15.1) using the gene profiles of 90A cohort, 91A cohort, and 181A cohort or the extracted gene pools of interest in the assigned cohorts. To quantify the weight of hazard ratios associated with prognostic gene signature and the traditional prognostic factors in a given cohort of interest, both univariate and multivariate COX proportional hazard (COXPH) regression models in R package (version 2.15.1) were performed.

### 2.3. Features of the Adapted Network Analysis Based on the Dataset of 181 Gene Expression Profiles

The DNA microarray becomes mainstay technique used in medical research. In recent years, we have designed the network analysis for full prediction of a transcription network for a given transcription factor in a population of interest [11, 13]. However, we present the partial results predicted by a supervised network analysis mainly due to the existing limitations in this dataset of ours described in Sections 2.1 and 2.2. A supervised network analysis approach was developed [12].

We initially designed the network analysis approach including IHC stain to guide the network prediction, in part [13]. The predicted numbers of human putative transcription factors genome-wide are between 1,850 and 4,105 [16]. It is impossible to provide IHC stain for each transcription factor of interest using clinical tumor samples within the same cohort. Therefore, we used the data at mRNA level to find the inferred target genes for a TF of interest as the rule of thumb.

Statistically, to deal with continuous variables of interest, CID has the advantage to measure the subCID value of a subgroup with a small *N* number (*n*≒10) without increasing the statistical errors. Biologically, we chose the 1/10th subgrouping strategy (*n*≒10) among tested subgrouping strategies although each transcription factor of interest may need adjust subgrouping strategy for network analysis to increase both sensitivity and specificity of network analysis. However, we constantly compare subtype relevant transcriptional regulatory events that are normally in a small sample group (*n*≒30) in our model system. It is reasonable to set 1/10th subgrouping as the best of choice.

### 2.4. Experimental Design

The 181 gene expression profiles of the human infiltrating ductal breast carcinoma contain more than eight breast cancer intrinsic subtypes based on IHC/CISH results. This offers the opportunity of finding prognostic relevant gene pools among breast cancer subtypes. In this model system, Kaplan-Meier survival analysis [15] predicts *MYB* to be a favorable prognostic predictor in 181A cohort. In this study, the rationale for selecting 90th percentile as the cut-off point for Kaplan-Meier survival analysis is mainly to match the subgrouping strategy of both univariate and multivariate CID.

The transcriptional regulatory network analysis is highly sensitive in measuring both the existing and novel gene expression relationships between a TF and its potential target gene in a population of interest [13]. In addition, the gene expression relationships between the combinatorial interacted TFs (*N*≧2) and their potential shared target gene in a given population are measured [11]. Here, we designed a combined strategy including both network analysis and Kaplan-Meier survival analysis to find the prognostic relevant transcriptional regulatory subnetwork of *MYB*. The prognostic values of these network components (i.e., probes) were further predicted by Kaplan-Meier survival analysis. Thus, such strategy allows 10% population to be selected by their relevance to both the inferred transcriptional event and prognosis. For the transcription factor *MYB*, we proposed that the most relevant subnetwork of *MYB* with the highest subCID value in a subset of tumor samples may be co-localized with the top 10% tumor sample population that expresses high levels of *MYB* and indicates a favorable prognosis. Meanwhile, we added a few key steps to control confounders and to quickly locate the major prognostic features of *MYB*. First, we chose three populations of interest (i.e., 91A cohort, 90A cohort, and 181A cohort) and classified their prognostic predictors into four types based on their differential relevance among these populations. Second, we identified the subpool of genes that is not only a subpool of the prognostic predictors of a given type but the components of *MYB* inferred network. They were classified as genes with a given feature type. Third, we proposed those genes to represent the major prognostic feature of *MYB* based on the gathered evidence from the inferred transcriptional regulatory network of *MYB* in relation to biochemical phenotypes, malignant phenotypes, and supporting evidence from others. Fourth, we further selected the overlapping gene set in the given feature type of both 90A cohort and 181A cohort to be the consensus prognostic signature of *MYB*. Finally, the prognostic signature relevant to clinicopathological parameter(s), subtype(s), and treatment response(s) may be concluded from this study.

## 3. Results and Discussion

Our evaluation focused on the clinical pathophysiological and/or subtypical implication of *MYB*. Such genome-wide transcriptional activities might offer us new insights into the clinical behaviors of breast cancers, such as their responsiveness to standard cancer therapies and survival after surgical removal of breast tumor(s). For instance, both network prediction and some validated evidence suggested a transcription factor *STAT3* to be a center regulator of estrogen receptor negative (ER(−)) breast cancers [12]. Many potential and existing drug targets or genes resistant to standard cancer treatments have been identified via an established scheme for the network analysis [12]. Such new approach allows more valuable information to be extracted and they can be linked together to form functional networks. These inferred transcriptional regulatory networks in a clinical breast cancer model system are expected to assist us in unraveling the identity of breast cancer subtypes at molecular level. Meanwhile, the options for both cancer prevention and cancer treatment of different breast cancer subtypes may be indicated via this study. Finally, the discovery of the gene signature, which is prognostically relevant in a subset of highly *MYB* expressed breast tumors, is expected to be achieved.

### 3.1. The Most Relevant Transcriptional Regulatory Event of *MYB* in Regulating Genes for Predicting Clinical Outcome Is Neither Subtype Dependent Nor Unique Clinical Parameter Dependent

#### 3.1.1. The Potential Clinical Impact of *MYB* as a Tumor Suppressor and Its Relation with Favorable Prognostic Features of *MYB*

**Figure 1:**
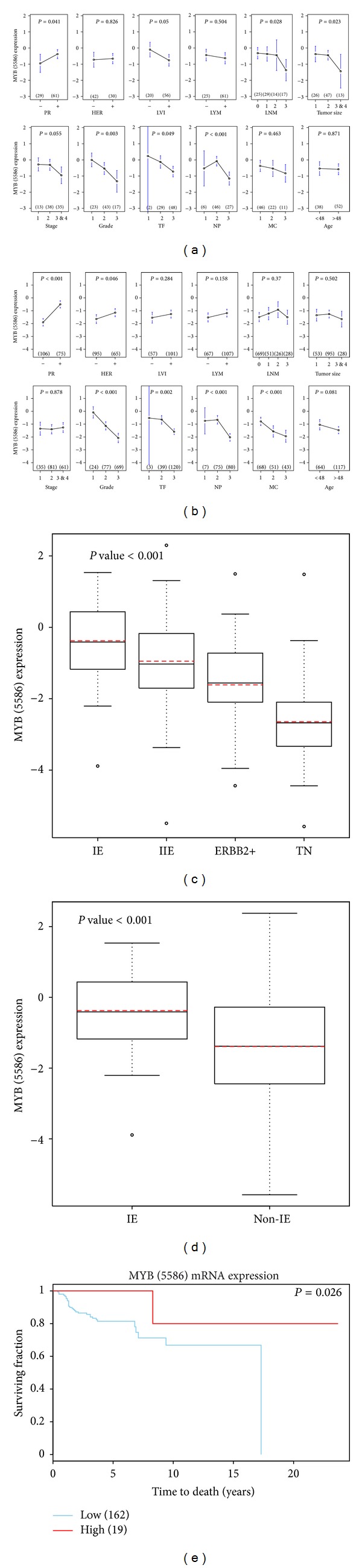
Clinical impact of *MYB* in two cohorts of infiltrating ductal breast carcinomas. ANOVA test results of *MYB* (5586) mRNA levels in eight clinical indices, progesterone receptor (PR), HER-2/neu (HER), lymphovascular invasion (LVI), lymph nodal category (lymph node metastasis status (LYM) and number of lymph node metastasis (LNM)), age, tumor size (size), grade (nuclear pleomorphism, mitotic count, and tubule formation), and cancer stage in 91A cohort and 181A cohort, respectively ((a) and (b)). Lower panel, box plot analysis of *MYB* (5586) mRNA levels in four subtypes (i.e., Groups IE, IIE, triple negatives (TN), and ERBB2+) (c) and in two types (i.e. group IE (IE) and non-group IE(non-IE)) (d). The red dot line within box stands for the mean value for each subgroup in the plot. The black line within box stands for the median value for each subgroup in the plot. The survival analysis (Kaplan-Meier survival analysis) on a breast tumor group with high *MYB* mRNA levels versus the group with low *MYB* mRNA levels in 181A cohort (e). The Agilent feature number for *MYB* is 5586.

The mRNA levels of *MYB* are relatively high in Group IE of ER(+) IDCs as compared to other subtypes in 181IDCs (Figures 1(c) and 1(d)). Typically, an increased gene expression of *MYB* is relevant in PR(+) IDCs (see results of ANOVA tests in Figures 1(a) and 1(b)). We predicted that the clinical outcome in these subtypes may be favorable due to the action of *MYB*, in part. In addition, *MYB *is one of the determinants for early tumor development in clinicopathological features of lymphovascular invasion (LVI), histological grade (Grade or G), tubule formation (TF), nuclear pleomorphism (NP), tumor size (size), and the number of lymph node metastasis (LNM) in 90A cohort (Figure 1(a)). It is also the significant determinant of early statuses for G, MC, NP, and TF in 181A cohort (Figure 1(b)).

To prove the clinical behavior of *MYB* due to its function as the transcription factor, a series of analyses was performed to dissect the major action of *MYB* via the *MYB* transcriptional regulatory network approach. As a result, the predicted tumor suppressive activities of* MYB* may be due to its transcriptional activities. These activities of potential *MYB* target genes are overlapping with some favorable prognostic predictors in the tested cohorts.

Two clinically relevant *MYB* clusters and the predicted *MYB *transcriptional activities suggest *ARNT2* to be the obligate gene partner of *MYB *(Figures 3(a) and 3(b)). It potentially co-contributes with *MYB* for its clinical impact on breast cancers which is significant in both 90A cohort and 181A cohort (Figure 3(a)). The results from Venn diagram analysis further demonstrate the most relevant activities of *MYB* in coupling with *ARNT2* via three networks of *MYB*_*ARNT2* in 90A cohort and 181A cohort, respectively (Figure 3(b)). The gene profiling of clinical relevance and of involved signal transduction pathways for two networks at the lower panel is demonstrated by bar charts suggesting the tumor suppressive effect of *MYB* (Figures S6.1–S6.9, in Supplementry Material available online at http://dx.doi.org/10.1155/2014/813067; Figures 3(c) and 3(d)). Histological grade is predicted to be heavily regulated by *MYB* while comparing to nine other clinical parameters in both 90A cohort and 181A cohort (Figures 1(a), 1(b), and 3(c)). In addition, the cancer-related signal transduction pathway (STP) for ribosome has more genes to be the inferred components of *MYB* network than other twelve STPs do (Figure 3(d)).

We observed that increased *MYB* expression may be associated with relatively early disease development, non-tumor component, and 41 prognostic relevant *MYB* inferred target probes (Figures S8.1-S8.2 and Figures 1(a) and 1(b)). *MYB* is predicted to suppress the expressions of key components in cancer-related signal transduction pathways, such as cell cycle, p53, PDGFRB, ERBB2 and VEGF (Figures S6.1–S6.3 and S6.5-S6.6). In addition, tumor suppressive activities of *MYB* are demonstrated by down-regulating a set of genes that are predicted to attenuate the pathophenotypic development of breast tumors (Figures S6.7–S6.9). For instance, the progression of histological grade, LVI, and tumor size are suppressed in a subset of *MYB* highly expressed breast tumors showing relatively low histological grade, LVI and tumor size. This may be due to the actions of some inferred gene components in the transcriptional regulatory network of *MYB*_*ARNT2*. Importantly, *ARNT2*, *MYB*, *XBP1,* and *SALL2* are the candidate drivers for attenuating histological grade promotion (Figure S6.7). *XBP1*, *SALL2*, *POU2F1*, *ARNT2,* and *MYB* are the candidate drivers in preventing LVI progression (Figure S6.8). *MYB*, *ARNT2,* and* POU2F1* are the potential drivers in attenuating the tumor size progression (Figure S6.9). Based on the brief analysis on the tumor suppressive activities of *MYB* described above, it remains largely unknown whether or not *MYB* suppresses the gene expressions of the risk factors, which are responsible for poor clinical outcome. However, we observed that the favorable prognostic feature of *MYB* may be due to partially down-regulating gene expressions for epithelial-to-mesenchymal transition (EMT) markers predicted by network analysis (Figure S6.10). Moreover, the EMT activities were predicted to be partially suppressed by *SALL2*.* SALL2* is a gene component of 41-gene signature and is a putative shared target gene of *MYB *and *ARNT2*. EMT related genes are involved in the program of development of cancer cells characterized by loss of cell adhesion, repression of E-cadherin expression, and increased cell mobility for promoting tumor metastasis.

#### 3.1.2. The Potential Clinical Impact of ARNT2 as the Obligate Transcription Factor Partner of *MYB* and Its Relation with Favorable Prognostic Features of *MYB*

Aryl-hydrocarbon receptor nuclear translocator 2 (ARNT2) was identified as a homologue with a high degree of sequence similarity to Aryl-hydrocarbon receptor nuclear translocator (ARNT) [17]. *ARNT2* is a transcription factor. Human ARNT2 cDNA was identified by Barrow et al. [18]. The actual function of *ARNT2* in cancer still remains largely unknown. Qin et al. [19] reported *ARNT2* affecting HIF1 regulatory signaling and metabolism in human breast cancer cell model. It is a potential favorable prognostic factor in breast cancer when it is elevated and it is expressed higher in tumor component than in non-tumor component [20]. Our finding shows that *ARNT2* is not a prognostic indicator in both 90A cohort and 181A cohort (III and IV of Figure S5.2). However, its mRNA expression is up-regulated in tumor component, which is consistent with the report [20]. Liu et al. [21] reported that ARNT2 dimerizes with SIM1 to up-regulate their downstream target genes (268 probes) *in vitro*, which are predicted to be functional in seven categories—transcription regulators, signaling components, metabolic enzymes, channels and transporters, cell adhesion and migration, miscellaneous and uncharacterized. Partial results of the supervised network analysis in Tables S1.3 and S1.4 show *MYB *and *ARNT2* shared target genes (57 genes) overlapping with the downstream target genes of ARNT2/SIM1 (Table S6.1). The *MYB* network predicts *ARNT2 *to be a target gene of *MYB* in 90A cohort (Figure S6.10).* ARNT2* and *MYB* share the large pools of target genes (7,225 probes in Table S1.3 and 5,308 probes in Table S1.4). A relatively lower amount of genes is putative target genes of *ARNT2*, which dimerizes with its essential TF partners. They are predicted to be not co-regulated by *MYB* (e.g., 2,322 probes of 152_ARNT2 in Table S1.3 and 3,962 probes of 120_ARNT2 in Table S1.4). This suggests the prognostic relevance of *ARNT2* alone to be less significant than that of *MYB* and *ARNT2* in our model system.

**Figure 2:**
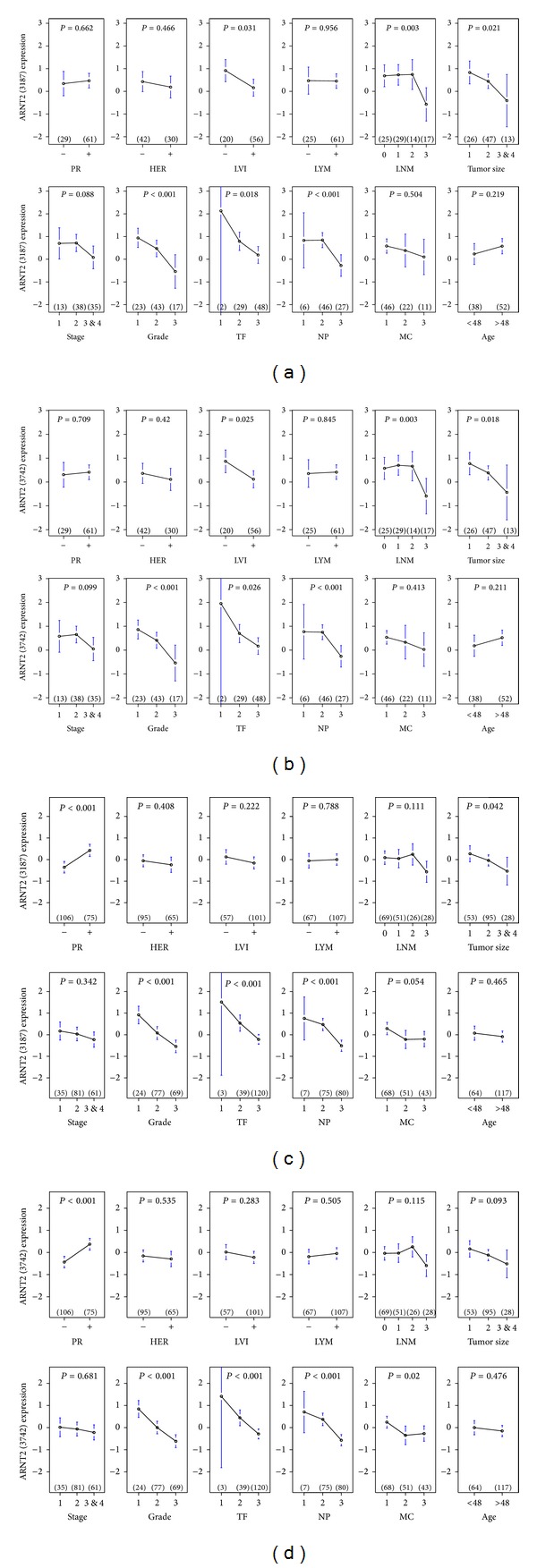
Clinical impact of* ARNT2* in two cohorts of infiltrating ductal breast carcinomas. ANOVA test results of ARNT2 (3187) and ARNT2 (3742) mRNA levels in eight clinical indices are shown in 90A cohort ((a) and (b)) and 181A cohort ((c) and (d)), respectively. There are two probes for ARNT2. They have the Agilent feature numbers 3187, 3742 and respectively.

*MYB *and *ARNT2* may mutually interact with each other in regulating their shared target genes during early tumor development and co-contribute to favorable prognosis indicated in both 90A cohort and 181A cohort (Figures S8.1 and S8.2). The supporting pieces of evidences are as follows. First, we observed *MYB* and one of *ARNT2* probes sharing the clinical impact on early development of histological grade, mitotic count, nuclear pleomorphism and tubule formation in 181A cohort (Figures 1(b) and 2(d)). Second, both *MYB* and *ARNT2* are determinants for the early development of LVI, G, TF, NP, size and LNM in 90A cohort (Figures 1(a), 2(a) and 2(b)). Third, the networks of *MYB*_*ARNT2* predict relatively low activities of the cancer-related signaling pathways due to not regulating key oncogenic signaling molecules or suppressing the oncogenic signaling molecules (Figures S6.1–S6.6). Fourth, the clinically relevant and cohort enriched networks of *MYB*_*ARNT2* may participate in breast cancer development only at early phase. For instance, relatively high levels of both *MYB* and *ARNT2* in the breast tumor components show their clinicopathological features with low grade; LVI negative and LYM negative (Figures S6.7–S6.9) in a subset of patients in 90A cohort.

### 3.2. The Classification of Prognostic Relevant *MYB_ARNT2* Subnetworks via Their Relevance in Predicting the Clinical Outcome in the Specific Cohort(s)

*MYB* is a predictor of favorable prognosis in 181A cohort (Figure 1(e)). It is likely that the overall clinical impact of *MYB* in this cohort may serve as a determining factor for the good clinical outcome.

Each tumor sample has the unique network of *MYB*_*ARNT2*. Based on the methodology used to establish the inferred network of *MYB*_*ARNT2*, the most relevant tumor suppressive activities of this network are determined mainly from the 1/10th tumor sample group that has relatively high mRNA levels of both *MYB* and *ARNT2* in a given cohort to contribute the highest subCID value based on CID subgrouping strategy [11]. We analyzed the components of this network genome-wide to further evaluate if these network components serve as prognostic indicators when each of them is expressed at a level within top ten percent (i.e., elevated) in a population of interest via Kaplan-Meier survival analysis [15]. Based on this screening strategy, only limited amounts of probes are found to be significant (*P*≦0.05) (Table 1 and Figure 5). The significance for each probe of interest in predicting clinical outcome is identified either in one population or in several populations. Three populations (90A cohort, 91A cohort and 181A cohort) were used to classify a gene pool potentially to be the prognostic indicators in at least one of three populations. Four subpools of genes have been derived from this classification strategy and have been designated as four feature types (Figure 4).

### 3.3. The Most Relevant Feature for Transcriptional Regulatory Event of *MYB* in Regulating Genes Responsible for the Favorable Clinical Outcome Is across Subtypes but Enriched in ER(+) IDCs (i.e., Feature Type II)

**Figure 3:**
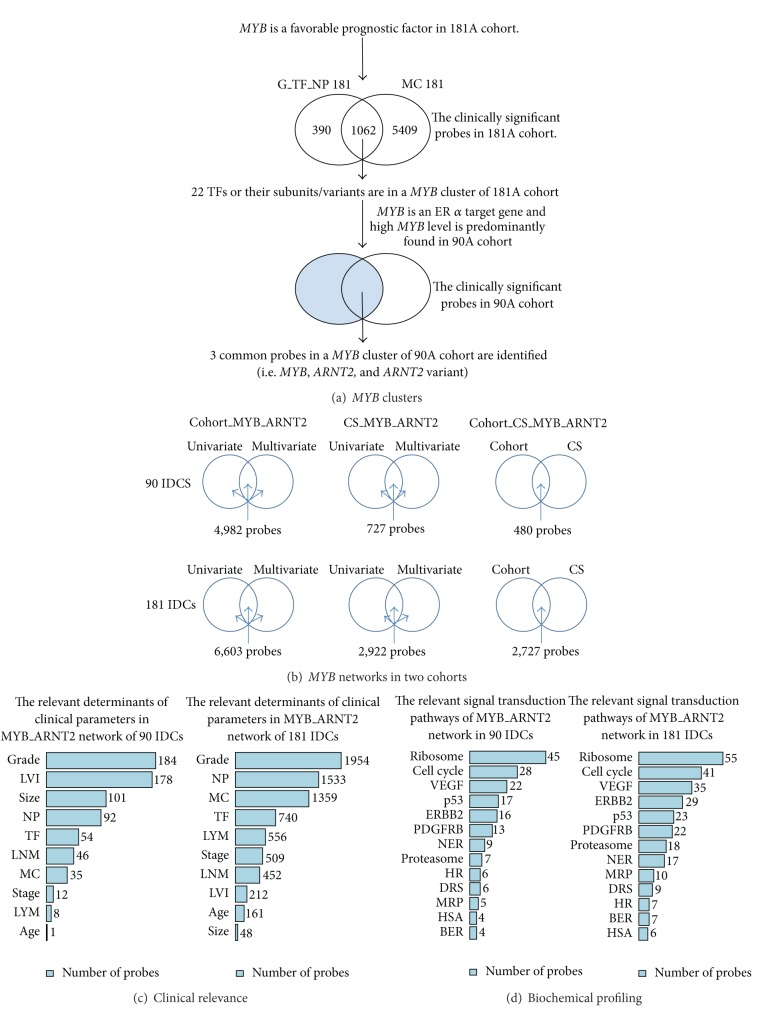
The clinical and/or cohort significance of inferred transcriptional activities of *MYB* and *ARNT2*. It is featured by *MYB *clusters (a),* MYB*_*ARNT2 *transcriptional regulatory networks (b), overlapped gene pools with clinical relevance (c), and overlapped gene pools with the biochemical profiling (d). The signal transduction pathways are derived from Kyoto Encyclopedia of Genes and Genomes (KEGG) database and National Center for Biotechnology Information (NCBI) pathway interaction database.

**Figure 4:**
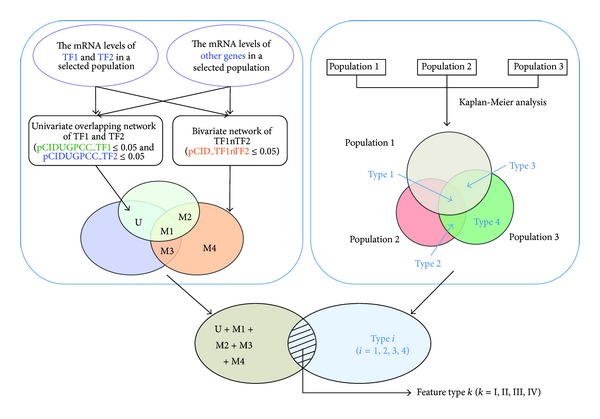
Classification on prognostically relevant subpools of genes in a TF1_TF2 network of the selected population. Step A. We established a full network of TF1 and TF2 consisting of both univariate and multivariate portions of the network in a population (cohort 3). TF2 is an obligate transcription factor partner of TF1 in this case. Therefore, when the gene pools are gathered for further analysis (Step B), they include the gene pools from U and M1–M4 that are demonstrated by a Venn diagram. M1–M4 stand for the expressions of four gene pools following four inferred regulatory mechanisms due to the combinatorial interactions between TF1 and TF2, respectively [11]. U stands for the gene pool in the overlapping network of TF1 and TF2 but without the gene pool derived from M1. Step B. We performed the genome-wide analysis of human breast tumor gene profiles in three cohorts of interest for their prognostic relevance using Kaplan-Meier survival analysis. The prognostic relevant gene pools in three cohorts have four types based on the differential relevance among cohorts 1, 2 and 3 for each prognostic predictor of interest. Each prognostic factor can be grouped into one of four types. We define them as Type *i*  (*i* = 1, 2, 3, and 4). “S” means significant. “NS” means insignificant. Types 1, 2, 3, and 4 are (S, S, S), (NS, S, S), (S, NS, S), and (NS, NS, S), respectively. A Venn diagram demonstrates the subpools of genes in relation with their prognostic relevance in at least one of three selected cohorts. Finally, the overlapped gene subpool between prognostic predictors of a given type and the full network of TF1_TF2 identified in cohort 3 is illustrated by a Venn diagram. In this study, the prognostic relevant genes in a full network of TF1_TF2 can be classified into four feature types. They are designated as feature type *k*  (*k* = I, II, III, and IV).

*MYB* mRNA is highly expressed in ER(+) breast cancers as compared to ER(−) ones (Figure 1(c)). It is elevated especially in group IE subtype (Figure 1(d)). Additionally, *MYB* is an estrogen responsive gene [22] and *ARNT2 *is a xenoestrogen responsive gene [23]. The *in vitro* data using the breast cancer cell model MCF-7 [10, 24] support our network prediction that *ARNT2 *is a *MYB* target gene. Thus, estrogen action on up-regulating activities of *MYB* in coupling with *ARNT2* may be the major feature of favorable prognosis for *MYB*. To address this specific event, we suspect the common gene pool shared by two networks of *MYB*_*ARNT2* with cohort relevance (see 90A cohort and 181 cohort in Figure 3(b)) may uniquely represent the prognostic feature of *MYB* that is not only conserved across subtypes but also enriched in ER(+) IDCs. Only feature type II closely presents this event while comparison was made among four feature types described below.

**Figure 5:**
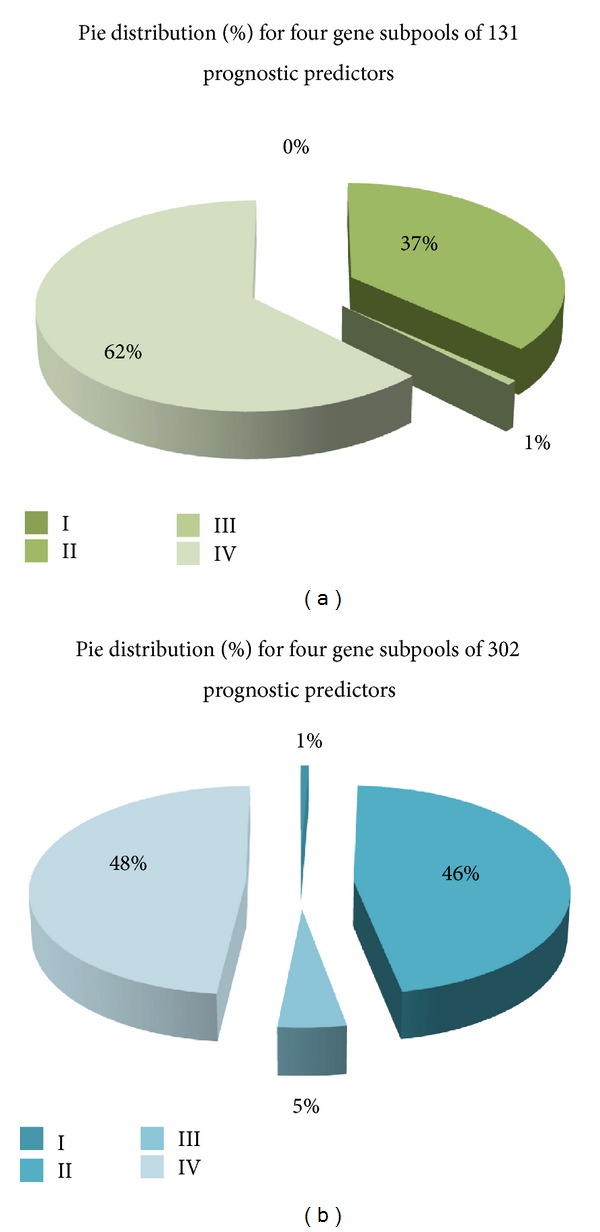
The pie chart for feature distribution of prognostic relevant genes in the network of *MYB_ARNT2*. First, two networks of *MYB_ARNT2* with cohort relevance (90A cohort and 181A cohort) identify 131 probes and 302 probes to be the potential prognostic factors in 90A cohort and 181A cohort, respectively. Second, the pie distribution made for four classified prognostic predictor subpools derived from overlapping between the cohort network of *MYB_ARNT2* and four types of prognostic indicators in three selected populations (91A cohort, 90A cohort, and 181A cohort), respectively. The pie chart demonstrates the sizes of four gene subpools for the given gene pool by its corresponding percentage to be distributed in a pie. The common trend shared by two pie charts is that size of gene subpools in four feature types followed an order of type IV > type II > type III > type I.

Total 131 probes (131/480) in the clinically significant and 90A cohort relevant network of *MYB*_*ARNT2* are identified to be prognostic predictors in at least one of three tested cohorts (90A cohort, 91A cohort, and 181A cohort). They are divided into four feature types (Table S4.1–S4.4). The pie distribution for these four feature types show the predominant groups falling in two cohorts—90A cohort and 181A&90A cohort (Figure 5 and Table 1).

On the other hand, 302 probes (302/2,727) in the clinically significant and 181A cohort relevant network of *MYB*_*ARNT2* are identified to be prognostic predictors in at least one of three tested cohorts (90A cohort, 91A cohort and 181A cohort). They are divided into four feature types (Tables S4.5–S4.8 of Additional file 1). The pie distribution for these four feature types shows the predominant groups falling in two cohorts—181A cohort and 181A&90A cohort (Figure 5 and Table 1).

**Figure 6:**
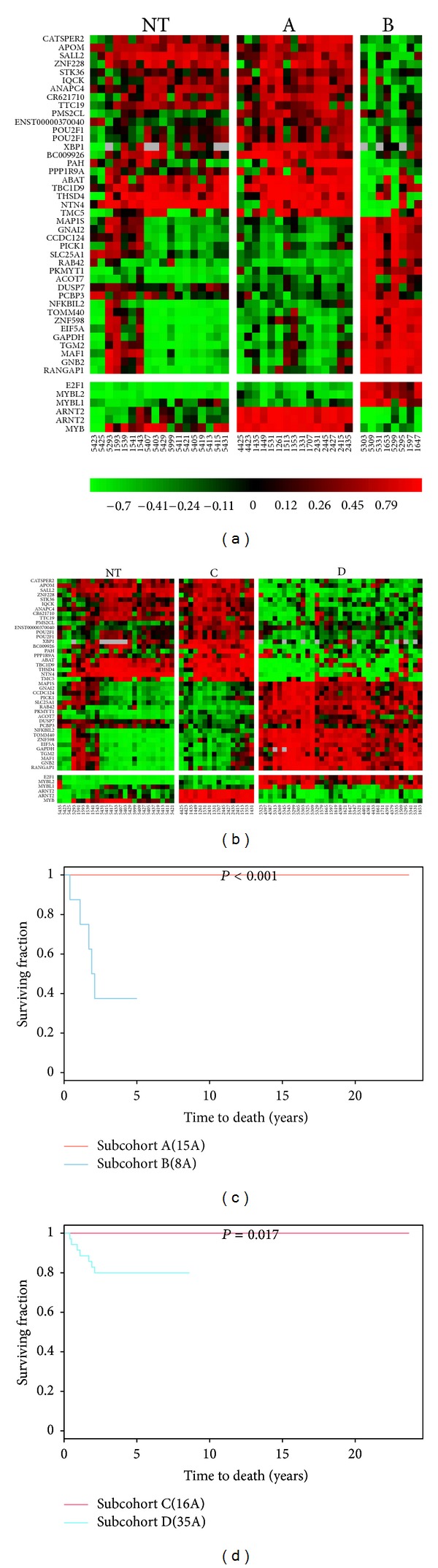
*In vivo* validation on a favorable prognostic signature in subsets of ER(+) IDCs and 181 IDCs. Panel A presents the heatmap displaying forty-one prognostic relevant probes, which are predicted to show the consensus expression dynamics in both 90A cohort and 181A cohort. These 41 probes are part of the most relevant transcriptional activities of both *MYB *and *ARNT2 *in a subset of ER(+) IDCs (a) or in the subset of 181 IDCs predominantly containing ER(+) subtype (b). Panel B demonstrates that the Kaplan-Meier survival curves significantly show the difference in clinical outcomes when comparing subcohorts A(15A)/B(8A) (c) and subcohorts C(16A)/D(35A) (d), respectively. It indicates forty-one probes to be the favorable prognostic signature driven by *MYB* in coupling with *ARNT2*. The tumor samples in subcohorts A/C have relatively higher mRNA levels of *MYB *and *ARNT2 *than those in subcohorts B/D. However, the tumor samples in subcohorts A/C have relatively lower mRNA levels of *MYBL1 *and *MYBL2 *than those in subcohorts B/D. “NT” stands for non-tumor component.

We further examined the heatmaps for a subpool of probes (41 probes) that is the shared subpool of probes in feature type II of the two cohort relevant networks of *MYB*_*ARNT2*  (Figures S8.1 and S8.2). Four selected subsets of patients (subcohorts A, B, C, and D) differentially expressing these 41 probes in a consensus manner within tumor tissues (Table S4.9 and Figure 6) were identified. We further validated the utility of 41 probes in prognosis* in vivo* (*P* < 0.001 for subcohort A versus subcohort B; *P* = 0.017 for subcohort C versus subcohort D in Figure 6). We found a trend of increasing expression levels of *MYBL1* and *L2* when *MYB* expression level becomes low in ER(+) subgroup and/or ER(−) subgroup that results in a poor survival outcome as compared to high *MYB* expressing subgroup (Figures 6(a) and 6(b)). It is likely that the bottom 10% of *MYB* expressing tumors may have the transcription activities shown in subcohorts B and D. We found array IDs 5309, 5343, 5325, 4401, 1711, 4391, and 5335 are within the bottom 10% of *MYB* expressing tumors.

### 3.4. The Annotated Functions of Prognostic Relevant Genes in the *MYB* Transcriptional Regulatory Subnetwork and the Novel Findings

The functional annotations of these 41 probes show that genes are involved in stress, ion channel, phosphorylation, dephosphorylation, transcription, translation, G protein signaling, and metabolism for amino acids and fatty acids according to Gene References into Function (Gene RIFs of NCBI), Gene Spring GX7.3.1, and the related literature. Network analysis indicates the biochemical profiling of those activities (Table S3.15). The function of each probe may not be limited by its current annotated function. *MYB* may differentially regulate those known physiological activities. Some transcription factors may act as the co-regulators of *MYB* to regulate those cellular activities. Importantly, the clinical tumor samples were collected at a time point when they were surgically removed from patients. Therefore, further studies in model systems using time course strategy will be appropriate to validate the roles of *MYB* based on its transcriptional dynamic in relation to the predicted activities described above. Additionally, they are potential factors to increase patient survival rate after receiving conventional cancer treatments and some of them are tumor suppressors (Table 2). Interestingly, the annotated functions of these genes are largely consistent with the published data for the major functional protein groups in *MYB* regulated genes from the human erythroleukemic cell line K562 model [25]. The most interesting finding is the inferred target genes of both *MYB* and *ARNT2* including *POU2F1*, *SALL2,* and *XBP1*. They are transcription factors that are also predicted to be the favorable prognostic predictors in both 90A cohort and 181A cohort (Figures S5.1 and S5.2). They are clinically relevant in early tumor development (Figure S7.1–S7.4 of Additional file 1). *ARNT2*, *POU2F1,* and *XBP1* are in *MYB* signature of MCF-7 [10]. *XBP1* is estrogen responsive [26]. However, high level of *XBP1s* (a splicing variant of *XBP1*) is associated with increased tumor growth, resistance to anti-estrogen therapy, and poor patient survival [27]. *SALL2* is a putative tumor suppressor [28]. *POU2F1* is the transcription factor for proliferation and may promote genomic instability and tumorigenesis in breast cancers [29]. The detailed functions of these TFs in breast tumor development are limited. For example, the mechanisms of how they cooperatively contribute to favorable prognosis will be the important research topics for better understanding of the prognostic features of *MYB*.

The favorable prognostic feature of *MYB* could be simply due to these tumours being more effectively treated, for instance, with Tamoxifen, or that they do not as easily undergo an EMT and metastasis. The lack of clinical treatment data in our model to support our network prediction is a drawback of this study. However, 41-gene signature has been validated by others [30–33].

In this study, we only found *NFKB1L2* to be chemoresistant gene [30] and it is predicted to be down-regulated by *MYB*. *NTN4*, which predicts good prognosis [31], is up-regulated by* MYB*. *PICK1*, which predicts poor prognosis [32], is down-regulated by *MYB*. On the contrary, *TBC1D9*, which is also known as multidrug resistance gene 1(MDR1) [33], is up-regulated by *MYB*. It is still early to conclude the treatment option and response to the treatment based on the 41-prognostic gene signature *in vivo*. First, the cohort study of ours is only a training set. We need an appropriate testing set to validate its reproducibility. Second, the cancer treatment data for the cohort of ours is incomplete based on the medical record. Third, the* in vitro* and *in vivo* studies of the gene signature at protein level and its relation to cancer treatments would be necessary to conclude genes for the cancer treatment option(s) and response(s) to cancer treatment(s).

Here, we claim that 41-gene signature is different from other published signatures due to a supervised network analysis approach. First, each functional transcription factor (e.g., *MYB*) has its own transcriptional mechanisms predicted by network analysis. Network analysis allows dissecting *MYB* activities by its transcriptional regulatory network. Second, a supervised network analysis has identified a potential prognostic relevant signature of *MYB* and *ARNT2* (i.e., 41-gene signature). The network analysis is a qualitative method. We observed that the expression levels of *MYB* inferred target genes vary a lot. As such, some probes in the 41-gene signature are not clinically significant. For example, *CR621710*, *TBC1D9*, *ZNF598* and *GAPDH* within the 41-gene signature are not clinically significant in 90A cohort (Table S5.1). *PPP1R9A* and *GNAI2* (Table S5.2) show no significant clinical impact in 181A cohort. Additionally, most of them (39/41) have their clinical significance to be shifted away from the clinical characteristics of *MYB* and *ARNT2* in 181A cohort (39/41) and in 90A cohort (37/41) (Tables S5.1-S5.2). The clinicopathological characteristics of subcohorts A, B, C, and D are partially overlapped (Table S5.3). This indicates the favorable prognostic activities of *MYB* and *ARNT2* to be preferentially at early tumor development but may be extended to the later event. Likewise, the late tumor development overlapping with a few early clinicopathological events is found in tumor samples with both suppressed activities of *MYB* and *ARNT2*. Importantly, the annotated activities of the 41-gene signature are similar to the common gene activities of *MYB in vitro* [25]. The transcriptional dynamic of this prognostic signature has shown to be across molecular subtypes but enriched in ER(+) IDCs (Figure 6). However, Table S5.4 shows those univariate COXPH analyses of subcohort A/B, subcohort A/nonA, and nine major traditional prognostic factors in 90A cohort to be not significant. Likewise, those of subcohort C/D, subcohort C/nonC, and nine major traditional prognostic factors in 181A cohort are not significant. This indicates the 41-gene signature to be not prognostic relevance in a subset of ER(+) IDCs showing transcriptional dynamic of this gene signature (i.e., subcohort A/B or subcohort C/D) and in those showing early tumor development with the 41-gene signature versus other gene expression patterns of the 41-gene signature in both 90A cohort and 181A cohort. Moreover, the 41-gene signature is not an independent prognostic factor in subcohort A/B, subcohort C/D, subcohort A/nonA and subcohort C/nonC based on multivariate COXPH analysis in both 90A cohort and 181A cohort. Importantly, only 181A cohort shows the traditional prognostic factors, LVI, size, LNM, stage, and LYM, to be prognostic relevant. Typically, LNM is the independent prognostic factor when comparison was made among tested prognostic factors.

We suspect that both univariate and multivariate COX proportional hazard (COXPH) analyses for this signature show not significant (Table S5.4) due to the unique regulatory mechanisms of *MYB* in coupling with *ARNT2* and the small *N* number for those tested cohorts. However, based on the rationale of supervised network analysis, we observed that Kaplan-Meier survival analysis predicts the prognostic significance of 41-gene signature in a subset of IDCs (Figure 6). Further investigations in a large population to evaluate the reproducibility of this favorable prognostic signature would be necessary.

### 3.5. The Clinical Roles of *MYB* Family Members in 90A Cohort and 181A Cohort

*MYB* family members—*MYB*, *MYBL1* and *MYBL2* have been studied in breast cancers. But, the genome-wide regulatory mechanisms for these TFs to their shared target genes in breast cancers are largely unknown.

#### 3.5.1. The Clinical Impacts of *MYB* Family Members

ANOVA tests on *MYB* for its clinical impact in 90A cohort and 181A cohort suggest its role in early tumor development. However, the expression levels of *MYBL1* and *MYBL2* are increased during later development of breast cancers.

High mRNA levels of *MYBL1* are the determinants of LNM, size, HER, G, NP, and MC in late breast tumor development (Figure S7.5). Increased mRNA levels of *MYBL2* are significantly associated with late LVI, PR, LNM, G, TF, NP and MC (Figure S7.6). ANOVA test shows *MYBL1* to be a promoter for the late clinicopathological progression of ER(+), ER(−), and 181 IDCs. *MYBL2* is a promoter for the late clinicopathological progression of both ER(+) IDCs and 181 IDCs.

#### 3.5.2. The Prognostic Values of *MYB* Family Members

*MYB* is predicted to be a favorable prognostic indicator in 181A cohort (Figure 1(e)). However, elevated* MYBL1* and *MYBL2* in 90A cohort predict poor clinical outcome, respectively (Figure S5.1). The preferential poor prognostic activities of *MYBL1 *and *MYBL2* are also indicated in ER(−) IDCs (*n* = 25) of subcohort D. Typically, we found it in two ERBB2 IDCs (array IDs 5305 and 1711) (Figure 6(b)). However, the prognostic feature of *MYBL1* and *L2* is less significant in ER(−) cohort (data not shown). This may be because the majority of those ER(−) IDCs were analyzed for their survival outcomes when they were less than 5 years from the first diagnosis. Therefore, the follow-up survival analysis to find out the prognostic features of* MYBL1* and *L2 *in ER(−) IDCs will be needed in the future.

The recent research evidence supports our finding that *MYB* is a potential favorable prognostic factor in luminal breast cancer [10]. Thorner et al. [6] demonstrated that increased *MYBL2 *expression is a significant predictor of poor survival and pathological complete response to neoadjuvant chemotherapy (e.g., DNA topoisomerase II *α* (TOP2A) inhibitors—doxorubicin and etoposide) in basal-like breast cancer. The *MYBL2* has been discovered as one of recurrence risk genes in tamoxifen-treated, node-negative breast cancer [34]. *MYBL1* is a transcription factor that is involved in mammary gland development [5]. It may play roles in the biology and/or pathogenesis of some neoplasia [35, 36]. Recent report mentioned *MYBL1* to be an oncogene [37].

#### 3.5.3. The Predicted Overlapping Networks of *MYB* Family Members in Breast Cancers in relation to Their Prognostic Features

**Figure 7:**
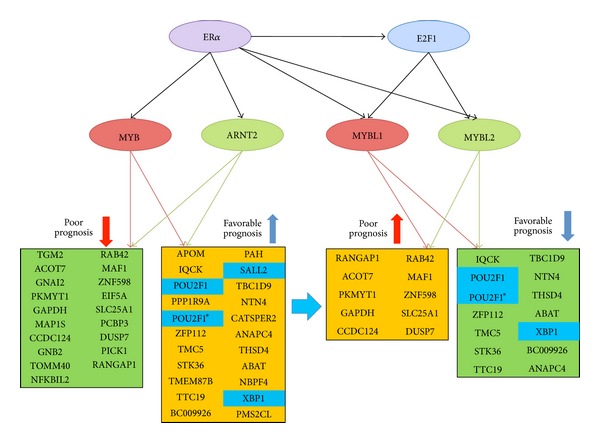
The inferred prognostic predictors partially antagonized by *MYBL1* and *MYBL2* during late tumor progression. This event is proposed to be differentially controlled by ER*α* and/or ER*α*_E2F1 promoter use pathways. Here, the novel predicted role of *MYBL1* and *MYBL2* may provide part of mechanism for *MYBL1* and *MYBL2* being the unfavorable prognostic predictors in a subset of ER(+) IDCs. The increased levels of both *MYBL1* and *MYBL2* are frequently accompanied with low expression levels of *MYB* and *ARNT2* observed in these tumor samples. There are twenty-four probes in this signature predicted to be shared target genes of both *MYBL1 *and *MYBL2*.

The c-Myb (MYB) protein was found to be associated with over 10,000 promoters as the evidence of being a master transcription factor in MCF-7 cell model [24]. *MYB*, *MYBL1* and *MYBL2* have different regulatory mechanisms but share the conserved DNA binding domain that strongly suggests the compensatory effects within family members in regulating their shared target genes [38, 39]. As such, we further analyze the shared target genes of *MYBL1*, *MYBL2, *and *MYB *in relation to their prognostic features (Table S4.10). As a result, this is the first time it was reported that *MYBL1* and *MYBL2* may partially antagonize the action of 24 probes in the favorable prognosis signature that are predicted to be regulated by *MYB* and *ARNT2* (Table 2 and Figure 7). Moreover, our data suggests that both *MYBL1* and *MYBL2* are predicted to be the shared target genes of E2F1 and ER*α*. The promoter region of *MYBL2 *has E2F1/3 binding site [40]. However, the current report showed the major regulatory element in *MYBL1* promoter region to be Sp1 sites and CCAT box (a NF-Y binding site) [41]. Further investigation in the cell model would be of interest to validate the novel findings of ours that the *E2F1* may regulate *MYBL1* expression. *MYBL1* and *MYBL2 *are estrogen responsive genes [34, 37]. They (E2F1, MYBL1, and MYBL2) are poor prognostic factors in 90A cohort (Figure S5.1). We, therefore, map the transcriptional activities of MYB family members and their gene partners during breast tumor development (Figure 7). Based on this hypothesized mechanism, the estrogen action on MYB family members at different time point of disease development may be shown by the ER*α* and ER*α*_E2F1 promoter use pathways. There are forty-one probes as the inferred target genes of *MYB* and *ARNT2*. *MYB* may actively suppress oncogenic activities of *NFKBIL2* [30], *GAPDH* [42], *RAB42* [43]*, EIF5A *[44], and *PICK1 *[32]. Additionally, *MYB* may promote good prognosis via up-regulating *NTN4* and *SALL2*. *NTN4* is a good prognostic factor [31]. *SALL2* is a putative tumor suppressor [28].

On the contrary, there are only twenty-four probes as the candidate target genes of *MYBL1* and *MYBL2* (Table 2). We have briefly evaluated some evidence (see Table 2) that may support the possible poor prognostic features of *MYBL1* and *MYBL2* and may offer new strategies in treating a subset of advanced ER(+) breast cancer expressing high levels of *MYBL1* and *MYBL2*. For instance, two suggested targets for cancer treatment, *GAPDH *and *RANGAP1*, are predicted to be up-regulated by *MYBL2* and *MYBL1*. Both *GAPDH* and *RAB42* (the RAS oncogene family member) may be up-regulated by these two TFs to promote oncogenic activities. *PKMTY1* is a serine/threonine protein kinase and a cell cycle regulatory gene that is predicted to be up-regulated by *MYBL2* and *MYBL1 *suggesting increased cell proliferating activities [45].

### 3.6. The Undiscovered Transcriptional Activities and Interaction among *MYB* Family Members

Our preliminary data on the clinical roles of *MYBL1* and *MYBL2* in ER(−) and ER(+) breast cancers (Figures S7.5 and S7.6) suggest that they are important to be further investigated in the future studies. Their interactions with *MYB* in different subtypes and in different clinicopathological statuses may alter the prognostic features of *MYBL1* and *MYBL2* in a subset of breast cancer population. Multiple drug targets for genes resistant to standard cancer therapies may be uncovered to aid with the prognostication of a subset of breast cancer patients and with alternate treatment options at the time of diagnosis.

## 4. Conclusions

*MYB* predicts a favorable prognosis across molecular subtypes of infiltrating ductal breast carcinomas but enriched in ER(+) IDCs. This specific event can be linked with a 41-gene prognostic signature or a core subnetwork of *MYB_ARNT2. *The supervised analysis for constructing an inferred transcriptional regulatory network is efficient and inexpensive. To our best knowledge, this signature is not the same as other published signatures that have been described in a recent review [46] due to different method and the supervised approach. It is predicted to fill in the gap between the traditional clinical prognostic factors and other published prognostic signatures. However, this may be true only for a subset of population (approximately 10% of a cohort) who obtain not only the most relevant dynamic changes of gene expression pattern for the selected gene set but also significance in the Kaplan-Meier survival analysis. Together, such experimental design may offer the opportunity for the personalized medicine to be discovered by the supervised network analysis.

*MYB* governs a large pool of target genes based on network analyses. We observed that *MYB* has an essential partner gene—*ARNT2*—that is low in non-tumor component but is up-regulated in breast tumors. Both transcription factors may coordinately suppress 13 cancer-related signal transduction pathways and some clinicopathological progression (<10 clinical parameters) in breast tumors via differentially regulating their shared target genes. This indicates the major clinical impact of both *MYB* and *ARNT2* to be tumor suppressive during early tumor development.

The functional annotated 41-gene prognostic signature indicates the major contributors associated with the prognostic features of *MYB* including up-regulating the transcriptional activities of three transcription factors—*POU2F1*, *SALL2,* and *XBP1* which are also favorable prognostic indicators in 90A cohort and 181A cohort. Silencing both *MYB* and *ARNT2* in 90 IDCs reveals an increase in expression levels of some unfavorable prognostic predictors. They include *E2F1*, *MYBL1,* and *MYBL2*. These transcription factors may partially antagonize the favorable activities of both *MYB* and *ARNT2* to lead the poor clinical outcome of a subset of patients. Importantly, knockdown of the transcriptional activities of *E2F1*, *MYBL1,* and *MYBL2* may be considered as the suggested treatment targets to improve prognosis for a subset of breast cancer population with ER(−) or with advanced ER(+) breast cancers who have elevated *E2F1*, *MYBL1* and *MYBL2 *in their breast tumors.

From this limited study, we only predict the major prognostic features of *MYB* with *in vivo* validation of 41-gene prognostic signature in a breast cancer model system. The detailed mechanisms of actions for MYB family in cancer development involving other transcription factor partners, such as *SALL2*, *XBP1,* and *POU2F1*, are still not clear. Further research to elucidate the roles of *MYB* family members in breast cancers in depth is necessary, such as in the large patient population studies and in studies using different* in vivo* and *in vitro* models.

## Supplementary Material

There are eight supplemental files gathered to be the additional file 1. We collected the Venn diagram figures and their corresponding gene pools to form the unique table. It consists of supplemental files 1-4. The major results of survival analyses are listed in supplemental file 5. We have gathered the key results of the network analyses in supplemental file 6. It includes the clinical relevant gene profiling, the biochemical profiling and the partially validated results of the network analysis. We put the ANOVA test results of a few important transcriptions factors for showing their clinical impacts in supplemental file 7. The gene expression patterns of the 41-gene signature in two subtypes of ER(+) IDCs and 181 IDCs are shown in supplemental file 8.

## Figures and Tables

**Table:** (a) 90A cohort

Feature type	90A cohort	91A cohort	181A cohort
I	0	0	0
II	48	0	48
III	1	1	0
IV	82	0	0

Total # probes	131	1	48

**Table:** (b) 181A cohort

Feature type	90A cohort	91A cohort	181A cohort
I	2	2	2
II	140	0	140
III	0	14	14
IV	0	0	146

Total # probes	142	16	302

**Table 2:** Functional annotation and transcriptional regulation patterns of the prognostic relevant signature.

Feature no.	Gene symbol	Regulated by MYB	Regulated by MYBL1 & L2	Biological function(s) and/or cancer-related activities
2654	APOM	Up	—	Protein
714	IQCK	Up	Down	SRC-3 binding protein
9472	POU2F1	Up	Down	TF
20014	PPP1R9A	Up	—	Phosphatase
11991	POU2F1	Up	Down	TF
7164	ZFP112(ZNF228)	Up	Down	Zinc finger protein
4051	TMC5	Up	Down	Transmembrane channel-like protein
9673	STK36	Up	Down	Serine/threonine kinase
1509	TMEM87B(CR621710)	Up	—	Transmembrane protein
6481	TTC19	Up	Down	Roles in protein-protein interactions
10396	BC009926	Up	Down	The inner mitochondrial membrane protein
20295	PAH	Up	—	amino acid metabolism
3096	SALL2	Up	—	TF and putative tumor suppressor
17750	TBC1D9	Up	Down	Multidrug resistance gene 1(MDR1)
20917	NTN4	Up	Down	Good prognostic factor
8570	CATSPER2	Up	—	Ion channel
6691	ANAPC4	Up	Down	Chromosome replication
10334	THSD4	Up	Down	A disintegrin and metalloproteinase
9326	ABAT	Up	Down	Aminotransferase
15762	NBPF4(ENST00000370040)	Up	—	Undefined function
10024	XBP1	Up	Down	TF
10998	PMS2CL	Up	—	Mismatch repair gene
11119	TGM2	Down	—	Metabolism
3069	ACOT7	Down	Up	Fatty acid metabolism
1039	GNAI2	Down	—	G protein
19802	PKMYT1	Down	Up	Kinase
20037	GAPDH	Down	Up	Candidate target for cancer treatment; proliferation and metastasis; glycolysis.
5918	MAP1S	Down	—	Morphology; microtubule associated protein
956	CCDC124	Down	Up	Undefined function
21198	GNB2	Down	—	G protein
13619	TOMM40	Down	—	Enzyme
18840	NFKBIL2	Down	—	A negative regulator of NFKB mediated transcription; the maintenance of genome stability; may cause chemoresistance
4681	RAB42	Down	Up	Putative Ras-related protein related to cell proliferation
3891	MAF1	Down	Up	Control transcription initiation
21760	ZNF598	Down	Up	Undefined function
17698	EIF5A	Down	—	Translation
7939	SLC25A1	Down	Up	Cellular component
7824	PCBP3	Down	—	Posttranscriptional activities
16475	DUSP7	Down	Up	Phosphatase
17776	PICK1	Down	—	Signaling molecule; poor prognosis; promote tumor growth
3073	RANGAP1	Down	Up	G protein signaling; a new target for cancer chemotherapy

## Abbreviations

ABAT: 4 Aminobutyrate aminotransferase
ACOT7: Acyl-CoA thioesterase 7
ANAPC4: Anaphase promoting complex subunit 4
APOM: Apolipoprotein M
ARNT2: Aryl-hydrocarbon receptor nuclear translocator 2
BER signaling: Signal transduction pathway of base excision repair (BER)
CATSPER2: Cation channel, sperm associated 2
CCDC124: Coiled-coil domain containing 124
Cell cycle signaling: Signal transduction pathway of cell cycle
Cohort: A group of individuals in a population all with the features suitable for the study of interest or a given population
DR signaling: Signal transduction pathway of DNA replication (DR)
DUSP7: Dual specificity phosphatase 7
E2F1: E2F transcription factor 1
EIF5A: Eukaryotic translation initiation factor 5A
ER: Estrogen receptor *α*
ERBB2 signaling: Signal transduction pathway of v-erb-b2 erythroblastic leukemia viral oncogene homolog 2, neuro/glioblastoma derived oncogene homolog (avian) (ERBB2)
ESR1: Estrogen receptor 1
GAPDH: Glyceraldehyde-3-phosphate dehydrogenase
GNAI2: Guanine nucleotide binding protein (G protein), alpha inhibiting activity polypeptide 2
GNB2: Guanine nucleotide binding protein (G protein), beta polypeptide 2
Grade: Histological grade
Group IE: ER(+)PR(+)
Group IIE: ER(+)PR(−)
HER: HER-2/neu
HR signaling: Signal transduction pathway of homologous recombination (HR)
HS*Α* signaling: Signal transduction pathway of $hsa03450$ (HS*Α*)
IQCK: IQ motif containing K
LNM: Number of lymph node metastasis
LVI: Lymphovascular invasion
LYM: Lymph node metastasis status
MAF1: MAF1 homolog (*S. cerevisiae*)
MAP1S: Microtubule-associated protein 1S
MC: Mitotic count
MRP signaling: Signal transduction pathway of mismatch repair pathway (MRP)
MYB/C MYB: v-myb myeloblastosis viral oncogene homolog (avian)
MYBL1/A MYB: v-myb myeloblastosis viral oncogene homolog (avian)-like 1
MYBL2/B MYB: v-myb myeloblastosis viral oncogene homolog (avian)-like 2
NBPF4: Neuroblastoma breakpoint family, member 4
NER signaling: Signal transduction pathway of nucleotide excision repair (NER)
NFKBIL2: Nuclear factor of kappa light polypeptide gene enhancer in B-cells inhibitor-like 2
NP: Nuclear pleomorphism
NTN4: Netrin 4
p53 signaling: Signal transduction pathway of p53
PAH: Phenylalanine hydroxylase
PCBP3: Poly(rC) binding protein 3
PDGFRB signaling: Signal transduction pathway of platelet-derived growth factor receptor, beta polypeptide (PDGFRB)
PICK1: Protein interacting with PRKCA 1
PKMYT1: Protein kinase, membrane associated tyrosine/threonine 1
PMS2CL: PMS2 C-terminal like pseudogene
POU2F1: POU class 2 homeobox 1
PPP1R9A: Protein phosphatase 1, regulatory (inhibitor) subunit 9A
PR: Progesterone receptor
Proteasome: Signal transduction pathway of proteasome
RAB42: RAB42, member RAS oncogene family
RANGAP1: Ran GTPase activating protein 1
Ribosome: Signal transduction pathway of ribosome
SALL2: Sal-like 2 (*Drosophila*)
Size: Tumor size
SLC25A1: Solute carrier family 25 (mitochondrial carrier; citrate transporter), member 1
STK36: Serine/threonine kinase 36, fused homolog (Drosophila)
TBC1D9: TBC1 domain family, member 9 (with GRAM domain)
TF: Tubule formation
TGM2: Transglutaminase 2 (C polypeptide, protein-glutamine-gamma-glutamyltransferase)
THSD4: Thrombospondin, type I, domain containing 4
TMC5: Transmembrane channel-like 5
TMEM87B: Transmembrane protein 87B
TOMM40: Translocase of outer mitochondrial membrane 40 homolog (yeast)
TTC19: Tetratricopeptide repeat domain 19
VEGF signaling: Signal transduction pathway of vascular endothelial growth factor (VEGF)
XBP1: X-box binding protein 1
ZFP112: Zinc finger protein 112 homolog (mouse)
ZNF598: Zinc finger protein 598.
