# Supplementary material for: A Supervised Network Analysis on Gene Expression Profiles of Breast Tumors Predicts a 41-Gene Prognostic Signature of the Transcription Factor MYB across Molecular Subtypes
Source: Comput Math Methods Med. 2014 Feb 3;2014:813067. doi: 10.1155/2014/813067 (PMC3930188; doi:10.1155/2014/813067)

Additional file 1 Liu *et al.* 2013

Supplementary information

Title: A supervised network analysis on gene expression profiles of breast tumors predicts a 41- gene prognostic signature of the transcription factor *MYB* across molecular subtypes

Table of content (Pages 1-8)

Suppls.1-4 are summarized gene pools in four combined illustrations with sets of their corresponding Venn diagrams. An unique table consists of suppl.1, 2, 3 and 4. The listed gene symbols are transcription factors and components of interest within this table except eight hundred ninety one of the gene symbols are left as "blank" within the table based on annotation provided by Gene Spring GX7.3.1 (March, 2011).The gene symbols high-lighted with light blue are transcription factors, transcription factor subunits and transcript variants of transcription factors. The remaining gene symbols are molecules of interest in this study. We only mark "Y" in a row of the assigned column that means the gene symbol to be one of the components listed in the analysis of interest. The gene symbols in the Venn diagrams are defined as follows. The gene symbols written by lower cases that are the gene probe names for gene probes having more than one transcripts. The gene symbols written by upper cases that are the gene names derived either from combined gene probe names or from the gene with only one corresponding transcript. For instance, *ARNT2* consists of two probe names – arnt2 and arnt2.1. arnt2.1 is a transcript variant of arnt2 (data not shown). *MYB* stands for a gene with a corresponding transcript.

Suppl. 5 has the prognostic values of transcription factors in the *MYB* subnetworks (Figure 7 and Table S4.10) in 90A cohort (groups IE and IIE) and 181A cohort, respectively.

Suppl. 6 contains heatmaps for (1) the common gene pools shared by six signal transduction pathway and the network of *MYB_ARNT2* in 90A cohort, respectively. (2) the common gene pools shared by three relevant clinical parameters and the clinically significant network of *MYB_ARNT2* in 90A cohort, respectively.

Suppl. 7 includes the meanplots for mRNA levels for *POU2F1*(9472), *POU2F1*(11991), *SALL2*(3096), *XBP1*(10024), *MYBL1*(C11830) and *MYBL2*(10757) in eight clinical categories and in three cohorts of infiltrating ductal carcinoma (IDCs), respectively.

Suppl. 8 shows the gene expression pattern of the prognostic relevant signature (41

probes) in relation to the gene expression pattern of four transcription factors in five cohorts, respectively.

Suppls. 1-4 (Pages 9-186)

Suppl. 1

Table S1.1. Clinicopathological significant cluster of histological grade (grade), tubule formation (TF) and nuclear pleomorphism (NP) in 181 infiltrating ductal breast carcinomas.

Table S1.2. Clinicopathological significant cluster of mitotic count, grade, TF and NP in 181 infiltrating ductal breast carcinomas.

Table S1.3. The overlapping network of *MYB* and *ARNT2* predicted to be relevant in 61 group IE and 91 ER(-) infiltrating ductal breast carcinomas.

Table S1.4. The overlapping network of *MYB* and *ARNT2* predicted to be relevant in 29 group IIE and 91 ER(-) infiltrating ductal breast carcinomas.

Table S1.5. The overlapping network of *MYB* and *ARNT2* predicted to be relevant to grade, MC, NP and TF in 181 infiltrating ductal breast carcinomas.

Table S1.6. The overlapping network of *MYB* and *ARNT2* predicted to be relevant to both ER(-) and two subtypes of ER(+) infiltrating ductal breast carcinomas.

Table S1.7. The overlapping bivariate network of *MYB* and *ARNT2* predicted to be relevant to two subtypes of ER(+) - groups IE and IIE and to 91 ER(-) infiltrating ductal breast carcinomas.(5,158 probes)

Table S1.8. The MYB_ARNT2 network derived from combining both univariate (Table S1.6) and bivariate (Table S1.7) networks of *MYB* and *ARNT2* relevant to 181 infiltrating ductal breast carcinomas. (6,603 probes)

Table S1.9. The MYB_ARNT2 network derived from combining both univariate (Table S1.5) and bivariate (Table S1.12) networks of *MYB* and *ARNT2* relevant to clinicaopathological parameters in 181 infiltrating ductal breast carcinomas. (2,922 probes)

Table S1.10. The MYB_ARNT2 network not only relevant to grade, NP, TF and MC (Table S1.9) but also to 181 infiltrating ductal breast carcinomas (Table S1.8). (2,727probes)

Table S1.11. The overlapping bivariate network of *MYB* and *ARNT2* predicted to be relevant grade, MC and NP in 181 infiltrating ductal breast carcinomas.

Table S1.12. The overlapping bivariate network of *MYB* and *ARNT2* predicted to be relevant grade, MC, NP and TF in 181 infiltrating ductal breast carcinomas.

Suppl. 2

Table S2.1. Clinicopathological significant cluster of grade, LVI and TF in 90 infiltrating ductal breast carcinomas.

Table S2.2. Clinicopathological significant cluster of grade, LVI, TF, NP and tumor size (size) in 90 infiltrating ductal breast carcinomas.

Table S2.3. Clinicopathological significant cluster of grade, LVI, TF, NP, tumor size (size) and LNM in 90 infiltrating ductal breast carcinomas.

Suppl. 3

Table S3.1. The overlapping network of *MYB* and *ARNT2* relevant to histological grade in 90 ER(+) infiltrating ductal breast carcinomas.

Table S3.2. The overlapping network of *MYB* and *ARNT2* relevant to LNM in 90 ER(+) infiltrating ductal breast carcinomas.

Table S3.3. The overlapping network of *MYB* and *ARNT2* relevant to LVI in 90 ER(+) infiltrating ductal breast carcinomas.

Table S3.4. The overlapping network of *MYB* and *ARNT2* relevant to NP in 90 ER(+) infiltrating ductal breast carcinomas.

Table S3.5. The overlapping network of *MYB* and *ARNT2* relevant to tumor size in 90ER(+) infiltrating ductal breast carcinomas.

Table S3.6. The overlapping network of *MYB* and *ARNT2* relevant to TF in 90 ER(+) infiltrating ductal breast carcinomas.

Table S3.7. The overlapping network of *MYB* and *ARNT2* relevant to grade, LNM and LVI in 90 ER(+) infiltrating ductal breast carcinomas.

Table S3.8. The overlapping network of *MYB* and *ARNT2* relevant to grade, LNM, LVI, NP and TF in 90 ER(+) infiltrating ductal breast carcinomas.

Table S3.9. The overlapping network of *MYB* and *ARNT2* relevant to grade, LNM, LVI, NP, TF and size in 90 ER(+) infiltrating ductal breast carcinomas.

Table S3.10. The overlapping univariate network of *MYB* and *ARNT2* relevant to an ER(+) subtype – group IE in 90 ER(+) infiltrating ductal breast carcinomas.

Table S3.11. The overlapping univariate network of *MYB* and *ARNT2* relevant to an ER(+) subtype – group IIE in 90 ER(+) infiltrating ductal breast carcinomas.

Table S3.12. The overlapping univariate network of *MYB* and *ARNT2* relevant to two ER(+) subtypes – groups IE and IIE in 90 ER(+) infiltrating ductal breast carcinomas.

Table S3.13. The MYB_ARNT2 network derived from combining both univariate (Table S3.12) and bivariate (Table S3.19) networks of *MYB* and *ARNT2* relevant to two ER(+) subtypes – groups IE and IIE in 90 ER(+) infiltrating ductal breast carcinomas. (4,982 probes)

Table S3.14. The MYB_ARNT2 network derived from combining both univariate (Table S3.9) and bivariate (Table S3.19) network of *MYB* and *ARNT2* relevant to grade, NP, TF, LNM, LVI and tumor size in 90 ER(+) infiltrating ductal breast carcinomas. (727 probes)

Table S3.15. The MYB_ARNT2 network not only relevant to grade, NP, TF, LNM, LVI and tumor size (Table S3.14) but also to two ER(+) subtypes – groups IE and IIE (Table S3.13) in 90 ER(+) infiltrating ductal breast carcinomas. (480 probes)

Table S3.16. The overlapping bivariate network of *MYB* and *ARNT2* relevant to grade, NP and TF in 90 ER(+) infiltrating ductal breast carcinomas.

Table S3.17. The overlapping bivariate network of *MYB* and *ARNT2* relevant to grade, NP, TF, LNM and LVI in 90 ER(+) infiltrating ductal breast carcinomas.

Table S3.18. The overlapping bivariate network of *MYB* and *ARNT2* relevant to grade, NP, TF, LNM, LVI and tumor size in 90 ER(+) infiltrating ductal breast carcinomas.

Table S3.19. The overlapping bivariate network of *MYB* and *ARNT2* relevant to two ER(+) subtypes – groups IE and IIE in 90 ER(+) infiltrating ductal breast carcinomas.

Suppl. 4

Table S4.1. The overlapping gene pool between network of 90_CS_MYB_ARNT2 and the prognosis predictors significant in 181, 91 and 90 infiltrating ductal breast carcinomas (or network of Feature type I_90_CS_MYB_ARNT2). Surv. 90 stands for probes to be significant in Kaplan-Meier survival analysis for 90A cohort. Likewise, Surv. 91 and Surv. 181 are based on such definition except in different cohorts.

Table S4.2. The overlapping gene pool between network of 90_CS_MYB_ARNT2 and prognosis predictors significant in both 181 and 90 infiltrating ductal breast carcinomas (or network of Feature type II_90_CS_MYB_ARNT2). Not Surv. 181 stands for probes to be not significant in Kaplan-Meier survival analysis for 181A cohort.

Table S4.3. The overlapping gene pool between network of 90_CS_MYB_ARNT2 and prognosis predictors significant in both 90 and 91 infiltrating ductal breast carcinomas (or network of Feature type III_90_CS_MYB_ARNT2).

Table S4.4. The overlapping gene pool between network of 90_CS_MYB_ARNT2 and prognosis predictors significant in 90 infiltrating ductal breast carcinomas (or network of Feature type IV_90_CS_MYB_ARNT2).

Table S4.5. The overlapping gene pool between network of 181_CS_MYB_ARNT2 and prognosis predictors significant in 91, 181 and 90 infiltrating ductal breast carcinomas (or network of Feature type I_181_CS_MYB_ARNT2).

Table S4.6. The overlapping gene pool between network of 181_CS_MYB_ARNT2 and prognosis predictors significant in both 181 and 90 infiltrating ductal breast carcinomas (or network of Feature type II_181_CS_MYB_ARNT2).

Table S4.7. The overlapping gene pool between network of 181_CS_MYB_ARNT2 and prognosis predictors significant in both 181and 91 infiltrating ductal breast carcinomas (or network of Feature type III_181_CS_MYB_ARNT2).

Table S4.8. The overlapping gene pool between network of 181_CS_MYB_ARNT2 and prognosis predictors significant in 181 infiltrating ductal breast carcinomas (or network of Feature type IV_181_CS_MYB_ARNT2).

Table S4.9. The overlapping gene pool between network of Feature type II_90_CS_MYB_ARNT2 and network of Feature type II_181_CS_MYB_ARNT2.

Table S4.10. The overlapping gene pool between 41 prognostic predictors and the common gene pool shared by the networks of *MYBL1* and *MYBL2* in 90A cohort.

Suppl. 5. (Pages 187-194)

Figure S5.1. The prognostic prediction of E2F1(7852), MYB(5586), MYBL1(C11830), MYBL2(10757) and XBP1(10024) in different cohorts (90A cohort, 181A cohort) by Kaplan-Meier survival analysis.

Figure S5.2. The prognostic prediction of ARNT2(3742), ARNT2(3187), POU2F1(947), POU2F1(11991), and SALL2(3096) in different cohorts (90A cohort, 181A cohort) by Kaplan-Meier survival analysis.

Table S5.1. The ANOVA results of 41-gene signature in 90A cohort.
The relevant clinicopathological characteristics for *MYB* and/or *ARNT2* are bolded. The p values of ANOVA tests to be less or equal to 0.05 are high-lighted with light blue. The gene symbols with no clinical significance are high-lighted with light yellow.

Table S5.2. The ANOVA results of 41-gene signature in 181A cohort.
The relevant clinicopathological characteristics for *MYB* and/or *ARNT2* are bolded. The p values of ANOVA tests to be less or equal to 0.05 are high-lighted with light blue. The gene symbols with no clinical significance are high-lighted with light yellow.

Table S5.3. Clinicopathological characteristics of four subcohorts of infiltrating ductal breast carcinomas.

Table S5.4. Univariate and multivariate analyses for survival on prognostic factors in 90A and 181A cohorts, respectively. The p values of t test in the COX proportional hazard model to be less or equal to 0.05 are high-lighted with light blue. The definitions for subcohorts A, B, C and D are in Figure 6 (see main text). Subcohort nonA stands for 90A cohort without subcohort A. Subcohort nonC stands for 181A cohort without subcohort C.

Suppl. 6. (Pages 195-201)

Figure S6.1. Heatmaps for the common gene pool in both cell cycle signal transduction pathway and the network of *MYB_ARNT2* in 90A cohort.

Figure S6.2. Heatmaps for the common gene pool in both VEGF signal transduction pathway and the network of *MYB_ARNT2* in 90A cohort.

Figure S6.3. Heatmaps for the common gene pool in both p53 signal transduction pathway and the network of *MYB_ARNT2* in 90A cohort.

Figure S6.4. Heatmaps for the common gene pool in both ribosome signal transduction pathway and the network of *MYB_ARNT2* in 90A cohort.

Figure S6.5. Heatmaps for the common gene pool in both PDGFRB signal transduction pathway and the network of *MYB_ARNT2* in 90A cohort.

Figure S6.6. Heatmaps for the common gene pool in both ERBB2 signal transduction pathway and the network of *MYB_ARNT2* in 90A cohort.

Figure S6.7. Heatmaps for the common gene pool significant for both histological grade (Grade) and the network of *MYB_ARNT2* in 90A cohort.

Figure S6.8. Heatmaps for the common gene pool significant for both lymphovascular invasion (LVI) and the network of *MYB_ARNT2* in 90A cohort.

Figure S6.9. Heatmaps for the common gene pool significant for both tumor size (size) and the network of *MYB_ARNT2* in 90A cohort.

Figure S6.10. The most relevant subnetworks of *MYB*, *ARNT2* and *SALL2* for epithelial-to-mesenchymal transition (EMT) activities in 90A cohort.

Table S6.1. . Partial results of the supervised network analysis in Tables S1.3 and S1.4 show *MYB* and *ARNT2* shared target genes (57 genes) overlapping with the downstream target genes of ARNT2/SIM1.

Suppl. 7. (Pages 202-213)

Figure S7.1. Mean plot analyses of mRNA levels for *POU2F1*(9472) in eight clinical categories and in three cohorts of infiltrating ductal carcinoma (IDCs), respectively.

Figure S7.2. Mean plot analyses of mRNA levels for *POU2F1*(11991) in eight clinical categories and in three cohorts of infiltrating ductal carcinoma (IDCs), respectively.

Figure S7.3. Mean plot analyses of mRNA levels for *SALL2* (3096) in eight clinical categories and in three cohorts of infiltrating ductal carcinoma (IDCs), respectively.

Figure S7.4. Mean plot analyses of mRNA levels for *XBP1* (10024) in eight clinical categories and in three cohorts of infiltrating ductal carcinoma (IDCs), respectively.

Figure S7.5. Mean plot analyses of mRNA levels for *MYBL1*(C11830) in eight clinical categories and in three cohorts of infiltrating ductal carcinoma (IDCs), respectively.

Figure S7.6. Mean plot analyses of mRNA levels for *MYBL2*(10757) in eight clinical categories and in three cohorts of infiltrating ductal carcinoma (IDCs), respectively.

Suppl. 8. (Page 214)

Figure S8.1. The heatmaps for the expression pattern of forty one probes in relation to the gene expression patterns of the four transcription factors (*ARNT2*, *MYB*, *MYBL1* and *MYBL2*) in three cohorts.

Figure S8.2. The heatmaps for the expression pattern of forty one probes in relation to the gene expression patterns of the four transcription factors (*ARNT2*, *MYB*, *MYBL1* and *MYBL2*) in two cohorts.

S1.1

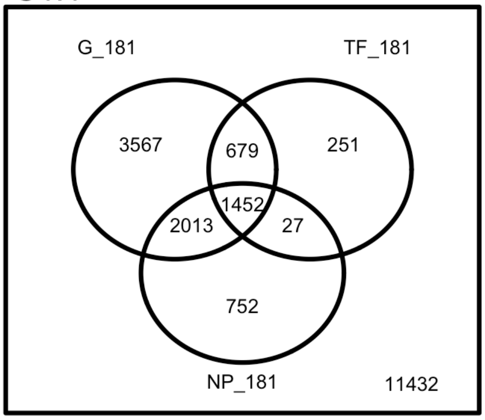

S1.2

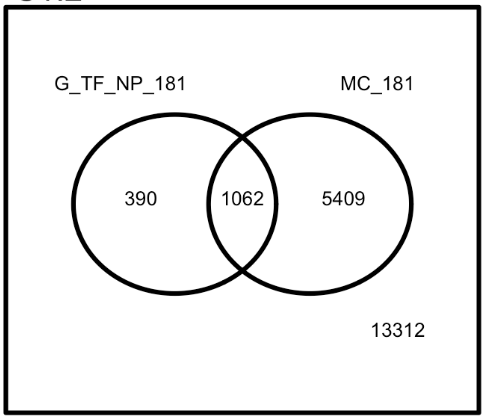

S1.3

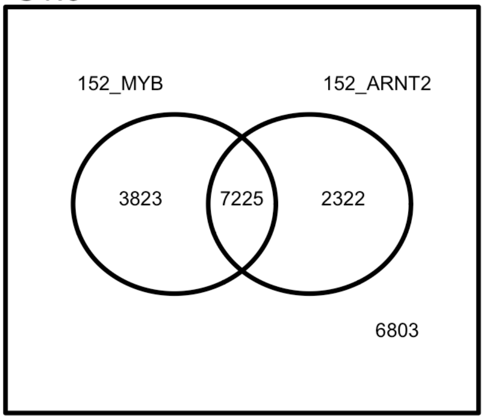

S1.4

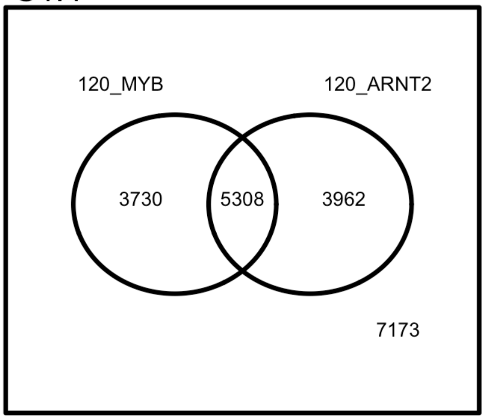

S1.5

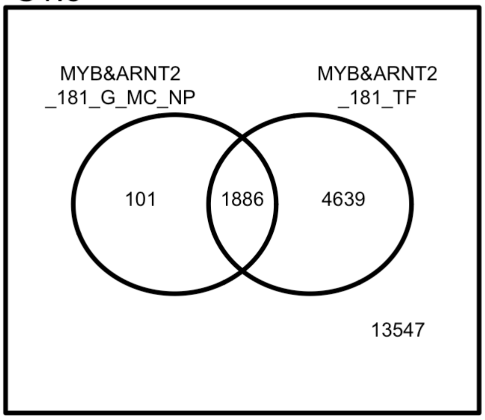

S1.6

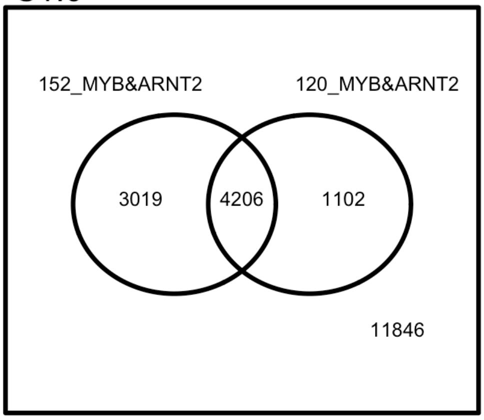

S1.7

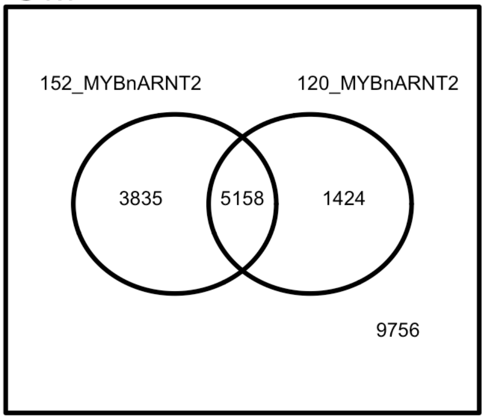

S1.8

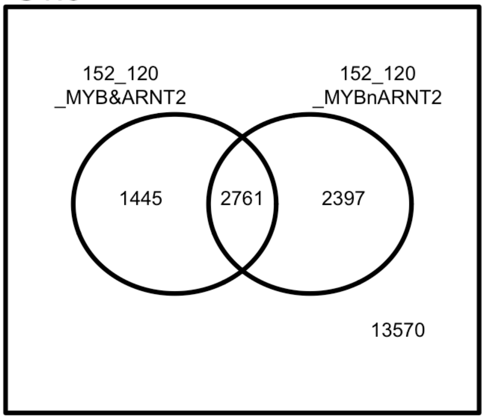

S1.9

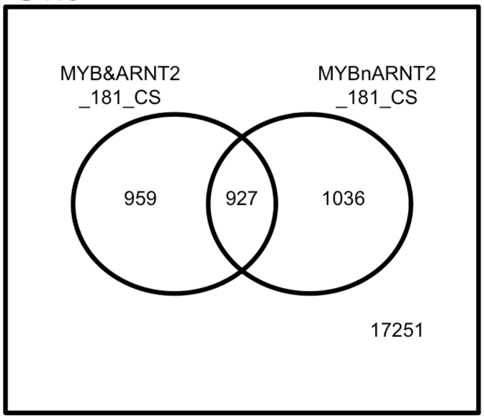

S1.10

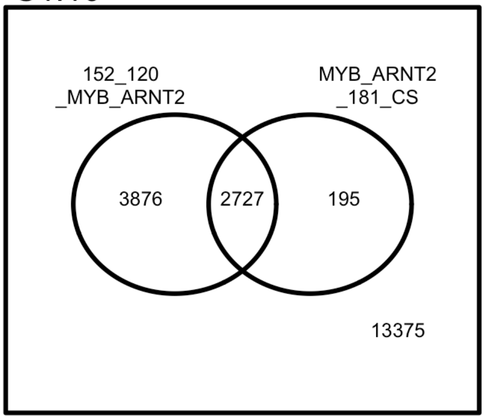

S1.11

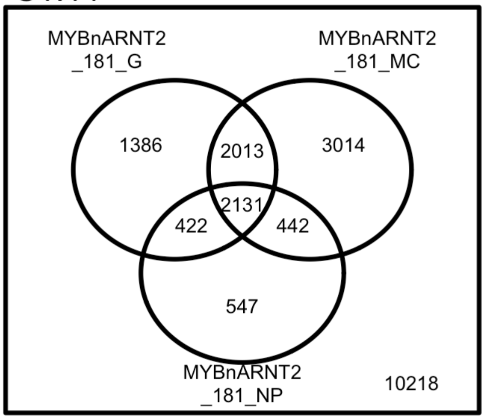

S1.12

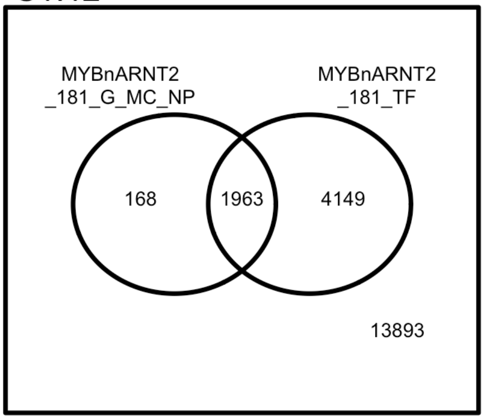

S2.1

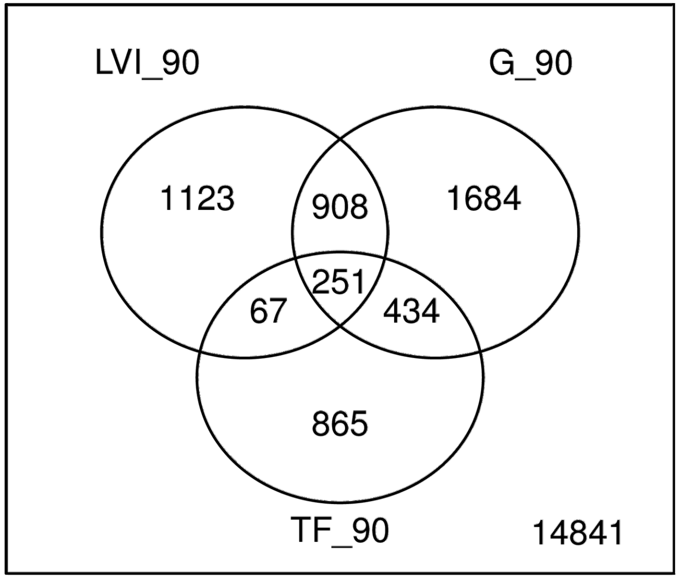

LVI_90
G_90
1123
908
1684
251
67
434
865
TF_90
14841

S2.2

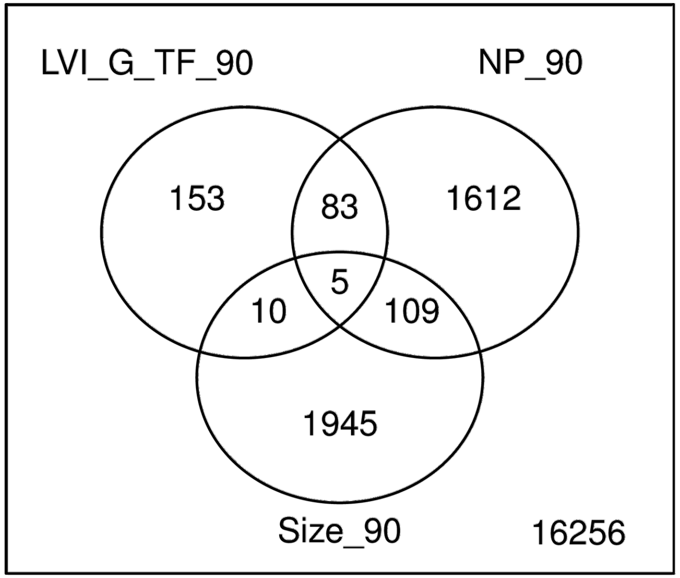

LVI_G_TF_90
NP_90
153
83
1612
5
10
109
1945
Size_90
16256

S2.3

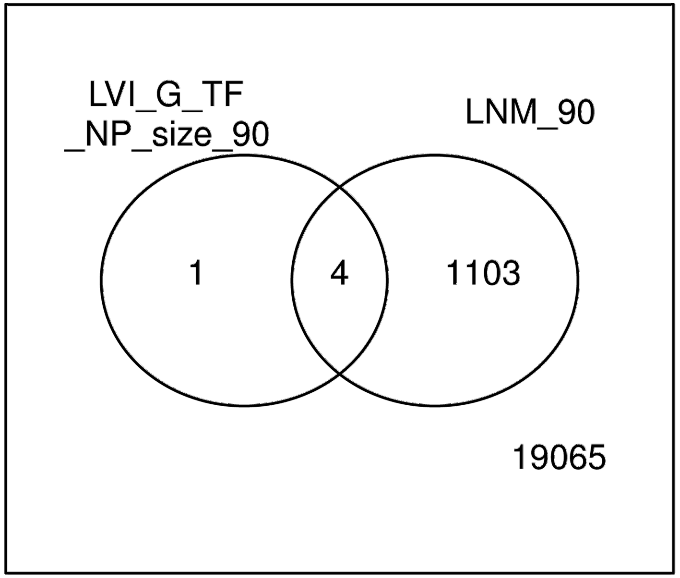

LVI_G_TF
_NP_size_90
LNM_90
1
4
1103
19065

S3.1

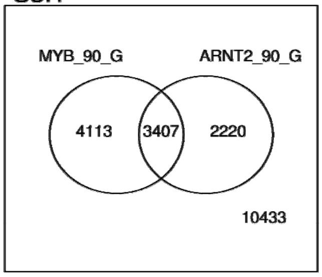

S3.2

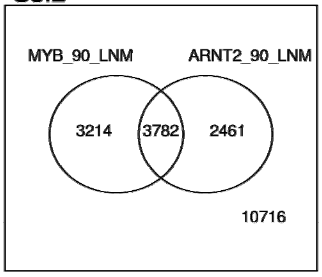

S3.3

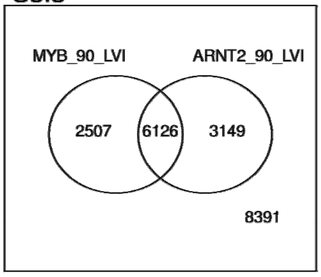

S3.4

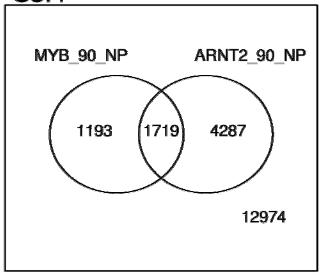

S3.5

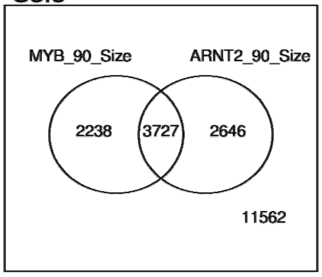

S3.6

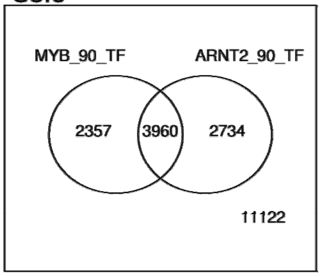

S3.7

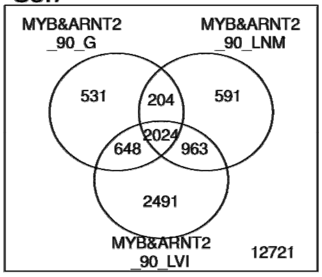

S3.8

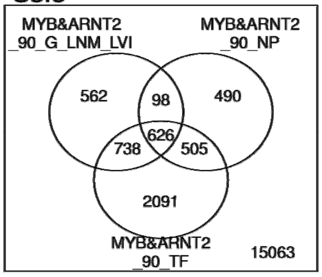

S3.9

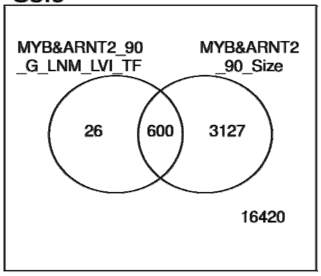

S3.10

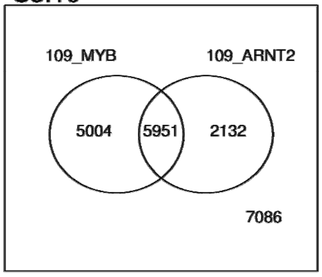

S3.11

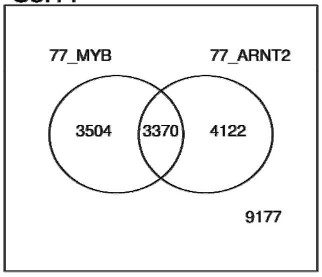

S3.12

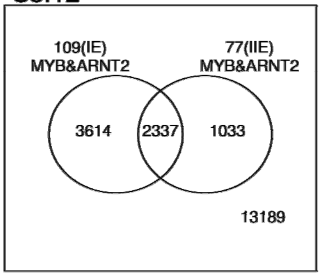

S3.13

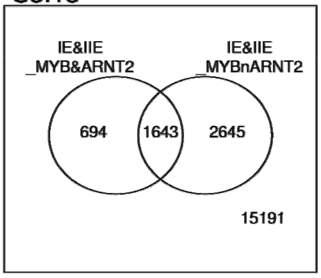

S3.14

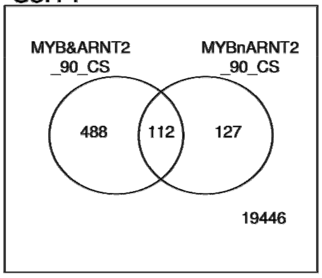

S3.15

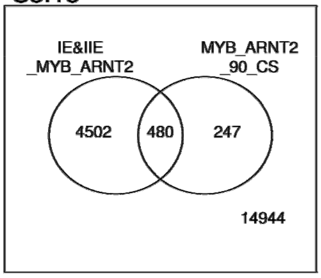

S3.16

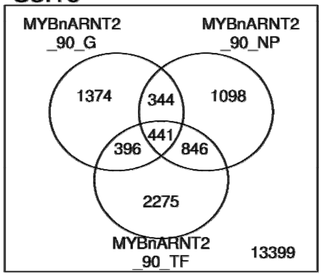

S3.17

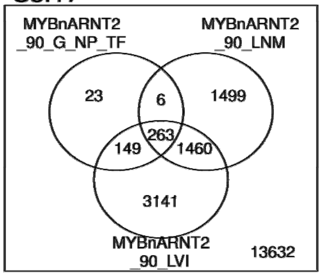

S3.18

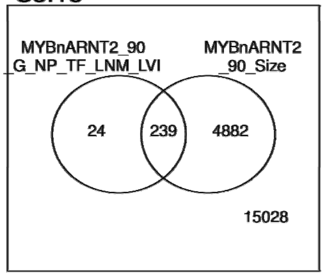

S3.19

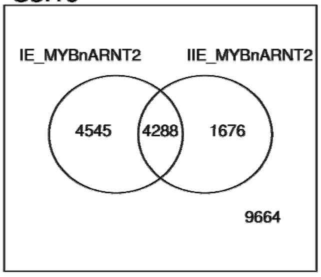

S4.1 90 feature type I

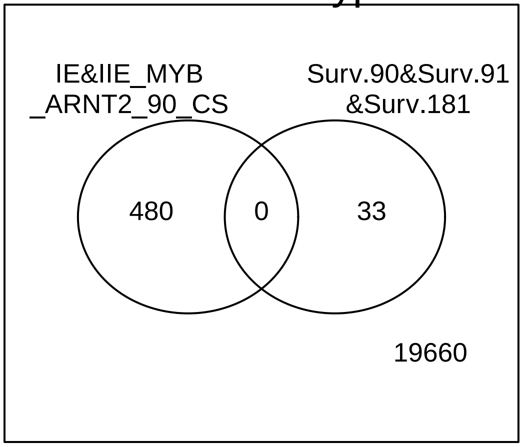

S4.2 90 feature type II

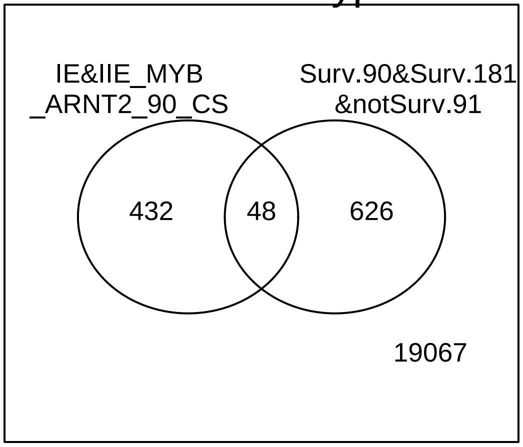

S4.3 90 feature typeIII

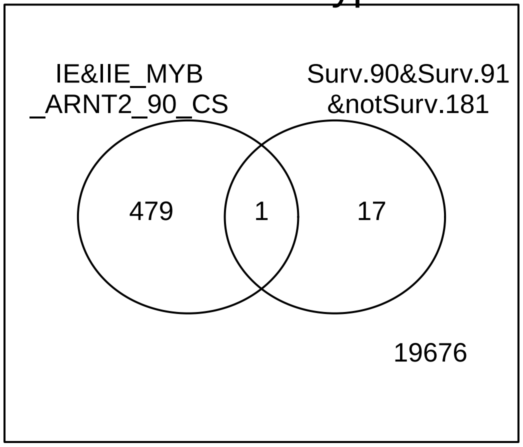

S4.4 90 feature type IV

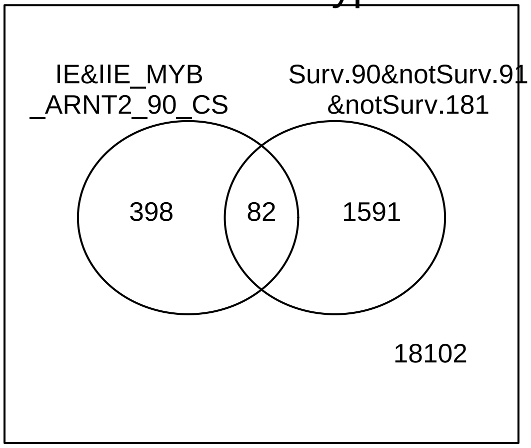

S4.5 181 feature type I

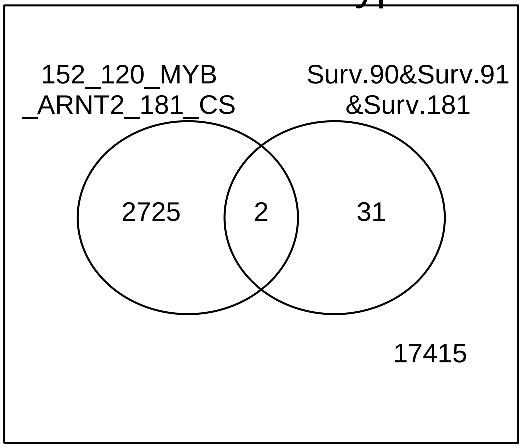

S4.6 181 feature type II

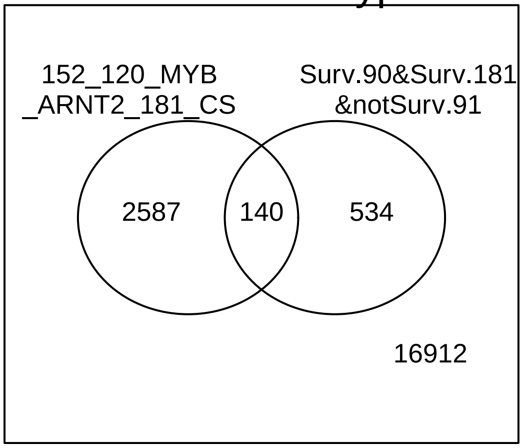

S4.7 181 feature type III

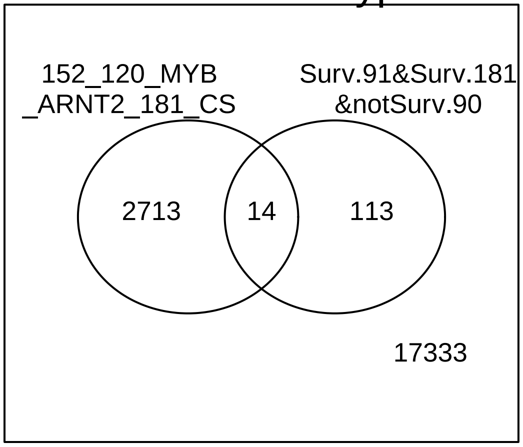

S4.8 181 feature type IV

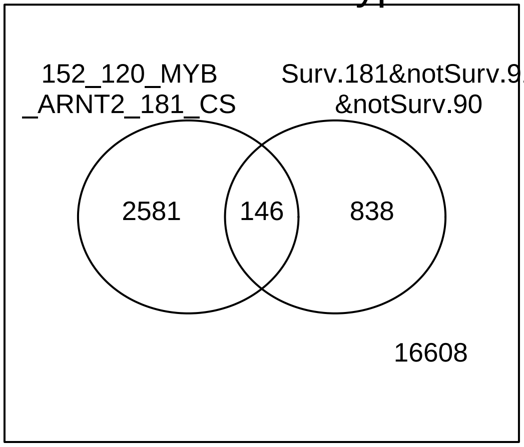

S4.9

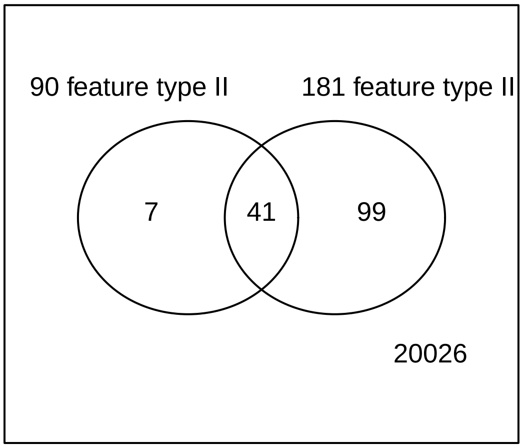

S4.10

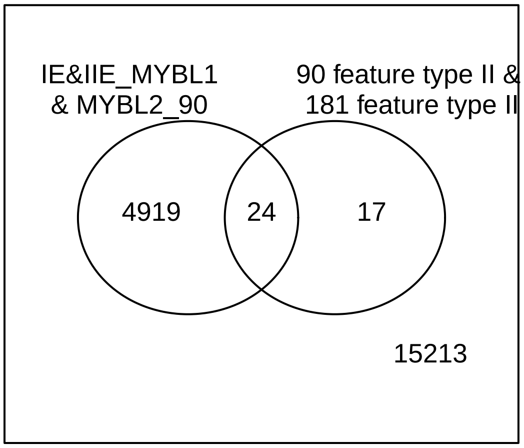

Feature.No.	ProbeName	GeneSymbol	S1.1	S1.2	S1.3	S1.4	S1.5	S1.6	S1.7	S1.8	S1.9	S1.10	S1.11	S1.12	S2.1	S2.2	S2.3	S3.1	S3.2	S3.3	S3.4	S3.5	S3.6	S3.7	S3.8	S3.9	S3.10	S3.11	S3.12	S3.13	S3.14	S3.15	S3.16	S3.17	S3.18	S3.19	S4.1	S4.2	S4.3	S4.4	S4.5	S4.6	S4.7	S4.8	S4.9	S4.10
530	A_23_P89460	AATF																	Y	Y		Y																								
14648	A_23_P63584	AHCTF1				Y																																								
10665	A_23_P71948	AKNA																				Y	Y																							
6780	A_23_P113111	AR			Y				Y	Y	Y	Y	Y	Y				Y	Y	Y		Y	Y	Y						Y						Y										
3486	A_23_P105644	ATF1							Y	Y													Y																							
4500	A_23_P369228	ATF2																					Y																							
19270	A_23_P209879	ATF2																		Y																										
16682	A_23_P120940	ATF4			Y	Y	Y	Y	Y	Y	Y	Y	Y	Y				Y		Y		Y	Y				Y	Y	Y	Y						Y										
19529	A_23_P119338	ATF5				Y			Y	Y																		Y																		
878	A_23_P59163	ATF6B																		Y			Y																							
12951	A_23_P370682	BATF2				Y	Y		Y	Y	Y	Y	Y						Y																											
9403	A_23_P160720	BATF3			Y	Y	Y	Y	Y	Y	Y	Y	Y	Y				Y	Y	Y			Y	Y						Y	Y	Y	Y	Y	Y	Y										
938	A_23_P111343	BCLAF1				Y																																								
19451	A_23_P7489	BDP1				Y																						Y		Y						Y										
619	A_23_P125463	BPTF				Y			Y	Y	Y	Y	Y	Y														Y																		
856	A_23_P114075	BPTF																												Y						Y										
10457	A_23_P213458	BTF3			Y	Y		Y	Y	Y	Y	Y	Y	Y				Y	Y	Y		Y	Y	Y			Y	Y	Y	Y						Y										
9734	A_23_P203649	CREBZF			Y	Y	Y	Y	Y	Y	Y	Y	Y	Y						Y							Y	Y	Y	Y																
14296	A_23_P169293	DMRT1				Y																					Y	Y	Y	Y																
4067	A_23_P365694	DMRT2							Y	Y																				Y						Y										
393	A_23_P325100	DMRT3	Y	Y	Y	Y	Y	Y	Y	Y	Y	Y	Y	Y									Y				Y			Y						Y								Y		
14847	A_23_P8663	DMTF1			Y	Y	Y	Y	Y	Y	Y	Y	Y	Y				Y	Y	Y		Y	Y	Y			Y	Y	Y	Y						Y										
7852	A_23_P80032	E2F1	Y	Y	Y	Y	Y	Y	Y	Y	Y	Y						Y	Y	Y	Y	Y		Y			Y			Y						Y										
101	A_23_P385034	E2F3							Y	Y																																				
16078	A_23_P152218	E2F4			Y													Y	Y	Y	Y	Y		Y																						
14993	A_23_P35871	E2F8	Y	Y																																										
14416	A_23_P15051	E4F1				Y														Y																										
17366	A_23_P2801	ELF1	Y	Y	Y						Y		Y	Y				Y		Y			Y				Y			Y						Y										
3799	A_23_P132948	ELF2	Y		Y	Y	Y	Y	Y	Y	Y	Y	Y	Y					Y	Y		Y					Y	Y	Y	Y						Y										
16930	A_23_P104188	ELF3			Y	Y		Y		Y										Y							Y	Y	Y	Y																
21288	A_23_P9621	ELF4																		Y							Y																			
20154	A_23_P13465	ELF5			Y	Y		Y	Y	Y	Y	Y	Y	Y				Y	Y	Y		Y		Y				Y		Y						Y										
2530	A_23_P204629	ELK3	Y		Y																						Y																			
6343	A_23_P161098	ELK4																Y	Y	Y				Y																						
5561	A_23_P309739	ESR1	Y	Y	Y	Y	Y	Y	Y	Y	Y	Y	Y	Y				Y	Y	Y			Y	Y			Y	Y	Y	Y						Y										
11573	A_23_P54100	ESR2																		Y			Y																							
5480	A_23_P1585	ESRRA			Y		Y				Y																Y																			
8943	A_23_P74901	ESRRG	Y						Y	Y	Y	Y	Y	Y	Y			Y	Y	Y	Y		Y	Y	Y			Y																		
11507	A_23_P127525	ETS1							Y	Y			Y					Y	Y	Y		Y	Y	Y						Y						Y										
10001	A_23_P257924	ETS2			Y																																									
129	A_23_P134366	ETV1																	Y	Y			Y																							
2456	A_23_P101831	ETV2							Y	Y																																				
12480	A_23_P400945	ETV3				Y																																								
9114	A_23_P105264	ETV6							Y	Y	Y	Y	Y	Y						Y							Y			Y						Y										
163	A_23_P42347	ETV7	Y	Y	Y				Y	Y																	Y																			
3805	A_23_P5460	FEV																		Y																										
C10107.1	A_23_P37127	FOXA1	Y	Y	Y	Y		Y	Y	Y	Y	Y	Y	Y				Y	Y	Y	Y	Y	Y	Y	Y	Y	Y	Y	Y	Y	Y	Y				Y										
3048	A_23_P208737	FOXA3																					Y																							
4099	A_23_P140527	FOXB1				Y																																								
12715	A_23_P258456	FOXC1							Y	Y	Y	Y	Y	Y																																
14242	A_23_P390504	FOXC1			Y				Y	Y	Y	Y	Y	Y									Y				Y			Y						Y										
21134	A_23_P387500	FOXC2			Y	Y		Y		Y										Y		Y	Y																							
6862	A_23_P255978	FOXD1			Y	Y		Y	Y	Y																	Y			Y						Y										
13346	A_23_P86171	FOXD2			Y	Y		Y		Y																																				
16479	A_23_P413585	FOXD2			Y	Y		Y		Y																																				
7572	A_23_P46560	FOXD3			Y	Y	Y	Y	Y	Y	Y	Y								Y		Y	Y				Y			Y						Y										
12533	A_23_P131507	FOXD4L1																					Y																							
183	A_23_P112666	FOXE3			Y	Y		Y	Y	Y								Y		Y	Y	Y	Y				Y	Y	Y	Y																
8958	A_23_P118254	FOXF1																		Y								Y																		
11800	A_23_P82959	FOXH1			Y	Y		Y	Y	Y										Y		Y	Y				Y			Y						Y										
21605	A_23_P255358	FOXI1			Y	Y		Y	Y	Y																	Y	Y	Y	Y																
5983	A_23_P348636	FOXJ1							Y	Y																																				
17375	A_23_P129752	FOXJ1			Y	Y		Y		Y										Y																										
22277	A_23_P204238	FOXJ2				Y			Y	Y																																				
17950	A_23_P566	FOXJ3			Y	Y		Y	Y	Y										Y							Y	Y	Y	Y						Y										
19833	A_23_P397969	FOXK2			Y																		Y				Y																			
8136	A_23_P365081	FOXL1			Y	Y		Y	Y	Y	Y	Y	Y	Y													Y	Y	Y	Y						Y										
9719	A_23_P110052	FOXL2			Y																						Y																			
6260	A_23_P151150	FOXM1							Y	Y								Y	Y	Y		Y		Y						Y						Y										
21183	A_23_P118693	FOXN1			Y	Y		Y		Y																																				
3603	A_23_P259192	FOXN2			Y														Y	Y		Y	Y																							
19105	A_23_P61463	FOXN2			Y															Y		Y	Y							Y						Y										
9611	A_23_P140405	FOXN3				Y														Y								Y																		
11979	A_23_P88434	FOXN3													Y					Y																										
8281	A_23_P151426	FOXO1																					Y																							
2539	A_23_P345575	FOXO3																Y										Y																		

3787	A_23_P70757	FOXO3			Y	Y		Y	Y	Y	Y	Y	Y	Y														Y																		
11951	A_23_P217487	FOXO4			Y	Y		Y		Y																	Y																			
14511	A_23_P155257	FOXP1			Y	Y		Y	Y	Y	Y	Y	Y	Y													Y	Y	Y	Y						Y										Y		
10520	A_23_P134542	FOXP2																		Y																										
8745	A_23_P122350	FOXQ1																	Y	Y		Y																								
16424	A_23_P143385	FOXS1			Y	Y		Y		Y								Y	Y	Y		Y	Y	Y			Y																			
14853	A_23_P73208	GABPB2			Y	Y	Y	Y		Y	Y	Y	Y	Y						Y	Y	Y																								
7325	A_23_P304464	GATA1	Y			Y																					Y	Y	Y	Y																
8502	A_23_P65609	GTF2A1																												Y						Y										
2682	A_23_P34628	GTF2B																Y		Y																										
13968	A_23_P211748	GTF2E1													Y																															
9784	A_23_P16144	GTF2F1			Y	Y		Y		Y										Y	Y	Y	Y				Y																			
12556	A_23_P36183	GTF2H1				Y												Y	Y	Y		Y	Y	Y			Y																			
8871	A_23_P252845	GTF2H2D																									Y	Y	Y	Y																
12501	A_23_P168567	GTF2I	Y	Y	Y	Y	Y	Y	Y	Y	Y	Y	Y	Y				Y	Y	Y	Y	Y	Y	Y	Y	Y	Y	Y	Y	Y	Y	Y	Y	Y	Y	Y				Y						
17400	A_23_P152335	GTF3C1																	Y	Y																										
7169	A_23_P169887	GTF3C2																			Y																									
4583	A_23_P429535	GTF3C3																		Y																										
18858	A_23_P112406	GTF3C5			Y	Y		Y		Y								Y	Y	Y	Y	Y	Y	Y	Y	Y	Y			Y	Y	Y				Y										
20433	A_23_P215787	HBP1																		Y			Y																							
20504	A_23_P57676	HLTF			Y																						Y	Y	Y	Y																
3506	A_23_P145752	HOXA1			Y													Y									Y			Y						Y										
8970	A_23_P253368	HOXA10																					Y							Y						Y										
14401	A_23_P42706	HOXA11			Y				Y	Y									Y								Y																			
16843	A_23_P111571	HOXA3																		Y	Y	Y																								
8514	A_23_P253982	HOXA4							Y	Y																																				
20448	A_23_P93773	HOXA5																					Y																							
8098	A_23_P82370	HOXA6																										Y																		
20730	A_23_P107283	HOXB2			Y				Y	Y	Y	Y	Y	Y													Y																			
19475	A_23_P100872	HOXB3							Y	Y																																				
18129	A_23_P89480	HOXB4																		Y			Y																							
4281	A_23_P78084	HOXB5																												Y						Y										
13428	A_23_P363316	HOXB5	Y	Y	Y		Y				Y		Y														Y																			
6273	A_23_P66682	HOXB6			Y																						Y																			
21229	A_23_P55276	HOXB7																Y					Y																							
11673	A_23_P38417	HOXB8			Y	Y		Y		Y									Y	Y		Y	Y																							
21299	A_23_P27013	HOXB9																									Y																			
6562	A_23_P36544	HOXC10			Y																									Y						Y										
14499	A_23_P47938	HOXC11																					Y																							
4505	A_23_P335981	HOXC12			Y	Y		Y		Y								Y	Y	Y		Y		Y			Y																			
9609	A_23_P2344	HOXC6			Y																						Y			Y						Y										
2839	A_23_P398476	HOXC8																												Y						Y										
6910	A_23_P25150	HOXC9							Y	Y	Y	Y	Y	Y							Y																									
6991	A_23_P120248	HOXD1																									Y																			
17831	A_23_P381368	HOXD10																		Y			Y							Y						Y										
21446	A_23_P131640	HOXD10							Y	Y										Y		Y																								
12702	A_23_P154420	HOXD12																Y	Y			Y																								
12655	A_23_P165796	HOXD13													Y																															
12474	A_23_P323180	HOXD3			Y													Y	Y			Y					Y																			
9247	A_23_P210164	HOXD8																									Y																			
17550	A_23_P253841	HSF1			Y	Y	Y	Y	Y	Y	Y	Y	Y	Y				Y	Y	Y	Y	Y	Y	Y	Y	Y	Y	Y	Y	Y	Y	Y				Y										
18017	A_23_P122746	HSF2				Y														Y								Y																		
15454	A_23_P96497	HSFX1			Y																																									
5788	A_23_P45579	HSFY2			Y																						Y																			
22061	A_23_P107875	KLF1	Y	Y	Y															Y							Y																			
3875	A_23_P44505	KLF11																												Y						Y										
7776	A_23_P319512	KLF11			Y													Y	Y	Y		Y	Y	Y			Y	Y	Y	Y						Y										
4409	A_23_P354805	KLF12			Y	Y		Y	Y	Y										Y		Y																								
874	A_23_P413150	KLF13			Y													Y	Y	Y		Y	Y	Y			Y																			
6422	A_23_P393627	KLF14							Y	Y																																				
11025	A_23_P40809	KLF15																		Y		Y					Y																			
3230	A_23_P391185	KLF16			Y				Y	Y								Y	Y	Y	Y	Y	Y	Y	Y	Y	Y			Y	Y	Y	Y			Y										
20852	A_23_P16476	KLF16			Y																						Y																			
11325	A_23_P434972	KLF17			Y																						Y																			
12602	A_23_P213306	KLF3																		Y		Y					Y																			
21670	A_23_P32233	KLF4																												Y						Y										
15152	A_23_P53891	KLF5	Y	Y					Y	Y																		Y		Y						Y										
21313	A_23_P67977	KLF7			Y	Y		Y		Y								Y		Y			Y				Y			Y						Y										
7411	A_23_P415401	KLF9				Y																																								
16710	A_23_P135139	KLF9			Y				Y	Y																	Y	Y	Y	Y						Y										
15056	A_23_P340271	LMX1A							Y	Y																	Y			Y						Y										
15150	A_23_P34690	LMX1A				Y														Y			Y																							
22083	A_23_P146708	LMX1B																		Y			Y																							
11878	A_23_P22033	MAZ																Y	Y	Y	Y	Y	Y	Y	Y	Y					Y															
12619	A_23_P33433	MAZ			Y													Y	Y	Y		Y		Y																						
8288	A_23_P46333	MTF2				Y																						Y																		
5586	A_23_P31073	MYB	Y	Y	Y	Y	Y	Y	Y	Y	Y	Y	Y	Y	Y	Y	Y	Y	Y	Y	Y	Y	Y	Y	Y	Y	Y	Y	Y	Y	Y	Y	Y	Y	Y	Y								Y		

C11830	A_23_P43157	MYBL1							Y	Y								Y	Y	Y				Y																					
10757	A_23_P143184	MYBL2	Y	Y	Y	Y	Y	Y	Y	Y	Y	Y						Y	Y	Y		Y		Y			Y	Y	Y	Y						Y									
9452	A_23_P215956	MYC							Y	Y																																			
137	A_23_P209583	MYCN																		Y			Y																						
2713	A_23_P210835	MYT1			Y																																								
C12928.2	A_23_P204641	NANOG							Y	Y								Y		Y		Y	Y																						
2987	A_23_P131115	NFIC			Y													Y	Y	Y		Y	Y	Y			Y																		
425	A_23_P165295	NFIX																		Y		Y								Y						Y									
2050	A_23_P94683	NFX1							Y	Y								Y		Y		Y	Y				Y																		
19441	A_23_P501583	NFYA				Y																																							
18845	A_23_P53668	NFYB			Y	Y		Y		Y										Y																									
18452	A_23_P22994	NFYC																	Y	Y							Y	Y	Y	Y															
17807	A_23_P396135	NKX2-5				Y																																							
11752	A_23_P160800	NR0B2	Y	Y														Y		Y							Y																		
22375	A_23_P204828	NR2C1			Y				Y	Y																	Y			Y						Y									
13146	A_23_P40936	NR2C2																					Y																						
16845	A_23_P338372	NR2E1																Y																											
3372	A_23_P205867	NR2E3			Y	Y		Y	Y	Y	Y	Y	Y	Y													Y	Y	Y	Y						Y									
22436	A_23_P348737	NR2F1																		Y			Y																						
2697	A_23_P422703	NR2F2			Y																						Y																		
18993	A_23_P88589	NR2F2			Y																						Y																		
2372	A_23_P108326	NR2F6			Y	Y	Y	Y	Y	Y	Y	Y						Y	Y	Y	Y	Y	Y	Y	Y	Y	Y				Y														
11190	A_23_P214059	NR3C1			Y	Y		Y		Y								Y	Y	Y		Y	Y	Y			Y	Y	Y	Y															
12491	A_23_P131207	NR4A2			Y						Y		Y	Y				Y									Y				Y		Y	Y	Y										
6953	A_23_P398566	NR4A3																									Y																		
652	A_23_P216913	NR5A1			Y																		Y																						
16670	A_23_P160996	NR5A2			Y	Y		Y		Y									Y	Y		Y	Y																						
4809	A_23_P216900	NR6A1			Y				Y	Y																				Y						Y									
18341	A_23_P59418	NRF1			Y	Y		Y		Y								Y	Y	Y		Y		Y																					
13835	A_23_P154849	OLIG1			Y	Y		Y		Y																		Y																	
16478	A_23_P211079	OLIG2																Y	Y	Y		Y		Y																					
20411	A_23_P382218	OLIG3																										Y																	
11252	A_23_P59691	PAX4			Y	Y		Y	Y	Y	Y	Y	Y	Y						Y		Y					Y	Y	Y	Y						Y									
11809	A_23_P138938	PGR	Y	Y	Y	Y	Y	Y		Y	Y	Y	Y	Y							Y		Y				Y																		
9472	A_23_P424002	POU2F1			Y	Y	Y	Y	Y	Y	Y	Y	Y	Y				Y	Y	Y	Y	Y	Y	Y	Y	Y	Y	Y	Y	Y	Y	Y				Y		Y				Y			Y	Y
11991	A_23_P97130	POU2F1			Y	Y	Y	Y	Y	Y	Y	Y	Y	Y				Y	Y	Y	Y	Y	Y	Y	Y	Y	Y	Y	Y	Y	Y	Y				Y		Y				Y			Y	Y
6868	A_23_P78532	POU2F2			Y	Y	Y	Y	Y	Y	Y	Y							Y	Y							Y																		
7119	A_23_P97038	POU3F1																												Y						Y									
560	A_23_P82027	POU3F2			Y																																								
5888	A_23_P114210	POU3F4							Y	Y																																			
18314	A_23_P213073	POU4F2			Y	Y		Y	Y	Y											Y									Y						Y									
15613	A_23_P121976	POU4F3																Y	Y																										
7193	A_23_P358564	POU5F2																				Y																							
8004	A_23_P147900	POU6F2																									Y																		
19371	A_23_P428329	PTF1A																	Y																										
20979	A_23_P207842	RARA			Y																						Y																		
16764	A_23_P211888	RARB			Y				Y	Y																		Y		Y						Y									
12114	A_23_P387552	RARG			Y													Y	Y	Y		Y	Y	Y			Y			Y						Y									
13459	A_23_P167081	REST				Y													Y	Y	Y							Y																	
22283	A_23_P71698	RORB			Y														Y	Y							Y																		
18	A_23_P372910	RORC			Y																																								
20491	A_23_P6245	RUNX1				Y																																							
9049	A_23_P418986	RUNX2			Y	Y	Y	Y	Y	Y	Y	Y															Y	Y	Y	Y						Y									
17558	A_23_P58921	RUNX2																		Y																									
8044	A_23_P51231	RUNX3	Y		Y																						Y																		
1276	A_23_P423197	RXRA																									Y																		
3935	A_23_P59179	RXRB																Y	Y	Y		Y	Y	Y			Y																		
4324	A_23_P54669	SALL1	Y	Y	Y																																								
4423	A_23_P328074	SALL1			Y														Y								Y																		
3096	A_23_P48585	SALL2	Y	Y	Y	Y	Y	Y	Y	Y	Y	Y	Y	Y	Y			Y	Y	Y	Y	Y	Y	Y	Y	Y	Y	Y	Y	Y	Y	Y	Y	Y	Y	Y		Y				Y			Y	
11429	A_23_P217698	SHOX			Y	Y		Y		Y									Y								Y																		
2009	A_23_P212870	SMAD1			Y				Y	Y											Y																								
11216	A_23_P15937	SMAD2			Y															Y																									
6878	A_23_P48936	SMAD3			Y	Y		Y	Y	Y										Y		Y					Y																		
11741	A_23_P359091	SMAD3			Y		Y				Y																Y																		
1114	A_23_P27346	SMAD4							Y	Y										Y										Y						Y									
18125	A_23_P144944	SMAD5																										Y		Y						Y									
8739	A_23_P65812	SMAD6																Y	Y	Y	Y	Y	Y	Y	Y	Y	Y				Y														
3259	A_23_P2814	SMAD9																			Y		Y																						
5055	A_23_P131846	SNAI1				Y			Y	Y	Y	Y	Y	Y									Y							Y						Y									
15437	A_23_P169039	SNAI2																	Y																										
10628	A_23_P53897	SOX1			Y																						Y																		
1640	A_23_P143694	SOX10			Y		Y		Y	Y	Y	Y	Y	Y				Y	Y	Y		Y	Y	Y			Y			Y						Y									
16191	A_23_P22378	SOX11																		Y																									
6456	A_23_P131887	SOX12																Y	Y			Y					Y			Y						Y									
6917	A_23_P255418	SOX18							Y	Y										Y		Y	Y																						
12467	A_23_P379089	SOX21			Y				Y	Y										Y							Y																		

17470	A_23_P82176	SOX4																		Y			Y																							
13277	A_23_P87952	SOX5	Y	Y	Y															Y		Y	Y				Y	Y	Y	Y																
10130	A_23_P343799	SOX6																Y												Y						Y										
19864	A_23_P306988	SOX7			Y																																									
12855	A_23_P26843	SOX9							Y	Y										Y			Y																							
7934	A_23_P366312	SP1							Y	Y	Y	Y	Y	Y													Y			Y						Y										
20927	A_23_P53397	SP1																				Y																								
7228	A_23_P381451	SP2			Y						Y		Y	Y				Y	Y	Y		Y		Y			Y																			
2113	A_23_P71064	SP4			Y																																									
6912	A_23_P135381	SP5			Y								Y					Y																												
20204	A_23_P111194	SPDEF				Y			Y	Y	Y	Y	Y	Y					Y	Y		Y	Y					Y		Y						Y										
21821	A_23_P27664	SPIB				Y																					Y																			
6730	A_23_P129786	SREBF1	Y	Y					Y	Y	Y	Y	Y	Y				Y												Y						Y										
2128	A_23_P419602	SREBF2			Y	Y	Y	Y	Y	Y	Y	Y	Y	Y				Y	Y	Y	Y	Y	Y	Y	Y	Y	Y	Y	Y	Y	Y	Y				Y				Y						
14413	A_23_P111020	SRF			Y	Y		Y		Y										Y							Y	Y	Y	Y																
2002	A_23_P76090	STAT2			Y													Y	Y	Y		Y	Y	Y			Y			Y						Y										
19536	A_23_P68031	STAT4	Y	Y																																										
2703	A_23_P100788	STAT5B					Y				Y							Y	Y	Y	Y	Y	Y	Y	Y	Y	Y				Y															
11867	A_23_P151930	TCF12			Y	Y		Y		Y																		Y		Y						Y										
4845	A_23_P93321	TCF19			Y													Y	Y	Y		Y	Y	Y			Y																			
15743	A_23_P49219	TCF25			Y	Y	Y	Y	Y	Y	Y	Y	Y	Y				Y	Y	Y	Y	Y	Y	Y	Y	Y	Y	Y	Y	Y	Y	Y				Y										
17729	A_23_P67708	TCF3	Y	Y	Y	Y		Y		Y																	Y	Y	Y	Y																
18437	A_23_P27332	TCF4																										Y																		
18795	A_23_P142872	TCF7L1							Y	Y	Y	Y	Y	Y													Y			Y						Y										
19623	A_23_P328501	TCF7L2							Y	Y										Y							Y																			
16948	A_23_P143147	TCFL5			Y																		Y																							
6013	A_23_P36234	TEAD1			Y	Y		Y	Y	Y												Y					Y	Y	Y	Y																
15404	A_23_P429340	TEAD1																									Y	Y	Y	Y																
16148	A_23_P145104	TFAP2B							Y	Y	Y	Y	Y	Y																Y						Y										
2606	A_23_P120472	TFAP2C																	Y																											
6686	A_23_P141092	TFAP4			Y				Y	Y									Y								Y																			
8490	A_23_P93499	TFB1M			Y													Y		Y																										
22124	A_23_P45940	TFB2M			Y																Y																									
19486	A_23_P347528	TFCP2			Y	Y		Y	Y	Y																																				
1496	A_23_P5301	TFCP2L1							Y	Y																																				
7375	A_23_P14243	TFDP1																Y																												
9366	A_23_P166964	TFDP2																	Y	Y			Y																							
14564	A_23_P10513	TFDP3			Y						Y		Y	Y				Y	Y								Y																			
13544	A_23_P84952	TFE3			Y	Y		Y	Y	Y	Y	Y	Y	Y				Y		Y		Y	Y				Y	Y	Y	Y						Y										
14822	A_23_P368724	TFEB			Y	Y	Y	Y	Y	Y	Y	Y											Y				Y																			
1981	A_23_P254296	TFEC	Y	Y	Y				Y	Y					Y			Y									Y																			
15965	A_23_P100772	UBTF			Y	Y		Y	Y	Y								Y	Y	Y	Y	Y	Y	Y	Y	Y					Y		Y													
18369	A_23_P153776	USF2			Y	Y		Y	Y	Y										Y		Y	Y				Y																			
6317	A_23_P162589	VDR																				Y																								
10024	A_23_P120845	XBP1	Y	Y	Y	Y		Y	Y	Y	Y	Y	Y	Y				Y		Y	Y	Y	Y				Y	Y	Y	Y	Y	Y	Y	Y	Y	Y		Y				Y			Y	Y
17461	A_23_P104224	A1CF	Y	Y	Y	Y		Y		Y								Y	Y	Y				Y			Y	Y	Y	Y						Y										
1583	A_23_P140858	A2BP1																									Y			Y						Y										
11948	A_23_P57568	A4GALT			Y	Y	Y	Y	Y	Y	Y	Y	Y	Y				Y	Y	Y		Y	Y	Y			Y	Y	Y	Y						Y										
15859	A_23_P64799	AAAS																Y	Y	Y	Y	Y	Y	Y	Y	Y					Y															
16457	A_23_P204395	AACS			Y																																									
15053	A_23_P80570	AADAC			Y	Y		Y	Y	Y	Y	Y	Y	Y									Y				Y			Y						Y										
19153	A_23_P132644	AADACL1																			Y																									
3375	A_23_P91970	AADACL2			Y																						Y			Y						Y										
6759	A_23_P51874	AADACL4			Y				Y	Y								Y	Y	Y				Y			Y			Y						Y										
10667	A_23_P253484	AADAT			Y																						Y			Y						Y										
8731	A_23_P56529	AAMP			Y	Y		Y		Y									Y	Y		Y																								
14239	A_23_P152527	AANAT							Y	Y										Y			Y				Y			Y						Y										
6929	A_23_P89020	AARS			Y	Y		Y	Y	Y								Y	Y	Y	Y		Y	Y	Y		Y																			
9384	A_23_P202988	AASDHPPT																		Y			Y																							
9432	A_23_P8754	AASS	Y	Y	Y	Y	Y	Y	Y	Y	Y	Y	Y	Y				Y	Y	Y		Y		Y			Y	Y	Y	Y						Y										
3267	A_23_P159166	AATK			Y													Y		Y		Y					Y			Y						Y										
9326	A_23_P152505	ABAT	Y	Y	Y	Y	Y	Y	Y	Y	Y	Y	Y	Y				Y	Y	Y		Y	Y	Y			Y	Y	Y	Y	Y	Y	Y	Y	Y	Y		Y				Y			Y	Y
21074	A_23_P141114	ABAT	Y	Y	Y	Y	Y	Y	Y	Y	Y	Y	Y	Y				Y	Y	Y		Y	Y	Y			Y			Y						Y										
14390	A_23_P216649	ABCA1	Y																																											
7810	A_23_P92607	ABCA10			Y	Y	Y	Y		Y	Y	Y	Y	Y				Y	Y	Y		Y	Y	Y			Y	Y	Y	Y						Y										
9681	A_23_P89422	ABCA10	Y	Y	Y	Y	Y	Y		Y	Y	Y						Y	Y	Y			Y	Y			Y			Y						Y										
12853	A_23_P56369	ABCA12				Y			Y	Y	Y	Y	Y	Y													Y	Y	Y	Y						Y										
3295	A_23_P140876	ABCA3			Y				Y	Y																				Y						Y										
18827	A_23_P160940	ABCA4			Y																																									
7394	A_23_P78018	ABCA5																		Y		Y																								
2279	A_23_P500400	ABCA6																					Y																							
19667	A_23_P39481	ABCA7			Y				Y	Y										Y	Y	Y	Y																							
6147	A_23_P118615	ABCA8			Y				Y	Y									Y				Y																							
6383	A_23_P107225	ABCA9																	Y																											
14212	A_23_P5442	ABCB6																		Y								Y		Y						Y										
21038	A_23_P171258	ABCB7				Y			Y	Y									Y	Y	Y	Y	Y																							
12869	A_23_P82254	ABCB8			Y	Y	Y	Y	Y	Y	Y	Y						Y	Y	Y		Y	Y	Y			Y	Y	Y	Y						Y										

14661	A_23_P128094	ABCB9																			Y			Y					Y																			
12661	A_23_P219013	ABCC10			Y	Y		Y		Y							Y	Y	Y	Y	Y	Y	Y	Y	Y	Y				Y															
1677	A_23_P141074	ABCC11							Y	Y	Y	Y	Y	Y																	Y						Y										
19709	A_23_P258221	ABCC5			Y	Y		Y	Y	Y																		Y	Y	Y	Y															
2205	A_23_P366825	ABCC6			Y														Y		Y	Y							Y						Y										
2692	A_23_P24774	ABCC8	Y	Y	Y	Y		Y	Y	Y	Y	Y	Y	Y									Y					Y			Y						Y										
19980	A_23_P156036	ABCC9				Y			Y	Y																				Y		Y						Y										
7924	A_23_P148556	ABCD1			Y	Y	Y	Y	Y	Y	Y	Y	Y	Y				Y	Y	Y	Y	Y	Y	Y	Y	Y	Y	Y	Y	Y	Y	Y				Y				Y						
12140	A_23_P10847	ABCD2			Y																						Y																			
16159	A_23_P74790	ABCD3			Y	Y	Y	Y	Y	Y	Y	Y	Y	Y						Y			Y				Y	Y	Y	Y						Y										
16552	A_23_P37333	ABCD4																										Y																			
21086	A_23_P251654	ABCF1			Y	Y		Y	Y	Y							Y	Y	Y				Y			Y	Y	Y	Y	Y															
16390	A_23_P215175	ABCF2			Y	Y		Y	Y	Y							Y	Y	Y		Y	Y	Y																							
22510	A_23_P167007	ABCF3			Y	Y	Y	Y		Y	Y	Y									Y		Y					Y	Y	Y	Y						Y										
10685	A_23_P18713	ABCG2			Y													Y		Y							Y			Y						Y										
19889	A_23_P203231	ABCG4			Y				Y	Y																																						
1352	A_23_P17242	ABHD1			Y	Y	Y	Y	Y	Y	Y	Y	Y	Y				Y		Y	Y	Y	Y				Y																		Y		
16793	A_23_P92213	ABHD10			Y	Y		Y		Y							Y		Y			Y																						
15578	A_23_P8558	ABHD11			Y	Y		Y	Y	Y							Y	Y	Y				Y			Y																			
19292	A_23_P149488	ABHD12			Y	Y	Y	Y	Y	Y	Y	Y	Y	Y				Y	Y	Y	Y	Y	Y	Y	Y	Y	Y			Y	Y	Y				Y										
15597	A_23_P205027	ABHD13																												Y						Y										
8235	A_23_P69267	ABHD14A							Y	Y																																						
12761	A_23_P140591	ABHD2																			Y																								
14351	A_23_P305759	ABHD3																					Y																						
17191	A_23_P205519	ABHD4			Y	Y	Y	Y	Y	Y	Y	Y							Y	Y		Y				Y																			
17669	A_23_P43898	ABHD7			Y				Y	Y			Y																																
10397	A_23_P50857	ABHD8				Y												Y	Y	Y	Y	Y	Y	Y	Y	Y						Y															
12036	A_23_P119593	ABHD9																Y		Y			Y				Y																			
5875	A_23_P79692	ABI2			Y				Y	Y														Y																						
16040	A_23_P141429	ABI3	Y	Y	Y	Y	Y	Y		Y	Y	Y	Y	Y					Y								Y			Y						Y										
10478	A_23_P354308	ABI3BP			Y	Y		Y		Y							Y	Y	Y		Y	Y	Y							Y						Y										
8019	A_23_P60180	ABL1																Y		Y	Y	Y	Y																							
3371	A_23_P138099	ABL2							Y	Y	Y	Y	Y	Y																															
16883	A_23_P202520	ABLIM1																Y		Y	Y	Y																								
1701	A_23_P256204	ABLIM3	Y	Y																										Y						Y										
11303	A_23_P59447	ABP1			Y																																										
11104	A_23_P109643	ABTB1																		Y																										
17865	A_23_P356616	ABTB2			Y	Y		Y	Y	Y	Y	Y	Y	Y				Y	Y	Y			Y	Y			Y	Y	Y	Y						Y										
13175	A_23_P69342	ACAA1							Y	Y																																						
6365	A_23_P87791	ACACB																Y	Y				Y						Y																	
21965	A_23_P25351	ACAD10																		Y			Y																						
17043	A_23_P212499	ACAD11			Y	Y	Y	Y	Y	Y	Y	Y							Y	Y	Y		Y	Y	Y			Y	Y	Y	Y						Y										
8029	A_23_P47426	ACAD8							Y	Y	Y	Y	Y	Y																															
3530	A_23_P96764	ACADM				Y														Y																										
9441	A_23_P158570	ACADSB	Y	Y	Y	Y		Y	Y	Y	Y	Y	Y	Y													Y	Y	Y	Y						Y										
11964	A_23_P206032	ACAN			Y				Y	Y																																						
10272	A_23_P107336	ACAP1	Y		Y	Y	Y	Y	Y	Y	Y	Y	Y	Y					Y	Y							Y																			
21348	A_23_P211773	ACAP2																					Y																						
14528	A_23_P23625	ACAP3			Y													Y	Y	Y		Y	Y	Y																							
9045	A_23_P200477	ACBD3																										Y																			
10330	A_23_P97795	ACBD5			Y																						Y																			
8272	A_23_P52127	ACBD6			Y		Y		Y	Y	Y	Y	Y	Y				Y	Y	Y	Y			Y			Y																			
22495	A_23_P55300	ACCN1				Y													Y	Y								Y		Y						Y										
4733	A_23_P204751	ACCN2			Y	Y	Y	Y		Y	Y	Y															Y	Y	Y	Y															
13	A_23_P258433	ACCN5			Y																						Y	Y	Y	Y						Y										
7771	A_23_P26375	ACD																		Y																										
7707	A_23_P38235	ACE																	Y	Y																										
7704	A_23_P252981	ACE2			Y				Y	Y															Y			Y						Y										
4432	A_23_P215245	ACHE			Y	Y	Y	Y	Y	Y	Y	Y	Y	Y				Y	Y								Y	Y	Y	Y						Y										
11823	A_23_P14383	ACIN1																		Y			Y																						
19053	A_23_P376727	ACMSD			Y	Y	Y	Y	Y	Y	Y	Y						Y	Y			Y				Y	Y	Y	Y						Y										
1243	A_23_P103149	ACO2			Y	Y	Y	Y	Y	Y	Y	Y							Y	Y	Y		Y	Y	Y			Y	Y	Y	Y															
21524	A_23_P305481	ACO2			Y	Y	Y	Y	Y	Y	Y	Y	Y	Y				Y	Y	Y	Y	Y	Y	Y	Y	Y	Y	Y	Y	Y	Y	Y				Y				Y						
5111	A_23_P325726	ACOT11			Y																						Y			Y						Y										
20080	A_23_P3111	ACOT2			Y																									Y						Y										
7190	A_23_P14515	ACOT4							Y	Y																																						
3069	A_23_P86259	ACOT7			Y	Y	Y	Y		Y	Y	Y							Y	Y	Y	Y	Y	Y	Y	Y	Y	Y	Y	Y	Y	Y	Y					Y				Y			Y	Y
3533	A_23_P143218	ACOT8			Y													Y	Y	Y		Y	Y	Y			Y	Y	Y	Y															
5990	A_23_P125624	ACOT9	Y	Y	Y	Y		Y		Y	Y	Y	Y	Y	Y													Y			Y						Y										
4354	A_23_P206949	ACOX1			Y				Y	Y															Y			Y						Y										
21147	A_23_P10182	ACOX2			Y				Y	Y									Y							Y			Y						Y										
11522	A_23_P143034	ACOXL																										Y																			
6008	A_23_P56798	ACP1				Y														Y			Y																						
22349	A_23_P138776	ACP2			Y	Y	Y	Y	Y	Y	Y	Y	Y	Y				Y	Y	Y	Y	Y	Y	Y	Y	Y	Y	Y	Y	Y	Y	Y				Y										
4591	A_23_P142075	ACP5																										Y																			
20059	A_23_P57760	ACPL2	Y		Y	Y	Y	Y	Y	Y	Y	Y	Y	Y				Y	Y	Y			Y	Y			Y	Y	Y	Y															
17787	A_23_P155524	ACPP			Y													Y																													

2737	A_23_P151120	ACRBP			Y	Y		Y		Y								Y	Y	Y		Y		Y			Y																			
16572	A_23_P171237	ACRC			Y	Y	Y	Y	Y	Y	Y	Y	Y	Y				Y		Y		Y					Y	Y	Y	Y						Y										
4959	A_23_P54488	ACSBG1																Y																												
5829	A_23_P259797	ACSF3			Y	Y		Y	Y	Y									Y	Y		Y					Y			Y						Y										
13602	A_23_P110212	ACSL1			Y	Y		Y	Y	Y									Y								Y			Y						Y										
1157	A_23_P217564	ACSL4							Y	Y																																				
15715	A_23_P138404	ACSL5							Y	Y										Y										Y						Y										
2651	A_23_P7562	ACSL6																Y																												
13874	A_23_P106933	ACSM1							Y	Y	Y	Y	Y	Y						Y			Y							Y						Y								Y		
15759	A_23_P66328	ACSM2B			Y																						Y																			
21596	A_23_P33524	ACSM5													Y				Y	Y																										
19487	A_23_P120594	ACSS1			Y	Y		Y		Y																																				
19548	A_23_P210900	ACSS2			Y	Y	Y	Y	Y	Y	Y	Y	Y	Y				Y	Y	Y	Y	Y	Y	Y	Y	Y	Y			Y	Y	Y				Y										
10896	A_23_P61341	ACSS3																Y																												
15167	A_23_P150053	ACTA2			Y															Y			Y				Y																			
7803	A_23_P135769	ACTB			Y													Y		Y	Y	Y	Y																							
14961	A_23_P31323	ACTB			Y	Y	Y	Y	Y	Y	Y	Y						Y	Y	Y	Y	Y	Y	Y	Y	Y	Y				Y		Y													
9757	A_23_P205895	ACTC1			Y																						Y			Y						Y										
21679	A_23_P44037	ACTG1																		Y																										
15961	A_23_P145675	ACTL6B			Y	Y	Y	Y	Y	Y	Y	Y						Y	Y	Y		Y	Y	Y			Y			Y						Y										
4622	A_23_P9186	ACTL7B			Y																		Y				Y	Y	Y	Y																
13489	A_23_P160388	ACTL8				Y														Y								Y																		
19062	A_23_P105957	ACTN1							Y	Y								Y		Y	Y	Y	Y				Y																			
6978	A_23_P138881	ACTN3			Y				Y	Y								Y	Y	Y	Y	Y		Y			Y																			
860	A_23_P101655	ACTN4			Y	Y	Y	Y	Y	Y	Y	Y	Y	Y				Y	Y	Y		Y		Y			Y	Y	Y	Y			Y			Y										
7973	A_23_P315241	ACTN4																Y	Y	Y		Y		Y																						
17127	A_23_P149871	ACTR1A			Y																						Y																			
8845	A_23_P28279	ACTR1B																		Y		Y																								
10629	A_23_P123193	ACTR3B			Y	Y		Y	Y	Y	Y	Y	Y	Y						Y			Y							Y						Y										
19377	A_23_P80694	ACTR8	Y	Y	Y	Y	Y	Y	Y	Y	Y	Y	Y	Y				Y	Y	Y			Y	Y			Y	Y	Y	Y						Y										
9981	A_23_P148835	ACTRT2			Y								Y																																	
9883	A_23_P99036	ACVR1B																					Y																							
4350	A_23_P153932	ACVR2A			Y																																									
12420	A_23_P109943	ACVR2B			Y														Y									Y		Y						Y										
15991	A_23_P204121	ACVRL1				Y			Y	Y																																				
131	A_23_P57868	ACY1				Y																						Y																		
5418	A_23_P47102	ACY3							Y	Y									Y																											
16522	A_23_P159421	ACYP2																Y		Y	Y	Y	Y																							
18387	A_23_P256988	ACYP2																Y		Y	Y	Y	Y																							
4661	A_23_P210482	ADA	Y	Y	Y	Y		Y	Y	Y								Y	Y	Y	Y		Y	Y	Y		Y			Y						Y										
12955	A_23_P41514	ADAD1							Y	Y								Y				Y					Y																			
17794	A_23_P49136	ADAD2				Y																																								
4121	A_23_P14845	ADAL							Y	Y										Y																										
5522	A_23_P170618	ADAM15			Y	Y		Y		Y									Y	Y		Y					Y																			
8065	A_23_P143120	ADAM17			Y				Y	Y								Y		Y	Y						Y																			
15321	A_23_P72533	ADAM18																			Y																									
21337	A_23_P374082	ADAM19	Y		Y				Y	Y	Y	Y	Y	Y	Y												Y																			
11198	A_23_P73441	ADAM2																Y									Y			Y						Y										
13555	A_23_P48698	ADAM20			Y																																									
3632	A_23_P215625	ADAM22																										Y																		
19861	A_23_P351667	ADAM23			Y													Y									Y																			
21141	A_23_P170362	ADAM28			Y																						Y																			
2822	A_23_P126363	ADAM30				Y																								Y						Y										
18729	A_23_P210731	ADAM33			Y	Y	Y	Y	Y	Y	Y	Y											Y				Y																			
2482	A_23_P54858	ADAM6			Y																																									
8142	A_23_P94296	ADAM7				Y																																								
4378	A_23_P115759	ADAM8	Y	Y																										Y						Y										
17242	A_23_P72643	ADAM9			Y	Y		Y	Y	Y																																				
20372	A_23_P256425	ADAMDEC1	Y	Y	Y		Y				Y				Y												Y																			
22321	A_23_P211039	ADAMTS1							Y	Y																				Y						Y										
3990	A_23_P393645	ADAMTS13	Y	Y	Y	Y		Y	Y	Y																	Y	Y	Y	Y						Y										
6949	A_23_P41629	ADAMTS16				Y													Y																											
10962	A_23_P386953	ADAMTS16																		Y																										
1787	A_23_P259851	ADAMTS19			Y	Y		Y	Y	Y																	Y	Y	Y	Y																
8405	A_23_P84498	ADAMTS19			Y	Y		Y	Y	Y	Y	Y	Y	Y														Y		Y						Y										
20604	A_23_P213610	ADAMTS2	Y																																											
5537	A_23_P33266	ADAMTS20			Y																						Y																			
7919	A_23_P86117	ADAMTS4			Y														Y			Y					Y																			
18209	A_23_P213319	ADAMTS6			Y				Y	Y									Y	Y							Y			Y						Y										
9502	A_23_P11005	ADAMTS7			Y				Y	Y										Y		Y	Y																							
20608	A_23_P37624	ADAMTS7			Y		Y				Y																Y																			
21336	A_23_P33356	ADAMTS9			Y				Y	Y																	Y			Y						Y										
3124	A_23_P348304	ADAMTSL1																				Y					Y																			
6066	A_23_P312415	ADAMTSL1																Y	Y	Y		Y	Y	Y																						
7088	A_23_P20692	ADAMTSL2			Y	Y		Y		Y										Y		Y	Y																							
14000	A_23_P43940	ADAMTSL3																		Y																										
22389	A_23_P41340	ADAMTSL3			Y	Y		Y	Y	Y	Y	Y	Y	Y					Y	Y							Y	Y	Y	Y						Y										

7772	A_23_P391938	ADAMTSL4				Y																								Y						Y										
16506	A_23_P137786	ADAMTSL4			Y	Y		Y	Y	Y																																		
17686	A_23_P5246	ADAMTSL5			Y	Y		Y		Y										Y	Y						Y																	
8074	A_23_P145865	ADAP1																	Y								Y																	
19025	A_23_P49816	ADAP2			Y				Y	Y										Y							Y																	
19976	A_23_P200439	ADAR							Y	Y								Y	Y	Y	Y	Y		Y																				
3876	A_23_P211207	ADARB1			Y				Y	Y								Y	Y	Y		Y		Y			Y																	
9267	A_23_P86751	ADARB2																				Y																						
19364	A_23_P422105	ADAT3			Y	Y		Y		Y																	Y																	
7794	A_23_P103371	ADC																Y																										
764	A_23_P25945	ADCK1																Y		Y		Y																						
556	A_23_P94365	ADCK5			Y	Y		Y	Y	Y								Y	Y	Y		Y	Y	Y			Y			Y						Y										
236	A_23_P33539	ADCY2			Y																						Y																	
8348	A_23_P25955	ADCY4	Y																Y	Y			Y																					
10600	A_23_P204001	ADCY6																			Y																							
14056	A_23_P106949	ADCY7	Y		Y	Y		Y		Y										Y								Y		Y						Y										
8768	A_23_P169989	ADCY8	Y	Y	Y	Y		Y		Y										Y		Y					Y	Y	Y	Y														
1623	A_23_P49436	ADCY9																		Y	Y		Y																					
11849	A_23_P38712	ADCYAP1																			Y		Y																					
7526	A_23_P157109	ADCYAP1R1	Y	Y	Y													Y			Y	Y																						
12567	A_23_P212768	ADD1																												Y						Y										
7474	A_23_P134953	ADFP	Y	Y	Y	Y		Y	Y	Y																		Y		Y						Y										
13271	A_23_P58359	ADH1A			Y																																							
20790	A_23_P213184	ADH1B			Y																																							
1880	A_23_P390133	ADH5									Y		Y	Y						Y																								
13138	A_23_P18692	ADH5																		Y										Y						Y										
19875	A_23_P41498	ADH6	Y		Y																						Y			Y						Y										
8964	A_23_P157569	ADHFE1				Y			Y	Y									Y											Y						Y										
3680	A_23_P148162	ADI1			Y																						Y			Y						Y										
21700	A_23_P148194	ADI1	Y	Y	Y	Y	Y	Y	Y	Y	Y	Y															Y																	
15451	A_23_P46627	ADIPOR1			Y													Y	Y	Y	Y	Y	Y	Y	Y	Y	Y				Y													
8864	A_23_P48121	ADIPOR2			Y	Y		Y	Y	Y																	Y																	
11285	A_23_P125333	ADK			Y																																							
17887	A_23_P127948	ADM			Y	Y		Y	Y	Y																																		
17593	A_23_P57602	ADM2																										Y		Y						Y										
10379	A_23_P254179	ADNP			Y	Y		Y	Y	Y	Y	Y	Y	Y						Y							Y			Y						Y										
1714	A_23_P164468	ADNP2																	Y	Y		Y	Y				Y																	
5124	A_23_P202340	ADO			Y	Y		Y	Y	Y										Y																								
19824	A_23_P74299	ADORA1				Y			Y	Y								Y	Y	Y		Y		Y						Y						Y										
16426	A_23_P55477	ADORA2B							Y	Y																				Y						Y										
2549	A_23_P126540	ADORA3	Y		Y	Y		Y		Y								Y	Y	Y		Y		Y			Y			Y						Y										
11514	A_23_P137931	ADORA3	Y		Y													Y																										
10944	A_23_P14716	ADPGK	Y		Y				Y	Y																				Y						Y										
1404	A_23_P7020	ADPRH	Y		Y	Y	Y	Y	Y	Y	Y	Y						Y									Y																	
10705	A_23_P408070	ADPRH																					Y																					
15628	A_23_P48438	ADPRHL1																	Y																									
16856	A_23_P34568	ADPRHL2			Y	Y	Y	Y		Y	Y	Y						Y	Y	Y	Y	Y	Y	Y	Y	Y	Y	Y	Y	Y	Y	Y														
926	A_23_P21929	ADRA1B			Y																						Y																	
10466	A_23_P131924	ADRA1D			Y	Y	Y	Y	Y	Y	Y	Y	Y	Y						Y			Y				Y			Y						Y										
863	A_23_P378926	ADRA2B																												Y						Y										
5077	A_23_P210148	ADRA2B																	Y	Y																								
20278	A_23_P256158	ADRA2C																	Y			Y																						
21595	A_23_P60802	ADRA2C																	Y			Y																						
11855	A_23_P145024	ADRB2							Y	Y	Y	Y	Y	Y					Y				Y							Y						Y										
3339	A_23_P68662	ADRM1			Y	Y	Y	Y	Y	Y	Y	Y						Y	Y	Y		Y	Y	Y			Y	Y	Y	Y						Y						Y				
21548	A_23_P155097	ADSL	Y	Y	Y	Y	Y	Y	Y	Y	Y	Y	Y	Y				Y	Y	Y		Y		Y			Y			Y						Y										
16635	A_23_P201432	ADSS			Y				Y	Y																	Y																	
13764	A_23_P76823	ADSSL1							Y	Y			Y																															
7862	A_23_P145916	AEBP1			Y													Y		Y	Y	Y	Y										Y													
16798	A_23_P116913	AEBP2																		Y																								
2952	A_23_P206079	AEN																Y		Y		Y								Y						Y										
13306	A_23_P165061	AES			Y																																							
13539	A_23_P341309	AES			Y	Y	Y	Y		Y	Y	Y	Y	Y				Y	Y	Y		Y	Y	Y			Y			Y						Y							Y			
4812	A_23_P406341	AFAP1L2			Y																						Y			Y						Y										
12436	A_23_P32675	AFF3	Y	Y	Y	Y		Y	Y	Y																		Y		Y						Y										
14667	A_23_P373464	AFF3			Y	Y		Y		Y																																		
11769	A_23_P218977	AFF4																Y	Y																									
12601	A_23_P355289	AFG3L1																		Y		Y																						
4946	A_23_P124065	AFG3L2			Y	Y		Y		Y										Y	Y	Y					Y																	
14881	A_23_P135454	AFG3L2			Y	Y		Y		Y										Y		Y					Y																	
18695	A_23_P58201	AFP																												Y						Y										
4258	A_23_P428381	AFTPH			Y	Y		Y		Y	Y	Y	Y	Y				Y	Y	Y			Y	Y						Y						Y										
14711	A_23_P102542	AFTPH			Y	Y	Y	Y	Y	Y	Y	Y	Y	Y				Y	Y	Y	Y	Y	Y	Y	Y	Y	Y	Y	Y	Y	Y	Y	Y	Y	Y	Y				Y						
15103	A_23_P7099	AGA							Y	Y										Y																								
7166	A_23_P2198	AGAP2			Y																																							
7703	A_23_P111452	AGAP3																			Y																							
3292	A_23_P138401	AGAP7			Y	Y	Y	Y	Y	Y	Y	Y	Y	Y						Y		Y					Y	Y	Y	Y						Y										

20395	A_23_P309930	AGBL1				Y																							Y		Y						Y											
3622	A_23_P255082	AGBL2			Y	Y	Y	Y	Y	Y	Y	Y	Y	Y				Y	Y	Y	Y	Y	Y	Y	Y	Y	Y	Y	Y	Y	Y	Y	Y	Y	Y	Y										
15766	A_23_P154466	AGBL5																			Y																									
12623	A_23_P158143	AGFG1			Y	Y		Y	Y	Y																			Y		Y						Y											
13304	A_23_P169529	AGFG1			Y	Y		Y	Y	Y																			Y		Y						Y											
5621	A_23_P250689	AGFG2							Y	Y																																				
9464	A_23_P41970	AGGF1			Y	Y		Y	Y	Y	Y	Y	Y	Y				Y	Y	Y		Y	Y	Y			Y	Y	Y	Y							Y											
10316	A_23_P250559	AGGF1			Y	Y	Y	Y	Y	Y	Y	Y	Y	Y				Y	Y	Y	Y	Y	Y	Y	Y	Y	Y	Y	Y	Y	Y	Y	Y			Y										
22427	A_23_P200298	AGL			Y	Y	Y	Y	Y	Y	Y	Y	Y	Y					Y	Y		Y						Y		Y						Y										
14197	A_23_P103720	AGMAT																			Y																									
15989	A_23_P123774	AGPAT2			Y	Y		Y		Y										Y																										
416	A_23_P356466	AGPAT3			Y		Y		Y	Y	Y	Y	Y					Y		Y	Y																									
13118	A_23_P218743	AGPAT3																					Y																							
21958	A_23_P58960	AGPAT4			Y				Y	Y										Y			Y																							
4694	A_23_P82839	AGPAT6																Y	Y	Y	Y	Y		Y						Y						Y										
14207	A_23_P69809	AGPAT9			Y				Y	Y									Y	Y		Y					Y																			
2090	A_23_P368645	AGPS							Y	Y																																				
16028	A_23_P154386	AGPS			Y															Y							Y																			
10312	A_23_P31407	AGR2			Y				Y	Y	Y	Y	Y	Y				Y	Y	Y		Y	Y	Y			Y			Y						Y										
14985	A_23_P42811	AGR3	Y	Y	Y	Y		Y	Y	Y	Y	Y	Y	Y				Y	Y	Y			Y	Y			Y	Y	Y	Y						Y										
3195	A_23_P49167	AGRP			Y													Y									Y																			
13948	A_23_P115261	AGT																									Y			Y						Y										
20681	A_23_P169277	AGTPBP1																		Y										Y						Y										
20184	A_23_P211835	AGTR1	Y	Y	Y																						Y																			
1813	A_23_P62309	AGTR2																									Y	Y	Y	Y																
2258	A_23_P147215	AGTRAP																				Y																								
6804	A_23_P158596	AGTRAP			Y	Y		Y		Y																	Y																			
1181	A_23_P257231	AGXT2L1	Y	Y	Y													Y	Y	Y	Y		Y	Y	Y		Y			Y						Y										
6896	A_23_P17575	AHCY			Y																																									
9119	A_23_P201110	AHCYL1	Y	Y	Y	Y	Y	Y	Y	Y	Y	Y	Y	Y				Y	Y	Y	Y	Y	Y	Y	Y	Y	Y	Y	Y	Y	Y	Y	Y	Y	Y	Y								Y		
13133	A_23_P368126	AHCYL1	Y	Y	Y	Y	Y	Y	Y	Y	Y	Y						Y	Y	Y		Y	Y	Y			Y	Y	Y	Y						Y								Y		
9583	A_23_P70746	AHI1	Y		Y	Y		Y	Y	Y	Y	Y	Y	Y					Y	Y			Y				Y	Y	Y	Y						Y										
16487	A_23_P127789	AHNAK																												Y						Y										
16577	A_23_P215566	AHR			Y													Y	Y	Y				Y			Y																			
66	A_23_P117599	AHSA1			Y	Y		Y		Y										Y																										
4493	A_23_P372467	AHSA2			Y	Y	Y	Y	Y	Y	Y	Y	Y	Y				Y	Y	Y		Y	Y	Y			Y	Y	Y	Y						Y										
1811	A_23_P155509	AHSG			Y																						Y			Y						Y										
1985	A_23_P97451	AIDA			Y	Y		Y	Y	Y										Y							Y																			
5254	A_23_P214627	AIF1	Y		Y																									Y						Y										
27	A_23_P392384	AIF1L			Y																						Y			Y						Y										
9838	A_23_P94380	AIF1L			Y																						Y																			
18624	A_23_P258194	AIFM3			Y													Y	Y	Y		Y		Y																						
11525	A_23_P93431	AIG1	Y	Y	Y	Y	Y	Y	Y	Y	Y	Y	Y	Y				Y	Y	Y		Y	Y	Y			Y	Y	Y	Y						Y										
2137	A_23_P70785	AIM1							Y	Y																																				
18817	A_23_P115000	AIM1L			Y		Y		Y	Y	Y	Y															Y																	Y		
8014	A_23_P12100	AIM2	Y	Y	Y																						Y																			
11498	A_23_P75380	AIP			Y		Y				Y									Y	Y	Y	Y				Y	Y	Y	Y																
12089	A_23_P68740	AIRE							Y	Y																		Y																		
18301	A_23_P217080	AK1																	Y	Y																										
4691	A_23_P146387	AK3				Y												Y	Y	Y		Y	Y	Y				Y		Y	Y	Y	Y	Y	Y	Y										
134	A_23_P200015	AK5	Y	Y	Y	Y		Y		Y									Y	Y							Y	Y	Y	Y						Y										
13290	A_23_P105963	AK7			Y																																									
17628	A_23_P317105	AKAP10			Y														Y																											
8472	A_23_P204929	AKAP11			Y	Y	Y	Y	Y	Y	Y	Y	Y	Y				Y	Y	Y	Y	Y	Y	Y	Y	Y	Y	Y	Y	Y	Y	Y				Y								Y		
22329	A_23_P218160	AKAP13																					Y																							
2156	A_23_P14035	AKAP3			Y																						Y																			
2955	A_23_P259594	AKAP7																	Y																											
21232	A_23_P142380	AKAP8							Y	Y																				Y						Y										
18135	A_23_P153767	AKAP8L			Y	Y		Y		Y										Y		Y																								
1564	A_23_P309261	AKAP9			Y	Y	Y	Y	Y	Y	Y	Y	Y	Y				Y	Y	Y	Y	Y	Y	Y	Y	Y	Y	Y	Y	Y	Y	Y				Y										
19642	A_23_P256148	AKIRIN1			Y																Y																									
13012	A_23_P7843	AKIRIN2							Y	Y										Y																										
13344	A_23_P428819	AKIRIN2																	Y	Y																										
14320	A_23_P46186	AKR1A1				Y			Y	Y	Y	Y	Y	Y																																
21110	A_23_P258190	AKR1B1	Y		Y	Y	Y	Y	Y	Y	Y	Y			Y						Y		Y				Y	Y	Y	Y						Y										
17037	A_23_P93641	AKR1B10																												Y						Y										
11405	A_23_P257971	AKR1C1				Y																																								
16656	A_23_P1307	AKR1C1				Y																																								
515	A_23_P138543	AKR1C3				Y																																								
3317	A_23_P149928	AKR1C4				Y												Y										Y																		
19491	A_23_P161314	AKR1CL1																					Y																							
21808	A_23_P1322	AKR1CL2	Y	Y	Y	Y	Y	Y	Y	Y	Y	Y	Y	Y				Y	Y	Y				Y			Y	Y	Y	Y						Y										
3687	A_23_P255185	AKR1D1			Y	Y		Y	Y	Y																																				
14504	A_23_P115356	AKR7A2																									Y																			
19766	A_23_P14358	AKT1			Y	Y	Y	Y		Y	Y	Y						Y	Y	Y	Y	Y	Y	Y	Y	Y	Y				Y															
3241	A_23_P153502	AKT1S1			Y													Y	Y	Y		Y	Y	Y			Y			Y						Y										
4458	A_23_P373475	AKT2			Y	Y		Y		Y								Y	Y	Y		Y	Y	Y			Y																			

13568	A_23_P208870	AKT2																	Y																											
742	A_23_P160354	AKT3							Y	Y																																					
2809	A_23_P163622	AKTIP			Y																																									
1174	A_23_P324278	ALAD																	Y	Y																										
18746	A_23_P257834	ALB			Y	Y		Y	Y	Y	Y	Y	Y	Y															Y		Y						Y										
1080	A_23_P41227	ALCAM							Y	Y			Y																		Y						Y										
1427	A_23_P50472	ALDH16A1			Y				Y	Y										Y			Y								Y						Y										
22282	A_23_P99956	ALDH1A2																	Y																											
9736	A_23_P205959	ALDH1A3			Y	Y		Y	Y	Y									Y										Y																		
7214	A_23_P135294	ALDH1B1			Y				Y	Y										Y								Y			Y						Y										
1986	A_23_P87941	ALDH1L2			Y																																									
9521	A_23_P36745	ALDH2																Y																												
4768	A_23_P129896	ALDH3A2	Y		Y	Y	Y	Y		Y	Y	Y	Y	Y				Y									Y			Y						Y										
12775	A_23_P202640	ALDH3B1			Y				Y	Y								Y	Y								Y																			
21856	A_23_P24308	ALDH3B2			Y													Y									Y																			
6630	A_23_P158945	ALDH4A1							Y	Y									Y	Y							Y	Y	Y	Y						Y										
6887	A_23_P170337	ALDH4A1							Y	Y	Y	Y	Y	Y						Y										Y						Y										
16485	A_23_P128967	ALDH6A1	Y	Y	Y	Y		Y	Y	Y	Y	Y	Y	Y				Y		Y																										
21818	A_23_P70233	ALDH7A1			Y																						Y																			
12487	A_23_P11884	ALDH9A1	Y	Y	Y	Y	Y	Y		Y	Y	Y						Y	Y	Y	Y	Y	Y	Y	Y	Y	Y	Y	Y	Y	Y	Y	Y			Y				Y						
2005	A_23_P88961	ALDOA			Y	Y		Y	Y	Y										Y	Y	Y					Y																			
9314	A_23_P415974	ALDOB							Y	Y								Y											Y						Y										
20640	A_23_P78112	ALDOC																		Y													Y													
2975	A_23_P66306	ALG1			Y	Y		Y		Y								Y	Y	Y		Y		Y			Y	Y	Y	Y																
10527	A_23_P60718	ALG12			Y	Y	Y	Y		Y	Y	Y							Y	Y							Y	Y	Y	Y						Y										
3977	A_23_P257425	ALG14																		Y			Y																							
17281	A_23_P7005	ALG3			Y	Y	Y	Y	Y	Y	Y	Y	Y	Y				Y	Y	Y	Y	Y	Y	Y	Y	Y	Y	Y	Y	Y	Y	Y				Y				Y						
14180	A_23_P13554	ALG8			Y																																									
13529	A_23_P324304	ALK							Y	Y																																				
18513	A_23_P170096	ALK																					Y																							
8395	A_23_P14544	ALKBH1			Y				Y	Y																																				
18334	A_23_P35987	ALKBH3																Y					Y																							
13184	A_23_P255523	ALKBH4			Y														Y	Y			Y																							
2184	A_23_P153046	ALKBH5	Y	Y	Y															Y							Y																			
9317	A_23_P39396	ALKBH6			Y	Y		Y		Y									Y	Y		Y																								
2862	A_23_P164760	ALKBH7			Y	Y		Y	Y	Y	Y	Y	Y	Y					Y	Y		Y	Y				Y	Y	Y	Y						Y										
1090	A_23_P312174	ALMS1			Y	Y	Y	Y	Y	Y	Y	Y	Y	Y					Y	Y		Y					Y	Y	Y	Y						Y										
21841	A_23_P435659	ALMS1P			Y	Y		Y		Y																																				
6202	A_23_P152906	ALOX12							Y	Y																		Y																		
17219	A_23_P83634	ALOX12B			Y																						Y																			
16081	A_23_P55373	ALOX15				Y																																								
15929	A_23_P60627	ALOX15B							Y	Y																																				
4140	A_23_P2793	ALOX5AP	Y		Y	Y		Y		Y																																				
8509	A_23_P144531	ALPK1			Y	Y		Y		Y	Y	Y	Y	Y						Y							Y	Y	Y	Y						Y										
19397	A_23_P15876	ALPK2			Y				Y	Y								Y	Y	Y		Y		Y																						
8933	A_23_P348728	ALPK3																Y	Y								Y																			
10498	A_23_P97043	ALPL																				Y																								
20299	A_23_P79587	ALPP				Y																																								
4515	A_23_P68182	ALPPL2																									Y			Y						Y										
4658	A_23_P209438	ALS2			Y	Y	Y	Y		Y	Y	Y						Y	Y	Y		Y	Y	Y			Y																			
14451	A_23_P377664	ALS2			Y	Y		Y	Y	Y									Y	Y		Y					Y																			
13517	A_23_P328642	ALS2CR11																		Y																										
13111	A_23_P370097	ALS2CR4																					Y																							
19385	A_23_P119880	ALS2CR4							Y	Y																																				
22572	A_23_P165828	ALS2CR8			Y				Y	Y																																				
7026	A_23_P83798	ALX1																Y									Y			Y						Y										
16469	A_23_P95200	ALX1			Y																						Y																			
6916	A_23_P389532	AMAC1							Y	Y	Y	Y	Y	Y					Y	Y							Y	Y	Y	Y						Y										
1740	A_23_P110676	AMACR							Y	Y	Y	Y	Y	Y																																
9185	A_23_P363754	AMBP			Y	Y	Y	Y	Y	Y	Y	Y	Y	Y													Y			Y						Y										
17480	A_23_P256504	AMBP			Y	Y		Y	Y	Y	Y	Y	Y	Y													Y	Y	Y	Y						Y						Y				
8834	A_23_P353742	AMBRA1																									Y	Y	Y	Y						Y										
20848	A_23_P203249	AMBRA1																		Y																										
21972	A_23_P214121	AMD1			Y																																									
12553	A_23_P162616	AMDHD1			Y														Y								Y																			
9991	A_23_P49351	AMDHD2			Y	Y		Y		Y										Y	Y	Y																								
15465	A_23_P141005	AMFR																		Y																										
2628	A_23_P78944	AMH																		Y		Y																								
5618	A_23_P1759	AMICA1			Y																		Y																							
10535	A_23_P14083	AMIGO2				Y																						Y																		
4001	A_23_P212589	AMIGO3																		Y																										
11032	A_23_P40039	AMMECR1L			Y	Y		Y	Y	Y										Y	Y		Y				Y			Y						Y										
20488	A_23_P209764	AMMECR1L			Y				Y	Y	Y	Y	Y	Y				Y	Y	Y		Y		Y			Y																			
13026	A_23_P48455	AMN				Y														Y			Y																							
12437	A_23_P138796	AMOTL1							Y	Y								Y	Y																											
19433	A_23_P166686	AMOTL2																					Y																							
19409	A_23_P201591	AMPD2							Y	Y	Y	Y	Y	Y													Y																			

2668	A_23_P31273	AMPH			Y	Y	Y	Y	Y	Y	Y	Y							Y			Y					Y	Y	Y	Y						Y										
14713	A_23_P257164	AMT	Y	Y	Y													Y		Y		Y					Y	Y	Y	Y						Y										
21948	A_23_P110224	AMTN			Y	Y		Y	Y	Y																	Y	Y	Y	Y						Y										
4066	A_23_P12204	AMY2A			Y																																									
11830	A_23_P17204	ANAPC1																		Y																										
9124	A_23_P250994	ANAPC10																					Y																							
11432	A_23_P253062	ANAPC2			Y				Y	Y								Y	Y	Y		Y	Y	Y						Y						Y										
4259	A_23_P144427	ANAPC4			Y	Y	Y	Y	Y	Y	Y	Y						Y	Y	Y	Y	Y		Y			Y	Y	Y	Y						Y										
6691	A_23_P317800	ANAPC4			Y	Y	Y	Y	Y	Y	Y	Y	Y	Y				Y	Y	Y	Y	Y	Y	Y	Y	Y	Y	Y	Y	Y	Y	Y	Y	Y	Y	Y		Y				Y			Y	Y
3127	A_23_P377830	ANAPC5			Y	Y		Y		Y								Y	Y	Y		Y		Y			Y																			
15197	A_23_P204448	ANAPC5																			Y																									
3747	A_23_P428738	ANG	Y	Y	Y	Y		Y	Y	Y								Y									Y	Y	Y	Y																
12657	A_23_P97697	ANGEL2			Y	Y		Y		Y										Y	Y	Y	Y				Y																			
13519	A_23_P343237	ANGEL2																					Y																							
18359	A_23_P216023	ANGPT1			Y														Y								Y																			
7907	A_23_P374104	ANGPTL2																		Y			Y																							
8873	A_23_P20864	ANGPTL2																		Y																										
11784	A_23_P160262	ANGPTL3																		Y																										
4766	A_23_P159328	ANGPTL4																									Y	Y	Y	Y																
8756	A_23_P50276	ANGPTL6			Y																						Y																			
1654	A_23_P114862	ANGPTL7																				Y																								
22162	A_23_P216108	ANK1				Y																																								
1684	A_23_P202269	ANK3											Y							Y	Y		Y																							
22291	A_23_P370544	ANKAR			Y	Y		Y	Y	Y								Y	Y	Y		Y	Y	Y			Y	Y	Y	Y						Y										
8757	A_23_P207620	ANKFY1			Y													Y	Y	Y				Y			Y	Y	Y	Y																
8237	A_23_P213766	ANKH							Y	Y								Y												Y						Y										
20680	A_23_P213326	ANKHD1			Y	Y	Y	Y	Y	Y	Y	Y	Y	Y						Y		Y	Y				Y	Y	Y	Y						Y										
4206	A_23_P58443	ANKHD1-EIF4EBP3	Y	Y																			Y																							
12633	A_23_P35943	ANKK1			Y	Y		Y	Y	Y																		Y		Y						Y										
18193	A_23_P142496	ANKLE1							Y	Y																																				
7674	A_23_P10859	ANKLE2			Y				Y	Y																																				
4383	A_23_P131463	ANKMY1																	Y																											
16378	A_23_P20002	ANKMY2			Y	Y		Y	Y	Y								Y	Y	Y		Y	Y	Y			Y	Y	Y	Y						Y										
3677	A_23_P41634	ANKRA2			Y	Y	Y	Y	Y	Y	Y	Y	Y	Y				Y	Y	Y	Y	Y	Y	Y	Y	Y	Y	Y	Y	Y	Y	Y	Y	Y	Y	Y								Y		
13005	A_23_P205048	ANKRD10			Y	Y	Y	Y	Y	Y	Y	Y	Y	Y				Y	Y	Y		Y		Y			Y	Y	Y	Y						Y										
11762	A_23_P3632	ANKRD11			Y																																									
16744	A_23_P163639	ANKRD11									Y		Y	Y				Y	Y	Y	Y	Y		Y																						
11880	A_23_P130293	ANKRD12				Y													Y									Y																		
14345	A_23_P380881	ANKRD13B																Y		Y		Y																								
16978	A_23_P114616	ANKRD13C																										Y																		
2420	A_23_P144378	ANKRD17				Y																						Y																		
12770	A_23_P158030	ANKRD18A																Y		Y							Y																			
14685	A_23_P305121	ANKRD23			Y		Y				Y											Y					Y																			
6168	A_23_P115593	ANKRD26			Y	Y		Y	Y	Y										Y								Y																		
7072	A_23_P97123	ANKRD26L1							Y	Y																																				
18365	A_23_P153637	ANKRD27							Y	Y																																				
1822	A_23_P109794	ANKRD28																Y	Y				Y				Y																			
5296	A_23_P12533	ANKRD30A	Y	Y	Y	Y		Y	Y	Y	Y	Y	Y	Y					Y				Y				Y	Y	Y	Y						Y										
15376	A_23_P144684	ANKRD32				Y														Y																										
566	A_23_P325690	ANKRD35	Y	Y	Y	Y		Y	Y	Y													Y				Y			Y						Y										
8300	A_23_P253622	ANKRD36B				Y			Y	Y	Y	Y	Y	Y														Y		Y						Y										
8390	A_23_P131322	ANKRD36B							Y	Y																				Y						Y										
11710	A_23_P210134	ANKRD36B							Y	Y																				Y						Y										
4990	A_23_P95027	ANKRD39			Y	Y	Y	Y		Y	Y	Y						Y									Y			Y						Y										
11192	A_23_P94095	ANKRD46				Y												Y	Y	Y		Y	Y	Y																						
12410	A_23_P24365	ANKRD49																					Y																							
16817	A_23_P256047	ANKRD5			Y				Y	Y	Y	Y	Y	Y						Y							Y																			
14543	A_23_P81012	ANKRD50				Y														Y								Y		Y						Y										
9486	A_23_P337878	ANKRD53																Y																												
18110	A_23_P120915	ANKRD54			Y															Y							Y																			
19152	A_23_P120354	ANKRD57			Y	Y		Y	Y	Y										Y			Y				Y	Y	Y	Y						Y										
21207	A_23_P70297	ANKRD6				Y	Y		Y	Y	Y	Y	Y	Y						Y							Y	Y	Y	Y						Y										
4537	A_23_P131875	ANKRD60							Y	Y									Y			Y	Y																							
19644	A_23_P250231	ANKRD7				Y			Y	Y																				Y						Y										
16763	A_23_P307525	ANKRD9			Y	Y		Y	Y	Y								Y	Y	Y	Y	Y	Y	Y	Y	Y	Y				Y															
7379	A_23_P356717	ANKS1B			Y	Y		Y	Y	Y	Y	Y	Y	Y													Y			Y						Y						Y				
14926	A_23_P21842	ANKS1B			Y	Y		Y		Y									Y	Y																										
17878	A_23_P362183	ANKS6	Y	Y	Y				Y	Y	Y	Y	Y	Y				Y		Y			Y				Y			Y						Y										
8789	A_23_P356684	ANLN																Y									Y																			
9296	A_23_P157099	ANLN																Y																												
10893	A_23_P53645	ANO6																					Y																							
10085	A_23_P3247	ANP32A			Y	Y	Y	Y	Y	Y	Y	Y	Y	Y							Y		Y				Y			Y						Y										
18742	A_23_P20615	ANP32B			Y				Y	Y								Y	Y	Y	Y	Y	Y	Y	Y	Y	Y			Y	Y	Y	Y			Y				Y						
15754	A_23_P92524	ANP32C																				Y																								
18604	A_23_P160934	ANP32E							Y	Y																																				
7627	A_23_P88621	ANPEP							Y	Y												Y																								
15549	A_23_P84576	ANTXR1																	Y																											

13213	A_23_P170733	ANTXR2																	Y																										
19804	A_23_P1170	ANUBL1				Y														Y			Y																					
17085	A_23_P94501	ANXA1	Y	Y	Y																																							
13706	A_23_P58328	ANXA10							Y	Y								Y	Y	Y		Y	Y	Y			Y	Y	Y	Y						Y										
12688	A_23_P35398	ANXA11																		Y			Y																					
21043	A_23_P146644	ANXA2			Y	Y		Y		Y			Y																															
9209	A_23_P121716	ANXA3				Y															Y																							
8748	A_23_P16976	ANXA4																	Y																									
12909	A_23_P69720	ANXA5				Y																																						
14861	A_23_P86573	ANXA7			Y																																							
11320	A_23_P103614	ANXA9			Y				Y	Y	Y	Y	Y	Y													Y			Y						Y										
19311	A_23_P145715	AOAH	Y	Y	Y				Y	Y																	Y																			
15054	A_23_P4133	AOC2				Y																	Y																					
20409	A_23_P26941	AOC3			Y	Y	Y	Y	Y	Y	Y	Y						Y	Y								Y																			
2032	A_23_P133	AOF2			Y																																							
14714	A_23_P154037	AOX1																									Y	Y	Y	Y						Y										
13566	A_23_P6392	AP1B1			Y	Y		Y	Y	Y								Y	Y	Y	Y	Y	Y	Y	Y	Y	Y	Y	Y	Y	Y	Y				Y										
14723	A_23_P2965	AP1G2																	Y	Y	Y	Y																						
3101	A_23_P55802	AP1M1			Y	Y	Y	Y	Y	Y	Y	Y	Y	Y				Y	Y	Y	Y	Y	Y	Y	Y	Y	Y				Y		Y	Y	Y											
17631	A_23_P157404	AP1S1																Y				Y																						
10933	A_23_P217384	AP1S2	Y		Y															Y	Y		Y										Y	Y												
12349	A_23_P385295	AP1S3			Y				Y	Y																																				
21177	A_23_P67796	AP1S3			Y	Y		Y	Y	Y																	Y																			
10427	A_23_P164889	AP2A1			Y		Y		Y	Y	Y	Y						Y	Y	Y		Y	Y	Y			Y			Y	Y	Y	Y	Y	Y	Y										
7797	A_23_P55149	AP2B1			Y	Y		Y		Y																				Y						Y										
13161	A_23_P155615	AP2M1			Y	Y	Y	Y		Y	Y	Y						Y	Y	Y	Y	Y	Y	Y	Y	Y	Y				Y		Y	Y	Y									Y		
13606	A_23_P50530	AP2S1			Y															Y							Y																			
19382	A_23_P136635	AP3B1			Y	Y	Y	Y	Y	Y	Y	Y	Y	Y				Y	Y	Y				Y			Y			Y						Y										
16131	A_23_P108135	AP3D1																												Y						Y										
2027	A_23_P158938	AP3M1							Y	Y																																				
20413	A_23_P69958	AP3S1													Y																															
1708	A_23_P100011	AP3S2				Y													Y	Y		Y																								
19859	A_23_P157051	AP4M1																Y																												
21881	A_23_P158231	APBA2							Y	Y	Y	Y	Y	Y																Y						Y										
20173	A_23_P67555	APBA3			Y	Y		Y	Y	Y	Y	Y	Y	Y														Y		Y						Y										
11755	A_23_P1998	APBB1			Y														Y	Y			Y				Y																			
1429	A_23_P401700	APBB1IP													Y																															
3702	A_23_P12549	APBB1IP			Y															Y	Y		Y				Y																			
4894	A_23_P10701	APBB2	Y	Y	Y	Y	Y	Y	Y	Y	Y	Y	Y	Y				Y	Y	Y		Y	Y	Y			Y	Y	Y	Y						Y										
9573	A_23_P110445	APBB3				Y																						Y		Y						Y										
3366	A_23_P70214	APC			Y	Y	Y	Y	Y	Y	Y	Y	Y	Y						Y		Y	Y				Y	Y	Y	Y																
13932	A_23_P337262	APCDD1			Y				Y	Y																																				
17770	A_23_P118983	APCDD1																									Y																			
958	A_23_P96827	APCS			Y		Y				Y																Y			Y						Y										
15381	A_23_P151649	APEX1																		Y							Y																			
20179	A_23_P256682	APEX2			Y	Y		Y		Y									Y	Y							Y	Y	Y	Y						Y										
18896	A_23_P930	APH1A			Y	Y		Y		Y								Y		Y	Y	Y	Y				Y	Y	Y	Y																
4967	A_23_P205997	APH1B							Y	Y																				Y						Y										
11958	A_23_P203250	API5			Y	Y		Y	Y	Y										Y		Y	Y				Y	Y	Y	Y																
5800	A_23_P2062	APIP			Y															Y			Y							Y						Y										
19641	A_23_P138016	APITD1			Y	Y	Y	Y		Y	Y	Y								Y			Y																							
1017	A_23_P147755	APLN			Y																						Y																			
3971	A_23_P202939	APLP2																			Y																									
3816	A_23_P201429	APOA2			Y	Y		Y	Y	Y	Y	Y	Y	Y													Y																			
1123	A_23_P87036	APOA4				Y														Y		Y								Y						Y										
17197	A_23_P75630	APOA5				Y																					Y	Y	Y	Y																
6306	A_23_P79591	APOB			Y	Y	Y	Y		Y	Y	Y	Y	Y					Y								Y	Y	Y	Y						Y										
9341	A_23_P54773	APOB48R			Y	Y		Y		Y										Y		Y	Y				Y																			
4665	A_23_P70387	APOBEC2				Y																						Y																		
100	A_23_P303876	APOBEC3A			Y													Y	Y	Y	Y	Y	Y	Y	Y	Y					Y															
22344	A_23_P109539	APOBEC3B	Y	Y	Y													Y	Y	Y	Y	Y	Y	Y	Y	Y	Y				Y															
2233	A_23_P120931	APOBEC3C																	Y																											
20057	A_23_P211511	APOBEC3D																Y	Y	Y		Y		Y																						
2640	A_23_P369966	APOBEC3F																Y	Y	Y	Y	Y	Y	Y	Y	Y					Y															
20558	A_23_P357101	APOBEC3F			Y		Y		Y	Y	Y	Y						Y	Y	Y				Y			Y																			
19807	A_23_P143713	APOBEC3G	Y	Y	Y		Y				Y																																			
21435	A_23_P4649	APOC1			Y				Y	Y										Y			Y				Y																			
11030	A_23_P417743	APOC2																			Y																									
2166	A_23_P203183	APOC3	Y															Y																												
5626	A_23_P164654	APOE			Y				Y	Y								Y	Y	Y	Y	Y	Y	Y	Y	Y	Y			Y	Y	Y	Y	Y	Y	Y										
21024	A_23_P38244	APOH	Y	Y	Y	Y		Y	Y	Y																	Y	Y	Y	Y																
17424	A_23_P17837	APOL1			Y	Y		Y		Y																																				
7295	A_23_P211489	APOL2			Y	Y		Y		Y										Y		Y	Y				Y																			
3206	A_23_P29237	APOL3							Y	Y										Y																										
5114	A_23_P211479	APOL4			Y				Y	Y													Y							Y						Y										
10964	A_23_P166438	APOL5				Y																	Y				Y	Y	Y	Y						Y										
20192	A_23_P155049	APOL6	Y	Y	Y	Y	Y	Y		Y	Y	Y	Y					Y		Y		Y					Y	Y	Y	Y			Y													

13225	A_23_P48217	APOLD1			Y																								Y																			
2654	A_23_P42265	APOM	Y	Y	Y	Y	Y	Y	Y	Y	Y	Y	Y	Y				Y	Y	Y	Y	Y	Y	Y	Y	Y	Y	Y	Y	Y	Y	Y	Y			Y		Y				Y			Y	
5076	A_23_P61127	APOO																		Y																										
13792	A_23_P6216	APP																Y																												
14891	A_23_P207280	APPBP2			Y	Y		Y	Y	Y	Y	Y	Y	Y													Y	Y	Y	Y						Y										
9193	A_23_P166663	APPL1				Y			Y	Y																		Y		Y						Y										
4951	A_23_P105747	APPL2							Y	Y			Y							Y																										
9802	A_23_P117955	APRT			Y	Y	Y	Y	Y	Y	Y	Y						Y	Y	Y		Y	Y	Y			Y	Y	Y	Y																
20279	A_23_P252775	APTX			Y	Y		Y		Y								Y	Y	Y			Y	Y																						
2885	A_23_P372834	AQP1																	Y	Y		Y	Y																							
5130	A_23_P19894	AQP1																Y	Y	Y		Y	Y	Y																						
1702	A_23_P354962	AQP10			Y																						Y																			
8349	A_23_P417974	AQP11			Y	Y		Y	Y	Y										Y	Y		Y				Y			Y						Y										
22128	A_23_P204721	AQP2																												Y						Y										
1010	A_23_P112482	AQP3			Y	Y		Y	Y	Y																																				
13498	A_23_P366812	AQP5			Y	Y	Y	Y		Y	Y	Y	Y	Y													Y	Y	Y	Y						Y										
836	A_23_P5666	AQP7																												Y						Y										
1430	A_23_P106362	AQP9	Y	Y	Y	Y		Y	Y	Y	Y	Y	Y	Y				Y		Y		Y					Y	Y	Y	Y						Y										
15178	A_23_P54330	AQR			Y	Y		Y	Y	Y								Y	Y	Y		Y	Y	Y																						
1924	A_23_P73511	ARAF			Y	Y	Y	Y		Y	Y	Y	Y	Y				Y	Y	Y	Y	Y	Y	Y	Y	Y	Y				Y															
10089	A_23_P98770	ARAP1			Y		Y				Y							Y	Y	Y	Y	Y	Y	Y	Y	Y	Y			Y	Y	Y				Y										
17747	A_23_P167389	ARAP3																					Y																							
18182	A_23_P203103	ARCN1				Y																																								
11622	A_23_P148546	ARD1A			Y	Y	Y	Y	Y	Y	Y	Y	Y	Y				Y	Y	Y		Y	Y	Y			Y	Y	Y	Y						Y										
3686	A_23_P155890	ARD1B			Y	Y	Y	Y	Y	Y	Y	Y	Y	Y				Y	Y	Y		Y	Y	Y			Y	Y	Y	Y						Y										
21562	A_23_P259071	AREG																										Y																		
18612	A_23_P36521	ARF3			Y													Y	Y	Y	Y	Y		Y			Y																			
5821	A_23_P431789	ARF4				Y																																								
6648	A_23_P254605	ARF5			Y		Y				Y									Y			Y				Y																			
14050	A_23_P48691	ARF6			Y													Y	Y	Y	Y	Y	Y	Y	Y	Y					Y															
8455	A_23_P210379	ARFGAP1																Y		Y	Y	Y	Y																							
6579	A_23_P161552	ARFGAP2			Y		Y				Y							Y	Y	Y	Y	Y	Y	Y	Y	Y	Y				Y															
3634	A_23_P68970	ARFGAP3			Y																																									
19988	A_23_P82941	ARFGEF1																	Y																											
12599	A_23_P5995	ARFGEF2																					Y					Y		Y						Y										
22431	A_23_P111321	ARG1																									Y																			
21825	A_23_P128728	ARG2																												Y						Y										
3014	A_23_P162776	ARGLU1			Y				Y	Y																	Y																			
8715	A_23_P21548	ARHGAP10	Y		Y	Y		Y	Y	Y								Y	Y	Y	Y		Y	Y	Y		Y	Y	Y	Y			Y	Y												
17842	A_23_P255166	ARHGAP15																Y																												
3330	A_23_P3716	ARHGAP17			Y																																									
17331	A_23_P1387	ARHGAP19																	Y																											
4225	A_23_P115605	ARHGAP21				Y			Y	Y	Y	Y	Y	Y																Y						Y							Y			
8414	A_23_P75310	ARHGAP22																		Y			Y																							
6429	A_23_P142974	ARHGAP25	Y		Y		Y		Y	Y	Y	Y						Y	Y	Y			Y	Y			Y																			
10419	A_23_P141336	ARHGAP27			Y	Y		Y		Y	Y	Y	Y	Y					Y	Y		Y	Y				Y	Y	Y	Y						Y										
19678	A_23_P149562	ARHGAP29																		Y																										
22554	A_23_P35045	ARHGAP30	Y		Y	Y	Y	Y	Y	Y	Y	Y			Y				Y	Y	Y		Y				Y				Y		Y	Y	Y											
7254	A_23_P159927	ARHGAP4			Y								Y					Y	Y	Y	Y	Y	Y	Y	Y	Y					Y															
8765	A_23_P255573	ARHGAP6																					Y																							
10587	A_23_P64661	ARHGAP9	Y		Y										Y																															
7673	A_23_P207760	ARHGDIA			Y													Y	Y	Y	Y	Y	Y	Y	Y	Y	Y				Y															
20991	A_23_P218253	ARHGDIG																Y																												
16708	A_23_P101321	ARHGEF1			Y	Y		Y		Y								Y	Y	Y		Y	Y	Y			Y	Y	Y	Y																
21904	A_23_P216282	ARHGEF10							Y	Y								Y												Y						Y										
10475	A_23_P381	ARHGEF10L							Y	Y																																				
662	A_23_P127642	ARHGEF12	Y	Y	Y	Y	Y	Y		Y	Y	Y						Y	Y	Y	Y	Y	Y	Y	Y	Y	Y	Y	Y	Y	Y	Y	Y	Y	Y	Y										
19009	A_23_P114670	ARHGEF16							Y	Y																																				
20870	A_23_P137890	ARHGEF19			Y				Y	Y	Y	Y	Y	Y				Y	Y	Y		Y		Y			Y			Y						Y										
11187	A_23_P51699	ARHGEF2																Y		Y	Y	Y	Y				Y																			
6197	A_23_P143885	ARHGEF3			Y															Y			Y				Y						Y													
21845	A_23_P253221	ARHGEF4							Y	Y									Y											Y						Y										
2445	A_23_P93988	ARHGEF5																					Y																							
8934	A_23_P85024	ARHGEF6			Y	Y		Y		Y																																				
3277	A_23_P105845	ARHGEF7			Y	Y		Y	Y	Y																																				
17814	A_23_P321855	ARHGEF7																												Y						Y										
17869	A_23_P251701	ARHGEF9							Y	Y																				Y						Y										
21867	A_23_P217879	ARID1A			Y	Y		Y	Y	Y										Y	Y	Y	Y				Y	Y	Y	Y						Y										
19388	A_23_P70699	ARID1B																Y	Y	Y			Y	Y																						
10383	A_23_P308469	ARID2	Y	Y	Y	Y	Y	Y	Y	Y	Y	Y						Y	Y	Y		Y	Y	Y			Y	Y	Y	Y	Y	Y	Y	Y	Y	Y										
18006	A_23_P16516	ARID3A			Y															Y	Y	Y																								
2891	A_23_P88580	ARID3B																Y																												
18892	A_23_P117515	ARID4A			Y				Y	Y										Y																										
15978	A_23_P201951	ARID4B			Y	Y	Y	Y	Y	Y	Y	Y						Y	Y	Y		Y	Y	Y			Y	Y	Y	Y						Y										
16386	A_23_P205855	ARIH1																		Y							Y																			
4054	A_23_P139747	ARL1			Y	Y		Y		Y																																				
10772	A_23_P76572	ARL11	Y		Y															Y	Y						Y																			

16619	A_23_P10081	ARL13B			Y	Y		Y	Y	Y										Y								Y			Y						Y									
9512	A_23_P92161	ARL14			Y				Y	Y								Y													Y						Y									
22544	A_23_P33484	ARL15																						Y																						
18623	A_23_P371885	ARL17P1			Y	Y		Y		Y													Y																							
8186	A_23_P98254	ARL2			Y	Y	Y	Y		Y	Y	Y						Y		Y	Y	Y	Y					Y																		
6251	A_23_P15182	ARL2BP																																												
17093	A_23_P63711	ARL3			Y	Y	Y	Y	Y	Y	Y	Y	Y	Y						Y		Y	Y					Y	Y	Y	Y						Y									
4227	A_23_P317620	ARL4C			Y	Y		Y	Y	Y	Y	Y	Y	Y						Y	Y	Y	Y					Y			Y						Y						Y				
8275	A_23_P251551	ARL4C			Y	Y		Y	Y	Y	Y	Y	Y	Y						Y	Y	Y	Y					Y															Y				
7890	A_23_P55184	ARL4D			Y		Y		Y	Y	Y	Y						Y			Y							Y																		
7972	A_23_P101949	ARL5A			Y	Y		Y		Y																		Y																		
3809	A_23_P378588	ARL5B			Y	Y		Y	Y	Y								Y	Y	Y		Y		Y				Y			Y						Y									
3855	A_23_P161407	ARL5B	Y	Y	Y	Y		Y	Y	Y								Y	Y	Y		Y		Y				Y			Y						Y									
9125	A_23_P80426	ARL6																					Y																							
21966	A_23_P47848	ARL6IP4																		Y			Y					Y																		
18317	A_23_P166640	ARL6IP5																		Y																										
21909	A_23_P28169	ARL6IP6																													Y						Y									
1083	A_23_P415040	ARL8A			Y																		Y								Y						Y									
7855	A_23_P138271	ARL8A																				Y																								
10319	A_23_P385217	ARL8B			Y	Y	Y	Y		Y	Y	Y								Y			Y					Y																		
18258	A_23_P147678	ARMC1							Y	Y																																				
510	A_23_P254140	ARMC2																			Y																									
9923	A_23_P86540	ARMC3			Y	Y		Y	Y	Y																		Y			Y						Y									
4889	A_23_P159548	ARMC4			Y				Y	Y								Y		Y			Y					Y																		
14446	A_23_P63600	ARMC4			Y																							Y																		
5444	A_23_P15375	ARMC7							Y	Y													Y																							
717	A_23_P18384	ARMC8			Y	Y	Y	Y		Y	Y	Y								Y			Y					Y	Y	Y	Y															
986	A_23_P29784	ARMC8			Y	Y	Y	Y	Y	Y	Y	Y	Y	Y						Y		Y	Y					Y	Y	Y	Y						Y									
564	A_23_P209731	ARMC9			Y	Y	Y	Y	Y	Y	Y	Y	Y	Y				Y	Y		Y							Y	Y	Y	Y						Y									
14097	A_23_P22682	ARMCX1			Y				Y	Y									Y	Y								Y			Y						Y									
15914	A_23_P73750	ARMCX2			Y				Y	Y								Y										Y			Y						Y									
10633	A_23_P217611	ARMCX3			Y	Y	Y	Y		Y	Y	Y						Y	Y	Y				Y				Y	Y	Y	Y						Y									
21707	A_23_P85188	ARMCX5																		Y			Y																							
21744	A_23_P62348	ARMCX6																		Y	Y																									
14819	A_23_P132793	ARMET			Y														Y									Y	Y	Y	Y						Y									
3187	A_23_P112784	ARNT2	Y		Y	Y	Y	Y	Y	Y	Y	Y	Y	Y	Y	Y	Y	Y	Y	Y	Y	Y	Y	Y	Y	Y	Y	Y	Y	Y	Y	Y	Y	Y	Y	Y										
3742	A_23_P83579	ARNT2	Y	Y	Y	Y	Y	Y	Y	Y	Y	Y	Y	Y	Y	Y	Y	Y	Y	Y	Y	Y	Y	Y	Y	Y	Y	Y	Y	Y	Y	Y	Y	Y	Y	Y										
20592	A_23_P162037	ARNTL																		Y																										
8947	A_23_P20193	ARPC1B			Y	Y	Y	Y	Y	Y	Y	Y						Y	Y	Y	Y	Y	Y	Y	Y	Y	Y	Y	Y	Y	Y	Y														
11819	A_23_P102122	ARPC2	Y		Y				Y	Y					Y													Y			Y						Y									
17490	A_23_P29566	ARPC4			Y	Y		Y	Y	Y										Y									Y																	
8266	A_23_P126803	ARPC5			Y	Y		Y		Y													Y					Y																		
17810	A_23_P135205	ARPC5L																											Y		Y						Y									
9315	A_23_P205768	ARPP-19																					Y					Y																		
19099	A_23_P158829	ARRB2			Y	Y	Y	Y	Y	Y	Y	Y	Y	Y				Y	Y	Y	Y	Y	Y	Y	Y	Y	Y				Y															
7877	A_23_P332312	ARRDC1			Y				Y	Y								Y	Y	Y		Y	Y	Y				Y																		
15101	A_23_P130965	ARRDC2			Y	Y		Y		Y									Y	Y								Y																		
5536	A_23_P423942	ARS2			Y																																									
8808	A_23_P255436	ARSA			Y	Y	Y	Y		Y	Y	Y								Y	Y	Y	Y					Y	Y	Y	Y															
9651	A_23_P58783	ARSB				Y														Y			Y																							
17912	A_23_P256085	ARSE	Y	Y	Y	Y		Y	Y	Y									Y	Y			Y					Y	Y	Y	Y						Y									
15386	A_23_P304304	ARSF			Y				Y	Y																		Y			Y						Y									
5127	A_23_P4161	ARSG	Y	Y	Y	Y		Y	Y	Y	Y	Y	Y	Y				Y		Y		Y	Y					Y	Y	Y	Y						Y									
7324	A_23_P213296	ARSJ																		Y																										
20492	A_23_P213539	ARSK																										Y																		
22331	A_23_P87363	ART1				Y			Y	Y			Y						Y	Y									Y		Y						Y									
4711	A_23_P80918	ART3							Y	Y	Y	Y	Y	Y																	Y						Y									
998	A_23_P427119	ART5																		Y			Y																							
16942	A_23_P75963	ART5			Y	Y		Y	Y	Y									Y	Y		Y	Y					Y																		
17473	A_23_P135995	ARV1			Y													Y	Y	Y	Y		Y	Y	Y			Y																		
5921	A_23_P109393	ARVCF			Y													Y	Y	Y		Y	Y	Y																						
21718	A_23_P12643	AS3MT	Y	Y	Y				Y	Y	Y	Y	Y	Y									Y					Y				Y		Y	Y	Y										
3463	A_23_P216325	ASAH1							Y	Y																																				
20051	A_23_P161171	ASAH2			Y	Y		Y	Y	Y																		Y																		
3255	A_23_P35995	ASAM			Y													Y	Y	Y				Y				Y																		
4927	A_23_P44833	ASAP1							Y	Y																																				
17090	A_23_P216278	ASAP1				Y	Y		Y	Y	Y	Y																	Y		Y						Y						Y				
12804	A_23_P114689	ASAP3																			Y																									
7729	A_23_P165365	ASB1			Y	Y		Y	Y	Y	Y	Y	Y	Y					Y	Y									Y		Y						Y									
11500	A_23_P427634	ASB10			Y																							Y	Y	Y	Y						Y									
13448	A_23_P250141	ASB10			Y	Y		Y	Y	Y																			Y		Y						Y									
2078	A_23_P202510	ASB13																Y																												
8423	A_23_P155268	ASB14			Y																																									
5464	A_23_P82625	ASB15			Y																		Y								Y						Y									
14643	A_23_P118581	ASB16			Y	Y		Y	Y	Y																					Y						Y									
19038	A_23_P56827	ASB3																Y	Y	Y	Y	Y	Y	Y	Y	Y	Y				Y	Y	Y				Y									
1339	A_23_P157205	ASB4																													Y						Y									

922	A_23_P144326	ASB5																		Y																									
18127	A_23_P158059	ASB6			Y	Y	Y	Y		Y	Y	Y							Y	Y			Y				Y	Y	Y	Y						Y										
12956	A_23_P117744	ASB7			Y				Y	Y	Y	Y	Y	Y				Y									Y			Y			Y			Y										
7834	A_23_P22200	ASB8																					Y																							
5022	A_23_P125643	ASB9																			Y						Y																			
16830	A_23_P143636	ASCC2			Y	Y	Y	Y		Y	Y	Y						Y	Y	Y	Y	Y	Y	Y	Y	Y	Y				Y															
2379	A_23_P19565	ASCC3				Y																					Y																			
14039	A_23_P25305	ASCL1			Y				Y	Y																	Y																			
16584	A_23_P105088	ASCL2																	Y	Y			Y																							
14777	A_23_P2083	ASCL3																Y	Y																											
8130	A_23_P19219	ASF1A	Y	Y																																										
15126	A_23_P119254	ASF1B			Y	Y		Y		Y	Y	Y	Y	Y				Y	Y	Y	Y	Y	Y	Y	Y	Y	Y				Y															
15523	A_23_P118722	ASGR1				Y														Y	Y		Y																							
22246	A_23_P130116	ASGR2																					Y																							
11721	A_23_P63083	ASH1L			Y						Y		Y	Y													Y			Y						Y										
20820	A_23_P120629	ASIP			Y	Y		Y	Y	Y													Y				Y	Y	Y	Y						Y										
11123	A_23_P26223	ASL			Y															Y		Y					Y																			
14147	A_23_P255534	ASMT																		Y																										
6809	A_23_P27627	ASNA1			Y	Y	Y	Y	Y	Y	Y	Y	Y	Y				Y	Y	Y	Y	Y		Y			Y																			
9933	A_23_P145694	ASNS			Y																						Y																			
18833	A_23_P91001	ASNSD1																		Y																										
21149	A_23_P170667	ASPHD1																				Y																								
944	A_23_P91640	ASPHD2	Y		Y	Y	Y	Y		Y	Y	Y	Y	Y				Y	Y	Y	Y		Y	Y	Y		Y																			
2168	A_23_P216429	ASPN													Y																															
11834	A_23_P417383	ASPRV1			Y	Y		Y		Y										Y		Y																								
19102	A_23_P32320	ASPSCR1			Y	Y		Y		Y								Y	Y	Y		Y	Y	Y			Y																			
22036	A_23_P203391	ASRGL1				Y																																								
14724	A_23_P31922	ASS1			Y	Y		Y		Y																																				
11902	A_23_P121137	ASTE1			Y	Y		Y	Y	Y										Y		Y	Y				Y	Y	Y	Y						Y										
2102	A_23_P102439	ASTL			Y	Y	Y	Y	Y	Y	Y	Y	Y	Y					Y	Y							Y	Y	Y	Y						Y								Y		
8822	A_23_P357936	ASTN2	Y	Y	Y	Y		Y	Y	Y	Y	Y	Y	Y													Y	Y	Y	Y						Y										
11056	A_23_P366965	ASXL1			Y	Y		Y	Y	Y								Y	Y	Y				Y			Y	Y	Y	Y						Y										
21749	A_23_P210300	ASXL2			Y	Y		Y	Y	Y																		Y																		
9188	A_23_P75288	ATAD1			Y	Y	Y	Y	Y	Y	Y	Y								Y							Y	Y	Y	Y						Y							Y			
16745	A_23_P201357	ATAD3A			Y	Y	Y	Y	Y	Y	Y	Y						Y	Y	Y		Y	Y	Y			Y	Y	Y	Y						Y										
13145	A_23_P386346	ATAD3B			Y	Y	Y	Y	Y	Y	Y	Y						Y	Y	Y		Y	Y	Y			Y	Y	Y	Y						Y										
15520	A_23_P74697	ATAD3B			Y	Y	Y	Y	Y	Y	Y	Y						Y	Y	Y		Y	Y	Y			Y	Y	Y	Y						Y										
3150	A_23_P118894	ATAD4							Y	Y																																				
3112	A_23_P255750	ATAD5			Y	Y		Y		Y																		Y		Y						Y										
4838	A_23_P101673	ATCAY																		Y			Y																							
12395	A_23_P433063	ATCAY																					Y																							
20806	A_23_P138574	ATE1			Y															Y		Y	Y																							
20383	A_23_P112950	ATF7IP	Y	Y					Y	Y	Y	Y	Y	Y																																
21705	A_23_P48278	ATF7IP				Y																																								
6904	A_23_P129466	ATF7IP2			Y													Y		Y		Y	Y																							
8416	A_23_P253256	ATG10	Y	Y	Y		Y		Y	Y	Y	Y	Y	Y				Y	Y	Y	Y		Y	Y	Y		Y			Y	Y	Y	Y	Y	Y	Y										
731	A_23_P210266	ATG16L1							Y	Y	Y	Y	Y	Y																Y						Y										
14786	A_23_P36305	ATG16L2			Y	Y		Y	Y	Y																																				
1806	A_23_P88163	ATG2B			Y	Y	Y	Y	Y	Y	Y	Y	Y	Y				Y	Y	Y	Y	Y	Y	Y	Y	Y	Y	Y	Y	Y	Y	Y				Y										
4417	A_23_P9903	ATG4B			Y													Y		Y							Y																			
7613	A_23_P254	ATG4C																		Y								Y																		
1761	A_23_P101342	ATG4D			Y	Y		Y		Y								Y	Y	Y		Y	Y	Y			Y																			
2930	A_23_P111381	ATG5	Y	Y																																										
11587	A_23_P143992	ATG7	Y		Y	Y		Y		Y																	Y																			
8010	A_23_P165450	ATG9A			Y	Y		Y	Y	Y			Y					Y	Y	Y		Y	Y	Y			Y																			
10145	A_23_P88351	ATL1																	Y			Y																								
10056	A_23_P209619	ATL2							Y	Y										Y		Y	Y																							
21788	A_23_P105028	ATL3			Y						Y		Y	Y													Y																			
15347	A_23_P35916	ATM			Y	Y		Y	Y	Y								Y	Y	Y		Y	Y	Y						Y						Y										
18685	A_23_P100133	ATMIN			Y													Y	Y	Y	Y		Y	Y	Y		Y																			
3710	A_23_P13885	ATN1																		Y																										
10289	A_23_P332246	ATOH1																									Y																			
12231	A_23_P378514	ATOH7			Y	Y		Y		Y																	Y																			
9687	A_23_P142878	ATOH8																Y	Y	Y		Y	Y	Y																						
5164	A_23_P144877	ATOX1							Y	Y																																				
6607	A_23_P65659	ATP10A			Y	Y		Y		Y										Y			Y																							
6541	A_23_P213825	ATP10B																					Y																							
14776	A_23_P251730	ATP11C			Y	Y		Y	Y	Y										Y								Y		Y						Y										
1629	A_23_P87982	ATP12A			Y	Y		Y		Y																	Y	Y	Y	Y																
6735	A_23_P5165	ATP13A1			Y	Y	Y	Y	Y	Y	Y	Y	Y	Y				Y	Y	Y	Y	Y	Y	Y	Y	Y	Y				Y											Y				
3227	A_23_P12423	ATP13A2																				Y	Y																							
817	A_23_P256405	ATP13A4			Y	Y		Y	Y	Y	Y	Y	Y	Y													Y	Y	Y	Y						Y										
8333	A_23_P29394	ATP13A4																												Y						Y										
13116	A_23_P1075	ATP1A1																Y	Y	Y				Y																						
8687	A_23_P148879	ATP1A2			Y	Y		Y		Y																				Y						Y										
8336	A_23_P27472	ATP1A3			Y	Y		Y		Y																																				
20483	A_23_P160177	ATP1A4			Y	Y		Y		Y																																				

8540	A_23_P62932	ATP1B1	Y	Y	Y														Y	Y	Y	Y					Y			Y						Y										
11098	A_23_P146943	ATP1B1	Y	Y	Y	Y		Y		Y	Y	Y	Y	Y				Y	Y	Y	Y	Y		Y			Y			Y						Y						Y				
8591	A_23_P305086	ATP1B2				Y			Y	Y																	Y	Y	Y	Y						Y										
9754	A_23_P68007	ATP1B3							Y	Y																																				
12469	A_23_P6951	ATP1B3																												Y						Y										
149	A_23_P217432	ATP1B4			Y	Y		Y	Y	Y													Y				Y	Y	Y	Y						Y										
13076	A_23_P83858	ATP2A1			Y	Y	Y	Y	Y	Y	Y	Y							Y	Y			Y				Y																			
17655	A_23_P53603	ATP2A2			Y																																									
1190	A_23_P207639	ATP2A3							Y	Y										Y			Y																							
11982	A_23_P128319	ATP2B1																									Y	Y	Y	Y																
12309	A_23_P18153	ATP2B2																									Y																			
2726	A_23_P22723	ATP2B3																										Y																		
7962	A_23_P337642	ATP2B3	Y																																											
13967	A_23_P11841	ATP2B4																									Y																			
1066	A_23_P143916	ATP2C1																					Y																							
20926	A_23_P117992	ATP2C2				Y			Y	Y																																				
14683	A_23_P119642	ATP4A				Y																Y																								
7897	A_23_P78423	ATP5A1																		Y			Y																							
2906	A_23_P33216	ATP5B			Y															Y		Y																								
8935	A_23_P63649	ATP5C1			Y																																									
2226	A_23_P5089	ATP5D																										Y																		
4031	A_23_P252322	ATP5E				Y																																								
1754	A_23_P46275	ATP5F1																Y		Y																										
1416	A_23_P87616	ATP5G2			Y				Y	Y	Y	Y	Y	Y													Y			Y						Y										
20466	A_23_P56678	ATP5G3			Y	Y		Y	Y	Y									Y									Y		Y						Y										
21216	A_23_P77818	ATP5H	Y	Y																																										
10966	A_23_P154832	ATP5J			Y																																									
7977	A_23_P215835	ATP5J2			Y	Y		Y		Y																	Y																			
16041	A_23_P107795	ATP5SL			Y				Y	Y																				Y						Y										
18159	A_23_P250462	ATP6AP1																										Y																		
1487	A_23_P11353	ATP6AP2			Y																		Y				Y																			
4115	A_23_P49735	ATP6V0A1			Y	Y		Y	Y	Y								Y	Y	Y		Y	Y	Y			Y			Y						Y										
12462	A_23_P137814	ATP6V0B			Y	Y	Y	Y	Y	Y	Y	Y	Y	Y				Y	Y	Y		Y	Y	Y			Y	Y	Y	Y						Y										
12539	A_23_P54636	ATP6V0D1			Y	Y	Y	Y	Y	Y	Y	Y							Y	Y	Y	Y	Y				Y																			
5365	A_23_P213840	ATP6V0E1				Y																																								
15482	A_23_P211965	ATP6V1A																			Y																									
6654	A_23_P31844	ATP6V1B2			Y	Y	Y	Y		Y	Y	Y								Y			Y				Y																			
1274	A_23_P250914	ATP6V1C2			Y	Y	Y	Y	Y	Y	Y	Y						Y	Y	Y		Y		Y			Y	Y	Y	Y						Y										
9225	A_23_P140115	ATP6V1D			Y				Y	Y								Y		Y			Y				Y																			
19176	A_23_P143545	ATP6V1E1			Y																																									
12615	A_23_P143044	ATP6V1E2																										Y																		
14731	A_23_P93623	ATP6V1F			Y	Y	Y	Y		Y	Y	Y	Y	Y						Y							Y																			
15677	A_23_P20882	ATP6V1G1																		Y																										
20325	A_23_P217731	ATP7A				Y												Y	Y	Y		Y	Y	Y				Y																		
6289	A_23_P205228	ATP7B			Y	Y	Y	Y	Y	Y	Y	Y	Y	Y				Y		Y	Y						Y	Y	Y	Y						Y										
3341	A_23_P30075	ATP8A1			Y				Y	Y								Y		Y			Y				Y			Y						Y										
22405	A_23_P79108	ATP8B3			Y														Y																											
2639	A_23_P102664	ATP9A			Y													Y	Y	Y	Y	Y		Y			Y			Y						Y										
20606	A_23_P10497	ATP9B																		Y																										
8912	A_23_P303320	ATPBD3			Y	Y		Y		Y								Y	Y	Y		Y	Y	Y				Y																		
13242	A_23_P77133	ATPBD4			Y														Y	Y							Y																			
14241	A_23_P126716	ATPIF1			Y	Y		Y	Y	Y	Y	Y	Y	Y				Y	Y	Y		Y	Y	Y			Y	Y	Y	Y						Y										
13797	A_23_P136054	ATR			Y	Y	Y	Y		Y	Y	Y						Y	Y	Y	Y	Y	Y	Y	Y	Y	Y				Y															
15640	A_23_P124664	ATR			Y	Y	Y	Y		Y	Y	Y								Y	Y	Y	Y				Y																			
13920	A_23_P136874	ATRX			Y	Y		Y	Y	Y								Y	Y	Y		Y	Y	Y			Y	Y	Y	Y						Y										
8331	A_23_P17927	ATXN10			Y																		Y																							
4497	A_23_P163814	ATXN1L			Y																						Y																			
1675	A_23_P48141	ATXN2			Y																																									
17738	A_23_P389907	ATXN2			Y	Y		Y	Y	Y	Y	Y	Y	Y				Y	Y	Y		Y		Y			Y	Y	Y	Y						Y										
157	A_23_P136044	ATXN2L																		Y		Y	Y				Y																			
5430	A_23_P257155	ATXN7				Y																						Y																		
825	A_23_P356109	ATXN7L1																												Y						Y										
261	A_23_P20852	AUH			Y	Y		Y		Y									Y	Y			Y				Y																			
5783	A_23_P253421	AUP1			Y	Y	Y	Y	Y	Y	Y	Y						Y	Y	Y	Y	Y	Y	Y	Y	Y	Y	Y	Y	Y	Y	Y				Y										
8500	A_23_P131866	AURKA	Y	Y		Y												Y		Y		Y						Y		Y						Y										
19659	A_23_P130182	AURKB							Y	Y									Y	Y		Y																								
6612	A_23_P50226	AURKC																Y																												
4786	A_23_P122906	AUTS2			Y	Y	Y	Y	Y	Y	Y	Y						Y		Y	Y	Y	Y				Y	Y	Y	Y																
17284	A_23_P100074	AVEN	Y																																											
15817	A_23_P390157	AVIL																	Y	Y							Y																			
4901	A_23_P109133	AVP			Y	Y		Y		Y										Y		Y	Y				Y																			
1279	A_23_P72340	AVPR1B			Y	Y		Y		Y									Y								Y	Y	Y	Y																
20708	A_23_P96583	AVPR2			Y	Y		Y		Y									Y	Y		Y					Y																			
18810	A_23_P159395	AXIN2																									Y	Y	Y	Y						Y										
19031	A_23_P148014	AXIN2																									Y																			
150	A_23_P208389	AXL																		Y																										
1319	A_23_P121010	AXUD1			Y	Y		Y		Y										Y		Y																								

12645	A_23_P71270	AZGP1							Y	Y	Y	Y	Y	Y				Y					Y					Y		Y			Y			Y										
19359	A_23_P109682	AZI2				Y														Y		Y	Y							Y						Y										
18224	A_23_P108564	B3GALT1																			Y																									
21851	A_23_P499	B3GALT2			Y																																									
13103	A_23_P35021	B3GALT6			Y	Y		Y	Y	Y								Y	Y	Y		Y		Y			Y	Y	Y	Y																
14013	A_23_P1833	B3GAT1																									Y																			
4686	A_23_P36142	B3GAT3			Y	Y		Y	Y	Y									Y	Y		Y	Y				Y	Y	Y	Y																
20660	A_23_P86899	B3GNT1	Y	Y	Y	Y		Y	Y	Y								Y	Y	Y		Y	Y	Y			Y	Y	Y	Y						Y										
17823	A_23_P500950	B3GNT2			Y																																									
18488	A_23_P78980	B3GNT3			Y	Y	Y	Y	Y	Y	Y	Y															Y	Y	Y	Y						Y										
1149	A_23_P76071	B3GNT4			Y		Y				Y							Y	Y			Y					Y			Y						Y										
11159	A_23_P18372	B3GNT5			Y				Y	Y	Y	Y	Y	Y																Y						Y										
11126	A_23_P366895	B3GNT6			Y				Y	Y																	Y			Y						Y										
19490	A_23_P127978	B3GNT6	Y		Y				Y	Y								Y	Y	Y				Y			Y																			
15228	A_23_P39755	B3GNT7			Y	Y		Y	Y	Y																	Y	Y	Y	Y																
9835	A_23_P66051	B3GNT9			Y	Y		Y		Y								Y	Y	Y		Y	Y	Y			Y																			
2268	A_23_P87461	B4GALNT1			Y	Y	Y	Y	Y	Y	Y	Y	Y	Y				Y	Y	Y		Y	Y	Y			Y	Y	Y	Y						Y										
4288	A_23_P331211	B4GALNT2			Y	Y		Y	Y	Y								Y	Y	Y		Y	Y	Y			Y	Y	Y	Y						Y										
10300	A_23_P149206	B4GALT2			Y				Y	Y								Y	Y	Y		Y	Y	Y			Y			Y						Y										
1870	A_23_P41872	B4GALT7			Y	Y	Y	Y	Y	Y	Y	Y						Y	Y	Y	Y	Y	Y	Y	Y	Y	Y			Y	Y	Y				Y										
10072	A_23_P152678	B9D1																															Y													
9526	A_23_P134663	BAALC				Y															Y																									
13272	A_23_P71810	BAAT																Y																												
22386	A_23_P52806	BACE1																	Y																											
7615	A_23_P154876	BACE2				Y																																								
5848	A_23_P150207	BAD			Y																						Y																			
12980	A_23_P146654	BAG1	Y	Y	Y	Y		Y	Y	Y										Y		Y	Y				Y	Y	Y	Y			Y			Y										
7695	A_23_P356554	BAG2							Y	Y																		Y		Y						Y										
18660	A_23_P255363	BAG2							Y	Y																		Y		Y						Y										
14980	A_23_P47077	BAG3																	Y	Y		Y																								
9476	A_23_P76712	BAGE							Y	Y			Y																	Y						Y										
14945	A_23_P388331	BAGE																		Y																										
9653	A_23_P49539	BAHCC1				Y			Y	Y																		Y		Y						Y										
1456	A_23_P117928	BAHD1																					Y																							
6321	A_23_P149019	BAI2	Y	Y	Y	Y		Y	Y	Y																	Y	Y	Y	Y						Y										
11199	A_23_P252556	BAI3			Y																																									
1970	A_23_P61810	BAIAP2							Y	Y																																				
14521	A_23_P33543	BAIAP2																	Y			Y																								
16203	A_23_P315836	BAIAP2				Y			Y	Y	Y	Y	Y	Y													Y	Y	Y	Y						Y										
18490	A_23_P251142	BAIAP2																	Y																											
4286	A_23_P168470	BAIAP2L1			Y	Y		Y	Y	Y								Y	Y	Y		Y		Y			Y																			
5224	A_23_P379039	BAIAP2L2			Y	Y		Y		Y									Y	Y							Y	Y	Y	Y																
13243	A_23_P163492	BAIAP3																												Y						Y										
18924	A_23_P145357	BAK1			Y	Y	Y	Y	Y	Y	Y	Y	Y	Y					Y	Y	Y	Y	Y				Y	Y	Y	Y						Y										
6671	A_23_P47210	BANF1			Y													Y		Y	Y		Y				Y																			
21580	A_23_P10232	BANK1	Y												Y					Y							Y																			
4856	A_23_P10414	BANP																		Y	Y																									
13092	A_23_P22263	BANP																		Y	Y							Y																		
14644	A_23_P251765	BANP			Y	Y		Y	Y	Y										Y		Y					Y																			
20478	A_23_P21819	BANP																		Y	Y							Y																		
20524	A_23_P259012	BAP1			Y	Y	Y	Y	Y	Y	Y	Y						Y	Y	Y	Y	Y	Y	Y	Y	Y	Y				Y															
19724	A_23_P9333	BARHL1																										Y																		
18791	A_23_P32277	BARX1				Y			Y	Y										Y		Y						Y																		
22287	A_23_P213386	BASP1																		Y																										
18902	A_23_P412409	BAT2D1			Y	Y		Y		Y									Y	Y																										
2292	A_23_P111141	BAT3			Y	Y	Y	Y	Y	Y	Y	Y	Y	Y				Y	Y	Y		Y	Y	Y			Y	Y	Y	Y						Y										
4862	A_23_P156696	BAT5			Y	Y		Y		Y									Y	Y		Y	Y																							
C13825	A_23_P76799	BAZ1A																		Y																										
19296	A_23_P215449	BAZ1B																	Y	Y	Y		Y				Y																			
5910	A_23_P120048	BAZ2B			Y	Y		Y	Y	Y	Y	Y	Y	Y					Y	Y		Y					Y	Y	Y	Y						Y										
10190	A_23_P382775	BBC3			Y	Y		Y	Y	Y									Y								Y			Y						Y										
15097	A_23_P90193	BBC3			Y				Y	Y	Y	Y	Y	Y														Y		Y						Y										
16676	A_23_P127495	BBOX1			Y	Y		Y	Y	Y	Y	Y	Y	Y					Y	Y							Y			Y						Y										
15270	A_23_P150267	BBS1	Y	Y	Y	Y	Y	Y	Y	Y	Y	Y	Y	Y				Y	Y	Y		Y	Y	Y			Y	Y	Y	Y						Y						Y				
15510	A_23_P95612	BBS10			Y	Y		Y		Y										Y							Y			Y						Y										
1973	A_23_P349481	BBS12			Y													Y	Y	Y				Y			Y																			
20948	A_23_P106835	BBS2	Y	Y	Y	Y	Y	Y	Y	Y	Y	Y	Y	Y				Y	Y	Y	Y	Y		Y			Y	Y	Y	Y						Y										
5015	A_23_P99961	BBS4			Y	Y	Y	Y	Y	Y	Y	Y	Y	Y				Y		Y	Y	Y	Y				Y	Y	Y	Y						Y								Y		
17054	A_23_P5785	BBS5																					Y																							
19673	A_23_P252913	BBS7							Y	Y										Y			Y																							
5217	A_23_P82356	BBS9				Y																						Y																		
8367	A_23_P121356	BBX			Y	Y		Y	Y	Y								Y		Y							Y	Y	Y	Y																
3690	A_23_P55716	BCAM			Y															Y																										
4865	A_23_P398574	BCAM																		Y			Y																							
22089	A_23_P160607	BCAN			Y	Y		Y		Y																	Y																			
15888	A_23_P412526	BCAP29																		Y																										
18356	A_23_P168782	BCAP29																		Y																										

13888	A_23_P45508	BCAP31			Y	Y		Y	Y	Y			Y					Y	Y	Y	Y	Y	Y	Y	Y	Y	Y				Y															
2259	A_23_P66319	BCAR1			Y															Y			Y																							
20195	A_23_P97394	BCAR3			Y	Y		Y		Y								Y		Y		Y					Y			Y						Y										
6305	A_23_P17420	BCAS1							Y	Y	Y	Y	Y	Y						Y										Y						Y						Y				
6522	A_23_P153068	BCAS3																		Y		Y	Y																							
9253	A_23_P40209	BCAS4			Y																						Y																			
17211	A_23_P87528	BCAT1			Y														Y																											
3404	A_23_P101551	BCAT2																			Y																									
4879	A_23_P202474	BCCIP				Y			Y	Y										Y								Y																		
10515	A_23_P338233	BCDIN3D			Y				Y	Y																	Y																			
5406	A_23_P212050	BCHE			Y																																									
5811	A_23_P67188	BCKDHA																	Y	Y		Y																								
3655	A_23_P3823	BCKDK																	Y	Y		Y	Y																							
20736	A_23_P365149	BCKDK																		Y	Y	Y																								
13520	A_23_P218584	BCL11A							Y	Y																																				
10911	A_23_P352266	BCL2	Y	Y	Y	Y		Y	Y	Y	Y	Y	Y	Y				Y	Y	Y		Y	Y	Y			Y	Y	Y	Y						Y										
14297	A_23_P208132	BCL2			Y	Y		Y	Y	Y									Y	Y			Y				Y																			
20687	A_23_P151995	BCL2A1	Y	Y	Y						Y		Y	Y																Y						Y										
13181	A_23_P210886	BCL2L1			Y															Y	Y	Y																								
8482	A_23_P163209	BCL2L10																	Y	Y			Y																							
1166	A_23_P154426	BCL2L11																Y																												
5391	A_23_P60693	BCL2L11							Y	Y																																				
22242	A_23_P50477	BCL2L12			Y	Y	Y	Y	Y	Y	Y	Y	Y	Y				Y	Y	Y		Y		Y			Y	Y	Y	Y						Y										
15898	A_23_P102965	BCL2L13			Y													Y		Y		Y					Y																			
13210	A_23_P128050	BCL2L14																	Y								Y																			
19578	A_23_P140219	BCL2L2				Y														Y		Y	Y					Y																		
17525	A_23_P4662	BCL3							Y	Y																																				
10544	A_23_P57856	BCL6	Y	Y	Y	Y	Y	Y		Y	Y	Y						Y	Y	Y		Y	Y	Y			Y			Y	Y	Y	Y	Y	Y	Y										
22222	A_23_P130130	BCL6B																									Y	Y	Y	Y																
1827	A_23_P61831	BCL7A			Y																						Y																			
10260	A_23_P400814	BCL7A																												Y						Y										
19869	A_23_P123007	BCL7B																Y	Y		Y																									
19096	A_23_P118283	BCL7C			Y	Y		Y		Y								Y	Y	Y		Y	Y	Y			Y			Y						Y										
11090	A_23_P200273	BCL9			Y	Y		Y	Y	Y																																				
12999	A_23_P116183	BCO2	Y		Y	Y	Y	Y	Y	Y	Y	Y						Y	Y	Y		Y	Y	Y			Y	Y	Y	Y						Y										
17061	A_23_P159741	BCOR	Y	Y	Y	Y	Y	Y	Y	Y	Y	Y	Y	Y													Y	Y	Y	Y						Y										
8792	A_23_P211377	BCR			Y													Y		Y			Y				Y	Y	Y	Y						Y										
20252	A_23_P503113	BCR			Y													Y		Y		Y	Y				Y																			
17971	A_23_P29754	BDH1																									Y																			
1957	A_23_P92490	BDH2			Y	Y		Y	Y	Y								Y	Y	Y	Y	Y	Y	Y	Y	Y	Y	Y	Y	Y	Y	Y				Y										
5919	A_23_P304897	BDKRB2			Y	Y		Y		Y																	Y																			
20010	A_23_P140142	BDKRB2																										Y																		
13553	A_23_P433071	BECN1			Y	Y		Y	Y	Y										Y			Y				Y			Y						Y										
2590	A_23_P72127	BEGAIN	Y	Y	Y				Y	Y					Y												Y																			
21133	A_23_P306173	BEND5							Y	Y								Y		Y		Y	Y							Y						Y										
16484	A_23_P333658	BEND6																	Y	Y																										
22265	A_23_P312358	BEND7							Y	Y													Y																							
378	A_23_P104992	BEST1																									Y																			
8918	A_23_P16225	BEST2			Y																																									
3359	A_23_P323801	BEST3																										Y																		
13125	A_23_P59701	BET1			Y	Y		Y		Y																																				
14125	A_23_P116482	BET1L			Y	Y	Y	Y	Y	Y	Y	Y								Y		Y	Y				Y	Y	Y	Y						Y										
17310	A_23_P127879	BET1L																			Y																									
18733	A_23_P159952	BEX1			Y	Y	Y	Y	Y	Y	Y	Y	Y	Y													Y																			
7341	A_23_P22735	BEX2			Y																		Y				Y																			
21125	A_23_P114391	BEX4			Y													Y	Y	Y		Y	Y	Y						Y						Y										
10231	A_23_P65963	BFAR																Y	Y	Y		Y		Y																						
12054	A_23_P159316	BFSP2							Y	Y																																				
9182	A_23_P34126	BGN																Y		Y			Y																							
10745	A_23_P331177	BHLHA15			Y	Y	Y	Y	Y	Y	Y	Y	Y	Y						Y		Y	Y				Y	Y	Y	Y						Y								Y		
18698	A_23_P402194	BHLHE22			Y																						Y																			
16167	A_23_P139500	BHLHE41				Y																																								
12011	A_23_P81581	BHMT			Y																																									
10335	A_23_P92983	BHMT2																												Y						Y										
6009	A_23_P86532	BICC1				Y																						Y																		
1208	A_23_P157751	BICD2			Y	Y		Y		Y								Y		Y		Y																								
13665	A_23_P407551	BICD2				Y			Y	Y										Y		Y																								
11046	A_23_P154929	BID																	Y																											
6766	A_23_P404667	BIK							Y	Y	Y	Y	Y	Y																																
16507	A_23_P103163	BIK							Y	Y																																				
17216	A_23_P165333	BIN1			Y	Y		Y	Y	Y	Y	Y	Y	Y						Y	Y	Y	Y				Y	Y	Y	Y																
3940	A_23_P162392	BIN2	Y		Y	Y	Y	Y	Y	Y	Y	Y	Y	Y	Y			Y	Y	Y			Y	Y			Y				Y		Y	Y	Y											
5218	A_23_P86959	BIRC2				Y													Y	Y		Y	Y																							
5024	A_23_P118815	BIRC5	Y	Y	Y	Y		Y	Y	Y								Y		Y							Y	Y	Y	Y						Y										
859	A_23_P142614	BIRC6				Y														Y																										
7485	A_23_P105833	BIVM							Y	Y	Y	Y	Y	Y																Y						Y							Y			
13717	A_23_P102591	BLCAP			Y													Y	Y	Y	Y	Y		Y			Y																			

14598	A_23_P419621	BLCAP			Y																									Y						Y										
15034	A_23_P31725	BLK			Y	Y	Y	Y		Y	Y	Y							Y	Y			Y				Y	Y	Y	Y																
3286	A_23_P88630	BLM																Y																												
19235	A_23_P141606	BLMH			Y	Y	Y	Y	Y	Y	Y	Y	Y	Y													Y	Y	Y	Y						Y										
19689	A_23_P310911	BLMH			Y	Y		Y	Y	Y	Y	Y	Y	Y													Y	Y	Y	Y																
3827	A_23_P52442	BLNK																			Y																									
1220	A_23_P71148	BLVRA							Y	Y	Y	Y	Y	Y																																
1256	A_23_P153351	BLVRB			Y				Y	Y									Y	Y																										
13015	A_23_P23266	BLZF1				Y													Y	Y	Y	Y	Y																							
7083	A_23_P379645	BMF																		Y			Y				Y																			
17758	A_23_P3478	BMF																Y										Y																		
7997	A_23_P115732	BMI1			Y	Y	Y	Y		Y	Y	Y								Y		Y	Y				Y	Y	Y	Y						Y										
19259	A_23_P314115	BMI1			Y	Y	Y	Y	Y	Y	Y	Y								Y		Y	Y				Y	Y	Y	Y						Y										
16439	A_23_P33277	BMP1			Y																	Y																								
16381	A_23_P11107	BMP15			Y														Y			Y																								
8665	A_23_P54140	BMP4																												Y						Y										
11569	A_23_P154643	BMP7																			Y		Y																							
16459	A_23_P68488	BMP7			Y													Y				Y	Y				Y																			
3616	A_23_P85922	BMP8A			Y																		Y				Y																			
14746	A_23_P126508	BMP8B			Y	Y		Y		Y								Y		Y	Y	Y	Y				Y	Y	Y	Y																
20470	A_23_P1431	BMPR1A																	Y	Y		Y																								
21400	A_23_P58223	BMPR1B	Y	Y	Y	Y		Y		Y									Y				Y				Y	Y	Y	Y						Y										
6960	A_23_P24068	BMS1																		Y	Y		Y																							
18654	A_23_P109420	BMS1	Y	Y	Y	Y		Y	Y	Y	Y	Y	Y	Y																Y						Y										
13309	A_23_P149936	BMS1P5			Y	Y	Y	Y	Y	Y	Y	Y	Y	Y				Y		Y		Y	Y				Y	Y	Y	Y						Y										
572	A_23_P22134	BNC1																										Y																		
15343	A_23_P7655	BNIP1			Y																																									
11682	A_23_P134925	BNIP3L			Y		Y				Y									Y																										
11984	A_23_P408708	BNIP3L																	Y	Y		Y																								
629	A_23_P257762	BOC			Y				Y	Y									Y																											
19295	A_23_P253029	BOK			Y	Y		Y	Y	Y												Y					Y																			
12212	A_23_P102084	BOLL							Y	Y																				Y						Y										
15717	A_23_P43800	BOP1			Y	Y	Y	Y	Y	Y	Y	Y	Y	Y				Y	Y	Y	Y	Y	Y	Y	Y	Y	Y	Y	Y	Y	Y	Y				Y										
13550	A_23_P254558	BPESC1																Y	Y	Y		Y		Y																						
15872	A_23_P70843	BPGM																	Y			Y																								
4535	A_23_P131786	BPI			Y				Y	Y																																				
15083	A_23_P254797	BPIL1			Y	Y		Y	Y	Y									Y	Y			Y				Y	Y	Y	Y						Y										
21050	A_23_P408953	BPIL2			Y																																									
9217	A_23_P42935	BRAF			Y																Y																									
C11046.4	A_23_P207400	BRCA1			Y	Y		Y	Y	Y										Y																										
7969	A_23_P416561	BRCC3							Y	Y																				Y						Y										
12195	A_23_P217659	BRCC3			Y													Y		Y		Y	Y																							
16976	A_23_P216689	BRD3			Y													Y		Y	Y		Y				Y																			
4349	A_23_P425104	BRD4			Y				Y	Y								Y	Y	Y		Y	Y	Y			Y																			
21321	A_23_P209011	BRD4			Y													Y		Y		Y					Y																			
12826	A_23_P145016	BRD8	Y	Y	Y	Y	Y	Y	Y	Y	Y	Y	Y	Y					Y	Y		Y	Y				Y	Y	Y	Y	Y	Y	Y	Y	Y	Y				Y						
16504	A_23_P19004	BRD9			Y													Y	Y	Y		Y	Y	Y																						
8653	A_23_P117416	BRF1																	Y	Y			Y																							
17451	A_23_P122915	BRI3	Y		Y	Y		Y	Y	Y	Y	Y	Y	Y						Y								Y		Y						Y										
4703	A_23_P25313	BRI3BP																												Y						Y										
14259	A_23_P15843	BRIP1				Y																																								
16784	A_23_P75500	BRMS1			Y	Y		Y		Y																	Y																			
2918	A_23_P110961	BRP44L	Y		Y				Y	Y																																				
6572	A_23_P250948	BRPF1																Y	Y	Y		Y		Y																						
11727	A_23_P156764	BRPF3			Y				Y	Y																	Y	Y	Y	Y																
3438	A_23_P33961	BRS3			Y																						Y			Y						Y										
7789	A_23_P405744	BRSK1	Y	Y																																										
7452	A_23_P32514	BRSK2			Y	Y		Y		Y									Y	Y	Y	Y	Y				Y	Y	Y	Y																
5791	A_23_P73451	BRUNOL4							Y	Y																																				
20821	A_23_P250661	BRUNOL5			Y	Y		Y		Y								Y									Y																			
15265	A_23_P140487	BRUNOL6							Y	Y																																				
13350	A_23_P211136	BRWD1			Y	Y	Y	Y	Y	Y	Y	Y	Y	Y				Y	Y	Y	Y	Y	Y	Y	Y	Y	Y	Y	Y	Y	Y	Y	Y	Y	Y	Y										
11409	A_23_P63930	BRWD2			Y	Y		Y	Y	Y	Y	Y	Y	Y						Y							Y	Y	Y	Y						Y										
3365	A_23_P379155	BRWD3			Y																									Y						Y										
16877	A_23_P150566	BSCL2							Y	Y																				Y						Y										
20798	A_23_P11705	BSDC1				Y																																								
6692	A_23_P142227	BSG			Y	Y	Y	Y	Y	Y	Y	Y	Y	Y				Y		Y	Y		Y				Y	Y	Y	Y						Y										
5705	A_23_P29735	BSN			Y	Y		Y		Y																	Y	Y	Y	Y																
2501	A_23_P7325	BST1	Y	Y											Y																															
1554	A_23_P39465	BST2																		Y																										
12977	A_23_P24017	BTAF1			Y	Y		Y		Y																	Y	Y	Y	Y																
15416	A_23_P408675	BTAF1				Y																Y																								
2162	A_23_P205830	BTBD1			Y																		Y							Y						Y										
4850	A_23_P99349	BTBD11																	Y																											
22086	A_23_P419712	BTBD11																Y																												
20945	A_23_P371124	BTBD12			Y	Y		Y	Y	Y																	Y			Y						Y										
1259	A_23_P100613	BTBD17			Y	Y	Y	Y		Y	Y	Y								Y			Y				Y	Y	Y	Y						Y										

19624	A_23_P153698	BTBD2			Y	Y	Y	Y		Y	Y	Y	Y	Y				Y	Y	Y	Y	Y	Y	Y	Y	Y	Y				Y															
16530	A_23_P102759	BTBD3							Y	Y																																				
11887	A_23_P88179	BTBD7							Y	Y	Y	Y	Y	Y				Y	Y	Y		Y	Y	Y						Y						Y										
280	A_23_P155348	BTD																									Y																			
8677	A_23_P62901	BTG2			Y	Y		Y	Y	Y																	Y	Y	Y	Y																
14312	A_23_P80068	BTG3	Y	Y	Y				Y	Y								Y												Y						Y										
10020	A_23_P47322	BTG4			Y																						Y																			
4268	A_23_P137139	BTK			Y																																									
2360	A_23_P80886	BTLA	Y	Y	Y														Y								Y																			
11291	A_23_P7985	BTN1A1				Y													Y									Y																		
20766	A_23_P167993	BTN2A1			Y				Y	Y	Y	Y	Y	Y				Y	Y	Y	Y	Y	Y	Y	Y	Y					Y															
14122	A_23_P145218	BTN2A3			Y	Y		Y		Y										Y			Y				Y																			
3943	A_23_P391264	BTN3A2																					Y																							
15470	A_23_P156596	BTN3A3			Y																																									
21091	A_23_P7412	BTNL8			Y																						Y																			
21632	A_23_P81278	BTNL9				Y																						Y																		
10352	A_23_P46819	BTRC	Y	Y	Y	Y	Y	Y	Y	Y	Y	Y	Y	Y					Y	Y		Y	Y				Y	Y	Y	Y						Y										
14141	A_23_P35427	BTRC			Y													Y	Y	Y				Y			Y			Y						Y										
18429	A_23_P124417	BUB1	Y	Y					Y	Y								Y												Y						Y										
19468	A_23_P320658	BUB3				Y																						Y																		
8574	A_23_P110980	BVES																												Y						Y										
2069	A_23_P32426	BXDC5			Y	Y		Y	Y	Y										Y							Y	Y	Y	Y																
15877	A_23_P359616	BXDC5				Y														Y																										
2245	A_23_P145194	BYSL							Y	Y										Y										Y						Y										
20108	A_23_P165418	BZW1																		Y																										
2057	A_23_P157215	BZW2							Y	Y																																				
14337	A_23_P35595	C10orf10			Y	Y		Y	Y	Y								Y	Y	Y	Y	Y	Y	Y	Y	Y	Y	Y	Y	Y	Y	Y	Y	Y	Y	Y										
16688	A_23_P314778	C10orf107			Y													Y									Y																			
2120	A_23_P113034	C10orf11																									Y																			
610	A_23_P202594	C10orf119			Y	Y		Y		Y										Y							Y																			
12749	A_23_P97952	C10orf125	Y	Y	Y	Y	Y	Y	Y	Y	Y	Y	Y	Y					Y	Y			Y				Y	Y	Y	Y						Y										
3513	A_23_P24101	C10orf128																					Y																							
20528	A_23_P24244	C10orf18			Y				Y	Y										Y		Y	Y																							
1928	A_23_P138461	C10orf2																Y	Y																											
10454	A_23_P1248	C10orf26																					Y																							
14083	A_23_P301330	C10orf28			Y	Y		Y		Y																																				
19550	A_23_P305140	C10orf32			Y	Y	Y	Y	Y	Y	Y	Y								Y							Y	Y	Y	Y																
10687	A_23_P63880	C10orf4			Y	Y	Y	Y	Y	Y	Y	Y	Y	Y					Y	Y		Y					Y	Y	Y	Y						Y										
9972	A_23_P324390	C10orf49																		Y			Y																							
19047	A_23_P24077	C10orf54			Y	Y		Y		Y									Y	Y			Y				Y																			
19395	A_23_P97858	C10orf57							Y	Y																																				
18069	A_23_P63660	C10orf58							Y	Y								Y																												
4752	A_23_P202508	C10orf59			Y														Y	Y		Y					Y																			
3564	A_23_P97807	C10orf63							Y	Y																																				
15006	A_23_P125392	C10orf68			Y	Y		Y		Y									Y	Y		Y	Y																							
9246	A_23_P86504	C10orf76			Y				Y	Y																																				
3727	A_23_P413193	C10orf78			Y	Y		Y	Y	Y																	Y	Y	Y	Y																
12107	A_23_P333038	C10orf79			Y	Y	Y	Y	Y	Y	Y	Y	Y	Y				Y	Y	Y		Y	Y	Y			Y	Y	Y	Y						Y										
15288	A_23_P1286	C10orf82			Y	Y	Y	Y	Y	Y	Y	Y						Y		Y		Y					Y	Y	Y	Y						Y								Y		
8620	A_23_P115749	C10orf84			Y	Y	Y	Y	Y	Y	Y	Y						Y	Y	Y		Y	Y	Y			Y	Y	Y	Y						Y										
3781	A_23_P24269	C10orf88							Y	Y																																				
17574	A_23_P75179	C10orf92				Y												Y				Y						Y																		
19461	A_23_P150350	C11orf1																		Y	Y		Y																							
638	A_23_P47527	C11orf10			Y		Y		Y	Y	Y	Y	Y	Y					Y	Y		Y								Y						Y										
4986	A_23_P104641	C11orf2																		Y		Y	Y																							
22185	A_23_P202773	C11orf20			Y																									Y						Y										
12023	A_23_P98671	C11orf21	Y	Y	Y														Y																											
20294	A_23_P12911	C11orf24							Y	Y																																				
10945	A_23_P64119	C11orf41							Y	Y									Y											Y						Y										
10754	A_23_P64287	C11orf46			Y	Y		Y	Y	Y								Y	Y	Y		Y		Y			Y	Y	Y	Y						Y										
17724	A_23_P1944	C11orf48																	Y																											
10939	A_23_P13512	C11orf51																			Y																									
9993	A_23_P1722	C11orf52			Y																		Y																							
13157	A_23_P47559	C11orf58				Y			Y	Y									Y	Y		Y	Y				Y	Y	Y	Y						Y										
15489	A_23_P2114	C11orf59			Y	Y		Y	Y	Y									Y	Y	Y	Y	Y				Y			Y						Y										
808	A_23_P116202	C11orf60			Y				Y	Y	Y	Y	Y	Y				Y	Y		Y	Y	Y							Y						Y										
8047	A_23_P52885	C11orf61			Y	Y		Y	Y	Y								Y	Y	Y		Y	Y	Y			Y																			
6761	A_23_P83749	C11orf63			Y	Y	Y	Y		Y	Y	Y						Y		Y		Y					Y																			
7624	A_23_P418485	C11orf65																Y		Y							Y																			
12543	A_23_P150238	C11orf68			Y															Y		Y	Y																							
10306	A_23_P116161	C11orf70			Y				Y	Y								Y	Y	Y		Y		Y																						
12188	A_23_P13147	C11orf71																					Y																							
7781	A_23_P139396	C11orf73			Y				Y	Y	Y	Y	Y	Y																Y						Y										
16065	A_23_P325131	C11orf74			Y	Y		Y		Y									Y	Y																										
893	A_23_P75430	C11orf75	Y	Y	Y	Y		Y	Y	Y	Y	Y	Y	Y	Y												Y	Y	Y	Y						Y										
18284	A_23_P1926	C11orf79			Y																						Y																			
15664	A_23_P116797	C12orf10																		Y			Y																							

12901	A_23_P388923	C12orf12																												Y						Y										
3525	A_23_P410613	C12orf23			Y																						Y																			
12479	A_23_P65000	C12orf24							Y	Y	Y	Y	Y	Y																Y						Y										
19283	A_23_P400924	C12orf30			Y				Y	Y								Y		Y		Y																								
3190	A_23_P105583	C12orf32																Y	Y			Y																								
3301	A_23_P98930	C12orf35			Y	Y		Y	Y	Y								Y	Y	Y		Y		Y			Y	Y	Y	Y																
10165	A_23_P2412	C12orf39																Y	Y	Y		Y		Y																						
16503	A_23_P322083	C12orf39			Y																																									
3425	A_23_P105372	C12orf41																			Y																									
4491	A_23_P116834	C12orf44			Y	Y	Y	Y		Y	Y	Y						Y	Y	Y		Y	Y	Y			Y	Y	Y	Y																
17011	A_23_P128532	C12orf45			Y																						Y	Y	Y	Y						Y										
4095	A_23_P99204	C12orf47			Y	Y	Y	Y		Y	Y	Y															Y																			
C9975.1	A_23_P87769	C12orf48	Y	Y																																										
15603	A_23_P13797	C12orf49	Y	Y	Y	Y		Y	Y	Y																	Y	Y	Y	Y						Y										
20694	A_23_P128486	C12orf5	Y	Y																										Y						Y										
12192	A_23_P309292	C12orf51				Y			Y	Y																				Y						Y										
17412	A_23_P162702	C12orf51			Y	Y		Y		Y	Y	Y	Y	Y				Y		Y		Y					Y	Y	Y	Y																
17859	A_23_P25282	C12orf53																		Y																										
15295	A_23_P405878	C12orf54			Y	Y		Y		Y																																				
4365	A_23_P350551	C12orf57	Y	Y	Y	Y		Y		Y								Y	Y	Y	Y			Y			Y																			
2865	A_23_P318938	C12orf59			Y	Y		Y	Y	Y																	Y	Y	Y	Y						Y										
16238	A_23_P364544	C12orf60			Y													Y		Y			Y				Y																			
7011	A_23_P151280	C12orf62							Y	Y																																				
1213	A_23_P408175	C12orf67																		Y	Y		Y				Y						Y													
7444	A_23_P368723	C13orf1			Y	Y	Y	Y	Y	Y	Y	Y	Y	Y				Y	Y	Y	Y	Y	Y	Y	Y	Y	Y	Y	Y	Y	Y	Y	Y	Y	Y	Y					Y					
10845	A_23_P348169	C13orf16				Y												Y	Y	Y		Y		Y				Y																		
14550	A_23_P205102	C13orf23																									Y	Y	Y	Y																
1561	A_23_P362046	C13orf27	Y	Y	Y														Y	Y		Y					Y																			
12566	A_23_P65302	C13orf28			Y																						Y																			
2823	A_23_P368909	C13orf29																										Y																		
10928	A_23_P14216	C13orf38													Y			Y	Y	Y	Y	Y		Y																						
7983	A_23_P25935	C14orf1			Y				Y	Y										Y																										
570	A_23_P380965	C14orf100																		Y			Y				Y																			
17320	A_23_P205580	C14orf100			Y															Y		Y					Y	Y	Y	Y																
11912	A_23_P205607	C14orf101			Y	Y	Y	Y	Y	Y	Y	Y							Y	Y		Y					Y	Y	Y	Y																
20171	A_23_P140355	C14orf115			Y																						Y																			
21771	A_23_P54198	C14orf118																	Y	Y		Y																								
12643	A_23_P37144	C14orf126																	Y	Y			Y																							
16723	A_23_P151525	C14orf132			Y	Y	Y	Y	Y	Y	Y	Y	Y	Y					Y								Y	Y	Y	Y						Y										
1798	A_23_P163148	C14orf133																		Y		Y																								
7869	A_23_P163059	C14orf135			Y	Y	Y	Y	Y	Y	Y	Y	Y	Y				Y	Y	Y		Y	Y	Y			Y	Y	Y	Y						Y										
6679	A_23_P128940	C14orf138			Y	Y		Y		Y																																				
15004	A_23_P14302	C14orf139																Y																												
234	A_23_P99571	C14orf142							Y	Y	Y	Y	Y	Y						Y			Y				Y																			
11475	A_23_P129014	C14orf143			Y	Y	Y	Y	Y	Y	Y	Y	Y	Y						Y			Y				Y			Y						Y										
2675	A_23_P320829	C14orf145																		Y																										
17496	A_23_P430201	C14orf145							Y	Y																				Y						Y										
9986	A_23_P382682	C14orf148			Y																						Y			Y						Y										
21758	A_23_P162879	C14orf153			Y						Y		Y	Y													Y																			
2667	A_23_P128986	C14orf156							Y	Y																	Y																			
21214	A_23_P48771	C14orf159																Y			Y	Y																								
19549	A_23_P14482	C14orf166				Y																																								
2933	A_23_P48728	C14orf179	Y	Y	Y	Y		Y	Y	Y										Y		Y	Y				Y	Y	Y	Y						Y										
13756	A_23_P205281	C14orf2																Y		Y	Y																									
9898	A_23_P76983	C14orf45			Y	Y	Y	Y	Y	Y	Y	Y	Y	Y				Y	Y	Y	Y	Y	Y	Y	Y	Y	Y	Y	Y	Y	Y	Y	Y			Y					Y					
11663	A_23_P411851	C14orf49				Y																																								
21483	A_23_P309973	C14orf50			Y																																									
6575	A_23_P105939	C14orf73				Y			Y	Y																				Y						Y										
10234	A_23_P420981	C14orf79	Y	Y					Y	Y																				Y						Y										
8751	A_23_P48581	C14orf93			Y				Y	Y								Y		Y							Y																			
11440	A_23_P65448	C14orf94																					Y				Y																			
14827	A_23_P88466	C15orf2																Y		Y																										
18163	A_23_P140705	C15orf23			Y																						Y																			
6684	A_23_P88680	C15orf24							Y	Y																																				
18139	A_23_P336612	C15orf26			Y													Y	Y	Y		Y		Y			Y			Y						Y										
14825	A_23_P14649	C15orf28			Y	Y		Y	Y	Y																		Y		Y						Y										
3722	A_23_P77279	C15orf29																		Y																										
18108	A_23_P368882	C15orf33				Y														Y			Y					Y																		
5382	A_23_P37455	C15orf34							Y	Y																																				
11997	A_23_P37514	C15orf39	Y	Y	Y				Y	Y								Y	Y				Y							Y						Y										
7950	A_23_P77201	C15orf41							Y	Y																	Y																			
4818	A_23_P345707	C15orf42							Y	Y			Y					Y	Y	Y		Y		Y			Y																			
11974	A_23_P325596	C15orf43			Y													Y	Y	Y		Y		Y			Y			Y						Y										
15867	A_23_P37474	C15orf44			Y															Y							Y																			
5465	A_23_P320858	C15orf49			Y	Y		Y		Y																	Y																			
8752	A_23_P54447	C15orf5			Y	Y	Y	Y	Y	Y	Y	Y						Y		Y		Y					Y	Y	Y	Y																
21660	A_23_P26143	C15orf61							Y	Y	Y	Y	Y	Y				Y	Y	Y		Y		Y						Y						Y										

15464	A_23_P206498	C16orf13																		Y		Y																								
10238	A_23_P3663	C16orf14																	Y	Y		Y																								
21256	A_23_P37840	C16orf3			Y	Y		Y	Y	Y	Y	Y	Y	Y													Y	Y	Y	Y						Y								Y		
21308	A_23_P100326	C16orf35																			Y																									
6094	A_23_P88757	C16orf42			Y	Y	Y	Y	Y	Y	Y	Y						Y	Y	Y		Y	Y	Y			Y			Y			Y			Y										
21154	A_23_P326319	C16orf45	Y	Y	Y	Y		Y	Y	Y	Y	Y	Y	Y				Y	Y	Y			Y	Y			Y	Y	Y	Y						Y										
16273	A_23_P410700	C16orf46																			Y																									
4580	A_23_P14972	C16orf48			Y	Y		Y		Y																	Y																			
19454	A_23_P15242	C16orf5			Y				Y	Y																																				
6663	A_23_P339053	C16orf52							Y	Y								Y		Y		Y								Y						Y										
725	A_23_P77422	C16orf53	Y	Y	Y	Y		Y		Y																				Y						Y										
721	A_23_P118018	C16orf54			Y																																									
15471	A_23_P206272	C16orf57			Y						Y		Y	Y																Y						Y										
639	A_23_P26557	C16orf59																Y	Y	Y				Y																						
11195	A_23_P106544	C16orf61	Y	Y	Y	Y		Y	Y	Y	Y	Y	Y	Y					Y								Y	Y	Y	Y						Y										
5226	A_23_P390744	C16orf63			Y	Y		Y		Y										Y							Y			Y						Y										
8467	A_23_P412501	C16orf65																			Y																									
6199	A_23_P129476	C16orf7			Y	Y	Y	Y	Y	Y	Y	Y	Y	Y					Y	Y		Y	Y				Y	Y	Y	Y						Y										
7192	A_23_P414281	C16orf71			Y	Y		Y	Y	Y	Y	Y	Y	Y				Y	Y	Y	Y	Y	Y	Y	Y	Y	Y	Y	Y	Y	Y	Y	Y	Y												
8650	A_23_P398545	C16orf73																									Y																			
20060	A_23_P408296	C16orf78			Y	Y		Y		Y																	Y																			
14055	A_23_P3514	C16orf80			Y	Y		Y	Y	Y	Y	Y	Y	Y																Y						Y										
19495	A_23_P49279	C16orf87	Y	Y	Y				Y	Y	Y	Y	Y	Y													Y			Y						Y										
6727	A_23_P66158	C16orf88			Y	Y		Y	Y	Y	Y	Y	Y	Y													Y																			
21462	A_23_P118412	C17orf28			Y	Y		Y	Y	Y	Y	Y	Y	Y				Y									Y			Y						Y										
10389	A_23_P3982	C17orf37				Y			Y	Y	Y	Y	Y	Y																Y						Y										
9064	A_23_P33607	C17orf42			Y	Y		Y		Y																	Y																			
4621	A_23_P363896	C17orf45	Y	Y																																										
18061	A_23_P55127	C17orf48																		Y			Y																							
4112	A_23_P141520	C17orf49			Y	Y		Y		Y												Y					Y																			
21711	A_23_P402932	C17orf50			Y	Y		Y		Y										Y			Y				Y																			
7244	A_23_P129967	C17orf53																				Y																								
16213	A_23_P301340	C17orf56																		Y																										
3379	A_23_P152962	C17orf59			Y	Y		Y	Y	Y										Y			Y				Y			Y						Y										
1142	A_23_P84438	C17orf60			Y															Y																										
2438	A_23_P118408	C17orf62			Y	Y	Y	Y	Y	Y	Y	Y						Y	Y	Y	Y	Y	Y	Y	Y	Y	Y				Y		Y													
4072	A_23_P141484	C17orf63			Y	Y		Y		Y								Y	Y	Y	Y	Y		Y			Y																			
16725	A_23_P430788	C17orf66			Y				Y	Y										Y		Y	Y				Y			Y						Y										
9853	A_23_P78190	C17orf68																		Y							Y																			
17124	A_23_P375434	C17orf69	Y	Y	Y	Y	Y	Y	Y	Y	Y	Y	Y	Y					Y	Y		Y	Y				Y	Y	Y	Y						Y										
2065	A_23_P113417	C17orf70			Y													Y		Y		Y	Y				Y			Y						Y										
14285	A_23_P66560	C17orf71																										Y																		
2680	A_23_P15639	C17orf75																					Y				Y																			
4745	A_23_P152858	C17orf79			Y																						Y	Y	Y	Y																
10434	A_23_P207927	C17orf80			Y	Y	Y	Y	Y	Y	Y	Y								Y							Y	Y	Y	Y																
10652	A_23_P15671	C17orf85			Y	Y	Y	Y	Y	Y	Y	Y	Y	Y				Y	Y	Y		Y		Y			Y			Y						Y										
15405	A_23_P49697	C17orf88			Y																						Y																			
20264	A_23_P10714	C17orf90			Y	Y		Y		Y									Y	Y		Y	Y				Y																			
2094	A_23_P49610	C17orf91				Y			Y	Y																				Y						Y										
16187	A_23_P141597	C17orf99				Y														Y			Y																							
9925	A_23_P207939	C18orf1			Y	Y		Y		Y																		Y																		
3348	A_23_P50052	C18orf12			Y				Y	Y										Y			Y				Y																			
16338	A_23_P435649	C18orf16			Y																						Y																			
10308	A_23_P377965	C18orf18							Y	Y																				Y						Y										
13149	A_23_P380978	C18orf19							Y	Y																																				
18078	A_23_P153128	C18orf2			Y																																									
20947	A_23_P101237	C18orf21			Y																																									
9885	A_23_P153170	C18orf24			Y	Y		Y	Y	Y	Y	Y	Y	Y													Y			Y						Y										
15032	A_23_P412554	C18orf25				Y			Y	Y																				Y						Y										
22180	A_23_P89824	C18orf45																		Y																										
15670	A_23_P78410	C18orf55																		Y																										
390	A_23_P78385	C18orf8			Y	Y	Y	Y		Y	Y	Y															Y	Y	Y	Y																
10095	A_23_P67508	C19orf10			Y	Y	Y	Y	Y	Y	Y	Y															Y																			
17484	A_23_P209195	C19orf12	Y	Y	Y	Y		Y	Y	Y	Y	Y	Y	Y														Y																		
1537	A_23_P303803	C19orf18																		Y																										
871	A_23_P390065	C19orf21			Y				Y	Y	Y	Y	Y	Y													Y			Y						Y										
15280	A_23_P315286	C19orf22							Y	Y								Y	Y	Y			Y	Y																						
21676	A_23_P332937	C19orf23			Y	Y		Y	Y	Y																	Y	Y	Y	Y						Y										
14068	A_23_P130926	C19orf24																		Y			Y																							
10219	A_23_P424597	C19orf25																				Y																								
22484	A_23_P308631	C19orf26			Y	Y		Y	Y	Y																																				
952	A_23_P345139	C19orf28			Y	Y		Y	Y	Y										Y	Y	Y	Y				Y																			
9097	A_23_P353667	C19orf30				Y																						Y																		
8387	A_23_P208788	C19orf33			Y				Y	Y	Y	Y	Y	Y				Y	Y	Y		Y		Y			Y																			
18076	A_23_P50674	C19orf36	Y	Y																																										
17920	A_23_P142055	C19orf38	Y		Y	Y	Y	Y	Y	Y	Y	Y							Y	Y							Y	Y	Y	Y						Y										
8878	A_23_P353085	C19orf39			Y				Y	Y																																				

11209	A_23_P101521	C19orf41																		Y																									
14030	A_23_P153357	C19orf42			Y	Y	Y	Y	Y	Y	Y	Y						Y	Y	Y	Y	Y	Y	Y	Y	Y	Y	Y	Y	Y	Y	Y				Y											
7354	A_23_P164821	C19orf43			Y	Y	Y	Y	Y	Y	Y	Y	Y	Y					Y	Y	Y	Y	Y				Y																				
14938	A_23_P27656	C19orf48			Y													Y	Y	Y	Y	Y	Y	Y	Y	Y	Y				Y																
19021	A_23_P50722	C19orf50			Y	Y	Y	Y		Y	Y	Y	Y	Y				Y									Y																				
16702	A_23_P310532	C19orf52			Y	Y	Y	Y	Y	Y	Y	Y	Y	Y						Y							Y	Y	Y	Y						Y											
9034	A_23_P218486	C19orf56																			Y		Y																								
12988	A_23_P330561	C19orf59			Y	Y		Y		Y																				Y						Y											
8825	A_23_P90375	C19orf6			Y	Y		Y	Y	Y										Y	Y	Y	Y																								
18271	A_23_P67589	C19orf60			Y	Y	Y	Y	Y	Y	Y	Y	Y	Y				Y	Y	Y	Y	Y	Y	Y	Y	Y	Y	Y	Y	Y	Y	Y	Y	Y	Y	Y											
13827	A_23_P56135	C19orf61			Y	Y		Y		Y								Y	Y	Y	Y	Y	Y	Y	Y	Y	Y				Y																
15187	A_23_P119715	C19orf62			Y	Y	Y	Y	Y	Y	Y	Y	Y	Y				Y	Y	Y		Y		Y			Y	Y	Y	Y																	
5971	A_23_P208674	C19orf63																	Y																												
5161	A_23_P4922	C19orf68			Y	Y		Y		Y																																					
457	A_23_P252145	C1GALT1	Y	Y	Y																																										
21597	A_23_P159839	C1GALT1C1			Y	Y		Y		Y																	Y																				
1560	A_23_P103433	C1orf102	Y	Y	Y	Y	Y	Y	Y	Y	Y	Y	Y	Y				Y	Y	Y	Y	Y	Y	Y	Y	Y	Y	Y	Y	Y	Y	Y	Y	Y	Y	Y											
13064	A_23_P160689	C1orf103																Y	Y	Y			Y	Y																							
1287	A_23_P343977	C1orf104			Y	Y		Y		Y								Y	Y				Y				Y			Y						Y											
8548	A_23_P1043	C1orf106			Y	Y		Y	Y	Y									Y	Y		Y					Y	Y	Y	Y						Y											
8056	A_23_P46337	C1orf107			Y				Y	Y													Y							Y						Y											
17479	A_23_P45970	C1orf109			Y																																										
6159	A_23_P11862	C1orf112																		Y																									
11334	A_23_P307536	C1orf113			Y	Y	Y	Y	Y	Y	Y	Y	Y	Y					Y	Y	Y	Y	Y				Y	Y	Y	Y						Y											
6267	A_23_P468	C1orf114	Y		Y	Y		Y		Y								Y									Y	Y	Y	Y						Y											
21964	A_23_P897	C1orf116			Y				Y	Y									Y								Y																				
11052	A_23_P23017	C1orf123			Y	Y		Y		Y																																					
21002	A_23_P433791	C1orf124																		Y																									
7655	A_23_P356425	C1orf125			Y															Y			Y																								
9248	A_23_P392235	C1orf125																												Y						Y											
14027	A_23_P309903	C1orf127							Y	Y																																					
5214	A_23_P200079	C1orf128																Y																													
11891	A_23_P136571	C1orf14																				Y					Y																				
18458	A_23_P49467	C1orf144			Y	Y		Y		Y								Y	Y	Y		Y		Y			Y																				
1412	A_23_P103442	C1orf149			Y	Y		Y	Y	Y																	Y																				
4921	A_23_P408787	C1orf150				Y																																									
1557	A_23_P350062	C1orf152			Y	Y	Y	Y	Y	Y	Y	Y	Y	Y					Y	Y	Y	Y	Y				Y	Y	Y	Y						Y											
21804	A_23_P85726	C1orf156																		Y																									
14691	A_23_P74668	C1orf158																					Y																								
15916	A_23_P160828	C1orf159			Y	Y		Y	Y	Y	Y	Y	Y	Y				Y	Y	Y		Y	Y	Y			Y			Y						Y											
6582	A_23_P343366	C1orf161			Y				Y	Y																	Y			Y						Y											
14062	A_23_P412562	C1orf162	Y	Y	Y										Y				Y	Y										Y						Y											
6341	A_23_P114566	C1orf163			Y				Y	Y	Y	Y	Y	Y													Y			Y						Y											
17321	A_23_P135742	C1orf170			Y				Y	Y																	Y			Y						Y											
14460	A_23_P800	C1orf172																			Y												Y														
7716	A_23_P160226	C1orf175							Y	Y																																					
11675	A_23_P394960	C1orf182			Y															Y							Y																				
13718	A_23_P149322	C1orf183			Y	Y		Y		Y									Y								Y	Y	Y	Y						Y											
6396	A_23_P95640	C1orf186																	Y	Y																									
1349	A_23_P103690	C1orf2																Y	Y	Y		Y		Y																							
11793	A_23_P149695	C1orf203			Y																						Y																				
18345	A_23_P113161	C1orf21	Y	Y	Y	Y	Y	Y	Y	Y	Y	Y	Y	Y					Y	Y	Y	Y	Y				Y	Y	Y	Y	Y	Y	Y	Y	Y	Y											
9066	A_23_P314666	C1orf212			Y	Y	Y	Y		Y	Y	Y							Y			Y					Y	Y	Y	Y																	
17309	A_23_P321370	C1orf216			Y														Y									Y																			
14932	A_23_P103511	C1orf226	Y		Y	Y		Y	Y	Y										Y							Y	Y	Y	Y						Y											
7614	A_23_P126129	C1orf25			Y	Y		Y		Y									Y	Y			Y				Y	Y	Y	Y																	
22207	A_23_P96931	C1orf26				Y			Y	Y										Y		Y																									
C10304.5	A_23_P282	C1orf27																		Y																									
1389	A_23_P873	C1orf38	Y	Y	Y	Y		Y	Y	Y					Y				Y	Y							Y																				
19916	A_23_P410712	C1orf51																	Y	Y			Y																								
14745	A_23_P74778	C1orf54	Y															Y																													
18520	A_23_P382829	C1orf56			Y		Y		Y	Y	Y	Y								Y			Y				Y																				
11624	A_23_P309361	C1orf59			Y																						Y																				
920	A_23_P201017	C1orf61			Y																						Y			Y						Y											
17022	A_23_P34396	C1orf63																					Y																								
6156	A_23_P376172	C1orf64			Y	Y		Y	Y	Y	Y	Y	Y	Y								Y						Y		Y						Y											
11369	A_23_P97712	C1orf68			Y	Y		Y		Y									Y				Y				Y																				
1240	A_23_P405175	C1orf71																										Y																			
16617	A_23_P348281	C1orf83				Y			Y	Y																		Y																			
12381	A_23_P74493	C1orf85							Y	Y										Y																									
18603	A_23_P386380	C1orf87			Y	Y	Y	Y	Y	Y	Y	Y								Y		Y	Y				Y	Y	Y	Y						Y											
12060	A_23_P126499	C1orf89																		Y		Y																							
21494	A_23_P104054	C1orf9			Y	Y	Y	Y	Y	Y	Y	Y								Y								Y																			
14421	A_23_P200396	C1orf91			Y																						Y	Y	Y	Y																	
1724	A_23_P374914	C1orf92																		Y																									
2995	A_23_P357235	C1orf93							Y	Y										Y		Y																							
17146	A_23_P114678	C1QA			Y																																										

1942	A_23_P137366	C1QB	Y		Y				Y	Y	Y	Y	Y	Y				Y	Y	Y		Y	Y	Y			Y			Y						Y										
1364	A_23_P370434	C1QBP																Y																												
4412	A_23_P125974	C1QC			Y														Y	Y			Y				Y			Y						Y										
10899	A_23_P1186	C1QL3			Y																		Y				Y																			
19108	A_23_P3956	C1QTNF1			Y						Y		Y	Y					Y								Y			Y						Y										
6405	A_23_P92899	C1QTNF2																Y		Y																										
711	A_23_P122068	C1QTNF3				Y													Y									Y																		
2348	A_23_P52597	C1QTNF4																					Y																							
3679	A_23_P167237	C1QTNF7			Y														Y	Y		Y																								
2832	A_23_P100330	C1QTNF8			Y	Y		Y		Y										Y			Y					Y		Y						Y										
770	A_23_P400505	C1QTNF9			Y																																									
12708	A_23_P125423	C1R			Y				Y	Y																	Y			Y						Y										
17566	A_23_P204766	C1RL			Y				Y	Y	Y	Y	Y	Y																																
21001	A_23_P363966	C1RL				Y			Y	Y								Y	Y	Y		Y		Y						Y						Y										
8188	A_23_P2492	C1S			Y				Y	Y																	Y																			
539	A_23_P145308	C2	Y	Y	Y	Y	Y	Y	Y	Y	Y	Y			Y			Y		Y	Y	Y																								
20082	A_23_P40295	C20orf103			Y	Y		Y	Y	Y												Y					Y	Y	Y	Y						Y										
17168	A_23_P109089	C20orf107			Y	Y		Y	Y	Y	Y	Y	Y	Y																Y						Y										
12024	A_23_P56969	C20orf11																Y		Y							Y																			
3668	A_23_P91423	C20orf112			Y																						Y																			
6436	A_23_P154784	C20orf114			Y	Y		Y		Y																	Y			Y						Y										
8562	A_23_P5936	C20orf117																				Y	Y																							
20597	A_23_P40315	C20orf12			Y	Y	Y	Y	Y	Y	Y	Y							Y	Y	Y	Y	Y				Y	Y	Y	Y	Y	Y	Y	Y	Y	Y				Y						
11623	A_23_P348767	C20orf132																Y																												
4949	A_23_P210891	C20orf134							Y	Y									Y																											
800	A_23_P131909	C20orf141																									Y																			
13715	A_23_P120364	C20orf149							Y	Y	Y	Y	Y	Y																Y						Y										
20591	A_23_P132041	C20orf152				Y												Y	Y									Y																		
1817	A_23_P91414	C20orf160			Y	Y	Y	Y	Y	Y	Y	Y			Y			Y	Y	Y		Y	Y	Y			Y	Y	Y	Y	Y	Y	Y	Y	Y	Y										
16468	A_23_P68505	C20orf177			Y	Y		Y	Y	Y										Y			Y				Y																			
8371	A_23_P57222	C20orf185			Y																						Y																			
2327	A_23_P80027	C20orf186			Y																						Y																			
13580	A_23_P131754	C20orf195																					Y				Y																			
22314	A_23_P17307	C20orf20				Y												Y	Y	Y				Y				Y																		
7353	A_23_P432506	C20orf200			Y																																									
1257	A_23_P102582	C20orf24	Y	Y	Y	Y	Y	Y	Y	Y	Y	Y	Y	Y													Y	Y	Y	Y						Y										
20778	A_23_P315410	C20orf26			Y	Y		Y		Y																																				
3648	A_23_P28878	C20orf27			Y	Y	Y	Y	Y	Y	Y	Y						Y	Y	Y		Y	Y	Y			Y			Y						Y						Y				
11265	A_23_P57137	C20orf29							Y	Y																				Y						Y										
9468	A_23_P109201	C20orf3							Y	Y																				Y						Y										
21330	A_23_P17482	C20orf30			Y	Y		Y		Y																	Y	Y	Y	Y						Y										
9917	A_23_P251043	C20orf39																	Y																											
8611	A_23_P210619	C20orf43																		Y								Y		Y						Y										
3418	A_23_P328034	C20orf96			Y																									Y						Y										
19469	A_23_P29032	C21orf119			Y	Y		Y		Y								Y	Y	Y		Y		Y			Y	Y	Y	Y																
15876	A_23_P143520	C21orf122																		Y							Y																			
3592	A_23_P413696	C21orf129	Y	Y	Y		Y				Y																Y	Y	Y	Y						Y										
5730	A_23_P211167	C21orf2																	Y				Y																							
15954	A_23_P109333	C21orf33																	Y	Y																										
20330	A_23_P102940	C21orf56			Y	Y		Y	Y	Y								Y	Y	Y		Y	Y	Y			Y	Y	Y	Y																
12699	A_23_P91491	C21orf59			Y				Y	Y	Y	Y	Y	Y				Y		Y		Y	Y				Y																			
17075	A_23_P132079	C21orf62																										Y																		
10990	A_23_P211064	C21orf66			Y																																									
12857	A_23_P211058	C21orf66			Y				Y	Y										Y		Y	Y																							
19581	A_23_P211196	C21orf67			Y	Y		Y		Y								Y	Y								Y																			
8622	A_23_P57283	C21orf7			Y																																									
19190	A_23_P61202	C21orf70			Y	Y		Y	Y	Y									Y	Y		Y	Y				Y	Y	Y	Y						Y										
16182	A_23_P417294	C21orf86			Y	Y	Y	Y	Y	Y	Y	Y																																		
20599	A_23_P372368	C21orf87																												Y						Y										
22534	A_23_P355433	C21orf88			Y	Y		Y	Y	Y										Y																										
14476	A_23_P319557	C21orf89							Y	Y												Y						Y																		
20643	A_23_P211015	C21orf91			Y																																									
10295	A_23_P17642	C21orf96			Y	Y		Y		Y																	Y	Y	Y	Y																
3119	A_23_P17773	C22orf13			Y	Y		Y	Y	Y								Y	Y	Y	Y	Y	Y	Y	Y	Y	Y				Y															
4601	A_23_P68790	C22orf25			Y	Y		Y	Y	Y										Y		Y	Y																							
7205	A_23_P433719	C22orf27																					Y																							
2669	A_23_P68892	C22orf28				Y																																								
15675	A_23_P399726	C22orf29																Y																												
2756	A_23_P211580	C22orf32																			Y												Y													
20696	A_23_P388244	C22orf39			Y	Y		Y		Y	Y	Y	Y	Y				Y	Y	Y		Y	Y	Y			Y	Y	Y	Y																
19379	A_23_P415511	C2orf15			Y	Y		Y	Y	Y	Y	Y	Y	Y				Y		Y			Y				Y			Y			Y			Y										
18928	A_23_P39950	C2orf16																					Y					Y																		
11178	A_23_P16845	C2orf24			Y	Y	Y	Y		Y	Y	Y						Y	Y	Y		Y	Y	Y			Y	Y	Y	Y																
14007	A_23_P120064	C2orf3			Y	Y		Y	Y	Y									Y	Y		Y																								
21709	A_23_P39971	C2orf30			Y																						Y																			
11941	A_23_P5481	C2orf37																		Y			Y																							
6950	A_23_P420256	C2orf43																		Y																										

6077	A_23_P154022	C2orf44				Y			Y	Y																		Y																		
19751	A_23_P131526	C2orf47																					Y																							
3053	A_23_P351328	C2orf53																										Y																		
4815	A_23_P60990	C2orf54			Y				Y	Y																	Y																			
17442	A_23_P314691	C2orf60			Y	Y	Y	Y	Y	Y	Y	Y							Y	Y		Y	Y				Y	Y	Y	Y						Y										
11674	A_23_P257417	C2orf63			Y	Y	Y	Y		Y	Y	Y						Y	Y	Y		Y	Y	Y			Y			Y	Y	Y	Y	Y	Y	Y				Y						
17102	A_23_P39910	C2orf64			Y	Y	Y	Y	Y	Y	Y	Y	Y	Y				Y	Y	Y	Y	Y	Y	Y	Y	Y	Y			Y	Y	Y	Y	Y	Y	Y								Y		
517	A_23_P142916	C2orf69			Y	Y		Y	Y	Y										Y			Y					Y		Y						Y										
1078	A_23_P131596	C2orf7			Y	Y	Y	Y	Y	Y	Y	Y							Y	Y							Y			Y						Y										
4266	A_23_P258748	C2orf83			Y																Y																									
9921	A_23_P2431	C3AR1	Y	Y	Y																																									
10580	A_23_P18413	C3orf1																					Y																							
8052	A_23_P420361	C3orf10			Y																																									
1163	A_23_P29655	C3orf14							Y	Y																																				
4830	A_23_P336670	C3orf17				Y														Y			Y																							
11100	A_23_P155477	C3orf18	Y	Y	Y		Y		Y	Y	Y	Y								Y							Y			Y						Y										
16693	A_23_P57801	C3orf19																												Y						Y										
19182	A_23_P361333	C3orf20																	Y								Y																			
13924	A_23_P345708	C3orf21			Y													Y		Y	Y	Y	Y				Y																			
13177	A_23_P373054	C3orf23			Y	Y		Y	Y	Y										Y	Y	Y	Y				Y	Y	Y	Y						Y										
19463	A_23_P318982	C3orf24			Y				Y	Y																																				
18219	A_23_P144005	C3orf32			Y																						Y																			
21294	A_23_P121465	C3orf34			Y															Y			Y								Y		Y	Y	Y											
10251	A_23_P60947	C3orf37				Y												Y	Y	Y		Y		Y				Y																		
14355	A_23_P251374	C3orf37																Y	Y									Y																		
15173	A_23_P32785	C3orf38																		Y			Y																							
2721	A_23_P18082	C3orf39							Y	Y																																				
21394	A_23_P251268	C3orf42			Y																						Y																			
21549	A_23_P346431	C3orf44																Y	Y	Y				Y				Y																		
18399	A_23_P18055	C3orf51			Y	Y		Y	Y	Y																		Y		Y						Y										
21488	A_23_P58009	C3orf52			Y	Y	Y	Y	Y	Y	Y	Y								Y			Y				Y																			
2218	A_23_P158470	C3orf54			Y	Y		Y	Y	Y									Y	Y			Y				Y																			
7913	A_23_P331790	C3orf57							Y	Y	Y	Y	Y	Y																Y						Y										
19582	A_23_P144134	C3orf58			Y	Y	Y	Y	Y	Y	Y	Y						Y		Y			Y				Y	Y	Y	Y																
3636	A_23_P6651	C3orf63			Y	Y		Y		Y										Y							Y	Y	Y	Y																
1133	A_23_P212310	C3orf75			Y				Y	Y																				Y						Y										
21245	A_23_P42279	C4B							Y	Y	Y	Y	Y	Y							Y									Y						Y								Y		
7194	A_23_P86157	C4BPB				Y													Y	Y								Y		Y						Y										
8114	A_23_P41512	C4orf15			Y	Y		Y		Y										Y			Y																							
4982	A_23_P69788	C4orf16	Y		Y	Y		Y	Y	Y	Y	Y	Y	Y					Y	Y			Y				Y	Y	Y	Y	Y	Y	Y	Y	Y	Y										
18355	A_23_P167298	C4orf17			Y																	Y																								
12526	A_23_P218928	C4orf18	Y	Y	Y	Y		Y	Y	Y								Y	Y	Y		Y		Y			Y	Y	Y	Y						Y										
6176	A_23_P29975	C4orf19			Y																																									
11347	A_23_P18590	C4orf23			Y	Y		Y		Y										Y		Y						Y																		
18299	A_23_P167358	C4orf29			Y	Y	Y	Y	Y	Y	Y	Y	Y	Y				Y		Y		Y	Y				Y	Y	Y	Y						Y										
18055	A_23_P144438	C4orf30																									Y																			
20777	A_23_P58390	C4orf32			Y	Y		Y	Y	Y	Y	Y	Y	Y						Y			Y				Y	Y	Y	Y						Y										
8439	A_23_P433152	C4orf33				Y			Y	Y								Y	Y	Y		Y	Y	Y				Y		Y						Y										
17274	A_23_P112634	C4orf34							Y	Y	Y	Y	Y	Y													Y																			
3612	A_23_P69630	C4orf35			Y																									Y						Y										
10122	A_23_P350454	C4orf38																					Y																							
19211	A_23_P170399	C4orf41			Y	Y	Y	Y	Y	Y	Y	Y						Y	Y	Y		Y	Y	Y			Y	Y	Y	Y						Y										
5431	A_23_P167269	C4orf43			Y	Y		Y		Y										Y							Y			Y						Y										
19136	A_23_P362694	C4orf7																												Y						Y										
19015	A_23_P121502	C4orf8			Y	Y	Y	Y	Y	Y	Y	Y						Y	Y	Y	Y	Y	Y	Y	Y	Y	Y	Y	Y	Y	Y	Y								Y						
16043	A_23_P71855	C5	Y		Y	Y	Y	Y	Y	Y	Y	Y	Y	Y				Y	Y	Y		Y		Y			Y	Y	Y	Y	Y	Y	Y	Y	Y	Y										
20302	A_23_P153562	C5AR1	Y	Y	Y															Y																										
17792	A_23_P81648	C5orf15			Y	Y	Y	Y		Y	Y	Y								Y							Y	Y	Y	Y						Y										
9118	A_23_P81441	C5orf20	Y		Y								Y						Y	Y							Y																			
7311	A_23_P433829	C5orf24																												Y						Y										
9466	A_23_P58538	C5orf26			Y	Y		Y		Y								Y	Y								Y	Y	Y	Y						Y										
4270	A_23_P121875	C5orf28																		Y			Y																							
15861	A_23_P122007	C5orf30	Y	Y	Y	Y	Y	Y	Y	Y	Y	Y	Y	Y				Y	Y	Y	Y	Y	Y	Y	Y	Y	Y	Y	Y	Y	Y	Y	Y	Y	Y	Y										
10103	A_23_P259506	C5orf32																			Y																									
13786	A_23_P337168	C5orf33																		Y	Y		Y																							
19356	A_23_P433111	C5orf35																	Y																											
13663	A_23_P413803	C5orf37			Y	Y		Y	Y	Y	Y	Y	Y	Y						Y		Y	Y				Y	Y	Y	Y						Y										
17499	A_23_P213934	C5orf4																Y	Y			Y						Y																		
12416	A_23_P404606	C5orf41																Y	Y	Y		Y		Y																						
8701	A_23_P213877	C5orf42			Y	Y	Y	Y	Y	Y	Y	Y								Y							Y	Y	Y	Y						Y										
8546	A_23_P121825	C5orf44			Y	Y	Y	Y	Y	Y	Y	Y	Y	Y				Y	Y	Y	Y	Y	Y	Y	Y	Y	Y	Y	Y	Y	Y	Y	Y	Y	Y	Y										
12449	A_23_P58604	C5orf45	Y	Y	Y	Y		Y	Y	Y								Y	Y	Y	Y	Y	Y	Y	Y	Y	Y	Y	Y	Y	Y	Y	Y	Y	Y	Y										
12233	A_23_P19176	C5orf46			Y	Y		Y	Y	Y																				Y						Y										
18504	A_23_P416801	C5orf51																	Y																											
5843	A_23_P81993	C6orf1																												Y						Y										
15512	A_23_P411379	C6orf1																		Y																										
17065	A_23_P156826	C6orf105																Y	Y	Y				Y																						

6984	A_23_P122586	C6orf106			Y	Y		Y	Y	Y								Y	Y	Y		Y	Y	Y			Y	Y	Y	Y						Y										
18582	A_23_P167951	C6orf108																									Y																			
6914	A_23_P134058	C6orf114																												Y						Y										
2619	A_23_P368750	C6orf118			Y	Y		Y	Y	Y			Y														Y	Y	Y	Y						Y										
11165	A_23_P156739	C6orf125																					Y																							
17429	A_23_P133764	C6orf129			Y	Y		Y		Y																	Y	Y	Y	Y						Y										
6420	A_23_P342091	C6orf130																		Y																										
1962	A_23_P70509	C6orf134			Y																						Y																			
18320	A_23_P300827	C6orf136			Y	Y		Y	Y	Y										Y			Y				Y																			
4353	A_23_P386398	C6orf141																									Y																			
19903	A_23_P145555	C6orf142			Y	Y		Y		Y																	Y	Y	Y	Y																
1911	A_23_P59118	C6orf15																				Y																								
1261	A_23_P308581	C6orf153			Y																						Y																			
9123	A_23_P156408	C6orf155			Y	Y	Y	Y	Y	Y	Y	Y	Y	Y				Y		Y	Y	Y	Y				Y			Y			Y			Y										
18880	A_23_P59255	C6orf162																												Y						Y										
951	A_23_P421054	C6orf165							Y	Y																																				
12812	A_23_P168167	C6orf168																				Y																								
1096	A_23_P326325	C6orf170				Y			Y	Y										Y																										
10360	A_23_P59358	C6orf182																	Y								Y																			
1544	A_23_P348749	C6orf199																										Y		Y						Y										
4355	A_23_P168165	C6orf208			Y	Y	Y	Y		Y	Y	Y															Y	Y	Y	Y						Y										
13294	A_23_P254472	C6orf211			Y	Y		Y	Y	Y	Y	Y	Y	Y						Y			Y				Y	Y	Y	Y						Y										
15378	A_23_P134026	C6orf35			Y	Y		Y		Y									Y	Y		Y	Y																							
16895	A_23_P93110	C6orf54																	Y			Y																								
12946	A_23_P256279	C6orf57			Y	Y		Y		Y									Y								Y	Y	Y	Y																
14892	A_23_P93599	C6orf58																Y	Y																											
1409	A_23_P422222	C6orf62				Y																																								
4919	A_23_P42514	C6orf62				Y																																								
5672	A_23_P31109	C6orf72							Y	Y										Y							Y																			
15162	A_23_P59319	C6orf72																									Y	Y	Y	Y						Y										
15822	A_23_P396842	C6orf89			Y	Y	Y	Y		Y	Y	Y						Y	Y	Y		Y		Y			Y	Y	Y	Y																
20514	A_23_P93510	C6orf97	Y	Y	Y	Y	Y	Y	Y	Y	Y	Y	Y	Y						Y			Y				Y	Y	Y	Y						Y										
8951	A_23_P145711	C7orf10																	Y																											
13863	A_23_P31218	C7orf13			Y																						Y																			
8167	A_23_P42836	C7orf20			Y	Y	Y	Y	Y	Y	Y	Y	Y	Y				Y	Y	Y	Y	Y	Y	Y	Y	Y	Y	Y	Y	Y	Y	Y	Y	Y	Y	Y										
2472	A_23_P157283	C7orf23																	Y																											
21858	A_23_P257073	C7orf25			Y	Y		Y		Y								Y	Y	Y	Y	Y	Y	Y	Y	Y	Y	Y	Y	Y	Y	Y														
9779	A_23_P168541	C7orf26																	Y																											
2154	A_23_P251896	C7orf27							Y	Y																				Y						Y										
22501	A_23_P59586	C7orf28A	Y	Y																																										
3006	A_23_P316460	C7orf29																										Y																		
15398	A_23_P145817	C7orf30																					Y																							
15823	A_23_P157316	C7orf34							Y	Y																		Y																		
19016	A_23_P326344	C7orf34			Y																																									
8542	A_23_P403521	C7orf36																Y	Y	Y		Y	Y	Y				Y																		
6881	A_23_P168810	C7orf38			Y	Y		Y		Y										Y			Y																							
13077	A_23_P367544	C7orf41			Y	Y		Y	Y	Y																	Y	Y	Y	Y																
18824	A_23_P93671	C7orf42			Y																																									
5994	A_23_P42642	C7orf43			Y	Y	Y	Y		Y	Y	Y							Y	Y		Y	Y				Y	Y	Y	Y						Y										
16718	A_23_P134477	C7orf50			Y	Y	Y	Y	Y	Y	Y	Y						Y	Y	Y	Y	Y	Y	Y	Y	Y	Y	Y	Y	Y	Y	Y				Y								Y		
2735	A_23_P407654	C7orf51			Y	Y	Y	Y		Y	Y	Y											Y				Y	Y	Y	Y						Y										
21304	A_23_P250097	C7orf54			Y	Y		Y	Y	Y																		Y																		
1797	A_23_P123209	C7orf58			Y	Y		Y		Y								Y		Y							Y																			
1327	A_23_P168705	C7orf60			Y																																									
19289	A_23_P93704	C7orf61																		Y	Y																									
19760	A_23_P8653	C7orf63	Y	Y	Y	Y	Y	Y	Y	Y	Y	Y	Y	Y				Y	Y	Y		Y		Y			Y	Y	Y	Y						Y										
1886	A_23_P46639	C8A																										Y																		
7209	A_23_P35252	C8B																												Y						Y										
2527	A_23_P20713	C8G			Y	Y		Y	Y	Y										Y		Y																								
4037	A_23_P123362	C8orf17			Y	Y		Y		Y									Y									Y																		
11770	A_23_P61268	C8orf30A			Y	Y	Y	Y		Y	Y	Y						Y	Y	Y				Y			Y																			
22074	A_23_P382128	C8orf31			Y				Y	Y																	Y																			
17201	A_23_P33113	C8orf33																	Y		Y																									
17997	A_23_P73311	C8orf34				Y																						Y																		
3268	A_23_P31677	C8orf38			Y	Y		Y	Y	Y																																				
15327	A_23_P134729	C8orf39			Y																						Y																			
20645	A_23_P253345	C8orf4			Y														Y								Y																			
8209	A_23_P123356	C8orf41	Y		Y				Y	Y												Y	Y																							
8738	A_23_P393686	C8orf42							Y	Y																																				
13826	A_23_P168965	C8orf44			Y																	Y	Y																							
4254	A_23_P353614	C8orf46							Y	Y			Y					Y												Y						Y										
13404	A_23_P333029	C8orf47							Y	Y																				Y						Y										
17907	A_23_P301981	C8orf48			Y				Y	Y								Y	Y	Y	Y	Y	Y	Y	Y	Y	Y				Y															
18744	A_23_P71502	C8orf51																Y	Y																											
15239	A_23_P257057	C8orf55			Y	Y	Y	Y	Y	Y	Y	Y	Y	Y				Y	Y	Y	Y	Y	Y	Y	Y	Y	Y	Y	Y	Y	Y	Y				Y										
14818	A_23_P310483	C8orf58							Y	Y																																				
670	A_23_P407595	C8orf6																					Y																							

758	A_23_P31747	C8orf76				Y																																									
15031	A_23_P170649	C8orf84	Y			Y									Y			Y		Y	Y										Y							Y											
11476	A_23_P110736	C9			Y				Y	Y																		Y																					
5330	A_23_P123732	C9orf103			Y	Y		Y	Y	Y	Y	Y	Y	Y				Y		Y										Y						Y											
6900	A_23_P9392	C9orf114							Y	Y											Y		Y	Y																							
5542	A_23_P422115	C9orf116	Y	Y	Y	Y		Y	Y	Y	Y	Y	Y	Y				Y									Y			Y							Y											
7469	A_23_P325626	C9orf126	Y																				Y	Y																							
5677	A_23_P393188	C9orf140																			Y				Y																							
153	A_23_P83007	C9orf150							Y	Y	Y	Y	Y	Y													Y			Y							Y											
15275	A_23_P158048	C9orf16			Y	Y	Y	Y	Y	Y	Y	Y	Y	Y				Y		Y		Y	Y				Y	Y	Y	Y						Y											
3215	A_23_P331092	C9orf163			Y																						Y																					
5169	A_23_P147106	C9orf167																	Y	Y	Y		Y																							
8383	A_23_P169409	C9orf23			Y																																								
3007	A_23_P217009	C9orf24							Y	Y																																				
1138	A_23_P20804	C9orf25			Y	Y		Y	Y	Y											Y	Y		Y							Y						Y											
11598	A_23_P159425	C9orf3																Y					Y																							
9046	A_23_P146584	C9orf30				Y			Y	Y	Y	Y	Y	Y																	Y							Y											
17108	A_23_P60330	C9orf37																			Y	Y																									
7406	A_23_P135031	C9orf4				Y																	Y																							
10221	A_23_P306345	C9orf41																			Y		Y																								
17896	A_23_P216931	C9orf45	Y	Y	Y	Y	Y	Y	Y	Y	Y	Y	Y	Y				Y	Y	Y				Y			Y	Y	Y	Y													Y				
1400	A_23_P255869	C9orf46			Y															Y				Y				Y																					
9856	A_23_P146417	C9orf5			Y	Y		Y		Y									Y	Y		Y	Y				Y	Y	Y	Y																	
16665	A_23_P216564	C9orf6																Y	Y	Y		Y	Y	Y																						
4467	A_23_P422831	C9orf61			Y				Y	Y																																				
8948	A_23_P157766	C9orf68			Y				Y	Y	Y	Y	Y	Y				Y	Y	Y			Y	Y			Y	Y	Y	Y			Y			Y											
78	A_23_P123692	C9orf7							Y	Y											Y		Y	Y																							
6050	A_23_P323836	C9orf75							Y	Y																					Y							Y											
6974	A_23_P9443	C9orf78																				Y																								
1122	A_23_P397899	C9orf80																			Y				Y																							
14002	A_23_P123763	C9orf82			Y	Y		Y		Y										Y		Y	Y																							
8075	A_23_P219181	C9orf86			Y													Y	Y	Y		Y	Y	Y																						
15188	A_23_P32135	C9orf9																			Y																										
21181	A_23_P32285	C9orf91			Y																						Y																					
1369	A_23_P32036	C9orf95																Y	Y	Y				Y																							
16250	A_23_P83200	C9orf98			Y																							Y																				
8649	A_23_P168916	CA1							Y	Y																																				
15121	A_23_P372234	CA12	Y	Y	Y	Y	Y	Y	Y	Y	Y	Y	Y	Y				Y		Y		Y	Y				Y	Y	Y	Y						Y											
18131	A_23_P163336	CA12	Y	Y	Y	Y	Y	Y	Y	Y	Y	Y	Y	Y				Y	Y	Y		Y	Y	Y			Y	Y	Y	Y						Y											
2909	A_23_P63379	CA14	Y	Y	Y	Y		Y	Y	Y					Y			Y		Y							Y	Y	Y	Y						Y											
19981	A_23_P8913	CA2																					Y																							
4405	A_23_P95683	CA5A	Y	Y	Y																						Y																					
7479	A_23_P307346	CA5B			Y																						Y																					
15868	A_23_P126002	CA6			Y	Y	Y	Y	Y	Y	Y	Y	Y	Y				Y	Y	Y		Y		Y			Y	Y	Y	Y						Y											
16234	A_23_P83838	CA8							Y	Y											Y																								
17962	A_23_P158165	CA8							Y	Y											Y																								
20098	A_23_P79419	CAB39																			Y																										
3208	A_23_P204870	CAB39L							Y	Y							Y																											
1705	A_23_P85593	CABC1																	Y																										
18444	A_23_P68827	CABIN1			Y													Y	Y	Y		Y	Y	Y			Y																					
18288	A_23_P411761	CABP1																Y	Y	Y		Y		Y																						
14801	A_23_P138745	CABP2				Y																							Y																				
18597	A_23_P308021	CABP4			Y															Y																										
16975	A_23_P21747	CABP5			Y				Y	Y																	Y																					
21681	A_23_P55984	CABP5				Y																																							
3730	A_23_P314712	CABYR																			Y	Y		Y										Y													
2921	A_23_P39486	CACNA1A																Y	Y				Y																							
15477	A_23_P162534	CACNA1C			Y	Y		Y	Y	Y																	Y	Y	Y	Y						Y											
16780	A_23_P373025	CACNA1C	Y	Y	Y	Y	Y	Y	Y	Y	Y	Y						Y	Y	Y		Y		Y			Y	Y	Y	Y						Y											
22401	A_23_P365767	CACNA1D	Y	Y	Y	Y	Y	Y	Y	Y	Y	Y	Y	Y						Y			Y				Y	Y	Y	Y						Y											
7290	A_23_P34554	CACNA1E				Y														Y																										
16304	A_23_P107247	CACNA1G				Y														Y																										
11466	A_23_P3483	CACNA1H																												Y							Y											
15176	A_23_P85765	CACNA1S				Y															Y																								
21600	A_23_P166866	CACNA2D2				Y																																							
12636	A_23_P147166	CACNA2D3																												Y							Y											
13634	A_23_P353014	CACNA2D4							Y	Y								Y																										
18447	A_23_P25326	CACNA2D4			Y				Y	Y																	Y			Y							Y											
14772	A_23_P206976	CACNB1			Y	Y		Y	Y	Y																	Y	Y	Y	Y						Y											
3843	A_23_P96242	CACNB2			Y																						Y																					
13860	A_23_P326852	CACNB2	Y		Y															Y																										
6167	A_23_P371794	CACNB3			Y															Y			Y				Y			Y							Y											
15942	A_23_P204019	CACNB3							Y	Y																					Y							Y											
5386	A_23_P209227	CACNB4							Y	Y																																				
13486	A_23_P84000	CACNG2							Y	Y																																				
8246	A_23_P66177	CACNG3			Y																						Y																					
6033	A_23_P77899	CACNG4							Y	Y																					Y							Y											

1204	A_23_P164789	CACNG6			Y	Y		Y	Y	Y									Y	Y		Y	Y				Y																		
19366	A_23_P4782	CACNG7			Y																						Y																		
21354	A_23_P420218	CACNG8			Y															Y																									
19839	A_23_P74034	CACYBP	Y	Y																							Y																		
13440	A_23_P40044	CAD																		Y		Y																							
2659	A_23_P203120	CADM1			Y		Y		Y	Y	Y	Y							Y																										
10736	A_23_P201156	CADM3																		Y		Y	Y																						
6655	A_23_P31555	CADPS2			Y				Y	Y	Y	Y	Y	Y													Y			Y						Y										
7331	A_23_P21092	CALB2			Y				Y	Y																				Y						Y										
19205	A_23_P53096	CALCB																												Y						Y										
18375	A_23_P98995	CALCOCO1																Y		Y																									
6762	A_23_P42575	CALD1																		Y																									
5534	A_23_P202219	CALHM2																					Y																						
351	A_23_P163179	CALM1			Y										Y																														
22418	A_23_P326170	CALM2				Y																																							
15652	A_23_P4944	CALM3			Y	Y		Y		Y								Y	Y	Y	Y	Y	Y	Y	Y	Y	Y				Y														
11929	A_23_P393080	CALML3	Y	Y	Y	Y	Y	Y	Y	Y	Y	Y								Y	Y	Y	Y				Y			Y						Y										
15726	A_23_P60617	CALML3			Y																						Y																		
14705	A_23_P129188	CALML4																												Y						Y										
2893	A_23_P124095	CALML5			Y	Y		Y	Y	Y																	Y	Y	Y	Y						Y										
16137	A_23_P43841	CALML5			Y	Y		Y	Y	Y																	Y	Y	Y	Y						Y										
729	A_23_P147698	CALN1			Y	Y	Y	Y	Y	Y	Y	Y	Y	Y													Y			Y						Y										
15504	A_23_P67288	CALR			Y																																								
18269	A_23_P122815	CALU			Y				Y	Y	Y	Y	Y	Y				Y		Y							Y																		
3992	A_23_P121215	CAMK1			Y													Y	Y	Y		Y	Y	Y																					
17222	A_23_P124252	CAMK1D																		Y																									
3792	A_23_P97401	CAMK1G			Y				Y	Y																	Y			Y						Y										
194	A_23_P42882	CAMK2B			Y	Y		Y	Y	Y																	Y																		
2446	A_23_P144458	CAMK2D			Y																																								
3387	A_23_P46903	CAMK2G			Y	Y	Y	Y	Y	Y	Y	Y						Y	Y	Y			Y	Y			Y			Y						Y										
2684	A_23_P11800	CAMK2N1			Y	Y	Y	Y	Y	Y	Y	Y	Y	Y													Y	Y	Y	Y						Y										
1766	A_23_P18404	CAMK2N2																		Y	Y	Y																							
2067	A_23_P213728	CAMLG			Y	Y		Y	Y	Y								Y	Y	Y		Y	Y	Y			Y	Y	Y	Y						Y										
9654	A_23_P138117	CAMTA1																												Y						Y										
9198	A_23_P365189	CAMTA2																		Y																									
15114	A_23_P250102	CAND2							Y	Y																				Y						Y										
13997	A_23_P26756	CANT1							Y	Y									Y											Y						Y										
2176	A_23_P421664	CAP2	Y	Y	Y	Y	Y	Y	Y	Y	Y	Y	Y	Y				Y	Y	Y	Y	Y	Y	Y	Y	Y	Y	Y	Y	Y	Y	Y	Y	Y	Y	Y										
16802	A_23_P156417	CAP2	Y	Y	Y	Y	Y	Y	Y	Y	Y	Y	Y	Y				Y	Y	Y	Y	Y	Y	Y	Y	Y	Y	Y	Y	Y	Y	Y	Y	Y	Y	Y										
3980	A_23_P138834	CAPN1			Y	Y	Y	Y	Y	Y	Y	Y						Y	Y	Y	Y	Y	Y	Y	Y	Y	Y	Y	Y	Y	Y	Y				Y										
15352	A_23_P101972	CAPN13							Y	Y	Y	Y	Y	Y																Y						Y										
16186	A_23_P23924	CAPN2																Y		Y																									
8476	A_23_P218190	CAPN3			Y	Y		Y		Y																	Y			Y						Y										
3066	A_23_P105197	CAPN5			Y														Y	Y	Y	Y	Y																						
3181	A_23_P217570	CAPN6			Y				Y	Y																	Y			Y						Y										
7514	A_23_P381378	CAPN7			Y													Y	Y	Y		Y	Y	Y			Y			Y	Y	Y	Y	Y	Y	Y										
13113	A_23_P80598	CAPN7			Y	Y		Y	Y	Y										Y	Y	Y	Y				Y																		
1025	A_23_P83381	CAPN8	Y	Y	Y	Y	Y	Y	Y	Y	Y	Y	Y	Y				Y	Y	Y	Y	Y	Y	Y	Y	Y	Y	Y	Y	Y	Y	Y	Y			Y										
9005	A_23_P149427	CAPN9																	Y			Y																							
10639	A_23_P67648	CAPNS1			Y	Y		Y	Y	Y								Y	Y	Y	Y	Y	Y	Y	Y	Y	Y				Y														
16994	A_23_P152406	CAPNS2			Y	Y		Y		Y								Y	Y	Y				Y			Y																		
1044	A_23_P98722	CAPRIN1			Y	Y		Y	Y	Y										Y			Y				Y																		
21528	A_23_P87532	CAPRIN2			Y	Y		Y	Y	Y												Y	Y				Y																		
19331	A_23_P78958	CAPS																Y	Y																										
14281	A_23_P380376	CAPS2			Y	Y		Y		Y								Y	Y	Y			Y	Y			Y	Y	Y	Y						Y										
7889	A_23_P133536	CAPSL			Y	Y	Y	Y	Y	Y	Y	Y	Y	Y				Y	Y			Y					Y	Y	Y	Y						Y										
13379	A_23_P307940	CAPZA2			Y																																								
19892	A_23_P157231	CAPZA2			Y																																								
7094	A_23_P126752	CAPZB			Y	Y	Y	Y		Y	Y	Y						Y	Y	Y	Y	Y		Y			Y																Y			
15654	A_23_P434890	CARD10			Y				Y	Y																	Y																		
20097	A_23_P109520	CARD10			Y	Y		Y		Y										Y	Y		Y				Y																		
2250	A_23_P82327	CARD11			Y						Y		Y	Y						Y		Y	Y																						
3225	A_23_P64168	CARD16	Y	Y	Y																						Y																		
3570	A_23_P75568	CARD17	Y	Y	Y																						Y			Y						Y										
9200	A_23_P41854	CARD6	Y																																										
4372	A_23_P500433	CARD9			Y	Y		Y	Y	Y										Y										Y						Y										
17861	A_23_P38085	CARHSP1			Y	Y		Y	Y	Y								Y		Y	Y	Y	Y				Y																		
10542	A_23_P88046	CARKD			Y																																								
14548	A_23_P203514	CARS			Y	Y		Y		Y	Y	Y	Y	Y						Y		Y					Y																	Y		
20209	A_23_P128624	CARS2							Y	Y																																			
17173	A_23_P167493	CARTPT			Y																																								
7370	A_23_P95231	CASC1			Y	Y	Y	Y	Y	Y	Y	Y	Y	Y						Y			Y				Y	Y	Y	Y						Y										
21325	A_23_P164354	CASC3			Y													Y	Y	Y		Y	Y	Y																					
12896	A_23_P215607	CASD1							Y	Y										Y			Y																						
15517	A_23_P159821	CASK																	Y	Y																									
6283	A_23_P206466	CASKIN1			Y	Y		Y	Y	Y								Y	Y	Y		Y	Y	Y			Y	Y	Y	Y																
13153	A_23_P44360	CASKIN2																					Y																						

9333	A_23_P202978	CASP1			Y																						Y																			
16347	A_23_P108480	CASP10				Y																																								
18211	A_23_P215701	CASP2			Y															Y	Y	Y		Y		Y			Y																			
5404	A_23_P35912	CASP4			Y				Y	Y																																				
2366	A_23_P47304	CASP5			Y				Y	Y																	Y																			
10477	A_23_P12572	CASP7			Y	Y		Y		Y								Y	Y	Y				Y			Y	Y	Y	Y																
13129	A_23_P58898	CASP8AP2			Y	Y	Y	Y	Y	Y	Y	Y								Y		Y																								
12941	A_23_P97309	CASP9	Y	Y	Y	Y	Y	Y	Y	Y	Y	Y	Y	Y				Y	Y	Y		Y	Y	Y			Y	Y	Y	Y	Y	Y	Y	Y	Y	Y								Y		
499	A_23_P29719	CASR							Y	Y																																				
15641	A_23_P213518	CAST			Y	Y		Y		Y								Y	Y	Y		Y		Y			Y	Y	Y	Y						Y										
6529	A_23_P201295	CASZ1																		Y																										
6298	A_23_P105138	CAT			Y		Y				Y									Y							Y	Y	Y	Y						Y										
2132	A_23_P52681	CATSPER1			Y	Y	Y	Y	Y	Y	Y	Y															Y	Y	Y	Y						Y										
8570	A_23_P88489	CATSPER2	Y	Y	Y	Y	Y	Y	Y	Y	Y	Y	Y	Y				Y	Y	Y	Y	Y	Y	Y	Y	Y	Y	Y	Y	Y	Y	Y	Y	Y	Y	Y		Y				Y			Y	
7663	A_23_P77041	CATSPERB							Y	Y	Y	Y	Y	Y																																
15098	A_23_P123071	CAV2			Y	Y		Y		Y										Y							Y			Y						Y										
9562	A_23_P155394	CAV3																			Y																									
4697	A_23_P502884	CBFA2T2																										Y																		
21094	A_23_P166167	CBFA2T2																									Y																			
7260	A_23_P500741	CBFA2T3							Y	Y			Y																																	
13798	A_23_P37794	CBFB			Y																						Y																			
17674	A_23_P113634	CBFB	Y	Y					Y	Y																				Y						Y										
10898	A_23_P53234	CBL			Y	Y		Y		Y																		Y																		
20603	A_23_P212715	CBLB							Y	Y																																				
8592	A_23_P397910	CBLC																Y																												
12806	A_23_P67121	CBLC																Y																												
2466	A_23_P146004	CBLL1																		Y	Y	Y																								
512	A_23_P3155	CBLN3			Y				Y	Y																																				
11868	A_23_P57085	CBLN4																										Y																		
13537	A_23_P29047	CBR1			Y	Y		Y		Y	Y	Y	Y	Y					Y	Y		Y						Y																		
4134	A_23_P213237	CBR4							Y	Y									Y	Y																										
8901	A_23_P166306	CBS			Y	Y		Y	Y	Y										Y							Y	Y	Y	Y						Y										
11913	A_23_P108724	CBWD2			Y	Y		Y		Y										Y			Y					Y																		
11255	A_23_P89621	CBX4																Y	Y	Y	Y	Y	Y	Y	Y	Y					Y															
13038	A_23_P40677	CBX6			Y													Y	Y	Y		Y	Y	Y			Y																			
13508	A_23_P55421	CBX8			Y	Y		Y		Y								Y	Y	Y		Y	Y	Y			Y			Y						Y										
20554	A_23_P68927	CBY1			Y				Y	Y												Y					Y																			
2086	A_23_P55869	CC2D1A			Y				Y	Y									Y	Y	Y	Y	Y				Y			Y						Y										
21350	A_23_P115842	CCAR1			Y	Y	Y	Y	Y	Y	Y	Y								Y		Y						Y																		
17152	A_23_P55544	CCBE1				Y																Y																								
12046	A_23_P46017	CCBL2																	Y	Y		Y	Y																							
21371	A_23_P65983	CCDC102A			Y				Y	Y	Y	Y	Y	Y						Y		Y	Y				Y	Y	Y	Y						Y										
8743	A_23_P44466	CCDC102B																Y					Y																							
113	A_23_P26928	CCDC103	Y	Y	Y	Y		Y	Y	Y								Y		Y							Y	Y	Y	Y																
15853	A_23_P68327	CCDC104			Y	Y		Y	Y	Y									Y	Y		Y	Y				Y	Y	Y	Y						Y										
17875	A_23_P346405	CCDC109A																									Y																			
22021	A_23_P384532	CCDC11			Y	Y		Y		Y										Y		Y					Y																			
2312	A_23_P358470	CCDC111			Y	Y		Y	Y	Y								Y		Y	Y	Y	Y																							
14363	A_23_P163519	CCDC113																Y		Y		Y																								
10683	A_23_P318914	CCDC114							Y	Y																																				
17265	A_23_P17074	CCDC115																		Y	Y																									
1550	A_23_P342108	CCDC116																												Y						Y										
4443	A_23_P432034	CCDC117			Y	Y		Y		Y																	Y	Y	Y	Y																
22503	A_23_P372334	CCDC12																	Y																											
7032	A_23_P73543	CCDC120			Y													Y	Y	Y	Y	Y	Y	Y	Y	Y	Y	Y	Y	Y	Y	Y														
15747	A_23_P5742	CCDC121			Y	Y		Y		Y										Y		Y	Y																							
17693	A_23_P130856	CCDC123				Y			Y	Y																	Y																			
956	A_23_P342118	CCDC124			Y	Y	Y	Y	Y	Y	Y	Y	Y	Y				Y	Y	Y	Y	Y	Y	Y	Y	Y	Y	Y	Y	Y	Y	Y	Y			Y		Y				Y			Y	Y
16103	A_23_P432591	CCDC125			Y	Y		Y	Y	Y	Y	Y	Y	Y				Y	Y	Y	Y	Y		Y			Y			Y						Y										
11035	A_23_P168592	CCDC126																	Y	Y		Y	Y																							
7765	A_23_P101465	CCDC130																		Y		Y																								
15219	A_23_P215614	CCDC132																	Y	Y			Y																							
5238	A_23_P155106	CCDC134			Y	Y	Y	Y	Y	Y	Y	Y	Y	Y				Y	Y	Y		Y		Y			Y	Y	Y	Y						Y										
19898	A_23_P129366	CCDC135			Y																						Y																			
13788	A_23_P384056	CCDC14			Y	Y		Y	Y	Y																	Y																			
20218	A_23_P429478	CCDC140																												Y						Y										
18326	A_23_P424712	CCDC142			Y	Y		Y	Y	Y																				Y						Y										
8842	A_23_P168771	CCDC146			Y	Y	Y	Y	Y	Y	Y	Y	Y	Y				Y	Y	Y	Y	Y	Y	Y	Y	Y	Y	Y	Y	Y	Y	Y				Y										
21750	A_23_P411825	CCDC148	Y		Y	Y		Y	Y	Y										Y							Y																			
3519	A_23_P98477	CCDC15	Y		Y				Y	Y													Y				Y	Y	Y	Y						Y										
6993	A_23_P44942	CCDC15			Y				Y	Y								Y	Y	Y		Y	Y	Y																						
3976	A_23_P39574	CCDC150			Y	Y		Y	Y	Y									Y				Y																							
10180	A_23_P320190	CCDC150																				Y																								
10420	A_23_P326890	CCDC151																		Y		Y																								
7953	A_23_P430800	CCDC17																			Y																									
12265	A_23_P51805	CCDC18																		Y		Y																								
12082	A_23_P62642	CCDC19							Y	Y																																				

11382	A_23_P200876	CCDC21			Y																						Y																		
9523	A_23_P114144	CCDC22			Y	Y		Y	Y	Y								Y	Y	Y	Y	Y	Y	Y	Y	Y	Y	Y	Y	Y	Y	Y				Y										
18037	A_23_P420320	CCDC24																Y	Y			Y																								
11472	A_23_P71300	CCDC25			Y	Y	Y	Y	Y	Y	Y	Y	Y	Y					Y	Y		Y	Y				Y	Y	Y	Y						Y										
8836	A_23_P364487	CCDC26	Y		Y	Y		Y		Y								Y									Y																			
1575	A_23_P31085	CCDC28A			Y	Y		Y		Y								Y		Y							Y																			
14783	A_23_P62764	CCDC28B				Y														Y																										
12336	A_23_P127305	CCDC3			Y																																									
16638	A_23_P77178	CCDC33			Y	Y		Y		Y										Y	Y	Y	Y				Y																			
19774	A_23_P203773	CCDC34				Y																																								
18649	A_23_P162378	CCDC41			Y	Y	Y	Y	Y	Y	Y	Y	Y						Y	Y																										
15665	A_23_P402174	CCDC42			Y	Y		Y	Y	Y																	Y	Y	Y	Y						Y										
833	A_23_P4124	CCDC43			Y				Y	Y										Y							Y																			
15043	A_23_P73142	CCDC44			Y	Y		Y		Y								Y	Y	Y			Y	Y			Y	Y	Y	Y						Y										
557	A_23_P307400	CCDC45				Y	Y		Y	Y	Y	Y								Y							Y	Y	Y	Y																
18200	A_23_P83976	CCDC46							Y	Y																				Y						Y										
8332	A_23_P49616	CCDC47			Y																																									
20357	A_23_P166566	CCDC48							Y	Y	Y	Y	Y	Y																																
7706	A_23_P413796	CCDC5																		Y																										
21470	A_23_P384024	CCDC50							Y	Y																				Y						Y										
11261	A_23_P69362	CCDC51																	Y																											
12426	A_23_P109733	CCDC52			Y	Y	Y	Y	Y	Y	Y	Y	Y	Y					Y	Y			Y				Y	Y	Y	Y																
7527	A_23_P53567	CCDC53			Y															Y							Y																			
13140	A_23_P27180	CCDC55			Y	Y		Y		Y	Y	Y	Y	Y													Y	Y	Y	Y																
17994	A_23_P100802	CCDC56			Y																						Y																			
19853	A_23_P127117	CCDC6																Y	Y																											
19370	A_23_P117026	CCDC64																	Y	Y																										
14227	A_23_P47904	CCDC65							Y	Y																																				
14382	A_23_P363255	CCDC68																				Y	Y																							
2540	A_23_P41096	CCDC72			Y	Y		Y	Y	Y																		Y																		
7150	A_23_P401718	CCDC74B	Y	Y	Y	Y	Y	Y	Y	Y	Y	Y	Y	Y				Y	Y		Y	Y	Y				Y	Y	Y	Y						Y										
17340	A_23_P51324	CCDC76			Y	Y		Y	Y	Y								Y	Y	Y		Y	Y	Y																						
13185	A_23_P99172	CCDC77							Y	Y																																				
20078	A_23_P354864	CCDC78			Y																						Y			Y						Y										
7421	A_23_P164958	CCDC8							Y	Y													Y							Y						Y										
18500	A_23_P128008	CCDC81			Y																						Y			Y						Y										
19806	A_23_P217972	CCDC82			Y				Y	Y												Y					Y			Y						Y										
20007	A_23_P312246	CCDC82	Y	Y																																										
8513	A_23_P150249	CCDC85B			Y		Y				Y								Y	Y	Y	Y	Y				Y																			
3174	A_23_P161918	CCDC86																				Y																								
15629	A_23_P47715	CCDC89																		Y										Y						Y										
16991	A_23_P256618	CCDC90A			Y	Y		Y	Y	Y	Y	Y	Y	Y													Y	Y	Y	Y						Y										
15041	A_23_P162127	CCDC90B																		Y			Y																							
6969	A_23_P162277	CCDC91			Y	Y	Y	Y	Y	Y	Y	Y	Y	Y					Y	Y		Y					Y	Y	Y	Y						Y										
20652	A_23_P98900	CCDC92											Y					Y		Y	Y	Y	Y				Y			Y						Y										
7928	A_23_P91065	CCDC93	Y	Y																										Y						Y										
14568	A_23_P79658	CCDC93			Y	Y	Y	Y	Y	Y	Y	Y								Y		Y					Y			Y						Y										
19695	A_23_P130865	CCDC94			Y	Y	Y	Y		Y	Y	Y								Y		Y					Y			Y						Y										
9063	A_23_P323814	CCDC96							Y	Y	Y	Y	Y	Y				Y		Y	Y	Y								Y						Y								Y		
4922	A_23_P145330	CCHCR1																	Y	Y		Y																								
1127	A_23_P425681	CCK			Y	Y		Y	Y	Y											Y																									
885	A_23_P41395	CCKAR			Y																						Y																			
1795	A_23_P162007	CCKBR							Y	Y								Y																												
8740	A_23_P66635	CCL11	Y	Y																													Y													
3553	A_23_P26965	CCL13	Y	Y	Y	Y	Y	Y		Y	Y	Y						Y		Y							Y	Y	Y	Y																
12665	A_23_P218369	CCL15			Y														Y	Y		Y	Y																							
17483	A_23_P207582	CCL16	Y	Y	Y	Y		Y		Y									Y	Y							Y	Y	Y	Y						Y										
19321	A_23_P26325	CCL17	Y		Y																						Y			Y						Y										
17187	A_23_P55270	CCL18			Y	Y	Y	Y	Y	Y	Y	Y	Y	Y													Y	Y	Y	Y						Y										
3485	A_23_P123853	CCL19			Y														Y								Y																			
5622	A_23_P89431	CCL2			Y	Y		Y	Y	Y													Y					Y		Y						Y										
20743	A_23_P17064	CCL20																	Y																											
18669	A_23_P215491	CCL24							Y	Y																																				
10908	A_23_P55828	CCL25							Y	Y																																				
18362	A_23_P502949	CCL25			Y																									Y						Y										
19740	A_23_P215484	CCL26																									Y																			
11925	A_23_P135248	CCL27																		Y			Y																							
2376	A_23_P503072	CCL28																				Y																								
5904	A_23_P213403	CCL28																				Y																								
15959	A_23_P207569	CCL4	Y	Y	Y	Y		Y	Y	Y																		Y		Y						Y										
14862	A_23_P152837	CCL5	Y		Y				Y	Y									Y																											
21803	A_23_P78037	CCL7	Y	Y	Y	Y		Y	Y	Y								Y										Y		Y						Y										
992	A_23_P207457	CCL8	Y	Y		Y									Y																															
8027	A_23_P123125	CCM2			Y	Y		Y	Y	Y			Y					Y	Y	Y	Y	Y	Y	Y	Y	Y	Y				Y															
2670	A_23_P48414	CCNA1				Y																						Y																		
C8747.9	A_23_P58321	CCNA2			Y				Y	Y								Y		Y							Y																			
21982	A_23_P122197	CCNB1	Y	Y																																										

7326	A_23_P65757	CCNB2	Y	Y														Y	Y	Y		Y		Y						Y						Y										
14792	A_23_P171107	CCNB3			Y	Y		Y		Y																																				
2097	A_23_P202837	CCND1	Y	Y	Y																						Y	Y	Y	Y																
16100	A_23_P139881	CCND2	Y	Y	Y	Y		Y		Y																				Y						Y										
13432	A_23_P214464	CCND3			Y	Y		Y	Y	Y								Y	Y	Y	Y	Y	Y	Y	Y	Y					Y															
21827	A_23_P209200	CCNE1	Y	Y	Y	Y		Y	Y	Y										Y			Y				Y	Y	Y	Y						Y										
8553	A_23_P37954	CCNF																Y	Y	Y	Y	Y	Y	Y	Y	Y	Y				Y		Y													
1313	A_23_P58606	CCNG1				Y			Y	Y										Y	Y		Y																							
15946	A_23_P110122	CCNG2			Y	Y	Y	Y	Y	Y	Y	Y	Y	Y				Y	Y	Y		Y	Y	Y			Y	Y	Y	Y						Y										
11780	A_23_P30338	CCNH			Y	Y	Y	Y	Y	Y	Y	Y	Y	Y				Y	Y	Y		Y	Y	Y			Y	Y	Y	Y						Y										
7014	A_23_P69521	CCNI			Y	Y	Y	Y		Y	Y	Y															Y	Y	Y	Y																
6175	A_23_P24176	CCNJ				Y														Y																										
13864	A_23_P7684	CCNJL																												Y						Y										
9389	A_23_P132456	CCNL1			Y	Y	Y	Y	Y	Y	Y	Y										Y					Y	Y	Y	Y																
19896	A_23_P217899	CCNL2	Y	Y	Y	Y		Y		Y								Y		Y							Y	Y	Y	Y						Y										
18371	A_23_P92860	CCNO							Y	Y	Y	Y	Y	Y																Y						Y										
2494	A_23_P209316	CCNT2			Y	Y		Y	Y	Y																		Y		Y						Y										
18098	A_23_P434419	CCNY			Y				Y	Y	Y	Y	Y	Y				Y	Y	Y	Y	Y	Y	Y	Y	Y	Y				Y		Y													
21316	A_23_P90634	CCNYL1	Y	Y	Y	Y		Y		Y																				Y						Y										
C7406.4	A_23_P48897	CCPG1																Y	Y	Y		Y		Y																						
13073	A_23_P6849	CCR1				Y			Y	Y																																				
5285	A_23_P141367	CCR10			Y	Y		Y	Y	Y									Y	Y	Y	Y	Y				Y																			
12256	A_23_P212354	CCR2	Y	Y	Y																						Y																			
3126	A_23_P412321	CCR5	Y	Y	Y										Y					Y							Y																			
22410	A_23_P343398	CCR7	Y		Y														Y								Y																			
9232	A_23_P212360	CCR9													Y																															
9517	A_23_P20752	CCRK			Y	Y	Y	Y	Y	Y	Y	Y	Y	Y				Y	Y	Y	Y	Y	Y	Y	Y	Y	Y	Y	Y	Y	Y	Y	Y	Y	Y									Y		
1500	A_23_P6909	CCRL1			Y																																									
10649	A_23_P69310	CCRL2	Y	Y	Y		Y		Y	Y	Y	Y			Y												Y			Y						Y										
18788	A_23_P160631	CCT3																Y	Y																											
7496	A_23_P4083	CCT6B																									Y	Y	Y	Y																
6906	A_23_P102401	CCT7			Y				Y	Y								Y	Y								Y			Y						Y										
3688	A_23_P377365	CCT8L2																					Y					Y																		
16564	A_23_P29103	CCT8L2							Y	Y																																				
18590	A_23_P214176	CD109																	Y																											
4754	A_23_P110456	CD14	Y	Y	Y	Y	Y	Y	Y	Y	Y	Y	Y	Y				Y	Y	Y		Y	Y	Y			Y	Y	Y	Y						Y										
12447	A_23_P95470	CD151			Y																	Y																								
12815	A_23_P259783	CD151																				Y																								
584	A_23_P33723	CD163	Y	Y	Y										Y					Y							Y																			
1948	A_23_P61466	CD163L1			Y	Y	Y	Y	Y	Y	Y	Y								Y							Y	Y	Y	Y																
577	A_23_P254756	CD164																										Y																		
7301	A_23_P257815	CD180			Y															Y			Y				Y																			
21796	A_23_P113572	CD19	Y		Y								Y														Y																			
16024	A_23_P402670	CD1A			Y																																									
22507	A_23_P63167	CD1A																	Y								Y			Y						Y										
136	A_23_P351844	CD1B																Y																												
11694	A_23_P34911	CD1B																Y									Y																			
1284	A_23_P51766	CD1C																Y																												
18548	A_23_P74575	CD1D																	Y																											
10719	A_23_P201160	CD1E																Y																												
2785	A_23_P161076	CD2	Y	Y																																										
8279	A_23_P121480	CD200																	Y		Y		Y																							
8928	A_23_P141997	CD209			Y				Y	Y										Y										Y						Y										
11022	A_23_P209059	CD22							Y	Y																				Y						Y										
20783	A_23_P78330	CD226	Y	Y	Y																																									
10224	A_23_P114457	CD24																Y																												
21020	A_23_P34676	CD247	Y	Y	Y																																									
1579	A_23_P48088	CD27			Y																						Y																			
11505	A_23_P54313	CD276																		Y																										
17743	A_23_P393598	CD276			Y				Y	Y	Y	Y	Y	Y				Y		Y		Y	Y				Y			Y						Y										
15709	A_23_P156484	CD2AP				Y			Y	Y																		Y		Y						Y										
21799	A_23_P61552	CD2BP2			Y	Y		Y		Y										Y		Y						Y																		
11778	A_23_P207036	CD300A			Y	Y	Y	Y	Y	Y	Y	Y								Y		Y	Y					Y		Y						Y										
17372	A_23_P26771	CD300C			Y	Y		Y	Y	Y	Y	Y	Y	Y				Y	Y	Y				Y				Y		Y						Y										
13701	A_23_P55020	CD300LF	Y	Y	Y				Y	Y										Y							Y																			
172	A_23_P38310	CD300LG																										Y																		
17517	A_23_P119693	CD320							Y	Y																																				
7135	A_23_P23829	CD34	Y		Y	Y		Y		Y					Y				Y	Y		Y	Y				Y																			
21853	A_23_P111583	CD36																				Y					Y																			
15890	A_23_P142135	CD37	Y		Y	Y	Y	Y	Y	Y	Y	Y							Y	Y	Y		Y				Y	Y	Y	Y	Y	Y	Y	Y	Y											
9905	A_23_P167328	CD38	Y	Y	Y				Y	Y										Y							Y																			
12754	A_23_P138985	CD3D																Y																												
22179	A_23_P416744	CD3E			Y	Y		Y		Y													Y				Y																			
14331	A_23_P105553	CD4			Y														Y								Y																			
11671	A_23_P57036	CD40	Y		Y		Y		Y	Y	Y	Y								Y																										
14592	A_23_P24870	CD44			Y																																									
19558	A_23_P201758	CD46																		Y			Y				Y																			

20486	A_23_P6935	CD47	Y	Y	Y	Y		Y	Y	Y	Y	Y	Y	Y														Y		Y						Y										
1463	A_23_P74145	CD48	Y		Y																																								
15518	A_23_P85800	CD52	Y		Y				Y	Y									Y								Y																		
17038	A_23_P74538	CD53	Y	Y	Y				Y	Y					Y						Y						Y																		
13106	A_23_P374862	CD55																	Y																										
1494	A_23_P138308	CD58	Y	Y	Y																																								
10634	A_23_P75523	CD59																Y	Y	Y				Y																					
2752	A_23_P139162	CD6				Y			Y	Y																	Y	Y	Y	Y															
5496	A_23_P139476	CD63			Y	Y		Y	Y	Y	Y	Y	Y	Y								Y								Y						Y										
1383	A_23_P15394	CD68	Y		Y	Y	Y	Y	Y	Y	Y	Y						Y	Y	Y	Y	Y	Y	Y	Y	Y	Y	Y	Y	Y	Y	Y				Y										
1933	A_23_P118862	CD7							Y	Y								Y									Y			Y						Y										
19625	A_23_P431815	CD7				Y																	Y				Y	Y	Y	Y						Y										
6461	A_23_P119207	CD70			Y	Y	Y	Y	Y	Y	Y	Y	Y	Y																Y						Y										
8919	A_23_P250245	CD72	Y		Y	Y	Y	Y	Y	Y	Y	Y			Y			Y	Y	Y			Y	Y			Y																			
767	A_23_P70095	CD74	Y	Y	Y	Y	Y	Y	Y	Y	Y	Y								Y			Y				Y	Y	Y	Y																
9480	A_23_P107735	CD79A			Y				Y	Y	Y	Y	Y	Y				Y	Y	Y			Y	Y			Y																			
7100	A_23_P207201	CD79B			Y	Y		Y		Y	Y	Y	Y	Y													Y																			
19338	A_23_P13423	CD81																	Y																										
17793	A_23_P1782	CD82	Y		Y	Y	Y	Y	Y	Y	Y	Y	Y	Y						Y		Y	Y				Y	Y	Y	Y						Y										
17337	A_23_P70670	CD83	Y																																										
20731	A_23_P361938	CD84	Y		Y		Y		Y	Y	Y	Y			Y			Y	Y	Y	Y	Y	Y	Y	Y	Y	Y				Y															
20815	A_23_P51253	CD84			Y																																								
20510	A_23_P109988	CD86	Y	Y	Y	Y		Y	Y	Y					Y															Y						Y										
C11652.5	A_23_P159335	CD8B																	Y																										
5636	A_23_P76364	CD9																					Y																						
4958	A_23_P210811	CD93																									Y			Y						Y										
1789	A_23_P32770	CD96							Y	Y																	Y																		
17117	A_23_P44158	CD96							Y	Y								Y																											
15180	A_23_P502312	CD97			Y	Y	Y	Y	Y	Y	Y	Y						Y	Y	Y			Y	Y			Y	Y	Y	Y						Y										
21136	A_23_P217510	CD99			Y										Y					Y			Y				Y																		
3474	A_23_P253052	CD99L2			Y																						Y																		
11645	A_23_P34597	CDA							Y	Y																				Y						Y										
22480	A_23_P48299	CDADC1			Y	Y	Y	Y	Y	Y	Y	Y						Y	Y	Y		Y	Y	Y			Y	Y	Y	Y						Y										
19672	A_23_P150035	CDC123	Y	Y					Y	Y	Y	Y	Y	Y				Y												Y						Y										
1501	A_23_P201921	CDC14A																			Y																								
4909	A_23_P138507	CDC2	Y	Y	Y																						Y																		
7748	A_23_P149195	CDC20			Y													Y	Y	Y		Y		Y			Y	Y	Y	Y						Y										
2134	A_23_P374281	CDC20B																	Y																										
20910	A_23_P133629	CDC23			Y	Y		Y	Y	Y										Y		Y					Y																		
19910	A_23_P121423	CDC25A			Y													Y	Y	Y	Y	Y		Y																					
9099	A_23_P210726	CDC25B			Y	Y	Y	Y	Y	Y	Y	Y	Y	Y				Y	Y	Y	Y	Y	Y	Y	Y	Y	Y			Y	Y	Y	Y	Y	Y	Y				Y						
4600	A_23_P215296	CDC2L5			Y	Y	Y	Y	Y	Y	Y	Y						Y	Y	Y	Y	Y	Y	Y	Y	Y	Y	Y	Y	Y	Y	Y				Y										
3526	A_23_P258447	CDC2L6																					Y																						
7643	A_23_P164999	CDC34			Y	Y	Y	Y	Y	Y	Y	Y	Y	Y				Y	Y	Y	Y	Y	Y	Y	Y	Y	Y	Y	Y	Y	Y	Y				Y								Y		
15236	A_23_P130527	CDC37			Y	Y	Y	Y	Y	Y	Y	Y	Y	Y				Y	Y	Y	Y	Y	Y	Y	Y	Y	Y	Y	Y	Y	Y	Y				Y										
15552	A_23_P20553	CDC37L1			Y				Y	Y									Y	Y		Y	Y				Y			Y						Y										
96	A_23_P33455	CDC40																	Y								Y																		
17049	A_23_P200560	CDC42				Y	Y				Y									Y								Y		Y						Y										
6808	A_23_P256190	CDC42BPA			Y																						Y																		
15682	A_23_P44908	CDC42BPA																									Y																		
5093	A_23_P380766	CDC42BPB			Y				Y	Y																																			
10802	A_23_P99529	CDC42BPB																		Y			Y																						
18992	A_23_P166453	CDC42EP1			Y	Y	Y	Y	Y	Y	Y	Y	Y	Y				Y	Y	Y		Y	Y	Y			Y	Y	Y	Y						Y										
9436	A_23_P209636	CDC42EP3																												Y						Y										
5125	A_23_P66891	CDC42EP4			Y																																								
18443	A_23_P414391	CDC42EP5	Y		Y	Y	Y	Y	Y	Y	Y	Y	Y	Y				Y	Y	Y		Y	Y	Y			Y	Y	Y	Y						Y										
10473	A_23_P167767	CDC42SE2	Y	Y	Y	Y		Y		Y					Y												Y																		
19145	A_23_P430764	CDC42SE2			Y																																								
12990	A_23_P57379	CDC45L			Y													Y	Y								Y																		
10817	A_23_P156471	CDC5L							Y	Y																																			
20699	A_23_P137731	CDC73																					Y																						
20106	A_23_P385861	CDCA2																									Y																		
6237	A_23_P162481	CDCA3																		Y	Y	Y																							
8442	A_23_P205449	CDCA4																	Y	Y	Y	Y					Y																		
11468	A_23_P104651	CDCA5	Y	Y	Y													Y		Y							Y																		
17416	A_23_P251421	CDCA7	Y	Y	Y				Y	Y	Y	Y	Y	Y				Y	Y	Y		Y		Y			Y			Y						Y										
9984	A_23_P375	CDCA8			Y													Y		Y							Y																		
7419	A_23_P32474	CDCP1				Y												Y																											
16722	A_23_P144654	CDH10			Y																						Y	Y	Y	Y						Y										
4284	A_23_P84118	CDH18	Y	Y														Y																											
21259	A_23_P38735	CDH19			Y	Y		Y	Y	Y																	Y	Y	Y	Y															
20045	A_23_P55592	CDH20																Y		Y		Y																							
459	A_23_P40186	CDH22																Y	Y	Y			Y	Y																					
10240	A_23_P364437	CDH23			Y																																								
1294	A_23_P25790	CDH24			Y				Y	Y										Y		Y	Y																						
4005	A_23_P49155	CDH3			Y	Y		Y	Y	Y	Y	Y	Y	Y													Y			Y						Y										
455	A_23_P17593	CDH4																Y	Y	Y		Y		Y																					

18651	A_23_P28997	CDH4			Y				Y	Y	Y	Y	Y	Y				Y		Y	Y	Y	Y				Y			Y						Y						Y				
1867	A_23_P129496	CDH5			Y													Y	Y	Y		Y	Y	Y			Y	Y	Y	Y	Y	Y	Y	Y	Y	Y										
20220	A_23_P214011	CDH6			Y																																							
14362	A_23_P92999	CDH9													Y			Y		Y																								
4483	A_23_P32377	CDK10			Y	Y		Y		Y								Y	Y	Y		Y	Y	Y			Y																			
18797	A_23_P152245	CDK10			Y	Y	Y	Y	Y	Y	Y	Y						Y	Y	Y		Y	Y	Y			Y	Y	Y	Y																
10936	A_23_P98891	CDK2			Y																																							
6279	A_23_P139486	CDK2AP1			Y	Y		Y	Y	Y	Y	Y	Y	Y				Y		Y		Y								Y						Y						Y				
13612	A_23_P104579	CDK2AP2			Y	Y		Y	Y	Y									Y	Y		Y	Y				Y																			
20576	A_23_P89155	CDK3			Y	Y	Y	Y	Y	Y	Y	Y	Y	Y				Y	Y	Y	Y	Y	Y	Y	Y	Y	Y				Y															
11989	A_23_P24997	CDK4			Y	Y		Y		Y								Y	Y	Y		Y		Y			Y																			
8141	A_23_P8400	CDK5			Y	Y		Y		Y								Y	Y	Y	Y	Y	Y	Y	Y	Y	Y				Y															
10377	A_23_P79323	CDK5R2			Y																						Y			Y						Y										
6970	A_23_P257278	CDK5RAP1			Y															Y	Y	Y																						
8323	A_23_P83110	CDK5RAP2				Y																						Y																		
22212	A_23_P168651	CDK6			Y				Y	Y			Y					Y		Y	Y	Y	Y				Y																			
7820	A_23_P133585	CDK7			Y	Y		Y	Y	Y	Y	Y	Y	Y				Y	Y	Y		Y	Y	Y			Y	Y	Y	Y						Y										
16446	A_23_P128572	CDK8			Y	Y	Y	Y	Y	Y	Y	Y	Y	Y				Y	Y	Y	Y	Y		Y			Y	Y	Y	Y						Y										
8339	A_23_P110643	CDKL3			Y	Y	Y	Y	Y	Y	Y	Y						Y	Y	Y	Y	Y	Y	Y	Y	Y	Y			Y	Y	Y				Y				Y						
20878	A_23_P59208	CDKN1A																		Y	Y	Y	Y																					
9655	A_23_P428129	CDKN1C																					Y																					
21531	A_23_P43490	CDKN2A			Y	Y		Y	Y	Y								Y	Y	Y		Y	Y	Y			Y			Y						Y										
4361	A_23_P258002	CDKN2AIP			Y	Y		Y	Y	Y								Y	Y	Y		Y		Y			Y			Y						Y										
9379	A_23_P216815	CDKN2B																Y		Y	Y	Y	Y																					
6932	A_23_P85460	CDKN2C																		Y			Y																					
21205	A_23_P89941	CDKN2D	Y										Y														Y																			
7517	A_23_P48669	CDKN3	Y	Y	Y	Y		Y	Y	Y								Y									Y	Y	Y	Y																
12693	A_23_P98335	CDON							Y	Y																																		
19029	A_23_P125495	CDR1																Y				Y																						
16134	A_23_P3963	CDR2L	Y	Y	Y	Y		Y	Y	Y								Y	Y	Y		Y	Y	Y			Y	Y	Y	Y																
6854	A_23_P427083	CDRT4			Y	Y		Y	Y	Y								Y		Y		Y	Y				Y			Y	Y	Y	Y	Y	Y	Y										
8219	A_23_P7245	CDS1																		Y			Y																					
21112	A_23_P154698	CDS2			Y	Y	Y	Y		Y	Y	Y								Y	Y	Y					Y																			
14165	A_23_P37704	CDT1	Y	Y	Y	Y	Y	Y	Y	Y	Y	Y			Y	Y	Y	Y	Y	Y	Y	Y	Y	Y	Y	Y	Y	Y	Y	Y	Y	Y	Y	Y		Y				Y						
19075	A_23_P114314	CDX4			Y																																							
11202	A_23_P134082	CDYL			Y													Y									Y																			
4374	A_23_P371871	CDYL2			Y														Y	Y		Y	Y				Y																			
887	A_23_P78595	CEACAM21			Y																																							
659	A_23_P218441	CEACAM6							Y	Y																																		
4195	A_23_P375494	CEBPA			Y				Y	Y								Y	Y	Y		Y	Y	Y			Y			Y						Y										
8066	A_23_P208798	CEBPA			Y				Y	Y								Y	Y	Y		Y	Y	Y			Y			Y						Y										
43	A_23_P143243	CEBPB			Y	Y		Y	Y	Y	Y	Y	Y	Y				Y	Y	Y	Y	Y	Y	Y	Y	Y	Y	Y	Y	Y	Y	Y				Y				Y						
9345	A_23_P411296	CEBPB			Y	Y		Y	Y	Y	Y	Y	Y	Y						Y								Y		Y						Y						Y				
674	A_23_P2990	CEBPE			Y	Y		Y	Y	Y	Y	Y	Y	Y						Y		Y	Y				Y	Y	Y	Y						Y										
2185	A_23_P356755	CEBPG			Y				Y	Y																																		
15368	A_23_P208801	CEBPG																	Y	Y																								
13194	A_23_P119964	CEBPZ			Y	Y	Y	Y	Y	Y	Y	Y								Y		Y	Y																					
17609	A_23_P211326	CECR2																					Y																					
10178	A_23_P57370	CECR5			Y	Y	Y	Y	Y	Y	Y	Y	Y	Y				Y	Y	Y	Y	Y	Y	Y	Y	Y	Y	Y	Y	Y	Y	Y	Y	Y	Y	Y										
122	A_23_P123795	CEL			Y	Y		Y	Y	Y										Y			Y				Y	Y	Y	Y						Y										
20185	A_23_P132378	CELSR1	Y	Y	Y	Y	Y	Y	Y	Y	Y	Y	Y	Y				Y	Y	Y	Y		Y	Y	Y		Y	Y	Y	Y	Y	Y	Y	Y	Y	Y										
266	A_23_P92090	CELSR3				Y												Y	Y		Y																									
11637	A_23_P158635	CEND1			Y																																							
13101	A_23_P108910	CENPA	Y	Y	Y	Y		Y	Y	Y	Y	Y	Y	Y				Y	Y	Y		Y		Y			Y	Y	Y	Y						Y										
8199	A_23_P257905	CENPB			Y													Y	Y	Y	Y	Y	Y	Y	Y	Y	Y				Y															
5896	A_23_P167194	CENPC1			Y	Y		Y	Y	Y								Y	Y	Y				Y				Y																		
5242	A_23_P204904	CENPJ	Y																								Y																			
8732	A_23_P126116	CENPL							Y	Y																				Y						Y										
14069	A_23_P103159	CENPM			Y	Y		Y	Y	Y								Y		Y							Y			Y						Y										
8459	A_23_P88740	CENPN	Y	Y																	Y	Y																								
7034	A_23_P50990	CENPO			Y																						Y																			
4326	A_23_P163576	CENPT			Y		Y				Y							Y	Y	Y		Y		Y			Y																			
21770	A_23_P32183	CEP110			Y	Y		Y	Y	Y									Y									Y		Y						Y										
470	A_23_P434929	CEP120			Y	Y		Y		Y																		Y																		
6982	A_23_P212792	CEP135			Y	Y	Y	Y	Y	Y	Y	Y						Y	Y	Y		Y		Y			Y																			
16554	A_23_P18479	CEP135				Y			Y	Y																																		
6659	A_23_P75609	CEP164				Y			Y	Y																																		
6889	A_23_P348857	CEP170																	Y																									
14953	A_23_P23147	CEP170							Y	Y																																		
11120	A_23_P89709	CEP192			Y																																							
8522	A_23_P102832	CEP250																Y	Y	Y	Y	Y	Y	Y	Y	Y					Y															
4212	A_23_P25463	CEP290			Y	Y		Y	Y	Y									Y	Y		Y					Y	Y	Y	Y						Y										
12171	A_23_P36867	CEP290			Y	Y	Y	Y	Y	Y	Y	Y						Y	Y	Y		Y		Y			Y	Y	Y	Y						Y								Y		
3978	A_23_P201816	CEP350			Y	Y	Y	Y	Y	Y	Y	Y	Y	Y					Y	Y	Y	Y	Y				Y	Y	Y	Y												Y				
17003	A_23_P115872	CEP55	Y	Y														Y																												
19823	A_23_P86822	CEP57																	Y																									
6128	A_23_P10002	CEP63			Y	Y	Y	Y	Y	Y	Y	Y							Y	Y		Y	Y				Y	Y	Y	Y																

14561	A_23_P21409	CEP63			Y	Y		Y		Y										Y			Y																							
20548	A_23_P210327	CEP68			Y	Y		Y		Y																		Y																		
2714	A_23_P21473	CEP70																		Y			Y																							
19830	A_23_P32875	CEP70																		Y			Y																							
15158	A_23_P302654	CEP72																	Y																											
1049	A_23_P107513	CEP76																			Y																									
16333	A_23_P353436	CEP78			Y													Y	Y	Y		Y	Y	Y																						
13572	A_23_P143944	CEP97				Y			Y	Y									Y	Y		Y	Y					Y		Y						Y										
8988	A_23_P258357	CER1			Y																						Y			Y						Y										
10340	A_23_P329798	CER1																												Y						Y										
4684	A_23_P83293	CERCAM			Y																		Y				Y																			
22210	A_23_P211659	CERK	Y	Y	Y	Y		Y	Y	Y	Y	Y	Y	Y														Y		Y						Y										
10551	A_23_P206733	CES1			Y																	Y	Y																							
16438	A_23_P218247	CES3																Y																												
11132	A_23_P118234	CES7																									Y																			
21733	A_23_P66974	CETN1			Y	Y		Y	Y	Y										Y			Y				Y			Y						Y										
17063	A_23_P73493	CETN2																		Y																										
10972	A_23_P7732	CETN3			Y	Y	Y	Y		Y	Y	Y	Y							Y	Y	Y	Y				Y	Y	Y	Y																
6681	A_23_P49376	CETP							Y	Y																																				
19733	A_23_P156687	CFB	Y	Y	Y	Y		Y	Y	Y	Y	Y	Y	Y				Y	Y	Y		Y	Y	Y			Y	Y	Y	Y			Y			Y										
16325	A_23_P119562	CFD																	Y																											
11594	A_23_P89123	CFDP1			Y																									Y						Y										
8393	A_23_P114740	CFH																										Y																		
20532	A_23_P953	CFHR2			Y																						Y																			
16435	A_23_P35815	CFL1			Y	Y	Y	Y		Y	Y	Y	Y	Y				Y	Y	Y	Y	Y	Y	Y	Y	Y	Y				Y		Y													
1018	A_23_P65401	CFL2							Y	Y																				Y						Y										
19526	A_23_P209394	CFLAR	Y	Y	Y	Y	Y	Y	Y	Y	Y	Y							Y	Y							Y	Y	Y	Y						Y										
21273	A_23_P395493	CFLP1			Y		Y				Y		Y	Y				Y	Y	Y	Y	Y	Y	Y	Y	Y	Y				Y															
15921	A_23_P22444	CFP	Y		Y														Y				Y				Y																			
6353	A_23_P42386	CGA			Y	Y		Y		Y																	Y																			
4674	A_23_P16301	CGB7																		Y																										
16247	A_23_P363156	CGGBP1			Y	Y	Y	Y		Y	Y	Y								Y		Y	Y				Y	Y	Y	Y																
11722	A_23_P163306	CGNL1			Y	Y		Y	Y	Y								Y	Y	Y		Y	Y	Y			Y	Y	Y	Y						Y										
3084	A_23_P86470	CH25H																	Y																											
1361	A_23_P14863	CHAC1																		Y							Y	Y	Y	Y																
18602	A_23_P28571	CHAC2	Y	Y	Y																						Y																			
13207	A_23_P26976	CHAD			Y	Y		Y	Y	Y	Y	Y	Y	Y													Y	Y	Y	Y																
C12795.4	A_23_P57306	CHAF1B																Y	Y									Y																		
5420	A_23_P371613	CHCHD1							Y	Y																																				
5068	A_23_P251923	CHCHD3																												Y						Y										
16225	A_23_P154279	CHCHD5			Y				Y	Y	Y	Y	Y	Y				Y		Y	Y						Y															Y				
1347	A_23_P91870	CHCHD6																		Y																										
18908	A_23_P70201	CHD1			Y	Y	Y	Y	Y	Y	Y	Y	Y						Y	Y		Y					Y	Y	Y	Y						Y										
16972	A_23_P51297	CHD1L																									Y																			
17879	A_23_P206068	CHD2				Y																																								
17992	A_23_P53541	CHD4																Y		Y	Y	Y	Y																							
7737	A_23_P385254	CHD5																										Y		Y						Y										
13334	A_23_P91212	CHD6									Y		Y	Y																Y						Y										
10402	A_23_P140238	CHD8	Y	Y	Y	Y		Y	Y	Y	Y	Y	Y	Y						Y	Y	Y					Y	Y	Y	Y						Y								Y		
4623	A_23_P206424	CHD9	Y	Y											Y			Y	Y																											
13123	A_23_P116123	CHEK1	Y	Y																																										
18691	A_23_P109453	CHEK2	Y	Y	Y				Y	Y								Y	Y	Y		Y		Y			Y																			
9886	A_23_P16139	CHERP			Y				Y	Y	Y	Y	Y	Y				Y	Y	Y		Y		Y			Y																			
12579	A_23_P87474	CHFR			Y															Y			Y																							
16123	A_23_P14595	CHGA																					Y																							
14208	A_23_P382584	CHGB			Y																						Y																			
14484	A_23_P137672	CHI3L1			Y	Y		Y	Y	Y	Y	Y	Y	Y					Y	Y							Y	Y	Y	Y						Y										
16199	A_23_P12082	CHI3L2							Y	Y	Y	Y	Y	Y													Y			Y						Y										
12611	A_23_P34888	CHIA			Y	Y		Y	Y	Y																	Y	Y	Y	Y																
20519	A_23_P256019	CHIC2	Y		Y	Y		Y	Y	Y	Y	Y	Y	Y				Y	Y	Y	Y	Y	Y	Y	Y	Y	Y	Y	Y	Y	Y	Y	Y			Y				Y						
2254	A_23_P124742	CHKA																					Y																							
6793	A_23_P136135	CHKA																									Y																			
13067	A_23_P22542	CHM			Y	Y		Y		Y										Y		Y					Y	Y	Y	Y						Y										
13477	A_23_P331138	CHM																		Y																										
3275	A_23_P106694	CHMP1A			Y	Y	Y	Y	Y	Y	Y	Y						Y	Y	Y	Y	Y	Y	Y	Y	Y	Y	Y	Y	Y	Y	Y				Y										
21137	A_23_P153236	CHMP2A	Y	Y	Y				Y	Y								Y		Y	Y	Y	Y				Y				Y		Y	Y	Y											
11996	A_23_P43019	CHMP4C																		Y			Y																							
17610	A_23_P432598	CHMP4C							Y	Y																																				
4385	A_23_P170159	CHMP6			Y	Y		Y	Y	Y								Y	Y	Y		Y	Y	Y			Y			Y						Y										
14224	A_23_P10156	CHMP6			Y													Y	Y	Y		Y	Y	Y																						
12228	A_23_P303203	CHMP7				Y												Y		Y		Y	Y																							
10816	A_23_P111564	CHN2				Y																																								
14718	A_23_P68669	CHODL							Y	Y	Y	Y	Y	Y																Y						Y										
15836	A_23_P206606	CHP2				Y																																								
14652	A_23_P131287	CHPF			Y	Y	Y	Y	Y	Y	Y	Y	Y	Y				Y	Y	Y	Y	Y	Y	Y	Y	Y	Y	Y	Y	Y	Y	Y				Y										
14557	A_23_P105571	CHPT1			Y	Y		Y	Y	Y											Y		Y																							
17660	A_23_P123544	CHRAC1			Y	Y		Y	Y	Y								Y	Y	Y	Y	Y	Y	Y	Y	Y	Y				Y															

14969	A_23_P502047	CHRD	Y	Y	Y	Y	Y	Y	Y	Y	Y	Y															Y	Y	Y	Y			Y	Y		Y										
8510	A_23_P145606	CHRM2							Y	Y	Y	Y	Y	Y													Y			Y						Y										
15802	A_23_P401472	CHRM3							Y	Y																																				
7978	A_23_P104845	CHRM4							Y	Y																																				
12965	A_23_P411188	CHRNA10			Y	Y		Y		Y											Y						Y																			
12255	A_23_P253911	CHRNA3																									Y																			
412	A_23_P88691	CHRNA5							Y	Y								Y		Y							Y			Y						Y										
3454	A_23_P157593	CHRNA6																Y			Y																									
12375	A_23_P163390	CHRNA7			Y																						Y																			
18020	A_23_P61508	CHRNA9	Y	Y	Y	Y		Y	Y	Y										Y							Y	Y	Y	Y						Y										
4567	A_23_P207108	CHRNB1			Y													Y				Y					Y																			
1003	A_23_P368624	CHRNB2				Y																	Y																							
13903	A_23_P74641	CHRNB2			Y	Y		Y	Y	Y								Y	Y	Y	Y	Y	Y	Y	Y	Y	Y				Y		Y	Y	Y											
17623	A_23_P77296	CHRNB4																									Y																			
13871	A_23_P430558	CHRND				Y			Y	Y																	Y	Y	Y	Y						Y										
4938	A_23_P98200	CHST1																									Y																			
9101	A_23_P102351	CHST10	Y	Y	Y																						Y			Y						Y										
6099	A_23_P139919	CHST11	Y		Y	Y		Y	Y	Y	Y	Y	Y	Y	Y						Y									Y						Y										
6029	A_23_P80473	CHST13			Y															Y	Y	Y	Y				Y			Y						Y										
4309	A_23_P106532	CHST14																					Y																							
15655	A_23_P40850	CHST2			Y				Y	Y									Y	Y		Y	Y				Y																			
20463	A_23_P35481	CHST3																	Y	Y			Y																							
5302	A_23_P141035	CHST4							Y	Y																				Y						Y										
19343	A_23_P430670	CHST5			Y	Y	Y	Y	Y	Y	Y	Y	Y	Y				Y		Y			Y				Y	Y	Y	Y						Y								Y		
20158	A_23_P206868	CHST5			Y	Y		Y		Y										Y							Y																			
12063	A_23_P319617	CHST7				Y																																								
15116	A_23_P33833	CHST7																		Y	Y	Y	Y																							
2110	A_23_P90273	CHST8			Y															Y							Y																			
19180	A_23_P37484	CHSY1							Y	Y										Y		Y					Y			Y						Y										
1727	A_23_P354297	CHTF18																												Y						Y										
15909	A_23_P37237	CHURC1																									Y																			
3194	A_23_P77360	CIAO1				Y			Y	Y	Y	Y	Y	Y														Y		Y						Y										
3962	A_23_P410059	CIAO1			Y	Y		Y	Y	Y	Y	Y	Y	Y				Y	Y	Y		Y	Y	Y			Y	Y	Y	Y						Y										
13549	A_23_P88781	CIAPIN1				Y			Y	Y										Y		Y	Y				Y																			
21192	A_23_P65890	CIB2			Y				Y	Y								Y				Y								Y						Y										
3491	A_23_P55731	CIC																	Y	Y		Y	Y																							
1538	A_23_P376704	CIDEA			Y															Y							Y																			
13965	A_23_P37359	CIDEB																Y	Y	Y			Y	Y			Y																			
3975	A_23_P92021	CIDEC																		Y																										
6973	A_23_P33384	CIITA	Y		Y	Y		Y	Y	Y								Y		Y							Y																			
2310	A_23_P377318	CILP2																					Y																							
18212	A_23_P108238	CILP2																				Y																								
13559	A_23_P39814	CIR				Y												Y	Y	Y				Y																						
5031	A_23_P54626	CIRH1A							Y	Y	Y	Y	Y	Y							Y																									
9667	A_23_P35467	CISD1																									Y																			
11153	A_23_P69695	CISD2	Y	Y	Y	Y		Y		Y	Y	Y	Y	Y																Y						Y										
19097	A_23_P144096	CISH	Y	Y	Y	Y		Y	Y	Y								Y									Y	Y	Y	Y						Y										
4469	A_23_P204496	CIT			Y		Y		Y	Y	Y	Y															Y			Y						Y										
349	A_23_P73517	CITED1	Y	Y	Y	Y		Y	Y	Y	Y	Y	Y	Y													Y	Y	Y	Y						Y								Y		
7521	A_23_P306804	CITED4							Y	Y										Y		Y																								
12305	A_23_P74155	CITED4							Y	Y																																				
10524	A_23_P32224	CIZ1			Y	Y	Y	Y	Y	Y	Y	Y	Y	Y				Y	Y	Y	Y	Y	Y	Y	Y	Y	Y				Y															
15858	A_23_P48058	CKAP4			Y	Y	Y	Y		Y	Y	Y						Y	Y	Y	Y	Y	Y	Y	Y	Y	Y				Y															
7616	A_23_P25674	CKB			Y															Y	Y	Y	Y																							
16064	A_23_P206396	CKLF	Y	Y	Y	Y	Y	Y	Y	Y	Y	Y	Y	Y													Y	Y	Y	Y						Y										
740	A_23_P50247	CKM			Y				Y	Y													Y				Y																			
3287	A_23_P163227	CKMT1A																Y																												
7334	A_23_P144778	CKMT2																	Y																											
16153	A_23_P45917	CKS1B			Y	Y		Y		Y								Y	Y	Y		Y		Y			Y	Y	Y	Y																
7010	A_23_P71727	CKS2																		Y		Y								Y						Y										
19204	A_23_P44295	CLASP2	Y	Y	Y	Y		Y	Y	Y	Y	Y	Y	Y				Y	Y	Y		Y	Y	Y			Y	Y	Y	Y						Y										
8944	A_23_P101684	CLC				Y			Y	Y																		Y																		
10313	A_23_P51217	CLCA1			Y	Y		Y		Y																	Y																			
6049	A_23_P397248	CLCA2			Y	Y		Y	Y	Y	Y	Y	Y	Y																																
9670	A_23_P62620	CLCA2			Y	Y		Y	Y	Y	Y	Y	Y	Y																Y						Y								Y		
13494	A_23_P34382	CLCA3							Y	Y																				Y						Y										
5232	A_23_P74809	CLCC1				Y														Y		Y																								
19683	A_23_P59772	CLCN1																									Y																			
22149	A_23_P124476	CLCN3			Y				Y	Y									Y	Y		Y					Y																			
16875	A_23_P217748	CLCN4																												Y						Y										
3045	A_23_P96340	CLCN5			Y													Y	Y	Y				Y			Y																			
14310	A_23_P369209	CLCN5																Y									Y			Y						Y										
15661	A_23_P200361	CLCN6																					Y				Y			Y						Y										
9873	A_23_P129334	CLCN7			Y	Y	Y	Y	Y	Y	Y	Y						Y	Y	Y	Y	Y	Y	Y	Y	Y	Y			Y	Y	Y	Y			Y								Y		
6424	A_23_P57779	CLDN1																Y	Y	Y			Y	Y																						
1126	A_23_P29800	CLDN11							Y	Y																																				
1486	A_23_P405088	CLDN11			Y	Y		Y	Y	Y																		Y																		

3467	A_23_P157268	CLDN12	Y		Y	Y	Y	Y		Y	Y	Y							Y	Y	Y	Y	Y				Y	Y	Y	Y													
3575	A_23_P412515	CLDN12																					Y																						
13332	A_23_P91512	CLDN14			Y	Y		Y		Y																																		
4008	A_23_P8783	CLDN15			Y	Y	Y	Y	Y	Y	Y	Y						Y	Y	Y	Y	Y	Y	Y	Y	Y	Y			Y	Y	Y				Y										
8267	A_23_P336693	CLDN18																										Y																			
8981	A_23_P6986	CLDN18			Y				Y	Y																	Y	Y	Y	Y							Y										
11114	A_23_P394947	CLDN2			Y	Y		Y		Y																	Y																			
21123	A_23_P125562	CLDN2			Y																							Y		Y							Y										
10930	A_23_P134854	CLDN23				Y																																								
8985	A_23_P71017	CLDN3																		Y											Y							Y										
12621	A_23_P6321	CLDN5																		Y	Y	Y			Y	Y																					
6428	A_23_P37942	CLDN6			Y																						Y			Y							Y										
3221	A_23_P164283	CLDN7																													Y							Y										
773	A_23_P132063	CLDN8			Y	Y		Y	Y	Y	Y	Y	Y	Y													Y																			
5991	A_23_P427012	CLDN8			Y																																								
16003	A_23_P54811	CLDN9			Y																																								
16428	A_23_P351138	CLDN9	Y	Y	Y				Y	Y								Y									Y			Y							Y										
20707	A_23_P141508	CLEC10A			Y														Y	Y							Y																			
15760	A_23_P153487	CLEC11A			Y	Y		Y		Y									Y	Y	Y	Y																								
18001	A_23_P204689	CLEC2D																				Y																							
1377	A_23_P42924	CLEC2L				Y																																								
13931	A_23_P26404	CLEC3A							Y	Y									Y	Y																										
11312	A_23_P69497	CLEC3B																					Y							Y							Y										
16429	A_23_P48029	CLEC4A	Y	Y	Y																																								
10091	A_23_P2616	CLEC4C			Y															Y							Y	Y	Y	Y															
15074	A_23_P25235	CLEC4D	Y	Y	Y	Y		Y	Y	Y									Y								Y	Y	Y	Y							Y										
6979	A_23_P162439	CLEC4E	Y	Y	Y		Y				Y															Y																			
17701	A_23_P380990	CLEC4F																			Y																									
21929	A_23_P153390	CLEC4G			Y																									Y							Y										
17291	A_23_P208482	CLEC4M																		Y	Y	Y				Y			Y																			
11006	A_23_P71165	CLEC5A																		Y			Y						Y																		
13372	A_23_P321984	CLECL1							Y	Y																																				
555	A_23_P18684	CLGN			Y	Y	Y	Y	Y	Y	Y	Y	Y	Y						Y							Y	Y	Y	Y	Y	Y	Y	Y	Y	Y										
11088	A_23_P30888	CLIC1			Y	Y	Y	Y	Y	Y	Y	Y	Y	Y				Y	Y	Y	Y	Y	Y	Y	Y	Y	Y	Y	Y	Y	Y	Y				Y										
5973	A_23_P254654	CLIC3			Y	Y		Y	Y	Y	Y	Y	Y	Y						Y							Y	Y	Y	Y							Y										
3938	A_23_P259189	CLIC4			Y						Y		Y	Y													Y			Y							Y										
15889	A_23_P416774	CLIC5			Y														Y	Y			Y				Y																			
19923	A_23_P132088	CLIC6			Y	Y		Y		Y								Y	Y	Y		Y		Y			Y																			
21062	A_23_P385067	CLIC6	Y	Y	Y	Y	Y	Y		Y	Y	Y						Y	Y	Y		Y		Y			Y														Y				
1584	A_23_P25003	CLIP1			Y	Y		Y		Y									Y				Y				Y																			
20381	A_23_P215479	CLIP2			Y	Y	Y	Y	Y	Y	Y	Y	Y	Y				Y	Y	Y	Y	Y	Y	Y	Y	Y	Y			Y	Y	Y				Y										
8375	A_23_P16817	CLK1			Y	Y		Y		Y								Y	Y	Y		Y	Y	Y																						
873	A_23_P85879	CLK2			Y																						Y	Y	Y	Y															
11946	A_23_P124678	CLK3				Y														Y	Y	Y	Y	Y																						
12534	A_23_P99974	CLK3																		Y	Y	Y	Y	Y		Y																					
20688	A_23_P10322	CLK3																		Y	Y	Y	Y	Y		Y							Y		Y	Y	Y											
9006	A_23_P61674	CLK4			Y	Y		Y	Y	Y								Y	Y	Y		Y		Y				Y																	
18642	A_23_P25709	CLMN			Y				Y	Y														Y																						
19515	A_23_P88971	CLN3			Y	Y	Y	Y	Y	Y	Y	Y						Y	Y	Y		Y	Y	Y			Y			Y			Y			Y										
2033	A_23_P117286	CLN5			Y	Y		Y		Y								Y	Y	Y		Y		Y			Y																			
22311	A_23_P117794	CLN6			Y	Y		Y		Y								Y	Y	Y		Y	Y	Y			Y	Y	Y	Y															
15672	A_23_P253734	CLN8																					Y																						
9978	A_23_P127995	CLNS1A			Y														Y		Y					Y																			
16894	A_23_P419038	CLOCK			Y	Y	Y	Y	Y	Y	Y	Y								Y								Y		Y							Y										
15476	A_23_P75978	CLPB			Y								Y						Y							Y	Y	Y	Y															
19732	A_23_P4754	CLPP			Y	Y		Y		Y																	Y	Y	Y	Y															
9251	A_23_P8142	CLPS																										Y		Y							Y										
11817	A_23_P50241	CLPTM1			Y	Y		Y		Y								Y	Y	Y		Y		Y			Y																			
8892	A_23_P110504	CLPTM1L			Y	Y		Y		Y									Y	Y		Y					Y			Y							Y										
6647	A_23_P140511	CLPX			Y				Y	Y			Y													Y																			
13104	A_23_P364567	CLRN1																													Y							Y										
14758	A_23_P80523	CLRN1																				Y	Y																						
6318	A_23_P147599	CLSTN1																		Y		Y			Y																						
377	A_23_P212608	CLSTN2			Y	Y		Y	Y	Y													Y				Y	Y	Y	Y							Y										
17885	A_23_P219144	CLTA			Y	Y		Y	Y	Y										Y							Y																			
2099	A_23_P252677	CLTB			Y	Y	Y	Y	Y	Y	Y	Y						Y	Y	Y		Y	Y	Y			Y	Y	Y	Y							Y										
7717	A_23_P60610	CLTB			Y	Y	Y	Y	Y	Y	Y	Y	Y	Y				Y	Y	Y		Y	Y	Y			Y	Y	Y	Y							Y										
10741	A_23_P43742	CLTB			Y	Y	Y	Y	Y	Y	Y	Y						Y	Y	Y		Y	Y	Y			Y	Y	Y	Y							Y										
14807	A_23_P118543	CLTC							Y	Y																																				
16149	A_23_P132166	CLTCL1																					Y																								
15697	A_23_P215918	CLU																					Y		Y																						
16042	A_23_P77714	CLUAP1			Y	Y	Y	Y	Y	Y	Y	Y	Y	Y				Y	Y	Y				Y			Y	Y	Y	Y							Y										
20134	A_23_P55568	CLUL1			Y	Y		Y	Y	Y									Y	Y			Y				Y																			
5012	A_23_P376586	CLYBL			Y	Y	Y	Y	Y	Y	Y	Y	Y	Y				Y		Y			Y				Y	Y	Y	Y															
7697	A_23_P151778	CMA1																			Y																									
8477	A_23_P144668	CMBL			Y				Y	Y	Y	Y	Y	Y						Y							Y			Y							Y										
17292	A_23_P100263	CMIP			Y	Y		Y		Y										Y		Y	Y				Y																			

8100	A_23_P105461	CMKLR1																		Y			Y																							
9589	A_23_P115366	CMPK1			Y																						Y																		
6426	A_23_P106663	CMTM1			Y		Y				Y																Y																		
15131	A_23_P152234	CMTM2	Y	Y																																									
705	A_23_P88865	CMTM3																	Y																										
12279	A_23_P391980	CMTM4			Y																						Y																		
8814	A_23_P106042	CMTM5							Y	Y	Y	Y	Y	Y					Y											Y						Y										
6180	A_23_P256413	CMTM7	Y						Y	Y	Y	Y	Y	Y						Y			Y							Y						Y										
7825	A_23_P124946	CMYA5			Y	Y	Y	Y	Y	Y	Y	Y	Y	Y				Y	Y	Y		Y		Y			Y	Y	Y	Y						Y										
3768	A_23_P84651	CNBP				Y														Y			Y																							
1686	A_23_P9869	CNDP1			Y	Y		Y	Y	Y																	Y	Y	Y	Y						Y										
7204	A_23_P27473	CNFN				Y																						Y																	
4638	A_23_P92535	CNGA1			Y														Y	Y								Y																	
9513	A_23_P11163	CNGA2																										Y																	
17186	A_23_P2006	CNGA4			Y	Y		Y		Y																																			
15177	A_23_P37718	CNGB1			Y	Y	Y	Y	Y	Y	Y	Y	Y	Y					Y	Y			Y				Y			Y						Y								Y		
7486	A_23_P384044	CNIH3																	Y	Y																									
20819	A_23_P23341	CNKSR1																										Y																	
2558	A_23_P72097	CNKSR2							Y	Y																																			
242	A_23_P16239	CNN1																					Y																						
16878	A_23_P125233	CNN1																					Y																						
15750	A_23_P16673	CNN2			Y	Y		Y		Y										Y		Y	Y				Y																		
22178	A_23_P138168	CNN3							Y	Y																																			
16819	A_23_P46947	CNNM1																Y	Y	Y		Y		Y																					
1989	A_23_P119923	CNNM4			Y																						Y																		
6690	A_23_P69675	CNO			Y																																								
6975	A_23_P162336	CNOT2			Y	Y		Y	Y	Y									Y	Y		Y					Y	Y	Y	Y						Y										
18799	A_23_P38987	CNOT3			Y													Y	Y	Y		Y		Y			Y																		
12345	A_23_P300781	CNOT4			Y	Y		Y	Y	Y										Y	Y	Y					Y	Y	Y	Y															
13635	A_23_P167419	CNOT6			Y	Y	Y	Y	Y	Y	Y	Y	Y	Y						Y		Y	Y				Y	Y	Y	Y												Y				
14888	A_23_P216310	CNOT7																		Y																									
1545	A_23_P110843	CNOT8			Y	Y	Y	Y	Y	Y	Y	Y	Y	Y						Y			Y				Y	Y	Y	Y						Y										
20869	A_23_P21838	CNP			Y	Y		Y		Y								Y	Y		Y	Y					Y																		
6192	A_23_P19352	CNPY3			Y	Y		Y	Y	Y								Y	Y	Y	Y	Y	Y	Y	Y	Y	Y			Y	Y	Y	Y			Y										
4715	A_23_P310931	CNR2			Y	Y	Y	Y		Y	Y	Y							Y	Y			Y				Y	Y	Y	Y						Y										
15789	A_23_P329357	CNRIP1																		Y			Y																						
2982	A_23_P55196	CNTD1			Y	Y		Y	Y	Y	Y	Y	Y	Y									Y							Y						Y										
3326	A_23_P78903	CNTD2			Y	Y		Y	Y	Y	Y	Y	Y	Y				Y	Y	Y				Y						Y						Y										
9407	A_23_P9405	CNTFR			Y	Y		Y	Y	Y	Y	Y	Y	Y				Y	Y	Y			Y	Y			Y			Y						Y										
10879	A_23_P250173	CNTN3			Y	Y	Y	Y		Y	Y	Y						Y	Y																										
10317	A_23_P411112	CNTNAP1																		Y																									
5948	A_23_P84399	CNTNAP2							Y	Y																																			
19666	A_23_P9135	CNTNAP3							Y	Y																	Y			Y						Y										
17559	A_23_P4142	COASY							Y	Y																				Y						Y										
835	A_23_P79289	COBLL1																					Y																						
12418	A_23_P148150	COBRA1			Y	Y		Y	Y	Y								Y	Y	Y		Y	Y	Y			Y			Y						Y										
22233	A_23_P135402	COBRA1			Y	Y	Y	Y	Y	Y	Y	Y						Y	Y	Y	Y	Y	Y	Y	Y	Y	Y	Y	Y	Y	Y	Y	Y			Y										
18813	A_23_P160807	COG2	Y	Y	Y	Y		Y	Y	Y	Y	Y	Y	Y				Y	Y	Y	Y		Y	Y	Y		Y			Y						Y										
18787	A_23_P2692	COG3			Y		Y				Y							Y	Y	Y		Y		Y			Y	Y	Y	Y						Y										
8092	A_23_P134614	COG5																		Y			Y																						
21102	A_23_P53866	COG6			Y	Y		Y		Y								Y	Y	Y		Y		Y																					
22413	A_23_P11806	COL11A1																	Y			Y																							
302	A_23_P214168	COL12A1			Y	Y		Y		Y									Y		Y	Y						Y																	
14455	A_23_P216361	COL14A1																										Y		Y						Y										
6601	A_23_P160318	COL16A1																									Y	Y	Y	Y						Y										
1438	A_23_P255244	COL1A2																			Y	Y						Y																	
11411	A_23_P74701	COL24A1			Y	Y		Y		Y								Y	Y	Y			Y	Y						Y						Y										
3354	A_23_P158096	COL27A1			Y	Y	Y	Y	Y	Y	Y	Y	Y	Y				Y	Y	Y	Y	Y	Y	Y	Y	Y	Y			Y	Y	Y				Y										
4770	A_23_P142527	COL3A1							Y	Y																																			
15691	A_23_P205031	COL4A2							Y	Y																				Y						Y										
18546	A_23_P61748	COL4A3BP				Y			Y	Y																		Y		Y						Y										
21570	A_23_P254522	COL4A4	Y		Y	Y		Y	Y	Y	Y	Y	Y	Y						Y			Y				Y	Y	Y	Y						Y										
5969	A_23_P45365	COL4A5	Y	Y	Y	Y	Y	Y	Y	Y	Y	Y	Y	Y				Y	Y	Y	Y	Y	Y	Y	Y	Y	Y	Y	Y	Y	Y	Y				Y										
15688	A_23_P217379	COL4A6	Y	Y	Y	Y	Y	Y		Y	Y	Y						Y		Y		Y					Y			Y						Y										
1267	A_23_P83818	COL5A1																		Y		Y																							
9272	A_23_P10391	COL5A2				Y													Y		Y	Y						Y																	
22529	A_23_P33196	COL5A2																	Y		Y	Y						Y																	
5346	A_23_P131614	COL6A3																	Y									Y																	
10462	A_23_P144067	COL7A1	Y	Y	Y													Y	Y								Y	Y	Y	Y																
5350	A_23_P80436	COL8A1				Y																																								
15839	A_23_P137597	COL8A2																	Y			Y						Y																	
13345	A_23_P30593	COL9A1																									Y																		
15332	A_23_P120131	COLEC11																		Y	Y		Y																						
3811	A_23_P27306	COLEC12							Y	Y								Y	Y	Y	Y			Y			Y			Y						Y										
21440	A_23_P212123	COLQ									Y		Y	Y													Y			Y						Y										
7118	A_23_P252403	COMMD10			Y				Y	Y	Y	Y	Y	Y						Y	Y		Y				Y			Y						Y										
8128	A_23_P40782	COMMD2			Y				Y	Y																				Y						Y										

14429	A_23_P20255	COMMD5			Y	Y		Y		Y										Y							Y			Y						Y										
22047	A_23_P420428	COMMD7			Y	Y	Y	Y		Y	Y	Y								Y	Y	Y					Y	Y	Y	Y																
20831	A_23_P44257	COMMD8																	Y																											
3588	A_23_P87323	COMMD9			Y															Y		Y					Y																			
7201	A_23_P98710	COMMD9																		Y																										
2523	A_23_P251680	COMT			Y	Y		Y	Y	Y	Y	Y	Y	Y				Y	Y	Y		Y	Y	Y			Y	Y	Y	Y						Y										
11179	A_23_P115862	COMTD1			Y				Y	Y																																				
5609	A_23_P187	COPA			Y														Y	Y		Y					Y																			
8817	A_23_P138895	COPB1			Y	Y	Y	Y	Y	Y	Y	Y							Y	Y		Y					Y	Y	Y	Y						Y										
15994	A_23_P91891	COPB2				Y																	Y				Y																			
8411	A_23_P209089	COPE			Y	Y	Y	Y	Y	Y	Y	Y	Y	Y				Y	Y	Y	Y	Y	Y	Y	Y	Y	Y	Y	Y	Y	Y	Y				Y				Y						
19897	A_23_P44617	COPG			Y	Y		Y		Y								Y		Y	Y	Y					Y																			
20334	A_23_P61281	COPG2			Y				Y	Y										Y																										
13335	A_23_P26021	COPS2																		Y																										
14191	A_23_P43779	COPS4																		Y			Y																							
12774	A_23_P8432	COPS6																Y	Y	Y		Y		Y																						
15120	A_23_P2473	COPS7A			Y															Y	Y	Y	Y																							
18847	A_23_P79725	COPS7B			Y	Y	Y	Y		Y	Y	Y	Y	Y				Y	Y	Y	Y	Y	Y	Y	Y	Y	Y				Y										Y					
5466	A_23_P116802	COPZ1	Y	Y	Y	Y		Y	Y	Y	Y	Y	Y	Y						Y			Y				Y			Y						Y										
5733	A_23_P101093	COPZ2																					Y																							
10746	A_23_P354798	COQ10A	Y	Y	Y	Y	Y	Y	Y	Y	Y	Y						Y		Y		Y	Y				Y			Y	Y	Y	Y	Y	Y	Y										
13088	A_23_P124733	COQ2			Y	Y		Y		Y																	Y	Y	Y	Y						Y										
16675	A_23_P113482	COQ2			Y	Y		Y	Y	Y																	Y	Y	Y	Y						Y										
21582	A_23_P112493	COQ4			Y	Y		Y	Y	Y																				Y						Y										
9911	A_23_P105625	COQ5			Y	Y		Y	Y	Y	Y	Y	Y	Y				Y		Y	Y						Y																			
1832	A_23_P65577	COQ6	Y	Y	Y		Y		Y	Y	Y	Y	Y	Y	Y			Y		Y	Y	Y	Y				Y			Y			Y			Y										
383	A_23_P152322	COQ7	Y		Y																						Y																			
15371	A_23_P14928	COQ9				Y																	Y					Y																		
1833	A_23_P106761	CORO1A	Y		Y	Y	Y	Y	Y	Y	Y	Y						Y	Y	Y	Y	Y	Y	Y	Y	Y	Y				Y		Y	Y	Y											
11038	A_23_P104589	CORO1B			Y	Y	Y	Y	Y	Y	Y	Y	Y	Y				Y	Y	Y	Y	Y	Y	Y	Y	Y	Y	Y	Y	Y	Y	Y				Y										
9393	A_23_P53456	CORO1C	Y	Y		Y			Y	Y					Y													Y		Y						Y										
9664	A_23_P146444	CORO2A			Y	Y		Y	Y	Y	Y	Y	Y	Y						Y		Y																								
14773	A_23_P117694	CORO2B			Y															Y							Y																			
9318	A_23_P107322	CORO6			Y													Y									Y			Y						Y										
1551	A_23_P38046	CORO7			Y	Y		Y	Y	Y								Y	Y	Y	Y	Y	Y	Y	Y	Y	Y				Y															
7531	A_23_P149413	CORT							Y	Y																				Y						Y										
4020	A_23_P83620	COTL1			Y	Y		Y	Y	Y	Y	Y	Y	Y				Y	Y	Y	Y	Y	Y	Y	Y	Y	Y			Y	Y	Y				Y										
21411	A_23_P301922	COX1			Y	Y	Y	Y	Y	Y	Y	Y	Y	Y													Y	Y	Y	Y						Y								Y		
17653	A_23_P55123	COX10			Y	Y		Y		Y	Y	Y	Y	Y				Y	Y	Y				Y			Y																	Y		
5767	A_23_P202374	COX15																										Y																		
11467	A_23_P61123	COX16			Y																																									
6354	A_23_P428388	COX17			Y																		Y																							
11972	A_23_P321485	COX18																					Y																							
1923	A_23_P141028	COX4I1			Y	Y	Y	Y	Y	Y	Y	Y	Y	Y				Y		Y	Y		Y				Y	Y	Y	Y						Y										
9231	A_23_P106859	COX4NB	Y	Y	Y	Y	Y	Y	Y	Y	Y	Y	Y	Y					Y	Y							Y	Y	Y	Y						Y										
6800	A_23_P84259	COX5A	Y	Y	Y	Y		Y		Y																		Y																		
11658	A_23_P44586	COX5A	Y	Y	Y	Y		Y		Y																		Y																		
11590	A_23_P401524	COX6A2			Y	Y		Y	Y	Y										Y		Y	Y				Y																			
11023	A_23_P108244	COX6B1							Y	Y																																				
13361	A_23_P8900	COX6C			Y				Y	Y									Y	Y			Y				Y																			
11354	A_23_P67661	COX7A1			Y	Y		Y	Y	Y																	Y			Y						Y										
4303	A_23_P110811	COX7C			Y				Y	Y								Y		Y	Y	Y	Y				Y			Y						Y										
949	A_23_P69084	CP				Y																																								
8750	A_23_P414793	CP				Y			Y	Y								Y		Y								Y																		
16108	A_23_P26495	CP110	Y		Y	Y	Y	Y	Y	Y	Y	Y	Y	Y						Y			Y				Y	Y	Y	Y						Y										
8794	A_23_P18017	CPA3							Y	Y					Y																															
15794	A_23_P19754	CPA4			Y																						Y			Y						Y										
17132	A_23_P215109	CPA5																				Y																								
18517	A_23_P216291	CPA6							Y	Y									Y																											
17964	A_23_P6615	CPB1			Y	Y		Y	Y	Y	Y	Y	Y	Y						Y							Y	Y	Y	Y						Y										
8295	A_23_P25502	CPB2	Y	Y	Y															Y		Y					Y			Y						Y										
15060	A_23_P207837	CPD							Y	Y																				Y						Y										
22302	A_23_P259442	CPE																									Y																			
19111	A_23_P106322	CPEB1			Y																																									
5898	A_23_P46812	CPEB3			Y	Y		Y	Y	Y	Y	Y	Y	Y						Y		Y						Y		Y						Y										
3123	A_23_P251937	CPEB4			Y	Y		Y		Y									Y	Y		Y					Y			Y						Y										
5823	A_23_P29983	CPN2							Y	Y								Y	Y	Y		Y		Y						Y						Y										
8122	A_23_P121030	CPN2			Y																																									
20168	A_23_P218720	CPNE1																		Y	Y	Y																								
12104	A_23_P37988	CPNE2			Y	Y	Y	Y	Y	Y	Y	Y	Y	Y				Y	Y	Y	Y	Y	Y	Y	Y	Y	Y			Y	Y	Y				Y				Y						
18459	A_23_P94370	CPNE3			Y																						Y																			
14976	A_23_P259811	CPNE4			Y													Y	Y																											
16829	A_23_P327551	CPNE4																												Y						Y										
4018	A_23_P122606	CPNE5			Y	Y		Y		Y																	Y	Y	Y	Y																
9760	A_23_P360804	CPNE5	Y		Y		Y		Y	Y	Y	Y	Y	Y					Y	Y							Y																			
14394	A_23_P151596	CPNE6			Y	Y		Y	Y	Y																	Y	Y	Y	Y						Y										
20282	A_23_P26424	CPNE7									Y		Y	Y																Y						Y										

15340	A_23_P87556	CPNE8																										Y		Y						Y										
20972	A_23_P57829	CPNE9			Y	Y	Y	Y	Y	Y	Y	Y	Y	Y				Y	Y								Y			Y						Y								Y		
20464	A_23_P102172	CPO																	Y																											
C10362.2	A_23_P5300	CPS1			Y				Y	Y	Y	Y	Y	Y				Y	Y	Y				Y			Y																			
12128	A_23_P169108	CPSF1			Y	Y	Y	Y	Y	Y	Y	Y						Y	Y	Y	Y	Y	Y	Y	Y	Y	Y				Y															
8152	A_23_P28683	CPSF3			Y															Y																										
7016	A_23_P35030	CPSF3L			Y	Y		Y		Y									Y	Y		Y	Y																							
13875	A_23_P42997	CPSF4			Y	Y		Y		Y									Y	Y			Y				Y																			
4690	A_23_P204039	CPSF6			Y	Y		Y		Y										Y																										
6508	A_23_P104563	CPT1A																Y		Y										Y						Y										
9963	A_23_P218811	CPT1B	Y	Y	Y	Y		Y	Y	Y																	Y	Y	Y	Y																
1920	A_23_P16283	CPT1C																		Y			Y																							
12289	A_23_P145743	CPVL	Y		Y										Y							Y								Y						Y										
16585	A_23_P6066	CPXM1																		Y			Y																							
5201	A_23_P58251	CPZ																		Y	Y	Y	Y																							
5040	A_23_P61158	CR1	Y																								Y																			
7518	A_23_P83961	CR1			Y															Y							Y																			
15351	A_23_P63551	CR1			Y																																									
15366	A_23_P124542	CR2	Y		Y																						Y			Y						Y										
21763	A_23_P117882	CRABP1																Y	Y			Y					Y																			
19278	A_23_P405064	CRABP2			Y																						Y																			
10480	A_23_P139604	CRADD																	Y																											
17963	A_23_P32196	CRAT			Y				Y	Y																																				
15569	A_23_P12357	CRB1																									Y																			
4300	A_23_P250930	CRBN			Y																																									
12125	A_23_P12155	CRCT1																									Y																			
5757	A_23_P79231	CREB1				Y																						Y																		
13415	A_23_P150407	CREB3L1																					Y																							
4655	A_23_P215121	CREB3L2			Y	Y	Y	Y	Y	Y	Y	Y							Y			Y					Y	Y	Y	Y						Y										
10309	A_23_P108082	CREB3L3	Y	Y	Y																						Y			Y						Y										
7182	A_23_P63232	CREB3L4	Y	Y	Y				Y	Y	Y	Y	Y	Y																Y						Y										
18032	A_23_P157117	CREB5			Y	Y		Y		Y										Y																										
16499	A_23_P163850	CREBBP			Y																																									
18653	A_23_P85742	CREG1	Y	Y	Y	Y		Y	Y	Y					Y			Y	Y	Y		Y		Y			Y	Y	Y	Y						Y										
1038	A_23_P33465	CRELD2			Y	Y	Y	Y	Y	Y	Y	Y						Y	Y	Y		Y	Y	Y			Y	Y	Y	Y						Y										
7130	A_23_P125098	CRELD2			Y	Y		Y	Y	Y								Y	Y	Y		Y	Y	Y			Y	Y	Y	Y																
8240	A_23_P31755	CRH			Y				Y	Y																	Y			Y						Y										
22113	A_23_P30567	CRHBP																			Y		Y																							
14075	A_23_P414884	CRHR1			Y	Y		Y		Y									Y	Y		Y	Y				Y																			
20838	A_23_P100974	CRHR1																									Y																			
3750	A_23_P51105	CRIM1	Y	Y	Y																						Y			Y						Y										
8628	A_23_P33285	CRIP1			Y				Y	Y	Y	Y	Y	Y													Y			Y						Y										
17850	A_23_P44674	CRIP1			Y	Y		Y	Y	Y	Y	Y	Y	Y													Y			Y						Y										
7127	A_23_P431330	CRIPAK	Y	Y	Y	Y	Y	Y	Y	Y	Y	Y	Y	Y				Y	Y	Y		Y	Y	Y			Y	Y	Y	Y						Y										
15996	A_23_P17219	CRIPT																												Y						Y										
18877	A_23_P419760	CRISP3				Y			Y	Y																																				
22052	A_23_P59958	CRISPLD1			Y	Y		Y	Y	Y																																				
4615	A_23_P83556	CRK																												Y						Y										
17977	A_23_P40535	CRKL																Y		Y	Y	Y	Y																							
21178	A_23_P56197	CRLF1																					Y																							
2167	A_23_P79951	CRLS1			Y	Y		Y	Y	Y	Y	Y	Y	Y						Y	Y		Y				Y			Y						Y										
6520	A_23_P131967	CRNKL1			Y	Y		Y	Y	Y	Y	Y	Y	Y						Y		Y	Y				Y	Y	Y	Y						Y										
7207	A_23_P207493	CROP			Y	Y	Y	Y	Y	Y	Y	Y	Y	Y						Y							Y	Y	Y	Y						Y										
13237	A_23_P168669	CROT			Y	Y		Y	Y	Y	Y	Y	Y	Y					Y	Y	Y	Y	Y				Y			Y	Y	Y	Y	Y	Y	Y										
6336	A_23_P51245	CRP	Y	Y																																										
4150	A_23_P50773	CRTC1			Y														Y	Y	Y	Y	Y																							
21834	A_23_P77228	CRTC3																	Y	Y																										
17707	A_23_P127394	CRY2			Y	Y		Y	Y	Y								Y									Y	Y	Y	Y						Y										
2593	A_23_P132147	CRYAA																				Y						Y																		
1345	A_23_P75589	CRYAB							Y	Y	Y	Y	Y	Y													Y			Y						Y										
13774	A_23_P4254	CRYBA1																Y		Y																										
180	A_23_P103034	CRYBA4																									Y	Y	Y	Y																
15807	A_23_P143621	CRYBB1			Y													Y		Y		Y					Y			Y						Y										
20293	A_23_P40574	CRYBB2																		Y																										
18478	A_23_P29177	CRYBB3			Y				Y	Y																		Y																		
14244	A_23_P165380	CRYGA				Y			Y	Y																				Y						Y										
276	A_23_P320705	CRYGD			Y																						Y																			
21309	A_23_P131215	CRYGD			Y						Y		Y	Y					Y								Y	Y	Y	Y						Y										
1252	A_23_P310439	CRYGN				Y													Y									Y																		
18235	A_23_P132736	CRYGS			Y		Y		Y	Y	Y	Y						Y	Y	Y		Y		Y			Y															Y				
7766	A_23_P401732	CRYL1							Y	Y																																				
10541	A_23_P114662	CRYZ			Y	Y	Y	Y	Y	Y	Y	Y						Y	Y	Y		Y	Y	Y			Y	Y	Y	Y						Y										
6611	A_23_P218733	CRYZL1			Y	Y		Y		Y									Y	Y			Y				Y																			
3580	A_23_P105738	CSAD				Y			Y	Y																		Y		Y						Y										
18529	A_23_P159637	CSAG1							Y	Y																																				
13778	A_23_P25229	CSDA				Y					Y		Y	Y						Y			Y				Y	Y	Y	Y																
1866	A_23_P407012	CSF1			Y	Y		Y		Y									Y	Y		Y	Y				Y	Y	Y	Y						Y										

9793	A_23_P73397	CSF1			Y															Y			Y																							
9639	A_23_P110791	CSF1R			Y				Y	Y	Y	Y	Y	Y													Y	Y	Y	Y						Y										
1524	A_23_P133408	CSF2			Y				Y	Y																	Y			Y						Y										
13171	A_23_P136174	CSF2RA	Y		Y															Y																										
4226	A_23_P120899	CSF2RB			Y	Y		Y	Y	Y																	Y																			
17254	A_23_P55054	CSF3							Y	Y																																				
2861	A_23_P126215	CSF3R			Y														Y	Y																										
1736	A_23_P149887	CSGALNACT2			Y	Y		Y	Y	Y									Y								Y	Y	Y	Y																
9554	A_23_P207180	CSHL1			Y																						Y																			
11577	A_23_P152020	CSK			Y	Y	Y	Y	Y	Y	Y	Y	Y	Y				Y	Y	Y		Y	Y	Y			Y																			
11299	A_23_P31776	CSMD1			Y																	Y																								
13223	A_23_P255203	CSMD1			Y															Y		Y	Y				Y																			
6255	A_23_P200374	CSMD2																				Y																								
18431	A_23_P252882	CSN3							Y	Y			Y														Y																			
7520	A_23_P213551	CSNK1A1			Y	Y		Y	Y	Y													Y				Y			Y						Y										
4776	A_23_P128653	CSNK1A1L	Y	Y	Y	Y		Y	Y	Y																	Y	Y	Y	Y						Y										
4307	A_23_P207896	CSNK1D			Y	Y	Y	Y	Y	Y	Y	Y						Y	Y	Y	Y	Y	Y	Y	Y	Y	Y				Y											Y				
8927	A_23_P40670	CSNK1E							Y	Y																				Y						Y										
18549	A_23_P56143	CSNK1G2			Y	Y	Y	Y	Y	Y	Y	Y								Y	Y	Y	Y				Y																			
3309	A_23_P213602	CSNK1G3			Y	Y		Y		Y								Y	Y	Y		Y		Y			Y	Y	Y	Y																
20102	A_23_P502575	CSNK2A1			Y	Y		Y		Y								Y	Y	Y	Y	Y	Y	Y	Y	Y	Y				Y															
2524	A_23_P14915	CSNK2A2							Y	Y																				Y						Y										
1244	A_23_P257557	CSNK2B			Y				Y	Y										Y							Y	Y	Y	Y						Y										
3946	A_23_P21975	CSPG4							Y	Y													Y																							
18508	A_23_P73873	CSPG4LYP1																					Y																							
22307	A_23_P155219	CSPG5				Y					Y		Y	Y													Y	Y	Y	Y																
9504	A_23_P71537	CSPP1			Y	Y		Y	Y	Y										Y			Y				Y	Y	Y	Y																
869	A_23_P151209	CSRNP2			Y	Y	Y	Y	Y	Y	Y	Y	Y	Y				Y	Y	Y	Y	Y	Y	Y	Y	Y	Y	Y	Y	Y	Y	Y														
15948	A_23_P74359	CSRP1			Y	Y		Y		Y								Y	Y	Y		Y	Y	Y																						
10744	A_23_P44724	CSRP2			Y				Y	Y	Y	Y	Y	Y						Y							Y			Y						Y										
16269	A_23_P501435	CSRP2BP			Y	Y		Y	Y	Y	Y	Y	Y	Y					Y	Y		Y	Y				Y	Y	Y	Y						Y										
2941	A_23_P24469	CSRP3			Y																						Y																			
17001	A_23_P154745	CST3																			Y																									
16768	A_23_P113506	CST5																												Y						Y										
10638	A_23_P68601	CST7	Y	Y	Y		Y		Y	Y	Y	Y								Y																										
10216	A_23_P143365	CST9L			Y	Y		Y	Y	Y	Y	Y	Y	Y						Y		Y	Y				Y	Y	Y	Y						Y						Y				
11323	A_23_P170231	CSTA	Y	Y	Y		Y		Y	Y	Y	Y						Y		Y	Y						Y	Y	Y	Y						Y										
14840	A_23_P41114	CSTA	Y	Y	Y				Y	Y																	Y			Y						Y										
7751	A_23_P154889	CSTB				Y							Y															Y																		
21004	A_23_P148372	CSTF2				Y																						Y																		
3155	A_23_P98310	CSTF3			Y	Y		Y	Y	Y								Y	Y	Y		Y	Y	Y			Y																			
8307	A_23_P132341	CTA-216E10.6																Y				Y																								
5353	A_23_P148541	CTAG1A			Y																																									
4998	A_23_P22693	CTAG2				Y														Y																										
16249	A_23_P63897	CTBP2			Y	Y	Y	Y	Y	Y	Y	Y	Y	Y						Y								Y		Y						Y										
6990	A_23_P201368	CTBS																Y	Y	Y		Y		Y																						
17397	A_23_P34031	CTD-2267G17.3							Y	Y			Y							Y																										
4848	A_23_P101111	CTDP1			Y	Y	Y	Y	Y	Y	Y	Y						Y	Y	Y		Y	Y	Y			Y	Y	Y	Y						Y										
4793	A_23_P28263	CTDSP1			Y													Y		Y		Y					Y																			
946	A_23_P81878	CTDSP2																		Y	Y		Y																							
11287	A_23_P44144	CTDSP2																		Y	Y		Y																							
7539	A_23_P151856	CTDSPL2			Y	Y		Y		Y										Y																										
7098	A_23_P163838	CTF1																												Y						Y										
13313	A_23_P399086	CTF8			Y	Y		Y		Y										Y		Y																								
18807	A_23_P126103	CTH																	Y	Y		Y	Y																							
12056	A_23_P111888	CTHRC1													Y																															
9038	A_23_P102481	CTLA4																					Y																							
933	A_23_P58647	CTNNA1			Y	Y	Y	Y	Y	Y	Y	Y	Y	Y					Y	Y		Y					Y	Y	Y	Y						Y										
12440	A_23_P84736	CTNNA2													Y																															
12604	A_23_P73117	CTNNA3			Y													Y	Y			Y					Y																			
146	A_23_P23586	CTNNBIP1			Y																																									
3138	A_23_P68403	CTNNBL1																		Y	Y	Y																								
4315	A_23_P251316	CTNND1																Y		Y																										
18250	A_23_P110624	CTNND2			Y				Y	Y																	Y																			
2123	A_23_P127676	CTR9			Y	Y		Y	Y	Y	Y	Y	Y	Y				Y	Y	Y		Y	Y	Y			Y	Y	Y	Y						Y										
21692	A_23_P15253	CTRB1																		Y	Y																									
18331	A_23_P152199	CTRL				Y			Y	Y																																				
21847	A_23_P17374	CTSA	Y		Y	Y		Y		Y								Y					Y				Y																			
81	A_23_P215944	CTSB			Y	Y		Y	Y	Y																																				
19946	A_23_P1552	CTSC	Y	Y	Y	Y		Y	Y	Y																		Y		Y						Y										
3712	A_23_P52556	CTSD			Y		Y				Y							Y	Y	Y	Y	Y	Y	Y	Y	Y	Y			Y	Y	Y				Y										
13023	A_23_P10291	CTSE																												Y						Y										
5977	A_23_P24433	CTSF	Y	Y	Y	Y		Y		Y								Y	Y								Y	Y	Y	Y						Y										
3403	A_23_P140384	CTSG				Y														Y			Y																							
19288	A_23_P14774	CTSH			Y				Y	Y																																				
4203	A_23_P94533	CTSL1	Y	Y	Y	Y		Y		Y																		Y		Y						Y										
15611	A_23_P146456	CTSL2				Y			Y	Y																				Y						Y										

14179	A_23_P110175	CTSO																	Y									Y																		
13762	A_23_P46141	CTSS	Y	Y	Y	Y		Y	Y	Y					Y						Y						Y																			
17812	A_23_P13027	CTSW							Y	Y																																				
18393	A_23_P40240	CTSZ							Y	Y																				Y						Y										
6734	A_23_P202823	CTTN																Y																												
20674	A_23_P215744	CTTNBP2			Y	Y	Y	Y		Y	Y	Y	Y	Y														Y		Y						Y										
318	A_23_P318420	CTTNBP2NL																												Y						Y										
18145	A_23_P126953	CTTNBP2NL							Y	Y																				Y						Y										
5110	A_23_P127027	CUBN				Y																																								
9872	A_23_P127088	CUEDC2																		Y		Y	Y							Y						Y										
2015	A_23_P63997	CUGBP1			Y	Y	Y	Y	Y	Y	Y	Y						Y	Y	Y		Y		Y			Y	Y	Y	Y						Y										
21385	A_23_P59657	CUL1							Y	Y																																				
16939	A_23_P209288	CUL3			Y	Y		Y	Y	Y								Y		Y	Y	Y	Y				Y	Y	Y	Y						Y										
15170	A_23_P151471	CUL4A			Y				Y	Y																																				
4826	A_23_P148454	CUL4B																		Y																										
12507	A_23_P203009	CUL5																	Y			Y																								
6004	A_23_P19343	CUL7			Y																		Y				Y																			
3355	A_23_P311740	CUL9			Y				Y	Y	Y	Y	Y	Y				Y				Y								Y						Y										
2354	A_23_P253376	CUX1			Y	Y		Y	Y	Y	Y	Y	Y	Y				Y	Y	Y	Y	Y	Y	Y	Y	Y	Y				Y															
4649	A_23_P2541	CUX2			Y	Y		Y	Y	Y								Y		Y		Y																								
12322	A_23_P98218	CWC15																					Y																							
2407	A_23_P161716	CWF19L2																Y	Y	Y		Y	Y	Y																						
8800	A_23_P37727	CX3CL1			Y	Y		Y		Y																				Y						Y										
11161	A_23_P132401	CX3CR1							Y	Y																	Y																			
14134	A_23_P407565	CX3CR1																		Y																										
8105	A_23_P212905	CXCL1			Y						Y		Y	Y														Y		Y						Y										
15797	A_23_P7144	CXCL1			Y	Y		Y		Y	Y	Y	Y	Y													Y	Y	Y	Y						Y										
13090	A_23_P124884	CXCL10	Y	Y	Y	Y	Y	Y	Y	Y	Y	Y	Y	Y					Y	Y							Y	Y	Y	Y						Y										
19478	A_23_P125278	CXCL11	Y	Y	Y	Y	Y	Y	Y	Y	Y	Y								Y							Y																			
20858	A_23_P202448	CXCL12																					Y																							
14377	A_23_P121692	CXCL13	Y	Y		Y			Y	Y																	Y	Y	Y	Y																
14588	A_23_P213745	CXCL14													Y	Y																														
1490	A_23_P38505	CXCL16			Y	Y		Y	Y	Y										Y			Y					Y		Y						Y										
7251	A_23_P50265	CXCL17			Y																																									
5756	A_23_P315364	CXCL2																												Y						Y										
20267	A_23_P257219	CXCL3			Y				Y	Y																		Y		Y						Y										
17453	A_23_P110204	CXCL5			Y																									Y						Y										
2440	A_23_P18452	CXCL9	Y	Y	Y				Y	Y										Y							Y	Y	Y	Y																
12921	A_23_P114299	CXCR3	Y		Y				Y	Y									Y				Y				Y																			
1469	A_23_P102000	CXCR4													Y																															
2462	A_23_P109913	CXCR6	Y	Y	Y				Y	Y								Y	Y	Y		Y		Y			Y			Y						Y										
3793	A_23_P146997	CXorf15				Y																																								
13952	A_23_P62227	CXorf21	Y	Y	Y		Y		Y	Y	Y	Y									Y						Y																			
3998	A_23_P217785	CXorf26			Y																																									
16240	A_23_P346389	CXorf36			Y	Y		Y		Y								Y	Y																											
21499	A_23_P217168	CXorf36			Y																																									
16207	A_23_P45294	CXorf39																					Y																							
13505	A_23_P306919	CXorf40B																Y																												
18436	A_23_P252630	CXorf40B							Y	Y								Y																												
19088	A_23_P387584	CXorf50				Y																Y					Y																			
6965	A_23_P255457	CXorf51			Y	Y		Y		Y												Y					Y			Y						Y										
9433	A_23_P171223	CXorf56			Y	Y		Y		Y																				Y						Y										
11924	A_23_P96369	CXorf57				Y																																								
12841	A_23_P432352	CXorf61							Y	Y			Y																	Y						Y										
20794	A_23_P336060	CXorf62			Y				Y	Y																																				
13165	A_23_P141779	CXXC1			Y	Y		Y		Y								Y	Y	Y		Y	Y	Y			Y																			
7424	A_23_P121676	CXXC4							Y	Y	Y	Y	Y	Y																Y						Y										
4024	A_23_P213680	CXXC5			Y	Y		Y	Y	Y								Y									Y	Y	Y	Y						Y										
14890	A_23_P218331	CYB561							Y	Y								Y	Y	Y				Y						Y						Y										
16050	A_23_P121326	CYB561D2							Y	Y																																				
12019	A_23_P101208	CYB5A	Y	Y	Y	Y		Y	Y	Y	Y	Y	Y	Y				Y	Y	Y	Y	Y	Y	Y	Y	Y	Y	Y	Y	Y	Y	Y	Y	Y	Y	Y										
7594	A_23_P206697	CYB5B			Y																Y																									
3445	A_23_P341392	CYB5D2	Y	Y	Y				Y	Y	Y	Y	Y	Y				Y	Y	Y		Y		Y			Y				Y		Y	Y	Y							Y				
7061	A_23_P55330	CYB5D2	Y	Y	Y	Y	Y	Y	Y	Y	Y	Y	Y	Y				Y	Y	Y		Y	Y	Y			Y	Y	Y	Y			Y			Y								Y		
12020	A_23_P52101	CYB5R1			Y						Y		Y	Y				Y									Y																			
20180	A_23_P2181	CYB5R2							Y	Y																																				
1682	A_23_P251065	CYB5R3			Y	Y	Y	Y	Y	Y	Y	Y						Y	Y	Y		Y	Y	Y			Y	Y	Y	Y																
18175	A_23_P502224	CYB5R3	Y		Y	Y	Y	Y	Y	Y	Y	Y										Y	Y				Y																			
15587	A_23_P30995	CYB5R4	Y	Y	Y													Y									Y																			
4737	A_23_P163506	CYBA			Y	Y	Y	Y	Y	Y	Y	Y							Y	Y	Y	Y	Y				Y	Y	Y	Y																
21684	A_23_P342132	CYBASC3			Y	Y	Y	Y	Y	Y	Y	Y								Y	Y	Y	Y				Y	Y	Y	Y						Y										
15384	A_23_P217258	CYBB	Y	Y	Y		Y		Y	Y	Y	Y																																		
11555	A_23_P209564	CYBRD1			Y	Y	Y	Y	Y	Y	Y	Y						Y	Y	Y	Y	Y	Y	Y	Y	Y	Y	Y	Y	Y	Y	Y	Y	Y	Y	Y										
15923	A_23_P32384	CYC1			Y	Y		Y	Y	Y									Y								Y	Y	Y	Y																
18951	A_23_P20980	CYC1			Y	Y	Y	Y	Y	Y	Y	Y	Y	Y				Y	Y	Y	Y	Y	Y	Y	Y	Y	Y	Y	Y	Y	Y	Y				Y										
20638	A_23_P9548	CYC1			Y	Y	Y	Y	Y	Y	Y	Y	Y	Y				Y	Y	Y	Y	Y		Y			Y	Y	Y	Y						Y										
2085	A_23_P255849	CYCS	Y	Y	Y	Y		Y		Y																	Y			Y						Y										

5053	A_23_P140646	CYFIP1																	Y																											
5174	A_23_P156117	CYFIP2																											Y						Y										
10965	A_23_P167509	CYFIP2				Y																																							
11970	A_23_P338981	CYGB			Y	Y		Y		Y	Y	Y	Y	Y				Y	Y	Y			Y	Y			Y	Y	Y	Y						Y									
16935	A_23_P65988	CYLD																		Y		Y																							
6966	A_23_P201129	CYMP							Y	Y																																			
19041	A_23_P96652	CYorf15B																Y																											
4166	A_23_P129167	CYP11A1			Y		Y				Y																Y																		
14697	A_23_P168928	CYP11B1			Y	Y	Y	Y		Y	Y	Y															Y																		
11058	A_23_P215997	CYP11B2																		Y																									
9382	A_23_P37410	CYP19A1			Y																						Y																		
5078	A_23_P209625	CYP1B1			Y	Y		Y	Y	Y																	Y			Y						Y									
17830	A_23_P257478	CYP21A2				Y															Y							Y																	
11024	A_23_P28815	CYP24A1			Y														Y	Y							Y																		
13703	A_23_P138655	CYP26A1	Y		Y													Y	Y	Y		Y		Y						Y						Y									
22235	A_23_P210100	CYP26B1			Y				Y	Y	Y	Y	Y	Y																Y						Y									
16189	A_23_P418312	CYP26C1			Y																																								
19480	A_23_P131308	CYP27A1			Y	Y	Y	Y	Y	Y	Y	Y	Y	Y				Y	Y	Y	Y	Y		Y			Y																		
4744	A_23_P36397	CYP27B1							Y	Y																																			
5819	A_23_P73043	CYP27C1																												Y						Y									
2463	A_23_P16130	CYP2A6			Y				Y	Y																	Y																		
8042	A_23_P27530	CYP2A7							Y	Y																																			
678	A_23_P208373	CYP2B6							Y	Y	Y	Y	Y	Y																															
20240	A_23_P38917	CYP2B6				Y			Y	Y	Y	Y	Y	Y																Y						Y									
10210	A_23_P121914	CYP2B7P1																												Y						Y									
11329	A_23_P52480	CYP2C18			Y																																								
12960	A_23_P161368	CYP2C8																Y									Y																		
19245	A_23_P380129	CYP2D6			Y																		Y																						
2538	A_23_P155118	CYP2D7P1																Y	Y	Y			Y	Y			Y																		
5205	A_23_P89981	CYP2F1																												Y						Y									
945	A_23_P103486	CYP2J2				Y			Y	Y								Y																											
5972	A_23_P202860	CYP2R1			Y				Y	Y	Y	Y	Y	Y						Y	Y	Y						Y		Y						Y									
7903	A_23_P155835	CYP2U1				Y													Y																										
5337	A_23_P133712	CYP39A1				Y			Y	Y	Y	Y	Y	Y																Y						Y									
10284	A_23_P215828	CYP3A43			Y													Y												Y						Y									
19973	A_23_P358917	CYP3A7																Y												Y						Y									
13504	A_23_P114708	CYP4B1							Y	Y												Y								Y						Y									
7120	A_23_P101869	CYP4F22				Y																								Y						Y									
6377	A_23_P72411	CYP4X1	Y	Y	Y				Y	Y	Y	Y	Y	Y				Y	Y	Y		Y		Y				Y		Y						Y									
3942	A_23_P318413	CYP4Z1							Y	Y																																			
6302	A_23_P103971	CYP4Z1	Y			Y			Y	Y	Y	Y	Y	Y				Y	Y	Y	Y		Y	Y	Y			Y		Y						Y									
16255	A_23_P913	CYP4Z1	Y	Y		Y			Y	Y	Y	Y	Y	Y				Y	Y	Y	Y		Y	Y	Y					Y						Y									
10034	A_23_P257716	CYP51A1			Y	Y		Y	Y	Y																	Y																		
18538	A_23_P146198	CYP7A1			Y														Y								Y																		
9614	A_23_P169092	CYP7B1			Y				Y	Y	Y	Y	Y	Y													Y			Y						Y									
152	A_23_P29490	CYP8B1																												Y						Y									
14785	A_23_P22660	CYSLTR1																Y												Y						Y									
4777	A_23_P162708	CYSLTR2			Y																																								
12528	A_23_P95184	CYTH1	Y																	Y										Y						Y									
17462	A_23_P83781	CYTH1	Y		Y	Y		Y		Y					Y				Y	Y	Y		Y				Y			Y						Y									
9447	A_23_P119377	CYTH2			Y	Y		Y		Y										Y	Y	Y	Y				Y																		
13909	A_23_P111593	CYTH3																Y		Y																									
7991	A_23_P155057	CYTH4	Y		Y	Y	Y	Y	Y	Y	Y	Y			Y				Y	Y	Y		Y				Y	Y	Y	Y															
20152	A_23_P90626	CYTIP			Y	Y		Y	Y	Y																																			
444	A_23_P371106	D4S234E			Y																																								
9041	A_23_P54119	DAAM1							Y	Y																																			
6589	A_23_P257871	DAB2													Y																														
2574	A_23_P32577	DACH1	Y	Y	Y	Y		Y	Y	Y	Y	Y	Y	Y						Y							Y	Y	Y	Y						Y									
10470	A_23_P205134	DACH1	Y	Y	Y	Y		Y	Y	Y	Y	Y	Y	Y						Y	Y						Y	Y	Y	Y						Y									
21774	A_23_P21178	DACH1	Y	Y	Y	Y		Y	Y	Y	Y	Y	Y	Y						Y							Y	Y	Y	Y						Y									
3828	A_23_P65518	DACT1													Y																														
20733	A_23_P360964	DACT3																		Y			Y																						
15667	A_23_P106056	DAD1			Y	Y		Y		Y																	Y																		
19297	A_23_P212579	DAG1			Y	Y		Y		Y								Y		Y		Y					Y	Y	Y	Y															
6856	A_23_P8532	DAGLB			Y													Y		Y		Y	Y				Y			Y						Y									
19665	A_23_P36127	DAK																Y	Y	Y				Y																					
20445	A_23_P135611	DALRD3							Y	Y																																			
20658	A_23_P435636	DAND5			Y																						Y	Y	Y	Y						Y									
11955	A_23_P252163	DAPK1	Y	Y	Y	Y		Y	Y	Y					Y			Y	Y	Y	Y	Y	Y	Y	Y	Y				Y	Y	Y	Y	Y	Y	Y				Y					
17524	A_23_P206150	DAPK2			Y				Y	Y			Y						Y	Y										Y						Y									
5253	A_23_P165028	DAPK3			Y													Y		Y	Y	Y	Y																						
5682	A_23_P255444	DAPP1			Y																						Y																		
17240	A_23_P115154	DARC			Y													Y	Y				Y				Y																		
7560	A_23_P108415	DARS							Y	Y													Y				Y																		
3963	A_23_P148980	DARS2			Y	Y		Y		Y																																			
6756	A_23_P122579	DAXX																	Y			Y																							
13238	A_23_P165247	DAZAP1			Y	Y		Y	Y	Y								Y	Y	Y		Y	Y	Y			Y	Y	Y	Y						Y									

16395	A_23_P40025	DAZAP2			Y				Y	Y										Y			Y				Y			Y						Y										
5364	A_23_P212105	DAZL																									Y																			
C12102.8	A_23_P254612	DBF4	Y	Y																																										
12966	A_23_P79199	DBI			Y	Y		Y	Y	Y																	Y																			
18801	A_23_P156284	DBN1							Y	Y								Y												Y						Y										
22538	A_23_P26434	DBNDD1			Y	Y	Y	Y	Y	Y	Y	Y						Y	Y	Y		Y	Y	Y			Y	Y	Y	Y						Y										
818	A_23_P28772	DBNDD2			Y																																									
22570	A_23_P168692	DBNL			Y	Y		Y	Y	Y								Y	Y	Y	Y	Y	Y	Y	Y	Y	Y	Y	Y	Y	Y	Y	Y			Y										
17347	A_23_P69452	DBR1			Y																																									
9290	A_23_P113138	DCAKD																Y																												
14913	A_23_P124522	DCAKD			Y	Y		Y	Y	Y																	Y			Y						Y										
17788	A_23_P258231	DCBLD1																	Y																											
15230	A_23_P134160	DCDC2	Y		Y	Y		Y	Y	Y								Y	Y	Y				Y			Y	Y	Y	Y			Y	Y		Y										
14067	A_23_P98640	DCHS1			Y	Y	Y	Y	Y	Y	Y	Y															Y			Y						Y										
2855	A_23_P163647	DCI	Y	Y	Y				Y	Y													Y				Y			Y						Y										
13709	A_23_P259438	DCK	Y																																											
7847	A_23_P369999	DCLK1	Y	Y	Y	Y	Y	Y	Y	Y	Y	Y	Y	Y	Y			Y	Y	Y	Y	Y		Y			Y	Y	Y	Y	Y	Y	Y	Y	Y	Y										
2546	A_23_P304524	DCLK2							Y	Y										Y																										
2408	A_23_P310864	DCLK3																Y				Y																								
3916	A_23_P75038	DCLRE1A			Y	Y		Y	Y	Y	Y	Y	Y	Y						Y			Y					Y																		
4512	A_23_P97372	DCLRE1B																		Y																										
7364	A_23_P64873	DCN																										Y																		
17598	A_23_P166826	DCP1A			Y	Y		Y	Y	Y	Y	Y	Y	Y					Y	Y		Y					Y	Y	Y	Y						Y										
22493	A_23_P313512	DCP1B			Y				Y	Y																																				
8260	A_23_P256868	DCP2							Y	Y										Y										Y						Y										
10909	A_23_P52738	DCPS			Y	Y		Y		Y																	Y	Y	Y	Y						Y										
18195	A_23_P76622	DCT			Y																									Y						Y										
3153	A_23_P81172	DCTD																Y																												
1147	A_23_P142835	DCTN1			Y	Y	Y	Y	Y	Y	Y	Y	Y	Y				Y	Y	Y	Y	Y	Y	Y	Y	Y	Y				Y															
11746	A_23_P76065	DCTN2			Y													Y		Y	Y	Y	Y				Y																			
17335	A_23_P360167	DCTN2																Y		Y	Y	Y	Y																							
4983	A_23_P251945	DCTN4			Y	Y	Y	Y	Y	Y	Y	Y	Y	Y				Y	Y	Y	Y	Y	Y	Y	Y	Y	Y	Y	Y	Y	Y	Y				Y										
7458	A_23_P309967	DCTN5				Y															Y	Y																								
2768	A_23_P33613	DCTPP1			Y	Y	Y	Y		Y	Y	Y							Y	Y		Y					Y																			
16791	A_23_P367044	DCUN1D3			Y																						Y																			
7501	A_23_P500328	DCX																									Y																			
10674	A_23_P44166	DCXR							Y	Y									Y																											
14188	A_23_P16666	DDA1			Y	Y		Y	Y	Y	Y	Y	Y	Y					Y	Y							Y	Y	Y	Y						Y										
17079	A_23_P201386	DDAH1	Y	Y	Y	Y	Y	Y	Y	Y	Y	Y	Y	Y						Y	Y		Y				Y	Y	Y	Y						Y										
1234	A_23_P19482	DDAH2	Y	Y	Y																						Y			Y						Y										
20907	A_23_P52610	DDB2			Y																						Y																			
1118	A_23_P145644	DDC			Y																		Y				Y																			
8485	A_23_P146325	DDEF1IT1			Y	Y		Y	Y	Y																		Y																		
19133	A_23_P205628	DDHD1	Y																								Y			Y						Y										
15438	A_23_P63114	DDI2																		Y		Y																								
11758	A_23_P104318	DDIT4			Y																						Y																			
3077	A_23_P30603	DDO							Y	Y																																				
17176	A_23_P149099	DDOST			Y	Y	Y	Y		Y	Y	Y						Y	Y	Y	Y	Y		Y			Y	Y	Y	Y																
7057	A_23_P93311	DDR1																Y	Y	Y				Y																						
9256	A_23_P452	DDR2																		Y																										
4341	A_23_P403968	DDRGK1			Y	Y		Y	Y	Y											Y						Y			Y						Y										
21450	A_23_P17764	DDT			Y																						Y																			
3494	A_23_P108554	DDX1				Y	Y				Y								Y	Y		Y						Y																		
22496	A_23_P32938	DDX10																		Y		Y	Y																							
7005	A_23_P203949	DDX11																		Y																										
17573	A_23_P140994	DDX19B			Y	Y	Y	Y	Y	Y	Y	Y	Y	Y									Y				Y			Y						Y										
19917	A_23_P63153	DDX20			Y	Y		Y	Y	Y								Y	Y	Y		Y	Y	Y				Y																		
2174	A_23_P64770	DDX23				Y			Y	Y																																				
7330	A_23_P151544	DDX24			Y															Y	Y	Y																								
21305	A_23_P138910	DDX25	Y	Y	Y	Y		Y		Y			Y														Y	Y	Y	Y						Y										
5901	A_23_P106633	DDX28																									Y			Y						Y										
15330	A_23_P78664	DDX39			Y	Y	Y	Y	Y	Y	Y	Y	Y	Y				Y	Y	Y	Y	Y	Y	Y	Y	Y	Y				Y															
1597	A_23_P217797	DDX3Y	Y		Y													Y									Y																			
3490	A_23_P256624	DDX4			Y				Y	Y	Y	Y	Y	Y													Y			Y						Y										
12522	A_23_P122113	DDX41																		Y	Y	Y																								
5401	A_23_P156445	DDX43				Y																																								
21210	A_23_P30378	DDX46			Y	Y		Y	Y	Y										Y								Y																		
15134	A_23_P65089	DDX47			Y															Y			Y																							
12626	A_23_P16573	DDX49			Y	Y	Y	Y		Y	Y	Y	Y					Y	Y	Y		Y		Y			Y	Y	Y	Y						Y										
20274	A_23_P15511	DDX5				Y																						Y																		
10487	A_23_P104451	DDX50			Y	Y		Y		Y										Y																										
8446	A_23_P118660	DDX52																		Y							Y																			
7833	A_23_P162374	DDX54			Y	Y		Y		Y								Y	Y	Y	Y	Y	Y	Y	Y	Y	Y	Y	Y	Y	Y	Y														
6693	A_23_P47843	DDX55			Y	Y		Y	Y	Y																		Y		Y						Y										
10433	A_23_P168683	DDX56			Y	Y		Y		Y								Y	Y	Y	Y	Y		Y																						
10011	A_23_P41470	DDX60																		Y			Y																							
323	A_23_P30069	DDX60L																	Y																											

10556	A_23_P124049	DEAF1																				Y																							
13009	A_23_P158533	DEAF1			Y															Y		Y																							
12327	A_23_P106973	DEC1_																	Y			Y																							
16941	A_23_P146486	DEC1_							Y	Y																					Y						Y										
7134	A_23_P71464	DECR1			Y																						Y																			
9019	A_23_P74711	DEDD			Y	Y		Y		Y																	Y	Y	Y	Y						Y										
7360	A_23_P323301	DEDD2			Y	Y	Y	Y		Y	Y	Y						Y	Y	Y	Y	Y	Y	Y	Y	Y	Y	Y	Y	Y	Y	Y													Y		
14566	A_23_P141908	DEDD2			Y	Y	Y	Y	Y	Y	Y	Y							Y	Y		Y	Y				Y	Y	Y	Y																
6401	A_23_P321913	DEF6			Y	Y	Y	Y		Y	Y	Y						Y	Y	Y		Y	Y	Y			Y			Y						Y										
11013	A_23_P19532	DEF6																									Y																			
12291	A_23_P88893	DEF8	Y	Y	Y	Y		Y	Y	Y	Y	Y	Y	Y				Y		Y			Y				Y	Y	Y	Y						Y										
22004	A_23_P31816	DEFA3			Y															Y							Y			Y						Y										
20933	A_23_P112086	DEFA5	Y	Y																								Y																		
10041	A_23_P71480	DEFB1			Y														Y																											
909	A_23_P425815	DEFB106B							Y	Y																																				
8325	A_23_P385843	DEFB127			Y																						Y																			
21319	A_23_P109109	DEFB132																												Y						Y										
11378	A_23_P88450	DEGS2				Y			Y	Y	Y	Y	Y	Y																Y						Y										
4316	A_23_P320897	DENND1B	Y	Y	Y	Y	Y	Y	Y	Y	Y	Y	Y	Y					Y	Y		Y	Y				Y																			
15496	A_23_P201605	DENND1B	Y	Y																			Y																							
15796	A_23_P78608	DENND1C	Y		Y																						Y																			
14839	A_23_P257581	DENND2A																		Y	Y		Y																							
19649	A_23_P46315	DENND2C			Y				Y	Y										Y							Y			Y						Y										
12906	A_23_P85952	DENND2D																Y		Y		Y	Y																							
1395	A_23_P774	DENND4B																Y	Y	Y	Y	Y	Y	Y	Y	Y					Y															
6963	A_23_P157726	DENND4C	Y	Y	Y	Y	Y	Y	Y	Y	Y	Y	Y	Y				Y	Y	Y		Y	Y	Y			Y	Y	Y	Y						Y										
13035	A_23_P321201	DENND5A				Y																																								
20307	A_23_P200310	DEPDC1			Y																						Y																			
11415	A_23_P361419	DEPDC1B	Y	Y																																										
14950	A_23_P91683	DEPDC5				Y																																								
2351	A_23_P60166	DEPDC6			Y				Y	Y								Y		Y																										
18925	A_23_P385126	DEPDC7			Y													Y	Y	Y			Y	Y			Y																			
4416	A_23_P330908	DERL1			Y	Y		Y		Y																		Y																		
6999	A_23_P216043	DERL1	Y	Y	Y	Y	Y	Y	Y	Y	Y	Y	Y	Y					Y	Y							Y	Y	Y	Y						Y										
8874	A_23_P118749	DERL2																		Y			Y																							
14343	A_23_P6362	DERL3			Y		Y		Y	Y	Y	Y															Y																			
3801	A_23_P90710	DES				Y																																								
12380	A_23_P26184	DET1			Y				Y	Y																	Y			Y						Y										
4719	A_23_P54441	DEXI																		Y		Y																								
882	A_23_P12189	DFFA	Y	Y																																										
14018	A_23_P44546	DFFB																					Y																							
7211	A_23_P112162	DGAT1			Y	Y		Y	Y	Y								Y	Y	Y	Y	Y	Y	Y	Y	Y	Y			Y	Y	Y				Y										
4810	A_23_P53198	DGAT2			Y	Y		Y	Y	Y	Y	Y	Y	Y						Y		Y					Y	Y	Y	Y						Y										
7901	A_23_P102973	DGCR14			Y	Y	Y	Y	Y	Y	Y	Y	Y	Y				Y	Y	Y		Y	Y	Y			Y	Y	Y	Y						Y										
9904	A_23_P211336	DGCR2			Y				Y	Y								Y	Y	Y		Y		Y																						
20174	A_23_P29114	DGCR5			Y				Y	Y	Y	Y	Y	Y				Y	Y	Y		Y		Y			Y	Y	Y	Y						Y										
19061	A_23_P17714	DGCR6			Y													Y		Y		Y					Y																			
5696	A_23_P143569	DGCR6L			Y	Y		Y	Y	Y								Y	Y	Y		Y		Y			Y			Y						Y										
8228	A_23_P105307	DGKA																		Y										Y						Y										
14246	A_23_P210253	DGKD				Y			Y	Y	Y	Y	Y	Y																Y						Y										
16542	A_23_P141163	DGKE	Y	Y	Y																						Y			Y						Y										
19125	A_23_P40926	DGKG			Y	Y		Y	Y	Y	Y	Y	Y	Y								Y								Y						Y										
7396	A_23_P111432	DGKI			Y	Y		Y	Y	Y										Y		Y					Y	Y	Y	Y						Y										
18979	A_23_P169909	DGKI																	Y								Y																			
19032	A_23_P132923	DGKQ			Y	Y	Y	Y	Y	Y	Y	Y								Y			Y				Y																	Y		
13861	A_23_P127627	DGKZ			Y															Y		Y	Y				Y																			
7630	A_23_P379481	DHCR24	Y	Y					Y	Y	Y	Y	Y	Y									Y				Y	Y	Y	Y						Y										
16120	A_23_P217821	DHCR24			Y																						Y			Y						Y										
13524	A_23_P24444	DHCR7			Y																						Y																			
21667	A_23_P201764	DHDDS			Y															Y		Y					Y																			
14369	A_23_P67367	DHDH																	Y								Y																			
17737	A_23_P167553	DHFR																									Y																			
18343	A_23_P44627	DHFR			Y	Y		Y	Y	Y	Y	Y	Y	Y														Y		Y						Y										
20661	A_23_P139312	DHFRL1																										Y																		
1808	A_23_P15202	DHODH			Y	Y		Y		Y									Y								Y																			
8350	A_23_P501887	DHPS			Y	Y		Y		Y										Y		Y																								
1418	A_23_P27005	DHRS11			Y	Y		Y		Y									Y	Y		Y					Y	Y	Y	Y																
7240	A_23_P205200	DHRS12							Y	Y								Y			Y	Y																								
6499	A_23_P48570	DHRS2							Y	Y	Y	Y	Y	Y																Y						Y										
12879	A_23_P321501	DHRS2				Y			Y	Y																		Y		Y						Y										
18926	A_23_P33759	DHRS3			Y	Y		Y	Y	Y									Y	Y		Y					Y																			
4449	A_23_P162982	DHRS4																Y	Y	Y		Y	Y	Y																						
18841	A_23_P56559	DHRS9	Y		Y				Y	Y																	Y	Y	Y	Y						Y										
10270	A_23_P254904	DHRSX																		Y																										
13538	A_23_P251339	DHRSX																		Y																										
8684	A_23_P12593	DHTKD1			Y													Y	Y	Y	Y	Y	Y	Y	Y	Y	Y			Y	Y	Y	Y			Y				Y						
16089	A_23_P69826	DHX15				Y			Y	Y																	Y			Y						Y										

11678	A_23_P168062	DHX16																	Y																											
11331	A_23_P81463	DHX29			Y	Y	Y	Y	Y	Y	Y	Y	Y	Y				Y	Y	Y		Y	Y	Y			Y	Y	Y	Y						Y										
10275	A_23_P321361	DHX30																	Y	Y		Y	Y																							
14266	A_23_P166609	DHX30																Y	Y	Y		Y	Y	Y																						
13094	A_23_P47004	DHX32																	Y	Y			Y				Y																			
17267	A_23_P5945	DHX35			Y	Y		Y	Y	Y																	Y	Y	Y	Y						Y										
21563	A_23_P57697	DHX36																		Y			Y																							
11556	A_23_P38011	DHX38			Y	Y	Y	Y	Y	Y	Y	Y	Y	Y				Y	Y	Y	Y	Y	Y	Y	Y	Y	Y			Y	Y	Y				Y										
21390	A_23_P152718	DHX40			Y	Y		Y	Y	Y										Y							Y	Y	Y	Y						Y										
11225	A_23_P16904	DHX57			Y	Y		Y	Y	Y																																		
2359	A_23_P38346	DHX58			Y																									Y						Y										
3778	A_23_P124224	DHX8			Y																						Y																			
22558	A_23_P97749	DHX9			Y	Y		Y	Y	Y										Y							Y																			
5369	A_23_P47800	DIABLO																		Y																										
9648	A_23_P213344	DIAPH1			Y	Y	Y	Y	Y	Y	Y	Y	Y	Y				Y	Y	Y	Y	Y	Y	Y	Y	Y	Y			Y	Y	Y				Y										
1065	A_23_P83871	DIAPH2				Y																																								
12588	A_23_P162719	DIAPH3																	Y																											
7743	A_23_P37111	DICER1			Y	Y		Y	Y	Y	Y	Y	Y	Y				Y	Y	Y		Y		Y			Y	Y	Y	Y						Y										
3713	A_23_P359596	DIDO1							Y	Y																																		
193	A_23_P58529	DIMT1L																			Y																									
14368	A_23_P105926	DIO3				Y																																								
18696	A_23_P140096	DIO3OS	Y	Y	Y	Y	Y	Y	Y	Y	Y	Y	Y	Y				Y		Y			Y				Y	Y	Y	Y						Y										
15235	A_23_P119728	DIRAS1			Y															Y	Y	Y	Y				Y																			
18270	A_23_P386942	DIRAS1																										Y		Y						Y										
20849	A_23_P379778	DIRAS2																										Y																		
7141	A_23_P149121	DIRAS3							Y	Y										Y							Y																			
6489	A_23_P131139	DIRC1																									Y																			
22183	A_23_P80778	DIRC2																					Y											Y													
8990	A_23_P48419	DIS3			Y	Y	Y	Y	Y	Y	Y	Y	Y	Y				Y	Y	Y	Y	Y	Y	Y	Y	Y	Y	Y	Y	Y	Y	Y	Y	Y	Y	Y										
9834	A_23_P65741	DIS3L	Y	Y	Y	Y		Y	Y	Y													Y				Y	Y	Y	Y																
11544	A_23_P406376	DIS3L2			Y	Y		Y		Y																	Y																			
15918	A_23_P113882	DIS3L2							Y	Y												Y																								
11447	A_23_P201319	DISP1			Y	Y	Y	Y	Y	Y	Y	Y	Y	Y				Y	Y	Y	Y	Y	Y	Y	Y	Y	Y	Y	Y	Y	Y	Y	Y	Y	Y	Y				Y						
20147	A_23_P137143	DKC1							Y	Y																																		
13288	A_23_P201109	DKFZP434C153																					Y																							
15473	A_23_P111797	DKFZp434F142			Y	Y		Y		Y								Y	Y	Y				Y			Y	Y	Y	Y						Y										
5119	A_23_P91630	DKFZP434P211			Y	Y		Y		Y									Y	Y		Y					Y	Y	Y	Y						Y										
13623	A_23_P150747	DKFZp547G183			Y	Y		Y	Y	Y									Y	Y		Y	Y				Y	Y	Y	Y																
18705	A_23_P96809	DKFZp566H0824			Y	Y		Y		Y	Y	Y	Y	Y														Y		Y						Y										
4637	A_23_P127911	DKFZP586H2123																Y	Y																											
1109	A_23_P403395	DKFZP586I1420			Y	Y		Y	Y	Y	Y	Y	Y	Y														Y		Y						Y										
7092	A_23_P24424	DKFZp761E198			Y	Y	Y	Y	Y	Y	Y	Y	Y	Y				Y		Y		Y					Y	Y	Y	Y						Y										
18203	A_23_P24129	DKK1			Y				Y	Y																		Y		Y						Y										
13500	A_23_P162047	DKK3																Y	Y																											
13973	A_23_P130743	DKKL1																										Y		Y						Y										
82	A_23_P203030	DLAT																		Y			Y																							
15154	A_23_P151337	DLEU1			Y	Y	Y	Y		Y	Y	Y						Y		Y																										
12783	A_23_P22072	DLEU2							Y	Y																																		
4547	A_23_P416142	DLG1			Y	Y		Y	Y	Y																																		
16797	A_23_P212034	DLG1			Y															Y			Y																							
11939	A_23_P113121	DLG2			Y				Y	Y																		Y		Y						Y										
15845	A_23_P124514	DLG2																Y			Y																									
9868	A_23_P411102	DLG4			Y	Y		Y	Y	Y									Y																											
5463	A_23_P161209	DLG5							Y	Y																		Y		Y						Y										
3629	A_23_P112128	DLGAP2			Y																Y																									
15348	A_23_P392314	DLGAP2																				Y																								
14721	A_23_P149707	DLGAP3			Y															Y		Y	Y																							
15576	A_23_P210419	DLGAP4			Y				Y	Y																																		
4033	A_23_P133786	DLK2			Y																						Y																			
13587	A_23_P167920	DLL1																Y																												
1070	A_23_P419641	DLL4			Y	Y		Y		Y								Y	Y	Y		Y	Y	Y			Y			Y						Y										
9048	A_23_P26266	DLL4			Y	Y		Y		Y								Y	Y	Y				Y			Y			Y						Y										
3924	A_23_P205697	DLST																Y																												
8410	A_23_P403466	DLX1																				Y																								
11251	A_23_P31268	DLX5									Y		Y	Y																														
22531	A_23_P318262	DLX5				Y			Y	Y																		Y																		
20489	A_23_P86599	DMBT1																		Y																										
8053	A_23_P209101	DMKN																		Y																										
13768	A_23_P320261	DMKN				Y			Y	Y									Y		Y	Y	Y							Y						Y										
8045	A_23_P300245	DMRTC2							Y	Y																																		
21603	A_23_P56010	DMWD			Y	Y		Y		Y										Y			Y				Y			Y						Y										
8035	A_23_P250571	DMXL1			Y	Y		Y	Y	Y	Y	Y	Y	Y					Y	Y		Y					Y	Y	Y	Y						Y										
20763	A_23_P113582	DMXL1			Y	Y	Y	Y	Y	Y	Y	Y							Y	Y		Y					Y	Y	Y	Y						Y										
2411	A_23_P14609	DMXL2			Y	Y		Y		Y								Y	Y	Y		Y		Y			Y	Y	Y	Y																
17471	A_23_P89236	DNAH17							Y	Y																				Y						Y										
22468	A_23_P33583	DNAH7			Y	Y	Y	Y	Y	Y	Y	Y	Y	Y				Y	Y	Y	Y	Y	Y	Y	Y	Y	Y	Y	Y	Y	Y	Y	Y	Y	Y	Y				Y						
13066	A_23_P60479	DNAJA1				Y																																								

9503	A_23_P15083	DNAJA2			Y																						Y																			
10821	A_23_P206140	DNAJA4							Y	Y																																			
1783	A_23_P90062	DNAJB1			Y																																								
16594	A_23_P166899	DNAJB11			Y				Y	Y																	Y			Y						Y										
3959	A_23_P52380	DNAJB12																		Y			Y																						
10390	A_23_P213199	DNAJB14				Y														Y								Y																	
3716	A_23_P209519	DNAJB2			Y	Y		Y	Y	Y								Y	Y	Y		Y		Y			Y																		
11040	A_23_P112241	DNAJB5							Y	Y																																			
1909	A_23_P258944	DNAJB9			Y																																								
18800	A_23_P127128	DNAJC1																												Y						Y										
12367	A_23_P90669	DNAJC10																		Y																									
12607	A_23_P383885	DNAJC10																		Y																									
13781	A_23_P103942	DNAJC11			Y				Y	Y									Y	Y		Y	Y				Y																		
11786	A_23_P127220	DNAJC12	Y	Y	Y	Y	Y	Y	Y	Y	Y	Y	Y	Y				Y	Y	Y	Y	Y	Y	Y	Y	Y	Y	Y	Y	Y	Y	Y				Y				Y						
7056	A_23_P150867	DNAJC14																Y																												
13227	A_23_P433079	DNAJC14			Y																									Y						Y										
9037	A_23_P123983	DNAJC18																Y	Y	Y				Y																					
1587	A_23_P121396	DNAJC19			Y	Y	Y	Y	Y	Y	Y	Y						Y	Y	Y	Y	Y	Y	Y	Y	Y	Y	Y	Y	Y	Y	Y														
2465	A_23_P431890	DNAJC19			Y	Y		Y		Y										Y			Y																						
1772	A_23_P110527	DNAJC21			Y														Y	Y			Y																						
606	A_23_P253661	DNAJC22																												Y						Y										
11085	A_23_P47421	DNAJC24																		Y			Y																						
8662	A_23_P17275	DNAJC27				Y																																							
10872	A_23_P68700	DNAJC28																		Y			Y																						
3733	A_23_P202769	DNAJC4			Y																						Y																		
15783	A_23_P8959	DNAJC5B													Y																														
8276	A_23_P417921	DNAJC5G																				Y																							
5426	A_23_P95359	DNAJC6																					Y							Y						Y										
9224	A_23_P37137	DNAJC8			Y	Y		Y		Y								Y			Y						Y																		
5532	A_23_P17880	DNAL4			Y	Y		Y	Y	Y										Y		Y					Y	Y	Y	Y						Y										
13889	A_23_P160377	DNALI1	Y	Y	Y	Y	Y	Y	Y	Y	Y	Y	Y	Y						Y		Y					Y	Y	Y	Y						Y										
10237	A_23_P66311	DNASE1																												Y						Y										
12904	A_23_P257993	DNASE1L3																	Y											Y						Y										
7955	A_23_P119262	DNASE2				Y			Y	Y																																			
12068	A_23_P126677	DNASE2B							Y	Y	Y	Y	Y	Y				Y												Y						Y										
2597	A_23_P316472	DNHD1				Y																																							
9481	A_23_P372496	DNHD2				Y																																							
19534	A_23_P216836	DNLZ			Y	Y		Y		Y								Y	Y	Y		Y	Y	Y			Y	Y	Y	Y																
15707	A_23_P204324	DNM1L				Y																																							
3441	A_23_P106472	DNM1P35			Y	Y		Y	Y	Y									Y	Y		Y	Y																						
9457	A_23_P130669	DNM2			Y	Y	Y	Y	Y	Y	Y	Y	Y	Y				Y	Y	Y	Y	Y	Y	Y	Y	Y	Y	Y	Y	Y	Y	Y				Y										
17414	A_23_P407070	DNM2			Y	Y	Y	Y	Y	Y	Y	Y	Y	Y				Y	Y	Y	Y	Y	Y	Y	Y	Y	Y	Y	Y	Y	Y	Y				Y										
7786	A_23_P28953	DNMT3B			Y													Y		Y																									
4056	A_23_P17673	DNMT3L			Y	Y		Y	Y	Y									Y								Y	Y	Y	Y																
18366	A_23_P142675	DNPEP																		Y																									
8210	A_23_P409489	DNTT	Y	Y	Y	Y		Y		Y								Y		Y			Y				Y																		
19737	A_23_P46970	DNTT	Y	Y	Y	Y		Y	Y	Y										Y			Y				Y	Y	Y	Y						Y										
973	A_23_P131816	DNTTIP1				Y														Y		Y						Y																	
C8607.7	A_23_P97584	DNTTIP2				Y														Y																									
18023	A_23_P152327	DOC2A			Y	Y		Y	Y	Y																	Y			Y						Y										
9456	A_23_P61927	DOCK1	Y	Y	Y	Y	Y	Y	Y	Y	Y	Y	Y	Y					Y	Y		Y					Y	Y	Y	Y						Y										
21301	A_23_P45059	DOCK1			Y	Y	Y	Y	Y	Y	Y	Y	Y	Y				Y	Y	Y		Y	Y	Y			Y	Y	Y	Y						Y										
21870	A_23_P16722	DOCK10																	Y																										
21861	A_23_P59637	DOCK4																Y	Y																										
19390	A_23_P60089	DOCK5			Y	Y		Y	Y	Y																	Y	Y	Y	Y						Y										
6622	A_23_P67299	DOCK6			Y				Y	Y								Y	Y	Y		Y	Y	Y			Y			Y						Y										
19553	A_23_P162766	DOCK9				Y														Y							Y	Y	Y	Y						Y										
17469	A_23_P125408	DOHH			Y	Y	Y	Y	Y	Y	Y	Y	Y	Y				Y	Y	Y	Y	Y	Y	Y	Y	Y	Y	Y	Y	Y	Y	Y	Y			Y										
20145	A_23_P134851	DOK2	Y	Y	Y	Y		Y	Y	Y								Y	Y	Y		Y	Y	Y			Y	Y	Y	Y	Y	Y	Y	Y	Y											
8162	A_23_P133506	DOK3	Y		Y	Y	Y	Y	Y	Y	Y	Y	Y	Y	Y				Y	Y	Y		Y				Y	Y	Y	Y			Y													
111	A_23_P40217	DOK5							Y	Y	Y	Y	Y	Y																Y						Y										
18372	A_23_P352484	DOK6			Y	Y	Y	Y	Y	Y	Y	Y							Y	Y							Y			Y						Y										
11230	A_23_P330017	DOLPP1																		Y		Y																							
6100	A_23_P133934	DOM3Z			Y	Y		Y	Y	Y										Y		Y					Y																		
14206	A_23_P253587	DOPEY2				Y			Y	Y																		Y																	
3498	A_23_P408768	DOT1L				Y																																							
19376	A_23_P101737	DOT1L																		Y	Y		Y																						
21017	A_23_P1775	DPAGT1			Y																						Y																		
94	A_23_P122508	DPCR1				Y																						Y																	
6555	A_23_P118025	DPEP2			Y										Y				Y	Y		Y	Y																						
3061	A_23_P129413	DPEP3							Y	Y													Y							Y						Y										
20764	A_23_P127446	DPF2																Y				Y																							
20321	A_23_P126426	DPH2			Y	Y	Y	Y		Y	Y	Y	Y	Y				Y	Y	Y		Y		Y			Y	Y	Y	Y																
9238	A_23_P217079	DPM2			Y	Y	Y	Y		Y	Y	Y						Y	Y	Y	Y	Y		Y			Y			Y						Y										
21925	A_23_P250506	DPM3				Y																						Y																	
9818	A_23_P102278	DPP10																			Y	Y																							
12996	A_23_P116091	DPP3			Y	Y		Y	Y	Y									Y	Y		Y	Y				Y	Y	Y	Y						Y										

20866	A_23_P170888	DPP6	Y																Y																											
4732	A_23_P32975	DPP7																		Y																										
17334	A_23_P21574	DPP7			Y															Y		Y	Y																							
17900	A_23_P72830	DPP7																		Y		Y																								
12603	A_23_P205921	DPP8			Y	Y		Y	Y	Y									Y	Y		Y	Y				Y	Y	Y	Y						Y										
19458	A_23_P208918	DPP9			Y	Y	Y	Y	Y	Y	Y	Y	Y	Y				Y	Y	Y	Y	Y	Y	Y	Y	Y	Y			Y	Y	Y				Y										
8625	A_23_P255925	DPPA4	Y	Y	Y	Y	Y	Y	Y	Y	Y	Y	Y	Y				Y									Y	Y	Y	Y						Y						Y				
1694	A_23_P427533	DPT	Y		Y															Y		Y	Y				Y																			
2437	A_23_P200741	DPT			Y																		Y				Y																			
19446	A_23_P20336	DPYSL2			Y	Y		Y		Y																	Y																			
4433	A_23_P7528	DPYSL3																		Y																										
7587	A_23_P401691	DPYSL5																										Y		Y						Y										
4880	A_23_P391725	DR1	Y	Y	Y				Y	Y					Y												Y																			
22420	A_23_P63205	DR1																												Y						Y										
13727	A_23_P99163	DRAM	Y	Y			Y				Y				Y						Y												Y	Y												
7933	A_23_P138857	DRAP1			Y		Y				Y		Y	Y						Y	Y	Y	Y				Y			Y						Y										
2224	A_23_P203062	DRD2			Y																																									
22324	A_23_P57733	DRD3			Y	Y		Y	Y	Y																	Y	Y	Y	Y						Y										
12519	A_23_P150162	DRD4																		Y	Y	Y																								
30	A_23_P68884	DRG1			Y	Y		Y		Y																																				
16331	A_23_P141656	DRG2			Y	Y		Y		Y									Y	Y		Y					Y	Y	Y	Y																
19494	A_23_P12693	DRGX				Y														Y		Y								Y						Y										
9743	A_23_P62196	DRP2																					Y																							
18887	A_23_P38696	DSC1				Y																																								
20109	A_23_P4494	DSC2			Y				Y	Y								Y									Y																			
7157	A_23_P211141	DSCAM	Y		Y								Y					Y																												
13898	A_23_P47340	DSCAML1			Y	Y		Y		Y																																				
4131	A_23_P394874	DSCR10			Y	Y		Y	Y	Y	Y	Y	Y	Y					Y								Y			Y						Y										
502	A_23_P154874	DSCR3			Y	Y		Y		Y																	Y	Y	Y	Y																
16443	A_23_P6252	DSCR8																Y		Y							Y																			
13688	A_23_P430691	DSCR9			Y	Y		Y		Y																	Y																			
17730	A_23_P27266	DSEL				Y													Y	Y																										
9579	A_23_P141730	DSG2							Y	Y																																				
21039	A_23_P165937	DSN1			Y													Y		Y							Y																			
12203	A_23_P31031	DSP																Y					Y																							
20879	A_23_P373541	DSPP																												Y						Y										
5796	A_23_P59388	DST	Y		Y	Y		Y		Y			Y														Y																			
14402	A_23_P408095	DSTN							Y	Y										Y			Y					Y																		
21173	A_23_P23893	DSTYK			Y				Y	Y																																				
21947	A_23_P17512	DTD1	Y	Y	Y				Y	Y	Y	Y	Y	Y				Y									Y																			
5567	A_23_P210319	DTNB			Y																						Y																			
C11293.1	A_23_P3215	DTWD1																		Y																										
20675	A_23_P346982	DTWD2							Y	Y																																				
20166	A_23_P395033	DTX2			Y	Y		Y		Y																	Y																			
5030	A_23_P336709	DTX3																Y																												
18879	A_23_P53247	DTX3	Y	Y	Y	Y	Y	Y	Y	Y	Y	Y						Y									Y	Y	Y	Y						Y										
17622	A_23_P135364	DTYMK			Y	Y		Y		Y								Y	Y	Y		Y		Y			Y	Y	Y	Y																
779	A_23_P4277	DULLARD																		Y																										
21108	A_23_P65696	DUOXA2			Y	Y	Y	Y	Y	Y	Y	Y	Y	Y				Y	Y	Y		Y	Y	Y			Y	Y	Y	Y						Y										
16090	A_23_P255569	DUS1L			Y	Y	Y	Y		Y	Y	Y	Y	Y				Y	Y	Y	Y	Y	Y	Y	Y	Y	Y	Y	Y	Y	Y	Y				Y										
4541	A_23_P88840	DUS2L			Y	Y	Y	Y	Y	Y	Y	Y							Y	Y			Y				Y	Y	Y	Y						Y										
16294	A_23_P325542	DUS3L			Y	Y	Y	Y	Y	Y	Y	Y	Y	Y				Y	Y	Y	Y	Y	Y	Y	Y	Y	Y	Y	Y	Y	Y	Y				Y										
11417	A_23_P215778	DUS4L			Y	Y		Y		Y								Y	Y	Y		Y	Y	Y			Y																			
7025	A_23_P376759	DUSP11			Y																																									
16788	A_23_P51508	DUSP12			Y															Y	Y		Y																							
21129	A_23_P104471	DUSP13			Y																						Y																			
6815	A_23_P390474	DUSP14							Y	Y										Y			Y																							
12235	A_23_P154766	DUSP15			Y	Y		Y		Y										Y		Y	Y				Y																			
21795	A_23_P90938	DUSP19																	Y	Y		Y	Y																							
8468	A_23_P108842	DUSP2			Y	Y		Y		Y										Y			Y					Y																		
12048	A_23_P120252	DUSP22			Y																																									
18725	A_23_P167905	DUSP22																					Y																							
1598	A_23_P146134	DUSP26							Y	Y																																				
21854	A_23_P329679	DUSP27																					Y																							
7638	A_23_P400255	DUSP3			Y	Y		Y	Y	Y										Y								Y		Y						Y										
9421	A_23_P134935	DUSP4							Y	Y	Y	Y	Y	Y													Y			Y						Y										
7110	A_23_P150016	DUSP5							Y	Y																																				
16475	A_23_P155430	DUSP7	Y	Y	Y	Y	Y	Y	Y	Y	Y	Y	Y	Y				Y	Y	Y	Y	Y	Y	Y	Y	Y	Y	Y	Y	Y	Y	Y				Y		Y				Y			Y	Y
4452	A_23_P171329	DUSP9			Y	Y		Y		Y								Y	Y	Y	Y	Y	Y	Y	Y	Y	Y				Y															
19282	A_23_P146876	DUX1																		Y																										
17933	A_23_P158257	DUX3			Y	Y		Y		Y										Y							Y	Y	Y	Y																
9696	A_23_P104413	DUX4			Y	Y		Y	Y	Y									Y	Y		Y	Y																							
12264	A_23_P347432	DVL1			Y	Y		Y		Y								Y	Y	Y	Y	Y	Y	Y	Y	Y	Y				Y															
16448	A_23_P55342	DVL2			Y	Y		Y		Y									Y	Y		Y																								
6073	A_23_P218884	DVL3																		Y																										
19046	A_23_P93737	DYNC1I1							Y	Y									Y											Y						Y										
1799	A_23_P154108	DYNC1I2			Y	Y	Y	Y		Y	Y	Y							Y	Y	Y		Y				Y																			

7649	A_23_P69141	DYNC1LI1			Y	Y		Y	Y	Y										Y			Y				Y	Y	Y	Y						Y										
20455	A_23_P147397	DYNC2H1	Y	Y	Y	Y	Y	Y	Y	Y	Y	Y	Y	Y				Y	Y	Y		Y	Y	Y			Y	Y	Y	Y						Y										
22108	A_23_P79168	DYNC2LI1			Y	Y	Y	Y	Y	Y	Y	Y	Y	Y				Y	Y	Y		Y	Y	Y			Y	Y	Y	Y						Y										
11764	A_23_P54992	DYNLL2			Y	Y		Y		Y									Y																											
10263	A_23_P94840	DYNLRB2			Y	Y		Y	Y	Y	Y	Y	Y	Y				Y	Y	Y		Y	Y	Y			Y	Y	Y	Y						Y										
20354	A_23_P73577	DYNLT3			Y	Y		Y		Y									Y	Y							Y			Y						Y										
20458	A_23_P211126	DYRK1A			Y	Y	Y	Y		Y	Y	Y							Y	Y			Y					Y																		
9961	A_23_P90297	DYRK1B			Y	Y		Y	Y	Y										Y		Y	Y				Y																			
18662	A_23_P204048	DYRK2	Y		Y										Y					Y			Y																							
1445	A_23_P12282	DYRK3							Y	Y													Y							Y						Y										
20587	A_23_P87899	DYRK4				Y			Y	Y	Y	Y	Y	Y																Y						Y										
202	A_23_P39925	DYSF	Y	Y	Y	Y		Y	Y	Y	Y	Y	Y	Y						Y			Y				Y	Y	Y	Y						Y										
12975	A_23_P37505	DYX1C1			Y	Y	Y	Y	Y	Y	Y	Y	Y	Y				Y	Y	Y		Y	Y	Y			Y	Y	Y	Y						Y										
15108	A_23_P14157	DZIP1							Y	Y	Y	Y	Y	Y				Y	Y	Y				Y						Y						Y										
2263	A_23_P435051	DZIP3			Y	Y	Y	Y	Y	Y	Y	Y	Y	Y					Y	Y							Y	Y	Y	Y						Y										
14066	A_23_P144165	DZIP3			Y	Y	Y	Y		Y	Y	Y	Y	Y					Y	Y		Y	Y				Y	Y	Y	Y																
4357	A_23_P166742	EAF1																Y																												
1360	A_23_P252201	EAF2	Y	Y	Y				Y	Y	Y	Y	Y	Y													Y			Y						Y										
15902	A_23_P128783	EAPP			Y													Y	Y	Y			Y	Y			Y																			
8031	A_23_P152353	EARS2	Y		Y	Y	Y	Y	Y	Y	Y	Y	Y	Y									Y				Y																			
20559	A_23_P135903	EBF1																		Y			Y																							
14302	A_23_P31829	EBF2				Y																																								
2744	A_23_P103631	EBNA1BP2																		Y			Y																							
3912	A_23_P115762	ECD			Y	Y	Y	Y	Y	Y	Y	Y	Y	Y						Y							Y																			
774	A_23_P200585	ECE1			Y																						Y																			
883	A_23_P92261	ECE2			Y	Y	Y	Y	Y	Y	Y	Y	Y	Y				Y	Y	Y		Y		Y			Y	Y	Y	Y						Y										
3637	A_23_P257786	ECE2			Y	Y		Y		Y	Y	Y	Y	Y					Y	Y							Y			Y						Y										
521	A_23_P131556	ECEL1			Y	Y		Y		Y									Y	Y			Y				Y			Y						Y										
13490	A_23_P153848	ECH1			Y													Y	Y	Y				Y																						
20387	A_23_P200203	ECHDC2																Y	Y	Y			Y	Y																						
5647	A_23_P104362	ECHS1																		Y			Y				Y			Y						Y										
6511	A_23_P149180	ECM1							Y	Y																																				
9438	A_23_P303671	ECM2																										Y																		
22355	A_23_P216430	ECM2																		Y																										
10650	A_23_P72654	ECSM2	Y		Y														Y				Y				Y																			
5958	A_23_P34045	EDA			Y		Y				Y								Y	Y		Y	Y				Y	Y	Y	Y						Y										
4250	A_23_P171132	EDA2R																			Y																									
15429	A_23_P503233	EDARADD	Y		Y	Y	Y	Y	Y	Y	Y	Y	Y	Y				Y	Y	Y	Y	Y	Y	Y	Y	Y	Y	Y	Y	Y	Y	Y								Y						
18722	A_23_P51621	EDARADD				Y			Y	Y																		Y		Y						Y										
4599	A_23_P323880	EDC4																	Y				Y																							
20487	A_23_P106641	EDC4							Y	Y	Y	Y	Y	Y													Y			Y						Y										
15674	A_23_P40975	EDEM1	Y	Y	Y				Y	Y																	Y	Y	Y	Y						Y										
320	A_23_P166188	EDEM2			Y	Y		Y		Y								Y	Y	Y	Y	Y	Y	Y	Y	Y	Y				Y															
2598	A_23_P200330	EDEM3																										Y		Y						Y										
6246	A_23_P10281	EDIL3																	Y																											
22457	A_23_P312150	EDN2																												Y						Y										
20904	A_23_P17438	EDN3																	Y			Y																								
4866	A_23_P7337	EDNRA			Y													Y		Y		Y	Y				Y																			
15787	A_23_P76159	EEA1			Y														Y	Y		Y																								
22407	A_23_P64761	EEA1																	Y																											
8483	A_23_P31838	EEF1D			Y	Y		Y	Y	Y	Y	Y	Y	Y														Y		Y						Y										
17644	A_23_P311039	EEF1DP3																		Y																										
14609	A_23_P140786	EEF2K																		Y			Y				Y																			
3172	A_23_P144202	EEFSEC			Y		Y				Y								Y								Y			Y						Y										
7823	A_23_P333498	EEPD1																									Y																			
3846	A_23_P146294	EFCAB1			Y																		Y				Y																			
7784	A_23_P149476	EFCAB2			Y	Y		Y	Y	Y									Y	Y							Y	Y	Y	Y						Y										
16944	A_23_P68978	EFCAB6			Y	Y		Y		Y	Y	Y	Y	Y													Y																			
17504	A_23_P24414	EFEMP2																		Y		Y	Y																							
4816	A_23_P76598	EFHA1			Y	Y	Y	Y	Y	Y	Y	Y	Y	Y				Y		Y			Y				Y	Y	Y	Y						Y										
20314	A_23_P214291	EFHC1	Y	Y	Y	Y	Y	Y	Y	Y	Y	Y	Y	Y				Y	Y	Y		Y	Y	Y			Y	Y	Y	Y			Y			Y										
114	A_23_P45338	EFHC2																Y	Y	Y				Y																						
11711	A_23_P33947	EFHC2			Y	Y		Y		Y	Y	Y	Y	Y				Y	Y	Y		Y	Y	Y			Y	Y	Y	Y																
1739	A_23_P154338	EFHD1			Y	Y		Y		Y																				Y						Y										
2108	A_23_P23443	EFHD2				Y													Y	Y			Y					Y																		
6642	A_23_P254512	EFNA1																		Y	Y		Y																							
2060	A_23_P119533	EFNA2			Y	Y	Y	Y	Y	Y	Y	Y	Y	Y						Y		Y	Y				Y	Y	Y	Y						Y										
21782	A_23_P149029	EFNA3			Y	Y		Y		Y									Y	Y		Y					Y	Y	Y	Y						Y										
9659	A_23_P322	EFNA4			Y	Y		Y		Y																	Y	Y	Y	Y																
13419	A_23_P66457	EFNB3			Y				Y	Y																	Y			Y						Y										
17208	A_23_P433588	EFNB3			Y	Y		Y		Y										Y							Y																			
8058	A_23_P152055	EFTUD1	Y	Y	Y	Y	Y	Y	Y	Y	Y	Y	Y	Y				Y	Y	Y		Y	Y	Y			Y	Y	Y	Y	Y	Y	Y	Y	Y	Y										
542	A_23_P155979	EGF																		Y																										
6698	A_23_P33996	EGFL6			Y				Y	Y																																				
14488	A_23_P123785	EGFL7			Y	Y		Y		Y										Y	Y	Y																								
2979	A_23_P215798	EGFR			Y	Y		Y		Y										Y							Y																			
9544	A_23_P343935	EGLN1																										Y		Y						Y										

2912	A_23_P162954	EGLN3				Y																																								
2981	A_23_P142986	EGR4							Y	Y																				Y							Y										
19580	A_23_P380318	EGR4				Y												Y				Y																								
9178	A_23_P119753	EHBP1				Y			Y	Y	Y	Y	Y	Y					Y	Y											Y							Y										
2207	A_23_P148114	EHBP1L1			Y	Y	Y	Y	Y	Y	Y	Y						Y	Y	Y	Y	Y	Y	Y	Y	Y	Y				Y																
19608	A_23_P52647	EHD1			Y	Y	Y	Y	Y	Y	Y	Y	Y	Y					Y	Y		Y					Y	Y	Y	Y																	
2561	A_23_P119390	EHD2			Y															Y			Y																							
16570	A_23_P79251	EHD3																		Y		Y	Y																							
17629	A_23_P163458	EHD4			Y	Y		Y		Y																																				
13994	A_23_P212099	EHHADH																												Y							Y										
9117	A_23_P217120	EHMT1			Y	Y		Y	Y	Y	Y	Y	Y	Y														Y																		
10884	A_23_P214638	EHMT2			Y	Y	Y	Y	Y	Y	Y	Y	Y					Y	Y	Y	Y	Y	Y	Y	Y	Y	Y				Y																
227	A_23_P54276	EID1			Y	Y		Y		Y																	Y	Y	Y	Y																	
10163	A_23_P365844	EID2			Y	Y		Y		Y										Y							Y	Y	Y	Y																	
2518	A_23_P368259	EID2B			Y	Y	Y	Y	Y	Y	Y	Y								Y			Y				Y	Y	Y	Y							Y										
4060	A_23_P45945	EIF1			Y																							Y																		
18567	A_23_P127468	EIF1AD			Y	Y		Y		Y										Y							Y	Y	Y	Y																	
10932	A_23_P35194	EIF1AX				Y														Y								Y																		
11455	A_23_P148629	EIF1AY				Y			Y	Y										Y								Y																		
20247	A_23_P44932	EIF2A			Y													Y	Y	Y	Y	Y	Y	Y	Y	Y	Y				Y																
7967	A_23_P135857	EIF2AK3			Y						Y		Y	Y						Y		Y	Y				Y																				
8797	A_23_P54540	EIF2AK4			Y															Y			Y																							
6521	A_23_P105313	EIF2B1			Y	Y	Y	Y	Y	Y	Y	Y															Y	Y	Y	Y																	
17589	A_23_P115046	EIF2B3			Y	Y		Y		Y																																				
21468	A_23_P112159	EIF2C2	Y	Y	Y	Y	Y	Y	Y	Y	Y	Y	Y	Y				Y	Y	Y	Y	Y	Y	Y	Y	Y	Y	Y	Y	Y	Y	Y	Y	Y	Y	Y										
15191	A_23_P97021	EIF2C3			Y	Y		Y	Y	Y	Y	Y	Y	Y						Y								Y																		
17437	A_23_P23171	EIF2C4																		Y		Y																								
9632	A_23_P105942	EIF2S1	Y	Y																																										
17218	A_23_P86550	EIF3A							Y	Y																		Y																		
12335	A_23_P77572	EIF3C																	Y	Y		Y	Y				Y																			
7647	A_23_P142774	EIF3F																									Y																			
1667	A_23_P38887	EIF3G			Y	Y	Y	Y		Y	Y	Y								Y		Y						Y																		
14128	A_23_P54297	EIF3J																		Y																									
6760	A_23_P150446	EIF3M																										Y																		
19835	A_23_P137103	EIF4A1			Y	Y		Y		Y										Y							Y																			
4912	A_23_P162679	EIF4B	Y	Y	Y	Y		Y	Y	Y																	Y	Y	Y	Y							Y										
16110	A_23_P165722	EIF4E2	Y	Y	Y	Y		Y	Y	Y	Y	Y	Y	Y	Y					Y							Y	Y	Y	Y							Y										
5870	A_23_P22224	EIF4EBP1			Y	Y	Y	Y	Y	Y	Y	Y	Y	Y				Y		Y		Y					Y	Y	Y	Y												Y				
11099	A_23_P218890	EIF4G1			Y													Y		Y	Y	Y	Y																							
18572	A_23_P394913	EIF4G1			Y	Y	Y	Y		Y	Y	Y						Y	Y	Y	Y	Y	Y	Y	Y	Y	Y				Y																
20765	A_23_P104892	EIF4G2				Y																						Y																		
3185	A_23_P126241	EIF4G3	Y	Y	Y	Y	Y	Y	Y	Y	Y	Y							Y	Y			Y				Y	Y	Y	Y							Y										
17698	A_23_P100925	EIF5A			Y	Y	Y	Y	Y	Y	Y	Y	Y	Y				Y	Y	Y	Y	Y	Y	Y	Y	Y	Y	Y	Y	Y	Y	Y	Y						Y				Y			Y	
C15682.6	A_23_P218608	EIF5B				Y													Y	Y		Y	Y																							
9145	A_23_P210939	EIF6			Y															Y	Y	Y	Y				Y			Y							Y										
10285	A_23_P2383	ELA1																									Y																			
18167	A_23_P130961	ELA2	Y	Y	Y	Y		Y	Y	Y										Y			Y				Y	Y	Y	Y																	
19571	A_23_P46238	ELA2A			Y																		Y				Y			Y							Y										
16610	A_23_P15942	ELAC1																											Y							Y										
19010	A_23_P388681	ELAVL1			Y	Y		Y	Y	Y																		Y																		
9204	A_23_P94571	ELAVL2			Y																																									
9259	A_23_P218499	ELAVL3			Y																							Y		Y							Y										
18884	A_23_P114755	ELAVL4							Y	Y																				Y							Y										
5979	A_23_P80310	ELFN2							Y	Y																																				
880	A_23_P27936	ELL				Y																																								
9960	A_23_P16537	ELL																		Y			Y																							
15358	A_23_P58506	ELL2																Y	Y								Y																			
580	A_23_P84369	ELMO1																					Y																							
971	A_23_P140827	ELMO3																	Y	Y		Y	Y				Y																			
5018	A_23_P305692	ELMOD2							Y	Y													Y																							
21911	A_23_P7282	ELMOD2			Y	Y	Y	Y	Y	Y	Y	Y	Y	Y					Y	Y		Y					Y	Y	Y	Y							Y								Y		
4539	A_23_P154256	ELMOD3							Y	Y																		Y		Y							Y										
9845	A_23_P215459	ELN			Y																																									
2001	A_23_P130689	ELOF1			Y	Y		Y		Y																																				
6646	A_23_P23380	ELOVL1			Y	Y	Y	Y	Y	Y	Y	Y	Y	Y				Y	Y	Y	Y	Y	Y	Y	Y	Y	Y	Y	Y	Y	Y	Y				Y										
15396	A_23_P418555	ELOVL2			Y																																									
20203	A_23_P251606	ELOVL2	Y	Y	Y														Y								Y																			
21204	A_23_P82065	ELOVL4			Y				Y	Y										Y							Y																			
617	A_23_P156498	ELOVL5	Y	Y	Y				Y	Y	Y	Y	Y	Y				Y		Y	Y		Y				Y			Y			Y			Y										
15811	A_23_P7361	ELOVL6			Y	Y		Y	Y	Y																	Y																			
692	A_23_P78438	ELP2	Y	Y	Y	Y	Y	Y	Y	Y	Y	Y	Y	Y				Y	Y	Y		Y	Y	Y			Y	Y	Y	Y							Y										
13711	A_23_P43034	ELP3			Y															Y			Y																							
21376	A_23_P24623	ELP4																	Y	Y																										
20181	A_23_P33664	ELSPBP1				Y												Y																												
21095	A_23_P62741	ELTD1	Y		Y										Y												Y																			
7845	A_23_P81474	EMB																	Y											Y							Y										
16630	A_23_P85171	EMD			Y	Y		Y		Y								Y	Y	Y		Y		Y			Y																			

5470	A_23_P87759	EMG1																					Y					Y																		
18684	A_23_P165848	EMILIN1			Y													Y	Y	Y	Y	Y	Y	Y	Y	Y	Y				Y															
20626	A_23_P27315	EMILIN2	Y	Y		Y			Y	Y	Y	Y	Y	Y														Y		Y						Y										
13673	A_23_P79816	EMILIN3			Y																						Y																			
2336	A_23_P205746	EML1			Y	Y		Y	Y	Y									Y											Y						Y										
18515	A_23_P127793	EML3			Y													Y	Y	Y	Y	Y	Y	Y	Y	Y					Y															
1743	A_23_P165896	EML4				Y																																								
13890	A_23_P143127	EML4				Y														Y																										
18198	A_23_P106682	EMP2							Y	Y	Y	Y	Y	Y																																
17726	A_23_P119362	EMP3			Y	Y	Y	Y	Y	Y	Y	Y	Y	Y				Y		Y	Y	Y	Y				Y				Y		Y	Y	Y											
14379	A_23_P27556	EMR1				Y																																								
3439	A_23_P502337	EMR2			Y	Y		Y	Y	Y																				Y						Y										
14471	A_23_P315122	EMX1			Y	Y		Y		Y										Y			Y				Y																			
1941	A_23_P44264	EMX2																	Y																											
8528	A_23_P56404	EN1							Y	Y	Y	Y	Y	Y						Y										Y						Y										
18406	A_23_P134433	EN2																		Y			Y																							
1745	A_23_P337767	ENAH																Y		Y																										
10087	A_23_P51397	ENAH				Y																																								
19169	A_23_P213424	ENC1																												Y						Y										
6561	A_23_P83266	ENDOG			Y	Y		Y	Y	Y									Y	Y		Y	Y				Y			Y						Y										
19915	A_23_P83328	ENG			Y	Y	Y	Y	Y	Y	Y	Y	Y	Y				Y	Y	Y	Y	Y	Y	Y	Y	Y	Y	Y	Y	Y	Y	Y	Y	Y	Y	Y										
12004	A_23_P137391	ENO1	Y	Y	Y	Y	Y	Y	Y	Y	Y	Y	Y	Y				Y	Y	Y	Y	Y	Y	Y	Y	Y	Y	Y	Y	Y	Y	Y								Y						
17382	A_23_P130149	ENO3	Y	Y	Y	Y	Y	Y		Y	Y	Y						Y		Y		Y					Y						Y													
1068	A_23_P15906	ENOSF1			Y				Y	Y																	Y																			
17256	A_23_P128575	ENOX1			Y	Y	Y	Y	Y	Y	Y	Y						Y	Y	Y			Y	Y			Y	Y	Y	Y						Y										
17112	A_23_P156880	ENPP1	Y	Y	Y	Y		Y	Y	Y	Y	Y	Y	Y				Y	Y	Y		Y	Y	Y			Y	Y	Y	Y						Y										
13579	A_23_P44215	ENPP3																		Y		Y																								
20336	A_23_P404536	ENPP3			Y				Y	Y																				Y						Y										
14702	A_23_P70318	ENPP4			Y				Y	Y									Y	Y	Y	Y	Y				Y	Y	Y	Y	Y	Y	Y	Y	Y											
1883	A_23_P214244	ENPP5			Y		Y		Y	Y	Y	Y							Y	Y	Y	Y	Y				Y				Y		Y	Y	Y											
17763	A_23_P410119	ENTHD1													Y																															
4414	A_23_P24260	ENTPD1	Y		Y										Y																															
12540	A_23_P212469	ENTPD3			Y	Y		Y		Y									Y								Y																			
5293	A_23_P216017	ENTPD4																	Y		Y	Y					Y																			
1505	A_23_P117576	ENTPD5																									Y																			
14313	A_23_P79999	ENTPD6																			Y	Y																								
2723	A_23_P86623	ENTPD7			Y																						Y																			
14606	A_23_P44342	ENTPD8			Y															Y			Y					Y		Y						Y										
3381	A_23_P82748	ENY2				Y																																								
9054	A_23_P121074	EOMES			Y				Y	Y									Y								Y			Y						Y										
5754	A_23_P253154	EP400			Y	Y	Y	Y	Y	Y	Y	Y	Y	Y				Y		Y							Y	Y	Y	Y						Y										
4869	A_23_P430120	EPAS1			Y																		Y																							
330	A_23_P154806	EPB41L1			Y																						Y	Y	Y	Y																
16497	A_23_P122717	EPB41L2																										Y																		
18041	A_23_P4536	EPB41L3																			Y																									
6245	A_23_P365210	EPB41L4A							Y	Y																																				
5202	A_23_P216556	EPB41L4B																		Y	Y	Y	Y																							
16045	A_23_P370514	EPB41L4B			Y	Y		Y		Y										Y		Y																								
6888	A_23_P209298	EPB41L5																					Y				Y																			
977	A_23_P219117	EPB49			Y																	Y					Y																			
1484	A_23_P93739	EPDR1			Y	Y		Y	Y	Y									Y	Y		Y	Y				Y	Y	Y	Y						Y										
6533	A_23_P29949	EPGN			Y				Y	Y																	Y			Y						Y										
11060	A_23_P157330	EPHA1			Y				Y	Y									Y																											
11875	A_23_P149281	EPHA2			Y	Y		Y		Y																																				
21828	A_23_P147046	EPHA3																									Y			Y						Y										
22110	A_23_P119899	EPHA4																												Y						Y										
8824	A_23_P411341	EPHA6							Y	Y	Y	Y	Y	Y													Y			Y						Y										
16816	A_23_P170116	EPHA6			Y																						Y																			
3577	A_23_P103199	EPHA8			Y	Y		Y	Y	Y									Y	Y		Y	Y				Y			Y						Y										
17253	A_23_P166994	EPHB1																												Y						Y										
6160	A_23_P200067	EPHB2			Y													Y		Y							Y																			
6236	A_23_P259220	EPHB3			Y				Y	Y	Y	Y	Y	Y				Y	Y	Y		Y		Y						Y						Y										
5559	A_23_P168443	EPHB4			Y													Y	Y	Y			Y	Y			Y																			
18294	A_23_P145935	EPHB6			Y	Y		Y		Y										Y		Y	Y				Y																			
21805	A_23_P34537	EPHX1			Y	Y		Y		Y	Y	Y	Y	Y				Y	Y	Y	Y	Y		Y			Y																			
6220	A_23_P8834	EPHX2			Y	Y	Y	Y		Y	Y	Y	Y	Y				Y		Y		Y	Y																					Y		
12243	A_23_P170600	EPM2A																					Y																							
18462	A_23_P59240	EPM2A							Y	Y										Y																										
15913	A_23_P415622	EPM2AIP1	Y	Y	Y	Y	Y	Y	Y	Y	Y	Y	Y	Y				Y	Y	Y		Y	Y	Y			Y	Y	Y	Y						Y								Y		
13974	A_23_P384072	EPN1				Y	Y				Y							Y		Y		Y	Y				Y	Y	Y	Y																
13028	A_23_P89310	EPN2	Y	Y	Y	Y		Y	Y	Y	Y	Y	Y	Y				Y				Y					Y	Y	Y	Y						Y										
22568	A_23_P130027	EPN3																Y		Y																										
22253	A_23_P145664	EPO																Y	Y											Y						Y										
17569	A_23_P38941	EPS15L1			Y													Y	Y	Y	Y	Y	Y	Y	Y	Y	Y				Y															
5488	A_23_P136347	EPS8				Y																					Y	Y	Y	Y																
787	A_23_P208779	EPS8L1							Y	Y																																				
5850	A_23_P10761	EPS8L2																			Y																									

17866	A_23_P136424	EPS8L2																	Y																								
10424	A_23_P160947	EPS8L3							Y	Y																																					
9999	A_23_P105794	EPSTI1	Y	Y	Y		Y				Y																																			
795	A_23_P135486	ERAF																										Y		Y					Y										
7432	A_23_P213499	ERAP1			Y													Y				Y						Y																			
784	A_23_P89249	ERBB2							Y	Y	Y	Y	Y	Y																															
21888	A_23_P30175	ERBB2IP			Y	Y	Y	Y	Y	Y	Y	Y	Y	Y					Y	Y	Y	Y	Y				Y	Y	Y	Y					Y										
2318	A_23_P203856	ERBB3																	Y																										
21079	A_23_P349416	ERBB3							Y	Y																			Y	Y	Y	Y					Y										
1767	A_23_P10276	ERBB4				Y																																							
18716	A_23_P107701	ERCC1			Y													Y	Y	Y		Y		Y			Y																			
6755	A_23_P130485	ERCC2			Y																Y																								
20366	A_23_P49087	ERCC4																		Y			Y				Y																			
6467	A_23_P117225	ERCC5			Y	Y	Y	Y	Y	Y	Y	Y	Y	Y				Y				Y						Y		Y					Y										
934	A_23_P1292	ERCC6			Y	Y		Y		Y							Y	Y	Y	Y	Y	Y	Y	Y	Y					Y															
10546	A_23_P58521	ERCC8																Y		Y			Y																							
22201	A_23_P120638	ERGIC3																Y	Y		Y	Y	Y																							
7941	A_23_P128734	ERH				Y																																							
8479	A_23_P129716	ERI2							Y	Y										Y						Y																			
14328	A_23_P202029	ERLIN1			Y				Y	Y										Y	Y					Y																			
19207	A_23_P169154	ERMP1			Y	Y	Y	Y	Y	Y	Y	Y	Y	Y				Y	Y	Y		Y	Y	Y			Y	Y	Y	Y					Y								Y		
11967	A_23_P436259	ERN1			Y	Y		Y	Y	Y									Y	Y								Y	Y	Y	Y																
988	A_23_P347623	ERO1LB							Y	Y			Y																																	
9635	A_23_P139925	ERP29							Y	Y																																			
15494	A_23_P46470	ERRFI1			Y	Y		Y	Y	Y											Y		Y	Y																							
2789	A_23_P47405	ESAM	Y	Y	Y	Y	Y	Y		Y	Y	Y			Y			Y	Y								Y	Y	Y	Y																
15844	A_23_P87961	ESD				Y												Y	Y	Y		Y		Y																					
5120	A_23_P154715	ESF1																		Y																									
12359	A_23_P32707	ESPL1																Y	Y	Y	Y	Y	Y	Y	Y	Y					Y		Y													
10625	A_23_P51117	ETAA1			Y	Y		Y	Y	Y									Y	Y	Y	Y	Y				Y	Y	Y	Y	Y	Y	Y	Y	Y	Y										
16849	A_23_P133573	ETF1			Y	Y	Y	Y	Y	Y	Y	Y	Y						Y	Y		Y					Y	Y	Y	Y					Y								Y		
753	A_23_P3402	ETFA				Y																																							
14301	A_23_P107653	ETFB				Y																	Y				Y																			
17432	A_23_P61447	ETFDH			Y		Y				Y		Y	Y				Y	Y	Y		Y	Y	Y			Y	Y	Y	Y	Y	Y	Y	Y	Y												
20924	A_23_P142294	ETHE1			Y	Y		Y	Y	Y									Y	Y																									
930	A_23_P204164	ETNK1			Y													Y		Y			Y																							
20132	A_23_P419239	ETNK1			Y															Y	Y		Y				Y																			
15061	A_23_P9831	ETV5																					Y																							
18898	A_23_P167051	EVC																	Y	Y																									
3052	A_23_P320225	EVC2			Y																		Y																							
15895	A_23_P78092	EVI2A	Y		Y																																							
17756	A_23_P66694	EVI2B	Y		Y				Y	Y																																			
16201	A_23_P34943	EVI5			Y	Y		Y		Y																		Y																	
21341	A_23_P419828	EVI5L			Y	Y		Y		Y							Y	Y	Y		Y		Y			Y			Y					Y										
18734	A_23_P129034	EVL				Y																						Y		Y					Y										
2576	A_23_P106963	EVPL			Y				Y	Y																		Y			Y					Y										
11043	A_23_P313394	EVX1			Y	Y		Y		Y											Y		Y	Y																							
16609	A_23_P19902	EVX1			Y	Y		Y	Y	Y											Y		Y	Y																							
15539	A_23_P91657	EWSR1			Y		Y				Y							Y	Y	Y	Y	Y		Y			Y																			
8942	A_23_P113972	EXOC1			Y	Y	Y	Y	Y	Y	Y	Y							Y	Y	Y		Y	Y	Y			Y	Y	Y	Y					Y						Y				
22109	A_23_P214350	EXOC2			Y	Y		Y	Y	Y																		Y	Y	Y	Y					Y										
16761	A_23_P93040	EXOC3			Y	Y		Y		Y							Y	Y	Y		Y	Y	Y			Y	Y	Y	Y																
238	A_23_P424734	EXOC3L			Y	Y		Y	Y	Y									Y	Y			Y																							
18400	A_23_P38860	EXOC3L2			Y	Y	Y	Y	Y	Y	Y	Y	Y	Y				Y	Y	Y		Y	Y	Y			Y	Y	Y	Y					Y										
19967	A_23_P135437	EXOC4																										Y		Y					Y										
6886	A_23_P413826	EXOC5																			Y																								
7999	A_23_P169576	EXOC6	Y	Y	Y	Y	Y	Y	Y	Y	Y	Y	Y	Y				Y	Y	Y	Y	Y	Y	Y	Y	Y	Y	Y	Y	Y	Y	Y				Y								Y		
174	A_23_P100553	EXOC7																		Y			Y																							
14638	A_23_P89162	EXOC7																	Y	Y			Y																							
15088	A_23_P430165	EXOC8			Y	Y	Y	Y	Y	Y	Y	Y	Y	Y				Y	Y	Y				Y			Y																			
16626	A_23_P250714	EXOG																	Y																										
19921	A_23_P62868	EXOSC10				Y			Y	Y									Y	Y	Y	Y	Y							Y					Y										
1868	A_23_P216390	EXOSC2																		Y																									
15813	A_23_P123905	EXOSC3							Y	Y														Y																							
18863	A_23_P32463	EXOSC4			Y	Y	Y	Y	Y	Y	Y	Y																	Y	Y	Y	Y					Y										
699	A_23_P89998	EXOSC5																	Y	Y		Y																								
10395	A_23_P100413	EXOSC6																					Y																							
6758	A_23_P81121	EXOSC9			Y	Y		Y		Y										Y								Y	Y	Y	Y																
1943	A_23_P403334	EXPH5																					Y																							
206	A_23_P43273	EXT1			Y	Y		Y	Y	Y	Y	Y	Y	Y						Y	Y										Y					Y						Y				
18324	A_23_P62999	EXTL1																Y		Y		Y	Y																							
11124	A_23_P114	EXTL2				Y			Y	Y									Y	Y								Y																	
3280	A_23_P123296	EXTL3			Y													Y	Y	Y		Y		Y			Y			Y					Y										
12823	A_23_P384405	EXTL3																Y	Y	Y		Y	Y	Y																					
9809	A_23_P502363	EYA1			Y		Y		Y	Y	Y	Y	Y	Y				Y									Y																			
3818	A_23_P57052	EYA2																		Y																									
15268	A_23_P500421	EYA2																		Y																									

21209	A_23_P74731	EYA3							Y	Y												Y								Y						Y										
2642	A_23_P145504	EYA4	Y	Y	Y	Y		Y		Y									Y	Y		Y					Y	Y	Y	Y						Y										
7497	A_23_P428219	EZH1																		Y																										
9499	A_23_P129982	EZH1																												Y						Y										
22308	A_23_P259641	EZH2	Y	Y																																										
16434	A_23_P19590	EZR																												Y						Y										
19716	A_23_P205172	F10																					Y																							
14127	A_23_P41314	F11																Y																												
7256	A_23_P167674	F12				Y												Y	Y			Y																								
17476	A_23_P93494	F13A1	Y												Y																															
12684	A_23_P46549	F13B																		Y																										
1488	A_23_P83478	F2	Y	Y	Y	Y	Y	Y	Y	Y	Y	Y	Y	Y				Y									Y																			
22301	A_23_P94879	F2			Y													Y									Y			Y						Y										
6301	A_23_P213562	F2R				Y																					Y	Y	Y	Y																
8777	A_23_P208421	F2RL3				Y																						Y																		
16057	A_23_P126782	F3			Y														Y								Y			Y						Y										
17169	A_23_P148768	F5																												Y						Y										
5492	A_23_P117298	F7			Y	Y	Y	Y	Y	Y	Y	Y	Y	Y				Y		Y		Y					Y	Y	Y	Y						Y										
12164	A_23_P217643	F8							Y	Y																																				
4101	A_23_P11262	F8A1				Y												Y	Y	Y				Y																						
253	A_23_P62406	F9				Y																						Y																		
8021	A_23_P49448	FA2H			Y				Y	Y	Y	Y	Y	Y																																
5826	A_23_P103223	FAAH	Y	Y	Y	Y		Y	Y	Y	Y	Y	Y	Y													Y			Y						Y										
8243	A_23_P113471	FAAH2			Y	Y		Y	Y	Y	Y	Y	Y	Y				Y	Y	Y	Y	Y	Y	Y	Y	Y	Y			Y	Y	Y	Y	Y	Y	Y										
21968	A_23_P79562	FABP1			Y	Y	Y	Y	Y	Y	Y	Y	Y	Y					Y								Y			Y						Y										
11689	A_23_P155949	FABP2			Y															Y							Y																			
5736	A_23_P59876	FABP5	Y	Y					Y	Y																				Y						Y										
15690	A_23_P43846	FABP6			Y	Y		Y	Y	Y																		Y																		
4028	A_23_P134139	FABP7			Y				Y	Y																	Y			Y						Y										
7788	A_23_P20215	FABP9																									Y																			
6253	A_23_P98581	FADS2																		Y																										
16685	A_23_P64399	FADS3							Y	Y	Y	Y	Y	Y													Y			Y						Y										
13188	A_23_P96853	FAF1																Y	Y			Y																								
21546	A_23_P118293	FAHD1			Y	Y	Y	Y	Y	Y	Y	Y								Y			Y				Y																			
5222	A_23_P5733	FAHD2A																		Y																										
3057	A_23_P138125	FAIM3			Y				Y	Y									Y	Y		Y	Y																							
7339	A_23_P402787	FAM100A																		Y		Y																								
1034	A_23_P112531	FAM102A			Y	Y		Y	Y	Y			Y							Y		Y					Y	Y	Y	Y						Y										
4566	A_23_P78289	FAM104A				Y														Y								Y																		
1414	A_23_P415061	FAM104B																									Y																			
16036	A_23_P84860	FAM107A																Y	Y	Y		Y		Y																						
10460	A_23_P149975	FAM107B																	Y																											
13584	A_23_P165080	FAM108A1			Y	Y	Y	Y	Y	Y	Y	Y						Y	Y	Y	Y	Y	Y	Y	Y	Y	Y			Y	Y	Y				Y								Y		
6146	A_23_P83149	FAM108B1																										Y																		
13443	A_23_P21912	FAM108C1							Y	Y																																				
19148	A_23_P386241	FAM110A							Y	Y	Y	Y	Y	Y																Y						Y										
12991	A_23_P395609	FAM110B			Y	Y		Y	Y	Y	Y	Y	Y	Y					Y								Y	Y	Y	Y						Y										
6905	A_23_P203332	FAM111A			Y	Y		Y	Y	Y								Y	Y	Y		Y		Y				Y		Y						Y										
2206	A_23_P17471	FAM113A			Y				Y	Y													Y																							
13833	A_23_P36888	FAM113B							Y	Y																				Y						Y										
16832	A_23_P132914	FAM114A1			Y														Y								Y																			
7730	A_23_P41912	FAM114A2			Y	Y		Y		Y													Y				Y	Y	Y	Y																
11644	A_23_P111786	FAM115A			Y	Y		Y		Y									Y	Y		Y					Y	Y	Y	Y						Y										
5450	A_23_P397980	FAM115C			Y				Y	Y																	Y			Y						Y										
22017	A_23_P319492	FAM118B			Y	Y		Y		Y																																				
9867	A_23_P209337	FAM119A			Y																						Y																			
19329	A_23_P313476	FAM120B			Y																						Y																			
7398	A_23_P409516	FAM122A																												Y						Y										
6948	A_23_P382688	FAM122B			Y																						Y																			
16640	A_23_P340188	FAM122C			Y													Y	Y	Y		Y		Y			Y	Y	Y	Y																
87	A_23_P308150	FAM123B			Y				Y	Y																				Y						Y										
8599	A_23_P8582	FAM126A			Y				Y	Y																				Y						Y										
4471	A_23_P85236	FAM127A			Y	Y	Y	Y	Y	Y	Y	Y						Y		Y		Y	Y				Y																			
10236	A_23_P62429	FAM127B			Y	Y	Y	Y	Y	Y	Y	Y	Y	Y				Y	Y	Y		Y	Y	Y			Y																			
5314	A_23_P165676	FAM128B																												Y						Y										
16128	A_23_P421326	FAM129A	Y	Y														Y	Y	Y		Y	Y	Y				Y		Y						Y										
17741	A_23_P217831	FAM129A	Y	Y														Y	Y	Y		Y		Y				Y		Y						Y										
9227	A_23_P417404	FAM129B			Y	Y		Y		Y									Y	Y		Y					Y																			
20471	A_23_P41204	FAM131A			Y		Y		Y	Y	Y	Y						Y	Y	Y	Y	Y	Y	Y	Y	Y	Y				Y													Y		
3231	A_23_P345808	FAM133A			Y																																									
11269	A_23_P315430	FAM134A	Y	Y	Y				Y	Y										Y			Y				Y						Y													
20951	A_23_P167599	FAM134B	Y	Y	Y	Y		Y	Y	Y								Y	Y	Y		Y	Y	Y			Y	Y	Y	Y						Y										
10981	A_23_P107195	FAM134C							Y	Y									Y																											
15830	A_23_P58862	FAM135A							Y	Y																		Y																		
10246	A_23_P108641	FAM136A																		Y	Y	Y																								
2412	A_23_P7137	FAM13A1			Y				Y	Y																																				
6811	A_23_P370651	FAM13A1																			Y																									

15763	A_23_P136460	FAM13B1			Y													Y	Y	Y				Y			Y	Y	Y	Y						Y										
20612	A_23_P45824	FAM151A				Y																	Y																							
9929	A_23_P9120	FAM154A			Y				Y	Y																	Y																			
22292	A_23_P22647	FAM155B			Y	Y	Y	Y		Y	Y	Y						Y	Y								Y	Y	Y	Y																
12741	A_23_P148519	FAM156A			Y	Y		Y	Y	Y																	Y	Y	Y	Y						Y										
19775	A_23_P365226	FAM161B			Y																																									
20719	A_23_P256542	FAM162A			Y													Y	Y	Y		Y	Y	Y			Y																			
4143	A_23_P145054	FAM162B																	Y																											
3689	A_23_P20363	FAM164A																		Y			Y																							
19243	A_23_P82051	FAM165B			Y				Y	Y											Y		Y				Y			Y						Y										
16202	A_23_P96950	FAM167B																Y	Y	Y				Y																						
13439	A_23_P44964	FAM171A1			Y				Y	Y	Y	Y	Y	Y						Y							Y			Y						Y										
463	A_23_P156067	FAM172A			Y				Y	Y	Y	Y	Y	Y																Y						Y										
13832	A_23_P133279	FAM173B																		Y	Y	Y	Y				Y																			
19750	A_23_P30283	FAM174A			Y	Y		Y	Y	Y								Y	Y	Y		Y	Y	Y			Y	Y	Y	Y						Y										
14282	A_23_P100001	FAM174B			Y				Y	Y	Y	Y	Y	Y													Y			Y						Y										
C12217	A_23_P253464	FAM175A																		Y																										
4774	A_23_P2935	FAM177A1				Y																																								
21150	A_23_P104282	FAM178A			Y	Y		Y		Y									Y	Y																										
17465	A_23_P258992	FAM178B			Y	Y		Y		Y								Y		Y		Y	Y				Y																			
10994	A_23_P3102	FAM179B			Y	Y	Y	Y	Y	Y	Y	Y	Y	Y					Y	Y		Y	Y				Y	Y	Y	Y	Y	Y	Y	Y	Y	Y				Y						
21994	A_23_P117380	FAM181A																					Y																							
10561	A_23_P167818	FAM184A			Y	Y		Y	Y	Y										Y																										
2364	A_23_P25445	FAM186B			Y	Y		Y		Y																	Y																			
17067	A_23_P304480	FAM19A5																	Y																											
4073	A_23_P352334	FAM20A	Y						Y	Y													Y																							
10468	A_23_P103923	FAM20B																		Y	Y		Y																							
16711	A_23_P250122	FAM20C			Y	Y		Y	Y	Y										Y	Y	Y					Y																			
5968	A_23_P363418	FAM26D																												Y						Y										
9860	A_23_P7827	FAM26F	Y	Y					Y	Y										Y								Y																		
16009	A_23_P146347	FAM29A				Y			Y	Y										Y			Y																							
2601	A_23_P140192	FAM30A			Y				Y	Y									Y								Y																			
7601	A_23_P50331	FAM32A	Y	Y	Y	Y		Y		Y					Y			Y		Y	Y	Y					Y																			
17687	A_23_P127275	FAM35A			Y	Y	Y	Y	Y	Y	Y	Y	Y	Y					Y	Y		Y	Y				Y	Y	Y	Y						Y										
8422	A_23_P12257	FAM36A																			Y																									
20120	A_23_P140736	FAM38A			Y	Y	Y	Y		Y	Y	Y							Y	Y		Y	Y				Y																			
10366	A_23_P130376	FAM38B	Y	Y	Y	Y		Y		Y					Y			Y	Y								Y	Y	Y	Y																
11818	A_23_P45485	FAM3A			Y	Y	Y	Y	Y	Y	Y	Y	Y	Y				Y	Y	Y		Y	Y	Y			Y	Y	Y	Y						Y										
6568	A_23_P166269	FAM3B			Y																																									
15344	A_23_P41145	FAM3D			Y																						Y	Y	Y	Y						Y										
11684	A_23_P422144	FAM43A			Y	Y		Y		Y								Y	Y	Y	Y	Y	Y	Y	Y	Y	Y				Y															
12803	A_23_P359052	FAM44A			Y	Y		Y		Y										Y							Y	Y	Y	Y																
4741	A_23_P345212	FAM44C																					Y																							
9172	A_23_P104351	FAM45A			Y	Y	Y	Y		Y	Y	Y						Y	Y	Y		Y		Y			Y	Y	Y	Y																
3914	A_23_P12196	FAM46B			Y																	Y																								
6570	A_23_P137751	FAM46C							Y	Y																	Y																			
17097	A_23_P427648	FAM47B																					Y																							
19565	A_23_P96445	FAM48B2																									Y	Y	Y	Y																
8693	A_23_P21560	FAM49A	Y	Y	Y	Y		Y	Y	Y					Y			Y	Y	Y			Y	Y			Y	Y	Y	Y						Y										
14261	A_23_P43255	FAM49B	Y	Y	Y	Y		Y		Y								Y		Y	Y																									
2627	A_23_P34107	FAM50A			Y	Y	Y	Y	Y	Y	Y	Y						Y	Y	Y	Y	Y	Y	Y	Y	Y	Y			Y	Y	Y				Y										
1048	A_23_P8240	FAM50B																Y	Y			Y																								
6703	A_23_P104509	FAM53B																					Y																							
12862	A_23_P167789	FAM53C				Y												Y		Y		Y						Y		Y						Y										
10961	A_23_P97195	FAM54B			Y																						Y																			
11679	A_23_P24543	FAM55A			Y																						Y			Y						Y										
21622	A_23_P132560	FAM55C			Y															Y																										
10805	A_23_P50000	FAM57A				Y			Y	Y	Y	Y	Y	Y																Y						Y										
21894	A_23_P163710	FAM57B																Y																												
12963	A_23_P404005	FAM58A	Y	Y	Y	Y	Y	Y	Y	Y	Y	Y	Y	Y													Y	Y	Y	Y						Y										
21115	A_23_P66948	FAM59A							Y	Y																		Y																		
21285	A_23_P35277	FAM5B																					Y																							
22458	A_23_P103812	FAM5C							Y	Y																	Y			Y						Y										
4170	A_23_P13663	FAM60A																		Y			Y				Y			Y						Y										
14300	A_23_P150852	FAM62A			Y													Y	Y	Y	Y	Y	Y	Y	Y	Y	Y				Y		Y													
18144	A_23_P8744	FAM62B							Y	Y								Y												Y						Y										
7609	A_23_P160546	FAM63A	Y	Y	Y	Y		Y		Y								Y		Y			Y				Y	Y	Y	Y																
C12575.6	A_23_P49878	FAM64A							Y	Y																				Y						Y										
11641	A_23_P10363	FAM65A			Y	Y		Y	Y	Y								Y	Y	Y		Y	Y	Y						Y						Y										
20833	A_23_P215009	FAM65B	Y		Y	Y	Y	Y	Y	Y	Y	Y						Y		Y			Y				Y			Y						Y										
19738	A_23_P84434	FAM70B			Y	Y		Y	Y	Y										Y			Y				Y																			
16946	A_23_P354288	FAM71A																Y	Y																											
18126	A_23_P378458	FAM71E2																												Y						Y										
4930	A_23_P134204	FAM71F1			Y	Y	Y	Y	Y	Y	Y	Y	Y	Y				Y									Y	Y	Y	Y														Y		
13445	A_23_P71858	FAM73B																		Y	Y	Y	Y																							
17648	A_23_P406355	FAM76B			Y													Y	Y	Y		Y	Y	Y																						
12490	A_23_P3295	FAM81A			Y	Y		Y	Y	Y										Y																										

4376	A_23_P360354	FAM81B			Y	Y	Y	Y	Y	Y	Y	Y	Y	Y				Y	Y	Y		Y		Y			Y	Y	Y	Y						Y										
22298	A_23_P102160	FAM82A1																Y	Y	Y				Y																						
2738	A_23_P152087	FAM82A2							Y	Y								Y																												
5765	A_23_P94889	FAM82B																										Y																		
8511	A_23_P83492	FAM82B	Y	Y	Y				Y	Y																	Y																			
22552	A_23_P251916	FAM82B																												Y						Y										
5423	A_23_P323751	FAM83D	Y	Y	Y	Y		Y	Y	Y								Y	Y	Y				Y			Y	Y	Y	Y						Y										
11686	A_23_P343963	FAM83F			Y	Y		Y		Y			Y						Y	Y								Y																		
6086	A_23_P118509	FAM83G																		Y										Y						Y										
16733	A_23_P428748	FAM84B			Y													Y	Y	Y		Y		Y			Y																			
3290	A_23_P124962	FAM89B			Y															Y			Y																							
14793	A_23_P133650	FAM8A1			Y	Y		Y		Y								Y	Y	Y		Y	Y	Y			Y	Y	Y	Y						Y										
11138	A_23_P151061	FAM90A1																Y																												
14119	A_23_P302103	FAM92A1							Y	Y									Y											Y						Y										
21693	A_23_P37636	FAM96A	Y	Y	Y																																									
13656	A_23_P77455	FAM96B			Y	Y	Y	Y	Y	Y	Y	Y	Y	Y				Y	Y	Y	Y	Y	Y	Y	Y	Y	Y	Y	Y	Y	Y	Y	Y	Y	Y	Y										
11583	A_23_P369975	FAM98C							Y	Y									Y											Y						Y										
C10129.9	A_23_P206441	FANCA							Y	Y								Y	Y	Y		Y		Y			Y	Y	Y	Y						Y										
15323	A_23_P32021	FANCC																									Y																			
1982	A_23_P143994	FANCD2																Y	Y	Y		Y	Y	Y																						
2656	A_23_P42335	FANCE																Y																												
13652	A_23_P12896	FANCF	Y		Y	Y	Y	Y	Y	Y	Y	Y	Y	Y				Y	Y	Y	Y	Y	Y	Y	Y	Y	Y	Y	Y	Y	Y	Y				Y										
45	A_23_P131383	FANCL			Y	Y	Y	Y	Y	Y	Y	Y						Y	Y	Y		Y	Y	Y			Y	Y	Y	Y												Y				
18173	A_23_P115785	FANK1	Y	Y	Y	Y		Y	Y	Y	Y	Y	Y	Y					Y	Y	Y	Y	Y				Y	Y	Y	Y						Y										
9060	A_23_P56746	FAP																		Y		Y																								
14910	A_23_P90849	FARP2			Y													Y																												
5652	A_23_P33720	FARS2			Y																																									
13638	A_23_P78682	FARSA				Y																																								
4842	A_23_P369815	FASLG	Y	Y																							Y																			
9794	A_23_P44132	FASN			Y				Y	Y								Y									Y																			
12631	A_23_P215140	FASTK			Y	Y		Y		Y								Y	Y	Y		Y	Y	Y																						
14804	A_23_P28590	FASTKD1							Y	Y										Y			Y																							
8059	A_23_P209649	FASTKD2			Y	Y		Y	Y	Y								Y		Y			Y					Y		Y						Y										
12166	A_23_P58489	FASTKD3			Y															Y	Y	Y	Y				Y																			
3849	A_23_P380724	FASTKD5			Y													Y		Y																										
14332	A_23_P69586	FAT1			Y				Y	Y	Y	Y	Y	Y									Y				Y			Y						Y										
10038	A_23_P18649	FAT4			Y	Y		Y		Y										Y							Y	Y	Y	Y																
10315	A_23_P127438	FAU			Y				Y	Y																	Y																			
9207	A_23_P78888	FBL																		Y																										
14516	A_23_P23454	FBLIM1			Y				Y	Y								Y		Y		Y					Y																			
132	A_23_P433016	FBLN1			Y	Y		Y		Y								Y	Y	Y	Y	Y	Y	Y	Y	Y					Y															
5847	A_23_P143981	FBLN2																					Y																							
6591	A_23_P65678	FBN1				Y													Y			Y						Y																		
16372	A_23_P257111	FBP1			Y				Y	Y																	Y			Y						Y										
6550	A_23_P83916	FBXL11																	Y	Y																										
7556	A_23_P379068	FBXL13					Y		Y	Y	Y	Y							Y	Y							Y			Y						Y										
18616	A_23_P36733	FBXL14			Y															Y			Y				Y	Y	Y	Y						Y										
20384	A_23_P423957	FBXL18			Y	Y		Y		Y																	Y																			
1441	A_23_P17955	FBXL2			Y		Y		Y	Y	Y	Y	Y	Y				Y	Y	Y	Y	Y	Y	Y	Y	Y	Y	Y	Y	Y	Y	Y														
8667	A_23_P167723	FBXL21			Y															Y		Y					Y	Y	Y	Y						Y										
1210	A_23_P140069	FBXL3			Y	Y		Y		Y										Y							Y	Y	Y	Y																
8623	A_23_P214739	FBXL4			Y	Y	Y	Y	Y	Y	Y	Y							Y	Y	Y	Y	Y				Y	Y	Y	Y						Y										
21908	A_23_P213247	FBXL5			Y	Y	Y	Y	Y	Y	Y	Y	Y	Y				Y	Y	Y		Y		Y			Y			Y						Y										
5056	A_23_P43296	FBXL6			Y	Y	Y	Y	Y	Y	Y	Y						Y	Y	Y	Y	Y	Y	Y	Y	Y	Y			Y	Y	Y				Y										
8690	A_23_P144827	FBXL7	Y	Y	Y	Y	Y	Y	Y	Y	Y	Y						Y	Y	Y		Y	Y	Y			Y	Y	Y	Y						Y										
13247	A_23_P14995	FBXL8			Y	Y		Y	Y	Y									Y	Y		Y	Y				Y			Y						Y										
1480	A_23_P79416	FBXO11			Y	Y	Y	Y	Y	Y	Y	Y								Y	Y	Y					Y			Y						Y										
8860	A_23_P90814	FBXO11			Y	Y	Y	Y	Y	Y	Y	Y							Y	Y		Y	Y				Y	Y	Y	Y						Y										
15415	A_23_P342709	FBXO15			Y				Y	Y																	Y																			
21142	A_23_P168846	FBXO16			Y	Y		Y		Y										Y							Y			Y						Y										
15957	A_23_P12849	FBXO18			Y				Y	Y								Y	Y	Y	Y	Y		Y			Y																			
8932	A_23_P53736	FBXO21			Y	Y	Y	Y	Y	Y	Y	Y	Y	Y						Y							Y	Y	Y	Y						Y										
16298	A_23_P65870	FBXO22			Y																																									
5669	A_23_P70888	FBXO24				Y																																								
14902	A_23_P94159	FBXO25				Y												Y	Y	Y	Y	Y	Y	Y	Y	Y					Y															
7297	A_23_P418029	FBXO27			Y	Y	Y	Y	Y	Y	Y	Y															Y	Y	Y	Y						Y										
16538	A_23_P108281	FBXO27			Y	Y	Y	Y		Y	Y	Y															Y																			
1139	A_23_P202844	FBXO3	Y	Y	Y	Y	Y	Y	Y	Y	Y	Y							Y	Y		Y	Y				Y	Y	Y	Y																
1246	A_23_P256231	FBXO30				Y			Y	Y										Y								Y																		
19630	A_23_P89030	FBXO31			Y	Y		Y	Y	Y	Y	Y	Y	Y				Y	Y	Y				Y				Y		Y						Y										
4263	A_23_P82814	FBXO32			Y				Y	Y										Y							Y																			
7937	A_23_P117527	FBXO34																			Y																									
10277	A_23_P432554	FBXO36			Y				Y	Y								Y		Y	Y	Y	Y				Y				Y		Y	Y	Y											
17929	A_23_P146811	FBXO38			Y	Y	Y	Y	Y	Y	Y	Y						Y	Y	Y	Y	Y	Y	Y	Y	Y	Y			Y	Y	Y				Y										
13293	A_23_P345098	FBXO39			Y																																									
20376	A_23_P58806	FBXO4																		Y	Y		Y																							
20610	A_23_P121013	FBXO40				Y																																								

11971	A_23_P160660	FBXO42			Y	Y	Y	Y	Y	Y	Y	Y							Y	Y	Y	Y					Y																				
21511	A_23_P97061	FBXO6	Y		Y	Y	Y	Y	Y	Y	Y	Y	Y	Y													Y	Y	Y	Y						Y								Y		
11200	A_23_P29225	FBXO7																Y		Y								Y																		
7020	A_23_P254120	FBXO9			Y						Y		Y	Y													Y			Y						Y										
9643	A_23_P214046	FBXW11			Y																						Y																			
18745	A_23_P104295	FBXW4																Y	Y																											
3823	A_23_P252608	FBXW4P1																												Y						Y										
21099	A_23_P434233	FBXW4P1																		Y		Y	Y																							
17308	A_23_P112397	FBXW5			Y	Y		Y	Y	Y	Y	Y	Y	Y				Y	Y	Y	Y	Y	Y	Y	Y	Y	Y	Y	Y	Y	Y	Y				Y										
16233	A_23_P48267	FBXW8			Y	Y	Y	Y	Y	Y	Y	Y	Y	Y				Y	Y	Y			Y	Y			Y	Y	Y	Y						Y										
3363	A_23_P16214	FBXW9			Y				Y	Y												Y					Y																			
11794	A_23_P208768	FCAR																									Y																			
14326	A_23_P160849	FCER1G	Y	Y	Y	Y	Y	Y		Y	Y	Y						Y									Y	Y	Y	Y						Y										
18239	A_23_P164773	FCER2			Y						Y		Y	Y																																
21617	A_23_P163130	FCF1							Y	Y																	Y			Y						Y										
4221	A_23_P32896	FCGBP	Y		Y													Y	Y	Y		Y		Y																						
15409	A_23_P21495	FCGBP	Y		Y													Y	Y	Y		Y		Y																						
906	A_23_P63395	FCGR1A	Y	Y	Y				Y	Y	Y	Y	Y	Y													Y	Y	Y	Y																
4645	A_23_P85716	FCGR2A	Y	Y	Y																																									
2426	A_23_P34650	FCGR2B	Y	Y	Y	Y	Y	Y	Y	Y	Y	Y	Y	Y				Y	Y	Y				Y			Y	Y	Y	Y						Y										
1741	A_23_P200728	FCGR3A	Y	Y	Y				Y	Y										Y							Y																			
5088	A_23_P55936	FCGRT																	Y	Y		Y	Y																							
6510	A_23_P157879	FCN1	Y	Y	Y		Y		Y	Y	Y	Y	Y	Y					Y	Y			Y				Y			Y						Y										
6941	A_23_P115246	FCN3			Y				Y	Y																																				
18386	A_23_P160751	FCRL2			Y				Y	Y																	Y	Y	Y	Y																
14129	A_23_P103803	FCRL3																												Y						Y										
21408	A_23_P358438	FCRL3	Y												Y																															
9815	A_23_P115192	FCRL4			Y	Y		Y		Y										Y	Y	Y	Y																							
9387	A_23_P201211	FCRL5			Y																						Y																			
20151	A_23_P46037	FCRLA																Y		Y		Y																								
18394	A_23_P350574	FCRLB			Y				Y	Y																	Y																			
1086	A_23_P71319	FDFT1							Y	Y																																				
6818	A_23_P23428	FDPS			Y	Y		Y		Y	Y	Y	Y	Y						Y		Y					Y	Y	Y	Y																
1161	A_23_P357780	FDX1			Y	Y		Y		Y																				Y						Y										
8151	A_23_P4685	FDX1L			Y	Y		Y		Y										Y		Y					Y	Y	Y	Y						Y										
4821	A_23_P89789	FECH			Y	Y		Y	Y	Y								Y		Y							Y																			
19209	A_23_P142277	FEM1A			Y													Y	Y	Y	Y	Y	Y	Y	Y	Y	Y				Y															
9149	A_23_P151970	FEM1B			Y																																									
2388	A_23_P41697	FEM1C				Y			Y	Y	Y	Y	Y	Y						Y			Y				Y																			
3029	A_23_P417237	FEM1C				Y																																								
12723	A_23_P87192	FEN1																		Y	Y	Y																								
17606	A_23_P124570	FER							Y	Y									Y																											
7850	A_23_P210956	FER1L4																	Y											Y						Y										
13087	A_23_P80048	FER1L4			Y																						Y			Y						Y										
6145	A_23_P422849	FERD3L			Y	Y	Y	Y		Y	Y	Y															Y	Y	Y	Y																
22365	A_23_P131935	FERMT1				Y			Y	Y																	Y			Y						Y										
16466	A_23_P64038	FERMT3	Y		Y	Y	Y	Y	Y	Y	Y	Y			Y				Y	Y	Y		Y				Y	Y	Y	Y	Y	Y	Y	Y	Y											
424	A_23_P14762	FES			Y	Y		Y	Y	Y	Y	Y	Y	Y				Y	Y	Y		Y	Y	Y			Y	Y	Y	Y						Y										
19592	A_23_P144126	FETUB			Y																						Y																			
11734	A_23_P202881	FEZ1																					Y																							
16343	A_23_P20158	FEZF1																Y	Y	Y		Y		Y																						
8881	A_23_P57925	FEZF2			Y																									Y						Y										
12237	A_23_P39356	FFAR1			Y						Y		Y	Y													Y			Y						Y										
4167	A_23_P50749	FFAR3							Y	Y																				Y						Y										
17591	A_23_P56239	FFAR3			Y	Y	Y	Y		Y	Y	Y								Y			Y				Y	Y	Y	Y						Y								Y		
9263	A_23_P114011	FGB																									Y																			
13333	A_23_P300852	FGD2	Y		Y				Y	Y																	Y	Y	Y	Y																
2829	A_23_P73220	FGD6			Y		Y		Y	Y	Y	Y							Y	Y							Y																			
22006	A_23_P377751	FGF10			Y														Y								Y																			
18427	A_23_P211727	FGF12																				Y																								
5729	A_23_P136763	FGF14																					Y				Y																			
8898	A_23_P254896	FGF16			Y																						Y																			
15676	A_23_P52714	FGF19			Y																						Y																			
22250	A_23_P218918	FGF2							Y	Y																																				
22509	A_23_P101564	FGF21							Y	Y																				Y						Y										
5612	A_23_P153878	FGF22																				Y																								
11881	A_23_P117095	FGF23			Y																																									
19188	A_23_P113204	FGF3																		Y		Y																								
10443	A_23_P14621	FGF7			Y														Y	Y	Y																									
2747	A_23_P105803	FGF9	Y												Y																															
1839	A_23_P30126	FGFBP1			Y				Y	Y								Y	Y	Y				Y						Y						Y										
18489	A_23_P41528	FGFBP2			Y																									Y						Y										
3284	A_23_P417994	FGFBP3																Y																												
554	A_23_P397274	FGFR1				Y																																								
14887	A_23_P145134	FGFR1OP			Y																Y																									
2532	A_23_P202334	FGFR2			Y	Y		Y	Y	Y													Y				Y																			
4358	A_23_P212832	FGFR3																				Y																								

9286	A_23_P500501	FGFR3			Y	Y		Y	Y	Y	Y	Y	Y	Y				Y	Y	Y		Y	Y	Y			Y			Y						Y										
10662	A_23_P92754	FGFR4			Y													Y	Y	Y	Y	Y	Y	Y	Y	Y	Y				Y															
5440	A_23_P148088	FGG				Y																																								
8177	A_23_P20484	FGL1																					Y																							
12194	A_23_P42969	FGL2	Y	Y	Y										Y												Y																			
19307	A_23_P103932	FGR	Y		Y																																									
12905	A_23_P125164	FHIT	Y	Y	Y																						Y																			
9732	A_23_P108751	FHL2							Y	Y										Y							Y			Y						Y										
16243	A_23_P114839	FHL3																Y		Y		Y																								
3705	A_23_P42358	FHL5							Y	Y																																				
8804	A_23_P37776	FHOD1			Y																		Y				Y																			
15081	A_23_P153107	FHOD3			Y				Y	Y	Y	Y	Y	Y				Y	Y								Y			Y						Y										
22563	A_23_P112187	FIBCD1																										Y																		
13481	A_23_P13018	FIBP																									Y																			
13736	A_23_P128246	FICD																												Y						Y										
6773	A_23_P145541	FIG4				Y																						Y																		
5853	A_23_P215208	FIGNL1				Y																																								
1290	A_23_P58337	FIP1L1			Y																																									
15232	A_23_P20183	FIS1				Y																																								
18150	A_23_P150693	FJX1			Y				Y	Y													Y																							
12494	A_23_P15727	FKBP10																									Y																			
16862	A_23_P215341	FKBP14																		Y																										
11908	A_23_P154665	FKBP1A			Y	Y		Y		Y									Y	Y		Y	Y				Y	Y	Y	Y																
6536	A_23_P142631	FKBP1B																												Y						Y										
22168	A_23_P161596	FKBP2							Y	Y																				Y						Y										
19142	A_23_P250874	FKBP6			Y																						Y			Y						Y										
17258	A_23_P50727	FKBP8			Y	Y	Y	Y	Y	Y	Y	Y	Y	Y				Y	Y	Y	Y	Y	Y	Y	Y	Y	Y	Y	Y	Y	Y	Y								Y						
13040	A_23_P164946	FKRP			Y													Y	Y	Y		Y	Y	Y																						
19794	A_23_P255827	FKSG2																												Y						Y										
19165	A_23_P165130	FKSG24			Y	Y	Y	Y	Y	Y	Y	Y								Y							Y			Y						Y										
4348	A_23_P33927	FKSG43							Y	Y																		Y		Y						Y										
21777	A_23_P216625	FKTN																		Y																										
18591	A_23_P34527	FLAD1			Y	Y		Y		Y								Y				Y					Y	Y	Y	Y																
16840	A_23_P138915	FLI1	Y						Y	Y										Y	Y																									
20202	A_23_P4425	FLII			Y	Y		Y	Y	Y								Y	Y	Y		Y	Y	Y			Y	Y	Y	Y																
9289	A_23_P6561	FLJ10213			Y	Y	Y	Y	Y	Y	Y	Y	Y	Y				Y	Y	Y	Y	Y		Y			Y	Y	Y	Y						Y										
3413	A_23_P99665	FLJ10357																		Y		Y	Y																							
8886	A_23_P427468	FLJ10404			Y																									Y						Y										
16526	A_23_P153521	FLJ10490				Y			Y	Y																		Y																		
5954	A_23_P77401	FLJ11151																	Y	Y																										
16102	A_23_P37545	FLJ11506			Y				Y	Y										Y			Y				Y																			
18187	A_23_P3922	FLJ11710			Y	Y		Y	Y	Y																		Y		Y						Y										
3791	A_23_P107578	FLJ11996			Y								Y														Y			Y						Y										
12234	A_23_P167464	FLJ12078			Y	Y	Y	Y	Y	Y	Y	Y						Y		Y		Y	Y										Y											Y		
21146	A_23_P116376	FLJ12529																		Y																										
6943	A_23_P54692	FLJ12547																	Y																											
10107	A_23_P348876	FLJ12616																			Y																									
19510	A_23_P321250	FLJ12986																										Y																		
17032	A_23_P110412	FLJ12993				Y																						Y																		
8963	A_23_P30163	FLJ13197	Y	Y	Y	Y		Y	Y	Y									Y	Y							Y	Y	Y	Y			Y	Y		Y										
2139	A_23_P23696	FLJ13310			Y	Y		Y		Y													Y				Y																			
5290	A_23_P122655	FLJ13744			Y	Y		Y		Y								Y	Y	Y		Y		Y			Y	Y	Y	Y						Y										
21568	A_23_P25056	FLJ13769			Y	Y		Y	Y	Y																		Y		Y						Y										
8867	A_23_P28574	FLJ14082			Y	Y		Y		Y								Y	Y	Y		Y	Y	Y							Y		Y	Y	Y											
292	A_23_P121	FLJ14100				Y																																								
16204	A_23_P77468	FLJ14327			Y	Y		Y	Y	Y	Y	Y	Y	Y														Y		Y						Y										
2349	A_23_P59602	FLJ20323			Y	Y	Y	Y	Y	Y	Y	Y	Y	Y				Y		Y		Y	Y				Y	Y	Y	Y																
3160	A_23_P57482	FLJ20464			Y	Y		Y		Y								Y	Y	Y		Y	Y	Y																						
14766	A_23_P72058	FLJ20674			Y				Y	Y			Y							Y		Y					Y																			
15809	A_23_P168368	FLJ21075			Y																						Y																			
6352	A_23_P11922	FLJ21272			Y	Y		Y	Y	Y									Y	Y	Y	Y						Y		Y						Y										
3814	A_23_P50597	FLJ21369																	Y																											
5753	A_23_P359497	FLJ22167				Y																																								
6997	A_23_P130604	FLJ22184			Y	Y		Y		Y										Y		Y	Y																							
21084	A_23_P87709	FLJ22662	Y																																											
22116	A_23_P68815	FLJ23185			Y				Y	Y								Y	Y			Y					Y																			
10374	A_23_P348253	FLJ23834			Y	Y		Y	Y	Y										Y			Y				Y	Y	Y	Y						Y										
338	A_23_P300136	FLJ23865																												Y						Y										
17725	A_23_P380266	FLJ23867																		Y			Y				Y																			
12544	A_23_P415633	FLJ25006			Y	Y		Y		Y																	Y																			
10644	A_23_P433183	FLJ25328			Y				Y	Y																	Y																			
8691	A_23_P342750	FLJ25694																												Y						Y										
13950	A_23_P398075	FLJ25770	Y															Y																												
6495	A_23_P390634	FLJ30594							Y	Y																				Y						Y										
10303	A_23_P343104	FLJ30901			Y																						Y																			
15582	A_23_P354326	FLJ31713			Y		Y				Y							Y									Y			Y						Y										
19266	A_23_P343859	FLJ32063			Y	Y		Y	Y	Y										Y		Y	Y				Y	Y	Y	Y						Y										

20627	A_23_P379768	FLJ32214				Y																																								
2323	A_23_P315310	FLJ32742																			Y						Y																			
20695	A_23_P399880	FLJ33996																		Y	Y		Y																							
7495	A_23_P43832	FLJ35220			Y	Y	Y	Y	Y	Y	Y	Y	Y	Y				Y	Y	Y		Y	Y	Y			Y	Y	Y	Y						Y										
9255	A_23_P22421	FLJ35220				Y														Y																										
9453	A_23_P342165	FLJ35700				Y			Y	Y																	Y			Y						Y										
18300	A_23_P331700	FLJ37078																Y			Y		Y																							
12133	A_23_P79758	FLJ38359			Y														Y	Y	Y		Y																							
22077	A_23_P313031	FLJ38379				Y																					Y																			
666	A_23_P141893	FLJ40125																Y	Y		Y																									
20207	A_23_P415675	FLJ40125				Y																																								
3146	A_23_P373708	FLJ40504							Y	Y	Y	Y	Y	Y																Y						Y										
1297	A_23_P96559	FLNA			Y													Y		Y	Y																									
4723	A_23_P211878	FLNB			Y																																									
6535	A_23_P214607	FLOT1																Y		Y																										
19427	A_23_P314526	FLOT1																		Y		Y																								
7740	A_23_P55318	FLOT2			Y	Y		Y		Y												Y					Y																			
14404	A_23_P99802	FLRT2																										Y																		
16867	A_23_P166109	FLRT3	Y	Y	Y				Y	Y	Y	Y	Y	Y						Y							Y			Y						Y										
13691	A_23_P99443	FLT3							Y	Y									Y																											
4352	A_23_P356070	FLT4																	Y																											
14748	A_23_P213394	FLT4			Y																						Y																			
9590	A_23_P140364	FLVCR2	Y	Y	Y				Y	Y					Y					Y							Y	Y	Y	Y						Y										
14962	A_23_P89012	FLYWCH1																				Y																								
1549	A_23_P89365	FMNL1			Y	Y	Y	Y	Y	Y	Y	Y						Y	Y	Y		Y	Y	Y			Y				Y		Y	Y	Y											
21773	A_23_P332744	FMNL2			Y				Y	Y	Y	Y	Y	Y																Y						Y										
4940	A_23_P138218	FMO2			Y														Y								Y																			
1825	A_23_P160990	FMO4	Y		Y				Y	Y	Y	Y	Y	Y													Y			Y						Y										
18220	A_23_P231	FMO5	Y	Y	Y				Y	Y								Y	Y	Y	Y	Y	Y	Y	Y	Y	Y			Y	Y	Y	Y	Y	Y	Y										
15897	A_23_P114883	FMOD			Y	Y		Y		Y																	Y																			
4706	A_23_P89215	FN3K			Y	Y		Y		Y	Y	Y	Y	Y									Y				Y	Y	Y	Y						Y										
10672	A_23_P77813	FN3KRP																		Y																										
1639	A_23_P201206	FNBP1L																		Y																										
10067	A_23_P417942	FNBP1L			Y	Y		Y	Y	Y			Y						Y	Y		Y	Y				Y	Y	Y	Y																
1780	A_23_P395595	FNBP4			Y	Y	Y	Y	Y	Y	Y	Y	Y	Y				Y	Y	Y		Y	Y	Y			Y	Y	Y	Y	Y	Y	Y	Y	Y	Y				Y						
22428	A_23_P150804	FNBP4																Y		Y		Y																								
15257	A_23_P25503	FNDC3A			Y	Y		Y		Y								Y	Y	Y		Y		Y			Y	Y	Y	Y																
19122	A_23_P253434	FNDC3B			Y	Y		Y	Y	Y																																				
2077	A_23_P16834	FNDC4							Y	Y								Y		Y		Y	Y				Y																			
16921	A_23_P420326	FNDC5																Y	Y	Y		Y		Y																						
12425	A_23_P156381	FNIP1				Y																						Y																		
7796	A_23_P25835	FNTB			Y																						Y																			
14593	A_23_P47622	FOLH1																									Y			Y						Y										
20490	A_23_P53176	FOLR1				Y			Y	Y	Y	Y	Y	Y																Y						Y										
15508	A_23_P47709	FOLR2	Y		Y													Y	Y	Y			Y	Y			Y																			
2172	A_23_P35769	FOLR4				Y			Y	Y																		Y		Y						Y										
13319	A_23_P161616	FOSL1				Y			Y	Y																	Y																			
17518	A_23_P322519	FOSL1				Y																																								
10832	A_23_P345581	FOSL2			Y	Y		Y	Y	Y										Y		Y	Y				Y																			
21033	A_23_P218553	FOSL2			Y	Y	Y	Y	Y	Y	Y	Y	Y	Y						Y		Y	Y				Y	Y	Y	Y						Y										
13687	A_23_P35875	FOXRED1			Y		Y				Y		Y					Y	Y	Y	Y	Y	Y	Y	Y	Y	Y			Y	Y	Y	Y	Y	Y	Y										
4561	A_23_P80305	FOXRED2			Y													Y	Y	Y	Y	Y	Y	Y	Y	Y	Y			Y	Y	Y				Y										
12185	A_23_P369944	FOXRED2																	Y	Y																										
20021	A_23_P9465	FPGS			Y	Y		Y	Y	Y																	Y	Y	Y	Y																
18183	A_23_P200030	FPGT				Y			Y	Y										Y			Y					Y																		
2700	A_23_P38795	FPR1	Y	Y	Y	Y	Y	Y		Y	Y	Y			Y			Y		Y			Y				Y	Y	Y	Y						Y										
5241	A_23_P55649	FPR2																		Y							Y			Y						Y										
7580	A_23_P208167	FPR3	Y	Y	Y	Y		Y		Y					Y			Y	Y	Y				Y			Y																			
21831	A_23_P259462	FRAS1				Y																																								
17607	A_23_P217049	FREQ	Y	Y	Y	Y		Y	Y	Y	Y	Y	Y	Y						Y							Y	Y	Y	Y						Y										
5046	A_23_P121772	FRG1			Y	Y	Y	Y		Y	Y	Y						Y	Y	Y		Y		Y			Y																			
1304	A_23_P133665	FRK																			Y																									
19694	A_23_P81717	FRMD1			Y																						Y			Y						Y										
3189	A_23_P22352	FRMD4A				Y																																								
10869	A_23_P25888	FRMD6			Y	Y	Y	Y	Y	Y	Y	Y						Y	Y	Y				Y			Y	Y	Y	Y						Y										
5538	A_23_P13002	FRMD8																										Y																		
13416	A_23_P169460	FRMPD1				Y																						Y																		
8441	A_23_P430785	FRS2																									Y																			
14947	A_23_P98980	FRS2				Y			Y	Y																		Y																		
6733	A_23_P167970	FRS3			Y		Y				Y		Y					Y	Y	Y		Y		Y			Y																			
22199	A_23_P105862	FRY	Y	Y	Y	Y		Y	Y	Y	Y	Y	Y	Y				Y	Y	Y		Y	Y	Y			Y	Y	Y	Y						Y								Y		
21034	A_23_P140337	FSCB			Y	Y		Y		Y																	Y																			
19002	A_23_P168531	FSCN1			Y													Y		Y	Y	Y					Y																			
12273	A_23_P93898	FSCN3			Y																																									
18008	A_23_P108699	FSHR			Y	Y		Y	Y	Y																	Y	Y	Y	Y																
13921	A_23_P353125	FSIP1			Y				Y	Y	Y	Y	Y	Y																Y						Y										
19720	A_23_P110531	FST			Y														Y	Y							Y																			

16627	A_23_P209160	FSTL3	Y	Y	Y																																									
1998	A_23_P92396	FSTL5			Y																																									
22326	A_23_P91552	FTCD																										Y																		
18577	A_23_P148410	FTHL17																				Y	Y																							
13370	A_23_P50498	FTL			Y	Y		Y		Y					Y					Y			Y				Y			Y						Y										
2037	A_23_P367596	FTMT			Y	Y		Y		Y										Y		Y					Y	Y	Y	Y																
14496	A_23_P113184	FTO	Y	Y	Y														Y	Y							Y	Y	Y	Y																
12443	A_23_P22473	FTSJ1			Y	Y	Y	Y	Y	Y	Y	Y	Y	Y				Y	Y	Y	Y	Y	Y	Y	Y	Y	Y	Y	Y	Y	Y	Y	Y			Y										
8503	A_23_P374149	FTSJD2			Y	Y		Y		Y								Y	Y	Y				Y																						
4929	A_23_P145408	FUCA2			Y													Y		Y	Y		Y				Y																			
13457	A_23_P375549	FUNDC1																		Y		Y	Y																							
14387	A_23_P3361	FURIN																Y			Y	Y																								
7782	A_23_P200080	FUSIP1			Y	Y		Y		Y										Y																										
14406	A_23_P107974	FUT2			Y																						Y	Y	Y	Y						Y										
13630	A_23_P165102	FUT3			Y	Y	Y	Y	Y	Y	Y	Y	Y	Y													Y	Y	Y	Y						Y										
14869	A_23_P360316	FUT3			Y	Y		Y	Y	Y	Y	Y	Y	Y													Y			Y						Y										
21669	A_23_P12965	FUT4							Y	Y	Y	Y	Y	Y														Y																		
19057	A_23_P101759	FUT5			Y	Y		Y	Y	Y																	Y																			
5641	A_23_P313632	FUT8			Y	Y		Y	Y	Y	Y	Y	Y	Y					Y	Y		Y					Y	Y	Y	Y																
12844	A_23_P19556	FUT9																				Y																								
11387	A_23_P4872	FUZ				Y																Y																								
15078	A_23_P373568	FUZ	Y	Y	Y																						Y																			
9955	A_23_P60520	FXN																		Y																										
17910	A_23_P4003	FXR2																		Y	Y	Y																								
14871	A_23_P108212	FXYD1																									Y																			
9848	A_23_P209043	FXYD3							Y	Y																																				
19650	A_23_P130995	FXYD5			Y	Y	Y	Y	Y	Y	Y	Y	Y	Y						Y	Y	Y	Y				Y	Y	Y	Y						Y										
10828	A_23_P150385	FXYD6																		Y			Y																							
2130	A_23_P119611	FXYD7			Y	Y		Y		Y													Y				Y	Y	Y	Y						Y										
8526	A_23_P122127	FYB																Y												Y						Y										
6069	A_23_P212339	FYCO1	Y	Y	Y	Y	Y	Y	Y	Y	Y	Y	Y	Y				Y	Y	Y		Y	Y	Y			Y	Y	Y	Y	Y	Y	Y	Y	Y	Y										
18256	A_23_P502142	FYN	Y						Y	Y					Y					Y			Y				Y						Y													
21886	A_23_P80827	FYTTD1																		Y																										
99	A_23_P203972	FZD10			Y				Y	Y								Y	Y	Y	Y	Y		Y																						
7916	A_23_P215922	FZD3			Y	Y		Y		Y	Y	Y	Y	Y						Y							Y																			
19406	A_23_P347471	FZD3			Y	Y		Y		Y										Y							Y																			
5085	A_23_P64617	FZD4							Y	Y	Y	Y	Y	Y																Y						Y								Y		
2778	A_23_P119826	FZD5			Y	Y		Y	Y	Y	Y	Y	Y	Y					Y	Y			Y				Y	Y	Y	Y						Y										
13493	A_23_P397999	FZD5			Y	Y	Y	Y	Y	Y	Y	Y	Y	Y						Y		Y					Y	Y	Y	Y						Y										
16562	A_23_P108437	FZD5							Y	Y													Y							Y						Y										
3957	A_23_P123276	FZD6																					Y																							
8791	A_23_P209449	FZD7							Y	Y																																				
20969	A_23_P1138	FZD8																					Y																							
10694	A_23_P59613	FZD9							Y	Y	Y	Y	Y	Y																																
7450	A_23_P142324	FZR1				Y																						Y																		
820	A_23_P74609	G0S2																									Y	Y	Y	Y																
11557	A_23_P306240	G30							Y	Y																																				
2588	A_23_P385014	G6PC			Y				Y	Y	Y	Y	Y	Y				Y	Y								Y	Y	Y	Y						Y										
4042	A_23_P207371	G6PC			Y																						Y																			
14396	A_23_P55174	G6PC3			Y	Y	Y	Y		Y	Y	Y								Y							Y			Y						Y										
618	A_23_P34093	G6PD			Y	Y		Y		Y									Y	Y							Y																			
2071	A_23_P153026	GAA																		Y			Y																							
19557	A_23_P255122	GAB3																Y												Y						Y										
10305	A_23_P27075	GABARAP			Y	Y	Y	Y	Y	Y	Y	Y																																		
7108	A_23_P118313	GABARAPL2																									Y																			
6372	A_23_P112275	GABBR2				Y			Y	Y								Y																												
3993	A_23_P125618	GABRA3			Y													Y		Y			Y				Y																			
6542	A_23_P252482	GABRA5																												Y						Y										
20188	A_23_P436618	GABRA5				Y			Y	Y																		Y		Y						Y										
12044	A_23_P125435	GABRB1																									Y																			
164	A_23_P14821	GABRB3				Y																						Y																		
20396	A_23_P10966	GABRB3																										Y																		
21523	A_23_P217818	GABRD			Y		Y		Y	Y	Y	Y						Y		Y							Y			Y						Y										
2786	A_23_P159775	GABRE			Y	Y		Y		Y										Y			Y				Y			Y						Y										
20270	A_23_P389496	GABRG1							Y	Y																				Y						Y										
16657	A_23_P213920	GABRG2																	Y																											
4773	A_23_P254159	GABRP			Y	Y		Y	Y	Y								Y	Y	Y		Y		Y						Y						Y										
15595	A_23_P328545	GABRP			Y	Y		Y	Y	Y	Y	Y	Y	Y				Y	Y	Y		Y	Y	Y						Y						Y										
20590	A_23_P256754	GABRR1																									Y																			
20637	A_23_P69024	GABRR3																												Y						Y										
6216	A_23_P209578	GAD1	Y	Y											Y																															
2490	A_23_P23221	GADD45A																Y		Y		Y																								
5373	A_23_P142506	GADD45B			Y	Y	Y	Y	Y	Y	Y	Y	Y	Y				Y		Y		Y	Y				Y	Y	Y	Y			Y			Y										
20762	A_23_P217196	GAGE4																		Y																										
20479	A_23_P155695	GAK			Y														Y	Y		Y	Y																							
9349	A_23_P374844	GAL			Y				Y	Y	Y	Y	Y	Y						Y	Y		Y				Y	Y	Y	Y						Y										
17927	A_23_P202624	GAL			Y				Y	Y	Y	Y	Y	Y									Y				Y																			

6527	A_23_P35830	GAL3ST3																		Y										Y						Y										
8911	A_23_P31242	GAL3ST4																											Y																	
16529	A_23_P25964	GALC																		Y																										
12390	A_23_P354154	GALE				Y			Y	Y																			Y		Y						Y										
20353	A_23_P160148	GALE							Y	Y																					Y						Y										
3279	A_23_P141126	GALK1			Y	Y	Y	Y	Y	Y	Y	Y						Y	Y	Y		Y	Y	Y			Y			Y						Y										
16961	A_23_P67971	GALM			Y	Y	Y	Y	Y	Y	Y	Y															Y	Y	Y	Y						Y										
15671	A_23_P383986	GALNAC4S-6ST			Y				Y	Y									Y																											
14651	A_23_P106562	GALNS			Y	Y	Y	Y		Y	Y	Y							Y	Y		Y					Y			Y						Y										
13368	A_23_P153137	GALNT1				Y													Y									Y																		
16565	A_23_P19102	GALNT10			Y	Y	Y	Y	Y	Y	Y	Y	Y	Y					Y	Y	Y	Y					Y	Y	Y	Y						Y										
4422	A_23_P8412	GALNT11							Y	Y																	Y			Y						Y										
654	A_23_P257731	GALNT12																					Y																							
15793	A_23_P116922	GALNT4			Y	Y		Y		Y																	Y	Y	Y	Y						Y										
17055	A_23_P413576	GALNT4																									Y	Y	Y	Y						Y										
5136	A_23_P204133	GALNT6			Y				Y	Y	Y	Y	Y	Y																Y						Y										
18941	A_23_P144384	GALNT7			Y		Y		Y	Y	Y	Y	Y	Y				Y	Y	Y	Y	Y	Y	Y	Y	Y	Y	Y	Y	Y	Y	Y				Y										
3691	A_23_P65101	GALNT8																					Y																							
8905	A_23_P76749	GALNTL1			Y																						Y																			
16815	A_23_P139418	GALNTL4			Y				Y	Y			Y					Y									Y	Y	Y	Y						Y										
5197	A_23_P429601	GALNTL5							Y	Y																																				
10393	A_23_P153155	GALR1																									Y																			
22097	A_23_P256663	GALR3			Y	Y		Y		Y										Y		Y	Y				Y																			
342	A_23_P108143	GAMT	Y	Y																																										
9777	A_23_P88873	GAN			Y																																									
2940	A_23_P399851	GANAB																		Y																										
20037	A_23_P13897	GAPDH			Y	Y		Y	Y	Y	Y	Y	Y	Y				Y	Y	Y	Y	Y	Y	Y	Y	Y		Y		Y	Y	Y				Y		Y				Y			Y	Y
21262	A_23_P67643	GAPDHS				Y			Y	Y								Y																												
9740	A_23_P18857	GAPT			Y															Y							Y																			
13720	A_23_P151569	GARNL1			Y	Y	Y	Y	Y	Y	Y	Y	Y	Y					Y	Y		Y	Y				Y	Y	Y	Y	Y	Y	Y	Y	Y	Y										
7248	A_23_P82361	GARS			Y																						Y																			
15049	A_23_P83134	GAS1			Y				Y	Y																				Y						Y										
22216	A_23_P32700	GAS2							Y	Y					Y			Y	Y	Y		Y		Y						Y						Y										
12123	A_23_P103044	GAS2L1				Y																																								
17897	A_23_P300239	GAS2L3							Y	Y																				Y						Y										
13773	A_23_P146917	GAS6																					Y																							
20117	A_23_P207258	GAS7			Y																						Y																			
20779	A_23_P206435	GAS8																	Y	Y		Y																								
8594	A_23_P159190	GAST							Y	Y	Y	Y	Y	Y																																
7640	A_23_P110022	GATA2			Y													Y	Y	Y				Y			Y																			
14967	A_23_P75056	GATA3	Y	Y	Y	Y		Y	Y	Y	Y	Y	Y	Y				Y	Y	Y		Y	Y	Y			Y	Y	Y	Y						Y										
14978	A_23_P82722	GATA4			Y				Y	Y										Y	Y		Y				Y																			
3113	A_23_P132048	GATA5				Y																																								
19542	A_23_P304450	GATA6																												Y						Y										
21778	A_23_P67008	GATA6																												Y						Y										
18161	A_23_P82509	GATAD1																			Y																									
17426	A_23_P131020	GATAD2A							Y	Y										Y	Y	Y	Y				Y	Y	Y	Y																
16453	A_23_P12320	GATAD2B																										Y		Y						Y										
14806	A_23_P76435	GATC																Y									Y	Y	Y	Y																
13603	A_23_P129064	GATM																Y		Y		Y																								
21930	A_23_P347643	GBA2																Y		Y	Y	Y																								
1890	A_23_P82669	GBAS				Y	Y				Y									Y	Y		Y																							
14493	A_23_P121082	GBE1																									Y			Y						Y										
3	A_23_P146576	GBGT1																		Y		Y	Y																							
1854	A_23_P26444	GBL			Y	Y		Y	Y	Y								Y	Y	Y		Y	Y	Y			Y			Y						Y										
130	A_23_P62890	GBP1	Y	Y					Y	Y										Y																										
5462	A_23_P85693	GBP2				Y																																								
14895	A_23_P51487	GBP3			Y	Y		Y		Y									Y	Y							Y																			
12475	A_23_P103496	GBP4			Y																		Y																							
21361	A_23_P74290	GBP5	Y	Y	Y		Y		Y	Y	Y	Y			Y					Y							Y																			
4002	A_23_P160817	GBP6			Y																						Y																			
14352	A_23_P130642	GBP6																												Y						Y										
7105	A_23_P97082	GBP7				Y																								Y						Y										
11214	A_23_P167349	GC				Y																																								
12000	A_23_P209373	GCC2				Y														Y		Y						Y																		
13328	A_23_P384090	GCC2			Y	Y		Y	Y	Y									Y	Y		Y	Y				Y	Y	Y	Y						Y										
4337	A_23_P90089	GCDH																		Y																										
16721	A_23_P254664	GCG																	Y		Y																									
21987	A_23_P15742	GCGR			Y																																									
16021	A_23_P163079	GCH1	Y	Y																																										
7838	A_23_P77328	GCHFR			Y				Y	Y										Y		Y	Y				Y																			
19460	A_23_P250206	GCK			Y													Y	Y	Y		Y	Y	Y			Y																			
18207	A_23_P145114	GCLC			Y																						Y																			
12138	A_23_P59285	GCM2			Y																						Y			Y						Y										
17369	A_23_P9232	GCNT1							Y	Y																				Y						Y										
14682	A_23_P420209	GCNT3																	Y																											
10923	A_23_P122077	GCNT4																	Y	Y																										

8313	A_23_P143258	GCNT7																		Y		Y																								
2243	A_23_P117752	GCOM1			Y															Y																										
18682	A_23_P117933	GCSH							Y	Y																																				
10432	A_23_P112352	GDA	Y	Y	Y	Y	Y	Y		Y	Y	Y	Y	Y								Y					Y	Y	Y	Y						Y						Y				
12280	A_23_P421587	GDAP1			Y		Y		Y	Y	Y	Y															Y																			
17393	A_23_P17356	GDAP1L1																											Y						Y									
22334	A_23_P11915	GDAP2																		Y																										
15507	A_23_P54763	GDE1	Y		Y	Y		Y	Y	Y																	Y			Y						Y									
3746	A_23_P209098	GDF1																		Y	Y	Y	Y																						
5639	A_23_P16523	GDF15																Y		Y										Y						Y									
10326	A_23_P61420	GDF3			Y													Y									Y																			
13336	A_23_P167585	GDF9			Y	Y		Y		Y																		Y																		
8311	A_23_P161446	GDI2			Y																																									
18360	A_23_P167683	GDNF																									Y																			
9997	A_23_P26503	GDPD3																	Y																											
4104	A_23_P87401	GDPD5																Y					Y																							
17854	A_23_P98844	GEFT																Y					Y																							
7116	A_23_P66872	GEMIN4																		Y																										
6241	A_23_P336513	GEMIN5				Y			Y	Y																		Y		Y						Y									
15105	A_23_P257482	GEMIN5			Y	Y	Y	Y		Y	Y	Y																Y																		
18119	A_23_P66593	GFAP																	Y								Y			Y						Y									
13019	A_23_P257365	GFI1																											Y						Y									
15443	A_23_P155288	GFM1				Y																																								
16520	A_23_P213431	GFM2							Y	Y	Y	Y	Y	Y					Y	Y		Y						Y																		
17695	A_23_P3574	GFOD2			Y	Y	Y	Y	Y	Y	Y	Y								Y		Y	Y				Y	Y	Y	Y						Y									
19594	A_23_P12686	GFRA1	Y	Y	Y	Y	Y	Y	Y	Y	Y	Y	Y	Y				Y		Y			Y				Y	Y	Y	Y						Y									
15530	A_23_P41992	GFRA3																Y	Y																											
8079	A_23_P166081	GFRA4																	Y				Y																							
15601	A_23_P132294	GGA1							Y	Y									Y																											
4313	A_23_P163732	GGA2																		Y	Y	Y																							
21849	A_23_P102292	GGCX																Y																												
5656	A_23_P134910	GGH									Y		Y	Y				Y																												
20647	A_23_P333552	GGN																										Y																		
19104	A_23_P38408	GGNBP2			Y	Y		Y		Y																																				
20054	A_23_P52189	GGPS1			Y	Y	Y	Y	Y	Y	Y	Y						Y	Y	Y	Y	Y	Y	Y	Y	Y					Y															
2584	A_23_P120809	GGT1			Y																							Y																		
12324	A_23_P166376	GGT5																Y	Y																											
16602	A_23_P66774	GGT6			Y	Y		Y		Y																		Y		Y						Y									
1113	A_23_P112452	GGTA1	Y	Y	Y	Y	Y	Y	Y	Y	Y	Y	Y	Y									Y				Y	Y	Y	Y						Y									
1674	A_23_P207160	GH2			Y				Y	Y											Y		Y				Y																			
10365	A_23_P207387	GHDC																				Y																								
8772	A_23_P104382	GHITM			Y	Y		Y		Y																	Y	Y	Y	Y																
21846	A_23_P40956	GHRL																									Y																			
11844	A_23_P109864	GHSR				Y			Y	Y									Y			Y						Y		Y						Y									
22337	A_23_P157055	GIGYF1																Y	Y	Y			Y	Y																						
18737	A_23_P142950	GIGYF2	Y	Y	Y	Y	Y	Y	Y	Y	Y	Y	Y	Y				Y	Y	Y	Y	Y	Y	Y	Y	Y	Y	Y	Y	Y	Y	Y	Y	Y	Y	Y										
10241	A_23_P31188	GIMAP1	Y		Y															Y			Y				Y																			
20808	A_23_P427023	GIMAP1	Y		Y		Y				Y							Y	Y	Y			Y	Y			Y			Y	Y	Y	Y	Y	Y	Y										
8781	A_23_P368681	GIMAP2	Y																																											
1158	A_23_P19790	GIMAP4			Y										Y				Y								Y																			
7368	A_23_P145631	GIMAP6	Y		Y										Y			Y	Y	Y			Y	Y			Y																			
1729	A_23_P8388	GIMAP7	Y	Y																																										
18043	A_23_P168388	GIMAP8	Y	Y	Y	Y	Y	Y	Y	Y	Y	Y						Y	Y	Y			Y	Y			Y	Y	Y	Y																
2781	A_23_P110611	GIN1																		Y																										
2232	A_23_P141459	GIP			Y																																									
19254	A_23_P130648	GIPC1			Y	Y	Y	Y	Y	Y	Y	Y	Y	Y				Y	Y	Y	Y	Y	Y	Y	Y	Y	Y	Y	Y	Y	Y	Y				Y									
20261	A_23_P130934	GIPC3			Y	Y	Y	Y	Y	Y	Y	Y								Y		Y						Y		Y						Y									
15204	A_23_P100912	GIT1			Y													Y	Y	Y		Y		Y			Y			Y			Y			Y									
3129	A_23_P501451	GIYD1																Y	Y	Y	Y	Y	Y	Y	Y	Y					Y															
22473	A_23_P93591	GJA1				Y			Y	Y																				Y						Y									
21673	A_23_P53815	GJA3				Y															Y							Y																		
3357	A_23_P1083	GJA4	Y		Y													Y	Y	Y			Y	Y			Y	Y	Y	Y																
19189	A_23_P371729	GJA5							Y	Y																				Y						Y									
19857	A_23_P148941	GJA5	Y	Y	Y	Y	Y	Y	Y	Y	Y	Y	Y	Y	Y			Y	Y	Y		Y	Y	Y			Y	Y	Y	Y																
5186	A_23_P23037	GJA8							Y	Y									Y																											
7880	A_23_P359609	GJA9			Y																							Y																		
20567	A_23_P74828	GJA9			Y	Y	Y	Y	Y	Y	Y	Y	Y	Y													Y	Y	Y	Y						Y								Y		
17199	A_23_P250446	GJB1			Y				Y	Y																	Y																			
1512	A_23_P204947	GJB2																			Y																									
18249	A_23_P35293	GJB5																Y	Y	Y		Y	Y	Y			Y	Y	Y	Y						Y									
14357	A_23_P2745	GJB6			Y	Y		Y		Y										Y																										
20506	A_23_P30983	GJB7																									Y			Y						Y									
10213	A_23_P428287	GJC1			Y																						Y	Y	Y	Y																
22035	A_23_P49724	GJC1				Y			Y	Y																																				
19617	A_23_P51729	GJC2																Y		Y			Y																							
7914	A_23_P59870	GJC3				Y			Y	Y										Y	Y		Y					Y		Y						Y									
16480	A_23_P427754	GJD3			Y	Y		Y		Y										Y		Y	Y				Y			Y						Y									

11113	A_23_P96556	GK			Y	Y		Y		Y									Y	Y	Y		Y	Y	Y				Y																		
20598	A_23_P258583	GK5			Y	Y		Y	Y	Y										Y		Y	Y																						
9376	A_23_P94546	GKAP1			Y	Y	Y	Y	Y	Y	Y	Y	Y	Y				Y	Y	Y		Y	Y	Y			Y	Y	Y	Y	Y	Y	Y	Y	Y	Y										
3265	A_23_P61318	GKN2							Y	Y																				Y						Y										
5474	A_23_P61531	GLB1			Y																																									
11523	A_23_P218574	GLB1L				Y																																								
19554	A_23_P82402	GLCCI1			Y				Y	Y	Y	Y	Y	Y				Y		Y		Y	Y							Y						Y						Y				
17029	A_23_P151870	GLCE			Y	Y	Y	Y	Y	Y	Y	Y	Y	Y					Y	Y	Y		Y				Y	Y	Y	Y						Y										
16605	A_23_P258367	GLE1																			Y																								
3090	A_23_P209241	GLI2			Y	Y	Y	Y	Y	Y	Y	Y								Y								Y	Y	Y	Y						Y										
4278	A_23_P111532	GLI3	Y	Y	Y	Y	Y	Y	Y	Y	Y	Y	Y	Y				Y	Y	Y	Y	Y	Y	Y	Y	Y	Y	Y	Y	Y	Y	Y				Y										
13904	A_23_P337141	GLI4			Y													Y	Y	Y	Y	Y				Y																			
4135	A_23_P203833	GLIPR1				Y													Y																									
21056	A_23_P364024	GLIPR1	Y	Y																										Y						Y										
11276	A_23_P317839	GLIPR1L1			Y																						Y			Y						Y										
12478	A_23_P71627	GLIPR2	Y	Y	Y	Y		Y	Y	Y																			Y						Y										
14399	A_23_P414913	GLIPR2	Y	Y							Y		Y	Y																Y						Y										
812	A_23_P323154	GLIS2																				Y						Y																	
20654	A_23_P43357	GLIS3				Y			Y	Y																	Y			Y						Y										
21493	A_23_P160742	GLMN																	Y																									
17594	A_23_P78269	GLOD4			Y																																									
8512	A_23_P156542	GLP1R			Y														Y								Y																			
22564	A_23_P156261	GLRA1			Y																										Y						Y										
22522	A_23_P257387	GLRA3				Y																									Y						Y										
1402	A_23_P125798	GLRA4			Y																										Y						Y										
13850	A_23_P213265	GLRB	Y	Y	Y						Y		Y	Y				Y	Y	Y		Y	Y	Y			Y																			
5102	A_23_P160503	GLRX2			Y	Y		Y		Y												Y					Y	Y	Y	Y						Y										
12227	A_23_P202227	GLRX3	Y	Y	Y												Y									Y																			
10430	A_23_P36416	GLS2	Y		Y	Y		Y	Y	Y	Y	Y	Y	Y						Y							Y	Y	Y	Y			Y													
1391	A_23_P209189	GLT25D1			Y	Y		Y	Y	Y	Y	Y	Y	Y				Y	Y	Y		Y	Y	Y			Y			Y						Y										
5576	A_23_P97610	GLT25D2																Y																											
2020	A_23_P258824	GLT6D1																									Y																			
6543	A_23_P132669	GLT8D1			Y	Y	Y	Y	Y	Y	Y	Y	Y	Y					Y	Y			Y				Y	Y	Y	Y	Y	Y	Y	Y	Y	Y										
15752	A_23_P25336	GLTP			Y												Y		Y			Y				Y																			
5156	A_23_P63281	GLTPD1			Y	Y	Y	Y	Y	Y	Y	Y						Y	Y	Y		Y	Y	Y			Y	Y	Y	Y						Y										
19691	A_23_P130773	GLTSCR1																	Y		Y	Y																							
12344	A_23_P39125	GLTSCR2			Y	Y		Y	Y	Y								Y										Y		Y						Y										
12924	A_23_P138665	GLUD1			Y	Y		Y	Y	Y	Y	Y	Y	Y					Y	Y							Y	Y	Y	Y						Y										
14033	A_23_P45361	GLUD2			Y	Y		Y	Y	Y	Y	Y	Y	Y												Y	Y	Y	Y						Y										
14866	A_23_P370666	GLYATL1							Y	Y	Y	Y	Y	Y				Y											Y						Y										
11657	A_23_P47484	GLYATL2			Y				Y	Y																Y																			
3479	A_23_P29621	GLYCTK			Y				Y	Y									Y	Y	Y	Y	Y																							
805	A_23_P144872	GM2A	Y		Y	Y		Y	Y	Y									Y	Y	Y		Y	Y	Y			Y	Y	Y	Y						Y										
4717	A_23_P335463	GMCL1																Y																											
16907	A_23_P28375	GMCL1																	Y			Y																								
3116	A_23_P83469	GMDS			Y	Y	Y	Y	Y	Y	Y	Y															Y																			
20588	A_23_P72068	GMDS			Y	Y	Y	Y	Y	Y	Y	Y							Y								Y	Y	Y	Y						Y										
14234	A_23_P205611	GMFB																												Y						Y										
16974	A_23_P208866	GMFG	Y		Y																										Y						Y										
21631	A_23_P56228	GMIP	Y		Y	Y	Y	Y	Y	Y	Y	Y	Y	Y					Y	Y		Y	Y				Y	Y	Y	Y						Y										
13297	A_23_P59976	GML			Y	Y		Y	Y	Y																Y	Y	Y	Y						Y										
6320	A_23_P67907	GMPPA			Y	Y		Y	Y	Y									Y	Y	Y	Y	Y	Y	Y	Y	Y	Y			Y	Y	Y	Y	Y	Y	Y										
9541	A_23_P92202	GMPPB			Y	Y	Y	Y		Y	Y	Y							Y	Y		Y					Y																			
6323	A_23_P142289	GNA11			Y				Y	Y									Y	Y	Y		Y	Y	Y																						
10918	A_23_P5059	GNA11			Y	Y	Y	Y	Y	Y	Y	Y							Y	Y	Y	Y	Y	Y	Y	Y	Y	Y				Y														
19995	A_23_P215269	GNA12			Y	Y	Y	Y	Y	Y	Y	Y									Y			Y				Y	Y	Y	Y						Y								Y		
9443	A_23_P379670	GNA13			Y																						Y																			
2594	A_23_P169479	GNA14							Y	Y																																				
20551	A_23_P415561	GNA14							Y	Y										Y											Y						Y										
19429	A_23_P16455	GNA15				Y			Y	Y										Y		Y																								
8005	A_23_P122976	GNAI1			Y																						Y			Y						Y										
1039	A_23_P144106	GNAI2			Y	Y	Y	Y	Y	Y	Y	Y	Y	Y				Y	Y	Y	Y	Y	Y	Y	Y	Y	Y			Y	Y	Y				Y		Y				Y			Y	
8508	A_23_P149581	GNAI3				Y											Y	Y																											
5316	A_23_P38689	GNAL				Y			Y	Y										Y							Y	Y	Y	Y						Y										
19600	A_23_P218694	GNAS	Y		Y	Y	Y	Y	Y	Y	Y	Y						Y	Y	Y	Y	Y		Y			Y															Y				
1803	A_23_P416581	GNAZ			Y				Y	Y																																				
17048	A_23_P22926	GNB1																			Y																								
15865	A_23_P218753	GNB1L			Y	Y	Y	Y	Y	Y	Y	Y	Y	Y							Y		Y				Y																			
21198	A_23_P82296	GNB2			Y	Y	Y	Y	Y	Y	Y	Y	Y	Y				Y	Y	Y	Y	Y	Y	Y	Y	Y	Y	Y	Y	Y	Y	Y	Y	Y	Y	Y		Y				Y			Y	Y
21274	A_23_P76350	GNB3			Y	Y		Y	Y	Y	Y	Y	Y	Y				Y	Y	Y	Y	Y	Y	Y	Y	Y	Y	Y	Y	Y	Y	Y	Y	Y	Y	Y										
12531	A_23_P18186	GNB4	Y	Y													Y		Y		Y																								
20635	A_23_P205778	GNB5																				Y																							
17886	A_23_P216489	GNE			Y																		Y				Y			Y						Y										
1178	A_23_P111701	GNG11																	Y			Y																								
13862	A_23_P412	GNG12			Y														Y								Y																	
20986	A_23_P311732	GNG12																Y	Y			Y																								
3430	A_23_P54736	GNG13			Y												Y		Y		Y																								

10780	A_23_P32536	GNG2			Y	Y		Y	Y	Y									Y	Y		Y	Y				Y																			
11307	A_23_P139192	GNG3			Y	Y		Y		Y																	Y			Y						Y										
9829	A_23_P153897	GNG7			Y	Y	Y	Y	Y	Y	Y	Y															Y			Y						Y										
22485	A_23_P426363	GNG8				Y			Y	Y																				Y						Y										
751	A_23_P26994	GNGT2	Y		Y				Y	Y										Y							Y	Y	Y	Y						Y										
16535	A_23_P145288	GNL1			Y	Y		Y		Y								Y	Y	Y	Y	Y	Y	Y	Y	Y	Y				Y															
80	A_23_P34578	GNL2																									Y																			
1004	A_23_P22499	GNL3L																Y																												
7084	A_23_P69918	GNL3L			Y	Y	Y	Y	Y	Y	Y	Y	Y	Y				Y	Y	Y		Y	Y	Y			Y	Y	Y	Y						Y								Y		
16812	A_23_P209954	GNLY	Y	Y	Y	Y	Y	Y	Y	Y	Y	Y															Y	Y	Y	Y						Y										
3256	A_23_P7952	GNMT							Y	Y																																				
17841	A_23_P144623	GNPDA1			Y															Y	Y		Y				Y																			
299	A_23_P144581	GNPDA2			Y	Y		Y		Y										Y							Y																			
5801	A_23_P14886	GNPTG																		Y	Y	Y																								
5392	A_23_P254594	GNRH1	Y		Y													Y																												
21196	A_23_P40272	GNRH2			Y	Y	Y	Y	Y	Y	Y	Y											Y				Y	Y	Y	Y						Y										
456	A_23_P379019	GNRHR2							Y	Y			Y					Y																												
19124	A_23_P139628	GNS			Y	Y	Y	Y	Y	Y	Y	Y						Y									Y	Y	Y	Y						Y										
9401	A_23_P258976	GOLGA1			Y				Y	Y																																				
2652	A_23_P203819	GOLGA3			Y	Y	Y	Y	Y	Y	Y	Y						Y		Y							Y	Y	Y	Y																
6897	A_23_P256391	GOLGA4																		Y																										
13266	A_23_P258572	GOLGA4																		Y																										
21243	A_23_P32869	GOLGA4																		Y																										
20225	A_23_P3193	GOLGA5																		Y			Y																							
4858	A_23_P71440	GOLGA7																										Y																		
9811	A_23_P26221	GOLGA8A			Y																																									
1861	A_23_P33173	GOLGB1			Y	Y		Y	Y	Y	Y	Y	Y	Y				Y	Y	Y		Y	Y	Y			Y	Y	Y	Y						Y										
2727	A_23_P256282	GOLGB1				Y																						Y																		
941	A_23_P158771	GOLIM4																										Y																		
16170	A_23_P146506	GOLM1			Y	Y	Y	Y	Y	Y	Y	Y	Y	Y									Y				Y	Y	Y	Y						Y										
3308	A_23_P156015	GOLPH3																					Y																							
17637	A_23_P200890	GOLPH3L			Y		Y				Y												Y																							
20882	A_23_P134734	GOLSYN			Y				Y	Y	Y	Y	Y	Y				Y	Y	Y			Y	Y			Y																			
17313	A_23_P137675	GOLT1A			Y																																									
7753	A_23_P162425	GOLT1B			Y	Y		Y		Y								Y		Y		Y					Y			Y						Y										
3488	A_23_P97250	GON4L	Y	Y	Y	Y	Y	Y	Y	Y	Y	Y	Y	Y				Y	Y	Y	Y	Y	Y	Y	Y	Y	Y	Y	Y	Y	Y	Y				Y				Y						
14386	A_23_P85862	GON4L			Y																						Y																			
20160	A_23_P332169	GON4L			Y															Y							Y																			
2274	A_23_P336040	GOPC							Y	Y																																				
10838	A_23_P58877	GOPC			Y	Y	Y	Y	Y	Y	Y	Y							Y	Y	Y	Y	Y					Y		Y						Y										
11044	A_23_P165494	GORASP2			Y	Y		Y	Y	Y																		Y																		
224	A_23_P252641	GOSR1																Y		Y																										
1892	A_23_P311608	GOSR2			Y	Y		Y	Y	Y	Y	Y	Y	Y						Y										Y						Y										
8169	A_23_P207719	GOSR2			Y	Y	Y	Y		Y	Y	Y						Y	Y	Y	Y			Y			Y	Y	Y	Y						Y										
683	A_23_P63825	GOT1			Y				Y	Y								Y		Y		Y					Y																			
21407	A_23_P152928	GP1BA																									Y																			
21662	A_23_P21421	GP2			Y	Y		Y		Y	Y	Y	Y	Y						Y								Y		Y						Y										
12893	A_23_P27784	GP6			Y																							Y		Y						Y										
21505	A_23_P33256	GP9			Y										Y								Y				Y																			
20277	A_23_P147383	GPAA1			Y	Y	Y	Y	Y	Y	Y	Y	Y	Y				Y	Y	Y	Y	Y	Y	Y	Y	Y	Y				Y															
14409	A_23_P27814	GPATCH1			Y	Y	Y	Y	Y	Y	Y	Y								Y	Y	Y					Y	Y	Y	Y																
21464	A_23_P34757	GPATCH2																		Y	Y		Y																							
8342	A_23_P149419	GPATCH3				Y														Y		Y	Y																							
19472	A_23_P400378	GPBAR1	Y	Y	Y	Y	Y	Y	Y	Y	Y	Y															Y			Y						Y										
3111	A_23_P30395	GPBP1			Y	Y	Y	Y	Y	Y	Y	Y	Y	Y				Y	Y	Y		Y	Y	Y			Y	Y	Y	Y						Y										
13456	A_23_P364465	GPBP1			Y	Y	Y	Y	Y	Y	Y	Y	Y	Y				Y	Y	Y		Y	Y	Y			Y	Y	Y	Y						Y										
2159	A_23_P209904	GPC1																		Y		Y																								
8171	A_23_P255462	GPC3	Y	Y	Y	Y		Y	Y	Y																	Y	Y	Y	Y						Y										
3432	A_23_P435407	GPC4			Y	Y	Y	Y		Y	Y	Y	Y	Y					Y	Y	Y		Y				Y	Y	Y	Y						Y										
3013	A_23_P112874	GPC5																Y																												
1146	A_23_P371682	GPC6																					Y																							
9725	A_23_P10629	GPC6																										Y																		
13081	A_23_P204736	GPD1			Y																						Y																			
21403	A_23_P318284	GPD1L			Y	Y		Y	Y	Y	Y	Y	Y	Y						Y		Y						Y		Y						Y										
1603	A_23_P119812	GPD2																			Y																									
18092	A_23_P47181	GPHA2				Y														Y		Y																								
10530	A_23_P336678	GPHB5																			Y							Y																		
15149	A_23_P250499	GPI			Y	Y	Y	Y		Y	Y	Y						Y	Y	Y		Y		Y			Y																			
576	A_23_P72697	GPIHBP1																	Y				Y																							
9278	A_23_P114155	GPKOW																		Y																										
21447	A_23_P145550	GPLD1				Y																					Y	Y	Y	Y						Y										
19093	A_23_P378416	GPM6B							Y	Y								Y		Y			Y																							
19826	A_23_P85067	GPM6B							Y	Y																																				
20621	A_23_P17144	GPN1																												Y						Y										
7243	A_23_P138025	GPN2																			Y																									
1110	A_23_P116999	GPN3			Y																																									
21170	A_23_P134426	GPNMB	Y												Y																															

7581	A_23_P217036	GPR107																		Y		Y																								
19405	A_23_P388762	GPR107																		Y		Y	Y																							
9663	A_23_P90011	GPR108			Y	Y	Y	Y	Y	Y	Y	Y	Y	Y					Y	Y		Y					Y			Y						Y										
1642	A_23_P64721	GPR109B			Y				Y	Y																	Y																			
212	A_23_P214267	GPR110			Y	Y		Y	Y	Y																	Y			Y						Y										
11297	A_23_P312901	GPR112				Y																																								
21071	A_23_P206290	GPR114			Y	Y		Y	Y	Y																	Y	Y	Y	Y						Y										
19044	A_23_P252874	GPR116			Y													Y																												
16739	A_23_P21425	GPR119	Y	Y	Y	Y	Y	Y	Y	Y	Y	Y							Y	Y			Y				Y			Y						Y										
20231	A_23_P326270	GPR123			Y	Y		Y	Y	Y																	Y	Y	Y	Y						Y										
4222	A_23_P43283	GPR124			Y													Y	Y	Y	Y	Y	Y	Y	Y	Y					Y															
12593	A_23_P158925	GPR125			Y				Y	Y										Y	Y						Y																			
3537	A_23_P214756	GPR126																			Y		Y																							
21863	A_23_P139516	GPR133							Y	Y													Y																							
19812	A_23_P205575	GPR135	Y	Y																							Y	Y	Y	Y						Y										
18689	A_23_P416686	GPR137			Y	Y		Y		Y	Y	Y	Y	Y				Y	Y	Y		Y	Y	Y			Y	Y	Y	Y																
11595	A_23_P140316	GPR137C			Y	Y		Y		Y																																				
14664	A_23_P26602	GPR139																Y	Y	Y		Y		Y																						
4789	A_23_P111550	GPR141			Y																						Y																			
8170	A_23_P20035	GPR146																		Y		Y	Y																							
17781	A_23_P6943	GPR15			Y																																									
15029	A_23_P144760	GPR151			Y																		Y				Y																			
9538	A_23_P97652	GPR153			Y	Y		Y	Y	Y									Y	Y		Y	Y				Y																			
17006	A_23_P92275	GPR156			Y	Y		Y	Y	Y										Y		Y	Y				Y																			
7985	A_23_P158	GPR157			Y	Y		Y	Y	Y										Y		Y	Y																							
16601	A_23_P167005	GPR160			Y				Y	Y	Y	Y	Y	Y						Y		Y					Y			Y						Y										
5989	A_23_P354320	GPR161			Y				Y	Y	Y	Y	Y	Y				Y	Y								Y			Y						Y										
22213	A_23_P103532	GPR161																									Y																			
3825	A_23_P99141	GPR162	Y	Y	Y		Y		Y	Y	Y	Y															Y																			
11553	A_23_P253317	GPR171	Y	Y	Y										Y					Y							Y																			
8409	A_23_P258281	GPR172A			Y	Y	Y	Y	Y	Y	Y	Y	Y	Y				Y	Y	Y	Y	Y	Y	Y	Y	Y	Y	Y	Y	Y	Y	Y				Y				Y						
11619	A_23_P15700	GPR172B			Y	Y	Y	Y	Y	Y	Y	Y	Y	Y				Y	Y	Y		Y		Y			Y	Y	Y	Y																
21432	A_23_P346291	GPR173			Y	Y		Y		Y									Y	Y		Y	Y				Y			Y						Y										
2321	A_23_P57643	GPR175			Y	Y	Y	Y	Y	Y	Y	Y	Y	Y				Y		Y	Y	Y	Y				Y	Y	Y	Y						Y										
9346	A_23_P200653	GPR177																Y												Y						Y										
10073	A_23_P14165	GPR18	Y		Y																																									
21656	A_23_P88021	GPR180			Y	Y		Y	Y	Y	Y	Y	Y	Y				Y									Y			Y						Y										
6701	A_23_P25566	GPR183	Y	Y	Y																						Y																			
5524	A_23_P159237	GPR20																	Y	Y		Y	Y																							
847	A_23_P149922	GPR26	Y		Y	Y		Y		Y										Y			Y				Y	Y	Y	Y						Y										
12656	A_23_P385835	GPR27																	Y	Y																										
21986	A_23_P142096	GPR32																Y																												
15261	A_23_P11201	GPR34	Y		Y																																									
4710	A_23_P154245	GPR35			Y	Y		Y		Y								Y	Y	Y		Y	Y	Y			Y	Y	Y	Y																
16456	A_23_P357284	GPR4	Y	Y	Y		Y				Y							Y	Y	Y			Y	Y			Y	Y	Y	Y																
1343	A_23_P47507	GPR44			Y																																									
5851	A_23_P85530	GPR52			Y				Y	Y																	Y																			
22234	A_23_P28353	GPR55																										Y																		
13475	A_23_P206280	GPR56			Y	Y		Y		Y																		Y																		
18850	A_23_P156919	GPR6																									Y																			
5961	A_23_P160964	GPR61			Y				Y	Y										Y			Y				Y																			
20040	A_23_P434289	GPR62			Y	Y		Y	Y	Y	Y	Y	Y	Y													Y			Y						Y										
3176	A_23_P253695	GPR64				Y			Y	Y																		Y																		
16369	A_23_P14564	GPR65	Y	Y	Y										Y																															
21166	A_23_P54243	GPR68																					Y																							
11803	A_23_P68234	GPR75			Y															Y																										
13496	A_23_P69652	GPR78				Y																																								
19996	A_23_P394246	GPR81	Y	Y	Y	Y		Y	Y	Y	Y	Y	Y	Y						Y							Y	Y	Y	Y						Y										
20424	A_23_P76126	GPR81			Y	Y	Y	Y	Y	Y	Y	Y	Y	Y				Y	Y	Y				Y			Y	Y	Y	Y						Y										
16501	A_23_P354889	GPR83			Y	Y		Y	Y	Y																				Y						Y										
18699	A_23_P25155	GPR84	Y	Y	Y	Y		Y	Y	Y	Y	Y	Y	Y														Y		Y						Y										
19613	A_23_P215687	GPR85																		Y																										
11115	A_23_P149745	GPR88			Y	Y		Y		Y																																				
20973	A_23_P140760	GPR97			Y	Y		Y		Y									Y								Y	Y	Y	Y																
4689	A_23_P19134	GPR98			Y				Y	Y	Y	Y	Y	Y																Y						Y										
18494	A_23_P96590	GPRASP1			Y	Y		Y	Y	Y										Y								Y		Y						Y										
7062	A_23_P408167	GPRASP2	Y	Y					Y	Y								Y		Y										Y						Y										
6062	A_23_P324327	GPRC5B			Y	Y	Y	Y	Y	Y	Y	Y						Y	Y	Y		Y	Y	Y			Y	Y	Y	Y						Y						Y				
1245	A_23_P38167	GPRC5C			Y				Y	Y									Y								Y																			
7298	A_23_P81683	GPRC6A																									Y																			
9500	A_23_P98115	GPRIN2			Y	Y		Y	Y	Y								Y	Y	Y		Y	Y	Y			Y	Y	Y	Y						Y										
21598	A_23_P343382	GPRIN2																										Y																		
22374	A_23_P158662	GPS1			Y	Y	Y	Y	Y	Y	Y	Y						Y	Y	Y	Y	Y	Y	Y	Y	Y	Y	Y	Y	Y	Y	Y	Y			Y										
6023	A_23_P218408	GPS2																		Y	Y	Y																								
5734	A_23_P112756	GPSM1							Y	Y																																				
10118	A_23_P135542	GPSM1							Y	Y																																				
11581	A_23_P219060	GPSM3			Y		Y				Y																Y																			

2023	A_23_P56339	GPSN2			Y													Y	Y	Y		Y		Y																						
15612	A_23_P37892	GPT2			Y				Y	Y																	Y			Y						Y										
1994	A_23_P250671	GPX1																	Y	Y		Y	Y																							
10392	A_23_P133475	GPX3			Y													Y	Y	Y	Y	Y	Y	Y	Y	Y					Y		Y	Y	Y											
3997	A_23_P28073	GPX4							Y	Y																				Y						Y										
11456	A_23_P73972	GPX7				Y			Y	Y			Y														Y			Y						Y										
8099	A_23_P56208	GRAMD1A			Y	Y		Y		Y								Y	Y	Y		Y	Y	Y			Y			Y						Y										
16323	A_23_P47386	GRAMD1B																		Y			Y																							
19400	A_23_P253012	GRAMD1C			Y	Y		Y	Y	Y	Y	Y	Y	Y						Y	Y		Y							Y						Y										
6001	A_23_P91795	GRAMD4				Y																						Y																		
5354	A_23_P49638	GRAP			Y	Y		Y	Y	Y	Y	Y	Y	Y				Y	Y	Y		Y	Y	Y			Y	Y	Y	Y																
5744	A_23_P392962	GRAP				Y														Y		Y	Y																							
19533	A_23_P143716	GRAP2			Y													Y																												
10576	A_23_P105442	GRASP																		Y																										
8369	A_23_P122863	GRB10																Y																												
14794	A_23_P154526	GRB14																					Y																							
1952	A_23_P89246	GRB2																Y	Y	Y		Y	Y	Y																						
16731	A_23_P418537	GRB2	Y	Y	Y	Y	Y	Y	Y	Y	Y	Y						Y	Y	Y	Y	Y	Y	Y	Y	Y	Y			Y	Y	Y	Y	Y	Y	Y				Y						
10859	A_23_P163989	GRB7			Y	Y		Y	Y	Y																																				
5935	A_23_P329768	GREB1	Y	Y	Y	Y		Y	Y	Y	Y	Y	Y	Y													Y	Y	Y	Y						Y										
20892	A_23_P108862	GREB1	Y								Y		Y	Y													Y																			
5892	A_23_P394337	GREM1			Y	Y		Y		Y										Y																										
16222	A_23_P432945	GREM1	Y		Y										Y																															
19166	A_23_P218189	GREM1				Y																																								
22090	A_23_P97181	GREM2																Y										Y																		
13203	A_23_P5882	GRHL1																		Y	Y		Y																							
529	A_23_P60225	GRHPR																		Y		Y					Y			Y						Y										
4486	A_23_P303072	GRIA1	Y	Y	Y	Y		Y		Y												Y																								
22132	A_23_P144939	GRIA1				Y																																								
7817	A_23_P171062	GRIA3				Y													Y			Y						Y																		
6785	A_23_P32805	GRID1																					Y																							
6923	A_23_P16063	GRIK5																												Y						Y										
7453	A_23_P113872	GRIN1			Y	Y		Y		Y										Y		Y	Y				Y																			
17234	A_23_P96071	GRIN1			Y	Y		Y	Y	Y																	Y	Y	Y	Y						Y										
18157	A_23_P84670	GRIN1				Y														Y		Y	Y																							
4211	A_23_P49546	GRIN2C																		Y																										
14551	A_23_P153549	GRIN2D			Y	Y		Y	Y	Y									Y	Y		Y	Y				Y																			
3242	A_23_P347541	GRIN3A			Y																						Y																			
17740	A_23_P316960	GRINA			Y	Y	Y	Y	Y	Y	Y	Y						Y	Y	Y	Y	Y	Y	Y	Y	Y	Y			Y	Y	Y				Y										
9230	A_23_P313543	GRK1			Y															Y	Y																									
11773	A_23_P44019	GRK1			Y															Y							Y																			
22535	A_23_P60891	GRK1				Y																																								
5697	A_23_P212756	GRK4			Y	Y	Y	Y	Y	Y	Y	Y	Y	Y				Y	Y	Y	Y	Y	Y	Y	Y	Y	Y	Y	Y	Y	Y	Y	Y	Y	Y	Y										
5695	A_23_P12884	GRK5			Y																																									
19983	A_23_P257256	GRK6	Y	Y	Y	Y	Y	Y	Y	Y	Y	Y	Y	Y				Y	Y	Y	Y	Y	Y	Y	Y	Y	Y	Y	Y	Y	Y	Y	Y	Y	Y	Y				Y						
9551	A_23_P259377	GRK7																					Y																							
11851	A_23_P39137	GRLF1											Y																	Y						Y										
9625	A_23_P252184	GRM2				Y																																								
124	A_23_P70588	GRM4			Y	Y	Y	Y		Y	Y	Y						Y									Y	Y	Y	Y						Y										
21211	A_23_P49708	GRN			Y	Y	Y	Y		Y	Y	Y	Y					Y	Y	Y	Y	Y		Y																						
806	A_23_P101131	GRP			Y	Y		Y	Y	Y								Y	Y	Y	Y	Y		Y			Y	Y	Y	Y																
1102	A_23_P92476	GRPEL1																												Y						Y										
8572	A_23_P404091	GRPEL2				Y																																								
8596	A_23_P62255	GRPR			Y																																									
1229	A_23_P46131	GRRP1			Y														Y	Y			Y				Y																			
2441	A_23_P212937	GRSF1			Y	Y		Y		Y										Y			Y				Y																			
17482	A_23_P76774	GSC			Y				Y	Y																	Y																			
7073	A_23_P109382	GSC2			Y														Y	Y							Y																			
2970	A_23_P341513	GSDMA			Y	Y		Y	Y	Y										Y		Y	Y				Y	Y	Y	Y						Y										
20525	A_23_P152605	GSDMA				Y					Y		Y	Y														Y																		
9055	A_23_P66454	GSDMB																					Y																							
21227	A_23_P60120	GSDMC				Y												Y		Y																										
11133	A_23_P112102	GSDMD			Y	Y	Y	Y		Y	Y	Y	Y					Y	Y	Y	Y	Y	Y	Y	Y	Y	Y				Y															
2648	A_23_P139872	GSG1			Y	Y		Y		Y									Y	Y							Y	Y	Y	Y																
5712	A_23_P164677	GSK3A			Y													Y	Y	Y		Y	Y	Y			Y	Y	Y	Y						Y										
18197	A_23_P255884	GSN			Y	Y	Y	Y		Y	Y	Y	Y	Y					Y	Y		Y	Y				Y															Y				
8880	A_23_P206309	GSPT1	Y		Y	Y		Y	Y	Y	Y	Y	Y	Y						Y			Y				Y	Y	Y	Y						Y										
5662	A_23_P19120	GSPT2																Y	Y			Y																								
5108	A_23_P210920	GSS																			Y																									
16637	A_23_P135417	GSTA1							Y	Y								Y												Y						Y										
9250	A_23_P214300	GSTA2																Y																												
18680	A_23_P253495	GSTA3				Y			Y	Y								Y				Y					Y			Y						Y										
21066	A_23_P93141	GSTA5							Y	Y	Y	Y	Y	Y				Y				Y								Y						Y										
12446	A_23_P213038	GSTCD			Y				Y	Y										Y							Y																			
19935	A_23_P31503	GSTK1			Y	Y		Y		Y																		Y																		
11294	A_23_P126794	GSTM1							Y	Y																																				
11069	A_23_P397208	GSTM2			Y				Y	Y										Y			Y							Y						Y										

22085	A_23_P12343	GSTM3			Y	Y		Y	Y	Y	Y	Y	Y	Y													Y	Y	Y	Y														Y		
17546	A_23_P97606	GSTM5																		Y			Y																							
4189	A_23_P202206	GSTO2			Y		Y				Y																Y																			
9608	A_23_P202658	GSTP1							Y	Y	Y	Y	Y	Y									Y				Y			Y						Y										
10770	A_23_P109427	GSTT2			Y				Y	Y																				Y						Y										
12675	A_23_P106204	GSTZ1							Y	Y																																				
165	A_23_P92453	GSX2				Y																						Y																		
21314	A_23_P131166	GTDC1																									Y																			
11618	A_23_P349817	GTF2F2																		Y																										
11857	A_23_P257694	GTF2IRD1																Y		Y		Y																								
5200	A_23_P59616	GTF2IRD2			Y	Y	Y	Y	Y	Y	Y	Y	Y	Y														Y																		
21424	A_23_P56437	GTF3C3																		Y		Y	Y							Y						Y										
11086	A_23_P103126	GTPBP1			Y	Y		Y	Y	Y	Y	Y	Y	Y				Y	Y	Y		Y	Y	Y			Y																			
16571	A_23_P91734	GTPBP1				Y			Y	Y																		Y																		
17918	A_23_P416106	GTPBP1			Y	Y	Y	Y		Y	Y	Y															Y																			
12561	A_23_P12874	GTPBP4							Y	Y								Y																												
9669	A_23_P258513	GTPBP5																		Y								Y																		
15765	A_23_P170873	GTPBP6			Y	Y		Y		Y								Y	Y	Y		Y	Y	Y																						
12296	A_23_P92281	GTPBP8			Y																						Y																			
1375	A_23_P57588	GTSE1																Y				Y																								
2706	A_23_P47949	GTSF1																				Y																								
1271	A_23_P63032	GUCA2B			Y	Y		Y		Y								Y	Y	Y			Y	Y			Y	Y	Y	Y						Y										
8832	A_23_P349997	GUCY1A2							Y	Y																																				
19127	A_23_P69573	GUCY1A3			Y																																									
14254	A_23_P14255	GUCY1B2			Y		Y				Y																Y																			
1191	A_23_P76312	GUCY2C			Y				Y	Y																				Y						Y										
22309	A_23_P130261	GUCY2D																										Y																		
17903	A_23_P360302	GUCY2F																									Y			Y						Y										
1681	A_23_P155959	GUF1				Y																																								
2039	A_23_P201097	GUK1			Y	Y	Y	Y	Y	Y	Y	Y							Y	Y			Y				Y	Y	Y	Y						Y										
17515	A_23_P334608	GUSB			Y													Y																												
4496	A_23_P29414	GYG1			Y	Y		Y		Y																	Y			Y						Y										
19452	A_23_P104617	GYLTL1B				Y			Y	Y																	Y																			
18829	A_23_P90957	GYPC			Y	Y	Y	Y		Y	Y	Y						Y	Y	Y	Y	Y	Y	Y	Y	Y	Y	Y	Y	Y	Y	Y	Y	Y	Y											
11554	A_23_P41307	GYPE																									Y																			
8062	A_23_P210797	GZF1							Y	Y																				Y						Y										
18169	A_23_P433188	GZF1			Y	Y	Y	Y	Y	Y	Y	Y						Y	Y	Y		Y	Y	Y			Y	Y	Y	Y			Y			Y										
14286	A_23_P133445	GZMA	Y	Y	Y				Y	Y																	Y																			
9489	A_23_P117602	GZMB	Y	Y	Y	Y	Y	Y	Y	Y	Y	Y							Y	Y							Y	Y	Y	Y						Y										
1541	A_23_P128993	GZMH	Y	Y	Y																						Y																			
4119	A_23_P410085	GZMH	Y	Y	Y		Y		Y	Y	Y	Y															Y	Y	Y	Y																
22439	A_23_P156216	GZMK	Y																																											
11457	A_23_P130835	GZMM																Y																												
18523	A_23_P139216	H19			Y													Y	Y	Y	Y	Y	Y	Y	Y	Y	Y				Y		Y	Y	Y											
12888	A_23_P372994	H1FOO			Y	Y		Y		Y																	Y	Y	Y	Y						Y										
1219	A_23_P96080	H1FX			Y													Y	Y	Y	Y	Y	Y	Y	Y	Y	Y				Y		Y													
15522	A_23_P171267	H2AFB1				Y														Y			Y																							
9946	A_23_P145904	H2AFV																Y																												
8436	A_23_P319432	H2AFX			Y	Y		Y		Y								Y	Y	Y				Y																						
13458	A_23_P47370	H2AFX			Y																						Y																			
14495	A_23_P70045	H2AFY																		Y	Y		Y																							
8284	A_23_P133147	H2AFZ	Y	Y	Y																																									
14587	A_23_P23141	H3F3A			Y	Y	Y	Y	Y	Y	Y	Y	Y	Y							Y						Y			Y						Y										
7372	A_23_P152516	H3F3B																Y		Y		Y																								
402	A_23_P28697	HAAO			Y	Y		Y		Y																	Y																			
7992	A_23_P310995	HABP4																			Y																									
6841	A_23_P250002	HACE1			Y	Y		Y	Y	Y	Y	Y	Y	Y						Y		Y	Y				Y	Y	Y	Y						Y										
11167	A_23_P121182	HACL1																Y					Y																							
21835	A_23_P167227	HADH																		Y			Y																							
18389	A_23_P159510	HADHA																		Y			Y																							
19499	A_23_P79703	HADHB			Y	Y	Y	Y	Y	Y	Y	Y	Y					Y		Y							Y																			
7831	A_23_P129685	HAGH			Y																						Y																			
1311	A_23_P66128	HAGHL																		Y		Y																								
8631	A_23_P61643	HAL	Y		Y																									Y						Y										
16998	A_23_P373521	HAND2																	Y	Y		Y								Y						Y										
22482	A_23_P154708	HAO1																										Y																		
21907	A_23_P46105	HAO2			Y																						Y																			
10955	A_23_P7727	HAPLN1							Y	Y																																				
3469	A_23_P149232	HAPLN2			Y	Y		Y	Y	Y												Y	Y				Y																			
22425	A_23_P14754	HAPLN3	Y	Y	Y	Y		Y	Y	Y	Y	Y	Y	Y	Y			Y	Y	Y	Y	Y	Y	Y	Y	Y	Y			Y	Y	Y				Y										
12884	A_23_P403507	HARBI1				Y			Y	Y																		Y																		
17228	A_23_P133232	HARS																		Y	Y																									
2398	A_23_P41588	HARS2			Y				Y	Y																	Y																			
C11699.1	A_23_P10205	HAS2																					Y																							
11221	A_23_P14971	HAS3							Y	Y																																				
2499	A_23_P339480	HAT1																					Y																							
16329	A_23_P347610	HAVCR1																									Y																			

11237	A_23_P18902	HAVCR2			Y																																								
4718	A_23_P115224	HAX1				Y																																							
17384	A_23_P37856	HBA1																				Y																							
12736	A_23_P26457	HBA2																				Y																							
9862	A_23_P203558	HBB			Y														Y	Y																									
16668	A_23_P87346	HBD			Y										Y					Y							Y																		
3223	A_23_P47674	HBE1			Y	Y		Y		Y								Y									Y																		
7806	A_23_P53137	HBG1			Y	Y		Y		Y																	Y																		
14381	A_23_P64538	HBG1			Y	Y		Y	Y	Y																	Y	Y	Y	Y															
4368	A_23_P15055	HBM																												Y						Y									
20024	A_23_P49254	HBQ1																		Y			Y																						
13072	A_23_P254379	HBS1L				Y			Y	Y																																			
12177	A_23_P115137	HBXIP																	Y	Y			Y																						
442	A_23_P3651	HBZ			Y																						Y																		
11450	A_23_P64499	HCCA2			Y	Y		Y		Y										Y		Y	Y				Y																		
1444	A_23_P257945	HCCS	Y	Y	Y	Y		Y	Y	Y																	Y	Y	Y	Y						Y									
20368	A_23_P26534	HCFC1R1																		Y																									
1325	A_23_P25403	HCFC2																	Y	Y																									
9168	A_23_P149798	hCG_1776259			Y	Y	Y	Y	Y	Y	Y	Y	Y	Y				Y		Y		Y					Y	Y	Y	Y						Y									
15262	A_23_P86088	hCG_20426				Y																						Y																	
21189	A_23_P254768	HCG4				Y																																							
19177	A_23_P251027	HCG9																									Y																		
6617	A_23_P102801	HCK	Y	Y	Y	Y		Y	Y	Y										Y																									
4126	A_23_P62026	HCLS1			Y	Y		Y	Y	Y					Y				Y	Y	Y	Y	Y				Y				Y		Y	Y	Y										
11562	A_23_P73429	HCLS1	Y						Y	Y					Y				Y																										
4262	A_23_P342612	HCN2			Y															Y		Y	Y																						
14123	A_23_P165263	HCN2			Y	Y		Y	Y	Y										Y							Y			Y						Y									
7790	A_23_P34827	HCN3			Y																																								
14826	A_23_P26201	HCN4									Y		Y	Y																															
8909	A_23_P111125	HCP5																	Y																										
21720	A_23_P129989	HCRT			Y	Y	Y	Y	Y	Y	Y	Y								Y	Y	Y	Y				Y			Y						Y									
18622	A_23_P74178	HCRTR1	Y	Y	Y	Y		Y	Y	Y	Y	Y	Y	Y						Y							Y	Y	Y	Y						Y									
5020	A_23_P39386	HCST			Y				Y	Y									Y	Y			Y				Y																		
22402	A_23_P155358	HDAC11			Y																						Y																		
10676	A_23_P122304	HDAC2							Y	Y																																			
6189	A_23_P7388	HDAC3																		Y		Y																							
9915	A_23_P210048	HDAC4			Y	Y		Y	Y	Y	Y	Y	Y	Y					Y								Y	Y	Y	Y						Y									
335	A_23_P26916	HDAC5				Y												Y		Y	Y	Y																							
9906	A_23_P250455	HDAC6																		Y			Y																						
14309	A_23_P2582	HDAC7																		Y			Y																						
14372	A_23_P84922	HDAC8																		Y			Y																						
12970	A_23_P65830	HDDC3																	Y																										
7177	A_23_P149239	HDGF			Y														Y			Y																							
22011	A_23_P101699	HDGF2			Y	Y		Y		Y								Y		Y	Y	Y					Y																		
10448	A_23_P301481	HDGFL1			Y				Y	Y																	Y																		
21473	A_23_P344451	HDGFRP3			Y	Y	Y	Y	Y	Y	Y	Y	Y	Y													Y			Y						Y									
14754	A_23_P153098	HDHD2	Y	Y	Y	Y	Y	Y	Y	Y	Y	Y	Y	Y				Y	Y	Y		Y	Y	Y			Y	Y	Y	Y						Y									
2395	A_23_P112541	HDHD3																												Y						Y									
19841	A_23_P131417	HDLBP			Y													Y		Y	Y	Y	Y				Y																		
14074	A_23_P386366	HDX																	Y			Y					Y																		
21510	A_23_P103628	HEATR1																			Y																								
19465	A_23_P77959	HEATR6			Y				Y	Y																	Y			Y						Y									
22226	A_23_P117082	HEBP1			Y	Y		Y	Y	Y								Y	Y	Y			Y	Y			Y																		
15118	A_23_P19691	HEBP2			Y																						Y																		
19457	A_23_P59349	HECA																		Y																									
5938	A_23_P54006	HECTD1			Y	Y	Y	Y	Y	Y	Y	Y	Y	Y						Y		Y	Y				Y	Y	Y	Y						Y									
21495	A_23_P355525	HECTD2			Y	Y		Y	Y	Y								Y	Y	Y		Y		Y						Y						Y									
16132	A_23_P149216	HECTD3			Y	Y		Y		Y																																			
21754	A_23_P50974	HECW2																												Y						Y									
7603	A_23_P253650	HEG1																		Y		Y	Y																						
4245	A_23_P92614	HEL308																Y		Y																									
22220	A_23_P43407	HEMGN	Y	Y	Y	Y	Y	Y		Y	Y	Y	Y	Y				Y	Y	Y				Y			Y	Y	Y	Y						Y									
927	A_23_P109934	HEMK1	Y	Y	Y	Y	Y	Y	Y	Y	Y	Y	Y	Y				Y	Y	Y		Y	Y	Y			Y	Y	Y	Y	Y	Y	Y	Y	Y	Y									
321	A_23_P22526	HEPH			Y				Y	Y	Y	Y	Y	Y																															
186	A_23_P257503	HERC1			Y	Y		Y		Y																	Y																		
6271	A_23_P48973	HERC2																												Y						Y									
11862	A_23_P33407	HERC2																		Y	Y																								
15433	A_23_P121585	HERC3																Y																											
17350	A_23_P202408	HERC4																Y	Y	Y		Y		Y																					
14784	A_23_P54846	HERPUD1			Y	Y	Y	Y	Y	Y	Y	Y															Y			Y						Y									
22488	A_23_P168490	HERPUD2							Y	Y																																			
12586	A_23_P17998	HES1							Y	Y	Y	Y	Y	Y																															
9086	A_23_P149448	HES4			Y	Y	Y	Y	Y	Y	Y	Y						Y	Y	Y	Y	Y	Y	Y	Y	Y	Y				Y														
7857	A_23_P131202	HES6			Y													Y	Y	Y	Y	Y	Y	Y	Y	Y					Y														
15692	A_23_P107394	HES7			Y	Y		Y	Y	Y								Y	Y	Y		Y	Y	Y			Y																		
3829	A_23_P121106	HESX1																		Y																									
13463	A_23_P300174	HEXDC			Y													Y	Y	Y			Y	Y																					

10740	A_23_P118552	HEXIM1	Y	Y	Y				Y	Y	Y	Y	Y	Y													Y			Y						Y										
20222	A_23_P377218	HEXIM2																Y		Y		Y																								
548	A_23_P157659	HEY1							Y	Y																				Y						Y										
19758	A_23_P168354	HEY2																		Y																										
69	A_23_P46245	HEYL				Y														Y		Y	Y																							
21648	A_23_P145204	HFE			Y																		Y																							
21630	A_23_P86315	HFE2																									Y																			
13061	A_23_P250164	HGD							Y	Y	Y	Y	Y	Y							Y									Y						Y										
3644	A_23_P93780	HGF				Y			Y	Y																				Y						Y										
18782	A_23_P58107	HGFAC			Y	Y		Y	Y	Y													Y				Y			Y						Y										
5144	A_23_P72627	HGS																		Y			Y																							
19225	A_23_P61230	HGS			Y	Y	Y	Y	Y	Y	Y	Y						Y	Y	Y	Y	Y	Y	Y	Y	Y	Y			Y	Y	Y				Y				Y						
15879	A_23_P147950	HGSNAT			Y	Y		Y		Y												Y					Y																			
12709	A_23_P136355	HHAT	Y	Y	Y	Y	Y	Y	Y	Y	Y	Y	Y	Y				Y	Y	Y		Y	Y	Y			Y	Y	Y	Y						Y										
21544	A_23_P167129	HHIP			Y																						Y																			
10097	A_23_P157556	HHLA1																										Y																		
17597	A_23_P166942	HHLA2																Y																												
15168	A_23_P200303	HHLA3																		Y			Y																							
9428	A_23_P45851	HIAT1																		Y																										
6623	A_23_P359144	HIATL1			Y	Y		Y		Y									Y	Y		Y	Y				Y	Y	Y	Y																
1334	A_23_P399169	HIC1			Y																	Y	Y				Y																			
14389	A_23_P129856	HIC1			Y														Y	Y		Y	Y				Y																			
15901	A_23_P141965	HICE1			Y	Y	Y	Y	Y	Y	Y	Y	Y	Y				Y	Y	Y	Y	Y	Y	Y	Y	Y	Y	Y	Y	Y	Y	Y				Y				Y						
20352	A_23_P46964	HIF1AN																Y		Y	Y	Y	Y				Y																			
6196	A_23_P374339	HIF3A			Y	Y		Y		Y								Y	Y	Y		Y		Y			Y	Y	Y	Y																
19990	A_23_P20022	HIG2																		Y							Y																			
20041	A_23_P40885	HIGD1A			Y															Y			Y																							
22417	A_23_P304383	HIGD2A			Y																																									
6961	A_23_P157784	HINT2							Y	Y																																				
19847	A_23_P71033	HIP1																												Y						Y										
2302	A_23_P95002	HIPK1																		Y			Y																							
22251	A_23_P103787	HIPK1																		Y			Y																							
12129	A_23_P169766	HIPK2			Y															Y																										
19590	A_23_P124200	HIPK2			Y																						Y																			
8737	A_23_P422809	HIPK3			Y																																									
20110	A_23_P140938	HIRIP3																	Y	Y																										
C10367	A_23_P213661	HISPPD1			Y	Y		Y	Y	Y										Y			Y				Y	Y	Y	Y						Y										
20546	A_23_P205819	HISPPD2A			Y																																									
4692	A_23_P70454	HIST1H1A				Y			Y	Y	Y	Y	Y	Y																Y						Y										
17539	A_23_P250385	HIST1H1B			Y													Y		Y																										
1015	A_23_P7992	HIST1H1D																					Y																							
2764	A_23_P133842	HIST1H1T																		Y																										
2620	A_23_P372860	HIST1H2AC			Y																						Y																			
6592	A_23_P167983	HIST1H2AC	Y		Y				Y	Y	Y	Y	Y	Y				Y		Y	Y		Y				Y																			
1592	A_23_P428184	HIST1H2AD							Y	Y																																				
12260	A_23_P59045	HIST1H2AE							Y	Y																																				
17250	A_23_P59064	HIST1H2AG																									Y																			
33	A_23_P81867	HIST1H2AH							Y	Y																																				
15993	A_23_P42220	HIST1H2AK				Y			Y	Y																		Y																		
305	A_23_P258477	HIST1H2AL			Y				Y	Y																	Y																			
16692	A_23_P323821	HIST1H2BA			Y																		Y				Y																			
6913	A_23_P111054	HIST1H2BB							Y	Y																																				
2695	A_23_P93180	HIST1H2BC							Y	Y																																				
345	A_23_P19379	HIST1H2BD							Y	Y																																				
4015	A_23_P42178	HIST1H2BF							Y	Y																																				
16566	A_23_P382694	HIST1H2BG							Y	Y																		Y																		
10666	A_23_P93240	HIST1H2BH				Y																						Y																		
11570	A_23_P366216	HIST1H2BH							Y	Y																																				
5845	A_23_P111042	HIST1H2BI							Y	Y																																				
6281	A_23_P133851	HIST1H2BJ							Y	Y																																				
6097	A_23_P8013	HIST1H2BL							Y	Y																																				
11412	A_23_P19408	HIST1H2BM							Y	Y																																				
11330	A_23_P214511	HIST1H2BN																Y		Y			Y				Y																			
18449	A_23_P402081	HIST1H2BN							Y	Y																																				
11584	A_23_P59069	HIST1H2BO							Y	Y																																				
15878	A_23_P111037	HIST1H3A			Y	Y		Y	Y	Y																																				
21338	A_23_P93258	HIST1H3B				Y														Y		Y	Y																							
20392	A_23_P133814	HIST1H3C	Y	Y	Y	Y		Y		Y								Y	Y	Y	Y	Y	Y	Y	Y	Y	Y			Y	Y	Y	Y	Y		Y										
12484	A_23_P219045	HIST1H3D							Y	Y																																				
61	A_23_P70445	HIST1H3E			Y	Y		Y	Y	Y																																				
16312	A_23_P42198	HIST1H3G	Y	Y	Y	Y		Y		Y								Y	Y	Y	Y	Y	Y	Y	Y	Y	Y				Y															
1771	A_23_P93282	HIST1H3J			Y	Y		Y	Y	Y																																				
14146	A_23_P431179	HIST1H4A			Y																																									
12954	A_23_P81850	HIST1H4B			Y	Y	Y	Y		Y	Y	Y															Y	Y	Y	Y																
4871	A_23_P323685	HIST1H4H							Y	Y																																				
17417	A_23_P361347	HIST1H4I																									Y																			
3852	A_23_P70480	HIST1H4L																									Y																			

19118	A_23_P309381	HIST2H2AA4				Y																																								
14722	A_23_P343927	HIST2H2AB																				Y																								
13692	A_23_P115377	HIST2H3D	Y	Y	Y	Y		Y		Y								Y	Y	Y	Y	Y	Y	Y	Y	Y	Y				Y		Y	Y												
8829	A_23_P332992	HIST3H2BB							Y	Y																																				
10124	A_23_P137909	HIST3H3			Y				Y	Y	Y	Y	Y	Y													Y																			
3984	A_23_P388871	HIST4H4																									Y																			
22005	A_23_P19619	HIVEP1				Y														Y		Y																								
18380	A_23_P214766	HIVEP2				Y																						Y																		
19539	A_23_P79429	HJURP	Y	Y	Y													Y		Y							Y	Y	Y	Y																
5568	A_23_P217952	HK1			Y				Y	Y																																				
17246	A_23_P202420	HKDC1			Y													Y	Y	Y				Y			Y																			
8660	A_23_P333528	HLA-A			Y	Y	Y	Y	Y	Y	Y	Y							Y	Y	Y	Y	Y				Y	Y	Y	Y																
16494	A_23_P168037	HLA-A			Y														Y	Y	Y		Y																							
90	A_23_P81938	HLA-B			Y													Y	Y	Y	Y	Y		Y																						
9770	A_23_P135523	HLA-B			Y	Y	Y	Y	Y	Y	Y	Y							Y	Y	Y	Y					Y																			
10007	A_23_P125107	HLA-B			Y	Y	Y	Y	Y	Y	Y	Y							Y	Y	Y	Y					Y																			
19143	A_23_P70542	HLA-B			Y	Y	Y	Y	Y	Y	Y	Y							Y	Y	Y	Y	Y				Y	Y	Y	Y																
9691	A_23_P95917	HLA-C			Y	Y	Y	Y	Y	Y	Y	Y						Y	Y	Y	Y	Y	Y	Y	Y	Y	Y				Y															
9688	A_23_P42304	HLA-DMA	Y	Y	Y	Y	Y	Y	Y	Y	Y	Y	Y	Y					Y	Y			Y				Y	Y	Y	Y						Y										
1613	A_23_P389924	HLA-DMB			Y				Y	Y																																				
15363	A_23_P30923	HLA-DOA	Y	Y	Y				Y	Y																	Y																			
9510	A_23_P30736	HLA-DOB	Y	Y	Y				Y	Y																	Y																			
21790	A_23_P30913	HLA-DPA1																Y												Y						Y										
3721	A_23_P258769	HLA-DPB1			Y																																									
20067	A_23_P59167	HLA-DPB2	Y	Y																																										
417	A_23_P30900	HLA-DQA1	Y		Y	Y	Y	Y	Y	Y	Y	Y															Y	Y	Y	Y						Y										
2000	A_23_P30721	HLA-DQA1	Y	Y	Y		Y				Y									Y							Y																			
7222	A_23_P96115	HLA-DQB1	Y		Y	Y		Y	Y	Y									Y	Y	Y	Y	Y				Y	Y	Y	Y						Y										
19323	A_23_P19611	HLA-DQB1	Y		Y	Y		Y	Y	Y									Y	Y			Y				Y	Y	Y	Y																
5934	A_23_P19510	HLA-DQB2	Y		Y	Y	Y	Y	Y	Y	Y	Y											Y				Y	Y	Y	Y						Y										
591	A_23_P19501	HLA-DRA	Y	Y	Y	Y	Y	Y	Y	Y	Y	Y								Y							Y			Y						Y										
22247	A_23_P156722	HLA-DRB1			Y	Y	Y	Y	Y	Y	Y	Y	Y	Y						Y							Y	Y	Y	Y						Y										
11563	A_23_P145341	HLA-DRB3	Y		Y	Y		Y	Y	Y									Y	Y			Y				Y																			
22099	A_23_P42407	HLA-DRB4			Y		Y		Y	Y	Y	Y								Y	Y	Y	Y				Y																			
12876	A_23_P31006	HLA-DRB5	Y		Y	Y	Y	Y	Y	Y	Y	Y	Y	Y					Y	Y		Y	Y				Y	Y	Y	Y						Y										
18988	A_23_P45099	HLA-DRB5			Y	Y		Y		Y																	Y	Y	Y	Y																
11653	A_23_P30848	HLA-E			Y	Y	Y	Y	Y	Y	Y	Y							Y	Y	Y	Y	Y				Y																			
16318	A_23_P145264	HLA-F			Y	Y	Y	Y	Y	Y	Y	Y	Y	Y					Y	Y	Y	Y	Y				Y																			
18709	A_23_P156645	HLA-G			Y	Y	Y	Y	Y	Y	Y	Y	Y						Y	Y	Y	Y	Y				Y	Y	Y	Y																
19279	A_23_P304991	HLCS			Y	Y		Y		Y								Y	Y	Y	Y	Y	Y	Y	Y	Y					Y		Y													
21592	A_23_P356585	HLF			Y													Y				Y																								
4989	A_23_P126263	HLX			Y	Y		Y	Y	Y									Y	Y	Y	Y	Y				Y																			
4988	A_23_P40344	HM13																												Y						Y										
5887	A_23_P134684	HMBOX1	Y	Y	Y	Y	Y	Y	Y	Y	Y	Y							Y	Y			Y				Y	Y	Y	Y						Y						Y				
1153	A_23_P148990	HMCN1			Y	Y	Y	Y	Y	Y	Y	Y	Y	Y				Y	Y	Y	Y	Y		Y			Y	Y	Y	Y						Y										
18600	A_23_P375854	HMCN2																		Y																										
16926	A_23_P42331	HMGA1			Y	Y	Y	Y	Y	Y	Y	Y	Y	Y				Y	Y	Y		Y	Y	Y			Y	Y	Y	Y	Y	Y	Y	Y	Y	Y				Y						
8070	A_23_P95929	HMGA2							Y	Y	Y	Y	Y	Y				Y	Y											Y						Y										
7957	A_23_P155765	HMGB2																					Y																							
9398	A_23_P217236	HMGB3																Y		Y																										
627	A_23_P373119	HMGB3L1			Y	Y		Y		Y								Y		Y							Y																			
10777	A_23_P11697	HMGB4																Y																												
1167	A_23_P30495	HMGCR			Y		Y		Y	Y	Y	Y	Y	Y					Y	Y		Y	Y							Y						Y										
1792	A_23_P399813	HMGCS1													Y																															
13935	A_23_P103588	HMGCS2							Y	Y																																				
6752	A_23_P19389	HMGN4			Y				Y	Y	Y	Y	Y	Y																Y						Y										
15294	A_23_P431981	HMGXB4			Y	Y		Y	Y	Y										Y	Y		Y					Y																		
6277	A_23_P374053	HMHA1											Y														Y																			
10104	A_23_P30204	HMHB1			Y				Y	Y																																				
17664	A_23_P120883	HMOX1							Y	Y																				Y						Y										
16492	A_23_P100501	HMOX2			Y	Y	Y	Y	Y	Y	Y	Y						Y	Y	Y		Y	Y	Y			Y	Y	Y	Y						Y										
16226	A_23_P72924	HMX1			Y	Y		Y	Y	Y								Y		Y		Y	Y				Y			Y						Y										
22104	A_23_P84323	HMX1			Y	Y		Y		Y								Y	Y	Y		Y	Y	Y			Y	Y	Y	Y																
11633	A_23_P100632	HN1	Y	Y	Y													Y	Y	Y			Y	Y			Y																			
20498	A_23_P434900	HN1L									Y		Y	Y																																
20552	A_23_P36757	HNF1A																												Y						Y										
21315	A_23_P424096	HNF4A																				Y					Y			Y						Y										
14937	A_23_P251453	HNF4G			Y	Y	Y	Y	Y	Y	Y	Y						Y	Y	Y		Y	Y	Y			Y	Y	Y	Y																
17095	A_23_P86660	HNRNPA3			Y	Y	Y	Y		Y	Y	Y							Y	Y							Y	Y	Y	Y																
4298	A_23_P19084	HNRNPAB			Y	Y		Y		Y										Y																										
8176	A_23_P11283	HNRNPH2			Y	Y	Y	Y		Y	Y	Y	Y							Y							Y															Y				
21545	A_23_P115824	HNRNPH3			Y	Y	Y	Y	Y	Y	Y	Y	Y	Y						Y							Y	Y	Y	Y																
19607	A_23_P216788	HNRNPK			Y	Y	Y	Y		Y	Y	Y						Y	Y	Y		Y		Y			Y	Y	Y	Y						Y										
10394	A_23_P119677	HNRNPL																		Y		Y	Y																							
2293	A_23_P45733	HNRNPR			Y	Y		Y		Y																	Y	Y	Y	Y						Y										
7405	A_23_P213156	HNRPDL				Y																																								
4604	A_23_P51100	HNRPLL							Y	Y																																				

21864	A_23_P41917	HOMER1				Y			Y	Y																																				
9173	A_23_P99907	HOMER2																		Y																										
13575	A_23_P27964	HOMER3			Y	Y		Y	Y	Y	Y	Y	Y	Y				Y		Y	Y	Y	Y				Y	Y	Y	Y						Y										
16462	A_23_P114872	HOOK1	Y		Y	Y		Y	Y	Y									Y									Y		Y						Y										
17058	A_23_P153436	HOOK2			Y	Y		Y		Y																	Y	Y	Y	Y																
16539	A_23_P216080	HOOK3			Y															Y			Y				Y																			
10609	A_23_P254507	HOPX	Y		Y													Y	Y	Y				Y			Y																			
4445	A_23_P327530	HORMAD1							Y	Y	Y	Y	Y	Y				Y	Y	Y		Y		Y			Y			Y						Y										
16719	A_23_P428366	HORMAD2							Y	Y																																				
22237	A_23_P126162	HPCA			Y	Y	Y	Y	Y	Y	Y	Y	Y	Y				Y		Y	Y	Y	Y				Y	Y	Y	Y			Y													
15565	A_23_P5831	HPCAL1			Y	Y	Y	Y	Y	Y	Y	Y						Y	Y	Y	Y	Y	Y	Y	Y	Y	Y	Y	Y	Y	Y	Y	Y							Y						
18062	A_23_P74449	HPDL			Y	Y		Y	Y	Y	Y	Y	Y	Y				Y	Y	Y		Y		Y			Y	Y	Y	Y						Y										
18438	A_23_P213050	HPGD																Y																												
18398	A_23_P101801	HPN	Y	Y	Y				Y	Y					Y			Y	Y	Y	Y		Y	Y	Y		Y						Y	Y												
58	A_23_P202361	HPS1	Y	Y	Y	Y		Y	Y	Y	Y	Y	Y	Y	Y			Y	Y	Y			Y	Y			Y	Y	Y	Y						Y										
C8741.1	A_23_P40821	HPS3			Y	Y	Y	Y	Y	Y	Y	Y								Y			Y				Y	Y	Y	Y																
7891	A_23_P256107	HPSE	Y	Y	Y	Y		Y	Y	Y					Y			Y		Y		Y					Y			Y						Y										
3521	A_23_P161998	HPX							Y	Y																				Y						Y										
15686	A_23_P500751	HR			Y	Y		Y	Y	Y	Y	Y	Y	Y				Y		Y			Y				Y			Y						Y										
20550	A_23_P256131	HR			Y	Y	Y	Y	Y	Y	Y	Y	Y	Y				Y		Y		Y	Y				Y	Y	Y	Y						Y										
10119	A_23_P98183	HRAS			Y															Y	Y		Y				Y																			
2587	A_23_P57658	HRASLS			Y	Y		Y		Y																		Y																		
3594	A_23_P105012	HRASLS2							Y	Y	Y	Y	Y	Y													Y	Y	Y	Y						Y								Y		
17684	A_23_P53018	HRASLS5			Y																		Y																							
10786	A_23_P142125	HRC							Y	Y																																				
22280	A_23_P94429	HRCT1							Y	Y									Y	Y										Y						Y										
5410	A_23_P412990	HRH3			Y																																									
16007	A_23_P25194	HRK									Y		Y	Y				Y			Y							Y		Y						Y										
18776	A_23_P154166	HS1BP3			Y	Y		Y		Y												Y								Y						Y										
5300	A_23_P121657	HS3ST1	Y	Y	Y	Y		Y	Y	Y										Y								Y		Y						Y										
8984	A_23_P118158	HS3ST2			Y				Y	Y										Y		Y	Y				Y																			
13397	A_23_P66526	HS3ST3A1							Y	Y									Y																											
15999	A_23_P351295	HS3ST5									Y		Y	Y																																
630	A_23_P158444	HS6ST2																Y	Y	Y		Y		Y			Y				Y		Y	Y	Y											
1738	A_23_P169828	HS6ST2			Y						Y		Y	Y				Y	Y	Y			Y	Y			Y	Y	Y	Y	Y	Y	Y	Y	Y	Y										
2129	A_23_P381523	HS6ST3			Y	Y		Y	Y	Y	Y	Y	Y	Y																																
16237	A_23_P32629	HS6ST3																												Y						Y										
14602	A_23_P61222	HSAJ2425				Y							Y						Y	Y		Y						Y		Y						Y										
11604	A_23_P328511	HSBP1			Y	Y	Y	Y	Y	Y	Y	Y						Y	Y		Y									Y						Y										
2870	A_23_P63209	HSD11B1	Y	Y	Y													Y	Y								Y																			
7945	A_23_P141992	HSD11B1L			Y	Y		Y		Y												Y					Y	Y	Y	Y																
7680	A_23_P15542	HSD17B1																	Y																											
2192	A_23_P21644	HSD17B11																					Y																							
11373	A_23_P408271	HSD17B11			Y																						Y																			
17389	A_23_P78762	HSD17B14				Y														Y			Y					Y																		
4217	A_23_P118065	HSD17B2			Y																						Y																			
156	A_23_P92954	HSD17B4	Y	Y	Y	Y	Y	Y	Y	Y	Y	Y	Y	Y						Y							Y	Y	Y	Y						Y										
6119	A_23_P11860	HSD17B7			Y				Y	Y													Y				Y																			
331	A_23_P81973	HSD17B8	Y	Y					Y	Y																		Y		Y						Y										
12470	A_23_P206810	HSD3B7			Y																						Y																			
546	A_23_P206324	HSDL1																					Y																							
4463	A_23_P169197	HSDL2			Y	Y	Y	Y	Y	Y	Y	Y	Y	Y				Y	Y	Y		Y	Y	Y			Y	Y	Y	Y	Y	Y	Y	Y	Y	Y										
15824	A_23_P143512	HSF2BP																Y																												
13275	A_23_P153372	HSH2D											Y							Y										Y						Y										
10739	A_23_P145085	HSP90AB1			Y													Y		Y							Y																			
14155	A_23_P91334	HSPA12B																Y		Y		Y	Y																							
C10579.5	A_23_P17602	HSPA13			Y	Y		Y		Y																	Y																			
15855	A_23_P63829	HSPA14	Y	Y																																										
2505	A_23_P111211	HSPA1A			Y	Y		Y		Y								Y	Y	Y	Y	Y	Y	Y	Y	Y					Y		Y													
6058	A_23_P88303	HSPA2			Y																Y						Y																			
10611	A_23_P83220	HSPA5			Y	Y		Y	Y	Y									Y	Y		Y	Y					Y																		
19606	A_23_P114903	HSPA6							Y	Y																																				
1224	A_23_P24597	HSPA8			Y	Y		Y		Y											Y																									
10354	A_23_P213973	HSPA9			Y	Y		Y	Y	Y																	Y	Y	Y	Y																
19825	A_23_P257704	HSPB1																	Y	Y																										
11349	A_23_P96872	HSPB11																					Y																							
22505	A_23_P153815	HSPB6			Y																						Y																			
6662	A_23_P103703	HSPB7			Y																						Y	Y	Y	Y																
18160	A_23_P162579	HSPB8																		Y		Y																								
20502	A_23_P416212	HSPB9			Y	Y		Y		Y									Y	Y		Y	Y				Y																			
20729	A_23_P250118	HSPBAP1				Y			Y	Y																				Y						Y										
12288	A_23_P170687	HSPBBP1			Y													Y	Y	Y	Y	Y		Y			Y																			
11588	A_23_P74252	HSPC157																				Y						Y																		
11005	A_23_P210330	HSPC159			Y														Y	Y																										
16118	A_23_P430818	HSPC159							Y	Y			Y																	Y						Y										
18658	A_23_P64129	HTATIP2			Y	Y		Y	Y	Y	Y	Y	Y	Y														Y		Y						Y										
1348	A_23_P45345	HTATSF1				Y																																								

19637	A_23_P212945	HTN3																										Y		Y								Y											
4456	A_23_P69834	HTR1A																		Y								Y																					
13250	A_23_P70648	HTR1E																Y	Y		Y																									
16800	A_23_P16953	HTR2B			Y																																								
4331	A_23_P433586	HTR2C																		Y		Y																								
21736	A_23_P170944	HTR2C																					Y																							
10583	A_23_P74766	HTR6				Y													Y										Y																		
5799	A_23_P500381	HTR7			Y	Y	Y	Y	Y	Y	Y	Y	Y	Y				Y	Y	Y	Y			Y			Y																			
15500	A_23_P1211	HTR7																Y																												
6244	A_23_P97990	HTRA1																Y	Y	Y		Y		Y				Y																		
18027	A_23_P17006	HTRA2			Y															Y			Y																							
16341	A_23_P44421	HTRA4	Y		Y																						Y																			
17035	A_23_P212749	HTT			Y	Y		Y	Y	Y																	Y	Y	Y	Y							Y										
21923	A_23_P113841	HUMBINDC							Y	Y																				Y							Y										
2548	A_23_P156976	HUS1			Y																						Y	Y	Y	Y																
8287	A_23_P133739	HUS1B			Y																																								
19083	A_23_P151166	HVCN1	Y		Y										Y																														
13804	A_23_P57927	HYAL2			Y	Y		Y		Y								Y	Y	Y	Y	Y	Y	Y	Y	Y					Y		Y													
6030	A_23_P250228	HYAL4							Y	Y																																			
691	A_23_P88996	HYDIN			Y																																								
21191	A_23_P127522	HYLS1																		Y																									
7261	A_23_P75642	HYOU1			Y	Y	Y	Y	Y	Y	Y	Y	Y	Y				Y	Y	Y		Y	Y	Y			Y			Y							Y						Y				
17403	A_23_P117683	HYPK							Y	Y	Y	Y	Y	Y							Y									Y							Y										
2722	A_23_P17287	IAH1																		Y																									
1728	A_23_P139648	IAPP			Y																						Y																			
6674	A_23_P74843	IARS2			Y															Y							Y																			
10271	A_23_P358995	IBTK			Y	Y	Y	Y	Y	Y	Y	Y	Y	Y						Y		Y	Y				Y	Y	Y	Y							Y										
19439	A_23_P215419	ICA1			Y	Y		Y	Y	Y	Y	Y	Y	Y						Y			Y							Y							Y										
9762	A_23_P17226	ICA1L			Y																									Y							Y										
18652	A_23_P153320	ICAM1	Y	Y	Y	Y		Y	Y	Y	Y	Y	Y	Y														Y		Y							Y										
21857	A_23_P152655	ICAM2	Y																Y																											
14631	A_23_P164691	ICAM3			Y	Y		Y	Y	Y									Y								Y																			
11730	A_23_P119143	ICAM5																		Y																									
2871	A_23_P371215	ICOS	Y	Y	Y																						Y																			
16917	A_23_P108893	ICOS			Y																																								
12790	A_23_P6272	ICOSLG			Y		Y		Y	Y	Y	Y	Y	Y				Y	Y	Y				Y			Y			Y							Y										
18180	A_23_P166280	ICOSLG																										Y																		
9934	A_23_P141194	ICT1																										Y																		
5693	A_23_P252306	ID1																					Y							Y							Y										
13119	A_23_P137381	ID3			Y				Y	Y										Y	Y		Y																							
21516	A_23_P59375	ID4							Y	Y																				Y							Y										
15099	A_23_P5376	IDH1				Y			Y	Y																				Y							Y										
16277	A_23_P129204	IDH2	Y		Y				Y	Y					Y			Y		Y							Y																			
1117	A_23_P140668	IDH3A																			Y																								
15708	A_23_P166068	IDH3B				Y			Y	Y																				Y							Y										
109	A_23_P34113	IDH3G			Y	Y	Y	Y	Y	Y	Y	Y						Y	Y	Y		Y	Y	Y			Y	Y	Y	Y							Y										
17131	A_23_P112026	IDO1	Y	Y	Y	Y	Y	Y	Y	Y	Y	Y								Y							Y	Y	Y	Y																
17615	A_23_P217470	IDS			Y																																								
10415	A_23_P42257	IER3	Y	Y	Y													Y		Y		Y	Y				Y																			
15803	A_23_P4474	IER3IP1																		Y																									
14489	A_23_P86330	IER5																Y										Y																		
20509	A_23_P217866	IFI16	Y	Y																																									
C7914.5	A_23_P48513	IFI27			Y	Y	Y	Y	Y	Y	Y	Y	Y	Y									Y				Y																			
3803	A_23_P53976	IFI27L1																		Y																									
20016	A_23_P153745	IFI30	Y	Y	Y	Y	Y	Y	Y	Y	Y	Y			Y				Y	Y	Y						Y			Y							Y										
19513	A_23_P152782	IFI35			Y	Y	Y	Y		Y	Y	Y																																	
1553	A_23_P73290	IFITM3				Y																								Y							Y										
7867	A_23_P87539	IFITM3																									Y																			
1735	A_23_P150664	IFITM5			Y	Y		Y	Y	Y										Y		Y	Y																							
19661	A_23_P311050	IFLTD1																												Y							Y										
2064	A_23_P135146	IFNA1			Y		Y		Y	Y	Y	Y															Y			Y							Y										
527	A_23_P9310	IFNA16																			Y																								
5735	A_23_P157920	IFNA16				Y																																							
913	A_23_P250251	IFNA2																									Y																			
5824	A_23_P211083	IFNAR2			Y				Y	Y																																			
14330	A_23_P151294	IFNG	Y	Y	Y				Y	Y																	Y			Y							Y										
19546	A_23_P8281	IFNGR1			Y														Y								Y			Y							Y										
17282	A_23_P29034	IFNGR2			Y																						Y																			
5825	A_23_P251825	IFRD1			Y																																								
17137	A_23_P212440	IFT122																	Y	Y	Y		Y				Y			Y							Y										
3806	A_23_P140725	IFT140			Y	Y		Y	Y	Y																				Y							Y										
17185	A_23_P165962	IFT52							Y	Y										Y																									
19966	A_23_P255711	IFT74			Y	Y	Y	Y	Y	Y	Y	Y							Y	Y		Y	Y				Y	Y	Y	Y	Y	Y	Y	Y	Y	Y										
13107	A_23_P72680	IFT81	Y		Y	Y	Y	Y	Y	Y	Y	Y	Y	Y				Y	Y	Y	Y	Y	Y	Y	Y	Y	Y	Y	Y	Y	Y	Y	Y	Y	Y	Y										
16425	A_23_P48339	IFT88	Y	Y	Y	Y	Y	Y	Y	Y	Y	Y	Y	Y	Y			Y	Y	Y	Y	Y	Y	Y	Y	Y	Y	Y	Y	Y	Y	Y				Y				Y						
20518	A_23_P171249	IGBP1							Y	Y								Y	Y	Y		Y	Y	Y						Y							Y										
9002	A_23_P13907	IGF1																					Y																							

14341	A_23_P205989	IGF1R																									Y																			
16568	A_23_P417282	IGF1R	Y	Y	Y	Y	Y	Y	Y	Y	Y	Y						Y	Y	Y	Y	Y	Y	Y	Y	Y	Y	Y	Y	Y	Y	Y				Y										
8197	A_23_P203458	IGF2			Y																		Y				Y			Y						Y										
21439	A_23_P421379	IGF2																		Y	Y	Y	Y																							
21991	A_23_P150609	IGF2			Y																						Y	Y	Y	Y						Y										
3764	A_23_P72985	IGF2BP1																				Y	Y																							
9998	A_23_P343954	IGF2BP1	Y	Y																							Y																			
C16062.7	A_23_P83498	IGF2BP1			Y																						Y																			
15256	A_23_P250156	IGF2BP2				Y			Y	Y																																				
21578	A_23_P19987	IGF2BP3							Y	Y																																				
1723	A_23_P334021	IGF2R			Y	Y		Y		Y																																				
13825	A_23_P156951	IGF2R																		Y	Y		Y																							
22350	A_23_P14892	IGFALS	Y	Y	Y	Y	Y	Y	Y	Y	Y	Y	Y	Y	Y			Y									Y	Y	Y	Y													Y		
8299	A_23_P42869	IGFBP1	Y	Y	Y															Y							Y			Y						Y										
14689	A_23_P119943	IGFBP2	Y		Y	Y		Y	Y	Y																	Y	Y	Y	Y																
13999	A_23_P154115	IGFBP5																									Y																			
11667	A_23_P128520	IGFBP6																		Y	Y	Y	Y																							
12031	A_23_P94711	IGFBPL1				Y																						Y		Y						Y										
6411	A_23_P158817	IGH@			Y																		Y																							
20906	A_23_P218128	IGH@	Y		Y														Y	Y		Y					Y																			
876	A_23_P124632	IGHA1	Y		Y	Y	Y	Y	Y	Y	Y	Y						Y	Y	Y				Y			Y	Y	Y	Y																
16161	A_23_P393713	IGHMBP2																		Y	Y	Y																								
8253	A_23_P167168	IGJ			Y																																								
6954	A_23_P68273	IGKV1-5	Y		Y				Y	Y								Y	Y	Y				Y			Y																			
9057	A_23_P32605	IGL@	Y	Y	Y	Y		Y	Y	Y																	Y			Y						Y										
C10869	A_23_P9788	IGL@			Y		Y		Y	Y	Y	Y	Y	Y				Y	Y	Y				Y			Y																			
12787	A_23_P120819	IGLL1	Y		Y	Y	Y	Y	Y	Y	Y	Y	Y	Y				Y	Y	Y				Y			Y																			
11319	A_23_P153208	IGLON5			Y																						Y																			
13471	A_23_P60848	IGLV3-25	Y		Y	Y	Y	Y	Y	Y	Y	Y	Y	Y				Y	Y	Y				Y			Y																			
2999	A_23_P125303	IGSF1			Y																						Y																			
16301	A_23_P333683	IGSF10			Y				Y	Y										Y							Y			Y						Y										
2271	A_23_P95899	IGSF21																									Y	Y	Y	Y																
20439	A_23_P126913	IGSF3																		Y																										
11169	A_23_P106629	IGSF6	Y		Y				Y	Y										Y			Y				Y																			
5157	A_23_P137418	IGSF8			Y	Y		Y		Y										Y	Y		Y				Y																			
1594	A_23_P133245	IK			Y	Y		Y	Y	Y																	Y	Y	Y	Y						Y										
14209	A_23_P169189	IKBKAP			Y	Y	Y	Y	Y	Y	Y	Y	Y	Y				Y	Y	Y	Y	Y	Y	Y	Y	Y	Y	Y	Y	Y	Y	Y	Y	Y	Y	Y										
21297	A_23_P216188	IKBKB			Y																																								
7333	A_23_P887	IKBKE																									Y																			
9028	A_23_P159920	IKBKG			Y	Y	Y	Y	Y	Y	Y	Y	Y	Y				Y	Y	Y	Y	Y	Y	Y	Y	Y	Y	Y	Y	Y	Y	Y	Y	Y	Y	Y										
2300	A_23_P147043	IKZF1			Y															Y																										
8408	A_23_P124934	IKZF1			Y	Y		Y		Y			Y														Y			Y						Y										
4173	A_23_P35667	IKZF5			Y	Y		Y	Y	Y										Y		Y	Y				Y	Y	Y	Y																
2194	A_23_P203173	IL10RA	Y		Y				Y	Y					Y				Y	Y			Y				Y																			
21472	A_23_P6231	IL10RB			Y																						Y																			
11635	A_23_P67169	IL11	Y	Y	Y													Y					Y				Y																			
21973	A_23_P71867	IL11RA																Y	Y	Y		Y		Y			Y			Y						Y										
5378	A_23_P80545	IL12A																Y	Y																											
1693	A_23_P101783	IL12RB1			Y	Y	Y	Y	Y	Y	Y	Y															Y			Y						Y										
13849	A_23_P72077	IL12RB2			Y	Y	Y	Y	Y	Y	Y	Y							Y																											
10490	A_23_P58627	IL13																			Y																								
6530	A_23_P137196	IL13RA1							Y	Y																																				
14829	A_23_P29953	IL15			Y																																								
22445	A_23_P138680	IL15RA	Y	Y	Y	Y	Y	Y	Y	Y	Y	Y	Y	Y									Y				Y	Y	Y	Y						Y							Y			
649	A_23_P61057	IL16			Y										Y				Y	Y							Y																			
8351	A_23_P332820	IL17A																			Y																								
22294	A_23_P156489	IL17A				Y			Y	Y																	Y			Y						Y										
1169	A_23_P167479	IL17B																	Y																										
9837	A_23_P49097	IL17C			Y	Y		Y		Y								Y	Y	Y		Y	Y	Y			Y	Y	Y	Y						Y										
3276	A_23_P167882	IL17F																	Y																										
22409	A_23_P17708	IL17RA			Y	Y		Y	Y	Y										Y		Y						Y																		
18755	A_23_P376044	IL17RB	Y		Y	Y		Y		Y								Y		Y			Y				Y																			
21767	A_23_P166767	IL17RC			Y	Y		Y		Y								Y		Y							Y																			
1060	A_23_P500206	IL17RE			Y	Y		Y	Y	Y	Y	Y	Y	Y					Y								Y	Y	Y	Y						Y										
5370	A_23_P104798	IL18	Y	Y	Y																																								
19250	A_23_P203622	IL18BP	Y		Y				Y	Y					Y			Y		Y							Y			Y						Y										
16291	A_23_P28334	IL18RAP																									Y																			
9110	A_23_P79518	IL1B																										Y		Y						Y										
20602	A_23_P255003	IL1F10			Y																		Y																							
12678	A_23_P28462	IL1F6																Y																											
4407	A_23_P5654	IL1F7																					Y																							
7699	A_23_P90925	IL1F8																	Y																										
6051	A_23_P68006	IL1R1																			Y																								
15172	A_23_P79398	IL1R2	Y	Y	Y														Y	Y								Y																		
7470	A_23_P336554	IL1RAP																					Y																							
17611	A_23_P170857	IL1RAP																	Y																										
3988	A_23_P84106	IL1RAPL1			Y				Y	Y																																				

15581	A_23_P56604	IL1RL2																		Y																										
14874	A_23_P401461	IL20							Y	Y	Y	Y	Y	Y													Y			Y						Y										
18611	A_23_P145514	IL20RA			Y				Y	Y	Y	Y	Y	Y						Y	Y						Y			Y						Y								Y		
15847	A_23_P206572	IL21R							Y	Y																																				
4035	A_23_P427516	IL22			Y																						Y	Y	Y	Y																
18246	A_23_P117115	IL22			Y	Y		Y		Y																																				
21225	A_23_P62610	IL22RA1																		Y																										
2552	A_23_P168288	IL22RA2			Y	Y	Y	Y	Y	Y	Y	Y	Y	Y													Y			Y						Y										
21587	A_23_P76078	IL23A																									Y																			
7853	A_23_P51951	IL24																												Y						Y										
273	A_23_P128503	IL26			Y	Y		Y		Y																																				
7582	A_23_P27606	IL27RA																	Y			Y																								
16287	A_23_P409438	IL28A			Y				Y	Y																				Y						Y										
21569	A_23_P373619	IL28B			Y																																									
281	A_23_P74112	IL28RA			Y													Y																												
9395	A_23_P337800	IL29																			Y																									
15445	A_23_P127288	IL2RA	Y	Y	Y	Y	Y	Y	Y	Y	Y	Y						Y		Y	Y						Y	Y	Y	Y						Y										
14729	A_23_P255430	IL2RB							Y	Y																																				
18558	A_23_P148464	IL2RG	Y		Y	Y	Y	Y	Y	Y	Y	Y							Y								Y																			
7844	A_23_P348828	IL3							Y	Y																																				
20583	A_23_P15146	IL32	Y	Y	Y	Y	Y	Y	Y	Y	Y	Y	Y	Y				Y	Y	Y			Y	Y			Y	Y	Y	Y	Y	Y	Y	Y	Y	Y										
16924	A_23_P31945	IL33			Y														Y				Y				Y																			
12035	A_23_P100380	IL34	Y		Y	Y		Y	Y	Y								Y	Y	Y		Y	Y	Y			Y			Y						Y										
18109	A_23_P45115	IL3RA			Y														Y				Y				Y						Y	Y												
21786	A_23_P253081	IL3RA	Y		Y	Y	Y	Y	Y	Y	Y	Y	Y	Y	Y			Y	Y	Y	Y			Y			Y	Y	Y	Y			Y	Y		Y										
7598	A_23_P213706	IL4																												Y						Y										
22205	A_23_P502520	IL4I1	Y	Y	Y	Y	Y	Y	Y	Y	Y	Y	Y	Y	Y					Y							Y	Y	Y	Y						Y										
19306	A_23_P129556	IL4R																					Y																							
20431	A_23_P71037	IL6																										Y																		
2475	A_23_P160776	IL6R			Y	Y		Y	Y	Y								Y	Y	Y	Y	Y	Y	Y	Y	Y	Y				Y															
6308	A_23_P58658	IL6ST	Y	Y	Y	Y		Y	Y	Y	Y	Y	Y	Y				Y	Y	Y		Y	Y	Y			Y	Y	Y	Y						Y								Y		
18804	A_23_P502470	IL6ST	Y	Y	Y	Y		Y	Y	Y	Y	Y	Y	Y				Y	Y	Y			Y	Y			Y	Y	Y	Y						Y								Y		
7357	A_23_P8961	IL7			Y													Y		Y							Y																			
14480	A_23_P404494	IL7R	Y		Y																						Y																			
19005	A_23_P81006	IL8			Y	Y		Y	Y	Y																	Y	Y	Y	Y						Y										
3248	A_23_P122163	IL9							Y	Y																	Y			Y						Y										
14316	A_23_P34066	IL9R				Y																								Y						Y										
9239	A_23_P109910	ILDR1			Y	Y	Y	Y	Y	Y	Y	Y	Y	Y				Y		Y	Y						Y	Y	Y	Y						Y										
17207	A_23_P257956	ILF2																	Y																											
7968	A_23_P435987	ILF3																Y	Y	Y		Y	Y	Y																						
9186	A_23_P218456	ILF3			Y													Y	Y	Y		Y		Y			Y																			
12742	A_23_P105063	ILK																		Y	Y	Y	Y																							
12481	A_23_P165148	ILVBL			Y				Y	Y			Y					Y		Y		Y					Y			Y						Y										
21820	A_23_P203305	IMMP1L																			Y																									
10070	A_23_P169800	IMMP2L																		Y																										
8727	A_23_P33803	IMP3			Y																																									
1215	A_23_P131519	IMP4																Y	Y	Y		Y		Y				Y																		
20796	A_23_P302094	IMP4			Y	Y	Y	Y	Y	Y	Y	Y	Y	Y					Y	Y		Y					Y	Y	Y	Y						Y										
3148	A_23_P50081	IMPA2			Y	Y	Y	Y	Y	Y	Y	Y	Y	Y				Y	Y	Y	Y	Y		Y			Y	Y	Y	Y						Y								Y		
2804	A_23_P208070	IMPACT			Y															Y			Y				Y																			
20196	A_23_P136232	IMPAD1	Y	Y		Y			Y	Y								Y																												
15100	A_23_P157237	IMPDH1			Y	Y	Y	Y	Y	Y	Y	Y						Y	Y	Y	Y	Y	Y	Y	Y	Y	Y	Y	Y	Y	Y	Y				Y										
13667	A_23_P18119	IMPG2				Y																								Y						Y										
5172	A_23_P35444	INA							Y	Y													Y				Y																			
1582	A_23_P126100	INADL				Y																	Y					Y		Y						Y										
5005	A_23_P116387	INCENP																		Y																										
628	A_23_P148602	INE1			Y	Y		Y		Y																		Y		Y						Y										
6835	A_23_P99437	ING1																					Y																							
10791	A_23_P83736	ING2			Y													Y		Y							Y																			
11465	A_23_P48070	ING4											Y							Y										Y						Y										
16844	A_23_P135595	ING5																		Y		Y																								
15011	A_23_P125687	INGX																	Y	Y			Y																							
9042	A_23_P122924	INHBA																	Y			Y																								
1192	A_23_P144223	INHBE			Y																		Y				Y			Y						Y										
4484	A_23_P70961	INMT																		Y			Y																							
14747	A_23_P106522	INO80			Y	Y		Y	Y	Y										Y										Y						Y										
6286	A_23_P209652	INO80D							Y	Y																				Y						Y										
19161	A_23_P385267	INO80E			Y	Y		Y		Y									Y	Y		Y					Y																			
3054	A_23_P393531	INPP4A	Y	Y	Y	Y		Y	Y	Y	Y	Y	Y	Y	Y			Y	Y	Y		Y	Y	Y			Y			Y						Y								Y		
14765	A_23_P56768	INPP4A	Y	Y	Y	Y		Y	Y	Y									Y								Y	Y	Y	Y						Y										
7236	A_23_P18559	INPP4B	Y	Y	Y				Y	Y	Y	Y	Y	Y					Y	Y							Y			Y						Y										
8230	A_23_P202307	INPP5A			Y				Y	Y								Y	Y	Y		Y	Y	Y			Y																			
2793	A_23_P22332	INPP5D			Y	Y	Y	Y	Y	Y	Y	Y							Y	Y			Y				Y																			
11669	A_23_P94878	INPP5E																Y		Y																										
15630	A_23_P35684	INPP5F			Y	Y	Y	Y	Y	Y	Y	Y	Y	Y					Y	Y							Y	Y	Y	Y						Y										
16482	A_23_P91669	INPP5J	Y	Y	Y	Y		Y	Y	Y	Y	Y	Y	Y													Y	Y	Y	Y						Y										
9789	A_23_P1981	INS				Y																																								

18335	A_23_P22027	INSIG1																Y																												
13166	A_23_P102454	INSIG2			Y																																									
284	A_23_P209006	INSL3																										Y																		
10337	A_23_P112220	INSL4	Y		Y													Y		Y		Y					Y																			
15184	A_23_P51479	INSL5			Y	Y		Y		Y										Y	Y	Y	Y																							
12374	A_23_P109185	INSM1			Y	Y		Y	Y	Y										Y													Y													
14854	A_23_P614	INSRR			Y				Y	Y																	Y			Y						Y										
20116	A_23_P10433	INTS1			Y	Y	Y	Y		Y	Y	Y						Y	Y	Y		Y	Y	Y			Y																			
3364	A_23_P81087	INTS12																		Y			Y																							
1676	A_23_P420269	INTS2			Y																						Y																			
14833	A_23_P203737	INTS4			Y															Y		Y																								
14133	A_23_P25653	INTS6			Y	Y	Y	Y		Y	Y	Y			Y			Y	Y	Y		Y		Y			Y																			
18913	A_23_P8848	INTS9																			Y																									
20560	A_23_P18234	IP6K2			Y	Y		Y	Y	Y										Y		Y					Y			Y						Y										
9746	A_23_P380371	IPMK							Y	Y																																				
9618	A_23_P384600	IPO13			Y	Y		Y		Y								Y	Y	Y		Y		Y			Y	Y	Y	Y																
13757	A_23_P162970	IPO4			Y	Y		Y		Y								Y	Y	Y		Y	Y	Y			Y																			
16970	A_23_P205007	IPO5			Y				Y	Y										Y	Y									Y						Y										
6380	A_23_P150714	IPO7																										Y																		
3558	A_23_P98923	IPO8																										Y																		
8017	A_23_P97159	IPO9																Y	Y	Y	Y	Y		Y																						
8071	A_23_P9662	IPP																					Y																							
10069	A_23_P123587	IPPK																Y	Y	Y		Y		Y																						
8741	A_23_P143068	IQCA1	Y		Y	Y		Y	Y	Y								Y	Y	Y		Y	Y	Y			Y	Y	Y	Y						Y										
22209	A_23_P73195	IQCA1							Y	Y									Y	Y		Y	Y																							
4688	A_23_P84140	IQCB1																		Y																										
13220	A_23_P74162	IQCC			Y	Y		Y		Y																				Y						Y										
4168	A_23_P150993	IQCD							Y	Y	Y	Y	Y	Y													Y																			
1425	A_23_P399756	IQCE			Y	Y	Y	Y	Y	Y	Y	Y						Y	Y	Y		Y	Y	Y			Y	Y	Y	Y						Y										
11919	A_23_P19852	IQCE			Y	Y		Y	Y	Y	Y	Y	Y	Y						Y		Y					Y	Y	Y	Y						Y										
11736	A_23_P316490	IQCF1			Y														Y								Y																			
9257	A_23_P29608	IQCF5																										Y																		
9076	A_23_P124381	IQCG							Y	Y	Y	Y	Y	Y					Y	Y			Y							Y						Y										
9077	A_23_P300070	IQCG							Y	Y	Y	Y	Y	Y						Y			Y							Y						Y										
714	A_23_P324523	IQCK	Y	Y	Y	Y		Y	Y	Y	Y	Y	Y	Y				Y	Y	Y	Y	Y	Y	Y	Y	Y	Y	Y	Y	Y	Y	Y	Y	Y	Y	Y		Y				Y			Y	Y
12915	A_23_P151985	IQGAP1			Y	Y		Y		Y													Y				Y	Y	Y	Y						Y										
15583	A_23_P253002	IQGAP2							Y	Y								Y	Y	Y		Y		Y				Y		Y						Y										
10018	A_23_P6741	IQSEC1			Y	Y		Y	Y	Y																	Y																			
22548	A_23_P349373	IQSEC3																										Y																		
19217	A_23_P349504	IQUB							Y	Y																																				
7832	A_23_P73780	IRAK1			Y	Y	Y	Y	Y	Y	Y	Y	Y	Y				Y	Y	Y	Y	Y	Y	Y	Y	Y	Y	Y	Y	Y	Y	Y				Y										
17210	A_23_P13978	IRAK4																		Y			Y				Y																			
4231	A_23_P41765	IRF1	Y	Y	Y	Y	Y	Y	Y	Y	Y	Y						Y	Y	Y			Y	Y			Y	Y	Y	Y																
16151	A_23_P136473	IRF2																		Y																										
15010	A_23_P27677	IRF3			Y	Y		Y		Y								Y	Y	Y		Y		Y																						
2435	A_23_P214361	IRF4			Y		Y		Y	Y	Y	Y	Y	Y					Y								Y																			
10189	A_23_P500271	IRF5																									Y																			
7443	A_23_P748	IRF6																Y	Y																											
20829	A_23_P127374	IRF7			Y	Y		Y		Y										Y		Y	Y				Y																			
13693	A_23_P129636	IRF8	Y	Y	Y				Y	Y					Y			Y	Y	Y				Y			Y																			
17231	A_23_P332190	IRF8	Y	Y	Y	Y		Y		Y					Y			Y	Y	Y				Y			Y	Y	Y	Y																
12639	A_23_P65442	IRF9																	Y	Y																										
2252	A_23_P165072	IRGQ			Y																						Y																			
8487	A_23_P90649	IRS1	Y	Y	Y	Y		Y		Y																	Y			Y						Y										
15085	A_23_P53838	IRS2																		Y			Y																							
17295	A_23_P133457	IRX1																					Y																							
18107	A_23_P156025	IRX2			Y													Y	Y	Y	Y			Y			Y																			
15373	A_23_P152237	IRX3							Y	Y										Y		Y	Y																							
241	A_23_P216766	ISCA1			Y																																									
7807	A_23_P151746	ISCA2							Y	Y																																				
9518	A_23_P340158	ISCU			Y				Y	Y																	Y																			
4186	A_23_P32404	ISG20			Y		Y		Y	Y	Y	Y	Y	Y						Y							Y			Y						Y										
20722	A_23_P126457	ISG20L2																	Y			Y																								
2389	A_23_P81529	ISL1																												Y						Y										
2733	A_23_P54471	ISL2																	Y	Y							Y																			
10075	A_23_P250982	ISOC1							Y	Y																	Y																			
11228	A_23_P153418	ISOC2			Y	Y		Y		Y									Y	Y		Y																								
2604	A_23_P155179	ISY1																					Y																							
19551	A_23_P124164	ISY1			Y				Y	Y	Y	Y	Y	Y						Y		Y																								
21701	A_23_P5131	ISYNA1																	Y	Y			Y																							
16067	A_23_P334123	ITFG1			Y																																									
2961	A_23_P66124	ITFG3			Y													Y	Y	Y		Y	Y	Y																						
16559	A_23_P256332	ITGA1																	Y								Y																			
6968	A_23_P206021	ITGA11																Y	Y																											
777	A_23_P70161	ITGA2	Y																											Y						Y										
20144	A_23_P77971	ITGA2B			Y		Y				Y		Y	Y				Y	Y	Y	Y	Y	Y	Y	Y	Y	Y			Y	Y	Y				Y										
14041	A_23_P36554	ITGA5																		Y			Y																							

12517	A_23_P210176	ITGA6																										Y		Y						Y										
910	A_23_P128084	ITGA7			Y	Y		Y		Y																	Y																			
4670	A_23_P252193	ITGA9				Y																								Y						Y										
7407	A_23_P129665	ITGAD			Y	Y		Y	Y	Y									Y	Y			Y																							
343	A_23_P218378	ITGAE				Y																																								
8795	A_23_P206806	ITGAL			Y		Y				Y								Y	Y			Y				Y																			
4199	A_23_P251749	ITGAM																									Y	Y	Y	Y																
11361	A_23_P124108	ITGAM	Y																																											
271	A_23_P50907	ITGAV																	Y																											
21776	A_23_P381984	ITGAV																	Y																											
3456	A_23_P118281	ITGAX			Y	Y		Y	Y	Y																																				
6911	A_23_P312132	ITGAX			Y	Y		Y		Y																	Y																			
16546	A_23_P104193	ITGB1																	Y																											
7821	A_23_P154507	ITGB1BP1	Y	Y					Y	Y																				Y						Y										
21512	A_23_P45424	ITGB1BP2	Y	Y	Y														Y								Y																			
14599	A_23_P208825	ITGB1BP3																		Y																										
19599	A_23_P211180	ITGB2	Y		Y	Y	Y	Y	Y	Y	Y	Y			Y												Y	Y	Y	Y																
C13365.2	A_23_P23765	ITGB3BP																		Y			Y																							
3697	A_23_P166627	ITGB5																	Y								Y																			
1836	A_23_P76529	ITGB7																Y																												
13043	A_23_P123060	ITGB8													Y																															
15838	A_23_P18223	ITIH1			Y																						Y	Y	Y	Y						Y										
4216	A_23_P202053	ITIH2	Y	Y	Y	Y	Y	Y	Y	Y	Y	Y	Y	Y				Y	Y	Y		Y		Y			Y	Y	Y	Y						Y										
625	A_23_P6821	ITIH3			Y																						Y																			
18570	A_23_P109881	ITIH4																Y																												
2222	A_23_P104252	ITIH5																	Y																											
7358	A_23_P97841	ITIH5																	Y																											
12389	A_23_P411993	ITIH5			Y													Y		Y		Y	Y																							
15342	A_23_P144740	ITK			Y																									Y						Y										
11665	A_23_P84388	ITLN1							Y	Y	Y	Y	Y	Y																Y						Y										
13353	A_23_P95790	ITLN1				Y												Y																												
18016	A_23_P209750	ITM2C			Y	Y	Y	Y	Y	Y	Y	Y							Y	Y	Y	Y	Y				Y																			
16361	A_23_P68529	ITPA			Y																						Y																			
2815	A_23_P37399	ITPK1																		Y										Y						Y										
14103	A_23_P65918	ITPKA			Y	Y		Y		Y																																				
2681	A_23_P372255	ITPKB																		Y																										
19994	A_23_P92042	ITPR1			Y	Y	Y	Y	Y	Y	Y	Y						Y	Y	Y		Y		Y			Y	Y	Y	Y						Y										
4669	A_23_P203933	ITPR2			Y	Y		Y	Y	Y									Y	Y		Y	Y				Y	Y	Y	Y	Y	Y	Y	Y	Y											
20186	A_23_P75122	ITPRIP			Y	Y	Y	Y	Y	Y	Y	Y	Y	Y				Y		Y	Y	Y	Y				Y	Y	Y	Y	Y	Y	Y	Y	Y	Y				Y						
4158	A_23_P252962	ITSN1				Y																																								
13800	A_23_P56457	ITSN2			Y	Y	Y	Y	Y	Y	Y	Y						Y	Y	Y		Y	Y	Y			Y	Y	Y	Y																
18775	A_23_P129313	IVD							Y	Y								Y	Y			Y								Y						Y										
6110	A_23_P353529	IVL																		Y																										
20556	A_23_P165566	IWS1			Y															Y			Y																							
2144	A_23_P210763	JAG1			Y	Y		Y		Y								Y	Y	Y		Y		Y			Y	Y	Y	Y						Y										
17585	A_23_P106024	JAG2							Y	Y								Y	Y	Y	Y	Y	Y	Y	Y	Y				Y	Y	Y				Y										
12034	A_23_P90393	JAK3			Y	Y		Y		Y																																				
21428	A_23_P329112	JAK3																		Y			Y				Y																			
8760	A_23_P144274	JAKMIP1																												Y						Y										
15737	A_23_P156387	JAKMIP2																	Y									Y																		
13812	A_23_P120667	JAM2																					Y																							
9711	A_23_P217998	JAM3																									Y			Y						Y										
10408	A_23_P204406	JARID1A			Y																							Y																		
2803	A_23_P201789	JARID1B	Y	Y	Y	Y	Y	Y		Y	Y	Y									Y						Y																			
13317	A_23_P399858	JAZF1			Y													Y	Y			Y					Y																			
8579	A_23_P49145	JCLN							Y	Y																																				
9050	A_23_P258031	JMJD1A			Y	Y	Y	Y		Y	Y	Y							Y	Y		Y	Y				Y																			
2939	A_23_P7783	JMJD1B			Y	Y	Y	Y	Y	Y	Y	Y	Y	Y					Y	Y		Y					Y	Y	Y	Y																
7836	A_23_P74446	JMJD2A																												Y						Y										
2160	A_23_P165051	JMJD2B	Y	Y					Y	Y																		Y		Y						Y										
1084	A_23_P112201	JMJD2C			Y															Y	Y	Y	Y				Y																			
14466	A_23_P127406	JMJD2D																												Y						Y										
9857	A_23_P115523	JMJD4			Y	Y	Y	Y	Y	Y	Y	Y						Y	Y	Y		Y	Y	Y			Y	Y	Y	Y						Y										
20918	A_23_P146873	JMJD5				Y												Y	Y	Y		Y		Y																						
21022	A_23_P218203	JMJD7							Y	Y	Y	Y	Y	Y				Y		Y		Y																						Y		
9427	A_23_P403424	JMJD7-PLA2G4B			Y	Y		Y		Y								Y		Y								Y																		
16776	A_23_P15073	JMJD8																		Y		Y																								
5984	A_23_P6479	JOSD1			Y				Y	Y								Y	Y	Y		Y		Y			Y			Y						Y										
1635	A_23_P67323	JOSD2			Y	Y	Y	Y	Y	Y	Y	Y						Y	Y	Y		Y	Y	Y			Y	Y	Y	Y						Y								Y		
12047	A_23_P394395	JPH2			Y																						Y																			
20198	A_23_P68423	JPH2				Y																																								
984	A_23_P8930	JRK				Y																																								
22049	A_23_P202737	JRKL			Y				Y	Y	Y	Y	Y	Y													Y			Y						Y										
20921	A_23_P34979	JTB																	Y																											
12706	A_23_P54055	JUB	Y		Y													Y	Y	Y		Y		Y			Y																			
10461	A_23_P142368	JUND			Y	Y		Y	Y	Y										Y		Y	Y				Y																			
8412	A_23_P501822	JUP																Y	Y																											

22066	A_23_P72513	JUP																Y		Y																										
4820	A_23_P429950	KAL1			Y	Y		Y		Y								Y	Y	Y		Y		Y			Y	Y	Y	Y																
18487	A_23_P307563	KALRN							Y	Y																																				
14280	A_23_P500130	KANK1							Y	Y					Y								Y				Y																			
2531	A_23_P50426	KANK2				Y														Y							Y			Y						Y										
4481	A_23_P55904	KANK2	Y	Y	Y	Y	Y	Y	Y	Y	Y	Y	Y	Y				Y		Y		Y	Y				Y	Y	Y	Y						Y										
5152	A_23_P131089	KANK3			Y	Y		Y		Y					Y			Y	Y	Y	Y	Y	Y	Y	Y	Y	Y	Y	Y	Y	Y	Y	Y	Y	Y											
4083	A_23_P152487	KARS			Y	Y		Y	Y	Y	Y	Y	Y	Y					Y																											
16697	A_23_P66608	KAT2A							Y	Y																																				
19082	A_23_P138847	KAT5																		Y	Y	Y	Y				Y																			
11234	A_23_P113803	KATNA1																		Y																										
14545	A_23_P117976	KATNB1			Y	Y		Y		Y																	Y	Y	Y	Y																
5181	A_23_P1225	KAZALD1																	Y																											
16960	A_23_P17190	KBTBD10			Y	Y		Y	Y	Y									Y	Y	Y	Y	Y				Y			Y						Y										
15950	A_23_P94319	KBTBD11			Y				Y	Y																	Y			Y						Y										
20916	A_23_P127557	KBTBD3																		Y			Y																							
16208	A_23_P202693	KBTBD4																Y																												
12819	A_23_P392435	KBTBD6				Y			Y	Y													Y																							
19768	A_23_P25605	KBTBD7			Y	Y	Y	Y	Y	Y	Y	Y	Y					Y		Y	Y	Y	Y																							
9292	A_23_P431248	KBTBD8			Y																						Y																			
7186	A_23_P126528	KCNA10			Y																																									
13873	A_23_P137923	KCNA2			Y																						Y			Y						Y										
5436	A_23_P201138	KCNA3							Y	Y			Y																																	
7427	A_23_P36843	KCNA5			Y	Y		Y		Y										Y			Y				Y			Y						Y										
8796	A_23_P417174	KCNA5																	Y																											
5439	A_23_P254542	KCNAB1			Y	Y		Y	Y	Y								Y	Y	Y		Y	Y	Y			Y																			
5841	A_23_P44494	KCNAB1																	Y	Y		Y																								
7113	A_23_P501238	KCNAB2	Y	Y					Y	Y																				Y						Y										
7760	A_23_P1007	KCNAB2			Y																																									
19700	A_23_P370454	KCNAB3				Y																																								
6803	A_23_P79860	KCNB1																Y	Y	Y				Y																						
2087	A_23_P71448	KCNB2			Y	Y	Y	Y	Y	Y	Y	Y															Y			Y						Y										
9261	A_23_P101516	KCNC3																					Y																							
20758	A_23_P12072	KCNC4			Y	Y	Y	Y	Y	Y	Y	Y	Y	Y						Y			Y				Y	Y	Y	Y						Y										
451	A_23_P315772	KCND1																				Y																								
2383	A_23_P217218	KCND1																	Y				Y																							
15263	A_23_P259251	KCND2				Y														Y																										
19501	A_23_P697	KCND3			Y																						Y			Y						Y										
20801	A_23_P120694	KCNE2							Y	Y										Y								Y		Y						Y										
6295	A_23_P24951	KCNE3	Y																																											
1573	A_23_P392575	KCNE4	Y	Y	Y													Y	Y	Y			Y	Y			Y			Y						Y										
12431	A_23_P101983	KCNE4	Y	Y	Y														Y				Y				Y																			
8529	A_23_P210580	KCNG1							Y	Y								Y	Y	Y				Y			Y	Y	Y	Y						Y										
8783	A_23_P61171	KCNG2																	Y																											
14434	A_23_P17301	KCNG3																				Y																								
367	A_23_P365248	KCNG4			Y	Y		Y		Y								Y	Y	Y			Y	Y			Y			Y						Y										
1214	A_23_P74943	KCNH1			Y		Y				Y																Y			Y						Y										
13707	A_23_P168403	KCNH2			Y	Y		Y		Y								Y																												
18379	A_23_P87917	KCNH3																		Y			Y																							
6744	A_23_P147764	KCNH4							Y	Y																				Y						Y										
7035	A_23_P83558	KCNH6			Y	Y		Y	Y	Y																	Y																			
766	A_23_P79554	KCNH7																Y																												
12136	A_23_P170636	KCNH8																									Y																			
9078	A_23_P202189	KCNIP2																		Y																										
9713	A_23_P165737	KCNIP3																		Y			Y																							
1322	A_23_P501781	KCNJ1			Y													Y		Y		Y					Y																			
7715	A_23_P202918	KCNJ1			Y	Y		Y	Y	Y																	Y	Y	Y	Y																
16122	A_23_P1973	KCNJ11																					Y																							
9987	A_23_P129821	KCNJ12																		Y	Y																									
15526	A_23_P312752	KCNJ13																												Y						Y										
16632	A_23_P108775	KCNJ13				Y																																								
17617	A_23_P130764	KCNJ14			Y	Y		Y	Y	Y										Y			Y				Y																			
20473	A_23_P17655	KCNJ15	Y	Y	Y						Y		Y	Y						Y			Y																							
10	A_23_P501193	KCNJ16			Y				Y	Y																	Y			Y						Y										
18903	A_23_P55219	KCNJ2			Y																						Y																			
6620	A_23_P153986	KCNJ3	Y	Y																								Y																		
14720	A_23_P257283	KCNJ4			Y															Y	Y	Y	Y																							
19277	A_23_P202927	KCNJ5	Y		Y				Y	Y					Y			Y		Y	Y						Y																			
13905	A_23_P29058	KCNJ6																					Y																							
6860	A_23_P126075	KCNK1	Y	Y																																										
7044	A_23_P37364	KCNK10							Y	Y	Y	Y	Y	Y								Y																								
3905	A_23_P108883	KCNK12			Y													Y	Y	Y		Y	Y	Y			Y																			
13996	A_23_P3171	KCNK13																			Y																									
5987	A_23_P210451	KCNK15			Y				Y	Y	Y	Y	Y	Y													Y			Y						Y										
17299	A_23_P253503	KCNK16																	Y																											
12268	A_23_P42127	KCNK17																					Y																							
21784	A_23_P115932	KCNK18																												Y						Y										

17449	A_23_P91104	KCNK3																Y		Y		Y	Y																							
18869	A_23_P357724	KCNK3																Y	Y	Y			Y	Y			Y																			
13163	A_23_P30728	KCNK5							Y	Y										Y			Y				Y																			
21437	A_23_P319423	KCNK5							Y	Y	Y	Y	Y	Y						Y										Y						Y										
22031	A_23_P50591	KCNK6			Y																									Y						Y										
17192	A_23_P86874	KCNK7	Y	Y	Y	Y	Y	Y	Y	Y	Y	Y	Y	Y					Y	Y		Y	Y				Y	Y	Y	Y						Y										
6517	A_23_P19142	KCNMB1																	Y											Y						Y										
3338	A_23_P135678	KCNMB3			Y																	Y					Y																			
17391	A_23_P119578	KCNN1			Y	Y	Y	Y	Y	Y	Y	Y	Y	Y													Y	Y	Y	Y						Y								Y		
13961	A_23_P500353	KCNN2																				Y																								
16194	A_23_P126167	KCNN3			Y		Y		Y	Y	Y	Y															Y			Y						Y								Y		
16477	A_23_P67529	KCNN4	Y		Y	Y	Y	Y	Y	Y	Y	Y	Y	Y				Y	Y	Y		Y	Y	Y			Y	Y	Y	Y						Y										
14228	A_23_P210400	KCNQ2																					Y																							
20663	A_23_P321840	KCNS1																												Y						Y										
8225	A_23_P111937	KCNS2							Y	Y																																				
6178	A_23_P120105	KCNS3	Y	Y	Y				Y	Y									Y	Y	Y	Y	Y				Y																			
12859	A_23_P146125	KCNV1				Y																																								
15550	A_23_P130347	KCTD1			Y																						Y																			
974	A_23_P162540	KCTD10	Y	Y																																										
14790	A_23_P377957	KCTD12	Y																																											
22221	A_23_P44845	KCTD13			Y				Y	Y										Y		Y	Y				Y			Y						Y										
10012	A_23_P78873	KCTD15			Y				Y	Y								Y	Y	Y		Y	Y	Y			Y																			
14053	A_23_P402157	KCTD17																		Y	Y	Y	Y																							
1828	A_23_P37785	KCTD19			Y																																									
21206	A_23_P374351	KCTD20			Y																																									
13695	A_23_P160406	KCTD3	Y	Y	Y	Y	Y	Y	Y	Y	Y	Y	Y	Y					Y	Y			Y				Y	Y	Y	Y						Y										
19791	A_23_P163759	KCTD5			Y	Y	Y	Y	Y	Y	Y	Y	Y	Y				Y	Y	Y	Y	Y	Y	Y	Y	Y	Y	Y	Y	Y	Y	Y	Y	Y	Y					Y						
792	A_23_P385246	KCTD6	Y	Y	Y	Y	Y	Y	Y	Y	Y	Y								Y			Y				Y	Y	Y	Y						Y										
10855	A_23_P94902	KCTD8																					Y				Y																			
20136	A_23_P43226	KCTD9							Y	Y																																				
C11080	A_23_P128613	KDELC1																												Y						Y										
22332	A_23_P105818	KDELC2			Y	Y		Y	Y	Y	Y	Y	Y	Y				Y	Y	Y	Y	Y	Y	Y	Y	Y	Y	Y	Y	Y	Y	Y	Y	Y	Y	Y										
18330	A_23_P4919	KDELR1			Y	Y	Y	Y		Y	Y	Y						Y		Y			Y				Y																			
16715	A_23_P19938	KDELR2			Y	Y		Y	Y	Y					Y			Y									Y	Y	Y	Y						Y										
6801	A_23_P211504	KDELR3							Y	Y	Y	Y	Y	Y																Y						Y										
1195	A_23_P58419	KDR			Y	Y	Y	Y		Y	Y	Y															Y	Y	Y	Y						Y							Y			
8774	A_23_P119141	KEAP1				Y														Y		Y																								
17765	A_23_P76275	KERA			Y	Y		Y	Y	Y	Y	Y	Y	Y													Y	Y	Y	Y						Y										
1199	A_23_P369449	KGFLP1																	Y	Y			Y																							
20076	A_23_P200386	KHDRBS1			Y	Y		Y	Y	Y	Y	Y	Y	Y						Y			Y				Y	Y	Y	Y						Y										
13543	A_23_P257335	KHDRBS3							Y	Y										Y																										
8301	A_23_P5845	KHK																Y	Y	Y		Y		Y																						
4430	A_23_P374881	KHSRP			Y																						Y	Y	Y	Y																
14954	A_23_P4741	KHSRP			Y	Y	Y	Y	Y	Y	Y	Y						Y	Y	Y	Y	Y	Y	Y	Y	Y	Y	Y	Y	Y	Y	Y	Y			Y										
9059	A_23_P51327	KIAA0040			Y																																									
15917	A_23_P435610	KIAA0090			Y													Y	Y	Y		Y	Y	Y							Y		Y	Y	Y											
15966	A_23_P207614	KIAA0100			Y																						Y			Y						Y										
18560	A_23_P151577	KIAA0125			Y				Y	Y																																				
7858	A_23_P213369	KIAA0141																					Y																							
9325	A_23_P206756	KIAA0174			Y																																									
13516	A_23_P152415	KIAA0182			Y																	Y																								
16903	A_23_P32643	KIAA0195			Y													Y	Y	Y	Y	Y	Y	Y	Y	Y	Y				Y															
11399	A_23_P167222	KIAA0232							Y	Y																																				
8489	A_23_P371787	KIAA0247			Y	Y		Y	Y	Y	Y	Y	Y	Y				Y	Y		Y	Y					Y			Y						Y										
9582	A_23_P140133	KIAA0247			Y																																									
21171	A_23_P37416	KIAA0256							Y	Y																	Y																			
2416	A_23_P54189	KIAA0317				Y			Y	Y												Y	Y																							
9764	A_23_P168324	KIAA0319				Y																																								
11906	A_23_P394216	KIAA0329			Y																																									
19396	A_23_P67476	KIAA0355			Y	Y	Y	Y	Y	Y	Y	Y						Y	Y	Y		Y	Y	Y			Y	Y	Y	Y						Y						Y				
19628	A_23_P324490	KIAA0355																	Y	Y		Y																								
8116	A_23_P146431	KIAA0368			Y	Y		Y	Y	Y					Y			Y	Y	Y		Y		Y				Y																		
18714	A_23_P134637	KIAA0415			Y	Y	Y	Y	Y	Y	Y	Y	Y	Y					Y	Y		Y	Y				Y	Y	Y	Y						Y										
12647	A_23_P26674	KIAA0430			Y	Y	Y	Y	Y	Y	Y	Y						Y	Y	Y		Y		Y			Y	Y	Y	Y						Y										
2196	A_23_P51639	KIAA0467																Y	Y	Y			Y	Y																						
9661	A_23_P206310	KIAA0513																	Y																											
3540	A_23_P367628	KIAA0528			Y	Y	Y	Y	Y	Y	Y	Y	Y	Y				Y	Y	Y	Y	Y	Y	Y	Y	Y	Y	Y	Y	Y	Y	Y				Y										
11699	A_23_P200043	KIAA0562			Y	Y	Y	Y		Y	Y	Y						Y		Y			Y				Y																			
3669	A_23_P99710	KIAA0586			Y	Y		Y		Y										Y			Y																							
14220	A_23_P146497	KIAA0649	Y		Y	Y		Y	Y	Y	Y	Y	Y	Y				Y									Y			Y						Y										
19214	A_23_P95292	KIAA0652			Y	Y	Y	Y		Y	Y	Y						Y	Y	Y	Y	Y					Y			Y						Y						Y				
13712	A_23_P4435	KIAA0664			Y	Y	Y	Y	Y	Y	Y	Y	Y	Y				Y	Y	Y		Y	Y	Y			Y	Y	Y	Y						Y										
16357	A_23_P89543	KIAA0753	Y	Y	Y	Y	Y	Y	Y	Y	Y	Y	Y	Y	Y			Y	Y	Y		Y	Y	Y			Y	Y	Y	Y	Y	Y	Y	Y	Y	Y										
1277	A_23_P30956	KIAA0776			Y	Y		Y	Y	Y										Y	Y	Y	Y					Y																		
3460	A_23_P309982	KIAA0831			Y	Y	Y	Y	Y	Y	Y	Y	Y	Y				Y	Y	Y	Y	Y	Y	Y	Y	Y	Y	Y	Y	Y	Y	Y	Y	Y	Y	Y										
6531	A_23_P201672	KIAA0859			Y																																									
13109	A_23_P74458	KIAA0907			Y	Y	Y	Y	Y	Y	Y	Y						Y	Y	Y		Y	Y	Y			Y																			

14526	A_23_P257242	KIAA0922															Y																												
14370	A_23_P104833	KIAA0999			Y												Y		Y		Y																								
16587	A_23_P145424	KIAA1009			Y	Y	Y	Y	Y	Y	Y	Y						Y	Y		Y					Y	Y	Y	Y																
10718	A_23_P107552	KIAA1012			Y	Y		Y	Y	Y								Y	Y		Y	Y				Y	Y	Y	Y	Y	Y	Y	Y	Y	Y										
13068	A_23_P147869	KIAA1109				Y												Y									Y		Y						Y										
594	A_23_P404108	KIAA1128				Y												Y																											
21801	A_23_P55376	KIAA1267			Y				Y	Y																Y																			
4437	A_23_P380815	KIAA1279			Y	Y		Y	Y	Y																Y	Y	Y	Y						Y										
10632	A_23_P384499	KIAA1303																Y																											
4273	A_23_P129005	KIAA1305																Y	Y																										
19081	A_23_P115392	KIAA1324							Y	Y																																			
15967	A_23_P320242	KIAA1324L			Y	Y	Y	Y		Y	Y	Y	Y				Y		Y		Y					Y	Y	Y	Y						Y										
6716	A_23_P99853	KIAA1370			Y	Y		Y	Y	Y	Y	Y	Y	Y			Y	Y	Y	Y	Y	Y	Y	Y	Y	Y			Y	Y	Y				Y										
14761	A_23_P413026	KIAA1383																	Y																										
18987	A_23_P419213	KIAA1407	Y	Y	Y	Y	Y	Y	Y	Y	Y	Y	Y	Y			Y	Y	Y	Y	Y	Y	Y	Y	Y	Y	Y	Y	Y	Y	Y	Y	Y	Y	Y			Y							
18628	A_23_P134994	KIAA1432																	Y			Y																							
173	A_23_P356125	KIAA1468			Y	Y		Y		Y								Y	Y	Y	Y	Y																							
10439	A_23_P250313	KIAA1524																								Y																			
2476	A_23_P43380	KIAA1539																				Y																							
15194	A_23_P70827	KIAA1549															Y	Y	Y		Y		Y																						
9788	A_23_P202587	KIAA1598				Y			Y	Y							Y	Y	Y		Y		Y				Y		Y						Y										
12676	A_23_P110243	KIAA1627				Y			Y	Y								Y	Y								Y		Y						Y										
5143	A_23_P130974	KIAA1683																			Y								Y						Y										
9966	A_23_P2732	KIAA1704																	Y		Y	Y																							
6322	A_23_P385911	KIAA1712																								Y																			
15314	A_23_P372925	KIAA1737	Y	Y	Y	Y	Y	Y	Y	Y	Y	Y	Y	Y			Y	Y	Y	Y	Y	Y	Y	Y	Y	Y	Y	Y	Y	Y	Y	Y	Y	Y	Y										
12773	A_23_P119040	KIAA1772	Y	Y	Y	Y		Y		Y							Y	Y	Y		Y		Y			Y																			
13959	A_23_P21673	KIAA1797			Y	Y		Y		Y									Y							Y	Y	Y	Y																
15222	A_23_P96883	KIAA1804			Y																					Y			Y						Y										
1790	A_23_P116166	KIAA1826			Y	Y		Y		Y							Y	Y	Y		Y	Y	Y			Y																			
18100	A_23_P31686	KIAA1967																	Y			Y																							
7250	A_23_P302410	KIAA1984	Y	Y																									Y						Y										
16678	A_23_P352358	KIAA2013			Y	Y	Y	Y	Y	Y	Y	Y	Y	Y			Y	Y	Y	Y	Y	Y	Y	Y	Y	Y	Y	Y	Y	Y	Y				Y										
22249	A_23_P51355	KIAA2013							Y	Y																Y	Y	Y	Y						Y										
22393	A_23_P157758	KIAA2026			Y				Y	Y								Y								Y			Y						Y										
11541	A_23_P214111	KIF13A			Y	Y		Y	Y	Y									Y		Y	Y				Y																			
7291	A_23_P147388	KIF13B	Y	Y	Y	Y	Y	Y	Y	Y	Y	Y	Y	Y			Y		Y			Y				Y			Y						Y										
6325	A_23_P80902	KIF15																				Y																							
13452	A_23_P17503	KIF16B			Y	Y	Y	Y	Y	Y	Y	Y	Y	Y			Y	Y	Y	Y	Y	Y	Y	Y	Y	Y	Y	Y	Y	Y	Y				Y				Y						
9795	A_23_P426270	KIF19	Y	Y	Y	Y		Y	Y	Y							Y	Y	Y			Y	Y			Y	Y	Y	Y						Y										
21819	A_23_P201287	KIF1B				Y												Y	Y																										
2265	A_23_P107369	KIF1C																				Y																							
C13361.9	A_23_P256956	KIF20A															Y		Y																										
1171	A_23_P126888	KIF21B																	Y	Y	Y	Y																							
13183	A_23_P54617	KIF22																								Y																			
3025	A_23_P43595	KIF24			Y	Y	Y	Y	Y	Y	Y	Y						Y	Y		Y	Y				Y	Y	Y	Y																
13167	A_23_P59410	KIF25																								Y																			
1403	A_23_P63541	KIF26B	Y																																										
18295	A_23_P387105	KIF26B																	Y			Y					Y		Y						Y										
18178	A_23_P71741	KIF27			Y	Y	Y	Y	Y	Y	Y	Y					Y	Y	Y		Y	Y	Y			Y	Y	Y	Y														Y		
19023	A_23_P34788	KIF2C			Y												Y		Y							Y																			
13563	A_23_P122027	KIF3A			Y	Y	Y	Y	Y	Y	Y	Y							Y							Y	Y	Y	Y						Y										
5592	A_23_P17558	KIF3B																											Y						Y										
19054	A_23_P148475	KIF4A															Y		Y																										
5976	A_23_P218025	KIF5A			Y																					Y																			
19035	A_23_P212307	KIF9	Y	Y	Y	Y		Y	Y	Y	Y	Y	Y	Y			Y	Y	Y	Y	Y	Y	Y	Y	Y	Y	Y	Y	Y	Y	Y	Y			Y				Y						
2197	A_23_P62920	KIFAP3			Y				Y	Y								Y	Y																										
C9385.5	A_23_P133954	KIFC1															Y		Y		Y																								
20872	A_23_P9106	KIFC2			Y	Y		Y		Y							Y	Y	Y		Y	Y	Y			Y																			
14517	A_23_P54576	KIFC3							Y	Y									Y	Y	Y	Y																							
10538	A_23_P50566	KIR2DL2																											Y						Y										
22274	A_23_P153596	KIR2DL2				Y																				Y																			
1426	A_23_P39174	KIR2DL3	Y	Y	Y	Y	Y	Y	Y	Y	Y	Y	Y	Y			Y	Y								Y			Y						Y										
14513	A_23_P381532	KIR2DS3																								Y																			
16673	A_23_P67437	KIR3DL1			Y																					Y																			
4517	A_23_P370574	KIR3DL3				Y													Y		Y																								
10612	A_23_P113994	KIRREL																											Y						Y										
11654	A_23_P16583	KIRREL2																	Y							Y																			
8101	A_23_P104741	KIRREL3																			Y																								
6778	A_23_P101761	KISS1R				Y													Y			Y																							
278	A_23_P204654	KITLG			Y				Y	Y																Y																			
3865	A_23_P323084	KITLG																								Y																			
16217	A_23_P350617	KLB	Y	Y	Y	Y		Y		Y							Y	Y	Y			Y	Y			Y	Y	Y	Y																
4904	A_23_P2873	KLC1															Y	Y	Y	Y	Y	Y	Y	Y	Y					Y															
12503	A_23_P52690	KLC2																	Y	Y		Y																							
17832	A_23_P4653	KLC3			Y	Y		Y	Y	Y																Y	Y	Y	Y						Y										
6921	A_23_P422766	KLHDC1			Y	Y	Y	Y		Y	Y	Y							Y		Y					Y	Y	Y	Y						Y										

7463	A_23_P54165	KLHDC2			Y				Y	Y													Y				Y	Y	Y	Y						Y										
20163	A_23_P156568	KLHDC3			Y	Y	Y	Y	Y	Y	Y	Y	Y	Y				Y	Y	Y	Y	Y	Y	Y	Y	Y	Y	Y	Y	Y	Y	Y				Y				Y						
11451	A_23_P163800	KLHDC4																	Y	Y	Y	Y																								
4419	A_23_P13673	KLHDC5							Y	Y	Y	Y	Y	Y																Y						Y										
12538	A_23_P6535	KLHDC7B			Y	Y		Y	Y	Y																		Y																		
1460	A_23_P1091	KLHDC8A			Y				Y	Y																	Y																			
9982	A_23_P125117	KLHDC8B			Y	Y	Y	Y		Y	Y	Y						Y	Y	Y	Y	Y	Y	Y	Y	Y					Y															
7445	A_23_P86100	KLHDC9	Y	Y	Y				Y	Y	Y	Y	Y	Y						Y	Y		Y				Y			Y						Y								Y		
17502	A_23_P2825	KLHL1																Y				Y																								
12208	A_23_P55388	KLHL11			Y	Y		Y	Y	Y										Y			Y																							
5438	A_23_P161041	KLHL12			Y																						Y																			
17884	A_23_P72503	KLHL2																		Y										Y						Y										
19714	A_23_P160283	KLHL20			Y	Y		Y		Y									Y	Y								Y																		
22479	A_23_P143580	KLHL22																Y		Y	Y	Y	Y																							
11582	A_23_P165408	KLHL23			Y	Y		Y	Y	Y										Y			Y				Y			Y						Y										
15627	A_23_P251574	KLHL24																									Y																			
15825	A_23_P65802	KLHL25				Y														Y	Y	Y	Y																							
19989	A_23_P39375	KLHL26			Y	Y		Y	Y	Y	Y	Y	Y	Y				Y	Y	Y		Y	Y	Y			Y	Y	Y	Y						Y										
11962	A_23_P205659	KLHL28			Y	Y	Y	Y		Y	Y	Y	Y					Y									Y																	Y		
14325	A_23_P209360	KLHL29			Y																																									
12290	A_23_P133546	KLHL3				Y																																								
2557	A_23_P252521	KLHL32																												Y						Y										
15431	A_23_P428887	KLHL34																		Y																										
18975	A_23_P351834	KLHL35																		Y																										
6476	A_23_P88802	KLHL36			Y	Y	Y	Y	Y	Y	Y	Y	Y	Y				Y	Y	Y	Y	Y	Y	Y	Y	Y	Y	Y	Y	Y	Y	Y				Y										
7744	A_23_P121527	KLHL5																Y	Y	Y		Y	Y	Y						Y	Y	Y	Y	Y	Y	Y										
8314	A_23_P215517	KLHL7			Y	Y		Y	Y	Y	Y	Y	Y	Y														Y		Y						Y										
1589	A_23_P83159	KLHL9			Y	Y	Y	Y	Y	Y	Y	Y	Y	Y				Y	Y	Y	Y	Y	Y	Y	Y	Y	Y			Y	Y	Y				Y										
4404	A_23_P16254	KLK1				Y													Y	Y		Y						Y																		
9482	A_23_P107911	KLK10			Y				Y	Y									Y											Y						Y										
9498	A_23_P101500	KLK11																		Y		Y																								
4304	A_23_P500010	KLK12																			Y																									
2754	A_23_P78717	KLK14				Y			Y	Y																				Y						Y										
2736	A_23_P4856	KLK15																					Y																							
14635	A_23_P107924	KLK2																	Y																											
22469	A_23_P208669	KLK3			Y																						Y																			
17967	A_23_P153475	KLK5							Y	Y								Y									Y			Y						Y										
8991	A_23_P142090	KLK6				Y			Y	Y																	Y			Y						Y										
359	A_23_P39056	KLK7			Y	Y		Y	Y	Y																																				
8992	A_23_P369343	KLK8							Y	Y																				Y						Y										
17478	A_23_P130694	KLK8							Y	Y																				Y						Y										
12433	A_23_P119311	KLK9				Y														Y										Y						Y										
17241	A_23_P88771	KLKBL4																	Y											Y						Y										
13526	A_23_P407662	KLRA1				Y																																								
5008	A_23_P90895	KLRAQ1																	Y																											
16370	A_23_P99275	KLRB1	Y		Y				Y	Y					Y				Y								Y																			
14441	A_23_P22232	KLRC2																Y			Y																									
3429	A_23_P218058	KLRC4																Y																												
13913	A_23_P204208	KLRD1	Y	Y	Y																						Y																			
9252	A_23_P64898	KLRG1							Y	Y									Y																											
11050	A_23_P8723	KLRG2																Y	Y																											
15179	A_23_P200838	KMO				Y			Y	Y																				Y						Y										
C10614.1	A_23_P136817	KNTC1			Y	Y		Y		Y										Y																										
11795	A_23_P61949	KPNA1			Y															Y			Y																							
16365	A_23_P351063	KPNA1																					Y																							
6798	A_23_P125265	KPNA2	Y	Y																Y																										
11193	A_23_P218835	KPNA4			Y																																									
1309	A_23_P156443	KPNA5																		Y																										
13822	A_23_P45889	KPNA6				Y																						Y																		
15474	A_23_P251128	KPNA6																		Y	Y	Y					Y			Y						Y										
14102	A_23_P67387	KPTN							Y	Y																				Y						Y										
7829	A_23_P45140	KRAS																										Y																		
13352	A_23_P56759	KRCC1																		Y	Y	Y																								
11196	A_23_P68851	KREMEN1			Y																		Y																							
15181	A_23_P211407	KREMEN1	Y	Y	Y														Y								Y																			
8001	A_23_P77612	KREMEN2			Y	Y		Y		Y								Y	Y	Y			Y	Y				Y																		
18978	A_23_P42848	KRIT1				Y	Y		Y	Y	Y	Y								Y			Y					Y																		
20845	A_23_P2211	KRR1				Y													Y	Y								Y																		
344	A_23_P49928	KRT13				Y			Y	Y																		Y																		
586	A_23_P27133	KRT15				Y																																								
834	A_23_P38537	KRT16				Y			Y	Y	Y	Y	Y	Y				Y		Y		Y								Y						Y										
6269	A_23_P96149	KRT17																												Y						Y										
19715	A_23_P99324	KRT18			Y	Y		Y	Y	Y	Y	Y	Y	Y																Y						Y										
10017	A_23_P122650	KRT18P30			Y	Y		Y	Y	Y	Y	Y	Y	Y																Y						Y										
18265	A_23_P66798	KRT19			Y	Y		Y	Y	Y	Y	Y	Y	Y				Y		Y		Y	Y				Y			Y	Y	Y	Y	Y	Y	Y										
10787	A_23_P425925	KRT222P			Y	Y		Y	Y	Y	Y	Y	Y	Y				Y	Y	Y		Y		Y			Y	Y	Y	Y						Y										
20437	A_23_P78248	KRT23							Y	Y													Y																							

12442	A_23_P15786	KRT25																									Y	Y	Y	Y																
19831	A_23_P27186	KRT26							Y	Y																																				
13882	A_23_P107465	KRT31	Y	Y		Y																	Y					Y																		
17271	A_23_P89601	KRT32			Y	Y		Y		Y								Y		Y	Y	Y						Y																		
1821	A_23_P78265	KRT33A																			Y																									
19417	A_23_P89665	KRT33B																												Y						Y										
13554	A_23_P78201	KRT35																									Y																			
21222	A_23_P55400	KRT36									Y		Y	Y													Y																			
19051	A_23_P118854	KRT37	Y	Y	Y	Y		Y	Y	Y										Y			Y				Y	Y	Y	Y						Y										
5398	A_23_P2671	KRT4			Y																																									
1526	A_23_P218040	KRT5			Y	Y		Y		Y									Y									Y																		
22288	A_23_P87653	KRT6A			Y				Y	Y	Y	Y	Y	Y						Y							Y			Y						Y										
14988	A_23_P76249	KRT6B			Y				Y	Y																	Y			Y						Y										
4117	A_23_P366939	KRT6C			Y				Y	Y	Y	Y	Y	Y						Y										Y						Y										
21228	A_23_P313591	KRT7			Y	Y	Y	Y	Y	Y	Y	Y							Y	Y							Y	Y	Y	Y						Y										
21404	A_23_P381945	KRT7			Y	Y	Y	Y	Y	Y	Y	Y						Y	Y	Y				Y			Y	Y	Y	Y						Y										
3315	A_23_P64854	KRT75	Y	Y	Y													Y		Y		Y					Y																			
18586	A_23_P61498	KRT76			Y																																									
3406	A_23_P14072	KRT8							Y	Y								Y	Y	Y				Y																						
10258	A_23_P25187	KRT81	Y	Y	Y	Y	Y	Y	Y	Y	Y	Y	Y	Y	Y			Y	Y	Y			Y	Y			Y	Y	Y	Y	Y	Y	Y	Y	Y											
21143	A_23_P53450	KRT82			Y																																									
18882	A_23_P204144	KRT85							Y	Y																				Y						Y										
7739	A_23_P363769	KRT86			Y	Y		Y		Y									Y								Y																			
22498	A_23_P139619	KRT86			Y	Y		Y	Y	Y								Y	Y	Y			Y	Y			Y	Y	Y	Y						Y										
18176	A_23_P15734	KRT9			Y	Y	Y	Y	Y	Y	Y	Y	Y	Y				Y	Y	Y		Y	Y	Y			Y	Y	Y	Y						Y										
5376	A_23_P66858	KRTAP17-1																					Y																							
10102	A_23_P153022	KRTAP2-4			Y	Y		Y	Y	Y								Y	Y	Y		Y	Y	Y			Y			Y						Y										
11976	A_23_P101048	KRTAP3-2				Y																																								
9976	A_23_P49988	KRTAP4-2			Y																	Y					Y																			
7255	A_23_P47093	KRTAP5-8																		Y			Y				Y	Y	Y	Y																
2521	A_23_P13048	KRTAP5-9			Y	Y	Y	Y	Y	Y	Y	Y							Y	Y	Y	Y	Y				Y	Y	Y	Y						Y										
11360	A_23_P312932	KRTAP8-1			Y																						Y			Y						Y										
16700	A_23_P89646	KRTAP9-2				Y																																								
9463	A_23_P101038	KRTAP9-3																		Y																										
19340	A_23_P149249	KRTCAP2				Y																					Y																			
10496	A_23_P90456	KRTDAP																		Y	Y		Y																							
11155	A_23_P84711	KSR2																									Y																			
6439	A_23_P135895	KTELC1							Y	Y										Y		Y																								
15234	A_23_P103273	KTI12																	Y											Y						Y										
9324	A_23_P106131	KTN1			Y	Y		Y	Y	Y			Y						Y	Y		Y					Y	Y	Y	Y						Y										
943	A_23_P399897	KY																			Y		Y																							
793	A_23_P56898	KYNU			Y				Y	Y	Y	Y	Y	Y													Y			Y						Y										
11580	A_23_P137484	L1TD1			Y	Y		Y	Y	Y								Y									Y	Y	Y	Y						Y										
17817	A_23_P356749	L1TD1	Y		Y	Y		Y		Y																	Y	Y	Y	Y						Y										
6375	A_23_P210445	L3MBTL			Y	Y		Y		Y					Y			Y		Y		Y	Y				Y	Y	Y	Y			Y													
9353	A_23_P80353	L3MBTL2			Y	Y	Y	Y	Y	Y	Y	Y	Y	Y				Y	Y	Y			Y	Y			Y			Y						Y										
17158	A_23_P327415	LACE1																			Y																									
10789	A_23_P64825	LACRT							Y	Y	Y	Y	Y	Y																																
15208	A_23_P252711	LACTB2																					Y																							
3024	A_23_P46090	LAD1			Y	Y		Y	Y	Y									Y	Y			Y				Y			Y						Y										
16569	A_23_P415510	LAD1	Y	Y	Y	Y		Y	Y	Y										Y							Y	Y	Y	Y						Y										
13069	A_23_P116942	LAG3	Y	Y	Y	Y	Y	Y	Y	Y	Y	Y	Y	Y				Y	Y	Y				Y			Y	Y	Y	Y						Y										
9944	A_23_P73763	LAGE3				Y												Y	Y	Y		Y		Y																						
16773	A_23_P209135	LAIR1	Y		Y	Y		Y	Y	Y					Y					Y	Y																									
20914	A_23_P209129	LAIR2	Y	Y	Y	Y		Y	Y	Y										Y							Y	Y	Y	Y																
1716	A_23_P116765	LALBA			Y	Y		Y	Y	Y								Y	Y	Y		Y	Y	Y			Y	Y	Y	Y						Y										
6108	A_23_P118967	LAMA1																									Y																			
4305	A_23_P70719	LAMA2							Y	Y																		Y																		
22034	A_23_P89780	LAMA3	Y	Y	Y	Y		Y		Y	Y	Y	Y	Y				Y	Y	Y				Y			Y																			
21118	A_23_P109269	LAMA5																Y	Y	Y	Y	Y	Y	Y	Y	Y					Y															
16809	A_23_P94030	LAMB1							Y	Y																	Y																			
12152	A_23_P21382	LAMB2	Y	Y	Y	Y		Y	Y	Y								Y	Y	Y		Y		Y			Y	Y	Y	Y						Y										
4218	A_23_P86012	LAMB3																												Y						Y										
3202	A_23_P201628	LAMC1																	Y																											
19164	A_23_P201636	LAMC2																Y	Y	Y				Y																						
20191	A_23_P217446	LAMP2			Y																						Y																			
10714	A_23_P29773	LAMP3	Y	Y																							Y																			
12333	A_23_P50887	LANCL1																					Y																							
10435	A_23_P82641	LANCL2			Y																																									
3314	A_23_P90659	LAPTM4A			Y															Y							Y	Y	Y	Y																
16769	A_23_P59926	LAPTM4B			Y	Y		Y	Y	Y																		Y																		
13274	A_23_P86283	LAPTM5	Y		Y	Y	Y	Y	Y	Y	Y	Y			Y				Y	Y	Y		Y				Y	Y	Y	Y																
17307	A_23_P91697	LARGE			Y																																									
13137	A_23_P392378	LARP1			Y													Y	Y	Y	Y	Y	Y	Y	Y	Y	Y				Y															
16216	A_23_P353744	LARP2																		Y		Y	Y																							
16874	A_23_P7253	LARP2							Y	Y	Y	Y	Y	Y						Y	Y	Y	Y								Y		Y	Y	Y											
17968	A_23_P7353	LARP2																Y	Y	Y		Y	Y	Y				Y																		

13951	A_23_P35677	LARP5																		Y			Y																							
15397	A_23_P117782	LARP6				Y			Y	Y																Y		Y				Y										
5582	A_23_P84070	LARP7			Y	Y		Y		Y										Y																								
5601	A_23_P113094	LARP7				Y														Y																								
9080	A_23_P218967	LARS			Y	Y	Y	Y	Y	Y	Y	Y								Y																								
8106	A_23_P89185	LASP1																		Y																										
12658	A_23_P77151	LASS3			Y																																							
22160	A_23_P153867	LASS4			Y	Y		Y		Y						Y		Y																								
14642	A_23_P76515	LASS5																			Y																									
11266	A_23_P214919	LATS1			Y																																							
9685	A_23_P433	LAX1	Y	Y	Y				Y	Y										Y							Y																			
3220	A_23_P427788	LAYN				Y																																						
8149	A_23_P127565	LAYN				Y																																						
4581	A_23_P120227	LBH			Y				Y	Y										Y			Y				Y																			
2951	A_23_P200493	LBR	Y	Y					Y	Y								Y	Y			Y							Y						Y										
4306	A_23_P86493	LBX1			Y	Y		Y	Y	Y								Y	Y		Y	Y				Y																			
6455	A_23_P79458	LBX2																		Y																										
2404	A_23_P312893	LCA5L							Y	Y																			Y						Y										
22539	A_23_P218230	LCAT			Y																					Y																				
4249	A_23_P404685	LCE1A			Y													Y		Y	Y	Y	Y				Y																			
1913	A_23_P368862	LCE1B							Y	Y	Y	Y	Y	Y					Y	Y								Y	Y	Y	Y						Y										
22135	A_23_P303891	LCE1C			Y	Y		Y	Y	Y										Y	Y	Y	Y				Y			Y						Y										
7632	A_23_P375524	LCE1D			Y	Y		Y		Y										Y	Y	Y	Y																							
824	A_23_P411321	LCE1F				Y																Y																							
21952	A_23_P74926	LCE2B			Y																																							
21746	A_23_P371284	LCE2D			Y															Y		Y																								
16590	A_23_P405295	LCE3C																	Y	Y						Y																				
2468	A_23_P340340	LCE3E			Y		Y		Y	Y	Y	Y	Y	Y				Y	Y	Y			Y	Y			Y			Y						Y										
6504	A_23_P104124	LCE3E							Y	Y																																		
20978	A_23_P304530	LCE5A			Y	Y		Y	Y	Y								Y	Y		Y	Y				Y	Y	Y	Y																	
7387	A_23_P103361	LCK	Y		Y	Y	Y	Y	Y	Y	Y	Y							Y	Y			Y				Y																			
7045	A_23_P33148	LCMT1			Y	Y		Y	Y	Y	Y	Y	Y	Y																															
13868	A_23_P112825	LCMT1			Y	Y		Y	Y	Y	Y	Y	Y	Y																															
15127	A_23_P124213	LCMT1			Y	Y		Y	Y	Y	Y	Y	Y	Y																															
3039	A_23_P106505	LCMT2			Y	Y		Y	Y	Y	Y	Y	Y	Y						Y		Y	Y				Y			Y						Y										
19652	A_23_P32122	LCN12			Y																																							
12033	A_23_P169437	LCN2			Y	Y		Y		Y	Y	Y	Y	Y																	Y		Y				Y										
14856	A_23_P83127	LCN9																											Y																		
18039	A_23_P30547	LCP2	Y	Y	Y											Y										Y																				
1451	A_23_P79213	LCT			Y				Y	Y								Y							Y	Y	Y	Y						Y										
6547	A_23_P88613	LCTL																		Y			Y																							
7966	A_23_P47565	LDHA																	Y																											
22392	A_23_P303789	LDHAL6A				Y												Y													Y						Y										
1141	A_23_P54362	LDHAL6B			Y																									Y						Y										
20259	A_23_P53476	LDHB				Y			Y	Y	Y	Y	Y	Y														Y		Y				Y										
14715	A_23_P53039	LDHC	Y	Y	Y		Y				Y		Y	Y					Y	Y						Y	Y	Y	Y						Y										
4476	A_23_P251906	LDLRAD3																Y											Y						Y										
20573	A_23_P114105	LDOC1			Y													Y		Y																								
7868	A_23_P143755	LDOC1L																													Y						Y										
17356	A_23_P132365	LDOC1L			Y				Y	Y													Y							Y						Y										
22419	A_23_P92831	LEAP2			Y	Y		Y	Y	Y								Y		Y		Y					Y	Y	Y	Y						Y										
15307	A_23_P25587	LECT1																		Y																										
5230	A_23_P110777	LECT2																													Y						Y										
18672	A_23_P213045	LEF1	Y	Y	Y														Y	Y						Y			Y						Y										
16925	A_23_P51307	LELP1			Y															Y		Y					Y																				
2852	A_23_P23123	LENEP			Y																					Y																				
1148	A_23_P314222	LEO1			Y	Y		Y	Y	Y	Y	Y	Y	Y					Y	Y		Y					Y	Y	Y	Y						Y										
8425	A_23_P31426	LEP																	Y	Y			Y																							
14483	A_23_P161135	LEPR				Y																																						
6351	A_23_P69179	LEPREL1							Y	Y																			Y						Y										
6731	A_23_P87752	LEPREL2																		Y																										
16582	A_23_P1126	LEPROT				Y														Y																								
15112	A_23_P348264	LETM2			Y	Y		Y		Y										Y							Y																				
20526	A_23_P117037	LETMD1	Y	Y					Y	Y								Y																											
8736	A_23_P8452	LFNG																									Y																				
1843	A_23_P166459	LGALS1																Y		Y			Y																							
1225	A_23_P5035	LGALS13																	Y		Y																									
2480	A_23_P120902	LGALS2			Y																																							
15175	A_23_P15353	LGALS3BP																		Y			Y																							
20286	A_23_P254917	LGALS4			Y													Y	Y				Y				Y			Y						Y										
17237	A_23_P63017	LGALS8							Y	Y																																		
8608	A_23_P116557	LGALS9			Y				Y	Y								Y	Y						Y																				
3082	A_23_P101025	LGALS9C			Y				Y	Y								Y	Y		Y				Y																				
8224	A_23_P7191	LGI2	Y												Y			Y		Y																								
14562	A_23_P21086	LGI3			Y															Y						Y																				
10647	A_23_P397485	LGI4																	Y																											
16020	A_23_P209070	LGI4																									Y																				

17472	A_23_P25994	LGMN	Y		Y	Y	Y	Y	Y	Y	Y	Y	Y	Y	Y			Y	Y	Y	Y	Y	Y	Y	Y	Y	Y				Y		Y	Y	Y												
16695	A_23_P98979	LGR5															Y	Y	Y				Y																						
19158	A_23_P23837	LGR6							Y	Y									Y																												
19914	A_23_P218505	LHB			Y	Y	Y	Y	Y	Y	Y	Y							Y	Y		Y	Y				Y			Y							Y											
6614	A_23_P88069	LHFP			Y																									Y						Y											
7738	A_23_P255104	LHFPL2	Y	Y																																										
13148	A_23_P75299	LHPP				Y																																								
21260	A_23_P43964	LHPP				Y																																								
18134	A_23_P9496	LHX3			Y																																									
5745	A_23_P32175	LHX6																Y	Y	Y		Y		Y			Y	Y	Y	Y																	
2301	A_23_P254081	LIAS				Y			Y	Y	Y	Y	Y	Y				Y					Y					Y		Y						Y									
1737	A_23_P155017	LIF																					Y																						
13843	A_23_P133517	LIFR				Y																																								
10769	A_23_P207272	LIG3			Y																																									
5880	A_23_P25579	LIG4			Y	Y		Y		Y									Y	Y	Y		Y	Y	Y			Y																		
4057	A_23_P164982	LILRA1			Y													Y		Y	Y	Y	Y																						
7975	A_23_P142205	LILRA2	Y	Y	Y	Y	Y	Y	Y	Y	Y	Y								Y							Y	Y	Y	Y																	
14487	A_23_P79094	LILRA3	Y	Y	Y	Y		Y		Y									Y	Y	Y		Y	Y	Y																					
1242	A_23_P90497	LILRA4	Y		Y	Y	Y	Y	Y	Y	Y	Y						Y	Y	Y		Y	Y	Y			Y	Y	Y	Y						Y									
3972	A_23_P4977	LILRB1	Y		Y	Y	Y	Y	Y	Y	Y	Y						Y	Y	Y	Y	Y	Y	Y	Y	Y	Y	Y	Y	Y	Y	Y														
17883	A_23_P208493	LILRB2	Y	Y	Y				Y	Y					Y												Y	Y	Y	Y						Y									
6613	A_23_P208500	LILRB3	Y	Y	Y	Y	Y	Y	Y	Y	Y	Y			Y				Y	Y			Y				Y	Y	Y	Y						Y									
21591	A_23_P16381	LILRB4			Y	Y	Y	Y	Y	Y	Y	Y								Y							Y																		
22500	A_23_P164784	LILRB5			Y				Y	Y	Y	Y	Y	Y						Y	Y	Y	Y							Y			Y			Y									
18822	A_23_P27781	LILRP2	Y	Y	Y	Y	Y	Y		Y	Y	Y						Y	Y	Y		Y	Y	Y			Y			Y							Y									
20440	A_23_P130435	LIM2				Y												Y	Y			Y					Y	Y	Y	Y						Y									
17985	A_23_P151267	LIMA1			Y	Y		Y	Y	Y	Y	Y	Y	Y					Y	Y		Y					Y	Y	Y	Y						Y									
1417	A_23_P166858	LIMD1																Y		Y		Y																							
13406	A_23_P434965	LIMD1																		Y																									
15393	A_23_P26823	LIMD2	Y	Y	Y	Y		Y	Y	Y									Y	Y	Y				Y			Y	Y	Y	Y																	
11134	A_23_P376088	LIME1																	Y	Y		Y	Y																						
8360	A_23_P211445	LIMK2				Y																																								
9043	A_23_P210358	LIMS1			Y																																									
17702	A_23_P142795	LIMS2																		Y			Y																						
250	A_23_P74892	LIN28	Y	Y	Y						Y		Y	Y													Y			Y						Y									
21487	A_23_P165186	LIN37			Y	Y		Y		Y																																				
20017	A_23_P99251	LIN7A							Y	Y																																				
1830	A_23_P164912	LIN7B							Y	Y																																				
1265	A_23_P301995	LIN9																												Y						Y									
12617	A_23_P206092	LINGO1			Y																					Y																		
144	A_23_P205946	LINS1			Y	Y		Y		Y																	Y																		
9553	A_23_P88561	LIPC																Y	Y								Y			Y						Y									
8368	A_23_P38876	LIPE			Y																																									
6464	A_23_P78405	LIPG																									Y			Y						Y									
6513	A_23_P84219	LIPH							Y	Y																																				
16915	A_23_P35624	LIPK																	Y				Y																						
11095	A_23_P501805	LIPT1																		Y			Y																						
4798	A_23_P3532	LITAF				Y													Y																									
8997	A_23_P341488	LIX1													Y					Y																									
20388	A_23_P342744	LIX1L			Y				Y	Y	Y	Y	Y	Y				Y	Y	Y	Y	Y	Y	Y	Y	Y	Y				Y															
16282	A_23_P130266	LLGL1																									Y																		
17796	A_23_P129740	LLGL2			Y	Y		Y	Y	Y									Y	Y	Y		Y	Y	Y			Y			Y						Y									
13062	A_23_P78344	LMAN1	Y	Y	Y	Y		Y		Y																		Y	Y	Y	Y																	
16514	A_23_P140630	LMAN1L			Y																		Y				Y																		
10125	A_23_P169599	LMAN2			Y				Y	Y									Y	Y		Y					Y																		
21501	A_23_P142708	LMAN2L			Y															Y						Y																		
15459	A_23_P215214	LMBR1							Y	Y										Y	Y									Y						Y									
9877	A_23_P150931	LMBR1L																		Y																									
14044	A_23_P6771	LMCD1							Y	Y																																				
4751	A_23_P88939	LMF1							Y	Y																																				
9977	A_23_P43786	LMLN			Y	Y		Y	Y	Y	Y	Y	Y	Y			Y	Y	Y	Y	Y	Y	Y	Y	Y	Y	Y	Y	Y	Y	Y	Y	Y	Y	Y				Y						
2821	A_23_P34835	LMNA																		Y																									
1177	A_23_P67723	LMNB2			Y	Y		Y	Y	Y									Y	Y	Y		Y	Y	Y			Y	Y	Y	Y						Y									
6472	A_23_P87310	LMO1																		Y																									
4153	A_23_P204317	LMO3			Y																																									
17053	A_23_P380181	LMO4			Y	Y		Y	Y	Y	Y	Y	Y	Y			Y	Y	Y		Y	Y	Y																					
9558	A_23_P205159	LMO7																	Y				Y																						
4558	A_23_P410746	LMTK2			Y				Y	Y							Y	Y	Y			Y	Y																					
5399	A_23_P164918	LMTK3							Y	Y																			Y						Y									
2624	A_23_P156063	LNPEP																												Y						Y									
21975	A_23_P213139	LNX1			Y				Y	Y									Y		Y																							
19424	A_23_P402287	LNX2			Y	Y		Y	Y	Y	Y	Y	Y	Y							Y		Y							Y						Y									
1120	A_23_P256903	LOC100128416			Y															Y			Y																						
17396	A_23_P112935	LOC100128641				Y																							Y																	
7122	A_23_P31013	LOC100129322			Y																																									
9039	A_23_P359174	LOC100130401	Y	Y	Y	Y	Y	Y	Y	Y	Y	Y															Y	Y	Y	Y																	
22438	A_23_P81484	LOC100130703																	Y									Y		Y						Y									

20836	A_23_P75348	LOC100130729			Y	Y	Y	Y	Y	Y	Y	Y																Y	Y	Y	Y						Y										
6203	A_23_P257497	LOC100130967							Y	Y																			Y																		
11062	A_23_P77724	LOC100131601																		Y			Y																						
16740	A_23_P4931	LOC100131878			Y	Y	Y	Y		Y	Y	Y						Y	Y	Y		Y	Y	Y			Y																		
16583	A_23_P426454	LOC100132288																Y																											
12670	A_23_P310552	LOC100133036																									Y	Y	Y	Y															
842	A_23_P70813	LOC100133056			Y	Y		Y	Y	Y								Y										Y		Y						Y									
550	A_23_P119242	LOC100133142																		Y			Y																						
1703	A_23_P259767	LOC100133739	Y	Y	Y	Y	Y	Y	Y	Y	Y	Y	Y	Y				Y	Y	Y				Y			Y	Y	Y	Y						Y									
17775	A_23_P158112	LOC100133920																									Y																		
12714	A_23_P116173	LOC120376							Y	Y																				Y						Y									
4918	A_23_P118203	LOC124220	Y	Y	Y				Y	Y	Y	Y	Y	Y							Y		Y				Y			Y						Y									
7754	A_23_P255897	LOC129293																		Y			Y																						
20118	A_23_P109676	LOC131691			Y																						Y																		
18486	A_23_P93960	LOC136242			Y																																								
13499	A_23_P98960	LOC144438			Y	Y	Y	Y	Y	Y	Y	Y	Y	Y				Y	Y	Y	Y	Y	Y	Y	Y	Y	Y	Y	Y	Y	Y	Y	Y			Y									
18152	A_23_P54793	LOC146429			Y	Y		Y	Y	Y																	Y																		
4030	A_23_P101246	LOC147645			Y																																								
9752	A_23_P55854	LOC147804			Y														Y	Y																									
19159	A_23_P131176	LOC151146																Y	Y	Y		Y	Y	Y																					
13600	A_23_P424051	LOC153328																				Y																							
8983	A_23_P320159	LOC170082																	Y																										
2267	A_23_P27692	LOC199800			Y				Y	Y													Y				Y			Y						Y									
4805	A_23_P421512	LOC220594			Y	Y		Y	Y	Y																	Y	Y	Y	Y						Y									
16142	A_23_P253181	LOC221442																	Y											Y						Y									
16735	A_23_P70683	LOC221710							Y	Y																																			
11004	A_23_P339744	LOC255783			Y	Y		Y	Y	Y									Y	Y		Y	Y				Y																		
12458	A_23_P131255	LOC26010	Y	Y	Y													Y																											
843	A_23_P334795	LOC283588				Y																					Y	Y	Y	Y															
16410	A_23_P435672	LOC338799																												Y						Y									
13082	A_23_P136724	LOC344887																									Y			Y						Y									
19854	A_23_P129103	LOC388152											Y							Y			Y																						
8115	A_23_P41267	LOC401127			Y		Y				Y																Y																		
1286	A_23_P150198	LOC440040			Y				Y	Y													Y				Y			Y						Y									
15956	A_23_P147365	LOC440258			Y	Y		Y	Y	Y	Y	Y	Y	Y						Y							Y	Y	Y	Y						Y									
18718	A_23_P43548	LOC442421			Y	Y	Y	Y	Y	Y	Y	Y							Y	Y		Y					Y	Y	Y	Y															
13990	A_23_P206501	LOC497190																		Y																									
4064	A_23_P90565	LOC51057																									Y			Y						Y									
2724	A_23_P149441	LOC51152			Y																						Y	Y	Y	Y						Y									
16427	A_23_P103269	LOC51336			Y				Y	Y																	Y																		
6548	A_23_P142239	LOC541469			Y																						Y																		
2244	A_23_P91779	LOC553158							Y	Y								Y												Y						Y									
12868	A_23_P130677	LOC55908	Y	Y	Y		Y				Y																Y																		
11439	A_23_P64837	LOC57228			Y	Y		Y	Y	Y									Y	Y			Y				Y	Y	Y	Y						Y									
10459	A_23_P81327	LOC57399			Y	Y	Y	Y		Y	Y	Y															Y			Y						Y									
1760	A_23_P259103	LOC63920			Y	Y	Y	Y	Y	Y	Y	Y	Y	Y					Y	Y		Y	Y				Y	Y	Y	Y						Y									
2282	A_23_P376449	LOC642413	Y	Y	Y	Y		Y		Y								Y										Y		Y						Y									
9013	A_23_P59828	LOC644236			Y	Y		Y	Y	Y																	Y																		
20840	A_23_P164324	LOC644297			Y	Y		Y		Y																		Y		Y						Y									
6091	A_23_P63953	LOC646119			Y														Y	Y							Y																		
2905	A_23_P72708	LOC646627																									Y																		
11346	A_23_P67737	LOC653284	Y	Y	Y	Y		Y	Y	Y	Y	Y	Y	Y				Y	Y	Y				Y			Y	Y	Y	Y						Y								Y		
2533	A_23_P258108	LOC727835			Y															Y							Y																		
4287	A_23_P22800	LOC728137			Y													Y									Y			Y						Y									
9705	A_23_P34200	LOC728137	Y																											Y						Y									
8921	A_23_P3694	LOC728276																												Y						Y									
17110	A_23_P27761	LOC728485																									Y																		
1281	A_23_P17752	LOC729314			Y	Y		Y	Y	Y																	Y	Y	Y	Y						Y									
11064	A_23_P56032	LOC729974																		Y																									
14015	A_23_P46447	LOC730102																Y	Y	Y	Y	Y	Y	Y	Y	Y					Y														
16015	A_23_P46673	LOC730167																					Y																						
8844	A_23_P254441	LOC791120	Y	Y	Y	Y	Y	Y	Y	Y	Y	Y	Y	Y				Y		Y		Y	Y				Y	Y	Y	Y						Y									
200	A_23_P4060	LOC79999			Y	Y		Y		Y									Y								Y	Y	Y	Y						Y									
5317	A_23_P50399	LOC90379			Y	Y	Y	Y		Y	Y	Y						Y	Y	Y		Y		Y			Y			Y						Y						Y				
11029	A_23_P41541	LOC90826			Y														Y	Y		Y	Y				Y																		
5809	A_23_P143600	LOC91316							Y	Y																																			
13906	A_23_P213166	LOC91431			Y	Y		Y		Y									Y	Y		Y																							
322	A_23_P257593	LOH12CR1																					Y																						
7822	A_23_P334103	LOH12CR1																					Y																						
20356	A_23_P166777	LOH3CR2A																				Y																							
6514	A_23_P208469	LONP1			Y				Y	Y										Y		Y					Y																		
18503	A_23_P118185	LONP2																					Y																						
7393	A_23_P94216	LONRF1			Y													Y	Y	Y				Y																					
14204	A_23_P114414	LONRF3																					Y																						
4639	A_23_P22297	LOR			Y																						Y																		
C12523.4	A_23_P122216	LOX							Y	Y																																			
4197	A_23_P124084	LOXL1																		Y			Y																						

C12868.4	A_23_P111995	LOXL2				Y			Y	Y																																				
17641	A_23_P39795	LOXL3			Y	Y		Y	Y	Y	Y	Y	Y	Y																																
17255	A_23_P12755	LOXL4																		Y									Y																	
12616	A_23_P95221	LPA			Y	Y	Y	Y	Y	Y	Y	Y	Y	Y						Y		Y					Y			Y						Y								Y		
15214	A_23_P502879	LPAR1				Y			Y	Y									Y	Y										Y						Y										
18963	A_23_P216609	LPAR1			Y	Y	Y	Y	Y	Y	Y	Y						Y	Y	Y		Y		Y			Y			Y						Y										
17264	A_23_P101829	LPAR2			Y	Y		Y		Y								Y		Y		Y					Y																			
637	A_23_P35035	LPAR3																												Y						Y										
6019	A_23_P436048	LPAR3																									Y																			
4103	A_23_P213471	LPCAT1			Y		Y		Y	Y	Y	Y						Y	Y	Y		Y	Y	Y			Y																			
11854	A_23_P141021	LPCAT2																	Y																											
17728	A_23_P49008	LPCAT4			Y	Y	Y	Y	Y	Y	Y	Y	Y	Y				Y	Y	Y	Y	Y	Y	Y	Y	Y	Y	Y	Y	Y	Y	Y	Y	Y	Y	Y										
2633	A_23_P208551	LPHN1																												Y						Y										
10136	A_23_P74042	LPHN2																Y																												
21938	A_23_P382718	LPIN1																Y		Y										Y						Y										
21860	A_23_P100583	LPO																										Y																		
10471	A_23_P251118	LPP																									Y																			
20242	A_23_P87150	LPXN	Y		Y	Y		Y	Y	Y													Y				Y			Y						Y										
14314	A_23_P167179	LRAT																				Y	Y																							
8138	A_23_P251992	LRBA			Y	Y	Y	Y	Y	Y	Y	Y	Y	Y				Y	Y	Y	Y	Y	Y	Y	Y	Y	Y	Y	Y	Y	Y	Y				Y										
8782	A_23_P73809	LRCH2																Y																												
2235	A_23_P355364	LRCH3							Y	Y																				Y						Y										
2994	A_23_P256993	LRCH3			Y																		Y																							
22076	A_23_P95862	LRDD																				Y																								
15224	A_23_P81795	LRFN2							Y	Y																				Y						Y										
4244	A_23_P63980	LRFN4																Y		Y		Y	Y																							
6011	A_23_P50638	LRG1			Y				Y	Y	Y	Y	Y	Y				Y			Y																									
3626	A_23_P357216	LRGUK	Y	Y	Y													Y									Y			Y						Y										
13383	A_23_P109636	LRIG1	Y	Y	Y	Y	Y	Y	Y	Y	Y	Y	Y	Y				Y	Y	Y	Y	Y	Y	Y	Y	Y	Y	Y	Y	Y	Y	Y	Y	Y	Y	Y				Y						
19805	A_23_P160869	LRIG2			Y				Y	Y								Y	Y	Y		Y	Y	Y																						
22545	A_23_P47885	LRIG3																	Y																											
15472	A_23_P115780	LRIT1							Y	Y																				Y						Y										
8496	A_23_P124837	LRP1																		Y	Y	Y	Y																							
11238	A_23_P147623	LRP1																		Y			Y																							
1340	A_23_P19652	LRP11							Y	Y																																				
19003	A_23_P8906	LRP12							Y	Y																																				
15000	A_23_P5342	LRP1B			Y	Y	Y	Y		Y	Y	Y								Y		Y					Y	Y	Y	Y																
2058	A_23_P28295	LRP2			Y	Y		Y	Y	Y	Y	Y	Y	Y				Y												Y			Y			Y										
19101	A_23_P30035	LRP2BP			Y	Y		Y	Y	Y								Y	Y	Y		Y		Y			Y	Y	Y	Y																
20484	A_23_P159349	LRP4			Y													Y																												
807	A_23_P1505	LRP5																	Y								Y																			
9399	A_23_P392457	LRP6							Y	Y																																				
19849	A_23_P150827	LRP6			Y				Y	Y	Y	Y	Y	Y													Y			Y						Y						Y				
20614	A_23_P200222	LRP8			Y																						Y																			
5924	A_23_P29845	LRPAP1			Y	Y		Y		Y										Y	Y	Y																								
20462	A_23_P5318	LRPPRC																		Y																										
15407	A_23_P215024	LRRC1																		Y																										
20332	A_23_P253958	LRRC17			Y	Y	Y	Y	Y	Y	Y	Y	Y	Y					Y	Y	Y	Y					Y	Y	Y	Y						Y										
9109	A_23_P95559	LRRC19			Y																						Y																			
13908	A_23_P364625	LRRC19																	Y	Y																										
3547	A_23_P61487	LRRC20																Y	Y																											
11342	A_23_P84289	LRRC20																Y	Y																											
3754	A_23_P165136	LRRC25	Y		Y													Y																												
11396	A_23_P205970	LRRC28																			Y																									
12320	A_23_P253597	LRRC3			Y	Y		Y		Y												Y																								
3996	A_23_P73233	LRRC31			Y	Y		Y	Y	Y	Y	Y	Y	Y														Y		Y						Y										
13006	A_23_P132856	LRRC33			Y				Y	Y																																				
205	A_23_P118042	LRRC36			Y	Y		Y	Y	Y									Y	Y		Y	Y																							
4550	A_23_P4107	LRRC37A4			Y	Y		Y		Y																				Y						Y										
14768	A_23_P107295	LRRC37B			Y	Y		Y	Y	Y																	Y																			
7658	A_23_P103877	LRRC38	Y	Y	Y													Y									Y																			
3266	A_23_P330581	LRRC39							Y	Y																				Y						Y										
11191	A_23_P8625	LRRC4																	Y																											
22131	A_23_P148969	LRRC40																		Y																										
13723	A_23_P137396	LRRC41			Y	Y		Y		Y								Y	Y	Y		Y		Y			Y	Y	Y	Y						Y										
739	A_23_P51278	LRRC42																					Y																							
20229	A_23_P381457	LRRC45			Y													Y		Y			Y							Y						Y										
20401	A_23_P152949	LRRC46			Y																						Y																			
3841	A_23_P255701	LRRC48																Y																												
19389	A_23_P129174	LRRC49	Y	Y	Y	Y	Y	Y	Y	Y	Y	Y	Y	Y				Y	Y	Y		Y	Y	Y			Y	Y	Y	Y						Y										
14299	A_23_P24457	LRRC4C				Y			Y	Y																		Y																		
21706	A_23_P54612	LRRC50			Y																						Y			Y						Y										
17577	A_23_P161872	LRRC55				Y																						Y																		
21903	A_23_P65910	LRRC57			Y																						Y			Y						Y										
22256	A_23_P207481	LRRC59			Y	Y		Y	Y	Y									Y	Y		Y																								
21702	A_23_P112004	LRRC6	Y	Y	Y	Y	Y	Y	Y	Y	Y	Y															Y	Y	Y	Y																
13698	A_23_P115167	LRRC8B																									Y																			

19725	A_23_P97365	LRRC8D							Y	Y																																			
22054	A_23_P157527	LRRCC1			Y	Y		Y		Y										Y																									
4256	A_23_P56416	LRRFIP1			Y	Y		Y	Y	Y	Y	Y	Y	Y									Y				Y	Y	Y	Y						Y										
852	A_23_P211807	LRRFIP2			Y																																									
3388	A_23_P331072	LRRIQ3				Y			Y	Y	Y	Y	Y	Y						Y			Y					Y		Y						Y										
19123	A_23_P338253	LRRK1				Y			Y	Y									Y			Y																								
12146	A_23_P128447	LRRK2																		Y		Y																								
11910	A_23_P11843	LRRN2							Y	Y																																			
13810	A_23_P31376	LRRN3																Y												Y						Y										
10900	A_23_P161954	LRRN4CL																									Y																			
15463	A_23_P131330	LRRTM1			Y																																									
17989	A_23_P123916	LRSAM1																		Y																										
13749	A_23_P40856	LRTM1																												Y						Y										
15281	A_23_P98763	LRTOMT				Y			Y	Y																	Y			Y						Y										
15521	A_23_P301851	LSAMP			Y	Y		Y		Y								Y									Y																			
1182	A_23_P39251	LSDP5			Y	Y		Y	Y	Y																	Y																			
8049	A_23_P132415	LSG1							Y	Y																																			
11898	A_23_P85630	LSM10			Y	Y	Y	Y		Y	Y	Y															Y	Y	Y	Y																
16210	A_23_P368194	LSM11			Y	Y		Y		Y																																				
8533	A_23_P307430	LSM12																Y	Y	Y	Y	Y		Y																						
6576	A_23_P56069	LSM14A				Y													Y	Y								Y																		
700	A_23_P362719	LSM14B			Y														Y	Y			Y																							
16581	A_23_P210981	LSM14B			Y	Y		Y		Y										Y		Y					Y																			
18501	A_23_P323783	LSM14B																Y																												
19489	A_23_P59147	LSM2																									Y			Y						Y										
891	A_23_P153757	LSM4			Y	Y	Y	Y	Y	Y	Y	Y						Y	Y	Y	Y	Y		Y			Y			Y						Y						Y				
16779	A_23_P93750	LSM5																		Y																										
14733	A_23_P259451	LSM6	Y	Y	Y															Y										Y						Y										
2072	A_23_P90463	LSM7			Y	Y	Y	Y	Y	Y	Y	Y								Y							Y	Y	Y	Y						Y										
15258	A_23_P38206	LSMD1			Y																																									
9821	A_23_P13382	LSP1			Y										Y			Y		Y			Y																							
9612	A_23_P142389	LSR			Y	Y		Y	Y	Y								Y	Y	Y		Y	Y	Y			Y			Y						Y										
13789	A_23_P327440	LSS			Y													Y	Y	Y		Y		Y			Y																			
14350	A_23_P214617	LST1	Y		Y				Y	Y																	Y																			
9400	A_23_P156683	LTA			Y	Y		Y	Y	Y										Y							Y			Y						Y										
9016	A_23_P388670	LTA4H			Y				Y	Y								Y		Y							Y																			
20216	A_23_P204593	LTA4H			Y				Y	Y								Y	Y	Y				Y			Y																			
3349	A_23_P93348	LTB	Y																																											
476	A_23_P163173	LTB4R2			Y				Y	Y										Y							Y																			
19874	A_23_P218144	LTBP2													Y			Y		Y													Y													
17249	A_23_P75479	LTBP3																		Y																										
14781	A_23_P141946	LTBP4																		Y		Y	Y																							
8654	A_23_P53558	LTBR			Y	Y		Y	Y	Y								Y	Y	Y	Y	Y	Y	Y	Y	Y	Y			Y	Y	Y				Y										
9378	A_23_P92802	LTC4S																		Y			Y																							
11787	A_23_P166847	LTF																Y		Y			Y				Y																			
6871	A_23_P59790	LUC7L2			Y	Y		Y		Y																	Y																			
9893	A_23_P22941	LUZP1							Y	Y																																			
10129	A_23_P254061	LUZPP1																					Y																							
3074	A_23_P58557	LVRN																			Y																									
13503	A_23_P134764	LY6D			Y	Y		Y	Y	Y	Y	Y	Y	Y				Y	Y								Y	Y	Y	Y						Y										
20155	A_23_P169077	LY6E			Y	Y		Y		Y																																				
20975	A_23_P8083	LY6G6C																												Y						Y										
11484	A_23_P70534	LY6G6F							Y	Y																	Y																			
7076	A_23_P134914	LY6H			Y	Y	Y	Y	Y	Y	Y	Y	Y	Y				Y	Y	Y		Y	Y	Y			Y	Y	Y	Y						Y										
15442	A_23_P397285	LY6K																Y																												
17533	A_23_P5591	LY75																									Y																			
1471	A_23_P22990	LY9				Y																																								
18154	A_23_P94230	LY96	Y												Y			Y																												
20571	A_23_P41327	LYAR							Y	Y																																			
15571	A_23_P165710	LYG1																		Y		Y	Y																							
11212	A_23_P5281	LYL1			Y				Y	Y								Y	Y	Y	Y	Y	Y	Y	Y	Y					Y															
19236	A_23_P147431	LYN	Y	Y	Y	Y		Y	Y	Y	Y	Y	Y	Y						Y			Y				Y	Y	Y	Y						Y										
11093	A_23_P111981	LYNX1			Y	Y		Y	Y	Y								Y		Y							Y	Y	Y	Y																
15606	A_23_P123376	LYNX1			Y																						Y			Y						Y										
19634	A_23_P257672	LYNX1			Y	Y	Y	Y	Y	Y	Y	Y								Y		Y	Y				Y																			
5289	A_23_P419696	LYPD1			Y	Y	Y	Y	Y	Y	Y	Y	Y	Y				Y	Y	Y		Y	Y	Y			Y	Y	Y	Y																
2771	A_23_P94186	LYPD2			Y																						Y																			
16270	A_23_P39265	LYPD3				Y			Y	Y																																				
12415	A_23_P311780	LYPD4																Y																												
15564	A_23_P90704	LYPD6			Y													Y	Y	Y	Y	Y	Y	Y	Y	Y					Y															
921	A_23_P79302	LYPD6B			Y				Y	Y	Y	Y	Y	Y				Y	Y	Y		Y	Y	Y			Y	Y	Y	Y						Y						Y				
3780	A_23_P214091	LYPLA1			Y				Y	Y																																				
15383	A_23_P96798	LYPLA2			Y	Y		Y		Y								Y	Y	Y		Y		Y			Y																			
18184	A_23_P54929	LYRM1																		Y			Y																							
10290	A_23_P42035	LYRM2			Y	Y		Y		Y																																				
24	A_23_P330490	LYSMD2																Y	Y																											
8090	A_23_P354074	LYST			Y	Y	Y	Y	Y	Y	Y	Y	Y	Y				Y	Y	Y		Y		Y			Y	Y	Y	Y						Y										

19481	A_23_P76192	LYZ	Y	Y	Y	Y		Y		Y																																				
18563	A_23_P121144	LYZL4							Y	Y																																				
12596	A_23_P784	LZIC																		Y																										
15732	A_23_P41049	LZTFL1	Y		Y	Y	Y	Y	Y	Y	Y	Y	Y	Y				Y	Y	Y		Y	Y	Y			Y	Y	Y	Y	Y	Y	Y	Y	Y	Y										
16523	A_23_P20446	LZTS1																					Y																							
18253	A_23_P204257	M6PR			Y				Y	Y																	Y																			
6169	A_23_P101707	M6PRBP1			Y	Y	Y	Y	Y	Y	Y	Y									Y	Y	Y	Y				Y																			
8644	A_23_P53881	MAB21L1			Y	Y		Y		Y								Y																												
11729	A_23_P217850	MACF1			Y	Y		Y	Y	Y									Y	Y		Y					Y	Y	Y	Y						Y										
15987	A_23_P86850	MACROD1																		Y																										
10106	A_23_P22398	MACROD2			Y	Y	Y	Y	Y	Y	Y	Y						Y	Y	Y	Y	Y	Y	Y	Y	Y	Y				Y															
2053	A_23_P42657	MAD1L1			Y	Y	Y	Y	Y	Y	Y	Y								Y	Y	Y	Y				Y	Y	Y	Y						Y										
669	A_23_P92441	MAD2L1	Y	Y	Y																																									
5322	A_23_P23206	MAD2L2			Y	Y	Y	Y		Y	Y	Y						Y	Y	Y		Y	Y	Y			Y	Y	Y	Y						Y										
12672	A_23_P153615	MADCAM1				Y																						Y																		
21696	A_23_P202708	MADD			Y	Y		Y		Y										Y			Y				Y																			
19822	A_23_P114932	MAEL																									Y																			
10205	A_23_P397376	MAF			Y				Y	Y													Y																							
3891	A_23_P33072	MAF1			Y	Y	Y	Y	Y	Y	Y	Y	Y	Y				Y	Y	Y	Y	Y	Y	Y	Y	Y	Y	Y	Y	Y	Y	Y	Y	Y	Y	Y		Y				Y			Y	Y
11575	A_23_P17345	MAFB	Y																																											
18728	A_23_P325025	MAFB			Y															Y			Y				Y																			
7019	A_23_P103110	MAFF			Y	Y	Y	Y		Y	Y	Y						Y		Y	Y	Y					Y			Y						Y										
85	A_23_P78209	MAFG																					Y																							
660	A_23_P390044	MAFG																		Y			Y																							
17670	A_23_P207759	MAFG			Y													Y	Y	Y	Y	Y	Y	Y	Y	Y					Y															
11739	A_23_P373598	MAFK				Y														Y	Y	Y	Y																							
13413	A_23_P111698	MAFK			Y	Y		Y	Y	Y										Y								Y																		
5519	A_23_P16553	MAG			Y													Y	Y	Y	Y	Y	Y	Y	Y	Y	Y			Y	Y	Y				Y										
17732	A_23_P96295	MAGEA1																		Y																										
9841	A_23_P137007	MAGEA10	Y		Y				Y	Y								Y	Y	Y				Y			Y	Y	Y	Y																
10951	A_23_P252928	MAGEA12																		Y										Y						Y										
C10323.7	A_23_P62095	MAGEA3																												Y						Y										
7816	A_23_P171175	MAGEA4			Y															Y																										
11224	A_23_P148399	MAGEA5																		Y										Y						Y										
C8748.9	A_23_P136870	MAGEA6			Y																																									
13169	A_23_P217554	MAGEA8			Y															Y							Y			Y						Y										
14425	A_23_P85094	MAGEA9			Y				Y	Y																	Y			Y						Y										
19653	A_23_P378574	MAGEB6			Y				Y	Y										Y			Y				Y																			
18419	A_23_P217178	MAGEC1																		Y							Y																			
8863	A_23_P62099	MAGEC2																Y									Y																			
11158	A_23_P502081	MAGEC3			Y																						Y			Y						Y										
9161	A_23_P124242	MAGED1							Y	Y																																				
19612	A_23_P135634	MAGED1							Y	Y																																				
19610	A_23_P33898	MAGED2	Y	Y	Y	Y		Y	Y	Y	Y	Y	Y	Y													Y	Y	Y	Y						Y										
4190	A_23_P159907	MAGED4B			Y	Y		Y	Y	Y								Y		Y		Y								Y						Y										
5390	A_23_P7033	MAGEF1			Y	Y		Y	Y	Y										Y		Y	Y				Y			Y						Y										
9004	A_23_P99996	MAGEL2																		Y																										
18205	A_23_P96099	MAGI1																		Y		Y						Y																		
19080	A_23_P21376	MAGI2	Y	Y	Y		Y		Y	Y	Y	Y						Y	Y	Y				Y			Y			Y						Y										
17015	A_23_P84941	MAGIX			Y	Y		Y		Y								Y	Y	Y				Y			Y	Y	Y	Y																
13308	A_23_P206830	Magmas																										Y																		
3385	A_23_P200211	MAGOH																		Y	Y		Y																							
15755	A_23_P148273	MAGT1			Y	Y		Y		Y																																				
7390	A_23_P214839	MAK			Y				Y	Y																	Y																			
18171	A_23_P20676	MAK10																Y	Y	Y			Y	Y																						
22284	A_23_P111961	MAK16							Y	Y																																				
21119	A_23_P17134	MAL							Y	Y																	Y																			
12168	A_23_P21150	MALAT1				Y			Y	Y																		Y		Y						Y										
2171	A_23_P102551	MALL			Y																						Y																			
11211	A_23_P96008	MALT1																					Y																							
17523	A_23_P157914	MAMDC2							Y	Y	Y	Y	Y	Y													Y			Y						Y										
13371	A_23_P71787	MAMDC4																		Y										Y						Y										
21441	A_23_P69566	MAML3																		Y		Y	Y																							
13940	A_23_P251075	MAMLD1																		Y																										
20742	A_23_P156431	MAN1A1				Y			Y	Y																																				
17012	A_23_P103571	MAN1A2																										Y		Y						Y										
16986	A_23_P135817	MAN1B1																				Y																								
17779	A_23_P157939	MAN1B1			Y				Y	Y																	Y			Y						Y										
19963	A_23_P94857	MAN1B1			Y	Y	Y	Y	Y	Y	Y	Y	Y	Y				Y	Y	Y	Y	Y	Y	Y	Y	Y	Y	Y	Y	Y	Y	Y	Y			Y										
21283	A_23_P171001	MAN1B1			Y	Y	Y	Y	Y	Y	Y	Y	Y	Y				Y	Y	Y	Y	Y	Y	Y	Y	Y	Y	Y	Y	Y	Y	Y	Y			Y										
10253	A_23_P18931	MAN2A1				Y																						Y																		
19985	A_23_P360769	MAN2A1				Y																						Y																		
12457	A_23_P37564	MAN2A2	Y	Y	Y	Y	Y	Y	Y	Y	Y	Y	Y	Y				Y		Y		Y	Y				Y	Y	Y	Y	Y	Y	Y	Y	Y	Y								Y		
22489	A_23_P206041	MAN2A2			Y				Y	Y										Y							Y				Y		Y	Y	Y											
20227	A_23_P27613	MAN2B1			Y				Y	Y										Y			Y				Y																			
17694	A_23_P250372	MAN2B2																									Y																			
12272	A_23_P26117	MAN2C1																		Y		Y																								

10709	A_23_P258698	MANBA																Y	Y								Y	Y	Y	Y																
14275	A_23_P131773	MANBAL			Y																																									
13525	A_23_P255663	MANEA																		Y			Y																							
3098	A_23_P96410	MAOA			Y		Y		Y	Y	Y	Y							Y	Y							Y															Y				
18268	A_23_P83857	MAOA																Y	Y	Y	Y	Y		Y						Y						Y										
18555	A_23_P85011	MAOB			Y	Y		Y	Y	Y								Y	Y	Y	Y	Y	Y	Y	Y	Y	Y			Y	Y	Y				Y										
12236	A_23_P163446	MAP1A																					Y																							
6772	A_23_P213573	MAP1B			Y	Y		Y		Y																	Y	Y	Y	Y																
21240	A_23_P90790	MAP1D																									Y																			
12163	A_23_P154786	MAP1LC3A			Y													Y	Y								Y																			
5918	A_23_P67582	MAP1S			Y	Y	Y	Y	Y	Y	Y	Y	Y	Y				Y	Y	Y	Y	Y	Y	Y	Y	Y	Y	Y	Y	Y	Y	Y				Y		Y				Y			Y	
20827	A_23_P153920	MAP2			Y				Y	Y	Y	Y	Y	Y				Y		Y							Y			Y						Y										
1873	A_23_P20248	MAP2K1				Y																																								
16195	A_23_P208839	MAP2K2			Y	Y	Y	Y	Y	Y	Y	Y	Y	Y				Y	Y	Y	Y	Y	Y	Y	Y	Y	Y			Y	Y	Y				Y								Y		
17973	A_23_P118422	MAP2K3			Y	Y	Y	Y	Y	Y	Y	Y	Y	Y				Y	Y	Y	Y	Y	Y	Y	Y	Y	Y	Y	Y	Y	Y	Y				Y							Y			
18964	A_23_P152687	MAP2K4	Y	Y	Y	Y		Y	Y	Y					Y			Y	Y	Y		Y	Y	Y			Y	Y	Y	Y						Y										
3699	A_23_P207445	MAP2K6																Y																												
11290	A_23_P78628	MAP2K7				Y																																								
21944	A_23_P41796	MAP3K1																	Y											Y						Y										
21554	A_23_P67503	MAP3K10																		Y		Y																								
14919	A_23_P98273	MAP3K11			Y	Y		Y		Y								Y	Y	Y		Y	Y	Y			Y			Y						Y										
11406	A_23_P105405	MAP3K12	Y	Y	Y	Y		Y	Y	Y																	Y	Y	Y	Y						Y										
20379	A_23_P207319	MAP3K14			Y	Y	Y	Y	Y	Y	Y	Y						Y	Y	Y		Y	Y	Y			Y																			
2504	A_23_P44004	MAP3K2			Y	Y	Y	Y	Y	Y	Y	Y							Y	Y		Y					Y	Y	Y	Y																
18130	A_23_P207138	MAP3K3			Y	Y	Y	Y	Y	Y	Y	Y	Y	Y				Y	Y	Y	Y	Y	Y	Y	Y	Y	Y				Y															
10344	A_23_P42096	MAP3K4			Y	Y	Y	Y	Y	Y	Y	Y	Y	Y				Y	Y	Y	Y	Y	Y	Y	Y	Y	Y	Y	Y	Y	Y	Y				Y										
19498	A_23_P126634	MAP3K6			Y													Y		Y		Y	Y				Y																			
12157	A_23_P500773	MAP3K7							Y	Y											Y																									
18097	A_23_P19237	MAP3K7			Y	Y		Y	Y	Y										Y							Y	Y	Y	Y						Y										
13172	A_23_P80345	MAP3K7IP1																Y	Y	Y		Y	Y	Y																						
4596	A_23_P19702	MAP3K7IP2			Y	Y		Y		Y								Y										Y																		
13270	A_23_P305033	MAP3K7IP3																		Y		Y																								
13696	A_23_P23947	MAP3K8	Y	Y																																										
10500	A_23_P65574	MAP3K9			Y															Y			Y				Y			Y						Y										
231	A_23_P211814	MAP4																		Y		Y	Y																							
7144	A_23_P5005	MAP4K1			Y																									Y						Y										
6408	A_23_P24404	MAP4K2			Y														Y	Y		Y	Y				Y																			
10153	A_23_P154130	MAP4K3			Y	Y	Y	Y	Y	Y	Y	Y	Y	Y				Y	Y	Y		Y	Y	Y			Y	Y	Y	Y						Y										
22197	A_23_P108604	MAP4K4				Y			Y	Y	Y	Y	Y	Y						Y							Y			Y						Y										
46	A_23_P205646	MAP4K5			Y	Y		Y	Y	Y									Y	Y							Y	Y	Y	Y																
4651	A_23_P350491	MAP4K5																												Y						Y										
18966	A_23_P47725	MAP6			Y	Y		Y	Y	Y								Y	Y	Y		Y		Y			Y			Y						Y										
7265	A_23_P80839	MAP6D1			Y																						Y																			
4944	A_23_P122736	MAP7			Y				Y	Y																																				
13033	A_23_P149075	MAP7D1			Y	Y	Y	Y	Y	Y	Y	Y	Y	Y				Y	Y	Y	Y	Y	Y	Y	Y	Y	Y	Y	Y	Y	Y	Y	Y			Y				Y						
17128	A_23_P92370	MAP9			Y	Y		Y		Y								Y	Y	Y		Y		Y			Y	Y	Y	Y						Y										
5249	A_23_P257895	MAPK1				Y																																								
1748	A_23_P45025	MAPK10																									Y																			
9739	A_23_P502274	MAPK11																				Y																								
20005	A_23_P252955	MAPK11			Y														Y	Y			Y				Y																			
7653	A_23_P40750	MAPK12				Y																Y																								
10577	A_23_P145376	MAPK13			Y		Y		Y	Y	Y	Y						Y		Y							Y																			
21036	A_23_P60962	MAPK15																		Y		Y	Y				Y	Y	Y	Y						Y										
8643	A_23_P140309	MAPK1IP1L			Y	Y		Y		Y																	Y																			
9707	A_23_P37907	MAPK3																Y	Y	Y		Y		Y																						
9176	A_23_P208101	MAPK4				Y			Y	Y										Y																										
6650	A_23_P100703	MAPK7																		Y			Y																							
11352	A_23_P202540	MAPK8			Y																																									
460	A_23_P116005	MAPK8IP1																		Y		Y	Y							Y						Y										
8849	A_23_P502078	MAPK8IP2			Y	Y		Y	Y	Y								Y		Y		Y					Y			Y						Y										
22278	A_23_P416048	MAPK8IP3																Y										Y																		
297	A_23_P216891	MAPKAP1			Y	Y		Y		Y								Y	Y	Y	Y	Y	Y	Y	Y	Y	Y				Y															
13384	A_23_P350467	MAPKAPK2			Y															Y																										
14159	A_23_P201483	MAPKAPK2			Y	Y	Y	Y	Y	Y	Y	Y							Y	Y		Y	Y				Y																			
7033	A_23_P314584	MAPKAPK3																		Y		Y																								
14778	A_23_P212413	MAPKAPK3				Y													Y																											
235	A_23_P76412	MAPKAPK5																Y	Y	Y	Y	Y	Y	Y	Y	Y					Y															
3087	A_23_P110362	MAPKSP1			Y	Y		Y		Y										Y			Y				Y			Y						Y										
17809	A_23_P418015	MAPRE2			Y				Y	Y																	Y			Y						Y										
1002	A_23_P207699	MAPT			Y																		Y				Y																			
5237	A_23_P47777	MARCH5_																			Y																									
1919	A_23_P33686	MARCH6_			Y	Y	Y	Y	Y	Y	Y	Y	Y	Y				Y		Y							Y	Y	Y	Y						Y										
12340	A_23_P12603	MARCH7_			Y	Y		Y	Y	Y									Y	Y	Y	Y	Y				Y	Y	Y	Y						Y										
8466	A_23_P102242	MARCH8_			Y															Y			Y																							
22272	A_23_P110492	MARCH9_			Y	Y		Y	Y	Y										Y	Y		Y					Y		Y						Y										
2881	A_23_P214222	MARCKS	Y		Y																		Y				Y																			
19110	A_23_P101992	MARCO	Y	Y	Y	Y		Y	Y	Y								Y									Y	Y	Y	Y						Y										

3178	A_23_P161595	MARK2			Y	Y	Y	Y		Y	Y	Y						Y	Y	Y		Y	Y	Y			Y			Y						Y						Y				
20723	A_23_P107717	MARK4			Y															Y							Y																			
21422	A_23_P128036	MARS			Y	Y	Y	Y		Y	Y	Y	Y	Y				Y	Y	Y	Y	Y	Y	Y	Y	Y	Y	Y	Y	Y	Y	Y	Y													
3618	A_23_P108492	MARS2				Y			Y	Y	Y	Y	Y	Y									Y																							
20056	A_23_P401675	MARVELD2			Y				Y	Y	Y	Y	Y	Y																Y						Y										
3161	A_23_P152428	MARVELD3			Y																						Y																			
17459	A_23_P168339	MAS1																												Y						Y										
13389	A_23_P214562	MAS1L			Y				Y	Y								Y	Y	Y		Y		Y			Y																			
15130	A_23_P212267	MASP1			Y															Y	Y	Y	Y																							
4429	A_23_P85672	MASP2																					Y																							
5894	A_23_P101480	MAST1																		Y		Y	Y																							
5072	A_23_P126006	MAST2																Y	Y	Y				Y																						
11506	A_23_P110571	MAST4	Y	Y	Y	Y		Y	Y	Y	Y	Y	Y	Y					Y	Y							Y	Y	Y	Y						Y										
22065	A_23_P401568	MAT2A			Y																																									
13356	A_23_P81408	MAT2B			Y																																									
11483	A_23_P50678	MATK			Y	Y		Y	Y	Y									Y	Y		Y	Y				Y	Y	Y	Y																
6391	A_23_P97677	MATN1																			Y																									
17735	A_23_P71328	MATN2							Y	Y																				Y						Y										
14787	A_23_P102058	MATN3			Y	Y	Y	Y	Y	Y	Y	Y							Y	Y							Y	Y	Y	Y						Y										
19298	A_23_P102622	MATN4			Y	Y		Y	Y	Y	Y	Y	Y	Y						Y			Y				Y			Y						Y										
5133	A_23_P70047	MATR3			Y	Y	Y	Y	Y	Y	Y	Y								Y		Y	Y				Y	Y	Y	Y						Y										
1826	A_23_P6433	MB							Y	Y								Y	Y	Y		Y		Y						Y						Y										
18457	A_23_P15864	MBD2	Y	Y																							Y																			
9154	A_23_P50837	MBD3																		Y		Y	Y																							
1226	A_23_P415395	MBD3L1			Y																																									
10532	A_23_P92154	MBD4				Y													Y	Y		Y	Y																							
3556	A_23_P142537	MBD5			Y	Y		Y		Y																				Y						Y										
21539	A_23_P393139	MBD6	Y	Y	Y	Y		Y	Y	Y	Y	Y	Y	Y					Y			Y	Y				Y	Y	Y	Y						Y										
16588	A_23_P2922	MBIP			Y	Y		Y	Y	Y																		Y		Y						Y										
16054	A_23_P96205	MBNL3																												Y						Y										
2195	A_23_P370637	MBOAT2																	Y																											
1365	A_23_P208085	MBP																												Y						Y										
17083	A_23_P107238	MBTD1																												Y						Y										
17268	A_23_P114095	MBTPS2																		Y			Y																							
957	A_23_P329271	MC1R							Y	Y										Y																										
3534	A_23_P66095	MC1R				Y												Y	Y	Y		Y		Y																						
3510	A_23_P38649	MC2R			Y																		Y				Y			Y						Y										
19284	A_23_P50039	MC5R			Y																							Y		Y						Y										
2907	A_23_P162171	MCAM			Y													Y		Y		Y	Y																							
19300	A_23_P132358	MCAT							Y	Y																				Y						Y										
2696	A_23_P95705	MCC			Y	Y		Y		Y																		Y		Y						Y										
11572	A_23_P377114	MCC			Y	Y		Y	Y	Y																	Y	Y	Y	Y						Y										
19398	A_23_P58036	MCCC1																									Y																			
2018	A_23_P18887	MCCC2			Y				Y	Y	Y	Y	Y	Y				Y		Y			Y				Y			Y						Y										
20139	A_23_P56654	MCEE																		Y			Y																							
2514	A_23_P45536	MCF2																									Y																			
743	A_23_P99498	MCF2L			Y	Y		Y	Y	Y	Y	Y	Y	Y														Y		Y						Y										
18168	A_23_P88099	MCF2L			Y	Y		Y	Y	Y																																				
2954	A_23_P211541	MCHR1				Y			Y	Y																																				
12041	A_23_P156784	MCHR2				Y													Y									Y																		
20429	A_23_P161474	MCM10																Y																												
1700	A_23_P250801	MCM2																Y																												
7602	A_23_P7873	MCM3																Y		Y																										
15192	A_23_P256694	MCM3APAS			Y	Y		Y	Y	Y																																				
21875	A_23_P370993	MCM4			Y													Y	Y	Y	Y			Y			Y	Y	Y	Y																
6838	A_23_P132277	MCM5			Y	Y	Y	Y	Y	Y	Y	Y	Y	Y				Y	Y	Y	Y	Y	Y	Y	Y	Y	Y	Y	Y	Y	Y	Y				Y				Y						
2221	A_23_P93690	MCM7			Y													Y	Y	Y				Y			Y																			
1093	A_23_P68547	MCM8			Y	Y		Y		Y																	Y	Y	Y	Y																
11772	A_23_P397347	MCM9			Y	Y	Y	Y	Y	Y	Y	Y						Y	Y	Y	Y	Y	Y	Y	Y	Y	Y			Y	Y	Y				Y										
10220	A_23_P27571	MCOLN1			Y	Y	Y	Y	Y	Y	Y	Y	Y	Y				Y	Y	Y	Y	Y	Y	Y	Y	Y	Y	Y	Y	Y	Y	Y	Y			Y										
9649	A_23_P12238	MCOLN3																	Y																											
20342	A_23_P84353	MCPH1			Y	Y		Y		Y																	Y																			
8335	A_23_P48237	MCRS1			Y	Y		Y		Y								Y	Y	Y		Y	Y	Y			Y																			
7125	A_23_P133293	MCTP1																		Y																										
20211	A_23_P114282	MCTS1			Y																																									
13162	A_23_P19455	MDC1				Y																																								
5963	A_23_P95650	MDGA2																										Y																		
7841	A_23_P31366	MDH2			Y	Y	Y	Y		Y	Y	Y						Y	Y	Y				Y			Y	Y	Y	Y																
20842	A_23_P204782	MDM1			Y	Y	Y	Y	Y	Y	Y	Y								Y							Y	Y	Y	Y						Y										
8802	A_23_P170969	MDM4	Y	Y																																										
9425	A_23_P103502	MDM4	Y	Y	Y	Y	Y	Y	Y	Y	Y	Y	Y	Y				Y				Y					Y	Y	Y	Y						Y										
1458	A_23_P365060	MDN1																									Y																			
4240	A_23_P8196	ME1				Y																																								
21075	A_23_P422026	ME1			Y	Y		Y	Y	Y	Y	Y	Y	Y														Y		Y						Y										
10895	A_23_P321261	ME2	Y	Y																										Y						Y										
15274	A_23_P116614	ME3			Y				Y	Y								Y		Y							Y			Y						Y										
21373	A_23_P30745	MEA1			Y													Y		Y							Y																			

19562	A_23_P114361	MECP2				Y																																								
11094	A_23_P114808	MECR			Y	Y		Y		Y								Y	Y	Y		Y		Y			Y																			
645	A_23_P124022	MED10											Y					Y		Y																										
18482	A_23_P47991	MED13L	Y	Y	Y	Y	Y	Y	Y	Y	Y	Y						Y	Y	Y	Y	Y	Y	Y	Y	Y	Y	Y	Y	Y	Y	Y				Y										
20362	A_23_P72187	MED14							Y	Y										Y																										
10403	A_23_P154954	MED15				Y																																								
3630	A_23_P56170	MED16			Y													Y	Y	Y		Y	Y	Y			Y																			
6557	A_23_P116023	MED17			Y													Y	Y	Y		Y	Y	Y																						
6209	A_23_P219028	MED20			Y	Y		Y	Y	Y	Y	Y	Y	Y					Y	Y		Y	Y				Y	Y	Y	Y						Y										
7769	A_23_P219154	MED22				Y																																								
15657	A_23_P501795	MED22			Y	Y	Y	Y		Y	Y	Y						Y	Y	Y	Y	Y	Y	Y	Y	Y	Y				Y															
13405	A_23_P145501	MED23			Y	Y	Y	Y	Y	Y	Y	Y	Y	Y				Y	Y	Y	Y	Y	Y	Y	Y	Y	Y	Y	Y	Y	Y	Y				Y										
8979	A_23_P124760	MED24			Y				Y	Y										Y	Y	Y	Y																							
6618	A_23_P164737	MED26																Y		Y			Y																							
16336	A_23_P317854	MED26			Y	Y	Y	Y	Y	Y	Y	Y							Y	Y			Y				Y	Y	Y	Y						Y								Y		
21873	A_23_P301871	MED27				Y																						Y		Y						Y										
21186	A_23_P7201	MED28			Y				Y	Y																				Y						Y										
17838	A_23_P31866	MED30							Y	Y	Y	Y	Y	Y																Y						Y										
11750	A_23_P128543	MED4				Y												Y										Y																		
14841	A_23_P30307	MED7				Y																					Y																			
7537	A_23_P46170	MED8																			Y																									
16244	A_23_P66402	MED9	Y	Y	Y															Y							Y																			
2409	A_23_P205939	MEF2A			Y																						Y			Y						Y										
10779	A_23_P353652	MEF2A																		Y																										
13187	A_23_P153786	MEF2B			Y	Y	Y	Y	Y	Y	Y	Y						Y	Y	Y		Y		Y			Y	Y	Y	Y																
16911	A_23_P320739	MEF2C																												Y						Y										
9922	A_23_P334182	MEF2D			Y																																									
14031	A_23_P51679	MEF2D																		Y			Y																							
4900	A_23_P140967	MEFV																					Y																							
21759	A_23_P213893	MEGF10							Y	Y																																				
9570	A_23_P54340	MEGF11			Y															Y		Y	Y				Y	Y	Y	Y						Y										
9219	A_23_P51181	MEGF6																Y		Y		Y	Y				Y																			
1669	A_23_P335206	MEGF8																		Y		Y	Y																							
15903	A_23_P353097	MEGF8			Y				Y	Y	Y	Y	Y	Y													Y			Y						Y										
7158	A_23_P211561	MEI1	Y	Y	Y				Y	Y																	Y																			
5808	A_23_P28324	MEIS1							Y	Y																																				
8672	A_23_P39731	MEIS1																		Y																										
3376	A_23_P78795	MEIS3																				Y																								
7152	A_23_P16787	MEMO1																									Y																			
15111	A_23_P75453	MEN1			Y														Y	Y		Y	Y							Y						Y										
11944	A_23_P107173	MEOX1			Y													Y	Y	Y		Y		Y																						
19344	A_23_P93122	MEP1A																	Y	Y	Y		Y																							
1251	A_23_P78353	MEP1B			Y																																									
11336	A_23_P42630	MEPCE																	Y	Y		Y	Y																							
13211	A_23_P99891	MESDC1																									Y																			
20406	A_23_P88503	MESDC2																					Y																							
20890	A_23_P77223	MESP1							Y	Y								Y	Y											Y						Y										
17974	A_23_P155907	METAP1				Y																																								
9356	A_23_P99260	METAP2				Y																																								
5856	A_23_P49256	METRN	Y	Y																																										
7561	A_23_P10591	METRNL			Y	Y		Y		Y																																				
4285	A_23_P365705	METT10D			Y																						Y			Y						Y										
15481	A_23_P153059	METT10D																										Y																		
10776	A_23_P420911	METT5D1			Y	Y	Y	Y	Y	Y	Y	Y	Y	Y				Y	Y	Y		Y	Y	Y			Y	Y	Y	Y	Y	Y	Y	Y	Y	Y				Y						
19194	A_23_P47788	METTL1																		Y																										
897	A_23_P115896	METTL10				Y			Y	Y									Y	Y										Y						Y										
21765	A_23_P71905	METTL11A			Y	Y	Y	Y	Y	Y	Y	Y	Y	Y						Y		Y					Y	Y	Y	Y						Y										
10593	A_23_P415021	METTL7A							Y	Y													Y																							
2365	A_23_P385162	METTL7B			Y	Y		Y	Y	Y													Y				Y																			
15931	A_23_P56555	METTL8																			Y		Y										Y													
4396	A_23_P206347	METTL9			Y	Y		Y	Y	Y									Y	Y		Y					Y	Y	Y	Y						Y										
68	A_23_P50137	MEX3C																		Y	Y																									
3866	A_23_P14636	MFAP1			Y	Y	Y	Y	Y	Y	Y	Y								Y			Y				Y																			
13708	A_23_P1021	MFAP2																		Y																										
8337	A_23_P167711	MFAP3			Y																						Y																			
12189	A_23_P58188	MFAP3L																		Y		Y	Y																							
3237	A_23_P87700	MFAP5													Y																															
6708	A_23_P405148	MFF																Y	Y	Y				Y																						
11937	A_23_P92994	MFF			Y	Y		Y		Y									Y	Y	Y	Y	Y				Y																			
14410	A_23_P48951	MFGE8			Y				Y	Y	Y	Y	Y	Y				Y	Y	Y		Y		Y			Y			Y						Y										
18919	A_23_P112078	MFHAS1				Y			Y	Y	Y	Y	Y	Y													Y			Y						Y										
19848	A_23_P212042	MFI2			Y	Y	Y	Y	Y	Y	Y	Y	Y	Y						Y							Y			Y						Y										
4937	A_23_P80648	MFN1																		Y		Y																								
9634	A_23_P126135	MFN2							Y	Y										Y																										
19055	A_23_P103100	MFNG			Y														Y	Y			Y																							
17568	A_23_P13562	MFRP																Y		Y			Y																							
17603	A_23_P166677	MFSD1	Y	Y	Y																																									

14614	A_23_P41246	MFSD10			Y	Y	Y	Y		Y	Y	Y						Y	Y	Y	Y	Y	Y	Y	Y	Y	Y	Y	Y	Y	Y	Y														
1374	A_23_P26704	MFSD11			Y																																									
14020	A_23_P253781	MFSD2				Y			Y	Y																																				
18854	A_23_P43817	MFSD2							Y	Y																				Y						Y										
20634	A_23_P28530	MFSD6																	Y																											
9947	A_23_P340218	MFSD6L							Y	Y											Y																									
4896	A_23_P72157	MFSD7																Y			Y																									
547	A_23_P30162	MFSD8																									Y																			
8353	A_23_P154158	MFSD9				Y																	Y				Y	Y	Y	Y																
3233	A_23_P37654	MGA			Y	Y		Y		Y										Y		Y																								
14519	A_23_P49049	MGA				Y														Y																										
13608	A_23_P42897	MGAM			Y														Y								Y																			
5978	A_23_P58476	MGAT1			Y	Y	Y	Y	Y	Y	Y	Y			Y			Y	Y	Y	Y		Y	Y	Y		Y																			
21747	A_23_P65558	MGAT2	Y																																											
8144	A_23_P91742	MGAT3																		Y																										
1372	A_23_P28507	MGAT4A																	Y	Y		Y	Y				Y			Y						Y										
9236	A_23_P213640	MGAT4B			Y	Y		Y		Y								Y	Y	Y		Y		Y																						
18470	A_23_P79032	MGC10814			Y	Y	Y	Y	Y	Y	Y	Y	Y	Y				Y	Y	Y			Y	Y			Y	Y	Y	Y						Y										
5738	A_23_P154294	MGC13005			Y	Y		Y		Y									Y	Y	Y	Y	Y				Y			Y						Y										
19773	A_23_P104138	MGC15634			Y	Y	Y	Y	Y	Y	Y	Y						Y	Y	Y		Y		Y			Y																			
802	A_23_P255027	MGC15705																			Y																									
15300	A_23_P79572	MGC16025			Y		Y		Y	Y	Y	Y							Y								Y																			
14603	A_23_P8363	MGC16075			Y																						Y	Y	Y	Y																
15025	A_23_P159986	MGC16121																									Y																			
20130	A_23_P133075	MGC16169			Y	Y	Y	Y	Y	Y	Y	Y							Y	Y							Y	Y	Y	Y						Y										
12836	A_23_P63736	MGC16291							Y	Y																																				
18333	A_23_P105361	MGC16384			Y	Y		Y	Y	Y																		Y		Y						Y										
13588	A_23_P343843	MGC16385																Y	Y	Y		Y		Y																						
21195	A_23_P408232	MGC23270																Y	Y			Y					Y																			
19301	A_23_P373100	MGC24103			Y	Y	Y	Y	Y	Y	Y	Y	Y	Y				Y	Y	Y		Y		Y			Y	Y	Y	Y						Y										
14831	A_23_P379106	MGC24125																												Y						Y										
13338	A_23_P372984	MGC24975																										Y		Y						Y										
5761	A_23_P389423	MGC26597			Y																						Y																			
17106	A_23_P393697	MGC27345			Y																						Y																			
12062	A_23_P73028	MGC2848				Y			Y	Y																																				
13841	A_23_P84596	MGC29506			Y		Y		Y	Y	Y	Y	Y	Y					Y								Y																			
10533	A_23_P355500	MGC3207			Y				Y	Y								Y		Y		Y																								
15110	A_23_P319682	MGC33407				Y																						Y																		
10516	A_23_P432448	MGC3771	Y	Y	Y	Y		Y		Y													Y				Y																			
6187	A_23_P348911	MGC40574			Y																																									
12471	A_23_P254863	MGC42105	Y		Y														Y	Y							Y																			
20613	A_23_P102681	MGC4294																		Y																										
5599	A_23_P405282	MGC45922			Y	Y		Y		Y										Y		Y	Y																							
15447	A_23_P350719	MGC52282			Y	Y		Y		Y								Y		Y			Y					Y																		
15828	A_23_P206648	MGC52282			Y	Y		Y		Y																	Y	Y	Y	Y																
11310	A_23_P139895	MGC5370			Y	Y		Y	Y	Y										Y							Y			Y						Y										
6096	A_23_P254181	MGC5566			Y															Y							Y																			
1721	A_23_P256055	MGC70863			Y	Y		Y	Y	Y																	Y																			
7086	A_23_P202170	MGEA5			Y	Y	Y	Y	Y	Y	Y	Y	Y	Y					Y	Y		Y					Y	Y	Y	Y						Y										
3046	A_23_P80438	MGLL																			Y																									
4555	A_23_P110167	MGST2									Y		Y	Y					Y																											
18282	A_23_P51548	MGST3																									Y																			
C10288	A_23_P4714	MIA							Y	Y																	Y			Y						Y										
16367	A_23_P117387	MIA2			Y																						Y	Y	Y	Y						Y										
2012	A_23_P433838	MIB1																												Y						Y										
9637	A_23_P436103	MIB2																					Y																							
10026	A_23_P214984	MICAL1			Y	Y		Y		Y									Y	Y	Y	Y	Y																							
6722	A_23_P2041	MICALCL			Y				Y	Y	Y	Y	Y	Y						Y			Y				Y			Y						Y										
8673	A_23_P68922	MICALL1			Y	Y	Y	Y	Y	Y	Y	Y						Y	Y	Y		Y		Y						Y						Y										
2070	A_23_P258220	MICALL2																Y		Y	Y	Y	Y																							
4489	A_23_P122516	MICB																									Y																			
3289	A_23_P10031	MID1							Y	Y																																				
7259	A_23_P170037	MID1							Y	Y																																				
16016	A_23_P32838	MID1							Y	Y																																				
9780	A_23_P305723	MIER1																		Y										Y						Y										
12365	A_23_P119683	MIER2																		Y																										
7484	A_23_P18993	MIER3																		Y			Y																							
1275	A_23_P91619	MIF			Y	Y		Y	Y	Y																	Y	Y	Y	Y						Y										
17783	A_23_P49566	MIF4GD			Y	Y		Y		Y								Y	Y	Y		Y	Y	Y			Y			Y						Y										
21430	A_23_P91827	MINA			Y																																									
11517	A_23_P164307	MINK1			Y															Y			Y							Y						Y										
8721	A_23_P115885	MINPP1			Y	Y		Y	Y	Y										Y							Y																			
2253	A_23_P68998	MIOX																												Y						Y										
634	A_23_P47806	MIP				Y																						Y																		
13054	A_23_P117274	MIPEP							Y	Y																																				
10047	A_23_P300867	MIPOL1	Y		Y				Y	Y	Y	Y	Y	Y				Y	Y	Y	Y	Y	Y	Y	Y	Y	Y	Y	Y	Y	Y	Y				Y										
7946	A_23_P78149	MIS12			Y	Y	Y	Y		Y	Y	Y						Y	Y	Y	Y	Y	Y	Y	Y	Y	Y	Y	Y	Y	Y	Y														

18878	A_23_P311020	MITD1																		Y																										
4253	A_23_P13176	MIZF				Y																																								
20400	A_23_P79962	MKKS																		Y		Y																								
4332	A_23_P348138	MKL1			Y	Y	Y	Y	Y	Y	Y	Y	Y	Y				Y	Y	Y	Y	Y	Y	Y	Y	Y	Y			Y	Y	Y	Y			Y							Y			
15361	A_23_P54556	MKL2	Y	Y	Y	Y	Y	Y	Y	Y	Y	Y	Y	Y				Y		Y			Y				Y	Y	Y	Y						Y						Y				
22536	A_23_P93613	MKLN1																	Y	Y																										
2066	A_23_P142305	MKNK2			Y	Y		Y	Y	Y								Y	Y	Y	Y	Y	Y	Y	Y	Y	Y				Y															
15399	A_23_P57807	MKRN2			Y				Y	Y										Y			Y																							
10098	A_23_P21339	MKRN3				Y			Y	Y																	Y			Y						Y										
12184	A_23_P129778	MKS1																	Y																											
C10163.6	A_23_P43350	MLANA	Y	Y	Y													Y									Y																			
15997	A_23_P2554	MLEC																												Y						Y										
3137	A_23_P143906	MLF1							Y	Y																																				
16683	A_23_P13872	MLF2			Y	Y		Y		Y								Y	Y	Y	Y	Y	Y	Y	Y	Y	Y	Y	Y	Y	Y	Y				Y				Y						
15551	A_23_P69058	MLH1			Y	Y		Y	Y	Y										Y							Y	Y	Y	Y						Y										
7881	A_23_P360911	MLH3			Y	Y		Y	Y	Y	Y	Y	Y	Y					Y	Y		Y	Y				Y	Y	Y	Y																
11097	A_23_P61050	MLKL	Y	Y	Y		Y		Y	Y	Y	Y	Y	Y													Y			Y						Y										
13620	A_23_P64217	MLL			Y	Y		Y	Y	Y																																				
3088	A_23_P13711	MLL2			Y				Y	Y										Y			Y							Y						Y										
11416	A_23_P168419	MLL3																										Y																		
7830	A_23_P142421	MLL4			Y													Y	Y	Y	Y	Y	Y	Y	Y	Y	Y				Y															
4384	A_23_P215580	MLL5			Y	Y	Y	Y	Y	Y	Y	Y						Y	Y	Y		Y		Y			Y	Y	Y	Y																
17839	A_23_P153827	MLLT1																		Y		Y																								
3968	A_23_P149912	MLLT10																Y	Y	Y				Y																						
17975	A_23_P23346	MLLT11							Y	Y									Y											Y						Y										
12942	A_23_P216693	MLLT3			Y	Y		Y		Y													Y					Y																		
3953	A_23_P256603	MLLT4			Y																	Y																								
772	A_23_P410653	MLLT6			Y	Y		Y		Y									Y	Y		Y	Y				Y	Y	Y	Y																
1423	A_23_P106985	MLLT6																	Y				Y																							
13960	A_23_P154400	MLPH			Y				Y	Y	Y	Y	Y	Y																Y						Y										
21329	A_23_P164148	MLX			Y	Y		Y	Y	Y	Y	Y	Y	Y				Y	Y	Y	Y	Y	Y	Y	Y	Y	Y	Y	Y	Y	Y	Y				Y										
4174	A_23_P9823	MLXIP	Y		Y													Y	Y	Y		Y	Y	Y			Y	Y	Y	Y																
13059	A_23_P33027	MLXIP			Y	Y		Y	Y	Y	Y	Y	Y	Y				Y	Y	Y		Y	Y	Y			Y	Y	Y	Y						Y										
15441	A_23_P88810	MLYCD				Y													Y									Y																		
16256	A_23_P2537	MMAB			Y				Y	Y	Y	Y	Y	Y																																
12891	A_23_P94932	MMADHC																					Y																							
10372	A_23_P164047	MMD																			Y	Y																								
13900	A_23_P212061	MME							Y	Y								Y	Y																											
13546	A_23_P138294	MMEL1			Y	Y		Y		Y																	Y																			
9896	A_23_P11224	MMGT1			Y													Y																												
6992	A_23_P1691	MMP1			Y																						Y																			
3676	A_23_P13096	MMP10																			Y																									
9787	A_23_P150316	MMP12	Y	Y	Y	Y		Y	Y	Y																				Y						Y										
16904	A_23_P340698	MMP12	Y	Y	Y	Y		Y	Y	Y																		Y		Y						Y										
10917	A_23_P205509	MMP14																		Y																										
8123	A_23_P100177	MMP15			Y	Y		Y		Y								Y	Y	Y		Y	Y	Y			Y																			
1877	A_23_P123448	MMP16			Y				Y	Y									Y								Y			Y						Y										
8233	A_23_P136413	MMP17																					Y																							
9702	A_23_P163787	MMP2			Y															Y		Y																								
10147	A_23_P74088	MMP23B																		Y			Y																							
2850	A_23_P2052	MMP26																												Y						Y										
3838	A_23_P35899	MMP27			Y																						Y																			
21090	A_23_P52761	MMP7	Y	Y	Y																						Y																			
12495	A_23_P40174	MMP9				Y							Y								Y							Y		Y						Y										
6084	A_23_P18535	MMRN1			Y				Y	Y									Y			Y					Y																			
4320	A_23_P150057	MMRN2			Y				Y	Y	Y	Y	Y	Y				Y	Y	Y			Y	Y			Y																			
9806	A_23_P6381	MN1				Y			Y	Y																				Y						Y										
6040	A_23_P32615	MNAT1			Y	Y		Y	Y	Y	Y	Y	Y	Y						Y							Y																			
10861	A_23_P99700	MNAT1			Y	Y	Y	Y	Y	Y	Y	Y																																		
5158	A_23_P133123	MND1	Y	Y	Y													Y									Y																			
16087	A_23_P137935	MNDA	Y	Y	Y																						Y																			
16573	A_23_P3302	MNS1				Y														Y			Y					Y																		
10675	A_23_P411072	MNT																									Y																			
20122	A_23_P218320	MNT																		Y			Y																							
18137	A_23_P253331	MNX1																	Y	Y																										
22202	A_23_P205389	MOAP1			Y	Y		Y		Y																	Y	Y	Y	Y																
19485	A_23_P347059	MOBKL1A			Y	Y	Y	Y	Y	Y	Y	Y						Y	Y	Y		Y	Y	Y			Y																			
7725	A_23_P108922	MOBKL1B			Y															Y			Y																							
20430	A_23_P146548	MOBKL2B							Y	Y	Y	Y	Y	Y													Y			Y						Y										
8651	A_23_P109614	MOBP							Y	Y												Y																								
C12387.4	A_23_P67042	MOCOS																		Y	Y		Y																							
17177	A_23_P58993	MOCS1																		Y		Y																								
22028	A_23_P92967	MOCS2							Y	Y										Y																										
13355	A_23_P28811	MOCS3																Y																												
247	A_23_P133878	MOG			Y																	Y					Y			Y						Y										
13964	A_23_P317796	MOGAT3				Y																																								
5273	A_23_P41075	MON1A				Y													Y																											

4954	A_23_P206400	MON1B																					Y																							
17500	A_23_P155027	MORC2																										Y																		
11909	A_23_P37574	MORF4L1			Y																						Y			Y						Y										
18759	A_23_P114401	MORF4L2																									Y																			
19181	A_23_P160129	MORN1			Y															Y			Y																							
13907	A_23_P98941	MORN3																		Y																										
15387	A_23_P332340	MORN3																Y												Y						Y										
12541	A_23_P20783	MORN5			Y																						Y																			
14911	A_23_P200685	MOSC2			Y	Y		Y	Y	Y	Y	Y	Y	Y						Y			Y				Y	Y	Y	Y																
11981	A_23_P406986	MOSPD2				Y														Y																										
6516	A_23_P161125	MOV10			Y													Y	Y	Y		Y	Y	Y			Y																			
1519	A_23_P143774	MOV10L1																												Y						Y										
2152	A_23_P26795	MPDU1																		Y			Y																							
21960	A_23_P71598	MPDZ			Y				Y	Y									Y	Y							Y			Y						Y										
15273	A_23_P140844	MPHOSPH6			Y	Y		Y		Y																																				
7870	A_23_P13657	MPHOSPH9			Y	Y		Y	Y	Y	Y	Y	Y	Y														Y																		
16720	A_23_P60585	MPI			Y	Y		Y		Y								Y		Y	Y	Y																								
12328	A_23_P130886	MPND			Y		Y		Y	Y	Y	Y	Y					Y		Y	Y	Y					Y			Y						Y										
13824	A_23_P141173	MPO																Y	Y																											
15142	A_23_P171296	MPP1	Y												Y																															
9819	A_23_P394255	MPP2			Y																																									
16342	A_23_P207325	MPP2																Y																												
4617	A_23_P141346	MPP3			Y				Y	Y										Y							Y			Y						Y										
2355	A_23_P131263	MPP4																Y																												
8657	A_23_P71053	MPP6							Y	Y													Y							Y						Y										
8126	A_23_P384663	MPP7	Y	Y					Y	Y			Y																	Y						Y										
22103	A_23_P52198	MPP7																					Y																							
8471	A_23_P146855	MPPED1			Y																						Y			Y						Y										
19264	A_23_P15342	MPRIP																		Y			Y				Y																			
8577	A_23_P132285	MPST			Y	Y	Y	Y	Y	Y	Y	Y						Y		Y		Y					Y			Y						Y										
8517	A_23_P143084	MPV17			Y	Y		Y		Y								Y	Y	Y	Y	Y		Y			Y			Y						Y										
6174	A_23_P138089	MPZ				Y													Y				Y					Y																		
19779	A_23_P11874	MPZL1			Y	Y		Y		Y																	Y	Y	Y	Y						Y										
1056	A_23_P150379	MPZL2				Y																						Y		Y						Y										
18237	A_23_P431939	MR1							Y	Y																																				
22299	A_23_P201837	MR1				Y												Y																												
11141	A_23_P41188	MRAS	Y	Y		Y			Y	Y	Y	Y	Y	Y														Y		Y						Y										
13745	A_23_P377197	MRAS			Y	Y		Y	Y	Y	Y	Y	Y	Y						Y		Y	Y				Y	Y	Y	Y						Y										
543	A_23_P12746	MRC1L1	Y	Y	Y																																									
2024	A_23_P49632	MRC2			Y	Y		Y		Y										Y							Y																			
3708	A_23_P150189	MRE11A				Y													Y	Y			Y					Y																		
7701	A_23_P108948	MREG							Y	Y	Y	Y	Y	Y													Y			Y						Y										
9490	A_23_P144445	MRFAP1			Y		Y		Y	Y	Y	Y	Y	Y													Y			Y						Y										
16705	A_23_P133058	MRFAP1L1			Y	Y		Y		Y													Y				Y																			
5992	A_23_P127352	MRGPRD			Y	Y		Y		Y																																				
13442	A_23_P432573	MRGPRF			Y															Y																										
12124	A_23_P389379	MRGPRX3			Y													Y		Y		Y																								
17978	A_23_P141813	MRLC2				Y			Y	Y																																				
20496	A_23_P15603	MRM1																Y	Y	Y		Y		Y																						
20922	A_23_P4546	MRO			Y										Y			Y												Y						Y										
16984	A_23_P116096	MRPL11			Y				Y	Y	Y	Y	Y	Y													Y			Y						Y										
17151	A_23_P10346	MRPL12			Y	Y		Y	Y	Y	Y	Y	Y	Y				Y	Y	Y		Y	Y	Y			Y	Y	Y	Y																
20966	A_23_P170352	MRPL12			Y	Y		Y	Y	Y								Y	Y	Y		Y	Y	Y			Y	Y	Y	Y																
19796	A_23_P94174	MRPL15	Y	Y	Y	Y		Y	Y	Y																	Y																			
249	A_23_P47497	MRPL16			Y	Y		Y		Y																																				
19401	A_23_P102262	MRPL19																					Y																							
18213	A_23_P7941	MRPL2			Y	Y	Y	Y	Y	Y	Y	Y	Y	Y				Y		Y							Y	Y	Y	Y						Y										
19413	A_23_P97473	MRPL20			Y													Y	Y	Y		Y		Y			Y																			
20175	A_23_P105044	MRPL23																												Y						Y										
3316	A_23_P129513	MRPL28			Y	Y		Y		Y								Y	Y	Y		Y	Y	Y																						
14307	A_23_P7030	MRPL3			Y	Y		Y		Y																																				
11926	A_23_P218619	MRPL30			Y	Y		Y		Y																	Y	Y	Y	Y																
17699	A_23_P256540	MRPL33																												Y						Y										
12417	A_23_P5266	MRPL34			Y	Y		Y		Y								Y	Y	Y		Y		Y			Y																			
12878	A_23_P135474	MRPL37			Y	Y		Y		Y	Y	Y	Y	Y				Y	Y	Y				Y			Y	Y	Y	Y						Y										
467	A_23_P54957	MRPL38																Y	Y	Y				Y																						
1324	A_23_P164702	MRPL4			Y	Y	Y	Y	Y	Y	Y	Y								Y		Y					Y	Y	Y	Y						Y										
813	A_23_P253552	MRPL40							Y	Y																																				
8054	A_23_P346243	MRPL41							Y	Y								Y	Y	Y		Y	Y	Y				Y		Y						Y										
19713	A_23_P398491	MRPL42P5																									Y																			
19333	A_23_P63689	MRPL43			Y	Y		Y	Y	Y									Y	Y		Y					Y																			
21926	A_23_P90589	MRPL44			Y	Y		Y		Y																	Y	Y	Y	Y						Y										
15700	A_23_P113623	MRPL45																									Y																			
1630	A_23_P138819	MRPL49			Y													Y		Y		Y					Y																			
18613	A_23_P99138	MRPL51							Y	Y	Y	Y	Y	Y																Y						Y										
12149	A_23_P70418	MRPS10			Y																																									
454	A_23_P50807	MRPS12			Y	Y		Y	Y	Y									Y	Y		Y						Y																		

20985	A_23_P114822	MRPS15																									Y																			
17815	A_23_P122384	MRPS18A			Y																						Y																			
260	A_23_P59107	MRPS18B			Y																																										
20402	A_23_P135104	MRPS2																Y	Y	Y		Y		Y			Y																			
10715	A_23_P106998	MRPS23			Y	Y		Y		Y																																			
16709	A_23_P42884	MRPS24			Y																						Y																			
19611	A_23_P18135	MRPS25			Y																																										
14443	A_23_P252536	MRPS26							Y	Y											Y						Y			Y						Y											
8497	A_23_P133332	MRPS27			Y	Y		Y	Y	Y	Y	Y	Y	Y													Y	Y	Y	Y						Y											
11537	A_23_P252369	MRPS30			Y																						Y			Y						Y											
9785	A_23_P162807	MRPS31																Y		Y	Y	Y																									
13929	A_23_P157352	MRPS33			Y	Y		Y		Y																Y			Y						Y											
19656	A_23_P163496	MRPS34							Y	Y																				Y						Y											
12226	A_23_P25073	MRPS35									Y		Y	Y																															
2443	A_23_P29575	MRPS36							Y	Y	Y	Y	Y	Y				Y		Y							Y			Y						Y											
1410	A_23_P28533	MRPS5							Y	Y								Y												Y						Y											
16330	A_23_P102890	MRPS6	Y		Y	Y		Y		Y					Y			Y	Y								Y																			
21163	A_23_P3973	MRPS7	Y	Y																Y																									
5329	A_23_P111373	MRS2																					Y																						
1087	A_23_P127760	MS4A12			Y																						Y																			
9550	A_23_P98561	MS4A14				Y																						Y																		
20364	A_23_P1904	MS4A2							Y	Y																																				
4282	A_23_P161914	MS4A3			Y																						Y																			
12587	A_23_P75769	MS4A4A	Y	Y	Y	Y		Y	Y	Y					Y			Y	Y	Y		Y		Y			Y	Y	Y	Y						Y											
16729	A_23_P203376	MS4A6A	Y	Y	Y				Y	Y										Y		Y	Y				Y	Y	Y	Y																	
16147	A_23_P416234	MS4A6E																Y		Y			Y																						
8154	A_23_P203351	MS4A7	Y	Y	Y										Y												Y																			
21844	A_23_P139147	MS4A8B																			Y																								
9084	A_23_P256943	MSC																		Y			Y																						
9636	A_23_P102471	MSH2							Y	Y									Y			Y																						
4702	A_23_P122001	MSH3	Y	Y	Y	Y	Y	Y	Y	Y	Y	Y	Y	Y				Y	Y	Y		Y	Y	Y			Y	Y	Y	Y						Y								Y		
19059	A_23_P93329	MSH5			Y																																										
19492	A_23_P102202	MSH6				Y			Y	Y									Y																									
22453	A_23_P369479	MSI2																					Y																						
20044	A_23_P217776	MSL3	Y	Y	Y	Y	Y	Y	Y	Y	Y	Y						Y		Y							Y	Y	Y	Y						Y											
4068	A_23_P77529	MSLN			Y	Y	Y	Y	Y	Y	Y	Y	Y	Y				Y	Y	Y		Y	Y	Y			Y	Y	Y	Y						Y											
6333	A_23_P73589	MSN	Y	Y	Y	Y	Y	Y	Y	Y	Y	Y	Y	Y						Y	Y		Y				Y			Y			Y			Y						Y				
8290	A_23_P216176	MSR1	Y												Y																														
636	A_23_P61426	MSRA																Y																											
7555	A_23_P97932	MSRB2				Y																																							
17069	A_23_P69399	MST1	Y	Y	Y																						Y	Y	Y	Y																	
2216	A_23_P165727	MSTN																												Y						Y											
2632	A_23_P51669	MSTO1																		Y		Y																							
1092	A_23_P110430	MSX1																Y		Y		Y																							
11011	A_23_P213910	MSX2							Y	Y																			Y						Y											
14464	A_23_P360232	MT1A																					Y																						
3110	A_23_P15179	MT1F			Y																		Y						Y						Y											
635	A_23_P60933	MT1G																										Y																	
13004	A_23_P392884	MT1M			Y				Y	Y								Y	Y	Y			Y	Y			Y	Y	Y	Y						Y											
1424	A_23_P303242	MT1X			Y																																										
347	A_23_P252413	MT2A							Y	Y																Y																			
1655	A_23_P315273	MT3			Y				Y	Y	Y	Y	Y	Y									Y																						
22258	A_23_P129629	MT3																		Y																									
8929	A_23_P152398	MT4																									Y			Y						Y											
21939	A_23_P158129	MTA1																			Y																								
21748	A_23_P203420	MTA2			Y														Y			Y				Y																			
11841	A_23_P154522	MTA3			Y	Y	Y	Y	Y	Y	Y	Y	Y	Y					Y	Y	Y		Y				Y																			
19422	A_23_P112368	MTAP																		Y																									
11144	A_23_P357794	MTBP							Y	Y																																				
12360	A_23_P145388	MTCH1			Y	Y		Y		Y									Y																										
21757	A_23_P64010	MTCH2			Y				Y	Y																Y																			
C12410.6	A_23_P250196	MTDH			Y	Y		Y		Y										Y							Y																			
21961	A_23_P408108	MTERF																		Y	Y																									
13851	A_23_P53530	MTERFD3			Y	Y	Y	Y	Y	Y	Y	Y	Y	Y				Y	Y	Y		Y	Y	Y			Y	Y	Y	Y						Y											
7146	A_23_P216210	MTFR1			Y	Y		Y	Y	Y																	Y	Y	Y	Y																	
6826	A_23_P113726	MTG1																					Y																						
20125	A_23_P214907	MTHFD1L	Y	Y					Y	Y	Y	Y	Y	Y																Y						Y											
2063	A_23_P163380	MTHFS			Y		Y		Y	Y	Y	Y	Y														Y			Y						Y											
15719	A_23_P40072	MTIF2				Y													Y			Y																						
12578	A_23_P53788	MTIF3																Y			Y																								
5768	A_23_P161507	MTL5			Y	Y	Y	Y	Y	Y	Y	Y	Y	Y						Y			Y				Y	Y	Y	Y						Y											
2657	A_23_P73530	MTMR1																Y																											
20990	A_23_P117833	MTMR10																										Y																	
9190	A_23_P377888	MTMR15	Y	Y	Y	Y	Y	Y	Y	Y	Y	Y	Y	Y					Y	Y		Y	Y				Y	Y	Y	Y						Y						Y				
17526	A_23_P152010	MTMR15			Y																		Y				Y																			
22236	A_23_P64018	MTMR2							Y	Y	Y	Y	Y	Y																															
1983	A_23_P84995	MTMR8			Y																								Y						Y											

12405	A_23_P71389	MTMR9																Y		Y		Y	Y																							
7292	A_23_P80987	MTNR1A			Y																						Y																			
1558	A_23_P127422	MTNR1B				Y			Y	Y										Y										Y						Y										
18632	A_23_P145067	MTO1																		Y																										
1940	A_23_P29209	MTP18																		Y		Y	Y																							
7464	A_23_P147098	MTPN			Y																																									
8155	A_23_P200930	MTR			Y	Y	Y	Y	Y	Y	Y	Y						Y	Y	Y		Y	Y	Y			Y																			
20123	A_23_P37005	MTRF1			Y	Y	Y	Y	Y	Y	Y	Y						Y		Y		Y	Y																							
10782	A_23_P214882	MTRF1L			Y	Y		Y		Y										Y			Y				Y	Y	Y	Y																
14732	A_23_P252211	MTRR				Y														Y		Y	Y																							
10373	A_23_P347632	MTSS1			Y																																									
22516	A_23_P213171	MTTP	Y															Y		Y							Y																			
3427	A_23_P94358	MTUS1																										Y		Y						Y										
18210	A_23_P137865	MTX1			Y	Y		Y	Y	Y								Y	Y	Y		Y	Y	Y			Y	Y	Y	Y																
7562	A_23_P120335	MTX2			Y	Y		Y		Y									Y	Y	Y		Y				Y																			
16659	A_23_P137856	MUC1							Y	Y	Y	Y	Y	Y							Y		Y							Y						Y										
14567	A_23_P155236	MUC13																			Y																									
6595	A_23_P24332	MUC15			Y	Y		Y		Y																																				
14422	A_23_P5211	MUC16			Y															Y																										
20700	A_23_P434369	MUC19																Y																												
9218	A_23_P170148	MUC2																	Y				Y																							
1698	A_23_P259022	MUC4			Y	Y		Y		Y																	Y	Y	Y	Y																
5634	A_23_P44818	MUC6							Y	Y																																				
4525	A_23_P121614	MUC7																		Y																										
13483	A_23_P150979	MUCL1							Y	Y	Y	Y	Y	Y													Y			Y						Y										
7170	A_23_P37265	MUDENG							Y	Y	Y	Y	Y	Y						Y		Y	Y																							
13611	A_23_P200598	MUL1			Y	Y		Y	Y	Y								Y	Y	Y	Y	Y		Y																						
10004	A_23_P400217	MUM1			Y				Y	Y										Y							Y																			
9058	A_23_P73571	MUM1L1			Y																						Y																			
14808	A_23_P202686	MUPCDH			Y	Y		Y		Y										Y			Y				Y																			
14828	A_23_P123804	MURC				Y														Y			Y																							
7986	A_23_P161634	MUS81																			Y																									
10242	A_23_P400235	MUT				Y	Y		Y	Y	Y	Y	Y							Y								Y		Y						Y										
519	A_23_P51656	MUTYH																	Y	Y																										
22391	A_23_P152125	MVD			Y	Y	Y	Y	Y	Y	Y	Y	Y	Y				Y	Y	Y	Y	Y	Y	Y	Y	Y	Y	Y	Y	Y	Y	Y				Y										
17938	A_23_P105592	MVK																Y	Y	Y		Y		Y			Y																			
18248	A_23_P409875	MVK																Y	Y	Y		Y		Y			Y																			
14494	A_23_P6263	MX2																Y																												
698	A_23_P124552	MXD3																		Y	Y		Y																							
15432	A_23_P259490	MXD4			Y	Y	Y	Y	Y	Y	Y	Y						Y	Y	Y		Y	Y	Y			Y	Y	Y	Y						Y										
16532	A_23_P161399	MXI1							Y	Y																																				
4251	A_23_P258132	MXRA5				Y													Y	Y	Y							Y																		
1902	A_23_P32444	MXRA8																		Y																										
11576	A_23_P27584	MYADM																		Y		Y	Y																							
20201	A_23_P16262	MYBPC2			Y	Y		Y		Y									Y								Y	Y	Y	Y						Y										
14171	A_23_P127385	MYBPC3																			Y																									
15027	A_23_P148737	MYBPH																									Y																			
7373	A_23_P52006	MYBPHL			Y	Y		Y		Y												Y																								
6898	A_23_P201655	MYCBP							Y	Y										Y	Y	Y	Y																							
18763	A_23_P151459	MYCBP2																		Y		Y																								
8051	A_23_P141415	MYCBPAP			Y	Y	Y	Y	Y	Y	Y	Y	Y	Y						Y		Y	Y				Y	Y	Y	Y			Y			Y										
9216	A_23_P67952	MYCNOS																					Y																							
3757	A_23_P31046	MYCT1			Y													Y	Y																											
737	A_23_P362659	MYD88	Y		Y	Y	Y	Y	Y	Y	Y	Y						Y	Y	Y	Y	Y	Y	Y	Y	Y	Y	Y	Y	Y	Y	Y				Y										
20341	A_23_P77079	MYEF2			Y	Y	Y	Y	Y	Y	Y	Y	Y	Y						Y			Y				Y	Y	Y	Y						Y										
19027	A_23_P87860	MYF5																Y																												
18677	A_23_P164081	MYH10				Y			Y	Y																																				
6300	A_23_P206920	MYH11																					Y																							
14815	A_23_P78734	MYH14			Y	Y		Y		Y										Y	Y	Y	Y				Y																			
486	A_23_P26863	MYH3			Y	Y		Y	Y	Y																	Y																			
11471	A_23_P37168	MYH6	Y	Y	Y																						Y																			
7665	A_23_P77931	MYH8																		Y																										
20074	A_23_P57497	MYH9			Y	Y	Y	Y	Y	Y	Y	Y	Y					Y	Y	Y	Y	Y	Y	Y	Y	Y	Y			Y	Y	Y	Y			Y								Y		
2464	A_23_P162547	MYL2			Y																						Y																			
11183	A_23_P49905	MYL4			Y																						Y																			
1637	A_23_P344973	MYL6			Y	Y		Y		Y										Y																										
6867	A_23_P203864	MYL6			Y	Y		Y	Y	Y																																				
2637	A_23_P2223	MYL6B			Y	Y		Y		Y								Y			Y									Y						Y										
7622	A_23_P210428	MYL9																		Y			Y																							
13381	A_23_P393015	MYLC2PL	Y		Y													Y									Y																			
6264	A_23_P31034	MYLIP	Y	Y		Y			Y	Y	Y	Y	Y	Y																Y						Y										
11092	A_23_P54755	MYLK3			Y	Y		Y	Y	Y										Y		Y	Y				Y			Y						Y										
16495	A_23_P258606	MYLPF																Y																												
2458	A_23_P27229	MYO15A																												Y						Y										
16285	A_23_P106952	MYO15B							Y	Y													Y				Y			Y						Y										
19353	A_23_P78122	MYO18A			Y				Y	Y										Y		Y																								
4464	A_23_P416656	MYO1C			Y	Y		Y		Y								Y	Y	Y		Y	Y	Y			Y																			

13662	A_23_P49828	MYO1D	Y	Y	Y	Y		Y		Y								Y	Y	Y		Y		Y			Y				Y		Y	Y	Y											
21652	A_23_P37497	MYO1E							Y	Y	Y	Y	Y	Y																																
14175	A_23_P142447	MYO1F	Y	Y	Y	Y	Y	Y		Y	Y	Y							Y	Y			Y				Y	Y	Y	Y																
5708	A_23_P257543	MYO1G			Y	Y	Y	Y	Y	Y	Y	Y	Y	Y					Y	Y		Y	Y				Y	Y	Y	Y						Y										
14531	A_23_P44600	MYO3B																	Y																											
2631	A_23_P117656	MYO5A			Y																Y																									
18937	A_23_P140434	MYO5C	Y	Y	Y	Y		Y	Y	Y	Y	Y	Y	Y				Y	Y	Y		Y	Y	Y			Y			Y						Y										
20787	A_23_P255952	MYO6			Y	Y	Y	Y	Y	Y	Y	Y	Y	Y				Y	Y	Y	Y	Y	Y	Y	Y	Y	Y	Y	Y	Y	Y	Y				Y										
12605	A_23_P98780	MYO7A			Y				Y	Y																																				
7149	A_23_P209799	MYO7B																Y	Y																											
16262	A_23_P205841	MYO9A			Y	Y		Y	Y	Y										Y		Y					Y			Y						Y										
20713	A_23_P101915	MYO9B	Y	Y	Y	Y	Y	Y	Y	Y	Y	Y	Y	Y				Y	Y	Y		Y		Y			Y	Y	Y	Y						Y										
18633	A_23_P53033	MYOD1			Y																		Y																							
10227	A_23_P86682	MYOF			Y	Y		Y		Y	Y	Y	Y	Y				Y		Y							Y	Y	Y	Y						Y										
6083	A_23_P258912	MYOM2																	Y																											
13796	A_23_P104438	MYPN																		Y																										
4578	A_23_P84032	MYRIP																	Y			Y																								
19221	A_23_P326760	MYRIP			Y	Y	Y	Y	Y	Y	Y	Y	Y	Y													Y	Y	Y	Y						Y										
4962	A_23_P100472	MYST1	Y	Y	Y	Y		Y	Y	Y																	Y																			
12098	A_23_P407628	MYST3			Y	Y		Y	Y	Y	Y	Y	Y	Y														Y																		
1476	A_23_P66081	N4BP1			Y	Y	Y	Y	Y	Y	Y	Y	Y	Y						Y	Y						Y																			
21616	A_23_P432610	N4BP1			Y				Y	Y																	Y																			
6299	A_23_P76659	N4BP2L1			Y	Y		Y		Y								Y		Y							Y																			
5909	A_23_P53856	N4BP2L2			Y															Y							Y																			
14894	A_23_P65262	N4BP2L2			Y																						Y			Y						Y										
10126	A_23_P58747	N4BP3							Y	Y																				Y						Y										
11765	A_23_P80086	N6AMT1			Y																						Y																			
17570	A_23_P151368	N6AMT2																			Y																									
5021	A_23_P155666	NAAA			Y	Y		Y	Y	Y	Y	Y	Y	Y													Y																			
10051	A_23_P52668	NAALADL1																		Y			Y																							
7257	A_23_P209805	NAB1							Y	Y	Y	Y	Y	Y																																
2026	A_23_P368187	NAB2																	Y	Y		Y	Y																							
9150	A_23_P76151	NAB2																		Y	Y		Y																							
15501	A_23_P162228	NACA			Y	Y		Y	Y	Y									Y	Y		Y					Y	Y	Y	Y						Y										
21402	A_23_P59889	NACAP1			Y	Y	Y	Y	Y	Y	Y	Y						Y	Y	Y				Y			Y	Y	Y	Y																
4562	A_23_P113825	NACC2			Y	Y		Y	Y	Y	Y	Y	Y	Y						Y								Y		Y						Y										
13930	A_23_P200056	NADK			Y	Y		Y		Y										Y		Y																								
4886	A_23_P154208	NAGK			Y	Y	Y	Y	Y	Y	Y	Y							Y	Y	Y	Y	Y				Y	Y	Y	Y						Y										
21028	A_23_P26945	NAGLU			Y	Y	Y	Y	Y	Y	Y	Y						Y	Y	Y	Y	Y	Y	Y	Y	Y	Y				Y															
7662	A_23_P38041	NAGPA																	Y	Y	Y	Y	Y																							
12877	A_23_P253803	NAIF1							Y	Y																																				
2647	A_23_P110473	NAIP			Y	Y	Y	Y	Y	Y	Y	Y							Y	Y		Y					Y	Y	Y	Y						Y										
7139	A_23_P305060	NAMPT			Y																																									
272	A_23_P427039	NANP																												Y						Y										
9584	A_23_P53646	NAP1L1				Y																																								
14506	A_23_P65047	NAP1L1				Y													Y	Y																										
3901	A_23_P125705	NAP1L2							Y	Y																	Y	Y	Y	Y						Y										
11666	A_23_P125717	NAP1L3			Y	Y		Y		Y									Y								Y	Y	Y	Y						Y										
19556	A_23_P36215	NAP1L4			Y													Y		Y		Y					Y																			
1411	A_23_P144369	NAP1L5			Y		Y		Y	Y	Y	Y						Y	Y	Y		Y		Y			Y			Y						Y										
6631	A_23_P55990	NAPA			Y																																									
20855	A_23_P109193	NAPB				Y																																								
15555	A_23_P141764	NAPG																										Y																		
22442	A_23_P258312	NAPRT1																	Y																											
19767	A_23_P90130	NAPSA			Y														Y	Y			Y				Y																			
20344	A_23_P90125	NAPSB	Y		Y													Y	Y	Y			Y	Y			Y																			
15364	A_23_P218317	NARF	Y	Y	Y	Y		Y	Y	Y	Y	Y	Y	Y				Y		Y			Y				Y	Y	Y	Y																
15553	A_23_P37877	NARFL				Y														Y		Y																								
6629	A_23_P212825	NARG1																									Y																			
3133	A_23_P14204	NARG1L																Y																												
15068	A_23_P251786	NARG1L			Y	Y	Y	Y	Y	Y	Y	Y	Y	Y				Y	Y	Y		Y		Y			Y	Y	Y	Y						Y										
4837	A_23_P48920	NARG2				Y																																								
20219	A_23_P54389	NARG2			Y	Y		Y	Y	Y	Y	Y	Y	Y						Y		Y					Y	Y	Y	Y						Y										
19987	A_23_P101185	NARS							Y	Y									Y	Y		Y																								
19120	A_23_P24960	NARS2																		Y			Y										Y													
13802	A_23_P95596	NAT1	Y	Y	Y	Y	Y	Y	Y	Y	Y	Y	Y	Y				Y	Y	Y	Y		Y	Y	Y		Y	Y	Y	Y			Y			Y								Y		
11589	A_23_P87329	NAT10																	Y	Y		Y	Y																							
20784	A_23_P25868	NAT12			Y	Y		Y		Y									Y	Y		Y	Y																							
21663	A_23_P121122	NAT13																					Y																							
11106	A_23_P50389	NAT14																		Y		Y																								
13316	A_23_P89113	NAT15			Y													Y	Y	Y		Y	Y	Y			Y																			
15402	A_23_P31798	NAT2	Y	Y	Y	Y	Y	Y	Y	Y	Y	Y	Y	Y				Y	Y	Y	Y	Y	Y	Y	Y	Y	Y	Y	Y	Y	Y	Y	Y	Y	Y	Y										
13235	A_23_P102770	NAT5																		Y			Y																							
7899	A_23_P212400	NAT6	Y	Y	Y																																									
8890	A_23_P154379	NAT8			Y	Y		Y		Y																	Y																			
20348	A_23_P165764	NAT8B			Y	Y		Y	Y	Y																		Y																		
1481	A_23_P66421	NAT9			Y	Y		Y	Y	Y								Y	Y	Y			Y	Y			Y	Y	Y	Y																

2869	A_23_P52727	NAV2	Y		Y	Y	Y	Y		Y	Y	Y			Y			Y	Y	Y		Y	Y	Y			Y	Y	Y	Y																
11911	A_23_P393639	NAV2			Y	Y		Y		Y																	Y	Y	Y	Y						Y										
5608	A_23_P13740	NAV3	Y	Y	Y	Y		Y	Y	Y								Y	Y	Y				Y			Y	Y	Y	Y						Y										
240	A_23_P21128	NBEA	Y	Y	Y	Y	Y	Y	Y	Y	Y	Y	Y	Y						Y	Y	Y					Y	Y	Y	Y						Y										
8584	A_23_P65278	NBEA	Y	Y	Y	Y	Y	Y	Y	Y	Y	Y	Y	Y				Y		Y	Y	Y					Y	Y	Y	Y						Y										
21481	A_23_P51958	NBL1																		Y																										
14089	A_23_P133000	NBLA00301																Y				Y																								
21761	A_23_P103454	NBPF3			Y	Y	Y	Y	Y	Y	Y	Y								Y			Y					Y														Y				
15762	A_23_P399250	NBPF4	Y	Y	Y	Y	Y	Y	Y	Y	Y	Y			Y			Y	Y	Y		Y	Y	Y			Y	Y	Y	Y	Y	Y	Y	Y	Y	Y		Y				Y			Y	
13441	A_23_P207399	NBR1	Y	Y	Y	Y	Y	Y	Y	Y	Y	Y						Y	Y	Y		Y	Y	Y			Y	Y	Y	Y						Y						Y				
17326	A_23_P215883	NCALD							Y	Y											Y						Y			Y						Y										
17663	A_23_P211028	NCAM2			Y	Y		Y	Y	Y																		Y		Y						Y										
8856	A_23_P25293	NCAPD2																Y	Y	Y		Y	Y	Y																						
20741	A_23_P155815	NCAPG	Y	Y																																										
18468	A_23_P168747	NCAPG2																					Y																							
7308	A_23_P258754	NCAPH	Y	Y	Y													Y									Y	Y	Y	Y																
9414	A_23_P80398	NCAPH2			Y	Y		Y		Y								Y	Y	Y		Y	Y	Y			Y	Y	Y	Y						Y										
458	A_23_P257104	NCBP1			Y				Y	Y										Y																										
8993	A_23_P42746	NCF1	Y	Y	Y	Y	Y	Y	Y	Y	Y	Y	Y	Y				Y	Y	Y		Y	Y	Y			Y	Y	Y	Y	Y	Y	Y	Y	Y	Y										
8843	A_23_P138194	NCF2	Y	Y	Y																																									
10201	A_23_P109508	NCF4	Y	Y	Y	Y	Y	Y	Y	Y	Y	Y	Y	Y	Y			Y	Y	Y	Y	Y	Y	Y	Y	Y	Y	Y	Y	Y	Y	Y	Y	Y	Y					Y						
8055	A_23_P255785	NCK1	Y	Y																																										
3851	A_23_P50962	NCK2			Y	Y		Y	Y	Y								Y	Y	Y		Y		Y			Y			Y						Y										
8602	A_23_P73239	NCKAP1																		Y	Y		Y																							
19247	A_23_P128195	NCKAP1L							Y	Y																				Y						Y										
18584	A_23_P29634	NCKIPSD	Y		Y																																									
15145	A_23_P130894	NCLN			Y	Y	Y	Y	Y	Y	Y	Y	Y	Y				Y	Y	Y	Y	Y	Y	Y	Y	Y	Y	Y	Y	Y	Y	Y	Y	Y	Y	Y										
18770	A_23_P39594	NCOA1			Y	Y		Y		Y								Y	Y	Y		Y	Y	Y				Y																		
18261	A_23_P120442	NCOA3			Y	Y		Y		Y								Y		Y							Y																			
13658	A_23_P315843	NCOA5			Y	Y		Y		Y									Y	Y	Y	Y					Y	Y	Y	Y						Y										
4764	A_23_P259135	NCOA6			Y	Y	Y	Y	Y	Y	Y	Y															Y			Y						Y										
9228	A_23_P156957	NCOA7			Y																									Y						Y										
3964	A_23_P141645	NCOR1	Y	Y	Y	Y		Y		Y								Y	Y	Y	Y	Y	Y	Y	Y	Y	Y				Y		Y	Y	Y											
2380	A_23_P203891	NCOR2			Y	Y		Y	Y	Y																	Y	Y	Y	Y						Y										
9446	A_23_P108042	NCR1																		Y			Y																							
6628	A_23_P251881	NCR3			Y																						Y																			
22136	A_23_P27392	NCRNA00085			Y	Y		Y		Y										Y							Y	Y	Y	Y						Y										
11292	A_23_P19245	NCRNA00120							Y	Y									Y	Y	Y		Y				Y						Y													
5885	A_23_P154740	NCRNA00153			Y	Y	Y	Y	Y	Y	Y	Y						Y	Y	Y		Y	Y	Y			Y	Y	Y	Y						Y										
21076	A_23_P377839	NCRNA00161																												Y						Y										
13854	A_23_P321497	NCRNA00164			Y																																									
228	A_23_P34402	NCSTN			Y															Y																										
11565	A_23_P431851	ND2																Y																												
5004	A_23_P317055	ND6							Y	Y																																				
10215	A_23_P206901	NDE1			Y				Y	Y																				Y						Y										
17727	A_23_P26876	NDEL1			Y	Y		Y	Y	Y										Y										Y						Y										
440	A_23_P81247	NDFIP1			Y	Y		Y		Y																	Y																			
21233	A_23_P106405	NDN																		Y			Y																							
15622	A_23_P257407	NDOR1			Y	Y		Y		Y										Y		Y	Y				Y																			
12959	A_23_P20494	NDRG1			Y	Y	Y	Y	Y	Y	Y	Y	Y	Y						Y	Y	Y	Y				Y	Y	Y	Y						Y										
1636	A_23_P140748	NDRG4																		Y																										
1776	A_23_P30429	NDST1			Y																																									
17494	A_23_P159833	NDUFA1																		Y							Y																			
2111	A_23_P320185	NDUFA11			Y	Y	Y	Y	Y	Y	Y	Y								Y							Y	Y	Y	Y																
21699	A_23_P119622	NDUFA13			Y	Y		Y		Y										Y							Y	Y	Y	Y						Y										
11918	A_23_P257795	NDUFA2			Y				Y	Y			Y									Y					Y																			
5813	A_23_P145777	NDUFA4																		Y	Y																									
8565	A_23_P9615	NDUFA4L2			Y				Y	Y																	Y																			
1396	A_23_P215751	NDUFA5				Y														Y								Y																		
19799	A_23_P60675	NDUFA5				Y														Y																										
304	A_23_P91769	NDUFA6			Y				Y	Y																	Y																			
17520	A_23_P108303	NDUFA7																					Y																							
12482	A_23_P43566	NDUFA8				Y													Y																											
3329	A_23_P140423	NDUFB1							Y	Y																				Y						Y										
10995	A_23_P22765	NDUFB11			Y																																									
12329	A_23_P82600	NDUFB2			Y	Y		Y	Y	Y																		Y																		
20911	A_23_P51009	NDUFB3																		Y		Y	Y																							
4913	A_23_P69468	NDUFB4																		Y			Y				Y																			
5250	A_23_P43613	NDUFB6																		Y			Y																							
13110	A_23_P50872	NDUFB7			Y	Y	Y	Y	Y	Y	Y	Y	Y	Y				Y	Y	Y	Y	Y	Y	Y	Y	Y	Y	Y	Y	Y	Y	Y				Y				Y						
14527	A_23_P24164	NDUFB8			Y	Y		Y		Y																		Y																		
7096	A_23_P157669	NDUFB9			Y	Y		Y	Y	Y										Y							Y	Y	Y	Y						Y										
6498	A_23_P2152	NDUFC2							Y	Y																																				
3740	A_23_P131365	NDUFS1																		Y			Y				Y																			
22368	A_23_P149470	NDUFS2				Y																						Y																		
7163	A_23_P75398	NDUFS3			Y														Y	Y	Y	Y					Y																			
6052	A_23_P257198	NDUFS4			Y	Y		Y	Y	Y									Y	Y			Y				Y			Y						Y										

6837	A_23_P10463	NDUFS5				Y														Y																									
17435	A_23_P122228	NDUFS6			Y																						Y																		
11201	A_23_P165090	NDUFS7			Y	Y		Y	Y	Y			Y							Y		Y					Y																		
12216	A_23_P86774	NDUFS8			Y	Y		Y		Y										Y							Y																		
1159	A_23_P127353	NDUFV1			Y															Y							Y																		
838	A_23_P345793	NECAB1			Y				Y	Y																																			
18664	A_23_P68628	NECAB3																												Y						Y										
8366	A_23_P13840	NECAP1																					Y																							
15089	A_23_P2409	NEDD1			Y	Y	Y	Y	Y	Y	Y	Y						Y	Y	Y		Y	Y	Y			Y	Y	Y	Y							Y										
16231	A_23_P356624	NEDD1			Y	Y	Y	Y		Y	Y	Y						Y	Y	Y		Y	Y	Y			Y																			
18683	A_23_P95287	NEDD4																	Y																											
19064	A_23_P27273	NEDD4L			Y	Y		Y		Y								Y	Y	Y		Y	Y	Y			Y																			
2266	A_23_P88420	NEDD8																Y									Y																			
17805	A_23_P42414	NEDD9																		Y							Y																			
7494	A_23_P300600	NEFH							Y	Y																				Y						Y										
757	A_23_P216236	NEFM																		Y			Y																							
19342	A_23_P129157	NEIL1	Y	Y	Y													Y				Y					Y																			
1101	A_23_P94121	NEIL2			Y						Y		Y	Y				Y		Y							Y																			
15245	A_23_P155711	NEIL3																		Y			Y																							
16263	A_23_P124427	NEK1			Y	Y	Y	Y	Y	Y	Y	Y	Y	Y					Y	Y			Y				Y	Y	Y	Y							Y										
16380	A_23_P259801	NEK1			Y	Y		Y	Y	Y									Y	Y			Y				Y	Y	Y	Y							Y										
14079	A_23_P337270	NEK10	Y	Y	Y	Y		Y	Y	Y					Y												Y	Y	Y	Y							Y										
19574	A_23_P211973	NEK11			Y	Y		Y		Y																																				
13547	A_23_P76718	NEK3			Y	Y	Y	Y		Y	Y	Y	Y					Y	Y			Y																								
5596	A_23_P121272	NEK4	Y	Y	Y	Y	Y	Y	Y	Y	Y	Y	Y	Y					Y	Y			Y				Y	Y	Y	Y	Y	Y	Y	Y	Y	Y										
12600	A_23_P216920	NEK6																Y			Y																									
19654	A_23_P152873	NEK8																					Y																							
3514	A_23_P398509	NEK9																									Y																			
17287	A_23_P73000	NELF																		Y		Y																								
20515	A_23_P32064	NELF			Y		Y				Y		Y					Y	Y	Y	Y	Y	Y	Y	Y	Y	Y			Y	Y	Y				Y										
6507	A_23_P10025	NELL2			Y	Y		Y	Y	Y			Y					Y	Y	Y			Y	Y			Y	Y	Y	Y																
10080	A_23_P14798	NEO1			Y				Y	Y									Y	Y	Y						Y						Y													
2008	A_23_P103675	NES			Y				Y	Y								Y	Y	Y	Y	Y	Y	Y	Y	Y	Y			Y	Y	Y				Y										
8450	A_23_P83931	NET1			Y	Y		Y	Y	Y																																				
5266	A_23_P4490	NETO1																Y																												
15744	A_23_P90980	NEU2			Y																																									
911	A_23_P203713	NEU3			Y																																									
21635	A_23_P218620	NEU4			Y																						Y																			
15216	A_23_P322562	NEURL			Y																						Y																			
7382	A_23_P209484	NEUROD1																									Y																			
18031	A_23_P49579	NEUROD2			Y	Y	Y	Y		Y	Y	Y								Y			Y				Y																	Y		
12776	A_23_P30392	NEUROG1				Y																						Y																		
11367	A_23_P35534	NEUROG3																		Y		Y	Y																							
4092	A_23_P200001	NEXN																	Y									Y																		
8378	A_23_P207596	NF1																										Y		Y						Y										
11102	A_23_P338878	NF2	Y		Y	Y		Y	Y	Y	Y	Y	Y	Y						Y							Y	Y	Y	Y						Y										
5512	A_23_P6510	NFAM1			Y				Y	Y										Y		Y	Y				Y																			
19477	A_23_P3766	NFAT5																		Y		Y					Y	Y	Y	Y																
11204	A_23_P107523	NFATC1			Y	Y		Y	Y	Y										Y		Y																								
19704	A_23_P300150	NFATC1							Y	Y								Y	Y	Y				Y			Y			Y						Y										
5142	A_23_P91278	NFATC2			Y	Y	Y	Y	Y	Y	Y	Y	Y	Y				Y	Y	Y			Y	Y			Y	Y	Y	Y						Y										
19327	A_23_P125153	NFATC2IP			Y																						Y																			
13597	A_23_P77440	NFATC3																			Y																									
8554	A_23_P140394	NFATC4			Y	Y		Y		Y								Y		Y		Y	Y				Y																			
11903	A_23_P13753	NFE2			Y	Y		Y		Y																	Y			Y						Y										
14569	A_23_P78302	NFE2L1																Y		Y	Y	Y	Y																							
7159	A_23_P5761	NFE2L2			Y				Y	Y										Y							Y																			
C12028.6	A_23_P42718	NFE2L3																									Y																			
20420	A_23_P85682	NFIA	Y	Y		Y			Y	Y	Y	Y	Y	Y					Y	Y	Y							Y		Y						Y								Y		
14578	A_23_P216448	NFIB							Y	Y													Y				Y			Y						Y										
1077	A_23_P32253	NFIL3	Y	Y	Y	Y		Y	Y	Y																		Y		Y						Y										
15977	A_23_P30024	NFKB1			Y													Y	Y	Y	Y	Y		Y																						
13817	A_23_P202156	NFKB2			Y	Y	Y	Y	Y	Y	Y	Y	Y	Y					Y	Y	Y	Y	Y							Y						Y										
13599	A_23_P79086	NFKBIB			Y	Y		Y		Y								Y	Y	Y	Y	Y	Y	Y	Y	Y	Y	Y	Y	Y	Y	Y								Y						
14427	A_23_P383422	NFKBID			Y	Y		Y		Y									Y			Y	Y				Y																			
21504	A_23_P30655	NFKBIE							Y	Y	Y	Y	Y	Y																Y						Y										
5301	A_23_P145297	NFKBIL1																		Y																										
18840	A_23_P216355	NFKBIL2			Y	Y	Y	Y		Y	Y	Y						Y	Y	Y	Y	Y	Y	Y	Y	Y	Y			Y	Y	Y	Y			Y		Y				Y			Y	
10528	A_23_P212089	NFKBIZ				Y			Y	Y								Y		Y		Y						Y																		
13342	A_23_P102842	NFS1			Y																									Y						Y										
13152	A_23_P147296	NFU1			Y	Y		Y		Y								Y	Y	Y		Y	Y	Y																						
22562	A_23_P407090	NFXL1							Y	Y																																				
776	A_23_P15599	NGFR				Y																																								
185	A_23_P45524	NGFRAP1																					Y																							
3658	A_23_P140596	NGRN			Y				Y	Y																	Y																			
8764	A_23_P341567	NHEDC2																	Y																											
14875	A_23_P81092	NHEDC2				Y																					Y																			

6710	A_23_P56521	NHEJ1			Y																																									
3270	A_23_P45771	NHLH1																																												
20066	A_23_P412491	NHLH2																Y	Y	Y		Y		Y			Y			Y						Y										
3182	A_23_P46767	NHLRC2			Y	Y	Y	Y	Y	Y	Y	Y								Y							Y	Y	Y	Y						Y										
15756	A_23_P35376	NHLRC2				Y												Y	Y								Y	Y	Y	Y																
11283	A_23_P418934	NHLRC3	Y	Y	Y	Y	Y	Y		Y	Y	Y	Y	Y				Y				Y						Y		Y						Y										
2136	A_23_P80362	NHP2L1			Y	Y	Y	Y	Y	Y	Y	Y	Y	Y													Y	Y	Y	Y						Y										
21426	A_23_P110005	NICN1			Y													Y	Y	Y		Y		Y																						
20714	A_23_P200928	NID1																Y																												
6846	A_23_P163087	NID2																										Y																		
17751	A_23_P5415	NIF3L1																					Y																							
5600	A_23_P54147	NIN			Y	Y		Y	Y	Y																																				
2748	A_23_P48109	NINJ2			Y				Y	Y										Y							Y																			
2990	A_23_P100441	NIP30																	Y																											
18226	A_23_P54834	NIP7	Y	Y	Y	Y		Y	Y	Y	Y	Y	Y	Y																Y						Y										
1915	A_23_P213883	NIPBL			Y	Y		Y	Y	Y										Y							Y	Y	Y	Y						Y										
381	A_23_P120860	NIPSNAP1			Y	Y		Y		Y								Y	Y	Y		Y		Y			Y																			
4590	A_23_P376655	NIT1			Y						Y		Y	Y							Y		Y				Y																			
15138	A_23_P51376	NKAIN1			Y	Y		Y		Y																	Y			Y						Y										
12796	A_23_P170209	NKAIN2			Y													Y		Y		Y																								
22490	A_23_P316501	NKAIN2																		Y																										
18541	A_23_P17318	NKAIN4																												Y						Y										
3389	A_23_P88791	NKD1			Y													Y	Y		Y						Y																			
20508	A_23_P41813	NKD2			Y	Y	Y	Y	Y	Y	Y	Y								Y			Y				Y	Y	Y	Y						Y								Y		
4746	A_23_P119042	NKG7			Y														Y				Y				Y																			
3243	A_23_P338519	NKIRAS1																		Y																										
12696	A_23_P95397	NKIRAS1			Y															Y			Y				Y																			
1888	A_23_P10135	NKIRAS2			Y	Y	Y	Y	Y	Y	Y	Y						Y	Y	Y	Y	Y	Y	Y	Y	Y	Y			Y	Y	Y	Y			Y										
10613	A_23_P11214	NKRF																		Y																										
1387	A_23_P212002	NKTR			Y	Y	Y	Y	Y	Y	Y	Y	Y	Y					Y	Y		Y					Y	Y	Y	Y						Y										
12760	A_23_P14322	NKX2-1			Y	Y		Y		Y										Y																										
19426	A_23_P17522	NKX2-2																Y																												
19407	A_23_P25726	NKX2-8																Y		Y		Y	Y																							
20206	A_23_P341547	NKX2-8				Y																																								
11253	A_23_P157545	NKX3-1			Y	Y	Y	Y	Y	Y	Y	Y	Y	Y						Y							Y																			
8668	A_23_P255934	NKX3-2			Y	Y		Y	Y	Y										Y								Y		Y						Y										
2702	A_23_P92342	NKX6-1			Y																		Y				Y																			
245	A_23_P402804	NKX6-3																		Y			Y																							
1671	A_23_P141315	NLE1																		Y		Y																								
16290	A_23_P18123	NLGN1			Y	Y		Y		Y										Y							Y	Y	Y	Y						Y										
21124	A_23_P207125	NLGN2																		Y	Y		Y																							
10804	A_23_P62303	NLGN3																									Y																			
2506	A_23_P121437	NLGN4Y			Y														Y								Y																			
1731	A_23_P15647	NLK			Y																						Y																			
19226	A_23_P329399	NLRC3				Y																																								
10423	A_23_P119835	NLRC4	Y	Y	Y										Y			Y																												
15248	A_23_P253295	NLRC5	Y																																											
17269	A_23_P316340	NLRC5	Y	Y	Y	Y		Y	Y	Y										Y																										
19147	A_23_P402892	NLRC5			Y	Y		Y	Y	Y										Y			Y				Y	Y	Y	Y						Y										
7304	A_23_P107356	NLRP1																		Y			Y																							
19670	A_23_P89550	NLRP1																Y		Y		Y																								
138	A_23_P403488	NLRP10																Y			Y																									
20167	A_23_P35735	NLRP10				Y			Y	Y																																				
17777	A_23_P130824	NLRP2				Y													Y									Y																		
13785	A_23_P4962	NLRP5																			Y																									
2599	A_23_P501265	NLRX1																		Y																										
18636	A_23_P88522	NMB			Y	Y		Y	Y	Y	Y	Y	Y	Y					Y	Y		Y						Y		Y						Y										
7102	A_23_P70634	NMBR																Y																												
1670	A_23_P152804	NME1				Y																																								
9406	A_23_P352414	NME3	Y	Y					Y	Y	Y	Y	Y	Y																Y						Y										
16377	A_23_P152115	NME3	Y	Y	Y				Y	Y	Y	Y	Y	Y													Y			Y						Y										
19834	A_23_P88919	NME4																		Y		Y																								
16114	A_23_P156400	NME5	Y	Y	Y	Y	Y	Y	Y	Y	Y	Y	Y	Y				Y	Y	Y		Y	Y	Y			Y	Y	Y	Y						Y								Y		
8971	A_23_P259224	NME6																	Y																											
15290	A_23_P313961	NME6																	Y																											
4499	A_23_P46063	NME7			Y	Y		Y	Y	Y										Y	Y						Y			Y						Y										
C12743	A_23_P154235	NMI	Y	Y																																										
22208	A_23_P345183	NMNAT2																					Y																							
13815	A_23_P374913	NMNAT3			Y	Y		Y		Y																																				
15857	A_23_P57686	NMNAT3			Y	Y		Y		Y													Y				Y	Y	Y	Y						Y										
16896	A_23_P26649	NMRAL1			Y	Y		Y		Y								Y	Y	Y	Y	Y		Y			Y																			
21962	A_23_P107373	NMT1																										Y																		
40	A_23_P138686	NMT2																									Y																			
5665	A_23_P209700	NMUR1							Y	Y												Y						Y		Y						Y										
10971	A_23_P127584	NNMT							Y	Y								Y		Y			Y								Y		Y	Y	Y											
21735	A_23_P70148	NNT							Y	Y																																				
14361	A_23_P106822	NOB1			Y	Y	Y	Y	Y	Y	Y	Y	Y	Y				Y	Y	Y		Y	Y	Y			Y			Y						Y										

1075	A_23_P138058	NOC2L			Y	Y		Y	Y	Y								Y	Y	Y	Y	Y	Y	Y	Y	Y	Y	Y	Y	Y	Y	Y	Y	Y	Y	Y										
C16207.8	A_23_P202496	NOC3L																		Y																										
21412	A_23_P158627	NOC4L			Y	Y	Y	Y		Y	Y	Y						Y	Y	Y		Y	Y	Y			Y	Y	Y	Y						Y										
20081	A_23_P254002	NOD1																Y			Y		Y				Y						Y													
9320	A_23_P127322	NODAL																Y																												
4981	A_23_P129765	NOG			Y	Y		Y		Y								Y		Y										Y						Y										
18146	A_23_P341938	NOG																		Y																										
781	A_23_P154457	NOL10							Y	Y										Y																										
10467	A_23_P6456	NOL12			Y	Y	Y	Y	Y	Y	Y	Y	Y	Y				Y	Y	Y		Y		Y			Y	Y	Y	Y			Y			Y										
1695	A_23_P206371	NOL3	Y	Y																Y																										
2945	A_23_P71591	NOL8			Y	Y	Y	Y		Y	Y	Y						Y	Y	Y		Y		Y			Y	Y	Y	Y																
11245	A_23_P202140	NOLC1			Y	Y		Y		Y										Y		Y					Y																			
11296	A_23_P66344	NOMO1																		Y	Y																									
3836	A_23_P34033	NONO																		Y																										
9717	A_23_P54477	NOP10			Y	Y	Y	Y	Y	Y	Y	Y															Y																			
3018	A_23_P144726	NOP16																										Y																		
511	A_23_P204364	NOP2			Y	Y		Y	Y	Y								Y	Y	Y		Y	Y	Y			Y	Y	Y	Y						Y										
12281	A_23_P91328	NOP56																Y	Y		Y																									
20273	A_23_P204791	NOS1																					Y																							
11905	A_23_P74309	NOS1AP				Y																																								
319	A_23_P70849	NOS3			Y				Y	Y										Y							Y	Y	Y	Y						Y										
21444	A_23_P107959	NOSIP			Y	Y	Y	Y		Y	Y	Y								Y		Y	Y				Y	Y	Y	Y																
8648	A_23_P79360	NOSTRIN	Y	Y	Y	Y		Y	Y	Y	Y	Y	Y	Y	Y			Y	Y	Y	Y	Y	Y	Y	Y	Y	Y	Y	Y	Y	Y	Y	Y	Y	Y	Y				Y						
13742	A_23_P60393	NOTCH1			Y	Y		Y	Y	Y	Y	Y	Y	Y				Y		Y		Y	Y				Y	Y	Y	Y						Y										
15023	A_23_P200792	NOTCH2																		Y			Y				Y																			
12008	A_23_P108201	NOTCH3																					Y																							
1633	A_23_P205435	NOVA1	Y	Y	Y	Y		Y		Y											Y	Y																								
18688	A_23_P377717	NOVA2			Y	Y		Y		Y										Y		Y	Y				Y			Y						Y										
17458	A_23_P217280	NOX1							Y	Y																																				
3739	A_23_P82099	NOX3																			Y																									
15620	A_23_P47148	NOX4																				Y																								
17270	A_23_P254353	NOXA1							Y	Y																				Y						Y										
20897	A_23_P140256	NP	Y	Y	Y	Y	Y	Y	Y	Y	Y	Y	Y	Y	Y			Y	Y	Y			Y	Y			Y	Y	Y	Y						Y										
15298	A_23_P218302	N-PAC			Y														Y								Y			Y						Y										
18080	A_23_P396785	N-PAC																	Y	Y		Y																								
10531	A_23_P146111	NPAL2			Y	Y		Y		Y																	Y	Y	Y	Y																
8076	A_23_P46604	NPAL3			Y	Y		Y	Y	Y																	Y	Y	Y	Y						Y										
18218	A_23_P27734	NPAS1	Y	Y																																										
18581	A_23_P415984	NPAS2			Y	Y		Y		Y												Y																								
5903	A_23_P2942	NPAS3																												Y						Y										
889	A_23_P342787	NPAS4			Y				Y	Y																	Y																			
6162	A_23_P75494	NPAS4			Y	Y		Y		Y																	Y	Y	Y	Y						Y										
16069	A_23_P86889	NPAS4																												Y						Y										
5150	A_23_P300740	NPB			Y	Y		Y	Y	Y	Y	Y	Y	Y					Y	Y			Y					Y		Y						Y										
18266	A_23_P38545	NPB			Y	Y		Y		Y																																				
10851	A_23_P20458	NPBWR1				Y														Y			Y																							
16985	A_23_P40334	NPBWR2																		Y			Y				Y	Y	Y	Y																
19535	A_23_P107581	NPC1							Y	Y																																				
8902	A_23_P20075	NPC1L1			Y																																									
4695	A_23_P48717	NPC2			Y	Y		Y	Y	Y																																				
13649	A_23_P259362	NPCDR1			Y	Y		Y		Y										Y		Y	Y				Y																			
14903	A_23_P146565	NPDC1	Y	Y					Y	Y	Y	Y	Y	Y														Y		Y						Y										
7137	A_23_P84836	NPEPPS			Y	Y		Y		Y																	Y	Y	Y	Y						Y										
20682	A_23_P164327	NPEPPS							Y	Y	Y	Y	Y	Y				Y		Y		Y					Y																			
12250	A_23_P204079	NPFF			Y	Y		Y		Y																																				
1296	A_23_P1452	NPFFR1			Y				Y	Y																	Y																			
16173	A_23_P328652	NPHP3			Y	Y	Y	Y	Y	Y	Y	Y	Y	Y				Y	Y	Y		Y	Y	Y			Y	Y	Y	Y						Y										
22303	A_23_P35055	NPHS2																				Y																								
14248	A_23_P77744	NPIP			Y	Y		Y	Y	Y																	Y	Y	Y	Y						Y										
4257	A_23_P62820	NPL	Y		Y																																									
14502	A_23_P381431	NPL	Y	Y	Y										Y												Y																			
761	A_23_P60640	NPLOC4			Y	Y		Y		Y									Y	Y	Y	Y	Y				Y																			
1988	A_23_P72044	NPLOC4			Y	Y		Y		Y									Y	Y	Y	Y	Y				Y																			
17436	A_23_P169007	NPM2			Y															Y																										
2495	A_23_P52298	NPM3			Y				Y	Y	Y	Y	Y	Y								Y					Y			Y						Y										
1905	A_23_P253896	NPNT			Y				Y	Y																																				
17723	A_23_P74059	NPPA																												Y						Y										
3913	A_23_P147711	NPR1																												Y						Y										
1172	A_23_P253531	NPR3																Y																												
1641	A_23_P385192	NPSA			Y	Y		Y		Y									Y				Y				Y																			
7885	A_23_P124905	NPTX1			Y	Y	Y	Y		Y	Y	Y										Y					Y	Y	Y	Y						Y								Y		
13488	A_23_P82651	NPTX2																Y	Y																											
3334	A_23_P29282	NPTXR																	Y				Y																							
4725	A_23_P256470	NPY				Y																																								
269	A_23_P69699	NPY1R	Y		Y																		Y				Y			Y						Y										
8096	A_23_P251836	NPY5R																		Y		Y	Y																							
17339	A_23_P58953	NQO2			Y	Y	Y	Y		Y	Y	Y																Y																		

6114	A_23_P73632	NR0B1																Y			Y		Y																							
7631	A_23_P67330	NR1H2			Y															Y	Y	Y	Y																							
2625	A_23_P47135	NR1H3			Y																																									
1532	A_23_P25396	NR1H4	Y															Y		Y			Y																							
17275	A_23_P109788	NR1I2			Y				Y	Y																				Y						Y										
15985	A_23_P126698	NR1I3			Y																						Y																			
22093	A_23_P411284	NR2C2AP			Y	Y	Y	Y		Y	Y	Y						Y	Y	Y		Y		Y			Y	Y	Y	Y						Y										
9147	A_23_P402765	NRAP	Y		Y	Y		Y		Y																																				
C12756	A_23_P63189	NRAS	Y	Y					Y	Y	Y	Y	Y	Y	Y			Y		Y	Y	Y	Y							Y			Y			Y										
18228	A_23_P63807	NRBF2	Y	Y																										Y						Y										
16557	A_23_P165442	NRBP1			Y	Y	Y	Y	Y	Y	Y	Y						Y	Y	Y	Y	Y	Y	Y	Y	Y	Y	Y	Y	Y	Y	Y	Y							Y						
12352	A_23_P23006	NRD1			Y																																									
11559	A_23_P136493	NRG1							Y	Y								Y	Y	Y		Y	Y	Y			Y			Y						Y										
12532	A_23_P315815	NRG1							Y	Y								Y	Y	Y		Y		Y			Y			Y						Y										
12001	A_23_P213695	NRG2																Y										Y																		
4701	A_23_P116264	NRGN			Y	Y	Y	Y	Y	Y	Y	Y						Y	Y	Y		Y	Y	Y			Y																			
14405	A_23_P211007	NRIP1			Y				Y	Y																		Y		Y						Y										
1646	A_23_P13929	NRIP2			Y				Y	Y																		Y		Y						Y										
3910	A_23_P47682	NRIP3			Y														Y								Y																			
10162	A_23_P140202	NRL				Y												Y	Y	Y				Y																						
4878	A_23_P8052	NRM																		Y							Y																			
585	A_23_P82088	NRN1				Y			Y	Y									Y	Y								Y																		
5781	A_23_P86390	NRP1			Y																						Y																			
195	A_23_P429555	NRP2			Y																						Y			Y						Y										
15411	A_23_P393727	NRP2							Y	Y																																				
20946	A_23_P90363	NRTN			Y													Y	Y	Y		Y	Y	Y			Y			Y						Y										
4362	A_23_P113341	NRXN3							Y	Y																																				
9684	A_23_P251841	NRXN3			Y													Y	Y								Y																			
10508	A_23_P412427	NS3BP	Y		Y																									Y						Y										
C8472.5	A_23_P136909	NSBP1				Y	Y				Y									Y																										
9306	A_23_P171026	NSDHL			Y	Y		Y		Y								Y	Y																											
1749	A_23_P210719	NSFL1C				Y			Y	Y	Y	Y	Y	Y																Y						Y										
20340	A_23_P149336	NSL1			Y	Y	Y	Y		Y	Y	Y								Y								Y																		
17394	A_23_P134805	NSMAF							Y	Y																																				
6702	A_23_P95823	NSMCE1			Y																																									
19992	A_23_P422794	NSMCE2							Y	Y																																				
13080	A_23_P251505	NSUN5			Y	Y	Y	Y		Y	Y	Y						Y	Y	Y	Y	Y	Y	Y	Y	Y	Y				Y															
5886	A_23_P82412	NSUN5B			Y	Y	Y	Y		Y	Y	Y						Y	Y	Y		Y	Y	Y			Y																			
15614	A_23_P66439	NT5C																					Y																							
17355	A_23_P317675	NT5C1B																Y		Y																										
6774	A_23_P97906	NT5C2			Y															Y	Y						Y			Y						Y										
12007	A_23_P49919	NT5C3L							Y	Y	Y	Y	Y	Y					Y	Y		Y						Y		Y						Y										
5297	A_23_P382043	NT5DC1			Y	Y		Y	Y	Y	Y	Y	Y	Y						Y	Y	Y	Y				Y			Y						Y										
17188	A_23_P219004	NT5DC1			Y															Y	Y	Y	Y																							
626	A_23_P44836	NT5DC2			Y	Y	Y	Y	Y	Y	Y	Y						Y	Y	Y		Y	Y	Y			Y			Y						Y						Y				
1250	A_23_P36805	NT5DC3			Y				Y	Y																																				
8084	A_23_P170497	NTAN1													Y																															
7385	A_23_P96091	NTF3			Y	Y		Y	Y	Y																																				
20135	A_23_P360797	NTF3				Y			Y	Y								Y	Y	Y		Y		Y			Y	Y	Y	Y																
16022	A_23_P88901	NTHL1				Y													Y	Y		Y																								
18921	A_23_P15478	NTN1							Y	Y																	Y	Y	Y	Y						Y										
7949	A_23_P100408	NTN3			Y	Y		Y		Y																	Y																			
20917	A_23_P204630	NTN4	Y	Y	Y	Y	Y	Y	Y	Y	Y	Y	Y	Y				Y	Y	Y	Y	Y	Y	Y	Y	Y	Y	Y	Y	Y	Y	Y				Y		Y				Y			Y	Y
8461	A_23_P201544	NTNG1	Y	Y	Y	Y		Y		Y									Y									Y																		
5746	A_23_P423331	NTNG2													Y								Y																							
1657	A_23_P34804	NTRK1																	Y	Y		Y	Y				Y																			
7069	A_23_P216772	NTRK2			Y				Y	Y																	Y			Y						Y										
21111	A_23_P205900	NTRK3			Y				Y	Y																																				
4202	A_23_P36882	NTS	Y	Y	Y	Y	Y	Y	Y	Y	Y	Y	Y	Y					Y	Y		Y	Y				Y	Y	Y	Y						Y										
5064	A_23_P108979	NTSR1				Y																					Y	Y	Y	Y																
10641	A_23_P315212	NTSR2																		Y																										
15511	A_23_P348257	NUAK1																Y	Y		Y	Y																								
17373	A_23_P215157	NUB1	Y	Y																																										
20977	A_23_P118074	NUBP1																		Y		Y																								
17947	A_23_P49078	NUBP2			Y	Y		Y		Y								Y	Y	Y	Y	Y	Y	Y	Y	Y	Y			Y	Y	Y				Y										
15042	A_23_P170906	NUBPL																		Y			Y																							
11607	A_23_P78771	NUCB1			Y	Y		Y		Y										Y	Y	Y	Y				Y																			
4011	A_23_P13364	NUCB2			Y	Y	Y	Y	Y	Y	Y	Y	Y	Y					Y	Y		Y	Y				Y	Y	Y	Y						Y										
2819	A_23_P74648	NUDC																		Y																										
C10729.1	A_23_P123343	NUDCD1	Y	Y	Y				Y	Y																	Y			Y						Y										
2075	A_23_P31477	NUDCD3			Y	Y		Y		Y								Y		Y		Y					Y																			
12821	A_23_P134295	NUDT1	Y	Y	Y	Y		Y		Y								Y		Y		Y					Y	Y	Y	Y						Y										
20545	A_23_P73787	NUDT10																	Y	Y																										
22404	A_23_P62387	NUDT11							Y	Y																																				
16787	A_23_P259090	NUDT12	Y	Y	Y	Y		Y	Y	Y	Y	Y	Y	Y				Y	Y	Y		Y	Y	Y			Y	Y	Y	Y						Y										
2740	A_23_P310560	NUDT16			Y				Y	Y								Y	Y	Y		Y		Y			Y																			
15227	A_23_P123457	NUDT18			Y	Y		Y	Y	Y									Y								Y			Y						Y										

5566	A_23_P39215	NUDT19			Y	Y		Y		Y								Y	Y	Y		Y	Y	Y			Y																			
6824	A_23_P2366	NUDT4			Y																																									
19584	A_23_P1196	NUDT5	Y	Y																										Y						Y										
9131	A_23_P155857	NUDT6			Y	Y	Y	Y	Y	Y	Y	Y	Y	Y				Y		Y			Y				Y			Y	Y	Y	Y	Y	Y	Y				Y						
11176	A_23_P52569	NUDT8			Y	Y	Y	Y	Y	Y	Y	Y	Y	Y						Y			Y				Y			Y						Y										
17605	A_23_P94860	NUDT9			Y														Y	Y							Y																			
11612	A_23_P36939	NUFIP1				Y														Y								Y																		
22347	A_23_P88381	NUMB																Y			Y																									
16905	A_23_P14062	NUP107			Y	Y		Y		Y																																				
19707	A_23_P12503	NUP133			Y	Y		Y	Y	Y	Y	Y	Y	Y						Y	Y	Y	Y				Y			Y						Y										
21031	A_23_P122254	NUP153				Y			Y	Y																																				
13750	A_23_P43726	NUP160							Y	Y																																				
712	A_23_P257091	NUP214			Y															Y	Y	Y	Y																							
279	A_23_P102320	NUP35			Y														Y	Y		Y	Y																							
7022	A_23_P419065	NUP35			Y														Y	Y	Y	Y	Y																							
21253	A_23_P31055	NUP43			Y	Y		Y		Y	Y	Y	Y	Y						Y																										
4734	A_23_P434768	NUP50				Y														Y								Y																		
7212	A_23_P92320	NUP54				Y																																								
17534	A_23_P208683	NUP62			Y	Y		Y		Y								Y	Y	Y		Y	Y	Y			Y																			
15051	A_23_P33914	NUP62CL	Y		Y													Y	Y	Y		Y	Y	Y																						
6857	A_23_P26777	NUP85			Y													Y		Y			Y																							
11231	A_23_P89056	NUP93			Y	Y		Y	Y	Y									Y	Y	Y	Y	Y				Y	Y	Y	Y																
14469	A_23_P203586	NUP98																	Y																											
1818	A_23_P204891	NUPL1				Y																								Y						Y										
18170	A_23_P123039	NUPL2																		Y			Y																							
16491	A_23_P100362	NUPR1			Y	Y	Y	Y	Y	Y	Y	Y	Y	Y													Y	Y	Y	Y						Y										
199	A_23_P93082	NUS1			Y				Y	Y																				Y						Y										
19438	A_23_P118038	NUTF2			Y	Y		Y	Y	Y	Y	Y	Y	Y				Y	Y	Y	Y	Y	Y	Y	Y	Y	Y	Y	Y	Y	Y	Y	Y			Y										
3880	A_23_P96990	NVL			Y	Y	Y	Y	Y	Y	Y	Y	Y	Y							Y						Y																			
15067	A_23_P13353	NXF1			Y	Y		Y		Y																																				
1088	A_23_P114354	NXF5				Y			Y	Y																				Y						Y										
3459	A_23_P61778	NXN			Y				Y	Y	Y	Y	Y	Y				Y		Y		Y	Y				Y																			
3986	A_23_P111604	NXPH1			Y	Y		Y	Y	Y																	Y			Y						Y										
5352	A_23_P4191	NXPH3			Y															Y			Y				Y	Y	Y	Y						Y										
20771	A_23_P251695	NXT1				Y			Y	Y	Y	Y	Y	Y														Y		Y						Y										
14735	A_23_P85136	NXT2			Y																									Y						Y										
17911	A_23_P137046	NYX			Y																						Y																			
18311	A_23_P310590	OAF			Y								Y					Y	Y	Y	Y	Y	Y	Y	Y	Y	Y				Y															
11460	A_23_P64828	OAS1																			Y																									
15561	A_23_P139786	OASL											Y							Y																										
2377	A_23_P409951	OAZ1			Y				Y	Y			Y							Y										Y						Y										
9386	A_23_P106462	OAZ2	Y	Y																							Y																			
11421	A_23_P432587	OAZ3																			Y																									
4908	A_23_P329198	OBFC2A	Y	Y	Y																									Y						Y										
17848	A_23_P87482	OBFC2B																		Y	Y						Y																			
11395	A_23_P112327	OBP2A							Y	Y										Y								Y																		
18172	A_23_P129133	OCA2			Y				Y	Y										Y			Y							Y						Y										
13631	A_23_P90523	OCEL1																		Y																										
3518	A_23_P213093	OCIAD1			Y	Y	Y	Y		Y	Y	Y						Y		Y			Y				Y	Y	Y	Y																
13084	A_23_P121702	OCIAD2			Y	Y		Y	Y	Y																				Y						Y										
22072	A_23_P92672	OCLN							Y	Y																																				
14846	A_23_P32903	OCRL			Y	Y		Y		Y									Y	Y			Y				Y																			
9126	A_23_P58229	ODAM																Y		Y		Y																								
3257	A_23_P165840	ODC1	Y	Y	Y						Y		Y	Y				Y	Y	Y			Y	Y			Y	Y	Y	Y						Y										
3844	A_23_P60488	ODF2				Y																																								
20679	A_23_P53094	ODF3			Y				Y	Y	Y	Y	Y	Y													Y																			
13236	A_23_P357900	ODF3L1							Y	Y																																				
9679	A_23_P47746	ODZ4																	Y				Y																							
6952	A_23_P254226	OFD1				Y																						Y		Y						Y										
3923	A_23_P123140	OGDH			Y													Y	Y	Y	Y	Y	Y	Y	Y	Y	Y				Y															
4584	A_23_P53324	OGFOD2			Y															Y	Y	Y					Y																			
22139	A_23_P28707	OGFR			Y	Y		Y		Y										Y	Y	Y	Y				Y	Y	Y	Y																
17302	A_23_P7791	OGFRL1							Y	Y	Y	Y	Y	Y													Y			Y						Y										
12122	A_23_P82990	OGN				Y			Y	Y																		Y		Y						Y										
22150	A_23_P381979	OGT			Y	Y		Y	Y	Y																																				
16433	A_23_P379614	OIP5	Y	Y	Y													Y									Y																			
15558	A_23_P63777	OIT3	Y																																											
1881	A_23_P28420	OLA1			Y	Y		Y	Y	Y									Y	Y		Y	Y				Y																			
9890	A_23_P219161	OLFM1			Y																						Y																			
3251	A_23_P67151	OLFM2			Y	Y		Y	Y	Y																	Y			Y						Y										
17755	A_23_P2789	OLFM4																	Y			Y																								
19907	A_23_P147664	OLFML1																					Y																							
21977	A_23_P137689	OLFML2B			Y						Y		Y	Y													Y																			
17227	A_23_P115172	OLFML3																	Y											Y						Y										
18466	A_23_P138137	OMA1			Y	Y		Y	Y	Y	Y	Y	Y	Y						Y	Y		Y				Y	Y	Y	Y						Y										
9731	A_23_P55288	OMG																									Y																			
1533	A_23_P211797	OPA1			Y	Y		Y	Y	Y													Y																							

20988	A_23_P67115	OPA3			Y	Y		Y		Y										Y			Y				Y																			
18377	A_23_P75247	OPALIN																												Y						Y										
6082	A_23_P62420	OPHN1							Y	Y																				Y						Y										
2236	A_23_P114374	OPN1LW																												Y						Y										
6687	A_23_P96623	OPN1MW							Y	Y										Y																										
1483	A_23_P419156	OPN1SW				Y																					Y																			
4025	A_23_P74391	OPN3			Y																						Y																			
17852	A_23_P24234	OPN4																					Y																							
6813	A_23_P169061	OPRK1				Y																						Y																		
19970	A_23_P6140	OPRL1	Y	Y	Y	Y		Y		Y										Y			Y				Y																			
16235	A_23_P145480	OPRM1				Y																																								
19706	A_23_P97096	OPTC							Y	Y																	Y																			
11071	A_23_P1461	OPTN			Y				Y	Y																	Y																			
1432	A_23_P75867	OR10A4			Y	Y		Y		Y																	Y	Y	Y	Y																
1253	A_23_P76184	OR10AD1			Y																																									
1307	A_23_P25798	OR10G2			Y																									Y						Y										
17521	A_23_P87066	OR10G7																	Y																											
17882	A_23_P125204	OR10G8				Y																						Y																		
15090	A_23_P50799	OR10H2			Y				Y	Y									Y	Y			Y				Y			Y						Y										
7505	A_23_P397468	OR10J1																									Y																			
15827	A_23_P126031	OR10J5				Y			Y	Y																	Y	Y	Y	Y						Y										
10008	A_23_P12109	OR10K1			Y																	Y					Y																			
16385	A_23_P36112	OR10V1							Y	Y																																				
17721	A_23_P161901	OR10W1			Y																						Y																			
3132	A_23_P160715	OR10Z1				Y																					Y	Y	Y	Y																
13767	A_23_P59099	OR11A1							Y	Y																				Y						Y										
20367	A_23_P37231	OR11G2							Y	Y																																				
8676	A_23_P25826	OR11H6																		Y		Y	Y																							
4511	A_23_P97755	OR11L1			Y	Y		Y	Y	Y										Y			Y				Y			Y						Y										
1269	A_23_P93295	OR12D2			Y	Y		Y		Y																	Y	Y	Y	Y																
20069	A_23_P43396	OR13C8			Y																						Y																			
16717	A_23_P74976	OR13G1				Y																																								
22079	A_23_P161108	OR14A16							Y	Y																																				
13124	A_23_P115424	OR14C36				Y			Y	Y																																				
15128	A_23_P130281	OR1A2																		Y			Y																							
5794	A_23_P86350	OR1C1																												Y						Y										
16968	A_23_P15838	OR1D4																												Y						Y										
16614	A_23_P101073	OR1E1	Y		Y	Y	Y	Y	Y	Y	Y	Y							Y	Y	Y	Y	Y				Y	Y	Y	Y																
12737	A_23_P107499	OR1E2			Y	Y		Y	Y	Y									Y	Y		Y	Y				Y	Y	Y	Y																
13671	A_23_P318890	OR1F1			Y													Y	Y	Y		Y		Y																						
3362	A_23_P38634	OR1G1																					Y																							
17913	A_23_P9372	OR1J1			Y														Y								Y																			
10108	A_23_P71841	OR1L6			Y	Y		Y		Y																	Y	Y	Y	Y																
11871	A_23_P135226	OR1N2																			Y																									
13576	A_23_P82585	OR2A1			Y	Y	Y	Y	Y	Y	Y	Y	Y	Y					Y	Y			Y				Y	Y	Y	Y						Y										
11900	A_23_P59783	OR2A14																			Y																									
7798	A_23_P301175	OR2B11			Y	Y		Y	Y	Y																				Y						Y										
20997	A_23_P163737	OR2C1																									Y																			
3422	A_23_P115568	OR2C3			Y																						Y																			
22435	A_23_P63572	OR2G3																	Y	Y		Y								Y						Y										
13362	A_23_P111092	OR2H1																		Y	Y		Y																							
20183	A_23_P19428	OR2J2				Y																								Y						Y										
741	A_23_P382137	OR2L13							Y	Y																																				
10291	A_23_P126945	OR2L3																		Y																										
14101	A_23_P104039	OR2M7			Y																						Y																			
6643	A_23_P135007	OR2S2			Y																																									
382	A_23_P35174	OR2T10																										Y																		
15790	A_23_P52040	OR2T2							Y	Y																				Y						Y										
7683	A_23_P126820	OR2T4																									Y																			
17448	A_23_P12378	OR2T5																					Y																							
2338	A_23_P255389	OR2W1																										Y																		
481	A_23_P39417	OR2Z1			Y	Y		Y		Y								Y	Y	Y				Y			Y	Y	Y	Y																
12694	A_23_P50031	OR3A1																									Y																			
18981	A_23_P55504	OR3A2			Y	Y		Y		Y																	Y																			
3143	A_23_P107483	OR3A3				Y																																								
2189	A_23_P139103	OR4A15																Y		Y	Y																									
16444	A_23_P116496	OR4A47				Y														Y																										
2356	A_23_P52914	OR4C15	Y		Y																						Y																			
15910	A_23_P2077	OR4C3			Y																						Y																			
13993	A_23_P98512	OR4C6																Y	Y			Y																								
14477	A_23_P66392	OR4D1			Y				Y	Y																	Y			Y						Y										
20068	A_23_P77797	OR4D2			Y		Y		Y	Y	Y	Y							Y	Y							Y			Y						Y										
19736	A_23_P75659	OR4D5				Y																																								
704	A_23_P87167	OR4D6			Y																						Y			Y						Y										
13030	A_23_P3287	OR4F15																									Y																			
19084	A_23_P48882	OR4F6			Y	Y		Y	Y	Y																				Y						Y										
1966	A_23_P2973	OR4K1			Y																						Y																			

5168	A_23_P76891	OR4K15			Y				Y	Y									Y								Y																			
20639	A_23_P54089	OR4K17				Y																																								
21284	A_23_P99688	OR4K2																									Y			Y						Y										
16106	A_23_P117485	OR4M1																Y		Y	Y	Y											Y													
19764	A_23_P13479	OR4S1																				Y								Y						Y										
11726	A_23_P104925	OR4S2				Y																																								
15964	A_23_P13493	OR51E1			Y																																									
2361	A_23_P116506	OR51G2			Y				Y	Y																	Y																			
11938	A_23_P162062	OR51L1							Y	Y																	Y																			
7705	A_23_P127899	OR51S1																												Y						Y										
1540	A_23_P24856	OR51T1			Y	Y		Y		Y																		Y		Y						Y										
13095	A_23_P127859	OR52E4				Y																																								
19072	A_23_P98746	OR52K2				Y																						Y		Y						Y										
2257	A_23_P53070	OR52N1																									Y			Y						Y										
3209	A_23_P64474	OR52N5			Y	Y		Y	Y	Y									Y								Y																			
19739	A_23_P13407	OR52W1																												Y						Y										
17351	A_23_P13417	OR56B4																			Y																									
11833	A_23_P75764	OR5A1				Y			Y	Y																																				
4509	A_23_P3007	OR5AU1			Y	Y	Y	Y	Y	Y	Y	Y															Y	Y	Y	Y						Y										
2103	A_23_P116344	OR5B3																									Y																			
3832	A_23_P60441	OR5C1			Y																																									
17790	A_23_P36057	OR5D14			Y	Y		Y		Y								Y	Y	Y		Y	Y	Y			Y	Y	Y	Y																
3493	A_23_P127684	OR5F1			Y																						Y			Y						Y										
20161	A_23_P116301	OR5F1			Y															Y		Y																								
13982	A_23_P166984	OR5H2				Y																																								
2856	A_23_P155586	OR5H6							Y	Y																				Y						Y										
2311	A_23_P87115	OR5J2				Y													Y	Y			Y				Y	Y	Y	Y						Y										
3675	A_23_P24653	OR5L1							Y	Y													Y							Y						Y										
1939	A_23_P1855	OR5L2																			Y																									
22338	A_23_P64333	OR5M10							Y	Y																	Y			Y						Y										
10009	A_23_P104939	OR5M9																									Y																			
10575	A_23_P42241	OR5V1																		Y			Y																							
20649	A_23_P161852	OR5W2			Y				Y	Y																	Y	Y	Y	Y						Y										
22140	A_23_P20116	OR6B1			Y	Y		Y		Y			Y					Y	Y								Y	Y	Y	Y						Y										
14556	A_23_P108793	OR6B2				Y												Y	Y	Y	Y	Y		Y			Y																			
15617	A_23_P86369	OR6F1																		Y		Y	Y																							
7727	A_23_P51761	OR6K2			Y				Y	Y																	Y																			
13943	A_23_P161891	OR6Q1																										Y																		
3081	A_23_P54075	OR6S1																												Y						Y										
65	A_23_P161815	OR6T1				Y																																								
16445	A_23_P168714	OR6V1			Y	Y	Y	Y	Y	Y	Y	Y											Y				Y			Y						Y								Y		
18965	A_23_P123164	OR6W1P																			Y																									
12014	A_23_P139036	OR6X1			Y																																									
1624	A_23_P28023	OR7A17			Y				Y	Y																	Y																			
21686	A_23_P16630	OR7A5				Y																																								
15147	A_23_P108265	OR7C2			Y																						Y																			
9827	A_23_P27511	OR7D2			Y															Y	Y	Y	Y																							
14716	A_23_P130544	OR7D4																									Y																			
18128	A_23_P16110	OR7E24	Y	Y	Y	Y		Y	Y	Y	Y	Y	Y	Y													Y	Y	Y	Y																
5737	A_23_P408930	OR7E5P	Y	Y														Y	Y	Y				Y			Y			Y						Y										
7808	A_23_P150441	OR8B3							Y	Y																																				
8551	A_23_P1819	OR8B8			Y	Y		Y	Y	Y	Y	Y	Y	Y													Y	Y	Y	Y						Y										
18404	A_23_P127662	OR8D1																		Y		Y																								
16398	A_23_P395326	OR8G5			Y													Y	Y								Y																			
13287	A_23_P53378	OR8S1																			Y		Y																							
9265	A_23_P24674	OR8U1			Y													Y	Y	Y			Y	Y			Y	Y	Y	Y																
20212	A_23_P36067	OR9G4			Y															Y	Y	Y					Y																			
579	A_23_P24698	OR9I1																												Y						Y										
14115	A_23_P99016	OR9K2			Y	Y		Y		Y										Y		Y																								
17305	A_23_P64358	OR9Q1			Y	Y		Y	Y	Y																																				
5092	A_23_P258837	ORAI1			Y	Y	Y	Y	Y	Y	Y	Y	Y	Y				Y	Y	Y		Y	Y	Y			Y	Y	Y	Y						Y										
17341	A_23_P70915	ORAI2			Y															Y		Y	Y																							
3728	A_23_P106898	ORAI3																												Y						Y										
648	A_23_P45799	ORC1L			Y													Y	Y	Y		Y		Y			Y																			
2837	A_23_P42045	ORC3L				Y																																								
8967	A_23_P131149	ORC4L																		Y	Y		Y																							
997	A_23_P31414	ORC5L																		Y																										
2565	A_23_P169494	ORM1			Y	Y		Y		Y																																				
14167	A_23_P9485	ORM2			Y	Y		Y		Y																																				
13934	A_23_P120194	ORMDL1																		Y																										
21725	A_23_P87500	ORMDL2							Y	Y																																				
14610	A_23_P129824	ORMDL3			Y		Y		Y	Y	Y	Y								Y			Y				Y																			
15557	A_23_P38190	ORMDL3							Y	Y																																				
16513	A_23_P203790	OS9																Y		Y	Y	Y	Y										Y													
8406	A_23_P24709	OSBP																					Y										Y													
21877	A_23_P336449	OSBP																					Y																									
7437	A_23_P321935	OSBP2																					Y																									

4137	A_23_P6914	OSBPL11				Y			Y	Y										Y																										
9869	A_23_P10442	OSBPL1A			Y														Y	Y	Y		Y				Y																			
4007	A_23_P210995	OSBPL2				Y													Y	Y		Y																								
10375	A_23_P108823	OSBPL6			Y	Y		Y	Y	Y	Y	Y	Y	Y					Y	Y		Y						Y		Y						Y										
11703	A_23_P204810	OSBPL8																	Y									Y																		
21800	A_23_P148916	OSBPL9							Y	Y									Y	Y		Y	Y				Y																			
5663	A_23_P50365	OSCAR			Y	Y	Y	Y	Y	Y	Y	Y																Y		Y						Y										
15186	A_23_P54078	OSGEP																			Y																									
10052	A_23_P210082	OSGEPL1																		Y																										
10422	A_23_P166408	OSM			Y	Y		Y	Y	Y																				Y						Y										
6957	A_23_P156290	OSMR																	Y	Y			Y																							
12	A_23_P323270	OSR1			Y				Y	Y																				Y						Y										
21542	A_23_P40761	OSTalpha																Y												Y						Y										
20512	A_23_P411814	OSTC			Y				Y	Y																				Y						Y										
16486	A_23_P304395	OSTCL			Y				Y	Y																				Y						Y										
12730	A_23_P60296	OSTF1	Y		Y	Y		Y	Y	Y												Y					Y	Y	Y	Y																
14461	A_23_P255175	OSTN																			Y									Y						Y										
3602	A_23_P392511	OTOP2			Y	Y		Y		Y																																				
6924	A_23_P250431	OTOR							Y	Y																																				
17891	A_23_P90991	OTOS																		Y																										
924	A_23_P24375	OTUB1			Y	Y		Y	Y	Y										Y		Y					Y	Y	Y	Y						Y										
17921	A_23_P74264	OTUD3			Y																																									
20485	A_23_P69642	OTUD4																Y																												
12441	A_23_P3413	OTUD7A			Y	Y		Y	Y	Y								Y	Y	Y		Y	Y	Y			Y	Y	Y	Y						Y										
3885	A_23_P138158	OTUD7B							Y	Y																																				
14741	A_23_P403284	OTX1			Y				Y	Y																																				
17519	A_23_P108362	OTX1	Y	Y	Y																																									
3653	A_23_P118462	OVCA2				Y																																								
7414	A_23_P162192	OVCH2			Y																																									
18958	A_23_P103756	OVGP1				Y			Y	Y																																				
3535	A_23_P202810	OVOL1							Y	Y																				Y						Y										
18814	A_23_P143348	OVOL2			Y																																									
22022	A_23_P25069	OVOS2							Y	Y																				Y						Y										
13790	A_23_P2998	OXA1L																		Y																										
21551	A_23_P20905	OXCT1																		Y																										
15124	A_23_P137896	OXCT2																		Y																										
21306	A_23_P139986	OXGR1																		Y			Y				Y			Y						Y										
2662	A_23_P418413	OXSR1			Y				Y	Y										Y		Y					Y			Y						Y										
3346	A_23_P57133	OXT			Y		Y				Y							Y	Y	Y				Y			Y																			
21080	A_23_P132619	OXTR			Y				Y	Y																	Y																			
12006	A_23_P372845	P2RX1																Y				Y																								
9902	A_23_P73468	P2RX2			Y	Y		Y	Y	Y									Y	Y										Y						Y										
20582	A_23_P124003	P2RX2			Y	Y		Y	Y	Y																	Y	Y	Y	Y																
19018	A_23_P53625	P2RX4																Y				Y																								
15724	A_23_P68797	P2RX6			Y	Y		Y	Y	Y									Y	Y			Y				Y																			
8851	A_23_P211939	P2RY14																	Y																											
11258	A_23_P24903	P2RY2							Y	Y											Y							Y																		
13485	A_23_P114307	P2RY4			Y	Y		Y		Y																	Y	Y	Y	Y						Y										
10880	A_23_P2705	P2RY5				Y																						Y		Y						Y										
11789	A_23_P64611	P2RY6	Y	Y	Y	Y	Y	Y	Y	Y	Y	Y	Y	Y													Y	Y	Y	Y						Y										
20850	A_23_P127957	P4HA3													Y					Y																										
10411	A_23_P107412	P4HB			Y	Y	Y	Y		Y	Y	Y						Y	Y	Y	Y	Y	Y	Y	Y	Y	Y				Y															
13560	A_23_P139334	PAAF1			Y	Y		Y	Y	Y										Y							Y			Y						Y										
11763	A_23_P48314	PABPC3																									Y																			
17212	A_23_P51718	PABPC4			Y													Y	Y	Y	Y	Y	Y	Y	Y	Y	Y				Y															
9474	A_23_P84047	PABPC5																										Y																		
3921	A_23_P128828	PABPN1																Y	Y	Y	Y	Y		Y			Y																			
4683	A_23_P138870	PACS1			Y	Y		Y		Y																	Y																			
2332	A_23_P80377	PACSIN2																Y	Y			Y																								
1035	A_23_P115478	PADI1			Y														Y	Y		Y	Y				Y	Y	Y	Y						Y										
10077	A_23_P201747	PADI2	Y	Y	Y	Y		Y	Y	Y								Y	Y	Y				Y			Y	Y	Y	Y						Y										
16548	A_23_P126869	PADI3			Y	Y		Y		Y																		Y		Y						Y										
9593	A_23_P138260	PADI4			Y	Y	Y	Y	Y	Y	Y	Y						Y	Y	Y			Y	Y			Y	Y	Y	Y						Y										
8621	A_23_P142266	PAF1																												Y						Y										
7263	A_23_P255503	PAFAH1B2																												Y						Y										
13204	A_23_P137765	PAFAH2			Y	Y		Y		Y																																				
18630	A_23_P171104	PAGE1	Y		Y	Y		Y		Y								Y	Y	Y		Y	Y	Y			Y	Y	Y	Y						Y										
20295	A_23_P2502	PAH	Y		Y	Y	Y	Y	Y	Y	Y	Y	Y	Y				Y	Y	Y	Y	Y	Y	Y	Y	Y	Y	Y	Y	Y	Y	Y						Y				Y			Y	
12572	A_23_P207811	PAIP1			Y	Y		Y		Y									Y	Y	Y		Y				Y	Y	Y	Y																
C11706.5	A_23_P122674	PAK1IP1							Y	Y																																				
20472	A_23_P106441	PAK2																Y									Y																			
10006	A_23_P346813	PAK3			Y						Y		Y	Y													Y																			
1482	A_23_P67683	PAK4			Y	Y	Y	Y		Y	Y	Y						Y	Y	Y		Y	Y	Y			Y																			
3717	A_23_P206200	PAK6			Y				Y	Y										Y			Y				Y			Y						Y										
8166	A_23_P129569	PALB2			Y	Y		Y	Y	Y										Y			Y				Y																			
15973	A_23_P213102	PALLD																	Y									Y																		
8524	A_23_P342654	PALM2-AKAP2			Y	Y		Y		Y																	Y	Y	Y	Y																

5590	A_23_P23664	PALMD																												Y						Y										
13348	A_23_P213678	PAM							Y	Y								Y	Y	Y		Y		Y			Y	Y	Y	Y						Y										
12101	A_23_P64689	PAN2	Y	Y	Y	Y		Y	Y	Y	Y	Y	Y	Y				Y	Y	Y		Y	Y	Y			Y	Y	Y	Y						Y										
17943	A_23_P127054	PANK1			Y																						Y	Y	Y	Y						Y										
12575	A_23_P149690	PANK4			Y														Y	Y		Y																								
10570	A_23_P155159	PANX2							Y	Y																				Y						Y										
14684	A_23_P87072	PANX3																	Y																											
16751	A_23_P385771	PAOX																	Y																											
18620	A_23_P314202	PAPD4			Y	Y	Y	Y	Y	Y	Y	Y						Y	Y	Y		Y	Y	Y			Y	Y	Y	Y																
8120	A_23_P129723	PAPD5			Y																																									
12903	A_23_P140347	PAPLN			Y																																									
12373	A_23_P209694	PAPOLG																		Y																										
9112	A_23_P216742	PAPPA			Y													Y		Y		Y					Y																			
12574	A_23_P351270	PAPPA																		Y		Y																								
12614	A_23_P144465	PAPSS1							Y	Y																																				
16394	A_23_P104492	PAPSS2			Y													Y									Y																			
14916	A_23_P66213	PAQR4			Y	Y	Y	Y		Y	Y	Y						Y	Y	Y		Y		Y			Y	Y	Y	Y						Y										
22502	A_23_P397334	PAQR4																Y	Y																											
22129	A_23_P163251	PAQR5							Y	Y																																				
17034	A_23_P97283	PAQR6																										Y																		
9580	A_23_P6001	PARD6B			Y	Y		Y		Y								Y				Y																								
6362	A_23_P141699	PARD6G			Y	Y		Y		Y										Y		Y	Y																							
18234	A_23_P147685	PARG																		Y																										
9313	A_23_P124687	PARK2																												Y						Y										
13501	A_23_P147465	PARK2			Y	Y		Y	Y	Y																	Y			Y						Y										
16912	A_23_P136077	PARK2																		Y																										
1085	A_23_P74740	PARK7			Y				Y	Y										Y							Y																			
17126	A_23_P92247	PARL			Y																																									
3264	A_23_P113026	PARN	Y		Y						Y		Y	Y													Y																			
14755	A_23_P114783	PARP1			Y	Y		Y		Y																																				
19781	A_23_P84762	PARP10			Y	Y		Y	Y	Y										Y		Y	Y																							
4109	A_23_P343837	PARP11																									Y																			
13901	A_23_P111804	PARP12				Y																																								
12445	A_23_P163278	PARP16			Y				Y	Y										Y							Y																			
9051	A_23_P163027	PARP2				Y																																								
16473	A_23_P92072	PARP3	Y	Y	Y	Y		Y	Y	Y								Y		Y	Y	Y	Y				Y	Y	Y	Y			Y	Y		Y										
3741	A_23_P151879	PARP6			Y		Y				Y							Y	Y	Y			Y	Y			Y																			
7282	A_23_P69383	PARP9																		Y																										
3915	A_23_P51291	PARS2																										Y																		
10123	A_23_P335268	PART1																Y																												
8324	A_23_P47640	PARVA				Y												Y		Y	Y	Y						Y																		
14391	A_23_P40718	PARVB	Y		Y	Y	Y	Y	Y	Y	Y	Y	Y	Y					Y	Y	Y	Y	Y				Y	Y	Y	Y						Y										
10429	A_23_P250412	PARVG	Y		Y										Y						Y		Y																							
7247	A_23_P209815	PASK																	Y																											
10121	A_23_P373077	PATL1			Y															Y			Y				Y																			
9394	A_23_P211459	PATZ1			Y	Y		Y	Y	Y																		Y																		
22070	A_23_P209492	PAX3																		Y		Y	Y																							
20416	A_23_P71624	PAX5							Y	Y	Y	Y	Y	Y																Y						Y										
18592	A_23_P1823	PAX6				Y																																								
17752	A_23_P126225	PAX7																		Y							Y			Y						Y										
11548	A_23_P59426	PAXIP1			Y	Y		Y	Y	Y	Y	Y	Y	Y					Y	Y	Y		Y				Y	Y	Y	Y	Y	Y	Y	Y	Y	Y				Y						
20157	A_23_P149998	PBLD	Y	Y	Y	Y		Y	Y	Y										Y								Y		Y						Y										
1489	A_23_P332260	PBOV1																			Y																									
12179	A_23_P255942	PBOV1			Y																						Y			Y						Y										
15309	A_23_P365874	PBRM1				Y			Y	Y																																				
18227	A_23_P218863	PBRM1			Y				Y	Y										Y										Y						Y										
11779	A_23_P62948	PBX1																	Y																											
8438	A_23_P103386	PBXIP1			Y	Y		Y		Y								Y	Y	Y	Y	Y	Y	Y	Y	Y	Y				Y															
1528	A_23_P63926	PCBD1																	Y											Y						Y										
2175	A_23_P367405	PCBD1																	Y											Y						Y										
10735	A_23_P204059	PCBP2																					Y																							
7824	A_23_P147255	PCBP3	Y		Y	Y	Y	Y		Y	Y	Y						Y	Y	Y	Y	Y	Y	Y	Y	Y	Y	Y	Y	Y	Y	Y	Y	Y	Y	Y		Y				Y			Y	
22434	A_23_P113274	PCBP3			Y	Y		Y		Y										Y			Y				Y																			
21556	A_23_P48358	PCCA			Y	Y	Y	Y	Y	Y	Y	Y	Y	Y				Y		Y		Y					Y	Y	Y	Y						Y										
3211	A_23_P121051	PCCB			Y	Y		Y		Y										Y																										
7566	A_23_P213359	PCDH1			Y													Y	Y	Y		Y	Y	Y																						
1699	A_23_P158851	PCDH10																												Y						Y										
19496	A_23_P147314	PCDH11Y				Y																	Y																							
3948	A_23_P423302	PCDH12	Y		Y													Y					Y																							
1211	A_23_P161331	PCDH15																									Y			Y						Y										
2255	A_23_P256872	PCDH18																												Y						Y										
647	A_23_P204885	PCDH20							Y	Y										Y		Y																								
7329	A_23_P133338	PCDH24			Y	Y		Y		Y																	Y			Y						Y										
20028	A_23_P378366	PCDH7																	Y																											
14144	A_23_P36985	PCDH8							Y	Y			Y																																	
3861	A_23_P167401	PCDHB11			Y	Y	Y	Y	Y	Y	Y	Y															Y	Y	Y	Y																
17642	A_23_P156010	PCDHB12			Y																						Y																			

3408	A_23_P144627	PCDHB13			Y																		Y				Y																			
12516	A_23_P58464	PCDHB6																									Y																			
14628	A_23_P256553	PCDHB7				Y												Y																												
13173	A_23_P41599	PCDHB8																Y	Y																											
5965	A_23_P354734	PCDHGA8			Y	Y		Y		Y										Y			Y				Y																			
5358	A_23_P116578	PCF11			Y	Y		Y		Y										Y		Y																								
8688	A_23_P21346	PCGF3																					Y																							
16476	A_23_P115702	PCGF6																		Y																										
750	A_23_P408249	PCK1			Y	Y		Y	Y	Y	Y	Y	Y	Y													Y	Y	Y	Y						Y						Y				
20281	A_23_P140207	PCK2			Y																																									
2363	A_23_P82950	PCM1			Y	Y	Y	Y	Y	Y	Y	Y	Y	Y				Y	Y	Y		Y	Y	Y			Y	Y	Y	Y						Y										
10710	A_23_P210827	PCMTD2			Y	Y		Y	Y	Y										Y		Y	Y				Y																			
2600	A_23_P114585	PCNXL2			Y																						Y																			
10614	A_23_P116063	PCNXL3																		Y		Y	Y																							
17612	A_23_P251494	PCOLCE			Y													Y		Y	Y	Y	Y				Y																			
523	A_23_P57709	PCOLCE2			Y				Y	Y																	Y	Y	Y	Y						Y										
1401	A_23_P109322	PCP4				Y																						Y																		
13451	A_23_P147936	PCP4				Y																	Y																							
18507	A_23_P213508	PCSK1			Y																						Y																			
5529	A_23_P22487	PCSK1N			Y				Y	Y									Y	Y	Y	Y	Y				Y			Y						Y										
20284	A_23_P68568	PCSK2																Y																												
6728	A_23_P257003	PCSK5			Y	Y		Y	Y	Y																	Y	Y	Y	Y																
12983	A_23_P151907	PCSK6			Y				Y	Y									Y				Y				Y	Y	Y	Y						Y										
4466	A_23_P203095	PCSK7			Y	Y	Y	Y	Y	Y	Y	Y						Y	Y	Y		Y	Y	Y			Y	Y	Y	Y						Y							Y			
222	A_23_P200240	PCSK9																Y	Y	Y		Y	Y	Y																						
13879	A_23_P501414	PCTK1			Y	Y		Y		Y								Y	Y	Y		Y	Y	Y			Y																			
7028	A_23_P95527	PCTK2			Y	Y		Y		Y									Y	Y		Y	Y				Y	Y	Y	Y																
18286	A_23_P33376	PCTK2			Y	Y		Y	Y	Y	Y	Y	Y	Y						Y			Y				Y	Y	Y	Y						Y										
2766	A_23_P201863	PCTK3							Y	Y																				Y						Y										
13193	A_23_P56637	PCYOX1			Y														Y	Y		Y	Y																							
13716	A_23_P30275	PCYOX1L			Y	Y	Y	Y	Y	Y	Y	Y	Y							Y							Y																			
11771	A_23_P252688	PCYT1A				Y														Y								Y																		
1186	A_23_P55407	PCYT2																	Y																											
2690	A_23_P151196	PDAP1			Y	Y		Y		Y										Y		Y																								
8893	A_23_P123256	PDAP1			Y															Y																										
6442	A_23_P23090	PDC																										Y		Y						Y										
10436	A_23_P136405	PDCD1	Y		Y		Y		Y	Y	Y	Y							Y	Y							Y	Y	Y	Y						Y								Y		
7921	A_23_P18325	PDCD10				Y																																								
14637	A_23_P94411	PDCD1LG2	Y	Y																																										
20343	A_23_P145146	PDCD2																Y		Y	Y	Y																								
1026	A_23_P27947	PDCD2L			Y																																									
2333	A_23_P258862	PDCD4							Y	Y																				Y						Y										
9535	A_23_P50608	PDCD5	Y	Y																																										
2019	A_23_P218997	PDCD6																					Y				Y																			
13032	A_23_P129118	PDCD7																Y	Y																											
6928	A_23_P210128	PDE11A																									Y																			
7878	A_23_P394367	PDE11A			Y		Y				Y																Y																			
12595	A_23_P343914	PDE12											Y							Y			Y																							
19837	A_23_P406508	PDE1C																		Y		Y					Y																			
15772	A_23_P87379	PDE2A																		Y	Y	Y																								
21331	A_23_P401106	PDE2A																					Y																							
13565	A_23_P60837	PDE3A																	Y																											
18962	A_23_P116114	PDE3B							Y	Y																																				
4893	A_23_P208339	PDE4A	Y	Y	Y				Y	Y										Y	Y																									
14092	A_23_P119583	PDE4C			Y	Y		Y	Y	Y	Y	Y	Y	Y														Y		Y						Y										
9953	A_23_P124456	PDE4D			Y														Y								Y																			
10333	A_23_P158406	PDE4D			Y				Y	Y	Y	Y	Y	Y																Y						Y										
2867	A_23_P200801	PDE4DIP			Y	Y		Y	Y	Y																	Y			Y						Y										
9373	A_23_P149150	PDE4DIP																Y	Y																											
19982	A_23_P81588	PDE6A			Y	Y		Y	Y	Y									Y									Y		Y						Y										
6364	A_23_P102517	PDE6D			Y	Y	Y	Y		Y	Y	Y																																		
17410	A_23_P59338	PDE7B																									Y																			
7620	A_23_P3261	PDE8A			Y				Y	Y										Y							Y			Y						Y										
20757	A_23_P394154	PDE8A																		Y										Y						Y										
1744	A_23_P259065	PDE8B	Y	Y	Y	Y	Y	Y	Y	Y	Y	Y	Y	Y				Y	Y	Y		Y		Y			Y	Y	Y	Y						Y										
17543	A_23_P29096	PDE9A			Y				Y	Y								Y									Y			Y						Y										
6034	A_23_P113701	PDGFA																					Y																							
12637	A_23_P58396	PDGFC																Y	Y	Y				Y																						
4503	A_23_P122189	PDGFRB			Y	Y	Y	Y	Y	Y	Y	Y	Y	Y						Y			Y				Y	Y	Y	Y						Y										
16093	A_23_P60148	PDGFRL																		Y																										
13234	A_23_P251095	PDHA1	Y	Y					Y	Y																																				
10318	A_23_P81026	PDHA2			Y	Y		Y		Y																	Y	Y	Y	Y						Y										
5955	A_23_P20932	PDHB			Y	Y		Y	Y	Y										Y			Y																							
17230	A_23_P36266	PDHX			Y				Y	Y										Y			Y				Y	Y	Y	Y						Y										
219	A_23_P325642	PDIA2	Y																																											
11893	A_23_P42800	PDIA4			Y	Y		Y		Y									Y	Y	Y		Y				Y																			
2291	A_23_P167040	PDIA5			Y	Y		Y		Y	Y	Y	Y	Y				Y		Y		Y					Y																			

7918	A_23_P56956	PDIA6			Y	Y		Y	Y	Y								Y		Y	Y									Y						Y										
9936	A_23_P51601	PDIK1L				Y			Y	Y																																				
3835	A_23_P10614	PDK1							Y	Y																																				
15413	A_23_P207517	PDK2																	Y																											
5786	A_23_P257087	PDK4																										Y																		
3320	A_23_P149987	PDLIM1							Y	Y																																				
11935	A_23_P20282	PDLIM2			Y				Y	Y	Y	Y	Y	Y						Y		Y	Y							Y						Y										
7175	A_23_P144896	PDLIM7																		Y		Y	Y																							
13541	A_23_P66219	PDPK1			Y																																									
18929	A_23_P430068	PDPN																	Y	Y																										
19961	A_23_P201322	PDPN	Y		Y				Y	Y										Y																										
8580	A_23_P144294	PDS5A																		Y																										
17419	A_23_P205098	PDS5B			Y	Y		Y	Y	Y									Y	Y							Y	Y	Y	Y						Y										
5866	A_23_P161152	PDSS1	Y	Y	Y	Y		Y		Y																	Y																			
7930	A_23_P134167	PDSS2			Y				Y	Y																																				
3929	A_23_P68730	PDXK			Y													Y	Y	Y		Y	Y	Y			Y			Y						Y										
18516	A_23_P211159	PDXK	Y	Y	Y	Y	Y	Y	Y	Y	Y	Y	Y	Y				Y		Y		Y	Y				Y	Y	Y	Y						Y										
11313	A_23_P155071	PDXP																		Y		Y	Y																							
902	A_23_P40262	PDYN																				Y																								
21261	A_23_P7402	PDZD2																		Y	Y																									
12489	A_23_P35974	PDZD3							Y	Y																																				
15018	A_23_P22717	PDZD4	Y	Y																																										
19151	A_23_P75097	PDZD7			Y	Y		Y	Y	Y	Y	Y	Y	Y														Y		Y						Y										
13558	A_23_P321452	PDZD8				Y					Y		Y	Y					Y	Y										Y						Y										
20538	A_23_P52121	PDZK1	Y	Y	Y	Y		Y	Y	Y	Y	Y	Y	Y				Y									Y	Y	Y	Y			Y			Y										
2621	A_23_P394304	PDZK1IP1			Y	Y	Y	Y	Y	Y	Y	Y															Y	Y	Y	Y																
15182	A_23_P160920	PDZK1IP1							Y	Y								Y		Y			Y					Y																		
21251	A_23_P11578	PEA15			Y	Y	Y	Y	Y	Y	Y	Y	Y	Y				Y	Y	Y		Y	Y	Y			Y	Y	Y	Y						Y										
6477	A_23_P46202	PEAR1							Y	Y																				Y						Y										
16822	A_23_P13609	PEBP1	Y	Y		Y														Y								Y		Y						Y										
22094	A_23_P157636	PEBP4	Y	Y																																										
4045	A_23_P252471	PECAM1	Y		Y				Y	Y	Y	Y	Y	Y	Y								Y				Y			Y						Y										
21151	A_23_P125025	PECAM1	Y		Y										Y			Y	Y	Y				Y			Y																			
20499	A_23_P156852	PECI				Y			Y	Y	Y	Y	Y	Y				Y	Y	Y		Y		Y				Y		Y						Y						Y				
3089	A_23_P313	PEF1																Y	Y	Y		Y	Y	Y			Y						Y													
20936	A_23_P120345	PELI1							Y	Y																				Y						Y										
18442	A_23_P65532	PELI2			Y	Y		Y	Y	Y								Y	Y	Y				Y			Y			Y						Y										
2635	A_23_P104694	PELI3			Y																																									
18621	A_23_P49898	PELP1																		Y																										
384	A_23_P163955	PEMT			Y	Y	Y	Y		Y	Y	Y								Y		Y					Y																			
6983	A_23_P83436	PEPD																		Y																										
20283	A_23_P209320	PER2			Y	Y	Y	Y	Y	Y	Y	Y	Y	Y				Y	Y	Y		Y		Y			Y	Y	Y	Y						Y						Y				
17364	A_23_P201461	PER3				Y			Y	Y																		Y		Y						Y										
18820	A_23_P207075	PERLD1							Y	Y								Y	Y	Y		Y		Y																						
2725	A_23_P369298	PERP																			Y																									
10853	A_23_P214950	PERP																		Y	Y																									
3144	A_23_P132260	PES1			Y	Y	Y	Y	Y	Y	Y	Y						Y	Y	Y		Y	Y	Y			Y	Y	Y	Y						Y										
9442	A_23_P92552	PET112L																Y				Y																								
4799	A_23_P20045	PEX1			Y				Y	Y	Y	Y	Y	Y					Y	Y	Y		Y					Y		Y						Y										
17031	A_23_P501745	PEX10			Y	Y		Y		Y										Y	Y	Y					Y																			
19216	A_23_P37560	PEX11A							Y	Y																	Y																			
3154	A_23_P101410	PEX11G				Y			Y	Y																				Y						Y										
4472	A_23_P321466	PEX11G							Y	Y																				Y						Y										
21887	A_23_P107149	PEX12																									Y																			
15547	A_23_P86810	PEX16			Y	Y		Y		Y									Y	Y		Y	Y				Y																			
1658	A_23_P259328	PEX3																		Y	Y		Y				Y																			
4635	A_23_P93543	PEX7	Y		Y	Y		Y	Y	Y	Y	Y	Y	Y						Y	Y		Y				Y			Y						Y										
664	A_23_P132987	PF4			Y	Y		Y	Y	Y	Y	Y	Y	Y														Y		Y						Y										
12154	A_23_P167150	PF4V1			Y	Y		Y	Y	Y																		Y		Y						Y										
20030	A_23_P51906	PFDN2							Y	Y																																				
232	A_23_P166023	PFDN4																												Y						Y										
1617	A_23_P122563	PFDN6				Y																																								
16337	A_23_P96362	PFKFB1			Y	Y		Y		Y																	Y	Y	Y	Y						Y										
2784	A_23_P255754	PFKFB3			Y	Y		Y		Y	Y	Y	Y	Y																																
10159	A_23_P29081	PFKL			Y													Y	Y	Y	Y	Y	Y	Y	Y	Y	Y				Y															
16616	A_23_P170848	PFKM			Y																						Y																			
102	A_23_P46928	PFKP			Y	Y		Y	Y	Y																		Y		Y						Y										
1098	A_23_P27094	PFN1			Y	Y	Y	Y	Y	Y	Y	Y	Y	Y				Y	Y	Y	Y	Y	Y	Y	Y	Y	Y	Y	Y	Y	Y	Y	Y	Y	Y	Y							Y			
15938	A_23_P145889	PFTK1																										Y																		
18339	A_23_P154050	PFTK2			Y																		Y					Y																		
1917	A_23_P138620	PGAM1			Y	Y		Y	Y	Y									Y	Y	Y	Y	Y																							
4529	A_23_P93942	PGAM2																		Y			Y																							
3983	A_23_P426396	PGAM5			Y	Y		Y	Y	Y								Y	Y	Y		Y		Y			Y																			
15900	A_23_P79247	PGAP1				Y																Y						Y																		
5154	A_23_P156625	PGBD1			Y	Y		Y	Y	Y													Y				Y			Y						Y										
16928	A_23_P334777	PGBD2			Y								Y							Y	Y		Y																							
19191	A_23_P319565	PGBD3			Y	Y		Y	Y	Y								Y	Y	Y		Y	Y	Y			Y			Y	Y	Y	Y	Y	Y	Y										

3641	A_23_P420331	PGBD4			Y	Y		Y	Y	Y																	Y	Y	Y	Y																
9822	A_23_P126648	PGBD5			Y	Y		Y	Y	Y	Y	Y	Y	Y				Y	Y	Y		Y	Y	Y			Y			Y						Y										
8204	A_23_P7963	PGC			Y				Y	Y										Y			Y				Y			Y						Y										
3001	A_23_P256735	PGCP				Y												Y												Y						Y										
14647	A_23_P126623	PGD			Y		Y				Y								Y	Y		Y	Y				Y																			
12499	A_23_P76992	PGF			Y	Y		Y	Y	Y								Y	Y	Y		Y	Y	Y			Y																			
20373	A_23_P256637	PGGT1B																									Y																			
8549	A_23_P125829	PGK1			Y	Y		Y	Y	Y											Y							Y																		
4536	A_23_P331063	PGK2							Y	Y												Y																								
8231	A_23_P153889	PGLS			Y	Y		Y	Y	Y			Y					Y	Y	Y	Y	Y	Y	Y	Y	Y	Y			Y	Y	Y				Y										
18101	A_23_P67669	PGLYRP2	Y		Y	Y		Y		Y																	Y	Y	Y	Y						Y										
20957	A_23_P96899	PGLYRP3			Y																						Y																			
5508	A_23_P7172	PGM2																		Y																										
9148	A_23_P10952	PGM5																										Y																		
22195	A_23_P167432	PGM5P2				Y																						Y		Y						Y										
2827	A_23_P66100	PGP			Y	Y		Y	Y	Y																				Y						Y										
3707	A_23_P218531	PGPEP1	Y	Y	Y				Y	Y	Y	Y	Y	Y						Y										Y						Y										
13196	A_23_P114275	PGRMC1																					Y																							
14695	A_23_P77833	PGS1			Y	Y	Y	Y	Y	Y	Y	Y	Y	Y				Y	Y	Y	Y	Y	Y	Y	Y	Y	Y				Y		Y	Y	Y											
11690	A_23_P28834	PHACTR3			Y	Y	Y	Y	Y	Y	Y	Y							Y	Y	Y	Y	Y				Y	Y	Y	Y												Y				
10450	A_23_P213908	PHAX				Y																																								
17203	A_23_P130040	PHB			Y														Y	Y			Y																							
12752	A_23_P323094	PHC1			Y				Y	Y	Y	Y	Y	Y								Y																								
21540	A_23_P204244	PHC1							Y	Y																				Y						Y										
9056	A_23_P34510	PHC2																Y	Y	Y			Y	Y																						
15401	A_23_P423866	PHC2			Y		Y				Y		Y	Y				Y	Y	Y	Y	Y	Y	Y	Y	Y	Y			Y	Y	Y				Y		Y								
7239	A_23_P409988	PHC3			Y								Y																	Y						Y										
8702	A_23_P114084	PHEX																		Y																										
756	A_23_P503200	PHF10																		Y																										
14868	A_23_P65174	PHF11			Y													Y	Y	Y		Y	Y	Y			Y																			
6833	A_23_P305761	PHF12																									Y																			
21370	A_23_P384559	PHF13																		Y																										
15600	A_23_P134384	PHF14																	Y	Y			Y																							
20319	A_23_P167256	PHF17				Y													Y	Y																										
17155	A_23_P21436	PHF19			Y	Y		Y		Y								Y	Y	Y	Y	Y	Y	Y	Y	Y	Y				Y															
17588	A_23_P502376	PHF2							Y	Y																				Y						Y										
5645	A_23_P134786	PHF20L1																	Y																											
16778	A_23_P216038	PHF20L1			Y	Y	Y	Y	Y	Y	Y	Y						Y	Y	Y		Y		Y				Y																		
9970	A_23_P202715	PHF21A							Y	Y												Y																								
21425	A_23_P359015	PHF21A							Y	Y									Y	Y		Y								Y						Y										
2177	A_23_P38478	PHF23	Y	Y																																										
7536	A_23_P70371	PHF3			Y	Y	Y	Y	Y	Y	Y	Y						Y	Y	Y		Y	Y	Y			Y	Y	Y	Y						Y										
14467	A_23_P253571	PHF5A																												Y						Y										
6213	A_23_P80684	PHF7				Y																																								
2305	A_23_P112991	PHF8																Y																												
17532	A_23_P85780	PHGDH			Y	Y		Y	Y	Y																	Y																			
18079	A_23_P145437	PHIP			Y	Y	Y	Y	Y	Y	Y	Y							Y	Y		Y						Y																		
18862	A_23_P159671	PHKA2			Y				Y	Y										Y							Y			Y						Y										
4709	A_23_P134653	PHKG1																		Y		Y	Y																							
15159	A_23_P304446	PHKG1			Y				Y	Y	Y	Y	Y	Y				Y	Y	Y		Y	Y	Y			Y			Y						Y										
13678	A_23_P76450	PHLDA1			Y																						Y	Y	Y	Y						Y										
9287	A_23_P62959	PHLDA3																Y		Y		Y	Y																							
2795	A_23_P24557	PHLDB1			Y				Y	Y										Y			Y				Y																			
18083	A_23_P250063	PHLDB2			Y	Y		Y	Y	Y								Y	Y	Y		Y		Y			Y			Y						Y										
11949	A_23_P165788	PHOSPHO2			Y	Y	Y	Y	Y	Y	Y	Y	Y	Y				Y	Y	Y	Y	Y	Y	Y	Y	Y	Y	Y	Y	Y	Y	Y				Y				Y						
6749	A_23_P75391	PHRF1																Y		Y	Y	Y																								
19149	A_23_P201179	PHTF1			Y	Y		Y	Y	Y									Y	Y		Y	Y																							
286	A_23_P433229	PHYHIP			Y														Y																											
8872	A_23_P71364	PI15			Y																						Y																			
19011	A_23_P102994	PI4KA			Y													Y		Y	Y	Y	Y																							
11547	A_23_P314725	PI4KB			Y														Y			Y																								
7625	A_23_P130316	PIAS2																Y	Y	Y			Y	Y																						
7283	A_23_P149678	PIAS3	Y	Y	Y																																									
21581	A_23_P90280	PIAS4			Y	Y		Y	Y	Y										Y		Y	Y				Y			Y						Y										
6619	A_23_P37026	PIBF1			Y	Y	Y	Y	Y	Y	Y	Y	Y					Y	Y	Y		Y	Y	Y			Y	Y	Y	Y																
15577	A_23_P411881	PIBF1			Y	Y	Y	Y	Y	Y	Y	Y						Y	Y	Y		Y	Y	Y			Y	Y	Y	Y																
6000	A_23_P203658	PICALM			Y																																									
20405	A_23_P147995	PICALM							Y	Y																				Y						Y										
17776	A_23_P91727	PICK1			Y	Y	Y	Y		Y	Y	Y						Y	Y	Y	Y	Y	Y	Y	Y	Y	Y	Y	Y	Y	Y	Y						Y				Y			Y	
17547	A_23_P21485	PID1			Y															Y		Y	Y				Y			Y						Y										
20959	A_23_P217399	PIGA																		Y			Y																							
6578	A_23_P77174	PIGB			Y														Y	Y			Y				Y																			
1108	A_23_P86250	PIGC																Y	Y	Y	Y	Y		Y									Y													
6706	A_23_P74852	PIGC																	Y	Y	Y																									
4447	A_23_P136544	PIGG			Y				Y	Y	Y	Y	Y	Y				Y		Y	Y	Y	Y				Y	Y	Y	Y						Y										
4239	A_23_P2884	PIGH			Y				Y	Y										Y							Y																			
2788	A_23_P38623	PIGL																		Y		Y	Y																							

9092	A_23_P103248	PIGM			Y																																								
6182	A_23_P78358	PIGN											Y					Y	Y	Y	Y	Y	Y	Y	Y	Y					Y														
19357	A_23_P502604	PIGQ																	Y				Y																						
21156	A_23_P140899	PIGQ			Y		Y		Y	Y	Y	Y	Y	Y				Y	Y	Y		Y		Y			Y			Y			Y			Y									
12093	A_23_P412343	PIGR							Y	Y																																			
11401	A_23_P66707	PIGS			Y															Y	Y	Y																							
12886	A_23_P79842	PIGT			Y																						Y			Y						Y									
21377	A_23_P46375	PIGV	Y	Y	Y				Y	Y								Y	Y	Y		Y	Y	Y			Y	Y	Y	Y						Y									
19273	A_23_P354170	PIGX																					Y																						
19504	A_23_P132975	PIGY																		Y								Y		Y						Y									
187	A_23_P418451	PIH1D2			Y	Y	Y	Y	Y	Y	Y	Y							Y	Y		Y	Y				Y	Y	Y	Y															
2341	A_23_P36166	PIK3C2A			Y																						Y			Y						Y									
10935	A_23_P200710	PIK3C2B							Y	Y																																			
581	A_23_P164536	PIK3C3			Y	Y		Y		Y								Y	Y	Y	Y	Y		Y																					
15218	A_23_P92057	PIK3CA							Y	Y																																			
15538	A_23_P346969	PIK3CB																					Y																						
15778	A_23_P92253	PIK3CB																		Y			Y																						
20832	A_23_P259833	PIK3CD	Y	Y	Y		Y		Y	Y	Y	Y	Y	Y				Y	Y	Y	Y		Y	Y	Y		Y			Y						Y									
12215	A_23_P109488	PIK3IP1			Y	Y		Y		Y								Y	Y	Y		Y		Y																					
10730	A_23_P144976	PIK3R1			Y																																								
12086	A_23_P142359	PIK3R2			Y													Y	Y	Y				Y																					
2042	A_23_P132525	PIK3R4			Y	Y		Y	Y	Y	Y	Y	Y	Y					Y	Y	Y	Y	Y				Y	Y	Y	Y						Y									
17306	A_23_P31224	PILRA			Y													Y	Y	Y	Y	Y	Y	Y	Y	Y	Y				Y														
7736	A_23_P19829	PILRB			Y	Y		Y	Y	Y												Y						Y																	
13041	A_23_P345118	PIM1	Y						Y	Y																																			
20692	A_23_P252343	PIM2	Y		Y	Y	Y	Y	Y	Y	Y	Y	Y	Y					Y	Y							Y	Y	Y	Y															
21386	A_23_P67162	PIN1			Y	Y	Y	Y		Y	Y	Y	Y					Y	Y	Y		Y	Y	Y			Y	Y	Y	Y						Y									
20349	A_23_P267	PIN1L			Y	Y		Y	Y	Y								Y		Y							Y																		
15125	A_23_P315345	PIN4			Y	Y		Y		Y								Y	Y	Y		Y	Y	Y			Y	Y	Y	Y															
10506	A_23_P23194	PINK1				Y																																							
19383	A_23_P82823	PINX1			Y													Y	Y																										
12892	A_23_P8702	PIP							Y	Y	Y	Y	Y	Y				Y	Y	Y		Y		Y			Y			Y						Y									
14864	A_23_P122694	PIP3-E							Y	Y													Y																						
13363	A_23_P104344	PIP4K2A			Y													Y	Y	Y	Y	Y	Y	Y	Y	Y					Y		Y												
16847	A_23_P162199	PIP4K2C																Y																											
11229	A_23_P368205	PIP5K1A			Y																																								
1608	A_23_P78950	PIP5K1C			Y														Y	Y	Y	Y	Y																						
8640	A_23_P164258	PIPOX							Y	Y	Y	Y	Y	Y														Y		Y						Y									
2150	A_23_P137035	PIR																		Y																									
17171	A_23_P77880	PITPNA			Y	Y		Y	Y	Y	Y	Y	Y	Y						Y	Y									Y						Y									
19314	A_23_P17786	PITPNB			Y																																								
4155	A_23_P66473	PITPNC1			Y	Y	Y	Y	Y	Y	Y	Y						Y	Y	Y	Y	Y	Y	Y	Y	Y	Y				Y														
7840	A_23_P374677	PITPNC1			Y	Y	Y	Y	Y	Y	Y	Y						Y	Y	Y	Y	Y	Y	Y	Y	Y	Y				Y														
19335	A_23_P1513	PITPNM1			Y	Y	Y	Y	Y	Y	Y	Y						Y		Y	Y	Y	Y				Y	Y	Y	Y															
21458	A_23_P403542	PITPNM2																				Y																							
9163	A_23_P66753	PITPNM3																			Y																								
6466	A_23_P52396	PITRM1			Y													Y					Y				Y	Y	Y	Y						Y									
21731	A_23_P58636	PITX1			Y															Y		Y					Y																		
18679	A_23_P104301	PITX3			Y	Y		Y	Y	Y										Y			Y				Y			Y						Y									
15044	A_23_P427760	PIWIL4	Y	Y																										Y						Y									
12379	A_23_P133470	PJA2			Y	Y	Y	Y		Y	Y	Y							Y	Y		Y	Y				Y	Y	Y	Y						Y									
21098	A_23_P77502	PKD1																Y	Y	Y	Y	Y	Y	Y	Y	Y					Y														
689	A_23_P318432	PKD1L1							Y	Y																				Y						Y									
4495	A_23_P129332	PKD1L2																	Y	Y		Y	Y				Y	Y	Y	Y															
2385	A_23_P12554	PKD2L1																									Y																		
22046	A_23_P120985	PKDREJ							Y	Y																																			
21157	A_23_P402187	PKHD1																												Y						Y									
16143	A_23_P94156	PKHD1L1																Y		Y	Y	Y																							
17297	A_23_P392485	PKHD1L1			Y	Y		Y	Y	Y	Y	Y	Y	Y				Y		Y							Y																		
17446	A_23_P31765	PKIA																					Y																						
17511	A_23_P145529	PKIB																		Y							Y																		
6265	A_23_P3258	PKM2			Y	Y	Y	Y		Y	Y	Y						Y	Y	Y	Y	Y		Y																					
19411	A_23_P206632	PKMYT1			Y	Y		Y	Y	Y								Y	Y	Y	Y	Y		Y			Y	Y	Y	Y						Y									
19802	A_23_P398523	PKMYT1			Y	Y	Y	Y	Y	Y	Y	Y						Y	Y	Y	Y	Y	Y	Y	Y	Y	Y			Y	Y	Y	Y			Y		Y				Y			Y	Y
10690	A_23_P67271	PKN1			Y	Y	Y	Y	Y	Y	Y	Y						Y	Y	Y	Y	Y	Y	Y	Y	Y	Y	Y	Y	Y	Y	Y				Y									
8	A_23_P23227	PKN2			Y	Y		Y		Y																																			
18794	A_23_P258982	PKN3			Y	Y		Y		Y								Y	Y	Y		Y	Y	Y			Y																		
18094	A_23_P211299	PKNOX1			Y	Y	Y	Y		Y	Y	Y	Y	Y					Y	Y	Y	Y	Y				Y																		
9103	A_23_P23296	PKP1			Y																																								
9087	A_23_P29816	PLA1A			Y													Y																											
3617	A_23_P88766	PLA2G10			Y				Y	Y	Y	Y	Y	Y													Y																		
14633	A_23_P30020	PLA2G12A			Y				Y	Y											Y		Y																						
11426	A_23_P3584	PLA2G15																		Y																									
5265	A_23_P116414	PLA2G16			Y	Y		Y	Y	Y	Y	Y	Y	Y													Y	Y	Y	Y						Y									
4336	A_23_P114857	PLA2G2E			Y	Y		Y		Y									Y	Y		Y								Y						Y									
5277	A_23_P200629	PLA2G2F				Y																								Y						Y									
10339	A_23_P17814	PLA2G3			Y	Y	Y	Y		Y	Y	Y	Y	Y													Y	Y	Y	Y						Y									

C10773.4	A_23_P11685	PLA2G4A							Y	Y																					Y						Y										
9206	A_23_P358528	PLA2G4D			Y															Y	Y		Y				Y																		
7570	A_23_P6464	PLA2G6							Y	Y																					Y						Y										
20112	A_23_P145096	PLA2G7	Y	Y	Y	Y		Y		Y					Y			Y		Y							Y			Y						Y										
3590	A_23_P148609	PLAC1																					Y																							
6635	A_23_P317820	PLAC1L																									Y																			
9851	A_23_P39294	PLAC2			Y																																									
20159	A_23_P359666	PLAC2			Y	Y		Y		Y								Y	Y	Y		Y		Y			Y	Y	Y	Y																
5044	A_23_P81219	PLAC8	Y		Y													Y	Y	Y		Y		Y			Y																			
19139	A_23_P411723	PLAG1																Y				Y																								
15880	A_23_P6151	PLAGL2			Y															Y		Y																								
8269	A_23_P82868	PLAT	Y	Y	Y	Y	Y	Y	Y	Y	Y	Y						Y	Y	Y		Y		Y			Y	Y	Y	Y						Y										
3064	A_23_P27867	PLAUR																		Y			Y																							
17205	A_23_P3468	PLCB2																		Y																										
22517	A_23_P343719	PLCB3			Y													Y	Y	Y	Y	Y	Y	Y	Y	Y	Y				Y		Y													
12542	A_23_P80739	PLCD1																		Y																										
21353	A_23_P351757	PLCD3																Y		Y	Y	Y	Y																							
14660	A_23_P385105	PLCD4	Y	Y	Y	Y	Y	Y	Y	Y	Y	Y	Y	Y						Y							Y	Y	Y	Y						Y										
14882	A_23_P165475	PLCD4				Y			Y	Y	Y	Y	Y	Y																Y						Y										
17762	A_23_P35617	PLCE1																									Y																			
4399	A_23_P254801	PLCG1																		Y	Y	Y																								
5941	A_23_P106675	PLCG2	Y	Y	Y	Y		Y	Y	Y									Y									Y		Y						Y										
16927	A_23_P80594	PLCL2	Y																											Y						Y										
19686	A_23_P155335	PLD1																												Y						Y										
17545	A_23_P27515	PLD3			Y	Y	Y	Y	Y	Y	Y	Y	Y	Y				Y	Y	Y	Y	Y	Y	Y	Y	Y	Y				Y		Y	Y	Y							Y				
11477	A_23_P88222	PLD4			Y	Y		Y	Y	Y										Y			Y				Y																			
72	A_23_P510	PLD5																			Y							Y																		
2145	A_23_P406889	PLDN																			Y																									
10834	A_23_P205808	PLDN																Y			Y																									
2169	A_23_P22112	PLEC1																					Y																							
9932	A_23_P209678	PLEK	Y	Y	Y		Y		Y	Y	Y	Y			Y					Y	Y						Y																			
8807	A_23_P151506	PLEK2																Y		Y																										
10822	A_23_P102419	PLEKHA3			Y															Y			Y																							
4504	A_23_P67361	PLEKHA4							Y	Y									Y	Y		Y	Y				Y	Y	Y	Y						Y										
20310	A_23_P218068	PLEKHA5			Y	Y	Y	Y	Y	Y	Y	Y	Y	Y					Y	Y		Y	Y				Y			Y						Y										
488	A_23_P148748	PLEKHA6																												Y						Y										
20105	A_23_P431268	PLEKHA6			Y				Y	Y	Y	Y	Y	Y						Y							Y																			
3471	A_23_P318296	PLEKHA8				Y																																								
18349	A_23_P93767	PLEKHA8																									Y																			
12249	A_23_P53211	PLEKHB1							Y	Y																				Y						Y										
8050	A_23_P28642	PLEKHB2			Y	Y	Y	Y		Y	Y	Y						Y	Y	Y	Y	Y		Y				Y																		
11693	A_23_P79133	PLEKHF1			Y	Y	Y	Y	Y	Y	Y	Y	Y	Y				Y	Y	Y	Y	Y	Y	Y	Y	Y	Y			Y	Y	Y				Y										
143	A_23_P20275	PLEKHF2							Y	Y	Y	Y	Y	Y														Y		Y						Y										
3709	A_23_P76901	PLEKHG3			Y				Y	Y					Y												Y																			
1052	A_23_P81640	PLEKHG4B																Y	Y																											
9277	A_23_P149626	PLEKHG5			Y																						Y																			
1649	A_23_P117335	PLEKHH1																				Y																								
9284	A_23_P164112	PLEKHM1			Y	Y		Y	Y	Y									Y	Y	Y	Y					Y																			
7732	A_23_P23617	PLEKHN1																			Y		Y																							
20609	A_23_P35114	PLEKHO1	Y		Y		Y		Y	Y	Y	Y	Y	Y	Y			Y	Y	Y	Y	Y	Y	Y	Y	Y	Y				Y															
20853	A_23_P129246	PLEKHO2	Y		Y	Y	Y	Y	Y	Y	Y	Y	Y	Y	Y					Y	Y		Y					Y		Y	Y	Y	Y	Y	Y	Y										
6252	A_23_P30693	PLG			Y																						Y																			
21695	A_23_P359630	PLGLB1			Y	Y	Y	Y	Y	Y	Y	Y			Y				Y			Y						Y																		
7846	A_23_P26153	PLIN																		Y			Y																							
9423	A_23_P118174	PLK1			Y	Y		Y	Y	Y	Y	Y	Y	Y				Y	Y	Y		Y		Y			Y	Y	Y	Y						Y										
16949	A_23_P30254	PLK2			Y	Y		Y	Y	Y	Y	Y	Y	Y					Y	Y		Y	Y				Y	Y	Y	Y						Y										
20187	A_23_P51646	PLK3																		Y			Y																							
11430	A_23_P43763	PLLP	Y	Y		Y			Y	Y																																				
21979	A_23_P30614	PLN													Y												Y																			
5336	A_23_P137525	PLOD1							Y	Y	Y	Y	Y	Y													Y			Y						Y										
15495	A_23_P211910	PLOD2							Y	Y																																				
20531	A_23_P168788	PLOD3							Y	Y	Y	Y	Y	Y						Y	Y	Y																								
2454	A_23_P85201	PLP1	Y		Y																						Y																			
16383	A_23_P84775	PLRG1																		Y			Y							Y						Y										
13330	A_23_P211900	PLS1			Y																																									
13263	A_23_P250607	PLS3			Y				Y	Y	Y	Y	Y	Y													Y	Y	Y	Y						Y								Y		
15758	A_23_P69109	PLSCR1																		Y																										
2210	A_23_P49597	PLSCR3																					Y				Y																			
16971	A_23_P91912	PLSCR4																	Y											Y						Y										
5357	A_23_P5983	PLTP	Y		Y	Y	Y	Y		Y	Y	Y	Y	Y	Y			Y	Y	Y	Y	Y	Y	Y	Y	Y	Y			Y	Y	Y	Y	Y	Y	Y										
21518	A_23_P56328	PLVAP	Y		Y	Y	Y	Y	Y	Y	Y	Y						Y		Y	Y	Y	Y				Y			Y			Y			Y										
11820	A_23_P161422	PLXDC2	Y																																											
13962	A_23_P57674	PLXNA1			Y															Y	Y	Y	Y				Y																			
13312	A_23_P46618	PLXNA2																									Y																			
22520	A_23_P307540	PLXNA2																	Y																											
2264	A_23_P11309	PLXNA3																			Y																									
6407	A_23_P111448	PLXNA4			Y				Y	Y																	Y			Y						Y										

7835	A_23_P60714	PLXNB2			Y	Y		Y		Y								Y	Y	Y	Y	Y	Y	Y	Y	Y	Y				Y															
8060	A_23_P137163	PLXNB3			Y																		Y																							
21710	A_23_P80752	PLXND1			Y													Y		Y	Y	Y	Y										Y													
C10565.5	A_23_P207999	PMAIP1			Y				Y	Y										Y	Y		Y				Y																			
18917	A_23_P170378	PMCHL1			Y	Y		Y	Y	Y																	Y																			
13521	A_23_P57089	PMEPA1			Y																																									
3581	A_23_P635	PMF1			Y	Y		Y		Y												Y					Y																			
2686	A_23_P205961	PML				Y																	Y																							
2782	A_23_P211598	PMM1			Y	Y	Y	Y	Y	Y	Y	Y						Y	Y	Y		Y	Y	Y			Y			Y						Y										
2566	A_23_P432360	PMM2			Y	Y		Y		Y								Y	Y	Y		Y		Y			Y																			
19326	A_23_P31615	PMP2																											Y						Y										
5588	A_23_P100711	PMP22							Y	Y									Y				Y				Y																			
17976	A_23_P32125	PMPCA			Y	Y		Y		Y									Y	Y		Y	Y				Y																			
13014	A_23_P134464	PMPCB			Y	Y		Y		Y										Y		Y	Y				Y																			
21347	A_23_P40063	PMS1			Y	Y		Y	Y	Y										Y			Y																							
10998	A_23_P6582	PMS2CL	Y	Y	Y				Y	Y	Y	Y	Y	Y				Y		Y	Y		Y				Y			Y	Y	Y	Y	Y	Y	Y		Y				Y			Y	
5986	A_23_P62383	PNCK																			Y																									
14905	A_23_P164883	PNKP																Y		Y		Y	Y																							
3888	A_23_P46879	PNLIP			Y	Y		Y		Y																	Y																			
18511	A_23_P24083	PNLIPRP2			Y	Y		Y	Y	Y																																				
6017	A_23_P52346	PNLIPRP3			Y																						Y																			
3083	A_23_P99771	PNMA1			Y				Y	Y																	Y	Y	Y	Y						Y										
10287	A_23_P112691	PNMA2			Y																																									
12828	A_23_P22422	PNMA3			Y				Y	Y																				Y						Y										
7728	A_23_P352950	PNMA5			Y	Y		Y	Y	Y										Y		Y	Y				Y			Y						Y										
3331	A_23_P84892	PNMA6A			Y		Y				Y									Y							Y																			
18473	A_23_P4951	PNMAL1							Y	Y																	Y																			
21838	A_23_P100642	PNMT							Y	Y	Y	Y	Y	Y																																
12992	A_23_P99582	PNN			Y	Y	Y	Y	Y	Y	Y	Y							Y	Y		Y	Y				Y	Y	Y	Y																
19801	A_23_P253321	PNOC	Y	Y	Y	Y	Y	Y	Y	Y	Y	Y															Y																			
13155	A_23_P10076	PNPLA2			Y				Y	Y								Y	Y	Y	Y	Y		Y																						
2542	A_23_P17914	PNPLA3	Y																																											
3994	A_23_P159797	PNPLA4																		Y																										
20523	A_23_P16163	PNPLA6			Y	Y		Y		Y										Y		Y	Y				Y																			
16206	A_23_P43455	PNPLA7																										Y																		
10156	A_23_P312718	PNPLA8																Y	Y	Y		Y	Y	Y																						
14983	A_23_P145074	PNRC1							Y	Y																				Y						Y										
2687	A_23_P103201	PNRC2			Y	Y	Y	Y	Y	Y	Y	Y	Y	Y					Y	Y			Y				Y	Y	Y	Y						Y										
2208	A_23_P368154	PODN			Y				Y	Y					Y			Y	Y	Y		Y	Y	Y																						
11798	A_23_P137446	PODN																					Y																							
13341	A_23_P121033	PODXL2																		Y	Y	Y	Y																							
17026	A_23_P159764	POF1B			Y	Y		Y	Y	Y																	Y	Y	Y	Y																
21843	A_23_P370483	POFUT1			Y	Y		Y	Y	Y									Y								Y	Y	Y	Y																
22565	A_23_P211227	POFUT2			Y	Y	Y	Y	Y	Y	Y	Y	Y	Y				Y	Y	Y	Y	Y	Y	Y	Y	Y	Y	Y	Y	Y	Y	Y	Y			Y										
14797	A_23_P137715	POGK																		Y			Y																							
22289	A_23_P63219	POGZ	Y	Y	Y																						Y	Y	Y	Y																
2074	A_23_P94980	POLA1				Y																																								
22033	A_23_P50456	POLD1			Y													Y	Y	Y				Y			Y																			
12673	A_23_P71140	POLD2			Y	Y	Y	Y		Y	Y	Y						Y	Y	Y		Y	Y	Y			Y														Y				
9293	A_23_P127367	POLD4							Y	Y																																				
10254	A_23_P360215	POLD4			Y				Y	Y									Y	Y	Y	Y	Y																							
17652	A_23_P100892	POLDIP2			Y	Y		Y	Y	Y								Y	Y	Y		Y		Y			Y																			
870	A_23_P502269	POLDIP3			Y	Y		Y	Y	Y								Y	Y	Y		Y	Y	Y			Y	Y	Y	Y						Y										
10983	A_23_P159355	POLE				Y																								Y						Y										
11091	A_23_P170751	POLE																	Y	Y			Y				Y			Y						Y										
11305	A_23_P135323	POLE3			Y																																									
8617	A_23_P383385	POLE4			Y	Y		Y		Y																	Y	Y	Y	Y																
13141	A_23_P154234	POLE4			Y	Y		Y		Y																		Y																		
3560	A_23_P3355	POLG							Y	Y																																				
15818	A_23_P26905	POLG2							Y	Y																		Y																		
6537	A_23_P145191	POLH	Y		Y	Y	Y	Y	Y	Y	Y	Y	Y	Y				Y	Y	Y	Y	Y	Y	Y	Y	Y	Y	Y	Y	Y	Y	Y				Y										
13315	A_23_P422714	POLH																									Y																			
11003	A_23_P306890	POLI			Y	Y	Y	Y	Y	Y	Y	Y	Y	Y				Y	Y	Y		Y	Y	Y			Y	Y	Y	Y						Y										
4513	A_23_P386450	POLK			Y	Y	Y	Y		Y	Y	Y							Y	Y		Y	Y				Y	Y	Y	Y						Y										
11019	A_23_P92773	POLK			Y	Y	Y	Y	Y	Y	Y	Y	Y	Y				Y	Y	Y		Y	Y	Y			Y	Y	Y	Y						Y										
18093	A_23_P397371	POLM			Y	Y		Y	Y	Y								Y		Y	Y	Y	Y				Y			Y						Y										
20622	A_23_P209987	POLR1B																		Y	Y		Y																							
14574	A_23_P70409	POLR1C			Y	Y	Y	Y		Y	Y	Y																																		
9954	A_23_P36924	POLR1D																		Y																										
17736	A_23_P9455	POLR1E																	Y			Y																								
5505	A_23_P141235	POLR2A	Y	Y	Y	Y		Y		Y	Y	Y	Y	Y				Y									Y	Y	Y	Y																
6125	A_23_P3527	POLR2C			Y	Y		Y		Y										Y																										
14439	A_23_P209778	POLR2D			Y	Y		Y	Y	Y								Y	Y	Y	Y	Y	Y	Y	Y	Y	Y	Y	Y	Y	Y	Y				Y				Y						
20146	A_23_P165280	POLR2E			Y	Y	Y	Y	Y	Y	Y	Y	Y	Y						Y	Y	Y					Y	Y	Y	Y						Y										
12705	A_23_P80321	POLR2F			Y	Y		Y	Y	Y								Y	Y	Y		Y		Y			Y	Y	Y	Y						Y										
14452	A_23_P42649	POLR2J																		Y																										
19647	A_23_P215259	POLR2J2			Y	Y		Y		Y																	Y																			

20047	A_23_P157452	POLR2K			Y																						Y																			
4252	A_23_P33022	POLR2L			Y	Y		Y	Y	Y								Y	Y	Y	Y	Y	Y	Y	Y	Y	Y			Y	Y	Y				Y										
8894	A_23_P87730	POLR3B			Y	Y	Y	Y	Y	Y	Y	Y	Y	Y						Y							Y																			
21378	A_23_P84565	POLR3D			Y	Y		Y	Y	Y									Y	Y	Y	Y	Y				Y	Y	Y	Y																
11260	A_23_P152181	POLR3E	Y		Y																						Y																			
10093	A_23_P166121	POLR3F							Y	Y										Y																										
13928	A_23_P341378	POLR3H			Y	Y	Y	Y		Y	Y	Y						Y	Y	Y		Y		Y			Y	Y	Y	Y						Y										
16157	A_23_P152297	POLR3K			Y																																									
18772	A_23_P131096	POLRMT																		Y		Y																								
544	A_23_P18823	POLS																									Y																			
2199	A_23_P311094	POM121L1P			Y				Y	Y													Y				Y																			
16891	A_23_P5875	POMC			Y	Y		Y		Y																																				
12061	A_23_P65250	POMP			Y	Y		Y		Y																																				
9160	A_23_P146361	POMT1																			Y																									
763	A_23_P151770	POMT2				Y														Y		Y																								
2328	A_23_P168584	POMZP3			Y	Y		Y		Y								Y		Y		Y	Y				Y																			
5897	A_23_P31399	PON2							Y	Y	Y	Y	Y	Y				Y	Y	Y	Y	Y	Y	Y	Y	Y	Y	Y	Y	Y	Y	Y														
17837	A_23_P215549	PON3																Y	Y	Y				Y						Y						Y										
289	A_23_P94133	POP1			Y																																									
6431	A_23_P90533	POP4	Y	Y	Y	Y		Y		Y																	Y	Y	Y	Y						Y										
20467	A_23_P134274	POP7			Y	Y	Y	Y		Y	Y	Y	Y														Y	Y	Y	Y																
3318	A_23_P19964	POR			Y	Y	Y	Y	Y	Y	Y	Y						Y	Y	Y			Y	Y			Y	Y	Y	Y						Y										
15353	A_23_P205111	POSTN																	Y	Y								Y																		
4415	A_23_P84610	POT1			Y	Y		Y		Y										Y			Y																							
531	A_23_P420348	POTED			Y	Y	Y	Y	Y	Y	Y	Y	Y	Y													Y	Y	Y	Y						Y										
4267	A_23_P312924	POU2AF1			Y		Y		Y	Y	Y	Y	Y					Y	Y								Y																			
15020	A_23_P161336	PPA1			Y																																									
21410	A_23_P27493	PPAN			Y	Y		Y	Y	Y	Y	Y	Y	Y						Y		Y	Y				Y	Y	Y	Y						Y										
19150	A_23_P4696	PPAN-P2RY11				Y			Y	Y									Y	Y		Y	Y																							
6981	A_23_P70060	PPAP2A																									Y																			
3766	A_23_P108294	PPAP2C			Y	Y	Y	Y	Y	Y	Y	Y	Y	Y				Y		Y		Y					Y																			
8839	A_23_P216128	PPAPDC1B				Y																																								
16327	A_23_P157736	PPAPDC3																					Y																							
6545	A_23_P40724	PPARA							Y	Y																				Y						Y										
15635	A_23_P306730	PPARA							Y	Y								Y		Y		Y								Y						Y										
12346	A_23_P18443	PPARGC1A																Y																												
13455	A_23_P213959	PPARGC1B			Y	Y		Y		Y										Y		Y	Y				Y																			
4105	A_23_P80940	PPAT				Y			Y	Y																																				
6851	A_23_P121596	PPBP			Y	Y		Y		Y																																				
2038	A_23_P92403	PPBPL2							Y	Y																				Y						Y										
22476	A_23_P163428	PPCDC				Y												Y	Y	Y		Y		Y			Y	Y	Y	Y																
20650	A_23_P51627	PPCS			Y	Y		Y	Y	Y										Y			Y																							
8261	A_23_P125502	PPEF1																		Y																										
4559	A_23_P167061	PPEF2			Y				Y	Y																	Y																			
1001	A_23_P302116	PPFIA1							Y	Y																																				
15325	A_23_P75509	PPFIA1			Y	Y		Y		Y									Y								Y																			
16177	A_23_P402936	PPFIA2																									Y																			
11907	A_23_P47867	PPFIBP1			Y														Y																											
9385	A_23_P150807	PPFIBP2			Y																																									
21193	A_23_P26233	PPIB			Y	Y	Y	Y	Y	Y	Y	Y	Y	Y				Y	Y	Y		Y		Y			Y																			
439	A_23_P74518	PPIE																			Y																									
5298	A_23_P313939	PPIF																									Y			Y						Y										
18416	A_23_P202104	PPIF																									Y																			
3117	A_23_P154411	PPIG				Y																						Y																		
10557	A_23_P17743	PPIL2																		Y		Y	Y																							
22376	A_23_P28213	PPIL3																		Y																										
6445	A_23_P42507	PPIL4			Y	Y	Y	Y	Y	Y	Y	Y							Y	Y		Y	Y				Y	Y	Y	Y						Y										
1907	A_23_P111361	PPIL6																		Y			Y				Y																			
1759	A_23_P151679	PPM1A			Y	Y	Y	Y	Y	Y	Y	Y	Y	Y				Y	Y	Y		Y		Y			Y	Y	Y	Y						Y										
5668	A_23_P209269	PPM1B			Y	Y		Y	Y	Y								Y	Y	Y		Y	Y	Y			Y			Y						Y										
8063	A_23_P209471	PPM1G			Y													Y	Y	Y			Y	Y			Y																			
6942	A_23_P258393	PPM1M			Y				Y	Y										Y																										
9692	A_23_P64567	PPME1																									Y																			
20025	A_23_P323227	PPP1CA			Y	Y	Y	Y	Y	Y	Y	Y						Y	Y	Y	Y	Y	Y	Y	Y	Y	Y	Y	Y	Y	Y	Y				Y										
15702	A_23_P83414	PPP1CB			Y	Y		Y		Y																	Y																			
14626	A_23_P19438	PPP1R11			Y	Y	Y	Y		Y	Y	Y						Y	Y	Y	Y	Y	Y	Y	Y	Y	Y	Y	Y	Y	Y	Y	Y													
167	A_23_P201790	PPP1R12B	Y	Y	Y	Y		Y		Y																	Y	Y	Y	Y						Y										
9820	A_23_P50575	PPP1R12C			Y	Y		Y	Y	Y								Y	Y	Y	Y	Y	Y	Y	Y	Y	Y			Y	Y	Y				Y										
13887	A_23_P130845	PPP1R14A							Y	Y									Y			Y	Y							Y						Y										
14539	A_23_P365821	PPP1R14B			Y	Y	Y	Y	Y	Y	Y	Y	Y	Y				Y	Y	Y		Y	Y	Y			Y	Y	Y	Y						Y										
426	A_23_P257617	PPP1R14C			Y				Y	Y	Y	Y	Y	Y																Y						Y										
22194	A_23_P45011	PPP1R14C			Y				Y	Y	Y	Y	Y	Y																Y						Y										
8563	A_23_P90172	PPP1R15A			Y	Y		Y		Y								Y		Y		Y	Y							Y						Y										
9718	A_23_P129835	PPP1R1B			Y														Y								Y	Y	Y	Y																
10154	A_23_P390445	PPP1R1C																									Y																			
1849	A_23_P17975	PPP1R2			Y																																									
10818	A_23_P34007	PPP1R2P9			Y																						Y			Y						Y										

16268	A_23_P216199	PPP1R3B																	Y																											
16174	A_23_P35414	PPP1R3C			Y	Y	Y	Y	Y	Y	Y	Y	Y	Y				Y	Y	Y	Y	Y	Y	Y	Y	Y	Y	Y	Y	Y	Y	Y				Y										
8956	A_23_P125533	PPP1R3F				Y																																								
14454	A_23_P12272	PPP1R8			Y	Y		Y		Y								Y	Y	Y			Y	Y			Y																			
20014	A_23_P145824	PPP1R9A			Y	Y	Y	Y		Y	Y	Y						Y	Y	Y	Y	Y	Y	Y	Y	Y	Y	Y	Y	Y	Y	Y						Y				Y			Y	
4759	A_23_P55242	PPP1R9B																		Y			Y																							
22023	A_23_P122036	PPP2CA				Y																																								
7021	A_23_P107661	PPP2R1A			Y													Y	Y	Y	Y	Y	Y	Y	Y	Y	Y				Y															
6491	A_23_P52793	PPP2R1B																Y		Y			Y																							
13265	A_23_P123539	PPP2R2A			Y				Y	Y													Y																							
17542	A_23_P213620	PPP2R2B																												Y						Y										
16481	A_23_P155660	PPP2R2C																Y	Y	Y				Y																						
1493	A_23_P158223	PPP2R2D																		Y			Y				Y																			
6945	A_23_P135778	PPP2R2D																		Y			Y							Y						Y										
8585	A_23_P253105	PPP2R2D																					Y																							
12563	A_23_P10401	PPP2R3A																					Y																							
9025	A_23_P45517	PPP2R3B			Y	Y	Y	Y	Y	Y	Y	Y						Y	Y	Y		Y	Y	Y			Y	Y	Y	Y						Y										
17784	A_23_P88201	PPP2R3C	Y	Y																																										
21175	A_23_P60458	PPP2R4			Y													Y		Y		Y																								
9449	A_23_P256432	PPP2R5A			Y																																									
3468	A_23_P35796	PPP2R5B			Y	Y		Y	Y	Y								Y	Y	Y	Y	Y	Y	Y	Y	Y	Y				Y		Y													
3857	A_23_P205236	PPP2R5C																		Y																										
10302	A_23_P167956	PPP2R5D			Y	Y	Y	Y	Y	Y	Y	Y	Y	Y				Y	Y	Y	Y	Y	Y	Y	Y	Y	Y	Y	Y	Y	Y	Y	Y			Y										
3628	A_23_P3042	PPP2R5E				Y														Y		Y						Y																		
15480	A_23_P92623	PPP3CA				Y																																								
14104	A_23_P202296	PPP3CB			Y	Y		Y	Y	Y										Y																										
5944	A_23_P100355	PPP4C			Y	Y	Y	Y	Y	Y	Y	Y						Y	Y	Y	Y	Y	Y	Y	Y	Y	Y				Y											Y				
15483	A_23_P15857	PPP4R1			Y																									Y						Y										
4165	A_23_P41159	PPP4R2																					Y																							
5454	A_23_P205367	PPP4R4	Y																	Y		Y	Y										Y													
13899	A_23_P153581	PPP5C			Y		Y				Y							Y	Y	Y		Y	Y	Y			Y																			
3651	A_23_P62659	PPT1			Y	Y		Y	Y	Y																	Y	Y	Y	Y																
2313	A_23_P110433	PPWD1			Y	Y	Y	Y	Y	Y	Y	Y	Y	Y				Y	Y	Y		Y	Y	Y			Y	Y	Y	Y						Y										
2393	A_23_P207336	PPY				Y																					Y																			
20868	A_23_P164253	PPY2			Y	Y		Y	Y	Y									Y								Y	Y	Y	Y						Y										
5718	A_23_P86726	PPYR1			Y	Y		Y	Y	Y										Y		Y	Y				Y			Y						Y										
20425	A_23_P62136	PQBP1			Y	Y		Y	Y	Y																	Y	Y	Y	Y																
12511	A_23_P118916	PQLC1				Y			Y	Y			Y					Y		Y		Y	Y							Y						Y										
21614	A_23_P15621	PRAC			Y	Y		Y		Y																		Y																		
2436	A_23_P250212	PRAGMIN																										Y																		
9839	A_23_P165219	PRAM1			Y																																									
22122	A_23_P166360	PRAME			Y				Y	Y																	Y																			
5937	A_23_P97465	PRAMEF2																					Y																							
4726	A_23_P126660	PRAMEF8			Y	Y		Y	Y	Y										Y		Y	Y				Y	Y	Y	Y						Y										
17954	A_23_P206059	PRC1																	Y	Y																										
12038	A_23_P93584	PRDM1			Y				Y	Y								Y		Y			Y				Y																			
12095	A_23_P350451	PRDM1			Y				Y	Y																	Y																			
7533	A_23_P35885	PRDM10			Y	Y	Y	Y	Y	Y	Y	Y						Y	Y	Y	Y	Y	Y	Y	Y	Y	Y	Y	Y	Y	Y	Y	Y	Y	Y	Y										
18583	A_23_P13057	PRDM11				Y														Y																										
14152	A_23_P43317	PRDM12																Y																												
7507	A_23_P433698	PRDM15																Y		Y																										
1784	A_23_P125972	PRDM16				Y																								Y						Y										
11504	A_23_P201071	PRDM2			Y	Y		Y		Y								Y	Y	Y	Y	Y		Y																						
21048	A_23_P117145	PRDM4			Y	Y		Y	Y	Y																	Y																			
7747	A_23_P81047	PRDM5				Y																																								
18539	A_23_P255328	PRDM8				Y																																								
19086	A_23_P11995	PRDX1			Y				Y	Y																	Y	Y	Y	Y																
8566	A_23_P63751	PRDX3			Y				Y	Y																																				
10881	A_23_P114232	PRDX4	Y	Y	Y	Y	Y	Y	Y	Y	Y	Y	Y	Y	Y			Y	Y	Y	Y	Y		Y			Y	Y	Y	Y			Y	Y		Y										
22018	A_23_P12989	PRDX5			Y	Y	Y	Y	Y	Y	Y	Y	Y	Y						Y		Y	Y				Y	Y	Y	Y																
948	A_23_P79161	PRELID1			Y	Y	Y	Y	Y	Y	Y	Y						Y	Y	Y	Y	Y	Y	Y	Y	Y	Y	Y	Y	Y	Y	Y				Y										
6632	A_23_P133755	PREP				Y			Y	Y																		Y																		
6554	A_23_P165325	PREPL	Y	Y	Y	Y	Y	Y		Y	Y	Y	Y	Y									Y																							
19203	A_23_P413641	PREX1																									Y																			
5204	A_23_P1473	PRF1			Y													Y	Y	Y			Y	Y			Y																			
2989	A_23_P218009	PRG2			Y	Y		Y	Y	Y																	Y																			
17105	A_23_P67567	PRG2	Y	Y	Y				Y	Y																				Y						Y										
7172	A_23_P160286	PRG4									Y		Y	Y																																
18986	A_23_P99076	PRH2							Y	Y																																				
13121	A_23_P79784	PRIC285			Y				Y	Y									Y	Y		Y	Y				Y																			
7374	A_23_P408285	PRICKLE1			Y															Y							Y																			
923	A_23_P348684	PRICKLE3																		Y																										
21059	A_23_P61009	PRIM2			Y				Y	Y			Y										Y				Y			Y						Y										
4426	A_23_P502808	PRIMA1			Y	Y		Y	Y	Y																	Y			Y						Y										
6567	A_23_P52109	PRKAA2			Y				Y	Y								Y	Y	Y	Y	Y	Y	Y	Y	Y	Y			Y	Y	Y	Y	Y	Y	Y										
9971	A_23_P60811	PRKAA2	Y	Y	Y	Y		Y	Y	Y	Y	Y	Y	Y	Y			Y	Y	Y		Y	Y	Y			Y	Y	Y	Y	Y	Y	Y	Y	Y	Y										
4234	A_23_P204503	PRKAB1			Y																						Y																			

254	A_23_P107865	PRKACA			Y																Y	Y	Y	Y	Y				Y																		
4369	A_23_P36513	PRKAG1			Y				Y	Y										Y		Y					Y																		
8680	A_23_P44366	PRKAG2																					Y							Y						Y										
22262	A_23_P314760	PRKAG2							Y	Y								Y		Y										Y						Y										
17667	A_23_P28258	PRKAG3																Y																												
2247	A_23_P164170	PRKAR1A			Y				Y	Y																																				
13386	A_23_P433753	PRKAR1A			Y	Y		Y	Y	Y																																				
10573	A_23_P83599	PRKAR1B																		Y		Y	Y																							
2928	A_23_P22214	PRKAR2A																									Y																			
11491	A_23_P42975	PRKAR2B			Y	Y		Y	Y	Y	Y	Y	Y	Y				Y		Y	Y		Y				Y																	Y		
7993	A_23_P55099	PRKCA			Y																						Y																			
10499	A_23_P420281	PRKCB																Y																												
18142	A_23_P144054	PRKCD			Y													Y	Y	Y	Y	Y	Y	Y	Y	Y	Y				Y															
9338	A_23_P203475	PRKCDBP																		Y			Y																							
21872	A_23_P250564	PRKCE																		Y																										
7942	A_23_P205567	PRKCH							Y	Y																																				
13427	A_23_P1374	PRKCQ																	Y																											
1964	A_23_P208615	PRKCSH			Y													Y	Y	Y	Y	Y	Y	Y	Y	Y	Y				Y															
20909	A_23_P51187	PRKCZ			Y																																									
12928	A_23_P78807	PRKD2			Y															Y	Y	Y																								
12398	A_23_P108574	PRKD3							Y	Y																																				
14450	A_23_P254955	PRKG1																	Y	Y		Y																								
19305	A_23_P136041	PRKG1																	Y																											
14433	A_23_P69663	PRKG2																	Y			Y																								
21865	A_23_P91019	PRKRA			Y				Y	Y									Y	Y	Y	Y	Y				Y			Y						Y										
8689	A_23_P203686	PRKRIR				Y			Y	Y										Y																										
18828	A_23_P217339	PRKX	Y	Y	Y	Y		Y	Y	Y	Y	Y	Y	Y									Y				Y			Y			Y	Y		Y										
17492	A_23_P137248	PRKY			Y				Y	Y	Y	Y	Y	Y									Y				Y			Y						Y										
13359	A_23_P143006	PRLH			Y															Y		Y	Y				Y																			
13883	A_23_P100189	PRM1			Y	Y	Y	Y		Y	Y	Y						Y		Y		Y																		Y				
8265	A_23_P106600	PRM2			Y	Y	Y	Y	Y	Y	Y	Y							Y	Y			Y				Y	Y	Y	Y																
21806	A_23_P39088	PRMT1			Y	Y		Y		Y								Y	Y	Y		Y	Y	Y			Y																			
19237	A_23_P211247	PRMT2			Y		Y				Y		Y	Y				Y		Y		Y	Y				Y			Y						Y										
3568	A_23_P9894	PRMT3			Y													Y		Y			Y				Y			Y						Y										
9525	A_23_P76851	PRMT5																Y	Y	Y	Y	Y		Y																						
14958	A_23_P12336	PRMT6				Y			Y	Y										Y			Y																							
20374	A_23_P139734	PRMT8																		Y																										
17537	A_23_P109143	PRNP			Y				Y	Y	Y	Y	Y	Y														Y		Y						Y						Y				
16613	A_23_P16694	PRO0132			Y	Y		Y	Y	Y								Y	Y	Y		Y	Y	Y			Y	Y	Y	Y	Y	Y	Y	Y	Y											
22030	A_23_P62793	PRO2012																												Y						Y										
9995	A_23_P430156	PRO2964			Y																						Y			Y						Y										
3907	A_23_P40100	PROC	Y	Y																Y			Y							Y						Y										
3879	A_23_P427148	PROCA1			Y				Y	Y																	Y			Y						Y										
4707	A_23_P80040	PROCR																		Y																										
1756	A_23_P68777	PRODH				Y			Y	Y													Y																							
13295	A_23_P165196	PRODH2	Y	Y	Y													Y	Y								Y																			
2241	A_23_P51745	PROK1			Y	Y		Y		Y										Y							Y	Y	Y	Y																
19360	A_23_P416089	PROKR1			Y	Y		Y	Y	Y																	Y																			
5332	A_23_P412603	PROKR2				Y			Y	Y																		Y																		
7038	A_23_P258462	PROM1																												Y						Y										
4598	A_23_P135848	PROP1			Y				Y	Y																	Y	Y	Y	Y						Y										
11080	A_23_P84510	PROS1			Y																									Y						Y										
16489	A_23_P137634	PROX1																Y				Y					Y																			
12744	A_23_P140082	PROZ																		Y			Y																							
14971	A_23_P97572	PRPF3																		Y			Y																							
19957	A_23_P142018	PRPF31			Y													Y	Y	Y		Y		Y			Y																			
5952	A_23_P201565	PRPF38B			Y	Y	Y	Y	Y	Y	Y	Y						Y	Y	Y		Y	Y	Y			Y	Y	Y	Y						Y										
20315	A_23_P163107	PRPF39			Y	Y	Y	Y	Y	Y	Y	Y							Y	Y		Y						Y																		
798	A_23_P60537	PRPF4																		Y			Y																							
3783	A_23_P16762	PRPF40A							Y	Y																																				
19743	A_23_P168236	PRPF4B			Y	Y		Y	Y	Y										Y		Y	Y							Y						Y										
13814	A_23_P154539	PRPF6																		Y	Y	Y																								
17323	A_23_P38219	PRPF8																		Y		Y																								
1439	A_23_P13713	PRPH																	Y																											
10353	A_23_P214459	PRPH2																										Y																		
332	A_23_P72961	PRPS1																		Y		Y																								
15400	A_23_P418516	PRPS1L1			Y																																									
1455	A_23_P96641	PRPS2							Y	Y																																				
17613	A_23_P49646	PRPSAP2			Y																																									
4553	A_23_P207301	PRR11			Y	Y		Y	Y	Y	Y	Y	Y	Y														Y		Y						Y										
2966	A_23_P55948	PRR12			Y	Y		Y		Y																																				
17404	A_23_P139575	PRR13			Y				Y	Y																				Y						Y										
16461	A_23_P49406	PRR14																	Y	Y			Y																							
13135	A_23_P431346	PRR15							Y	Y										Y		Y	Y																							
13170	A_23_P402241	PRR18				Y														Y			Y																							
12700	A_23_P76291	PRR4																Y																												
16027	A_23_P62040	PRR4	Y		Y	Y	Y	Y		Y	Y	Y	Y	Y				Y	Y		Y	Y					Y																			

19598	A_23_P80382	PRR5			Y	Y		Y	Y	Y										Y		Y	Y				Y	Y	Y	Y																
14353	A_23_P30464	PRR7																Y	Y	Y		Y		Y																						
21815	A_23_P254363	PRRG1			Y				Y	Y	Y	Y	Y	Y													Y																			
1626	A_23_P67355	PRRG2			Y	Y		Y		Y																	Y	Y	Y	Y						Y										
21980	A_23_P127663	PRRG4			Y				Y	Y																				Y						Y										
501	A_23_P214649	PRRT1			Y				Y	Y																	Y																			
22342	A_23_P66017	PRRT2	Y	Y	Y	Y	Y	Y	Y	Y	Y	Y	Y	Y				Y	Y			Y					Y	Y	Y	Y						Y										
324	A_23_P80626	PRRT3	Y	Y	Y	Y	Y	Y	Y	Y	Y	Y	Y	Y				Y		Y	Y	Y	Y				Y	Y	Y	Y						Y										
4237	A_23_P83298	PRRX2			Y													Y		Y		Y	Y																							
9796	A_23_P380754	PRSS1	Y	Y	Y										Y					Y							Y			Y						Y										
5920	A_23_P121637	PRSS12																		Y																										
15574	A_23_P340137	PRSS16				Y																						Y																		
10094	A_23_P310274	PRSS2													Y																															
11087	A_23_P335838	PRSS2	Y	Y	Y								Y							Y							Y			Y						Y										
5177	A_23_P129602	PRSS21																Y	Y	Y		Y		Y																						
8827	A_23_P66203	PRSS22			Y														Y								Y																			
16922	A_23_P150789	PRSS23																		Y							Y																			
5047	A_23_P106806	PRSS27			Y	Y		Y		Y								Y	Y	Y		Y		Y			Y	Y	Y	Y																
9026	A_23_P135257	PRSS3				Y																																								
12016	A_23_P89003	PRSS33							Y	Y								Y		Y							Y																			
15458	A_23_P102864	PRSS7	Y		Y													Y		Y			Y				Y			Y						Y										
6876	A_23_P251801	PRSSL1																Y																												
3164	A_23_P202004	PRTFDC1			Y	Y		Y	Y	Y	Y	Y	Y	Y													Y	Y	Y	Y						Y										
1556	A_23_P142345	PRTN3							Y	Y																	Y			Y						Y										
4769	A_23_P429959	PRX																												Y						Y										
21479	A_23_P11408	PRY2				Y																					Y																			
13713	A_23_P12675	PSAP			Y	Y		Y	Y	Y					Y			Y		Y	Y	Y	Y				Y																			
13569	A_23_P213020	PSAPL1			Y																																									
3250	A_23_P259692	PSAT1			Y	Y		Y	Y	Y	Y	Y	Y	Y													Y	Y	Y	Y						Y										
7883	A_23_P71379	PSCA	Y															Y	Y																											
22553	A_23_P30358	PSD2			Y	Y		Y		Y																																				
13228	A_23_P20392	PSD3			Y	Y		Y	Y	Y									Y	Y		Y					Y			Y						Y										
15542	A_23_P216167	PSD3	Y	Y	Y	Y		Y		Y								Y	Y	Y				Y				Y		Y						Y										
21668	A_23_P340729	PSEN1			Y																																									
19445	A_23_P103398	PSEN2			Y	Y		Y		Y								Y	Y	Y				Y			Y																			
11904	A_23_P153807	PSENEN			Y																																									
14585	A_23_P130948	PSG11			Y													Y		Y		Y					Y																			
11107	A_23_P56354	PSG3	Y	Y	Y	Y		Y		Y										Y							Y			Y						Y										
16076	A_23_P5103	PSG4				Y												Y		Y													Y													
3569	A_23_P119554	PSG8			Y																																									
21027	A_23_P39309	PSG9																									Y																			
2093	A_23_P390599	PSKH1			Y																		Y																							
3501	A_23_P100239	PSKH1			Y																						Y			Y						Y										
16498	A_23_P150286	PSMA1																	Y	Y																										
5112	A_23_P93954	PSMA2																		Y																										
17283	A_23_P345591	PSMA2			Y																																									
13893	A_23_P86216	PSMA5			Y	Y		Y	Y	Y																																				
15417	A_23_P25735	PSMA6	Y	Y	Y																																									
15593	A_23_P91464	PSMA7	Y						Y	Y								Y		Y																										
21436	A_23_P156526	PSMB1							Y	Y																																				
19568	A_23_P140805	PSMB10																Y																												
19312	A_23_P128836	PSMB11																									Y	Y	Y	Y						Y										
1293	A_23_P170058	PSMB2			Y	Y		Y		Y								Y		Y							Y	Y	Y	Y																
20090	A_23_P100576	PSMB3			Y	Y		Y		Y																																				
10775	A_23_P37191	PSMB5			Y														Y	Y							Y																			
2792	A_23_P15705	PSMB6			Y	Y		Y		Y																																				
8278	A_23_P250629	PSMB8			Y				Y	Y																		Y																		
5162	A_23_P111000	PSMB9	Y	Y	Y	Y	Y	Y	Y	Y	Y	Y	Y	Y													Y	Y	Y	Y																
5855	A_23_P123073	PSMC2			Y	Y	Y	Y	Y	Y	Y	Y						Y	Y	Y		Y		Y			Y	Y	Y	Y						Y										
10706	A_23_P304287	PSMC2	Y	Y																																										
21010	A_23_P104605	PSMC3			Y	Y	Y	Y		Y	Y	Y	Y	Y						Y	Y	Y	Y				Y																			
10441	A_23_P118582	PSMC3IP			Y	Y		Y		Y																	Y																			
1422	A_23_P119485	PSMC4			Y													Y	Y	Y	Y	Y	Y	Y	Y	Y	Y				Y															
7546	A_23_P164028	PSMC5							Y	Y										Y								Y		Y						Y										
19577	A_23_P209747	PSMD1																Y	Y	Y		Y		Y																						
14704	A_23_P207600	PSMD11			Y															Y	Y						Y																			
8213	A_23_P77876	PSMD12																		Y																										
6493	A_23_P75889	PSMD13			Y	Y	Y	Y		Y	Y	Y								Y							Y																			
15647	A_23_P165691	PSMD14	Y	Y																																										
20521	A_23_P144236	PSMD2			Y																																									
18690	A_23_P26785	PSMD3			Y				Y	Y								Y	Y	Y	Y	Y		Y																						
20474	A_23_P12168	PSMD4			Y	Y		Y		Y								Y				Y					Y	Y	Y	Y																
707	A_23_P43434	PSMD5							Y	Y										Y																										
2880	A_23_P109928	PSMD6																					Y																							
15618	A_23_P106741	PSMD7	Y	Y	Y				Y	Y									Y	Y	Y		Y																							
12025	A_23_P67459	PSMD8			Y																																									
11963	A_23_P203994	PSMD9			Y	Y		Y		Y										Y							Y	Y	Y	Y																

18765	A_23_P151610	PSME1																												Y						Y										
1815	A_23_P65427	PSME2			Y				Y	Y										Y																										
2845	A_23_P164141	PSME3																Y																												
8132	A_23_P79628	PSME4			Y																																									
13938	A_23_P79910	PSMF1			Y	Y	Y	Y	Y	Y	Y	Y	Y	Y				Y		Y		Y	Y				Y	Y	Y	Y							Y										
19974	A_23_P68717	PSMG1	Y	Y	Y	Y		Y	Y	Y								Y				Y					Y	Y	Y	Y							Y										
20316	A_23_P118909	PSMG2			Y				Y	Y								Y	Y	Y				Y			Y			Y							Y										
3604	A_23_P133900	PSORS1C1																	Y		Y																									
7457	A_23_P81922	PSORS1C2																					Y																							
21224	A_23_P76610	PSPC1			Y	Y	Y	Y	Y	Y	Y	Y	Y														Y																			
21159	A_23_P410507	PSPN			Y	Y	Y	Y		Y	Y	Y	Y	Y									Y				Y																			
15310	A_23_P12866	PSTK			Y	Y		Y		Y										Y																										
18915	A_23_P48997	PSTPIP1	Y		Y	Y	Y	Y		Y	Y	Y			Y			Y	Y	Y			Y	Y			Y	Y	Y	Y																
2901	A_23_P51926	PTAFR	Y	Y	Y	Y	Y	Y	Y	Y	Y	Y			Y			Y		Y			Y				Y																			
3794	A_23_P208981	PTBP1			Y	Y		Y		Y								Y	Y	Y		Y	Y	Y			Y																			
11205	A_23_P46396	PTBP2			Y	Y		Y	Y	Y																				Y						Y										
22556	A_23_P134650	PTCD1																	Y																											
498	A_23_P121932	PTCD2			Y	Y		Y	Y	Y	Y	Y	Y	Y													Y	Y	Y	Y							Y										
10135	A_23_P355311	PTCH2				Y			Y	Y																																				
21339	A_23_P303251	PTCHD1							Y	Y	Y	Y	Y	Y																Y						Y										
13151	A_23_P30755	PTCRA							Y	Y										Y			Y																							
8356	A_23_P168868	PTDSS1			Y	Y	Y	Y	Y	Y	Y	Y	Y	Y				Y	Y	Y	Y	Y	Y	Y	Y	Y	Y	Y	Y	Y	Y	Y				Y										
4269	A_23_P138772	PTDSS2																		Y			Y				Y																			
3804	A_23_P98085	PTEN			Y	Y	Y	Y	Y	Y	Y	Y								Y							Y	Y	Y	Y																
2182	A_23_P323774	PTER																		Y																										
11414	A_23_P202045	PTER																Y		Y																										
15648	A_23_P146554	PTGDS			Y														Y								Y																			
18837	A_23_P4808	PTGER1																Y	Y								Y			Y							Y										
22009	A_23_P151710	PTGER2																												Y						Y										
18315	A_23_P103328	PTGER3																	Y	Y																										
18421	A_23_P158857	PTGER3			Y				Y	Y									Y								Y																			
18009	A_23_P148047	PTGER4			Y														Y								Y																			
7679	A_23_P169446	PTGES			Y				Y	Y																	Y																			
1645	A_23_P43617	PTGES2			Y	Y		Y	Y	Y	Y	Y	Y	Y						Y							Y	Y	Y	Y																
3894	A_23_P11674	PTGFR			Y													Y	Y			Y	Y																							
6376	A_23_P114968	PTGFRN																		Y	Y	Y	Y										Y													
257	A_23_P340848	PTGIR			Y	Y	Y	Y	Y	Y	Y	Y	Y	Y	Y			Y	Y	Y		Y	Y	Y			Y	Y	Y	Y							Y										
22106	A_23_P91262	PTGIS				Y																																								
22426	A_23_P48713	PTGR2			Y	Y	Y	Y	Y	Y	Y	Y	Y	Y						Y	Y	Y	Y				Y			Y							Y										
20657	A_23_P24835	PTH							Y	Y																		Y		Y							Y										
22449	A_23_P167027	PTH1R																					Y																							
12883	A_23_P357374	PTH2																		Y			Y																							
7114	A_23_P28181	PTH2R			Y																Y																									
14782	A_23_P2271	PTHLH																Y																												
22008	A_23_P168836	PTK2B			Y				Y	Y										Y	Y	Y	Y				Y																			
16077	A_23_P56978	PTK6							Y	Y																				Y						Y										
2828	A_23_P122402	PTK7																Y		Y		Y																								
16727	A_23_P210283	PTMA																												Y						Y										
19098	A_23_P128333	PTMS																		Y			Y																							
11068	A_23_P303085	PTN			Y																						Y			Y						Y										
22173	A_23_P134213	PTN			Y																						Y			Y						Y										
17789	A_23_P325080	PTOV1			Y	Y		Y		Y								Y	Y	Y	Y	Y	Y	Y	Y	Y	Y			Y	Y	Y				Y										
6791	A_23_P200414	PTP4A2			Y	Y		Y	Y	Y	Y	Y	Y	Y						Y							Y	Y	Y	Y							Y										
9183	A_23_P112774	PTP4A3			Y	Y	Y	Y	Y	Y	Y	Y	Y	Y				Y	Y	Y		Y	Y	Y			Y	Y	Y	Y							Y										
316	A_23_P20876	PTPDC1				Y																																								
9751	A_23_P161352	PTPLA			Y	Y		Y	Y	Y	Y	Y	Y	Y																Y						Y										
21396	A_23_P1549	PTPMT1																															Y													
1437	A_23_P210569	PTPN1			Y													Y		Y	Y																									
5597	A_23_P204099	PTPN11																				Y					Y																			
16622	A_23_P99027	PTPN11			Y	Y		Y	Y	Y										Y							Y																			
3833	A_23_P8763	PTPN12			Y				Y	Y																	Y																			
12094	A_23_P18493	PTPN13			Y	Y	Y	Y	Y	Y	Y	Y	Y	Y					Y	Y	Y	Y					Y	Y	Y	Y							Y										
15701	A_23_P149111	PTPN14			Y				Y	Y	Y	Y	Y	Y																																
2234	A_23_P210015	PTPN18																Y	Y	Y		Y		Y																						
8525	A_23_P322101	PTPN18																Y	Y	Y		Y	Y	Y																						
10294	A_23_P77033	PTPN21			Y																																									
9531	A_23_P212329	PTPN23																Y	Y	Y		Y		Y																						
22206	A_23_P403898	PTPN3			Y	Y		Y		Y								Y		Y	Y	Y	Y				Y																			
5023	A_23_P162486	PTPN6			Y	Y	Y	Y	Y	Y	Y	Y							Y	Y	Y	Y	Y				Y	Y	Y	Y							Y										
13391	A_23_P201778	PTPN7	Y		Y	Y	Y	Y	Y	Y	Y	Y			Y				Y	Y	Y		Y				Y																			
5122	A_23_P28869	PTPRA			Y	Y		Y		Y										Y							Y	Y	Y	Y							Y										
7693	A_23_P33660	PTPRC	Y																																											
9638	A_23_P125451	PTPRC	Y	Y																																										
10225	A_23_P136837	PTPRC	Y		Y				Y	Y								Y									Y																			
19415	A_23_P148227	PTPRC	Y	Y																																										
6048	A_23_P98173	PTPRCAP			Y				Y	Y									Y				Y				Y																			
11718	A_23_P252132	PTPRD			Y														Y								Y																			

3646	A_23_P138495	PTPRE			Y																																								
19155	A_23_P200989	PTPRF																Y																											
19635	A_23_P41054	PTPRG			Y													Y	Y	Y		Y	Y	Y			Y	Y	Y	Y															
5408	A_23_P101642	PTPRH			Y																						Y																			
3694	A_23_P36275	PTPRJ			Y				Y	Y										Y										Y						Y										
10661	A_23_P254242	PTPRK			Y														Y	Y	Y	Y	Y																							
11407	A_23_P254924	PTPRK			Y	Y		Y		Y									Y	Y	Y	Y	Y				Y																			
12671	A_23_P255541	PTPRM																	Y																											
16239	A_23_P159255	PTPRM																	Y																											
14925	A_23_P90722	PTPRN			Y																						Y																			
20773	A_23_P113221	PTPRN2																			Y																									
16364	A_23_P47924	PTPRR																											Y						Y										
7384	A_23_P56163	PTPRS			Y													Y	Y	Y		Y		Y			Y																			
2643	A_23_P146970	PTPRT	Y	Y	Y	Y	Y	Y	Y	Y	Y	Y	Y	Y				Y	Y	Y	Y	Y	Y	Y	Y	Y	Y	Y	Y	Y	Y	Y	Y			Y										
6090	A_23_P135576	PTPRT	Y	Y	Y	Y	Y	Y	Y	Y	Y	Y	Y	Y				Y	Y	Y		Y	Y	Y			Y	Y	Y	Y			Y			Y										
12361	A_23_P168761	PTPRZ1													Y																															
10910	A_23_P141376	PTRF			Y													Y	Y	Y		Y	Y	Y			Y						Y													
4644	A_23_P15493	PTRH2				Y																						Y																		
9371	A_23_P127579	PTS							Y	Y													Y					Y		Y						Y										
3021	A_23_P60024	PTTG1	Y	Y	Y													Y	Y	Y		Y		Y			Y																			
6173	A_23_P7636	PTTG1	Y	Y														Y	Y	Y		Y		Y			Y																			
15753	A_23_P18579	PTTG2	Y	Y																Y		Y																								
22215	A_23_P121064	PTX3							Y	Y	Y	Y	Y	Y																Y						Y										
10906	A_23_P158366	PUF60			Y	Y	Y	Y	Y	Y	Y	Y						Y	Y	Y	Y	Y		Y			Y	Y	Y	Y						Y										
13561	A_23_P9756	PUF60			Y	Y	Y	Y	Y	Y	Y	Y						Y	Y	Y	Y	Y		Y			Y			Y						Y										
11856	A_23_P10815	PUM1			Y	Y		Y	Y	Y	Y	Y	Y	Y													Y																			
558	A_23_P5541	PUM2			Y	Y	Y	Y	Y	Y	Y	Y	Y	Y				Y	Y	Y	Y	Y	Y	Y	Y	Y	Y	Y	Y	Y	Y	Y				Y										
8093	A_23_P41755	PURA																		Y			Y																							
14817	A_23_P414856	PURB																		Y		Y	Y																							
12847	A_23_P111919	PURG																									Y																			
7428	A_23_P251776	PUS1																	Y	Y		Y	Y					Y																		
15215	A_23_P373687	PUS10																		Y		Y	Y																							
13347	A_23_P13073	PUS3																	Y	Y	Y	Y																								
10105	A_23_P36776	PUS7L																		Y																										
18143	A_23_P51884	PUSL1				Y			Y	Y																	Y	Y	Y	Y																
15832	A_23_P17844	PVALB			Y	Y	Y	Y	Y	Y	Y	Y	Y	Y					Y	Y							Y	Y	Y	Y						Y										
20913	A_23_P141894	PVR			Y	Y	Y	Y	Y	Y	Y	Y	Y	Y				Y	Y	Y	Y	Y	Y	Y	Y	Y	Y			Y	Y	Y	Y			Y										
10582	A_23_P168427	PVRIG	Y		Y													Y	Y																											
16999	A_23_P8424	PVRIG			Y		Y				Y								Y	Y			Y																							
20378	A_23_P76034	PVRL1			Y	Y	Y	Y		Y	Y	Y	Y	Y				Y	Y	Y		Y		Y			Y	Y	Y	Y						Y										
10349	A_23_P352867	PVRL2																	Y																											
11602	A_23_P208293	PVRL2			Y				Y	Y	Y	Y	Y	Y				Y					Y							Y						Y										
7498	A_23_P80763	PVRL3																	Y																											
14972	A_23_P105753	PWP1			Y	Y	Y	Y	Y	Y	Y	Y						Y	Y	Y			Y	Y			Y																			
6729	A_23_P102927	PWP2			Y	Y	Y	Y	Y	Y	Y	Y						Y	Y	Y		Y	Y	Y			Y			Y	Y	Y	Y	Y	Y	Y				Y						
1884	A_23_P135423	PXK																Y	Y	Y		Y	Y	Y				Y																		
8038	A_23_P91430	PXMP4							Y	Y																				Y						Y										
5686	A_23_P162573	PXN							Y	Y																																				
9897	A_23_P13969	PXN	Y	Y	Y	Y		Y		Y	Y	Y	Y	Y														Y		Y						Y										
14177	A_23_P26632	PYCARD			Y	Y		Y		Y								Y		Y							Y																			
7446	A_23_P130194	PYCR1			Y	Y	Y	Y		Y	Y	Y						Y	Y	Y		Y		Y			Y															Y				
13626	A_23_P149042	PYCR2				Y													Y	Y		Y																								
466	A_23_P82899	PYCRL			Y	Y	Y	Y		Y	Y	Y						Y	Y	Y		Y		Y			Y																			
11737	A_23_P383278	PYCRL			Y	Y	Y	Y	Y	Y	Y	Y						Y	Y	Y		Y	Y	Y			Y			Y						Y										
4494	A_23_P38030	PYDC1	Y		Y																																									
9487	A_23_P210840	PYGB			Y	Y		Y	Y	Y								Y	Y	Y	Y	Y		Y			Y																			
13933	A_23_P331670	PYGB			Y	Y		Y		Y								Y		Y	Y	Y					Y	Y	Y	Y																
21646	A_23_P48676	PYGL							Y	Y								Y	Y																											
18535	A_23_P405815	PYGM							Y	Y																																				
6035	A_23_P204187	PYROXD1																		Y																										
15980	A_23_P139682	PZP																					Y																							
10831	A_23_P135347	QARS			Y																																									
6556	A_23_P167212	QDPR			Y	Y	Y	Y	Y	Y	Y	Y	Y	Y						Y	Y		Y				Y	Y	Y	Y						Y						Y				
7522	A_23_P133049	QDPR			Y	Y	Y	Y	Y	Y	Y	Y	Y	Y						Y	Y		Y				Y	Y	Y	Y						Y						Y				
18124	A_23_P81759	QKI				Y			Y	Y																																				
11289	A_23_P16915	QPCT			Y	Y		Y		Y									Y								Y			Y						Y										
720	A_23_P130789	QPCTL			Y	Y		Y		Y								Y	Y	Y		Y	Y	Y			Y																			
15339	A_23_P218225	QPRT			Y				Y	Y								Y	Y	Y		Y		Y			Y																			
11311	A_23_P45108	QRICH1			Y																																									
104	A_23_P82181	QRSL1																Y																												
15615	A_23_P203283	QSER1																		Y			Y																							
2964	A_23_P12463	QSOX1	Y	Y	Y	Y	Y	Y	Y	Y	Y	Y						Y	Y	Y	Y		Y	Y	Y		Y	Y	Y	Y						Y										
6078	A_23_P89953	QTRT1																		Y																										
8895	A_23_P91930	QTRTD1			Y	Y		Y	Y	Y										Y		Y	Y				Y	Y	Y	Y																
7472	A_23_P380998	R3HDM1																		Y	Y																									
17557	A_23_P10374	R3HDM2			Y				Y	Y																																				
3443	A_23_P165879	RAB10			Y															Y	Y	Y	Y																							

4330	A_23_P77142	RAB11A			Y	Y	Y	Y	Y	Y	Y	Y	Y	Y													Y	Y	Y	Y						Y										
4478	A_23_P434454	RAB11B			Y	Y	Y	Y		Y	Y	Y						Y		Y		Y					Y	Y	Y	Y																
9545	A_23_P67748	RAB11B			Y	Y		Y		Y										Y																										
6670	A_23_P127135	RAB11FIP2			Y	Y	Y	Y	Y	Y	Y	Y						Y	Y	Y		Y		Y			Y	Y	Y	Y						Y										
3989	A_23_P38427	RAB11FIP4																	Y																											
9495	A_23_P27023	RAB11FIP4																												Y						Y										
11535	A_23_P46365	RAB13			Y																																									
12325	A_23_P216985	RAB14				Y												Y	Y	Y		Y		Y				Y																		
13279	A_23_P5778	RAB17																												Y						Y										
18164	A_23_P382148	RAB1A			Y																																									
9141	A_23_P64090	RAB1B																	Y	Y																										
19934	A_23_P128166	RAB21																		Y	Y																									
15093	A_23_P70794	RAB23							Y	Y																																				
18701	A_23_P115091	RAB25																	Y																											
8025	A_23_P54709	RAB26							Y	Y																																				
8766	A_23_P54367	RAB27A	Y	Y																								Y																		
738	A_23_P107611	RAB27B			Y				Y	Y	Y	Y	Y	Y						Y							Y			Y						Y										
2919	A_23_P113760	RAB28			Y																																									
11928	A_23_P125147	RAB28			Y																		Y																							
20568	A_23_P139359	RAB30	Y						Y	Y																				Y						Y										
6893	A_23_P42375	RAB32				Y					Y		Y	Y																																
10416	A_23_P147025	RAB33A																Y	Y																											
21203	A_23_P169569	RAB33B			Y	Y		Y		Y										Y			Y				Y	Y	Y	Y																
14912	A_23_P152876	RAB34																									Y																			
5878	A_23_P204484	RAB35	Y		Y	Y	Y	Y	Y	Y	Y	Y						Y	Y	Y	Y	Y	Y	Y	Y	Y	Y			Y	Y	Y	Y	Y	Y	Y										
2104	A_23_P161559	RAB38	Y		Y	Y		Y	Y	Y								Y	Y	Y				Y			Y																			
8786	A_23_P108200	RAB3A																										Y																		
2617	A_23_P62672	RAB3B			Y	Y	Y	Y	Y	Y	Y	Y							Y	Y		Y	Y				Y			Y						Y										
1581	A_23_P44794	RAB3C																Y																												
13724	A_23_P201664	RAB3GAP2			Y	Y	Y	Y	Y	Y	Y	Y	Y	Y					Y	Y	Y	Y	Y				Y	Y	Y	Y																
16604	A_23_P75802	RAB3IL1																		Y		Y																								
353	A_23_P128174	RAB3IP			Y	Y		Y	Y	Y	Y	Y	Y	Y				Y	Y	Y	Y	Y	Y	Y	Y	Y				Y	Y	Y				Y										
8305	A_23_P259912	RAB40A	Y	Y	Y	Y		Y		Y																	Y	Y	Y	Y																
10235	A_23_P396058	RAB40C																	Y	Y		Y																								
18778	A_23_P152289	RAB40C			Y	Y		Y		Y								Y	Y	Y		Y	Y	Y			Y																			
4681	A_23_P434919	RAB42	Y		Y	Y	Y	Y	Y	Y	Y	Y	Y	Y				Y	Y	Y	Y	Y	Y	Y	Y	Y	Y	Y	Y	Y	Y	Y				Y		Y				Y			Y	Y
5556	A_23_P77552	RAB43			Y	Y	Y	Y	Y	Y	Y	Y							Y	Y		Y	Y				Y	Y	Y	Y						Y						Y				
1965	A_23_P16119	RAB4B			Y				Y	Y			Y					Y	Y	Y			Y	Y						Y						Y										
5863	A_23_P212542	RAB5A			Y															Y																										
17932	A_23_P162238	RAB5B																			Y						Y																			
2841	A_23_P372411	RAB5C																Y	Y	Y	Y	Y	Y	Y	Y	Y	Y				Y															
19380	A_23_P107214	RAB5C																			Y	Y																								
18537	A_23_P211758	RAB7A	Y	Y	Y	Y		Y		Y																																				
16104	A_23_P61592	RAB7B			Y	Y		Y	Y	Y	Y	Y	Y	Y				Y	Y	Y		Y	Y	Y				Y		Y						Y										
19325	A_23_P252549	RAB7B							Y	Y								Y	Y	Y		Y		Y			Y																			
11063	A_23_P126939	RAB7L1			Y	Y		Y	Y	Y									Y	Y							Y																			
12964	A_23_P37535	RAB8B	Y		Y																																									
14137	A_23_P406448	RAB9B			Y																																									
6590	A_23_P38864	RABAC1																		Y																										
2390	A_23_P78158	RABEP1	Y	Y	Y	Y	Y	Y	Y	Y	Y	Y	Y	Y				Y	Y	Y		Y	Y	Y			Y	Y	Y	Y	Y	Y	Y	Y	Y	Y										
15278	A_23_P342759	RABEP1	Y	Y	Y	Y	Y	Y	Y	Y	Y	Y	Y	Y				Y	Y	Y		Y	Y	Y			Y	Y	Y	Y						Y										
22190	A_23_P61100	RABEP2			Y															Y		Y					Y																			
11390	A_23_P123814	RABEPK																	Y	Y		Y	Y																							
10054	A_23_P216958	RABGAP1																	Y																											
10572	A_23_P377264	RABGAP1L			Y	Y	Y	Y	Y	Y	Y	Y	Y					Y	Y	Y		Y		Y				Y		Y						Y										
656	A_23_P54218	RABGGTA																		Y		Y																								
613	A_23_P85371	RABGGTB				Y																																								
20691	A_23_P63503	RABIF	Y	Y		Y																					Y	Y	Y	Y																
11129	A_23_P158349	RABL3																					Y					Y		Y						Y										
20436	A_23_P369393	RABL3																		Y																										
3897	A_23_P6443	RABL4							Y	Y																																				
19419	A_23_P31584	RABL5			Y				Y	Y									Y	Y	Y	Y					Y	Y	Y	Y						Y										
865	A_23_P215406	RAC1				Y																																								
6378	A_23_P388071	RAC1			Y		Y				Y							Y		Y	Y	Y					Y			Y						Y										
44	A_23_P218774	RAC2			Y														Y				Y																							
22172	A_23_P125001	RAC3																		Y		Y	Y							Y						Y										
20019	A_23_P65040	RACGAP1P																					Y																							
8614	A_23_P144697	RAD1																					Y																							
8521	A_23_P159053	RAD17			Y	Y	Y	Y	Y	Y	Y	Y	Y	Y				Y	Y	Y	Y	Y	Y	Y	Y	Y	Y	Y	Y	Y	Y	Y				Y										
17457	A_23_P121222	RAD18																		Y			Y																							
13654	A_23_P20463	RAD21							Y	Y																																				
9151	A_23_P91317	RAD21L1																												Y						Y										
18901	A_23_P55889	RAD23A			Y	Y	Y	Y		Y	Y	Y						Y		Y		Y																								
3869	A_23_P255273	RAD23B			Y														Y																											
C9413.3	A_23_P250404	RAD50			Y	Y	Y	Y		Y	Y	Y								Y			Y				Y	Y	Y	Y																
10860	A_23_P88731	RAD51	Y	Y	Y	Y		Y	Y	Y								Y	Y	Y				Y			Y	Y	Y	Y						Y										
329	A_23_P48481	RAD51L1																												Y						Y										

19336	A_23_P500871	RAD51L3									Y		Y	Y					Y	Y		Y								Y						Y										
22014	A_23_P207260	RAD51L3							Y	Y	Y	Y	Y	Y						Y										Y						Y										
17190	A_23_P64990	RAD52							Y	Y												Y	Y																								
487	A_23_P94141	RAD54B			Y																								Y																				
19879	A_23_P74115	RAD54L																		Y									Y																				
22345	A_23_P18202	RAD54L2																															Y						Y										
20447	A_23_P12920	RAD9A			Y													Y		Y							Y			Y						Y										
21235	A_23_P40952	RAF1																			Y	Y																										
16017	A_23_P360744	RAG1							Y	Y										Y																											
13596	A_23_P76731	RAGE					Y		Y	Y	Y	Y	Y	Y						Y	Y	Y	Y				Y						Y												
6497	A_23_P77807	RAI1																Y	Y	Y		Y	Y	Y																						
11213	A_23_P417144	RAI1	Y	Y	Y	Y		Y		Y																	Y	Y	Y	Y															
6989	A_23_P254165	RAI2			Y	Y		Y	Y	Y							Y	Y			Y					Y	Y	Y	Y						Y										
7513	A_23_P215302	RALA																												Y																		
2605	A_23_P16733	RALB							Y	Y																																				
1585	A_23_P376599	RALBP1																															Y						Y										
19224	A_23_P409376	RALGDS			Y															Y	Y	Y																							
10724	A_23_P390148	RALGPS1	Y		Y	Y	Y	Y	Y	Y	Y	Y	Y	Y					Y	Y	Y	Y	Y				Y	Y	Y	Y	Y	Y	Y	Y	Y	Y				Y						
2704	A_23_P97517	RALGPS2			Y	Y		Y	Y	Y	Y	Y	Y	Y						Y		Y	Y				Y	Y	Y	Y															
6834	A_23_P328120	RALGPS2			Y				Y	Y	Y	Y	Y	Y							Y								Y		Y						Y										
20300	A_23_P109235	RALY			Y	Y	Y	Y	Y	Y	Y	Y						Y	Y	Y	Y	Y	Y	Y	Y	Y	Y			Y	Y	Y				Y				Y						
10187	A_23_P388220	RALYL																	Y																												
21862	A_23_P50946	RAMP1			Y																							Y																				
4102	A_23_P111737	RAMP3							Y	Y								Y			Y					Y																				
10385	A_23_P91590	RANBP1			Y													Y	Y	Y	Y	Y	Y	Y	Y	Y	Y				Y																
21067	A_23_P58819	RANBP17			Y	Y		Y	Y	Y										Y																											
12957	A_23_P131664	RANBP2			Y	Y		Y		Y										Y		Y						Y		Y						Y										
2913	A_23_P208973	RANBP3			Y													Y	Y	Y	Y	Y	Y	Y	Y	Y	Y			Y	Y	Y				Y		Y								
7348	A_23_P310331	RANBP3				Y												Y				Y																							
21583	A_23_P146379	RANBP6																												Y		Y						Y										
16493	A_23_P59268	RANBP9			Y	Y	Y	Y	Y	Y	Y	Y							Y		Y						Y		Y						Y										
3073	A_23_P333420	RANGAP1			Y	Y	Y	Y		Y	Y	Y						Y	Y	Y	Y	Y	Y	Y	Y	Y	Y	Y	Y	Y	Y	Y				Y		Y				Y			Y	Y
3516	A_23_P254498	RANGRF			Y		Y				Y										Y							Y																				
17925	A_23_P151384	RAP2A	Y	Y	Y	Y		Y	Y	Y	Y	Y	Y	Y				Y			Y						Y	Y	Y	Y						Y										
4177	A_23_P258270	RAP2B			Y	Y		Y	Y	Y	Y	Y	Y	Y				Y	Y	Y	Y	Y	Y	Y	Y	Y	Y			Y	Y	Y	Y			Y				Y						
9331	A_23_P216412	RAPGEF1																					Y																						
16271	A_23_P391764	RAPGEF1																			Y		Y																							
8758	A_23_P133095	RAPGEF2																			Y																									
20046	A_23_P17192	RAPGEF4			Y	Y	Y	Y	Y	Y	Y	Y	Y	Y					Y	Y							Y			Y						Y										
10621	A_23_P215505	RAPGEF5																					Y																						
21176	A_23_P144994	RAPGEF6			Y	Y		Y	Y	Y	Y	Y	Y	Y					Y	Y		Y				Y	Y	Y	Y						Y										
3715	A_23_P120291	RAPH1																Y	Y	Y		Y		Y																					
8064	A_23_P409966	RAPH1			Y	Y		Y		Y																																				
15986	A_23_P86801	RAPSN							Y	Y																																				
22241	A_23_P18078	RARRES1	Y	Y	Y	Y		Y	Y	Y								Y	Y	Y				Y			Y	Y	Y	Y						Y										
17745	A_23_P134237	RARRES2			Y				Y	Y	Y	Y	Y	Y	Y			Y		Y	Y		Y																						
13636	A_23_P1962	RARRES3							Y	Y								Y		Y										Y						Y										
7709	A_23_P69988	RARS			Y	Y		Y		Y										Y							Y																				
20580	A_23_P18939	RASA1			Y	Y	Y	Y	Y	Y	Y	Y						Y	Y	Y	Y	Y	Y	Y	Y	Y	Y	Y	Y	Y	Y	Y	Y			Y										
16547	A_23_P91964	RASA2																	Y																												
3800	A_23_P158394	RASA3			Y	Y		Y		Y									Y	Y			Y				Y	Y	Y	Y						Y										
4569	A_23_P385673	RASA3			Y	Y		Y	Y	Y								Y	Y	Y	Y	Y	Y	Y	Y	Y					Y																
11122	A_23_P168449	RASA4					Y				Y		Y					Y		Y	Y	Y	Y				Y																				
4293	A_23_P139600	RASAL1				Y			Y	Y										Y																									
2807	A_23_P86124	RASAL2			Y						Y		Y	Y																																	
18165	A_23_P79069	RASAL3													Y			Y																													
2056	A_23_P143673	RASD2																									Y																				
15057	A_23_P331186	RASGEF1B			Y																																								
20393	A_23_P58263	RASGEF1B				Y			Y	Y	Y	Y	Y	Y																Y						Y										
5118	A_23_P156300	RASGEF1C				Y												Y	Y	Y		Y	Y	Y			Y	Y	Y	Y						Y										
9621	A_23_P3379	RASGRF1																												Y																		
6879	A_23_P124642	RASGRP1																Y		Y			Y																						
18263	A_23_P113258	RASGRP1							Y	Y																																				
534	A_23_P64058	RASGRP2			Y								Y																																
14726	A_23_P209544	RASGRP3	Y	Y	Y	Y		Y	Y	Y																	Y																				
22002	A_23_P165007	RASGRP4							Y	Y																																				
6457	A_23_P119353	RASIP1																Y	Y				Y																						
17595	A_23_P38287	RASL10B			Y	Y		Y		Y																Y																				
713	A_23_P14124	RASL11A																	Y																												
8809	A_23_P69738	RASL11B			Y	Y		Y	Y	Y																Y	Y	Y	Y						Y										
3886	A_23_P48868	RASL12							Y	Y																																				
16736	A_23_P41066	RASSF1			Y	Y		Y		Y										Y		Y	Y				Y	Y	Y	Y															
22367	A_23_P166087	RASSF2	Y																																										
18605	A_23_P75260	RASSF4			Y		Y				Y												Y				Y																				
11012	A_23_P201497	RASSF5																									Y																				
15423	A_23_P420402	RASSF6																			Y											Y		Y	Y	Y														
10992	A_23_P52589	RASSF7																															Y						Y										

21764	A_23_P116720	RASSF8																					Y																							
16686	A_23_P56150	RAX2				Y			Y	Y																		Y		Y						Y										
16192	A_23_P123242	RBAK				Y			Y	Y									Y	Y							Y	Y	Y	Y						Y										
21322	A_23_P45902	RBBP4				Y																																								
461	A_23_P54781	RBBP6	Y	Y	Y	Y		Y	Y	Y																	Y	Y	Y	Y						Y										
9935	A_23_P363206	RBBP6			Y	Y		Y	Y	Y	Y	Y	Y	Y					Y	Y		Y																								
5281	A_23_P114221	RBBP7			Y	Y		Y	Y	Y	Y	Y	Y	Y						Y							Y			Y						Y										
19447	A_23_P252371	RBBP8	Y		Y	Y		Y		Y					Y			Y	Y	Y			Y	Y			Y	Y	Y	Y																
3732	A_23_P359504	RBBP9				Y																						Y																		
9107	A_23_P57181	RBBP9																												Y						Y										
22084	A_23_P257532	RBBP9				Y			Y	Y										Y	Y							Y		Y						Y										
9619	A_23_P166054	RBCK1			Y													Y	Y	Y	Y	Y	Y	Y	Y	Y	Y				Y															
2095	A_23_P9523	RBKS			Y		Y		Y	Y	Y	Y	Y	Y				Y	Y	Y	Y	Y	Y	Y	Y	Y	Y			Y	Y	Y	Y			Y										
7001	A_23_P125813	RBM10																Y	Y	Y			Y	Y																						
1673	A_23_P342000	RBM11			Y	Y	Y	Y	Y	Y	Y	Y	Y	Y						Y							Y	Y	Y	Y																
19313	A_23_P502832	RBM12			Y	Y	Y	Y	Y	Y	Y	Y								Y							Y	Y	Y	Y						Y										
13248	A_23_P150255	RBM14																	Y																											
20522	A_23_P161636	RBM14			Y	Y		Y		Y																		Y																		
8787	A_23_P34879	RBM15																		Y		Y																								
17799	A_23_P121405	RBM15B																Y	Y	Y		Y	Y	Y			Y																			
16748	A_23_P111303	RBM16			Y	Y	Y	Y	Y	Y	Y	Y	Y	Y					Y	Y	Y	Y	Y				Y	Y	Y	Y																
3837	A_23_P423315	RBM17	Y	Y					Y	Y	Y	Y	Y	Y				Y	Y	Y		Y		Y						Y						Y										
11956	A_23_P35645	RBM17			Y	Y	Y	Y	Y	Y	Y	Y						Y	Y	Y		Y		Y			Y			Y						Y										
21650	A_23_P20777	RBM18																										Y																		
11168	A_23_P204119	RBM19	Y	Y	Y	Y		Y	Y	Y																	Y	Y	Y	Y																
22414	A_23_P92895	RBM22			Y	Y		Y	Y	Y												Y					Y																			
14775	A_23_P88252	RBM23																		Y		Y																								
14857	A_23_P167812	RBM24			Y													Y	Y	Y			Y	Y			Y			Y						Y										
16447	A_23_P99762	RBM25				Y																						Y																		
17843	A_23_P218090	RBM26			Y	Y	Y	Y	Y	Y	Y	Y						Y	Y	Y		Y	Y	Y			Y	Y	Y	Y						Y						Y				
13976	A_23_P168629	RBM28																									Y																			
4114	A_23_P148308	RBM3			Y																																									
20929	A_23_P417111	RBM33																Y																												
9560	A_23_P259127	RBM35A																Y																												
4063	A_23_P100220	RBM35B																												Y						Y										
9576	A_23_P17425	RBM38			Y	Y		Y	Y	Y	Y	Y	Y	Y				Y	Y	Y		Y		Y			Y	Y	Y	Y						Y										
17343	A_23_P109254	RBM39			Y	Y	Y	Y	Y	Y	Y	Y															Y	Y	Y	Y																
16136	A_23_P217243	RBM41				Y												Y	Y	Y		Y	Y	Y																						
5514	A_23_P90442	RBM42			Y	Y	Y	Y	Y	Y	Y	Y						Y		Y		Y					Y																			
17683	A_23_P142994	RBM45			Y	Y	Y	Y		Y	Y	Y	Y	Y				Y	Y	Y		Y	Y	Y			Y																			
10117	A_23_P404580	RBM46																Y									Y			Y						Y										
3162	A_23_P132910	RBM47							Y	Y	Y	Y	Y	Y						Y			Y				Y			Y						Y										
14712	A_23_P1638	RBM4B																		Y		Y	Y																							
13136	A_23_P18276	RBM5			Y	Y		Y	Y	Y	Y	Y	Y	Y						Y		Y					Y	Y	Y	Y						Y						Y				
2718	A_23_P57941	RBM6			Y	Y		Y	Y	Y																		Y		Y						Y										
3512	A_23_P36496	RBMS1							Y	Y																				Y						Y										
15260	A_23_P10995	RBMS3			Y				Y	Y										Y			Y				Y			Y						Y										
1717	A_23_P96420	RBMX			Y	Y		Y		Y																																				
403	A_23_P424689	RBMY2FP																Y																												
8430	A_23_P75283	RBP4			Y				Y	Y																				Y						Y										
18553	A_23_P113244	RBPJ			Y	Y		Y	Y	Y								Y	Y	Y		Y		Y																						
20884	A_23_P29994	RBPJ			Y	Y		Y	Y	Y									Y	Y		Y						Y																		
15253	A_23_P346884	RBPJL																		Y																										
11712	A_23_P71316	RBPMS																Y	Y	Y	Y	Y	Y	Y	Y	Y	Y				Y															
11142	A_23_P211550	RBX1			Y	Y		Y		Y																				Y						Y										
18497	A_23_P166246	RCAN1																												Y						Y										
7574	A_23_P214258	RCAN2																										Y																		
3845	A_23_P391275	RCAN3																									Y																			
3228	A_23_P117163	RCBTB1	Y	Y	Y	Y	Y	Y	Y	Y	Y	Y	Y	Y				Y	Y	Y	Y	Y	Y	Y	Y	Y	Y	Y	Y	Y	Y	Y	Y	Y	Y											
1467	A_23_P14105	RCBTB2																Y	Y											Y						Y										
10562	A_23_P74953	RCC2			Y															Y							Y																			
18945	A_23_P106433	RCCD1																Y	Y	Y		Y		Y																						
528	A_23_P1641	RCE1			Y	Y	Y	Y		Y	Y	Y						Y	Y	Y		Y	Y	Y			Y			Y						Y										
16515	A_23_P35838	RCE1			Y	Y		Y		Y										Y		Y					Y																			
8077	A_23_P203299	RCN1																	Y																											
13836	A_23_P26207	RCN2			Y	Y		Y	Y	Y								Y				Y								Y						Y										
14789	A_23_P67339	RCN3	Y		Y	Y	Y	Y	Y	Y	Y	Y	Y		Y			Y	Y	Y	Y	Y	Y	Y	Y	Y	Y				Y		Y													
9370	A_23_P205247	RCOR1			Y																																									
3509	A_23_P24397	RCOR2																												Y						Y										
17196	A_23_P136317	RCOR3			Y		Y				Y								Y	Y		Y					Y																			
11629	A_23_P23279	RCSD1	Y	Y	Y										Y			Y	Y	Y				Y																						
13364	A_23_P122545	RDBP			Y	Y		Y		Y								Y	Y	Y				Y			Y	Y	Y	Y																
20982	A_23_P25683	RDH11			Y	Y		Y	Y	Y	Y	Y	Y	Y						Y	Y	Y	Y																			Y				
10731	A_23_P39185	RDH13																		Y	Y	Y																								
919	A_23_P90909	RDH14			Y	Y		Y		Y										Y		Y																								
1863	A_23_P203870	RDH5																					Y																							
4009	A_23_P50291	RDH8			Y	Y	Y	Y		Y	Y	Y															Y	Y	Y	Y						Y										

3788	A_23_P322550	REC8			Y																	Y					Y																						
6047	A_23_P83028	RECK																									Y	Y	Y	Y																		
17816	A_23_P71558	RECQL4			Y	Y		Y		Y								Y	Y	Y		Y	Y	Y			Y																						
15958	A_23_P96285	REEP1			Y	Y		Y	Y	Y	Y	Y	Y	Y						Y	Y	Y	Y							Y						Y									
10164	A_23_P96209	REEP4			Y	Y		Y		Y										Y	Y	Y	Y				Y																		
7342	A_23_P350045	REEP5							Y	Y	Y	Y	Y	Y					Y	Y										Y						Y									
8037	A_23_P90510	REEP6	Y	Y					Y	Y	Y	Y	Y	Y				Y		Y		Y						Y		Y						Y									
5881	A_23_P90743	REG1A							Y	Y																			Y						Y									
3003	A_23_P389500	REG1B			Y																						Y																		
2297	A_23_P108546	REG1P																													Y						Y									
9591	A_23_P114975	REG4			Y																						Y	Y	Y	Y						Y									
17273	A_23_P400310	REG4																										Y																	
19532	A_23_P56938	REL																										Y																	
1842	A_23_P104689	RELA			Y		Y		Y	Y	Y	Y								Y	Y	Y	Y				Y																		
18417	A_23_P55706	RELB							Y	Y										Y								Y		Y						Y									
10197	A_23_P382744	RELL2			Y															Y	Y	Y																							
17091	A_23_P69853	RELL2			Y	Y	Y	Y		Y	Y	Y						Y	Y	Y	Y	Y	Y	Y	Y	Y	Y				Y		Y												
2402	A_23_P47735	RELT			Y												Y	Y	Y		Y	Y	Y			Y																		
7909	A_23_P34637	REN																										Y																	
19179	A_23_P137155	RENBP			Y	Y		Y		Y										Y							Y	Y	Y	Y						Y									
12013	A_23_P36479	REP15																	Y																										
12368	A_23_P70748	REPS1				Y																																							
21658	A_23_P251387	REPS2			Y	Y	Y	Y	Y	Y	Y	Y	Y	Y						Y	Y	Y	Y				Y			Y						Y									
20595	A_23_P85392	RER1			Y	Y		Y		Y																	Y	Y	Y	Y																
20434	A_23_P204296	RERG	Y	Y	Y	Y		Y	Y	Y																	Y	Y	Y	Y																
9455	A_23_P64919	RERGL			Y																																								
19784	A_23_P202245	RET			Y	Y		Y	Y	Y	Y	Y	Y	Y													Y																		
13022	A_23_P119222	RETN	Y	Y		Y					Y		Y	Y				Y	Y	Y				Y				Y		Y			Y			Y									
19680	A_23_P92196	RETNLB			Y																																								
10867	A_23_P209946	RETSAT																										Y		Y						Y									
1751	A_23_P142935	REV1	Y		Y	Y		Y	Y	Y									Y	Y								Y		Y						Y									
17296	A_23_P214139	REV3L			Y	Y		Y		Y																																			
3941	A_23_P150365	REXO2			Y	Y		Y		Y																	Y																		
7908	A_23_P18465	RFC1			Y	Y	Y	Y	Y	Y	Y	Y								Y			Y																						
14990	A_23_P93823	RFC2			Y	Y		Y		Y								Y	Y	Y		Y		Y			Y	Y	Y	Y						Y									
18703	A_23_P14193	RFC3			Y				Y	Y																	Y																		
1474	A_23_P95302	RFC5			Y	Y		Y	Y	Y																	Y																		
21816	A_23_P339709	RFFL	Y	Y	Y	Y	Y	Y		Y	Y	Y	Y	Y				Y	Y	Y	Y	Y	Y	Y	Y	Y	Y	Y	Y	Y	Y	Y				Y									
21352	A_23_P216708	RFK																		Y																									
21101	A_23_P84629	RFNG			Y												Y	Y	Y	Y	Y	Y	Y	Y	Y	Y				Y		Y												
6194	A_23_P16370	RFPL4A																			Y																								
22416	A_23_P155441	RFT1			Y	Y		Y		Y								Y	Y	Y	Y	Y		Y			Y	Y	Y	Y																
8523	A_23_P33599	RFWD2																	Y	Y	Y	Y																							
17620	A_23_P396194	RFWD2																		Y	Y																								
22080	A_23_P92292	RFWD2																		Y	Y	Y																							
2325	A_23_P142039	RFX1																		Y			Y				Y																		
13388	A_23_P502350	RFX2			Y	Y		Y	Y	Y																	Y																		
15312	A_23_P50692	RFX2			Y	Y		Y		Y									Y			Y	Y				Y	Y	Y	Y																
20190	A_23_P414905	RFX3							Y	Y									Y	Y																									
21532	A_23_P9289	RFX3							Y	Y																			Y						Y									
55	A_23_P26107	RFX7																													Y						Y									
5029	A_23_P165180	RFXANK																		Y																									
12423	A_23_P315789	RFXAP			Y	Y		Y	Y	Y								Y		Y		Y	Y							Y						Y									
C9184.6	A_23_P166716	RG9MTD1				Y																																							
8380	A_23_P398637	RG9MTD2			Y	Y	Y	Y	Y	Y	Y	Y	Y	Y					Y	Y		Y	Y				Y	Y	Y	Y						Y									
2016	A_23_P400458	RG9MTD3	Y	Y	Y												Y		Y	Y	Y	Y				Y																		
10076	A_23_P115417	RGL1			Y																		Y																						
13253	A_23_P93383	RGL2																Y	Y																											
18526	A_23_P107903	RGL3	Y	Y	Y																						Y																		
15038	A_23_P306941	RGL4	Y	Y	Y				Y	Y								Y									Y																		
7109	A_23_P372305	RGMA			Y				Y	Y																	Y																		
13721	A_23_P58801	RGMB				Y																																							
3770	A_23_P259156	RGN							Y	Y					Y			Y		Y										Y						Y									
6112	A_23_P81298	RGNEF			Y	Y		Y		Y																	Y																		
327	A_23_P135013	RGP1			Y	Y		Y		Y																	Y																		
19339	A_23_P218637	RGPD5			Y	Y	Y	Y	Y	Y	Y	Y	Y	Y				Y	Y	Y	Y	Y	Y	Y	Y	Y	Y	Y	Y	Y	Y	Y	Y			Y									
10892	A_23_P138558	RGR																										Y																	
14308	A_23_P97141	RGS1			Y																																								
2755	A_23_P352435	RGS12			Y															Y		Y	Y																						
10293	A_23_P69510	RGS12																		Y			Y				Y																		
3171	A_23_P500093	RGS13							Y	Y					Y																														
12083	A_23_P103554	RGS13							Y	Y																																			
18196	A_23_P124931	RGS14			Y	Y	Y	Y	Y	Y	Y	Y	Y	Y						Y							Y	Y	Y	Y						Y									
18740	A_23_P217845	RGS16																										Y																	
12547	A_23_P302550	RGS18	Y	Y																																									
18236	A_23_P257014	RGS19			Y	Y	Y	Y	Y	Y	Y	Y	Y	Y					Y	Y	Y	Y	Y				Y	Y	Y	Y						Y									
18351	A_23_P114947	RGS2																													Y						Y									

8877	A_23_P73097	RGS20																Y									Y																			
14659	A_23_P134675	RGS22	Y	Y	Y	Y		Y		Y	Y	Y	Y	Y				Y		Y							Y				Y		Y	Y	Y									Y		
20328	A_23_P219197	RGS3			Y				Y	Y	Y	Y	Y	Y				Y		Y	Y	Y	Y							Y						Y										
19846	A_23_P46045	RGS5							Y	Y										Y																										
13031	A_23_P387548	RGS6			Y																						Y			Y						Y										
17636	A_23_P66877	RGS9																				Y																								
17368	A_23_P256579	RHAG	Y	Y	Y	Y	Y	Y	Y	Y	Y	Y	Y	Y						Y							Y	Y	Y	Y						Y										
2369	A_23_P147514	RHBDD1			Y				Y	Y								Y		Y		Y	Y				Y																			
816	A_23_P8561	RHBDD2			Y				Y	Y																	Y																			
16296	A_23_P155009	RHBDD3			Y	Y		Y		Y								Y	Y	Y		Y	Y	Y			Y			Y						Y										
18954	A_23_P163678	RHBDF1			Y																						Y																			
905	A_23_P118345	RHBDF2			Y	Y	Y	Y		Y	Y	Y							Y	Y		Y	Y				Y																			
6143	A_23_P329870	RHBDF2	Y	Y	Y	Y	Y	Y	Y	Y	Y	Y	Y	Y					Y	Y							Y	Y	Y	Y						Y										
21172	A_23_P26468	RHBDL1			Y	Y		Y	Y	Y										Y		Y	Y				Y																			
9104	A_23_P63432	RHBDL2			Y						Y		Y	Y													Y			Y						Y										
14645	A_23_P308731	RHBDL3			Y	Y		Y	Y	Y	Y	Y	Y	Y													Y	Y	Y	Y						Y										
6713	A_23_P51690	RHBG			Y	Y		Y		Y																	Y																			
17304	A_23_P151975	RHCG																		Y										Y						Y										
17586	A_23_P86270	RHD																									Y	Y	Y	Y																
14025	A_23_P57950	RHO				Y																						Y																		
16770	A_23_P51136	RHOB																Y																												
1099	A_23_P35634	RHOBTB1																												Y						Y										
5447	A_23_P20423	RHOBTB2			Y																						Y			Y						Y										
8671	A_23_P92710	RHOBTB3			Y																						Y																			
1857	A_23_P406422	RHOC			Y				Y	Y								Y		Y	Y	Y	Y																							
14823	A_23_P12514	RHOC																				Y																								
20938	A_23_P1523	RHOD				Y																Y																								
6573	A_23_P203983	RHOF			Y	Y	Y	Y	Y	Y	Y	Y	Y						Y	Y		Y	Y				Y																			
11743	A_23_P150718	RHOG	Y		Y	Y	Y	Y	Y	Y	Y	Y	Y	Y				Y	Y	Y	Y	Y	Y	Y	Y	Y	Y	Y	Y	Y	Y	Y	Y	Y	Y	Y										
C11622.6	A_23_P58132	RHOH							Y	Y																																				
16765	A_23_P89492	RHOT1																		Y							Y																			
83	A_23_P15067	RHOT2																Y	Y	Y	Y	Y	Y	Y	Y	Y					Y															
928	A_23_P424561	RHOV			Y	Y		Y	Y	Y								Y	Y	Y		Y	Y	Y				Y																		
19943	A_23_P117912	RHOV			Y	Y		Y	Y	Y								Y	Y	Y		Y	Y	Y																						
5574	A_23_P352494	RHOXF2			Y													Y																												
17023	A_23_P73677	RHOXF2B			Y	Y	Y	Y		Y	Y	Y						Y									Y	Y	Y	Y																
7401	A_23_P315386	RHPN1																		Y																										
12889	A_23_P87438	RHPN1							Y	Y																				Y						Y										
120	A_23_P119470	RHPN2																												Y						Y										
20925	A_23_P73667	RIBC1	Y	Y	Y	Y		Y	Y	Y																	Y	Y	Y	Y						Y										
17739	A_23_P64510	RIC3																	Y																											
11067	A_23_P64489	RIC8A																									Y			Y						Y										
21636	A_23_P64936	RIC8B																Y																												
9053	A_23_P161686	RICS			Y	Y	Y	Y	Y	Y	Y	Y	Y	Y						Y	Y	Y	Y				Y	Y	Y	Y	Y	Y	Y	Y	Y	Y										
1529	A_23_P367646	RICTOR							Y	Y																				Y						Y										
2324	A_23_P26815	RILP			Y	Y	Y	Y	Y	Y	Y	Y	Y	Y						Y							Y	Y	Y	Y						Y										
18816	A_23_P169934	RILPL1							Y	Y											Y																									
1709	A_23_P147786	RIMS2																									Y	Y	Y	Y																
14617	A_23_P64098	RIN1																		Y	Y		Y																							
2838	A_23_P91379	RIN2			Y																						Y																			
9799	A_23_P151820	RIN3			Y	Y		Y	Y	Y								Y	Y	Y	Y	Y	Y	Y	Y	Y	Y			Y	Y	Y				Y		Y								
1997	A_23_P93372	RING1																	Y																											
C7359.5	A_23_P133284	RIOK2			Y	Y	Y	Y		Y	Y	Y	Y	Y						Y	Y	Y					Y	Y	Y	Y																
18450	A_23_P55578	RIOK3																		Y			Y																							
2013	A_23_P370005	RIPK1							Y	Y																		Y		Y						Y										
1523	A_23_P252106	RIPK2			Y																						Y																			
16681	A_23_P14559	RIPK3			Y	Y		Y		Y								Y										Y																		
9505	A_23_P211260	RIPK4																		Y																										
16888	A_23_P70359	RIPPLY2							Y	Y																																				
2537	A_23_P316601	RIT1																												Y						Y										
16286	A_23_P10045	RIT2																				Y																								
8285	A_23_P8977	RLBP1L1																									Y																			
1963	A_23_P363123	RLBP1L2																					Y																							
16401	A_23_P126037	RLF			Y	Y		Y	Y	Y								Y	Y	Y		Y	Y	Y			Y																			
11074	A_23_P60210	RLN1			Y	Y		Y	Y	Y								Y	Y	Y		Y	Y	Y			Y	Y	Y	Y																
19701	A_23_P216454	RLN2			Y	Y		Y	Y	Y	Y	Y	Y	Y				Y	Y	Y		Y	Y	Y			Y	Y	Y	Y						Y						Y				
3374	A_23_P39008	RLN3			Y																						Y																			
20577	A_23_P112341	RMI1																		Y			Y																							
9974	A_23_P70708	RMND1			Y	Y		Y	Y	Y	Y	Y	Y	Y						Y			Y				Y	Y	Y	Y						Y										
2983	A_23_P257372	RMND5B			Y	Y		Y	Y	Y	Y	Y	Y	Y													Y	Y	Y	Y						Y										
19560	A_23_P401577	RMND5B																									Y																			
4468	A_23_P48596	RNASE1			Y	Y		Y	Y	Y					Y			Y	Y	Y		Y		Y																						
20346	A_23_P25807	RNASE13			Y	Y		Y		Y																																				
5717	A_23_P151638	RNASE2				Y																																								
7215	A_23_P205531	RNASE4	Y	Y	Y	Y		Y	Y	Y								Y	Y	Y		Y	Y	Y			Y	Y	Y	Y						Y										
22016	A_23_P3017	RNASE6	Y		Y																							Y																		
17010	A_23_P106080	RNASE7			Y																									Y						Y										

22351	A_23_P376124	RNASE8																Y										Y		Y						Y										
1121	A_23_P76872	RNASE9			Y				Y	Y																	Y			Y						Y										
8865	A_23_P27163	RNASEH1			Y	Y		Y		Y																				Y						Y										
17514	A_23_P164826	RNASEH2A																Y	Y	Y		Y		Y																						
21683	A_23_P140088	RNASEH2B																	Y	Y			Y																							
16897	A_23_P390172	RNASEL			Y	Y	Y	Y	Y	Y	Y	Y	Y	Y				Y	Y	Y		Y		Y			Y	Y	Y	Y						Y										
11427	A_23_P133600	RNASEN			Y	Y	Y	Y	Y	Y	Y	Y	Y	Y				Y	Y	Y		Y	Y	Y			Y	Y	Y	Y																
2340	A_23_P30707	RNASET2			Y													Y									Y																			
12069	A_23_P19302	RNASET2			Y	Y	Y	Y	Y	Y	Y	Y						Y	Y	Y		Y		Y			Y																			
12182	A_23_P53370	RND1							Y	Y																				Y						Y										
6604	A_23_P434807	RND2			Y	Y	Y	Y		Y	Y	Y															Y	Y	Y	Y						Y										
12798	A_23_P56709	RNF103			Y		Y		Y	Y	Y	Y	Y	Y						Y		Y	Y				Y			Y						Y						Y				
8559	A_23_P137434	RNF11																		Y																										
15544	A_23_P77160	RNF111			Y	Y	Y	Y		Y	Y	Y								Y			Y				Y	Y	Y	Y																
3011	A_23_P120458	RNF114			Y	Y		Y	Y	Y										Y							Y	Y	Y	Y						Y										
14658	A_23_P161064	RNF115																									Y																			
19156	A_23_P250054	RNF123			Y	Y		Y	Y	Y								Y	Y	Y	Y	Y	Y	Y	Y	Y	Y				Y		Y	Y	Y											
11636	A_23_P119709	RNF126			Y													Y	Y	Y		Y	Y	Y																						
11874	A_23_P41734	RNF130	Y	Y																										Y						Y										
5135	A_23_P42963	RNF133			Y														Y								Y																			
20673	A_23_P252281	RNF135			Y																						Y																			
21974	A_23_P255317	RNF14																		Y																										
2935	A_23_P259608	RNF144B							Y	Y																		Y																		
18202	A_23_P120153	RNF149				Y																																								
9723	A_23_P314131	RNF151							Y	Y																	Y			Y						Y										
3920	A_23_P89730	RNF165			Y																									Y						Y										
13661	A_23_P416244	RNF166			Y	Y	Y	Y	Y	Y	Y	Y	Y	Y				Y	Y	Y	Y	Y	Y	Y	Y	Y	Y			Y	Y	Y				Y										
2767	A_23_P141536	RNF167				Y																																								
12530	A_23_P99373	RNF17			Y															Y								Y																		
2650	A_23_P146209	RNF170				Y																																								
7459	A_23_P340263	RNF175							Y	Y			Y					Y		Y										Y						Y										
21369	A_23_P377996	RNF180																									Y			Y						Y										
10618	A_23_P108708	RNF181			Y															Y																										
20861	A_23_P83339	RNF183			Y	Y		Y		Y										Y							Y																			
13674	A_23_P103062	RNF185			Y													Y	Y	Y	Y	Y		Y			Y																			
5424	A_23_P126248	RNF186				Y													Y	Y		Y																								
11651	A_23_P321384	RNF19B			Y	Y	Y	Y		Y	Y	Y						Y	Y	Y	Y			Y			Y																			
3235	A_23_P216850	RNF20			Y	Y		Y	Y	Y										Y		Y	Y				Y																			
15849	A_23_P159217	RNF208			Y	Y	Y	Y	Y	Y	Y	Y							Y	Y			Y				Y																			
21372	A_23_P42724	RNF216																Y																												
14221	A_23_P353005	RNF217				Y																						Y																		
17552	A_23_P25638	RNF219																												Y						Y										
3584	A_23_P424401	RNF220			Y	Y	Y	Y		Y	Y	Y						Y	Y	Y		Y	Y	Y			Y			Y						Y										
7289	A_23_P201001	RNF220																				Y																								
6056	A_23_P39655	RNF25																		Y																										
752	A_23_P64630	RNF26			Y	Y	Y	Y	Y	Y	Y	Y	Y	Y				Y	Y	Y		Y	Y	Y			Y			Y						Y										
9245	A_23_P99632	RNF31																Y	Y	Y			Y	Y									Y	Y												
15632	A_23_P19816	RNF32																			Y		Y																							
20782	A_23_P128396	RNF34			Y	Y		Y		Y								Y		Y	Y	Y					Y																			
18050	A_23_P167311	RNF4			Y															Y	Y	Y																								
18505	A_23_P26616	RNF40																		Y			Y																							
7053	A_23_P98884	RNF41																									Y																			
14277	A_23_P3934	RNF43							Y	Y									Y											Y						Y										
8270	A_23_P213592	RNF44																		Y		Y	Y																							
2946	A_23_P320200	RNF5			Y	Y	Y	Y		Y	Y	Y						Y	Y	Y	Y	Y	Y	Y	Y	Y	Y				Y															
8969	A_23_P8095	RNF5			Y	Y		Y		Y								Y	Y	Y		Y		Y			Y																			
21713	A_23_P162734	RNF6																		Y			Y																							
9133	A_23_P109768	RNF7																					Y																							
641	A_23_P70384	RNF8																									Y																			
9759	A_23_P204158	RNFT2			Y				Y	Y																																				
20412	A_23_P202672	RNH1			Y	Y	Y	Y		Y	Y	Y																																		
14156	A_23_P55515	RNMT			Y																						Y																			
13615	A_23_P97770	RNPEP																	Y	Y		Y																								
20943	A_23_P17025	RNPEPL1			Y	Y	Y	Y	Y	Y	Y	Y	Y	Y				Y	Y	Y		Y	Y	Y			Y	Y	Y	Y						Y										
16936	A_23_P152268	RNPS1																Y	Y	Y	Y	Y	Y	Y	Y	Y					Y															
9121	A_23_P255796	ROBO2			Y	Y		Y		Y								Y		Y							Y																			
13289	A_23_P87082	ROBO3																		Y		Y	Y																							
19287	A_23_P356581	ROBO3																									Y																			
3593	A_23_P344421	ROBO4																Y		Y			Y																							
15945	A_23_P130429	ROCK1			Y														Y	Y							Y	Y	Y	Y																
2335	A_23_P9192	ROD1			Y																		Y				Y																			
16066	A_23_P100499	ROGDI																Y	Y	Y		Y		Y																						
14348	A_23_P105002	ROM1																Y																												
3444	A_23_P166922	ROPN1							Y	Y	Y	Y	Y	Y																Y						Y										
15623	A_23_P121885	ROPN1L			Y	Y		Y	Y	Y																	Y	Y	Y	Y						Y										
846	A_23_P12363	ROR1							Y	Y																	Y			Y						Y										
21978	A_23_P59972	RP1			Y																		Y																							

4613	A_23_P71778	RP11-216L13.5			Y																																								
11356	A_23_P52286	RP11-529I10.4																Y	Y	Y		Y	Y	Y																					
10942	A_23_P217901	RP11-544M22.4																			Y		Y																						
1200	A_23_P384551	RP11-93B14.6			Y																						Y																		
6398	A_23_P643	RP1-21O18.1			Y													Y		Y		Y	Y								Y		Y	Y	Y										
18550	A_23_P72968	RP13-102H20.1																									Y																		
15420	A_23_P166147	RP13-401N8.2																	Y	Y		Y																							
16388	A_23_P309396	RP1L1																Y																											
3826	A_23_P22433	RP2																		Y		Y																							
20589	A_23_P143374	RP4-691N24.1			Y	Y		Y	Y	Y	Y	Y	Y	Y				Y	Y	Y		Y	Y	Y			Y																		
10023	A_23_P85993	RP5-1000E10.4																			Y																								
13423	A_23_P353704	RP5-1022P6.2																		Y																									
21609	A_23_P91350	RP5-1022P6.2																		Y								Y																	
21946	A_23_P429521	RP5-1022P6.6																Y	Y	Y		Y		Y			Y	Y	Y	Y															
4391	A_23_P114649	RP5-1077B9.4			Y	Y	Y	Y	Y	Y	Y	Y	Y	Y				Y	Y	Y		Y	Y	Y			Y	Y	Y	Y						Y									
3896	A_23_P148858	RP5-886K2.1									Y		Y	Y																															
8039	A_23_P305074	RP6-166C19.11			Y	Y		Y		Y										Y		Y	Y				Y	Y	Y	Y						Y									
16933	A_23_P255216	RP9																									Y																		
3210	A_23_P107073	RPA1			Y																Y									Y						Y									
3500	A_23_P86133	RPA2																Y																											
13690	A_23_P254214	RPA4				Y												Y		Y		Y																							
15303	A_23_P66758	RPAIN			Y	Y	Y	Y		Y	Y	Y						Y	Y	Y		Y	Y	Y			Y	Y	Y	Y															
19436	A_23_P3453	RPAP1				Y			Y	Y																																			
C11545.9	A_23_P2683	RPAP3				Y														Y																									
2286	A_23_P136963	RPGR			Y	Y	Y	Y	Y	Y	Y	Y	Y	Y				Y	Y	Y		Y	Y	Y			Y	Y	Y	Y						Y									
3452	A_23_P251593	RPL10A	Y	Y	Y	Y	Y	Y	Y	Y	Y	Y	Y	Y													Y	Y	Y	Y															
15767	A_23_P341325	RPL10L			Y														Y	Y			Y																						
17503	A_23_P99754	RPL10L			Y	Y	Y	Y		Y	Y	Y																																	
10924	A_23_P217068	RPL12	Y	Y	Y	Y	Y	Y	Y	Y	Y	Y	Y	Y				Y		Y							Y	Y	Y	Y						Y						Y				
8129	A_23_P501849	RPL13																												Y						Y									
16995	A_23_P90143	RPL13A			Y	Y		Y	Y	Y	Y	Y	Y	Y														Y		Y						Y						Y				
10243	A_23_P18292	RPL14	Y	Y	Y	Y		Y	Y	Y	Y	Y	Y	Y													Y	Y	Y	Y						Y								Y		
12521	A_23_P253192	RPL15			Y	Y		Y		Y										Y							Y	Y	Y	Y						Y									
22269	A_23_P168218	RPL15			Y																																								
21218	A_23_P107604	RPL17			Y	Y	Y	Y	Y	Y	Y	Y						Y	Y	Y			Y	Y			Y															Y				
5184	A_23_P142146	RPL18																										Y																	
19619	A_23_P90383	RPL18A			Y	Y	Y	Y		Y	Y	Y						Y	Y	Y		Y	Y	Y			Y															Y				
4132	A_23_P2725	RPL21			Y																																								
8659	A_23_P320636	RPL22			Y	Y		Y	Y	Y																																			
14579	A_23_P26717	RPL23			Y																						Y																		
10414	A_23_P108932	RPL23AP13	Y	Y	Y	Y		Y	Y	Y								Y		Y							Y	Y	Y	Y						Y									
6369	A_23_P256933	RPL24			Y	Y	Y	Y	Y	Y	Y	Y	Y	Y													Y	Y	Y	Y															
17649	A_23_P38283	RPL26			Y	Y		Y	Y	Y	Y	Y	Y	Y						Y							Y	Y	Y	Y															
3394	A_23_P156265	RPL26L1																												Y						Y									
12021	A_23_P141387	RPL27			Y	Y		Y	Y	Y	Y	Y	Y	Y					Y								Y	Y	Y	Y						Y								Y		
8137	A_23_P203577	RPL27A			Y	Y		Y	Y	Y	Y	Y	Y	Y													Y	Y	Y	Y						Y								Y		
17166	A_23_P208356	RPL28			Y													Y	Y	Y		Y	Y	Y			Y																		
3299	A_23_P68942	RPL3			Y				Y	Y	Y	Y	Y	Y													Y																	Y		
7079	A_23_P210032	RPL31			Y				Y	Y									Y	Y			Y				Y																		
13230	A_23_P18149	RPL32			Y	Y		Y	Y	Y										Y							Y																		
7523	A_23_P7224	RPL34			Y	Y	Y	Y	Y	Y	Y	Y	Y	Y						Y							Y	Y	Y	Y												Y				
6148	A_23_P44956	RPL35A			Y	Y	Y	Y	Y	Y	Y	Y	Y	Y													Y	Y	Y	Y						Y									
16857	A_23_P217609	RPL36A	Y	Y	Y	Y		Y	Y	Y	Y	Y	Y	Y													Y	Y	Y	Y						Y						Y				
15532	A_23_P25902	RPL36AL			Y																																								
8535	A_23_P58721	RPL37			Y																																								
6384	A_23_P29595	RPL39L			Y	Y		Y	Y	Y	Y	Y	Y	Y					Y	Y							Y	Y	Y	Y															
8345	A_23_P118095	RPL3L				Y																																							
5495	A_23_P69429	RPL4			Y	Y		Y	Y	Y																	Y	Y	Y	Y															
9547	A_23_P12133	RPL5			Y															Y																									
3465	A_23_P151321	RPL6				Y																																							
4275	A_23_P146321	RPL7			Y	Y		Y	Y	Y																	Y	Y	Y	Y						Y									
12952	A_23_P31657	RPL8			Y				Y	Y	Y	Y	Y	Y																Y						Y									
16339	A_23_P7066	RPL9			Y				Y	Y										Y							Y																		
20797	A_23_P204477	RPLP0			Y				Y	Y	Y	Y	Y	Y													Y	Y	Y	Y						Y									
265	A_23_P132803	RPN1																Y		Y							Y																		
5159	A_23_P120390	RPN2			Y													Y									Y																		
2251	A_23_P211864	RPP14			Y																																								
17818	A_23_P214594	RPP21			Y															Y							Y			Y						Y									
9706	A_23_P3424	RPP25			Y	Y	Y	Y	Y	Y	Y	Y						Y	Y	Y		Y	Y	Y			Y	Y	Y	Y						Y									
20177	A_23_P150080	RPP38			Y	Y		Y		Y																	Y	Y	Y	Y						Y									
6515	A_23_P111297	RPP40																Y																											
2895	A_23_P55641	RPRD1A			Y	Y		Y		Y										Y			Y																						
9336	A_23_P91207	RPRD1B			Y				Y	Y																	Y			Y						Y									
10536	A_23_P82128	RPS12			Y				Y	Y																																			
13643	A_23_P24763	RPS13			Y	Y		Y	Y	Y										Y			Y				Y	Y	Y	Y						Y									
11840	A_23_P81492	RPS14			Y	Y		Y	Y	Y	Y	Y	Y	Y													Y			Y						Y									
9383	A_23_P209152	RPS15	Y	Y	Y	Y		Y	Y	Y	Y	Y	Y	Y													Y			Y						Y									

15636	A_23_P49286	RPS15A			Y	Y		Y	Y	Y	Y	Y	Y	Y													Y			Y						Y										
20596	A_23_P119130	RPS19			Y													Y	Y	Y		Y		Y			Y																				
10347	A_23_P106702	RPS2			Y																							Y																				
4113	A_23_P1206	RPS24			Y	Y		Y		Y																	Y																				
22546	A_23_P47357	RPS25			Y	Y	Y	Y	Y	Y	Y	Y	Y	Y						Y			Y				Y	Y	Y	Y							Y										
3549	A_23_P79737	RPS27A			Y	Y	Y	Y	Y	Y	Y	Y	Y	Y													Y	Y	Y	Y																
5014	A_23_P14734	RPS27L			Y	Y		Y		Y										Y							Y																				
13918	A_23_P142479	RPS28			Y	Y		Y	Y	Y	Y	Y	Y	Y													Y			Y						Y										
13301	A_23_P76961	RPS29			Y	Y		Y	Y	Y	Y	Y	Y	Y																Y						Y										
4586	A_23_P144497	RPS3A			Y	Y		Y	Y	Y																Y																				
12795	A_23_P125519	RPS4X	Y	Y	Y	Y	Y	Y	Y	Y	Y	Y	Y	Y				Y		Y		Y					Y	Y	Y	Y							Y								Y		
1976	A_23_P259314	RPS4Y1	Y	Y	Y	Y	Y	Y	Y	Y	Y	Y	Y	Y													Y	Y	Y	Y																
19140	A_23_P324384	RPS4Y2	Y	Y	Y	Y	Y	Y	Y	Y	Y	Y	Y	Y					Y								Y	Y	Y	Y							Y										
12394	A_23_P208260	RPS5			Y																																										
11677	A_23_P52086	RPS6KA1			Y	Y		Y	Y	Y										Y			Y				Y	Y	Y	Y																
14930	A_23_P335920	RPS6KA2																					Y																										
17103	A_23_P35787	RPS6KA4			Y	Y	Y	Y	Y	Y	Y	Y	Y	Y				Y	Y	Y		Y	Y	Y			Y	Y	Y	Y							Y										
18291	A_23_P37375	RPS6KA5			Y	Y	Y	Y	Y	Y	Y	Y	Y	Y					Y	Y	Y	Y	Y				Y	Y	Y	Y							Y								Y		
1207	A_23_P125596	RPS6KA6																		Y	Y																											
15607	A_23_P141329	RPS6KB1																										Y																				
21517	A_23_P24318	RPS6KB2			Y	Y		Y		Y								Y	Y		Y		Y																								
12518	A_23_P104105	RPS6KC1			Y	Y		Y		Y												Y				Y	Y	Y	Y																
14552	A_23_P428958	RPS6KL1			Y	Y	Y	Y	Y	Y	Y	Y	Y	Y				Y	Y	Y	Y	Y	Y	Y	Y	Y	Y			Y	Y	Y				Y										
22364	A_23_P141549	RPS7							Y	Y																																						
17383	A_23_P208535	RPS9				Y																																										
5101	A_23_P254415	RPSA			Y	Y		Y		Y																																						
18861	A_23_P49269	RPUSD1			Y	Y	Y	Y	Y	Y	Y	Y	Y	Y				Y	Y	Y	Y	Y	Y	Y	Y	Y	Y	Y	Y	Y	Y	Y				Y										
1837	A_23_P255916	RPUSD3			Y	Y	Y	Y	Y	Y	Y	Y	Y	Y				Y	Y	Y		Y		Y			Y	Y	Y	Y	Y	Y	Y	Y	Y	Y												
4394	A_23_P75540	RPUSD4			Y																																										
5658	A_23_P388840	RQCD1			Y																								Y		Y						Y										
18446	A_23_P5464	RQCD1			Y	Y		Y	Y	Y	Y	Y	Y	Y				Y		Y		Y	Y				Y	Y	Y	Y							Y										
12969	A_23_P169117	RRAGA							Y	Y																			Y						Y										
20901	A_23_P73717	RRAGB																	Y	Y																											
2731	A_23_P133684	RRAGD	Y						Y	Y										Y										Y						Y										
3523	A_23_P39076	RRAS			Y	Y	Y	Y	Y	Y	Y	Y						Y	Y	Y		Y	Y	Y			Y	Y	Y	Y							Y										
2555	A_23_P405761	RRAS2							Y	Y																		Y		Y						Y										
8613	A_23_P202852	RRAS2																Y																															
14479	A_23_P120566	RRBP1			Y	Y		Y		Y								Y		Y	Y		Y				Y																				
2728	A_23_P59293	RREB1																		Y		Y	Y																										
16353	A_23_P87351	RRM1			Y	Y	Y	Y	Y	Y	Y	Y								Y			Y				Y	Y	Y	Y																
11545	A_23_P136222	RRM2	Y	Y																										Y						Y										
20824	A_23_P206877	RRN3			Y																																										
22032	A_23_P80129	RRP1			Y	Y	Y	Y	Y	Y	Y	Y						Y	Y	Y		Y	Y	Y			Y	Y	Y	Y							Y										
1298	A_23_P256297	RRP1B							Y	Y																																						
10558	A_23_P29305	RRP7A			Y	Y	Y	Y	Y	Y	Y	Y	Y	Y				Y	Y	Y	Y	Y	Y	Y	Y	Y	Y	Y	Y	Y	Y	Y				Y										
20308	A_23_P6801	RRP9			Y	Y		Y		Y										Y	Y	Y	Y				Y	Y	Y	Y																
21751	A_23_P146186	RRS1																		Y																													
844	A_23_P120178	RSAD2	Y	Y		Y																																										
9610	A_23_P137957	RSBN1			Y															Y			Y																										
17618	A_23_P395534	RSC1A1				Y												Y	Y	Y	Y	Y	Y	Y	Y	Y					Y		Y	Y	Y											
22196	A_23_P139388	RSF1			Y	Y		Y	Y	Y									Y	Y		Y					Y			Y						Y										
8937	A_23_P102950	RSPH1	Y	Y	Y	Y		Y	Y	Y	Y	Y	Y	Y				Y		Y	Y	Y	Y				Y	Y	Y	Y							Y										
2511	A_23_P59397	RSPH3			Y				Y	Y										Y	Y		Y										Y													
20257	A_23_P258294	RSPO2			Y																																										
10497	A_23_P53267	RSRC2			Y	Y	Y	Y	Y	Y	Y	Y								Y							Y	Y	Y	Y																
14575	A_23_P138417	RSU1	Y	Y	Y	Y		Y	Y	Y	Y	Y	Y	Y	Y			Y	Y	Y		Y	Y	Y			Y	Y	Y	Y							Y										
18081	A_23_P149813	RSU1			Y	Y		Y	Y	Y	Y	Y	Y	Y						Y		Y					Y			Y						Y										
2451	A_23_P130653	RTBDN																					Y							Y						Y										
3640	A_23_P258621	RTCD1	Y	Y																																											
8131	A_23_P77321	RTF1																				Y					Y																				
17141	A_23_P52410	RTKN2																Y									Y																				
9880	A_23_P89902	RTN2				Y																																										
4450	A_23_P56930	RTN4																					Y																										
19276	A_23_P122775	RTN4IP1	Y	Y	Y													Y	Y								Y			Y						Y										
18540	A_23_P132175	RTN4R			Y	Y		Y		Y								Y	Y	Y	Y	Y	Y	Y	Y	Y	Y				Y															
5400	A_23_P66477	RTN4RL1	Y	Y	Y				Y	Y	Y	Y	Y	Y					Y				Y				Y			Y						Y						Y			
16471	A_23_P432056	RTN4RL1			Y				Y	Y																	Y																				
16545	A_23_P360413	RTN4RL2			Y	Y		Y	Y	Y										Y		Y	Y				Y			Y						Y										
14116	A_23_P57910	RTP3																		Y																													
9166	A_23_P337896	RTTN			Y	Y		Y		Y																		Y																			
20807	A_23_P66928	RTTN			Y	Y	Y	Y	Y	Y	Y	Y							Y	Y	Y	Y																							
2602	A_23_P73457	RUFY1	Y	Y	Y													Y		Y			Y				Y																				
3165	A_23_P250754	RUFY1	Y																	Y							Y																				
3480	A_23_P435546	RUFY2				Y			Y	Y																																						
22467	A_23_P212914	RUFY3																		Y								Y																			
18990	A_23_P129996	RUNDC1			Y				Y	Y																																						
13666	A_23_P49132	RUNDC2A			Y	Y	Y	Y	Y	Y	Y	Y	Y	Y						Y		Y	Y				Y			Y						Y										

20042	A_23_P66579	RUNDC3A																	Y	Y			Y																							
2844	A_23_P111724	RUNDC3B			Y				Y	Y																																					
22267	A_23_P12024	RUSC1			Y													Y	Y	Y		Y	Y	Y			Y																				
21281	A_23_P39110	RUVBL2			Y	Y	Y	Y		Y	Y	Y						Y	Y	Y		Y		Y			Y	Y	Y	Y						Y											
10120	A_23_P167834	RWDD1																Y	Y	Y	Y	Y	Y	Y	Y	Y					Y																
3928	A_23_P255076	RWDD2A			Y	Y		Y		Y																	Y																				
13376	A_23_P91479	RWDD2B																			Y		Y																								
5126	A_23_P933	RWDD3							Y	Y										Y								Y		Y						Y											
4367	A_23_P394276	RWDD4A			Y	Y	Y	Y	Y	Y	Y	Y						Y	Y	Y	Y	Y		Y			Y	Y	Y	Y						Y											
8012	A_23_P117261	RXFP2							Y	Y									Y				Y																								
9694	A_23_P29365	RYBP			Y	Y	Y	Y	Y	Y	Y	Y	Y	Y					Y	Y		Y	Y				Y	Y	Y	Y						Y											
18547	A_23_P305711	RYBP			Y	Y	Y	Y	Y	Y	Y	Y	Y	Y						Y							Y	Y	Y	Y						Y											
14186	A_23_P388081	RYK							Y	Y																																					
8703	A_23_P78867	RYR1				Y												Y	Y	Y	Y		Y	Y	Y																						
8271	A_23_P94838	RYR3																									Y																				
10407	A_23_P383227	S100A1			Y				Y	Y									Y																												
288	A_23_P137984	S100A10				Y																	Y																								
17422	A_23_P145863	S100A11			Y	Y		Y		Y								Y		Y		Y					Y	Y	Y	Y						Y											
18381	A_23_P126594	S100A11			Y													Y									Y																				
8078	A_23_P74001	S100A12			Y																						Y																				
13042	A_23_P372874	S100A13			Y																Y						Y																				
9214	A_23_P124619	S100A14																									Y																				
2112	A_23_P92122	S100A1L																Y																													
18720	A_23_P337070	S100A2																			Y																										
1022	A_23_P104071	S100A3																												Y						Y											
4868	A_23_P200288	S100A8			Y	Y		Y	Y	Y																	Y	Y	Y	Y						Y											
14403	A_23_P434809	S100A8	Y	Y	Y	Y	Y	Y	Y	Y	Y	Y						Y									Y	Y	Y	Y						Y											
2469	A_23_P23044	S100A9			Y	Y	Y	Y	Y	Y	Y	Y	Y	Y									Y				Y	Y	Y	Y						Y											
7015	A_23_P143529	S100B																	Y											Y						Y											
8696	A_23_P44996	S100G				Y																					Y			Y						Y											
15338	A_23_P58266	S100P																Y																													
14347	A_23_P338952	S100PBP			Y	Y	Y	Y	Y	Y	Y	Y	Y	Y						Y		Y	Y				Y	Y	Y	Y						Y											
10474	A_23_P81630	S100Z			Y															Y																											
17556	A_23_P160117	S1PR1	Y		Y													Y	Y	Y	Y		Y	Y	Y								Y	Y													
18874	A_23_P404481	S1PR1	Y	Y	Y										Y			Y	Y	Y		Y	Y	Y			Y																				
16586	A_23_P16089	S1PR2																									Y			Y						Y											
11884	A_23_P119502	S1PR4	Y	Y	Y				Y	Y								Y	Y								Y																				
5196	A_23_P107744	S1PR5																Y	Y	Y				Y																							
4836	A_23_P345128	SAA1							Y	Y																	Y			Y						Y											
21740	A_23_P306203	SAA2							Y	Y																		Y		Y						Y											
C10424.3	A_23_P87238	SAA4							Y	Y																		Y		Y						Y											
21611	A_23_P321349	SAAL1																					Y																								
631	A_23_P348298	SAC3D1			Y	Y		Y		Y								Y	Y	Y		Y		Y			Y			Y						Y											
19135	A_23_P116057	SAC3D1			Y	Y		Y		Y									Y	Y		Y	Y				Y																				
8469	A_23_P212383	SACM1L				Y														Y			Y							Y						Y											
839	A_23_P128663	SACS							Y	Y								Y	Y																												
3489	A_23_P308073	SACS							Y	Y								Y	Y	Y		Y		Y																							
12350	A_23_P16339	SAE1																									Y																				
17119	A_23_P208446	SAFB																		Y	Y		Y																								
12998	A_23_P5853	SAG			Y	Y		Y	Y	Y													Y				Y			Y						Y											
6908	A_23_P21943	SAGE1	Y	Y	Y													Y									Y			Y						Y											
7270	A_23_P33343	SAGE1	Y	Y	Y													Y									Y																				
13425	A_23_P40129	SAMD10																		Y			Y																								
2794	A_23_P61438	SAMD13							Y	Y																	Y																				
18927	A_23_P403500	SAMD14			Y	Y		Y		Y													Y				Y			Y						Y											
15403	A_23_P93524	SAMD3	Y	Y																																											
6915	A_23_P302081	SAMD4A				Y														Y			Y																								
1185	A_23_P67496	SAMD4B			Y																	Y					Y																				
15580	A_23_P63873	SAMD8			Y																																										
14612	A_23_P17341	SAMHD1	Y	Y	Y															Y	Y																										
1726	A_23_P29321	SAMM50			Y				Y	Y												Y																									
3821	A_23_P29005	SAMSN1	Y	Y																																											
17696	A_23_P154188	SAP130																		Y	Y																										
8413	A_23_P121602	SAP30																					Y																								
10949	A_23_P54950	SAP30BP	Y	Y	Y	Y		Y		Y								Y		Y		Y	Y				Y																				
12762	A_23_P368101	SAP30L			Y	Y		Y		Y								Y	Y	Y	Y	Y	Y	Y	Y	Y	Y				Y																
17774	A_23_P30509	SAP30L				Y																					Y	Y	Y	Y																	
11520	A_23_P119448	SAPS1			Y																		Y																								
19828	A_23_P127175	SAR1A			Y	Y		Y		Y					Y												Y																				
22125	A_23_P135079	SARDH			Y	Y		Y		Y										Y																											
2826	A_23_P90484	SARS2							Y	Y								Y																													
16408	A_23_P86884	SART1			Y													Y	Y	Y	Y	Y	Y	Y	Y	Y	Y				Y																
21269	A_23_P87664	SART3																Y	Y	Y	Y		Y	Y	Y																						
21615	A_23_P93442	SASH1				Y														Y		Y						Y																			
4490	A_23_P137016	SAT1	Y		Y																																										
9003	A_23_P15402	SAT2																Y	Y	Y				Y			Y																				
8612	A_23_P259741	SATB1																	Y																												

14183	A_23_P72297	SATB1							Y	Y																					Y		Y						Y										
9765	A_23_P65555	SAV1																	Y																										
21574	A_23_P70874	SBDS																	Y	Y									Y																
16508	A_23_P109606	SBF1			Y	Y	Y	Y	Y	Y	Y	Y	Y	Y				Y	Y	Y	Y	Y	Y	Y	Y	Y	Y	Y	Y	Y	Y	Y				Y				Y						
10586	A_23_P98731	SBF2	Y	Y	Y				Y	Y								Y	Y	Y				Y			Y			Y						Y										
15395	A_23_P419794	SBF2			Y	Y		Y	Y	Y									Y	Y							Y	Y	Y	Y						Y										
1470	A_23_P66174	SBK1			Y	Y	Y	Y	Y	Y	Y	Y	Y	Y					Y	Y			Y				Y	Y	Y	Y						Y										
3933	A_23_P5273	SBNO2			Y	Y	Y	Y	Y	Y	Y	Y	Y	Y				Y	Y	Y		Y	Y	Y			Y			Y						Y										
2146	A_23_P108251	SBSN			Y	Y		Y		Y																																				
12555	A_23_P110184	SC4MOL				Y																																								
1006	A_23_P98446	SC5DL									Y		Y	Y						Y																										
8536	A_23_P372888	SC5DL									Y		Y	Y						Y																										
8818	A_23_P38525	SC65																Y	Y	Y	Y	Y	Y	Y	Y	Y					Y															
11448	A_23_P110787	SCAMP1																					Y					Y																		
1931	A_23_P106451	SCAMP2				Y			Y	Y										Y																										
11222	A_23_P385079	SCAMP2			Y	Y		Y	Y	Y								Y	Y	Y	Y	Y	Y	Y	Y	Y					Y															
5176	A_23_P97271	SCAMP3																				Y																								
1465	A_23_P208957	SCAMP4				Y														Y			Y																							
406	A_23_P152047	SCAMP5																Y		Y																										
13772	A_23_P210977	SCAND1																		Y																										
21489	A_23_P205875	SCAND2																				Y					Y																			
5995	A_23_P252825	SCAP			Y	Y		Y	Y	Y								Y	Y	Y		Y	Y	Y			Y																			
3426	A_23_P163408	SCAPER																		Y	Y	Y	Y																							
16039	A_23_P94103	SCARA5																					Y																							
6783	A_23_P369201	SCARB1																				Y																								
22463	A_23_P203900	SCARB1			Y				Y	Y																																				
5844	A_23_P254692	SCARB2			Y																							Y																		
22313	A_23_P15414	SCARF1				Y																								Y						Y										
16759	A_23_P132185	SCARF2							Y	Y								Y		Y		Y	Y																							
3565	A_23_P62807	SCCPDH	Y	Y	Y	Y	Y	Y	Y	Y	Y	Y															Y	Y	Y	Y																
123	A_23_P213288	SCD5							Y	Y																																				
6150	A_23_P500000	SCEL			Y				Y	Y										Y							Y			Y						Y										
20337	A_23_P88209	SCFD1																		Y																										
21910	A_23_P54267	SCG3				Y																					Y	Y	Y	Y						Y										
2927	A_23_P62081	SCG5			Y																																									
17509	A_23_P84879	SCG5																									Y			Y						Y										
21666	A_23_P73480	SCG5																					Y							Y						Y										
7249	A_23_P150583	SCGB1A1																												Y						Y										
565	A_23_P150555	SCGB1D2			Y	Y		Y		Y																	Y																			
19450	A_23_P161940	SCGB2A2			Y	Y		Y	Y	Y																		Y		Y						Y										
11515	A_23_P30213	SCGB3A1			Y																																									
7548	A_23_P145006	SCGB3A2																									Y																			
15316	A_23_P257031	SCHIP1			Y				Y	Y	Y	Y	Y	Y				Y		Y							Y			Y						Y										
15453	A_23_P18656	SCLT1			Y																																									
14108	A_23_P12477	SCMH1			Y	Y	Y	Y		Y	Y	Y	Y					Y	Y								Y																			
14283	A_23_P96317	SCML1																	Y	Y		Y																								
16848	A_23_P8293	SCML4	Y	Y																							Y																			
3620	A_23_P158555	SCN10A			Y	Y		Y		Y										Y							Y	Y	Y	Y																
21064	A_23_P79015	SCN1B			Y													Y	Y											Y						Y										
2316	A_23_P13157	SCN2B			Y														Y								Y	Y	Y	Y						Y										
14393	A_23_P67896	SCN3A				Y																																								
22198	A_23_P141265	SCN4A			Y	Y	Y	Y	Y	Y	Y	Y								Y							Y			Y						Y										
16322	A_23_P303833	SCN4B			Y																						Y																			
6861	A_23_P43860	SCN5A			Y	Y		Y		Y												Y																								
14333	A_23_P102331	SCN7A							Y	Y																		Y																		
17178	A_23_P76243	SCN8A																Y																												
9365	A_23_P112726	SCN9A			Y				Y	Y								Y									Y			Y						Y										
5943	A_23_P128323	SCNN1A				Y			Y	Y									Y	Y																										
16099	A_23_P26524	SCNN1B			Y	Y		Y		Y																	Y	Y	Y	Y						Y										
10322	A_23_P46412	SCNN1D	Y	Y	Y	Y	Y	Y	Y	Y	Y	Y						Y	Y	Y		Y		Y				Y		Y						Y						Y				
7645	A_23_P206626	SCNN1G																Y	Y	Y				Y				Y																		
19367	A_23_P15466	SCO1																												Y						Y										
3956	A_23_P132388	SCO2			Y													Y	Y	Y				Y																						
18803	A_23_P152548	SCPEP1			Y	Y		Y	Y	Y																	Y			Y						Y										
8308	A_23_P167159	SCRG1							Y	Y									Y											Y						Y										
848	A_23_P159039	SCRIB																Y	Y	Y	Y	Y	Y	Y	Y	Y	Y				Y															
2371	A_23_P136265	SCRIB																Y	Y	Y	Y	Y	Y	Y	Y	Y					Y															
21654	A_23_P124873	SCRIB			Y	Y		Y	Y	Y								Y	Y	Y	Y	Y	Y	Y	Y	Y	Y				Y															
2367	A_23_P215355	SCRN1			Y													Y	Y	Y		Y		Y																						
13627	A_23_P17015	SCRN3																	Y	Y		Y	Y																							
11681	A_23_P147782	SCRT1																		Y			Y																							
19514	A_23_P104594	SCT																										Y																		
16080	A_23_P105144	SCUBE2	Y	Y	Y	Y		Y	Y	Y	Y	Y	Y	Y				Y	Y	Y		Y	Y	Y			Y	Y	Y	Y						Y										
18589	A_23_P75468	SCYL1			Y	Y	Y	Y	Y	Y	Y	Y	Y	Y				Y	Y	Y	Y	Y	Y	Y	Y	Y	Y			Y	Y	Y				Y				Y						
6487	A_23_P368145	SCYL1BP1			Y	Y		Y	Y	Y	Y	Y	Y	Y				Y	Y	Y	Y	Y	Y	Y	Y	Y	Y			Y	Y	Y				Y										
13837	A_23_P74320	SCYL3			Y	Y		Y	Y	Y	Y	Y	Y	Y				Y	Y	Y	Y	Y	Y	Y	Y	Y	Y	Y	Y	Y	Y	Y				Y				Y						
16061	A_23_P16944	SDC1			Y				Y	Y										Y										Y						Y										

8726	A_23_P8867	SDC2			Y		Y		Y	Y	Y	Y	Y	Y					Y				Y							Y						Y										
16359	A_23_P74878	SDC3																										Y																		
10866	A_23_P157576	SDCBP																				Y																								
11777	A_23_P168972	SDCBP																	Y																											
12383	A_23_P131899	SDCBP2			Y	Y		Y	Y	Y	Y	Y	Y	Y					Y	Y								Y														Y				
13748	A_23_P140328	SDCCAG1			Y	Y	Y	Y	Y	Y	Y	Y	Y	Y				Y	Y	Y		Y	Y	Y				Y	Y	Y	Y															
5216	A_23_P92629	SDCCAG10			Y	Y	Y	Y	Y	Y	Y	Y	Y	Y					Y	Y		Y						Y	Y	Y	Y													Y		
6532	A_23_P258272	SDCCAG3			Y	Y	Y	Y		Y	Y	Y							Y	Y		Y	Y																							
7377	A_23_P34546	SDCCAG8							Y	Y									Y	Y											Y						Y										
4243	A_23_P107311	SDF2			Y	Y		Y		Y																																				
17692	A_23_P6344	SDF2L1			Y				Y	Y			Y															Y			Y						Y										
6231	A_23_P201338	SDF4			Y	Y		Y		Y									Y	Y		Y						Y	Y	Y	Y															
20403	A_23_P250035	SDHA			Y	Y		Y		Y								Y	Y	Y	Y	Y	Y	Y	Y	Y		Y	Y	Y	Y	Y	Y													
9578	A_23_P149649	SDHB			Y	Y		Y		Y																																				
17948	A_23_P12245	SDHC			Y	Y		Y		Y																																				
8160	A_23_P89691	SDK2			Y	Y	Y	Y	Y	Y	Y	Y							Y	Y	Y	Y	Y					Y																		
6360	A_23_P257457	SDR16C5			Y	Y		Y	Y	Y	Y	Y	Y	Y																																
19719	A_23_P25091	SDR9C7			Y																																									
16052	A_23_P2645	SDS			Y													Y	Y	Y		Y	Y	Y				Y																		
18891	A_23_P53439	SDSL				Y			Y	Y								Y	Y	Y		Y	Y	Y																						
9910	A_23_P208009	SEC11C							Y	Y										Y																										
17123	A_23_P206960	SEC14L1	Y	Y	Y	Y	Y	Y	Y	Y	Y	Y																Y																		
5908	A_23_P17808	SEC14L2							Y	Y																					Y						Y										
962	A_23_P77704	SEC14L5			Y																							Y																		
6935	A_23_P251303	SEC16A							Y	Y	Y	Y	Y	Y																																
12808	A_23_P51918	SEC16B							Y	Y								Y		Y	Y	Y	Y								Y						Y										
20418	A_23_P259874	SEC22A	Y	Y																																										
18257	A_23_P211997	SEC22C				Y																						Y	Y	Y	Y															
22451	A_23_P6116	SEC23B			Y				Y	Y																																				
5882	A_23_P202602	SEC23IP	Y	Y	Y	Y	Y	Y	Y	Y	Y	Y	Y	Y														Y	Y	Y	Y						Y										
12794	A_23_P69680	SEC24B			Y	Y	Y	Y		Y	Y	Y							Y	Y		Y	Y					Y														Y				
19545	A_23_P138545	SEC24C																		Y			Y																							
11873	A_23_P170273	SEC31A																	Y			Y																								
15586	A_23_P35564	SEC31B			Y	Y	Y	Y	Y	Y	Y	Y						Y	Y	Y		Y		Y				Y	Y	Y	Y						Y										
7355	A_23_P212457	SEC61A1			Y	Y	Y	Y	Y	Y	Y	Y						Y	Y	Y	Y	Y	Y	Y	Y	Y		Y				Y														
11280	A_23_P135342	SEC61B			Y	Y		Y	Y	Y																																				
18052	A_23_P357860	SEC62				Y			Y	Y																					Y						Y										
4996	A_23_P214977	SEC63				Y													Y			Y						Y	Y	Y	Y															
5428	A_23_P84870	SECISBP2			Y	Y	Y	Y	Y	Y	Y	Y	Y	Y				Y	Y	Y		Y	Y	Y				Y	Y	Y	Y													Y		
19013	A_23_P207905	SECTM1			Y	Y		Y	Y	Y	Y	Y	Y	Y					Y	Y		Y							Y		Y						Y										
15891	A_23_P78311	SEH1L							Y	Y																		Y																		
1900	A_23_P88411	SEL1L																													Y						Y										
17590	A_23_P74625	SELENBP1																	Y																											
14767	A_23_P68300	SELI																	Y	Y			Y																							
16646	A_23_P364517	SELK							Y	Y																		Y																		
21478	A_23_P114921	SELL				Y																																								
7696	A_23_P126303	SELP			Y		Y		Y	Y	Y	Y							Y									Y																		
15035	A_23_P64860	SELPLG																					Y					Y																		
4016	A_23_P26084	SELS				Y			Y	Y	Y	Y	Y	Y					Y	Y																										
7912	A_23_P250042	SELT			Y																																									
16662	A_23_P250853	SEMA3A																Y	Y										Y																	
2096	A_23_P132718	SEMA3B	Y	Y	Y	Y		Y	Y	Y								Y		Y								Y	Y	Y	Y						Y										
15336	A_23_P256473	SEMA3C			Y	Y		Y	Y	Y	Y	Y	Y	Y					Y	Y		Y						Y	Y	Y	Y						Y										
3304	A_23_P405287	SEMA3D																			Y																									
3763	A_23_P59578	SEMA3E			Y	Y		Y	Y	Y																																				
15804	A_23_P6878	SEMA3F																Y																												
16451	A_23_P147907	SEMA4D	Y		Y	Y		Y		Y					Y			Y	Y	Y	Y		Y	Y	Y			Y	Y	Y	Y															
1112	A_23_P165608	SEMA4F							Y	Y																																				
16732	A_23_P213415	SEMA5A	Y	Y	Y																							Y	Y	Y	Y						Y										
8855	A_23_P121374	SEMA5B																		Y			Y																							
14485	A_23_P7752	SEMA6A			Y	Y		Y	Y	Y	Y	Y	Y	Y				Y	Y	Y				Y				Y	Y	Y	Y						Y										
19274	A_23_P208905	SEMA6B			Y	Y		Y		Y										Y		Y	Y					Y																		
4236	A_23_P138001	SEMA6C			Y														Y	Y			Y					Y			Y						Y										
13291	A_23_P106389	SEMA7A				Y																																								
16502	A_23_P204536	SENP1			Y	Y		Y	Y	Y										Y			Y					Y																		
17858	A_23_P62065	SENP2																					Y																							
13672	A_23_P163997	SENP3			Y	Y	Y	Y	Y	Y	Y	Y	Y	Y						Y		Y						Y			Y						Y										
7590	A_23_P29504	SENP5																					Y																							
22397	A_23_P214151	SENP6			Y	Y	Y	Y	Y	Y	Y	Y							Y	Y		Y						Y	Y	Y	Y						Y										
14456	A_23_P6714	SENP7			Y	Y		Y	Y	Y									Y	Y		Y	Y						Y																	
12100	A_23_P334263	SENP8			Y																							Y																		
2188	A_23_P150092	SEPHS1	Y	Y	Y	Y		Y	Y	Y								Y	Y	Y		Y		Y				Y	Y	Y	Y						Y										
3017	A_23_P146798	SEPHS2							Y	Y																																				
3464	A_23_P370625	SEPN1																		Y		Y																								
16881	A_23_P121926	SEPP1							Y	Y																					Y						Y										
4291	A_23_P167198	SEPSECS																		Y		Y	Y																							
10982	A_23_P9848	SEPT10_			Y																							Y																		

19761	A_23_P29124	SEPT3_							Y	Y										Y																										
8381	A_23_P207003	SEPT6_			Y													Y	Y	Y		Y	Y	Y			Y																			
10686	A_23_P17724	SEPT7_																Y		Y	Y	Y	Y																							
17452	A_23_P43175	SEPT9_			Y				Y	Y									Y	Y		Y					Y			Y						Y										
20386	A_23_P211572	SEPT9_							Y	Y	Y	Y	Y	Y					Y											Y						Y										
1129	A_23_P129490	SEPX1			Y	Y		Y	Y	Y										Y																										
620	A_23_P359111	SERBP1																												Y						Y										
17706	A_23_P200661	SERBP1																		Y										Y						Y										
8729	A_23_P69873	SERF1B																												Y						Y										
9113	A_23_P106294	SERF2			Y				Y	Y	Y	Y	Y	Y																Y						Y										
18910	A_23_P139207	SERGEF																		Y		Y	Y																							
17430	A_23_P120955	SERHL							Y	Y																				Y						Y										
2428	A_23_P253131	SERINC1				Y							Y							Y								Y																		
5490	A_23_P147326	SERINC2							Y	Y								Y	Y																											
6367	A_23_P79836	SERINC3																									Y																			
15249	A_23_P435496	SERINC3																			Y																									
20625	A_23_P91919	SERP1			Y															Y			Y																							
14753	A_23_P139965	SERP2	Y	Y	Y	Y	Y	Y		Y	Y	Y	Y	Y						Y		Y	Y				Y	Y	Y	Y						Y						Y				
4520	A_23_P128759	SERPINA10																Y																												
21590	A_23_P105974	SERPINA11			Y																																									
10581	A_23_P162916	SERPINA3																					Y																							
18933	A_23_P25720	SERPINA4				Y					Y		Y	Y														Y																		
22475	A_23_P205355	SERPINA5	Y	Y	Y	Y		Y	Y	Y								Y	Y			Y					Y	Y	Y	Y						Y										
19345	A_23_P117363	SERPINA6			Y																	Y																								
21167	A_23_P257307	SERPINA7																Y		Y																										
15279	A_23_P214330	SERPINB1																									Y																			
21139	A_23_P313981	SERPINB10							Y	Y																																				
1385	A_23_P107623	SERPINB12																					Y																							
14080	A_23_P119018	SERPINB13																			Y																									
19369	A_23_P55632	SERPINB3							Y	Y																				Y						Y										
475	A_23_P502413	SERPINB4																												Y						Y										
4828	A_23_P208126	SERPINB5							Y	Y																				Y						Y										
8600	A_23_P70348	SERPINB6																					Y																							
2846	A_23_P4561	SERPINB8				Y			Y	Y																		Y		Y						Y										
22323	A_23_P30687	SERPINB9							Y	Y																																				
18162	A_23_P114626	SERPINC1	Y	Y	Y	Y	Y	Y	Y	Y	Y	Y	Y	Y				Y	Y	Y		Y	Y	Y			Y	Y	Y	Y																
C10884.2	A_23_P6335	SERPIND1	Y	Y	Y						Y		Y	Y				Y		Y							Y																			
21644	A_23_P162861	SERPINE3				Y																						Y																		
20452	A_23_P100660	SERPINF1																		Y			Y										Y													
11662	A_23_P89270	SERPINF2																		Y																										
1547	A_23_P75998	SERPINH1			Y				Y	Y								Y		Y	Y	Y	Y				Y																			
4530	A_23_P166929	SERPINI1																					Y																							
15328	A_23_P132826	SERPINI2			Y														Y																											
5640	A_23_P56952	SERTAD2																Y																												
13212	A_23_P141954	SERTAD3																									Y			Y						Y										
6777	A_23_P318904	SERTAD4				Y																																								
17028	A_23_P93562	SESN1				Y			Y	Y	Y	Y	Y	Y						Y										Y						Y										
7395	A_23_P361448	SESN3			Y	Y		Y		Y								Y	Y	Y		Y		Y				Y																		
4188	A_23_P60971	SESTD1			Y	Y		Y	Y	Y									Y	Y			Y					Y																		
22188	A_23_P367610	SESTD1							Y	Y																																				
6248	A_23_P4551	SETBP1	Y	Y	Y	Y		Y	Y	Y								Y		Y		Y					Y	Y	Y	Y						Y										
15952	A_23_P129673	SETD1A			Y													Y	Y	Y	Y	Y	Y	Y	Y	Y					Y															
15360	A_23_P21216	SETD2	Y																									Y		Y						Y										
18853	A_23_P44758	SETD2			Y															Y																										
283	A_23_P25984	SETD3																Y																												
18811	A_23_P212204	SETD5			Y	Y	Y	Y	Y	Y	Y	Y	Y	Y						Y		Y	Y				Y	Y	Y	Y						Y										
2915	A_23_P126398	SETDB1																			Y	Y																								
12263	A_23_P341532	SETDB2							Y	Y								Y	Y	Y		Y		Y						Y						Y										
12351	A_23_P80642	SETMAR			Y	Y		Y	Y	Y	Y	Y	Y	Y						Y		Y	Y				Y	Y	Y	Y						Y										
983	A_23_P43530	SETX				Y																																								
12867	A_23_P49849	SEZ6									Y		Y	Y													Y			Y						Y										
15007	A_23_P389569	SEZ6			Y																						Y																			
8879	A_23_P80242	SEZ6L	Y	Y	Y	Y		Y	Y	Y																	Y																			
3175	A_23_P318220	SF1			Y															Y							Y																			
8026	A_23_P104680	SF3A1			Y	Y		Y		Y								Y	Y	Y		Y	Y	Y			Y																			
14940	A_23_P211436	SF3A1							Y	Y																																				
6290	A_23_P90339	SF3A2			Y															Y	Y	Y	Y				Y																			
11242	A_23_P108441	SF3B1			Y	Y	Y	Y	Y	Y	Y	Y								Y			Y				Y	Y	Y	Y						Y										
4400	A_23_P165402	SF3B14																												Y						Y										
5334	A_23_P64082	SF3B2																		Y	Y		Y																							
908	A_23_P135914	SF3B3							Y	Y									Y				Y																							
2262	A_23_P35125	SF3B4																Y	Y	Y		Y		Y																						
3409	A_23_P19576	SF3B5			Y													Y		Y							Y																			
19569	A_23_P119618	SF4			Y	Y		Y		Y										Y		Y					Y			Y						Y										
18448	A_23_P80278	SFI1				Y																						Y																		
14099	A_23_P259393	SFMBT1			Y	Y		Y		Y										Y			Y				Y																			
5318	A_23_P63248	SFN																Y																												

15933	A_23_P126925	SFPQ			Y	Y		Y		Y																				Y																
15777	A_23_P10121	SFRP1			Y				Y	Y																		Y			Y						Y										
15774	A_23_P81103	SFRP2																		Y																										
1165	A_23_P215320	SFRP4							Y	Y									Y																											
16354	A_23_P49517	SFRS1				Y																								Y																
3063	A_23_P11664	SFRS11			Y	Y	Y	Y	Y	Y	Y	Y	Y	Y					Y	Y		Y	Y				Y	Y	Y	Y						Y								Y		
C10374.7	A_23_P18772	SFRS12			Y	Y	Y	Y	Y	Y	Y	Y	Y	Y				Y	Y	Y		Y	Y	Y			Y	Y	Y	Y						Y										
19566	A_23_P413184	SFRS12IP1				Y	Y				Y							Y	Y	Y		Y		Y																						
3714	A_23_P16562	SFRS14			Y	Y	Y	Y	Y	Y	Y	Y																Y		Y						Y										
2421	A_23_P78509	SFRS16			Y						Y		Y	Y																																
16796	A_23_P158201	SFRS17A			Y	Y	Y	Y	Y	Y	Y	Y							Y								Y																			
18190	A_23_P342668	SFRS17A																				Y																								
5957	A_23_P122617	SFRS18			Y	Y	Y	Y	Y	Y	Y	Y							Y	Y		Y					Y	Y	Y	Y																
8595	A_23_P363426	SFRS2B																					Y																							
13257	A_23_P87839	SFRS2IP				Y			Y	Y										Y								Y																		
4084	A_23_P413761	SFRS3																												Y						Y										
11051	A_23_P126197	SFRS4							Y	Y																				Y						Y										
7117	A_23_P205310	SFRS5			Y													Y		Y		Y					Y	Y	Y	Y																
13086	A_23_P377819	SFRS5			Y	Y	Y	Y		Y	Y	Y						Y		Y		Y	Y				Y	Y	Y	Y						Y										
16687	A_23_P109019	SFRS6																									Y																			
3609	A_23_P39704	SFRS7																												Y						Y										
4296	A_23_P25456	SFRS8																		Y			Y																							
4425	A_23_P148785	SFT2D2	Y	Y	Y	Y		Y	Y	Y																				Y						Y										
17066	A_23_P252511	SFTA2			Y	Y		Y	Y	Y									Y	Y			Y				Y			Y						Y										
9355	A_23_P115652	SFTPA1B			Y																																									
11372	A_23_P202092	SFTPA2B			Y																																									
7793	A_23_P346337	SFXN1																		Y							Y																			
20756	A_23_P387722	SFXN4				Y																					Y	Y	Y	Y																
17901	A_23_P66637	SGCA			Y	Y		Y		Y										Y								Y																		
17931	A_23_P251104	SGCB			Y				Y	Y	Y	Y	Y	Y																Y						Y										
11611	A_23_P136254	SGCD																Y	Y									Y																		
15679	A_23_P147647	SGCD																	Y																											
13360	A_23_P254626	SGCE				Y																																								
5860	A_23_P423237	SGCG																Y																												
17647	A_23_P69100	SGEF									Y		Y	Y																Y						Y										
18002	A_23_P62881	SGIP1				Y													Y			Y																								
12747	A_23_P83835	SGK196				Y																																								
9305	A_23_P131796	SGK2	Y	Y	Y	Y	Y	Y	Y	Y	Y	Y						Y									Y	Y	Y	Y																
1648	A_23_P356088	SGMS1							Y	Y																																				
1145	A_23_P326204	SGMS2			Y	Y		Y	Y	Y																				Y						Y										
22325	A_23_P75325	SGPL1			Y	Y		Y		Y																		Y		Y						Y										
6845	A_23_P347048	SGPP1			Y																																									
4609	A_23_P153970	SGPP2	Y		Y																						Y																			
733	A_23_P125135	SGSH																Y																												
3391	A_23_P254254	SGSH			Y															Y																										
3987	A_23_P118476	SGSM2																Y	Y	Y		Y		Y			Y																			
21698	A_23_P166491	SGSM3			Y	Y	Y	Y	Y	Y	Y	Y						Y	Y	Y	Y	Y	Y	Y	Y	Y	Y	Y	Y	Y	Y	Y	Y	Y	Y	Y										
10596	A_23_P108331	SGTA																Y		Y		Y																								
18206	A_23_P7379	SGTB			Y															Y	Y	Y	Y																							
12502	A_23_P13945	SH2B3																		Y																										
5680	A_23_P160618	SH2D2A			Y		Y				Y							Y	Y	Y		Y	Y	Y			Y																			
20734	A_23_P67213	SH2D3A			Y	Y		Y		Y																		Y																		
14525	A_23_P71928	SH2D3C			Y	Y	Y	Y	Y	Y	Y	Y						Y	Y	Y		Y	Y	Y			Y	Y	Y	Y	Y	Y	Y	Y	Y											
2556	A_23_P169003	SH2D4A							Y	Y																																				
6814	A_23_P148297	SH3BGRL			Y	Y	Y	Y	Y	Y	Y	Y	Y	Y				Y	Y	Y	Y	Y	Y	Y	Y	Y	Y	Y	Y	Y	Y	Y				Y										
6268	A_23_P111267	SH3BGRL2			Y				Y	Y																	Y			Y						Y										
17956	A_23_P126388	SH3BGRL3	Y		Y	Y	Y	Y	Y	Y	Y	Y	Y	Y				Y		Y	Y	Y	Y				Y	Y	Y	Y						Y										
7425	A_23_P143693	SH3BP1			Y	Y		Y		Y										Y			Y				Y																			
17107	A_23_P110116	SH3BP2			Y	Y	Y	Y	Y	Y	Y	Y	Y	Y				Y	Y	Y	Y	Y	Y	Y	Y	Y	Y	Y	Y	Y	Y	Y	Y	Y	Y	Y										
16958	A_23_P79259	SH3BP4			Y				Y	Y												Y	Y				Y	Y	Y	Y																
6817	A_23_P143973	SH3BP5																										Y																		
15608	A_23_P426989	SH3BP5L																	Y			Y																								
7030	A_23_P33364	SH3D19			Y	Y		Y		Y	Y	Y	Y	Y				Y	Y	Y				Y			Y	Y	Y	Y						Y										
11135	A_23_P208925	SH3GL1			Y	Y	Y	Y	Y	Y	Y	Y	Y	Y				Y	Y	Y		Y	Y	Y			Y	Y	Y	Y						Y										
12403	A_23_P48988	SH3GL3																		Y								Y																		
13679	A_23_P22957	SH3GLB1																												Y						Y										
12301	A_23_P216995	SH3GLB2																		Y																										
16783	A_23_P374777	SH3KBP1							Y	Y																				Y						Y										
5820	A_23_P35456	SH3PXD2A																		Y																										
9792	A_23_P345224	SH3PXD2A							Y	Y																				Y						Y										
19346	A_23_P363846	SH3PXD2B			Y																						Y																			
18996	A_23_P351184	SH3RF2																			Y		Y																							
10725	A_23_P41390	SH3TC1			Y	Y		Y		Y								Y	Y	Y	Y	Y	Y	Y	Y	Y	Y	Y	Y	Y	Y	Y				Y										
19372	A_23_P68198	SH3YL1																		Y	Y	Y	Y								Y		Y	Y	Y											
5338	A_23_P334883	SHANK2			Y	Y	Y	Y	Y	Y	Y	Y							Y	Y	Y		Y										Y													
17174	A_23_P29341	SHANK3			Y	Y		Y	Y	Y										Y		Y	Y				Y	Y	Y	Y						Y										
18762	A_23_P94942	SHARPIN			Y	Y	Y	Y	Y	Y	Y	Y	Y	Y				Y	Y	Y	Y	Y	Y	Y	Y	Y	Y			Y	Y	Y				Y										

13955	A_23_P257743	SHB																		Y																										
19162	A_23_P207088	SHBG				Y																																								
16863	A_23_P111657	SHH																Y	Y	Y		Y	Y	Y																						
8157	A_23_P115573	SHISA4	Y	Y	Y	Y		Y	Y	Y																	Y			Y						Y										
9419	A_23_P212475	SHISA5																												Y						Y										
8392	A_23_P130560	SHKBP1			Y														Y	Y		Y																								
5577	A_23_P502654	SHMT1																Y																												
4659	A_23_P158239	SHMT2			Y	Y	Y	Y	Y	Y	Y	Y						Y	Y	Y	Y	Y	Y	Y	Y	Y	Y	Y	Y	Y	Y	Y				Y										
21742	A_23_P169629	SHMT2			Y	Y	Y	Y	Y	Y	Y	Y	Y	Y					Y	Y			Y				Y	Y	Y	Y						Y										
10778	A_23_P202565	SHOC2				Y																						Y																		
1937	A_23_P310350	SHPK	Y	Y					Y	Y								Y																												
10830	A_23_P337790	SHPRH			Y	Y		Y		Y	Y	Y	Y	Y						Y			Y				Y	Y	Y	Y																
4049	A_23_P113441	SHQ1																		Y																										
4546	A_23_P156185	SHROOM1	Y	Y	Y	Y	Y	Y	Y	Y	Y	Y	Y	Y														Y		Y						Y										
10808	A_23_P217755	SHROOM2			Y	Y	Y	Y	Y	Y	Y	Y	Y	Y				Y		Y			Y				Y	Y	Y	Y						Y										
8234	A_23_P58120	SHROOM3	Y		Y				Y	Y										Y	Y	Y	Y				Y			Y			Y			Y										
3735	A_23_P24616	SIAE			Y	Y		Y		Y								Y		Y		Y	Y				Y																			
2814	A_23_P69121	SIAH2	Y																				Y							Y						Y										
13300	A_23_P132515	SIDT1			Y				Y	Y	Y	Y	Y	Y					Y	Y		Y	Y				Y			Y						Y										
14514	A_23_P84344	SIGIRR							Y	Y																				Y						Y										
12729	A_23_P17480	SIGLEC1	Y		Y										Y			Y		Y		Y	Y																							
20543	A_23_P208182	SIGLEC10			Y	Y		Y		Y					Y			Y	Y											Y						Y										
4043	A_23_P119322	SIGLEC11			Y	Y		Y		Y								Y	Y	Y			Y	Y																						
18192	A_23_P164603	SIGLEC12			Y				Y	Y																	Y	Y	Y	Y																
20426	A_23_P15995	SIGLEC5	Y			Y																					Y	Y	Y	Y																
16516	A_23_P50182	SIGLEC7	Y		Y	Y		Y	Y	Y																				Y						Y										
20148	A_23_P355957	SIGLEC8																												Y						Y										
14486	A_23_P4583	SIGLEC9	Y	Y	Y	Y		Y		Y					Y			Y		Y								Y		Y						Y										
11516	A_23_P146637	SIGMAR1			Y	Y		Y		Y								Y	Y	Y	Y	Y		Y																						
22203	A_23_P252903	SIL1																		Y	Y																									
8072	A_23_P2233	SILV																												Y						Y										
18867	A_23_P214743	SIM1				Y																																								
21985	A_23_P367676	SIN3A			Y																						Y																			
18174	A_23_P90004	SIN3B																	Y	Y	Y	Y																								
17092	A_23_P127460	SIPA1			Y	Y	Y	Y	Y	Y	Y	Y	Y					Y	Y	Y	Y	Y	Y	Y	Y	Y	Y	Y	Y	Y	Y	Y														
20644	A_23_P76969	SIPA1L1			Y		Y				Y							Y	Y	Y	Y	Y		Y			Y																			
12562	A_23_P137470	SIPA1L2			Y																						Y																			
2564	A_23_P210708	SIRPA	Y		Y	Y		Y	Y	Y	Y	Y	Y	Y	Y					Y			Y							Y						Y										
11371	A_23_P6056	SIRPB2			Y										Y												Y																			
20667	A_23_P28857	SIRPG	Y	Y	Y	Y	Y	Y	Y	Y	Y	Y															Y			Y						Y										
2088	A_23_P98022	SIRT1			Y	Y	Y	Y	Y	Y	Y	Y	Y	Y					Y	Y	Y	Y	Y				Y	Y	Y	Y						Y										
9273	A_23_P142455	SIRT2			Y	Y	Y	Y	Y	Y	Y	Y	Y	Y				Y	Y	Y	Y	Y	Y	Y	Y	Y	Y	Y	Y	Y	Y	Y														
17941	A_23_P99226	SIRT4																Y		Y			Y																							
12976	A_23_P214815	SIRT5				Y														Y								Y																		
3958	A_23_P208847	SIRT6			Y	Y	Y	Y	Y	Y	Y	Y	Y	Y				Y	Y	Y		Y	Y	Y			Y			Y						Y										
15711	A_23_P66815	SIRT7			Y	Y	Y	Y	Y	Y	Y	Y	Y	Y				Y	Y	Y		Y	Y	Y			Y	Y	Y	Y						Y										
17935	A_23_P43364	SIT1	Y																								Y																			
3311	A_23_P76914	SIX1																												Y						Y										
8080	A_23_P28120	SIX2			Y				Y	Y	Y	Y	Y	Y													Y			Y						Y										
8753	A_23_P100730	SKAP1			Y				Y	Y													Y				Y																			
20365	A_23_P325631	SKI									Y		Y	Y				Y		Y		Y	Y				Y	Y	Y	Y																
16964	A_23_P168078	SKIV2L																		Y		Y	Y																							
10864	A_23_P110661	SKIV2L2			Y	Y	Y	Y	Y	Y	Y	Y	Y	Y				Y	Y	Y		Y	Y	Y			Y	Y	Y	Y						Y										
4116	A_23_P133424	SKP1			Y	Y		Y	Y	Y	Y	Y	Y	Y						Y							Y	Y	Y	Y						Y										
19893	A_23_P156310	SKP2							Y	Y																				Y						Y										
14019	A_23_P216340	SLA	Y	Y	Y										Y					Y																										
17094	A_23_P143173	SLA2			Y														Y																											
11446	A_23_P348146	SLAIN1																Y																												
21197	A_23_P62647	SLAMF1	Y	Y	Y				Y	Y									Y								Y	Y	Y	Y																
4457	A_23_P200155	SLAMF6			Y	Y		Y	Y	Y																	Y	Y	Y	Y						Y										
9920	A_23_P11582	SLAMF7	Y	Y	Y		Y		Y	Y	Y	Y															Y																			
18973	A_23_P200138	SLAMF8	Y	Y	Y	Y	Y	Y		Y	Y	Y	Y	Y	Y												Y	Y	Y	Y						Y										
2220	A_23_P7101	SLBP																					Y																							
14769	A_23_P257316	SLC10A3			Y	Y	Y	Y	Y	Y	Y	Y	Y	Y				Y	Y	Y		Y	Y	Y			Y	Y	Y	Y	Y	Y	Y	Y	Y	Y										
18999	A_23_P366254	SLC10A3			Y	Y	Y	Y	Y	Y	Y	Y	Y	Y				Y	Y	Y		Y	Y	Y			Y	Y	Y	Y						Y										
16599	A_23_P376439	SLC10A4																									Y			Y						Y										
8187	A_23_P133606	SLC12A2																				Y																								
21657	A_23_P389391	SLC12A4							Y	Y										Y		Y	Y				Y																			
17086	A_23_P355376	SLC12A5																					Y																							
22192	A_23_P65883	SLC12A6							Y	Y																	Y																			
4765	A_23_P61688	SLC12A7			Y																																									
5362	A_23_P110105	SLC12A8			Y				Y	Y										Y							Y																			
14340	A_23_P215233	SLC12A9			Y	Y		Y	Y	Y	Y	Y	Y	Y				Y	Y	Y		Y	Y	Y			Y																			
19263	A_23_P107307	SLC13A2			Y	Y	Y	Y	Y	Y	Y	Y	Y	Y				Y	Y	Y		Y	Y	Y			Y	Y	Y	Y						Y										
12242	A_23_P19778	SLC13A4																					Y																							
309	A_23_P66737	SLC13A5	Y	Y	Y													Y									Y																			
22499	A_23_P302662	SLC13A5																					Y																							

18244	A_23_P55616	SLC14A1	Y	Y	Y													Y		Y										Y						Y										
2997	A_23_P128609	SLC15A1			Y	Y		Y	Y	Y	Y	Y	Y	Y					Y								Y	Y	Y	Y						Y										
7956	A_23_P326940	SLC15A3			Y				Y	Y	Y	Y	Y	Y						Y		Y					Y																			
19255	A_23_P98953	SLC15A4																					Y							Y						Y										
16767	A_23_P126825	SLC16A1							Y	Y																																				
17980	A_23_P70274	SLC16A10				Y			Y	Y																																				
9599	A_23_P363313	SLC16A11			Y																																									
19904	A_23_P152901	SLC16A11																		Y			Y																							
22107	A_23_P159125	SLC16A5			Y																						Y																			
7226	A_23_P152791	SLC16A6			Y	Y		Y	Y	Y	Y	Y	Y	Y													Y	Y	Y	Y						Y										
7711	A_23_P117052	SLC16A7																									Y																			
10044	A_23_P155079	SLC16A8																		Y										Y						Y										
9095	A_23_P115726	SLC16A9			Y	Y	Y	Y		Y	Y	Y							Y	Y	Y	Y					Y																			
14157	A_23_P59048	SLC17A2																		Y			Y																							
12040	A_23_P255695	SLC17A3			Y				Y	Y	Y	Y	Y	Y														Y		Y						Y										
4916	A_23_P357270	SLC17A4																										Y																		
18967	A_23_P81835	SLC17A4			Y																																									
1030	A_23_P110892	SLC17A5																		Y																										
15209	A_23_P78750	SLC17A7																		Y			Y																							
7436	A_23_P159076	SLC17A8			Y																																									
17957	A_23_P52362	SLC18A3																Y	Y			Y																								
16505	A_23_P29083	SLC19A1			Y	Y	Y	Y	Y	Y	Y	Y						Y		Y		Y	Y				Y	Y	Y	Y						Y										
18243	A_23_P160466	SLC19A2			Y	Y	Y	Y	Y	Y	Y	Y	Y	Y				Y	Y	Y		Y	Y	Y			Y	Y	Y	Y						Y										
18496	A_23_P216468	SLC1A1	Y	Y	Y	Y	Y	Y		Y	Y	Y	Y	Y				Y	Y	Y	Y	Y	Y	Y	Y	Y	Y	Y	Y	Y	Y	Y				Y										
12509	A_23_P162068	SLC1A2	Y	Y	Y	Y		Y	Y	Y	Y	Y	Y	Y													Y			Y						Y										
8850	A_23_P250963	SLC1A3			Y																																									
4420	A_23_P91151	SLC1A4							Y	Y																																				
4442	A_23_P55997	SLC1A5			Y		Y				Y		Y	Y				Y	Y	Y	Y	Y	Y	Y	Y	Y	Y			Y	Y	Y				Y		Y								
6036	A_23_P220	SLC1A7			Y				Y	Y										Y			Y				Y																			
12692	A_23_P325562	SLC1A7																					Y							Y						Y										
2609	A_23_P165657	SLC20A1			Y	Y	Y	Y	Y	Y	Y	Y							Y	Y							Y			Y						Y										
4100	A_23_P94921	SLC20A2			Y				Y	Y																																				
862	A_23_P145569	SLC22A1			Y		Y				Y																Y																			
9652	A_23_P161975	SLC22A10			Y																						Y																			
6665	A_23_P35809	SLC22A12				Y			Y	Y																		Y																		
3908	A_23_P144123	SLC22A13																				Y	Y																							
16509	A_23_P132730	SLC22A14				Y																																								
22455	A_23_P388900	SLC22A15			Y	Y	Y	Y		Y	Y	Y							Y	Y		Y					Y			Y						Y										
4382	A_23_P42004	SLC22A16																									Y			Y						Y										
262	A_23_P54029	SLC22A17	Y	Y	Y	Y	Y	Y	Y	Y	Y	Y	Y	Y													Y	Y	Y	Y																
1506	A_23_P139260	SLC22A18																												Y						Y										
9529	A_23_P7882	SLC22A23			Y				Y	Y	Y	Y	Y	Y								Y	Y				Y																			
6045	A_23_P64419	SLC22A25			Y													Y				Y																								
15537	A_23_P156180	SLC22A4	Y	Y	Y	Y		Y		Y								Y	Y	Y		Y	Y	Y			Y	Y	Y	Y						Y										
10398	A_23_P167566	SLC22A5							Y	Y	Y	Y	Y	Y																Y						Y										
10266	A_23_P98616	SLC22A6				Y																																								
17316	A_23_P150590	SLC22A9																												Y						Y										
14605	A_23_P21990	SLC23A1																	Y			Y	Y																							
748	A_23_P6087	SLC23A2			Y																						Y																			
13945	A_23_P28252	SLC23A3																		Y			Y																							
4160	A_23_P205910	SLC24A1	Y	Y	Y	Y	Y	Y	Y	Y	Y	Y	Y	Y						Y			Y				Y	Y	Y	Y						Y						Y				
259	A_23_P503010	SLC24A4			Y																																									
12357	A_23_P328600	SLC24A6			Y	Y	Y	Y	Y	Y	Y	Y						Y	Y	Y	Y	Y	Y	Y	Y	Y	Y				Y															
7939	A_23_P120773	SLC25A1			Y	Y	Y	Y	Y	Y	Y	Y	Y	Y				Y	Y	Y	Y	Y	Y	Y	Y	Y	Y			Y	Y	Y				Y		Y				Y			Y	Y
16989	A_23_P158207	SLC25A10			Y	Y	Y	Y		Y	Y	Y								Y			Y				Y			Y						Y										
6392	A_23_P38497	SLC25A11			Y	Y	Y	Y		Y	Y	Y								Y	Y	Y	Y																							
8706	A_23_P142714	SLC25A12			Y														Y	Y	Y	Y	Y								Y		Y	Y	Y											
7048	A_23_P135966	SLC25A14			Y				Y	Y	Y	Y	Y	Y					Y	Y		Y	Y				Y	Y	Y	Y																
8609	A_23_P128650	SLC25A15			Y													Y																												
18545	A_23_P251271	SLC25A16			Y	Y	Y	Y		Y	Y	Y						Y		Y		Y					Y	Y	Y	Y																
15812	A_23_P422783	SLC25A17																					Y																							
2481	A_23_P91576	SLC25A18			Y	Y		Y	Y	Y																	Y			Y						Y										
15479	A_23_P375404	SLC25A19	Y	Y	Y				Y	Y								Y		Y		Y					Y																			
685	A_23_P92650	SLC25A2			Y	Y		Y		Y																				Y						Y										
4833	A_23_P72025	SLC25A20																				Y																								
13047	A_23_P136504	SLC25A21			Y	Y		Y		Y																																				
8803	A_23_P146790	SLC25A22			Y	Y	Y	Y	Y	Y	Y	Y	Y	Y						Y			Y				Y																			
9968	A_23_P208450	SLC25A23	Y																																											
551	A_23_P86206	SLC25A24	Y		Y	Y	Y	Y		Y	Y	Y						Y	Y	Y	Y	Y	Y	Y	Y	Y	Y				Y		Y	Y	Y											
14706	A_23_P74799	SLC25A24			Y	Y		Y		Y									Y	Y		Y	Y				Y	Y	Y	Y																
2193	A_23_P9432	SLC25A25			Y													Y	Y	Y				Y			Y																			
15217	A_23_P169878	SLC25A26			Y	Y		Y	Y	Y	Y	Y	Y	Y																Y						Y								Y		
12282	A_23_P81721	SLC25A27	Y	Y	Y																						Y																			
12072	A_23_P75220	SLC25A28																		Y	Y		Y				Y																			
7543	A_23_P77049	SLC25A29							Y	Y																																				
10071	A_23_P25204	SLC25A3							Y	Y																																				
1350	A_23_P430064	SLC25A32				Y																																								

2164	A_23_P36226	SLC25A33			Y																						Y	Y	Y	Y																
17700	A_23_P77939	SLC25A35			Y															Y			Y							Y						Y										
8110	A_23_P40896	SLC25A36							Y	Y																																				
3140	A_23_P216004	SLC25A37							Y	Y																				Y						Y										
18655	A_23_P250833	SLC25A37							Y	Y																				Y						Y										
7952	A_23_P100762	SLC25A39			Y													Y	Y	Y	Y	Y		Y																						
10082	A_23_P432626	SLC25A40																					Y																							
20721	A_23_P125668	SLC25A43			Y																																									
4932	A_23_P12034	SLC25A44			Y	Y		Y	Y	Y																	Y	Y	Y	Y						Y										
5221	A_23_P133375	SLC25A46																		Y								Y																		
7512	A_23_P137057	SLC25A5	Y	Y	Y	Y		Y	Y	Y								Y									Y																			
8327	A_23_P436179	SLC25A5	Y	Y	Y	Y		Y	Y	Y																																				
6153	A_23_P33752	SLC25A6			Y	Y	Y	Y	Y	Y	Y	Y							Y	Y	Y	Y	Y				Y																			
2287	A_23_P80953	SLC26A1																			Y																									
17381	A_23_P203807	SLC26A10			Y				Y	Y																	Y																			
21104	A_23_P125078	SLC26A11			Y																						Y																			
21878	A_23_P250951	SLC26A2							Y	Y	Y	Y	Y	Y													Y			Y						Y										
22528	A_23_P123228	SLC26A3																	Y	Y	Y																									
17719	A_23_P157391	SLC26A4			Y																						Y			Y						Y										
20128	A_23_P331560	SLC26A4																		Y		Y																								
15439	A_23_P8613	SLC26A5																			Y																									
17423	A_23_P257771	SLC26A6																		Y			Y							Y						Y										
16014	A_23_P131111	SLC27A1																										Y		Y						Y										
13036	A_23_P140450	SLC27A2	Y	Y	Y	Y		Y	Y	Y	Y	Y	Y	Y				Y	Y	Y		Y	Y	Y			Y	Y	Y	Y						Y										
9208	A_23_P149535	SLC27A3																					Y																							
2238	A_23_P259714	SLC27A4			Y	Y	Y	Y	Y	Y	Y	Y	Y	Y				Y	Y	Y		Y	Y	Y			Y			Y						Y										
17643	A_23_P41789	SLC27A6																	Y																											
12251	A_23_P98294	SLC29A2			Y	Y		Y	Y	Y																	Y	Y	Y	Y						Y										
10645	A_23_P357360	SLC29A3			Y				Y	Y																	Y																			
9195	A_23_P168554	SLC29A4			Y																																									
541	A_23_P211384	SLC2A11				Y																						Y																		
21397	A_23_P394996	SLC2A12							Y	Y																																				
3858	A_23_P113835	SLC2A13																		Y		Y					Y																			
7078	A_23_P10211	SLC2A13																		Y			Y																							
364	A_23_P139669	SLC2A3			Y	Y		Y	Y	Y																																				
6895	A_23_P102571	SLC2A4RG							Y	Y																				Y						Y										
19022	A_23_P160159	SLC2A5	Y	Y	Y	Y		Y	Y	Y																	Y	Y	Y	Y						Y										
15846	A_23_P169249	SLC2A6			Y	Y		Y		Y										Y		Y	Y				Y	Y	Y	Y																
6598	A_23_P148861	SLC2A7				Y																Y						Y																		
6258	A_23_P252783	SLC2A8			Y				Y	Y																				Y						Y										
2517	A_23_P213014	SLC2A9																				Y																								
4844	A_23_P23815	SLC30A1			Y																																									
9840	A_23_P86242	SLC30A10			Y										Y			Y		Y							Y																			
5764	A_23_P319083	SLC30A2																			Y		Y																							
15461	A_23_P120311	SLC30A3			Y	Y		Y	Y	Y										Y								Y		Y						Y										
7174	A_23_P117679	SLC30A4			Y	Y		Y	Y	Y										Y		Y								Y						Y										
2427	A_23_P218988	SLC30A5				Y			Y	Y	Y	Y	Y	Y				Y		Y		Y	Y				Y	Y	Y	Y						Y										
19506	A_23_P165394	SLC30A6			Y																																									
5455	A_23_P22915	SLC30A7			Y				Y	Y																	Y			Y						Y										
7322	A_23_P169074	SLC30A8			Y	Y		Y		Y								Y									Y																			
22111	A_23_P362798	SLC30A8			Y																																									
18011	A_23_P30044	SLC30A9																		Y																										
5140	A_23_P253409	SLC31A1			Y	Y	Y	Y	Y	Y	Y	Y															Y	Y	Y	Y																
6368	A_23_P217109	SLC31A2			Y																																									
5288	A_23_P58729	SLC34A1			Y				Y	Y																	Y			Y						Y										
5368	A_23_P133036	SLC34A2							Y	Y																																				
18619	A_23_P121646	SLC34A2			Y				Y	Y									Y	Y			Y				Y			Y						Y										
7757	A_23_P113682	SLC34A3				Y														Y			Y																							
7764	A_23_P58907	SLC35A1			Y	Y		Y		Y								Y		Y	Y	Y	Y				Y	Y	Y	Y																
18411	A_23_P217208	SLC35A2			Y	Y	Y	Y	Y	Y	Y	Y															Y	Y	Y	Y																
19778	A_23_P34460	SLC35A3																										Y																		
6934	A_23_P69844	SLC35A4			Y				Y	Y								Y	Y	Y		Y		Y																						
20996	A_23_P89455	SLC35B1	Y	Y	Y	Y		Y	Y	Y										Y							Y	Y	Y	Y						Y										
8888	A_23_P19257	SLC35B2			Y													Y	Y	Y		Y	Y	Y			Y																			
5624	A_23_P202720	SLC35C1			Y															Y			Y				Y																			
16624	A_23_P68452	SLC35C2			Y	Y	Y	Y	Y	Y	Y	Y						Y	Y	Y	Y	Y	Y	Y	Y	Y	Y			Y	Y	Y	Y	Y	Y	Y				Y						
4534	A_23_P357929	SLC35D1				Y																																								
4761	A_23_P32025	SLC35D2			Y																																									
22322	A_23_P208416	SLC35E1			Y	Y	Y	Y		Y	Y	Y						Y	Y	Y		Y		Y			Y																			
18063	A_23_P259357	SLC35E4			Y	Y		Y	Y	Y	Y	Y	Y	Y													Y	Y	Y	Y						Y										
7947	A_23_P339098	SLC35F2							Y	Y																				Y						Y										
7258	A_23_P361569	SLC35F5			Y															Y																										
14378	A_23_P167640	SLC36A1																									Y																			
9335	A_23_P70102	SLC36A2																				Y																								
6181	A_23_P17695	SLC37A1			Y		Y		Y	Y	Y	Y						Y	Y	Y		Y	Y	Y			Y			Y						Y										
20254	A_23_P86931	SLC37A2																												Y						Y										
22068	A_23_P95133	SLC37A3							Y	Y										Y	Y	Y																								

21310	A_23_P35970	SLC37A4																Y	Y	Y			Y	Y																				
20984	A_23_P99231	SLC38A1							Y	Y																																		
6374	A_23_P56488	SLC38A11				Y			Y	Y																			Y	Y	Y	Y																
9772	A_23_P218079	SLC38A2																		Y																								
10157	A_23_P155493	SLC38A3																	Y	Y																								
21643	A_23_P25487	SLC38A4									Y		Y	Y									Y				Y	Y	Y	Y						Y										
190	A_23_P84929	SLC38A5																		Y		Y	Y																					
20727	A_23_P88309	SLC38A6	Y																																									
9866	A_23_P65967	SLC38A7			Y	Y		Y	Y	Y	Y	Y	Y	Y						Y			Y				Y			Y						Y										
20339	A_23_P26348	SLC38A8			Y				Y	Y																	Y																			
20142	A_23_P334192	SLC38A9																		Y		Y	Y																					
3842	A_23_P23575	SLC39A1			Y	Y		Y	Y	Y	Y	Y	Y	Y				Y	Y	Y	Y	Y	Y	Y	Y	Y	Y	Y	Y	Y	Y	Y				Y										
17317	A_23_P119778	SLC39A10																		Y			Y																					
4145	A_23_P158969	SLC39A11																Y		Y																								
17439	A_23_P24149	SLC39A12																												Y						Y										
4041	A_23_P59950	SLC39A14			Y	Y		Y	Y	Y																	Y																			
1777	A_23_P388170	SLC39A3			Y		Y				Y									Y							Y																			
12962	A_23_P20502	SLC39A4			Y	Y	Y	Y		Y	Y	Y						Y	Y								Y																			
22525	A_23_P50167	SLC39A6	Y	Y	Y	Y	Y	Y	Y	Y	Y	Y	Y	Y					Y	Y			Y				Y	Y	Y	Y						Y								Y		
17720	A_23_P70571	SLC39A7			Y	Y		Y		Y												Y																								
4098	A_23_P205301	SLC39A9	Y	Y	Y	Y	Y	Y	Y	Y	Y	Y	Y	Y				Y		Y	Y	Y	Y				Y	Y	Y	Y			Y			Y										
2397	A_23_P75808	SLC3A2			Y	Y	Y	Y	Y	Y	Y	Y								Y	Y	Y	Y				Y																			
16209	A_23_P102391	SLC40A1							Y	Y	Y	Y	Y	Y																Y						Y										
2161	A_23_P368182	SLC41A1																									Y																			
1323	A_23_P204808	SLC41A2			Y	Y		Y	Y	Y																	Y																			
4936	A_23_P152640	SLC43A2			Y	Y		Y		Y										Y		Y	Y				Y	Y	Y	Y																
18498	A_23_P358545	SLC43A3							Y	Y	Y	Y	Y	Y									Y							Y						Y										
8249	A_23_P216630	SLC44A1			Y	Y	Y	Y	Y	Y	Y	Y	Y	Y					Y	Y	Y	Y	Y				Y	Y	Y	Y						Y										
15740	A_23_P208340	SLC44A2			Y	Y	Y	Y	Y	Y	Y	Y						Y	Y	Y		Y	Y	Y			Y	Y	Y	Y						Y										
10116	A_23_P86196	SLC44A3																Y		Y	Y	Y									Y		Y	Y	Y											
14288	A_23_P93352	SLC44A4			Y	Y		Y	Y	Y	Y	Y	Y	Y																Y						Y										
2353	A_23_P259049	SLC45A2																			Y																							
10060	A_23_P359766	SLC46A1			Y																						Y																			
8236	A_23_P207221	SLC47A1			Y													Y	Y								Y																			
20617	A_23_P77913	SLC47A2				Y			Y	Y																																		
9143	A_23_P77980	SLC4A1			Y				Y	Y									Y	Y			Y				Y			Y						Y										
13702	A_23_P154688	SLC4A11			Y	Y		Y	Y	Y																	Y			Y						Y										
1154	A_23_P56810	SLC4A1AP							Y	Y																																		
12686	A_23_P253382	SLC4A2			Y		Y				Y							Y	Y	Y	Y	Y	Y	Y	Y	Y	Y				Y															
14235	A_23_P132468	SLC4A7																												Y						Y										
10204	A_23_P72908	SLC4A8			Y	Y		Y	Y	Y	Y	Y	Y	Y													Y	Y	Y	Y						Y										
19348	A_23_P257176	SLC4A9				Y			Y	Y																				Y						Y										
19173	A_23_P17826	SLC5A1																									Y			Y						Y										
8670	A_23_P328022	SLC5A10			Y																						Y			Y						Y										
6725	A_23_P411277	SLC5A2			Y	Y		Y	Y	Y								Y	Y	Y	Y	Y	Y	Y	Y	Y	Y			Y	Y	Y				Y										
16707	A_23_P109308	SLC5A3			Y																									Y						Y										
10835	A_23_P101774	SLC5A5							Y	Y																																		
3756	A_23_P102508	SLC5A6			Y													Y	Y	Y		Y	Y	Y			Y																			
13511	A_23_P67871	SLC5A7																		Y																								
6946	A_23_P338542	SLC5A8			Y				Y	Y													Y				Y			Y						Y										
18518	A_23_P151135	SLC5A8			Y				Y	Y	Y	Y	Y	Y														Y		Y						Y										
16073	A_23_P76386	SLC6A12							Y	Y																																		
18719	A_23_P13725	SLC6A15																		Y																								
8480	A_23_P673	SLC6A17			Y	Y		Y	Y	Y										Y			Y				Y			Y						Y										
13261	A_23_P122246	SLC6A18																		Y																								
11965	A_23_P62070	SLC6A19							Y	Y																	Y																			
14481	A_23_P213460	SLC6A3																												Y						Y										
2227	A_23_P152995	SLC6A4			Y	Y		Y	Y	Y													Y				Y	Y	Y	Y						Y										
11059	A_23_P116624	SLC6A5			Y				Y	Y																				Y						Y										
22465	A_23_P69206	SLC6A6																												Y						Y										
4381	A_23_P343766	SLC6A7																					Y																					
18048	A_23_P70188	SLC6A7			Y													Y									Y																			
19206	A_23_P159937	SLC6A8				Y			Y	Y										Y		Y																								
7337	A_23_P11984	SLC6A9				Y			Y	Y																																		
1468	A_23_P45199	SLC7A11																									Y																			
8747	A_23_P336663	SLC7A13			Y				Y	Y	Y	Y	Y	Y													Y																			
5562	A_23_P253451	SLC7A14																									Y																			
10040	A_23_P120798	SLC7A4			Y	Y		Y		Y			Y																															
11021	A_23_P99642	SLC7A7	Y	Y	Y		Y				Y				Y												Y																			
22523	A_23_P205480	SLC7A8	Y	Y	Y	Y		Y	Y	Y	Y	Y	Y	Y																Y						Y										
21866	A_23_P142250	SLC7A9																									Y																			
15527	A_23_P119957	SLC8A1																Y	Y	Y		Y		Y				Y																		
22126	A_23_P23598	SLC9A1				Y																								Y						Y										
4890	A_23_P5536	SLC9A2			Y																						Y																			
6132	A_23_P308519	SLC9A3R1				Y			Y	Y																				Y						Y										
15223	A_23_P152593	SLC9A3R1				Y			Y	Y									Y											Y						Y										
21832	A_23_P16941	SLC9A4																				Y																								

21552	A_23_P22625	SLC9A6	Y	Y	Y				Y	Y	Y	Y	Y	Y									Y				Y			Y						Y										
7827	A_23_P11032	SLC9A7				Y																																									
11968	A_23_P252936	SLC9A8			Y	Y		Y		Y												Y																									
22533	A_23_P212482	SLC9A9			Y				Y	Y								Y					Y							Y						Y										
14117	A_23_P116867	SLCO1C1																	Y	Y	Y		Y		Y																						
4176	A_23_P135990	SLCO2A1			Y																		Y																						
19012	A_23_P150768	SLCO2B1	Y		Y										Y								Y																						
3200	A_23_P5901	SLCO4A1			Y	Y		Y	Y	Y	Y	Y	Y	Y														Y		Y						Y										
12669	A_23_P129925	SLFN11																													Y						Y										
18578	A_23_P402899	SLFN5			Y																						Y																			
16297	A_23_P379636	SLFNL1			Y	Y	Y	Y		Y	Y	Y							Y	Y		Y	Y				Y			Y						Y										
14287	A_23_P144348	SLIT2	Y	Y		Y																						Y		Y						Y										
4292	A_23_P58588	SLIT3																					Y																						
15072	A_23_P37041	SLITRK1							Y	Y																																					
18057	A_23_P217276	SLITRK2											Y							Y																									
18102	A_23_P211673	SLITRK2			Y	Y		Y	Y	Y																		Y		Y						Y										
6782	A_23_P18363	SLITRK3				Y																																									
9448	A_23_P373604	SLITRK4																	Y																										
13060	A_23_P65307	SLITRK6	Y	Y	Y	Y		Y		Y																	Y	Y	Y	Y																	
6079	A_23_P202199	SLK				Y														Y								Y																		
9766	A_23_P29441	SLMAP																	Y	Y			Y																						
21465	A_23_P38677	SLMO1							Y	Y	Y	Y	Y	Y				Y		Y										Y						Y										
16771	A_23_P210658	SLMO2							Y	Y											Y									Y						Y										
2931	A_23_P150343	SLN																Y	Y										Y						Y										
4178	A_23_P91230	SLPI			Y	Y		Y	Y	Y																	Y	Y	Y	Y						Y										
4704	A_23_P26094	SLTM			Y	Y	Y	Y	Y	Y	Y	Y	Y	Y					Y	Y		Y					Y	Y	Y	Y						Y						Y				
17111	A_23_P110702	SLU7			Y	Y	Y	Y	Y	Y	Y	Y							Y	Y							Y																			
22400	A_23_P331779	SLU7			Y	Y		Y		Y																	Y																			
16068	A_23_P400288	SLURP1			Y	Y		Y		Y									Y									Y																			
12628	A_23_P121869	SMA4	Y	Y	Y	Y		Y	Y	Y	Y	Y	Y	Y				Y		Y								Y			Y						Y										
5775	A_23_P255992	SMAD5OS																			Y																								
11782	A_23_P399797	SMAD5OS																		Y																									
14883	A_23_P44244	SMARCA1			Y	Y	Y	Y		Y	Y	Y							Y	Y								Y			Y						Y								Y	
821	A_23_P252765	SMARCA2	Y	Y	Y	Y		Y		Y						Y			Y	Y	Y		Y		Y			Y	Y	Y	Y																	
15645	A_23_P39034	SMARCA4			Y																																								
5922	A_23_P256716	SMARCA5				Y														Y	Y			Y				Y																			
C10443.5	A_23_P213255	SMARCAD1			Y	Y	Y	Y		Y	Y	Y							Y	Y		Y					Y	Y	Y	Y						Y										
21768	A_23_P68820	SMARCB1			Y	Y	Y	Y	Y	Y	Y	Y	Y	Y				Y	Y	Y	Y	Y	Y	Y	Y	Y	Y	Y	Y	Y	Y	Y	Y	Y	Y	Y				Y							
3378	A_23_P123952	SMARCC1			Y														Y	Y		Y					Y																			
485	A_23_P128073	SMARCC2	Y	Y	Y	Y		Y	Y	Y	Y	Y	Y	Y													Y	Y	Y	Y						Y										
6494	A_23_P66487	SMARCD2							Y	Y								Y	Y	Y		Y	Y	Y			Y			Y						Y										
12039	A_23_P122852	SMARCD3				Y			Y	Y																				Y						Y										
1718	A_23_P333060	SMARCE1			Y	Y		Y		Y																	Y																			
19521	A_23_P164387	SMARCE1			Y	Y	Y	Y	Y	Y	Y	Y	Y	Y					Y	Y							Y			Y						Y										
15115	A_23_P217411	SMC1A			Y	Y		Y		Y																																				
18686	A_23_P29325	SMC1B																									Y																			
18133	A_23_P138631	SMC3			Y	Y		Y	Y	Y																			Y																		
7351	A_23_P91902	SMC4			Y	Y		Y		Y																			Y																		
10449	A_23_P325716	SMC5																		Y		Y	Y																						
2281	A_23_P38723	SMCHD1																		Y																									
1217	A_23_P388798	SMCR5			Y	Y		Y		Y	Y	Y	Y	Y													Y	Y	Y	Y						Y										
19586	A_23_P501372	SMCR7	Y	Y																																										
4824	A_23_P103135	SMCR7L				Y			Y	Y			Y						Y	Y		Y																								
7987	A_23_P323057	SMCR7L			Y														Y	Y		Y	Y				Y																			
17046	A_23_P300124	SMCR8			Y	Y		Y	Y	Y											Y	Y		Y				Y																			
1115	A_23_P5863	SMEK2																		Y																									
16303	A_23_P26358	SMG1																													Y						Y										
19198	A_23_P322043	SMG5			Y	Y		Y		Y								Y	Y	Y		Y		Y			Y	Y	Y	Y																	
11518	A_23_P100677	SMG6			Y																Y			Y																						
14870	A_23_P256270	SMN2			Y	Y	Y	Y		Y	Y	Y							Y									Y																		
4200	A_23_P70818	SMO							Y	Y	Y	Y	Y	Y																	Y						Y										
6927	A_23_P102731	SMOX				Y			Y	Y	Y	Y	Y	Y														Y		Y						Y										
695	A_23_P163567	SMPD3																Y	Y																											
826	A_23_P152186	SMPD3				Y			Y	Y								Y	Y	Y					Y																					
11915	A_23_P347027	SMPD3							Y	Y																																					
16165	A_23_P72117	SMPDL3A				Y			Y	Y								Y	Y																											
8722	A_23_P160881	SMPDL3B																Y	Y																											
15927	A_23_P41365	SMR3A			Y																										Y						Y										
18454	A_23_P29965	SMR3B				Y																																									
3611	A_23_P251662	SMS				Y																						Y																		
10455	A_23_P211428	SMTN			Y	Y	Y	Y	Y	Y	Y	Y	Y	Y				Y	Y	Y	Y	Y	Y	Y	Y	Y	Y	Y	Y	Y	Y	Y	Y			Y				Y							
17402	A_23_P150502	SMTNL1			Y	Y		Y		Y										Y			Y				Y			Y						Y										
8329	A_23_P68165	SMYD1																Y				Y																							
5575	A_23_P91015	SMYD5			Y	Y	Y	Y	Y	Y	Y	Y						Y	Y	Y		Y		Y			Y	Y	Y	Y						Y										
19868	A_23_P29141	SNAP29			Y								Y																																	
3847	A_23_P37251	SNAPC1																					Y																						
13382	A_23_P67228	SNAPC2	Y															Y																												

19432	A_23_P71908	SNAPC3							Y	Y													Y																							
15467	A_23_P20728	SNAPC4			Y	Y		Y		Y								Y	Y	Y	Y	Y	Y	Y	Y	Y	Y				Y															
177	A_23_P29939	SNCA																												Y						Y										
9671	A_23_P19020	SNCAIP																Y																												
13373	A_23_P42829	SND1			Y																						Y																			
19420	A_23_P130064	SNF8																Y		Y		Y	Y																							
19780	A_23_P23173	SNIP1			Y																																									
15028	A_23_P152160	SNN	Y	Y	Y	Y		Y	Y	Y	Y	Y	Y	Y				Y	Y	Y			Y	Y			Y	Y	Y	Y						Y										
2942	A_23_P102706	SNPH							Y	Y																																				
2452	A_23_P211985	SNRK			Y	Y		Y		Y																																				
10648	A_23_P131626	SNRNP200				Y														Y		Y																								
21824	A_23_P152284	SNRNP25																Y																												
22263	A_23_P39775	SNRNP27	Y	Y	Y						Y		Y	Y													Y																			
3347	A_23_P306234	SNRNP48			Y	Y		Y		Y																																				
1762	A_23_P4909	SNRNP70			Y	Y		Y		Y								Y	Y	Y	Y	Y	Y	Y	Y	Y	Y				Y															
4090	A_23_P164718	SNRPA			Y													Y	Y	Y		Y	Y	Y			Y			Y						Y										
782	A_23_P154675	SNRPB			Y								Y					Y	Y	Y	Y	Y		Y			Y																			
4272	A_23_P59192	SNRPC							Y	Y								Y												Y						Y										
13487	A_23_P16346	SNRPD2																										Y																		
16558	A_23_P117068	SNRPF			Y																																									
8598	A_23_P102231	SNRPG	Y	Y	Y	Y		Y	Y	Y	Y	Y	Y	Y													Y	Y	Y	Y						Y										
17045	A_23_P57227	SNTA1			Y	Y		Y		Y								Y																												
8440	A_23_P95029	SNTB1				Y			Y	Y																				Y						Y										
9546	A_23_P33443	SNTB2																Y		Y	Y	Y																								
12454	A_23_P251132	SNTG2							Y	Y																				Y						Y										
4397	A_23_P256804	SNUPN			Y																						Y																			
18836	A_23_P95189	SNUPN																									Y																			
7371	A_23_P37345	SNW1			Y	Y	Y	Y	Y	Y	Y	Y	Y	Y					Y	Y		Y	Y				Y	Y	Y	Y						Y										
9775	A_23_P49033	SNX1																			Y																									
8156	A_23_P89343	SNX11																Y	Y	Y				Y																						
7013	A_23_P10699	SNX12			Y	Y		Y		Y								Y	Y	Y		Y		Y																						
12989	A_23_P368896	SNX12			Y	Y		Y		Y																																				
3693	A_23_P256342	SNX13							Y	Y										Y			Y																							
4395	A_23_P412980	SNX13							Y	Y										Y			Y																							
11365	A_23_P259054	SNX14			Y				Y	Y	Y	Y	Y	Y						Y			Y																							
7075	A_23_P258891	SNX16																		Y																										
18738	A_23_P28233	SNX17			Y	Y	Y	Y		Y	Y	Y							Y	Y	Y	Y	Y				Y	Y	Y	Y																
20205	A_23_P98345	SNX19																					Y																							
8179	A_23_P7697	SNX2																			Y																									
21293	A_23_P54496	SNX22				Y																	Y				Y			Y						Y										
14607	A_23_P19095	SNX24																	Y																											
2791	A_23_P18641	SNX25			Y	Y		Y	Y	Y									Y	Y								Y		Y						Y										
855	A_23_P5189	SNX26				Y												Y		Y	Y	Y	Y				Y																			
2840	A_23_P201238	SNX27				Y																																								
1605	A_23_P42498	SNX3	Y		Y	Y		Y		Y																																				
13217	A_23_P254404	SNX33																		Y	Y	Y																								
1898	A_23_P72568	SNX4			Y	Y		Y		Y										Y			Y																							
20607	A_23_P35066	SNX7							Y	Y																																				
18665	A_23_P414252	SNX8	Y		Y	Y	Y	Y	Y	Y	Y	Y	Y	Y						Y	Y	Y	Y				Y	Y	Y	Y						Y										
11685	A_23_P214789	SNX9			Y	Y	Y	Y	Y	Y	Y	Y							Y	Y	Y		Y				Y	Y	Y	Y						Y										
15604	A_23_P25475	SOAT2			Y	Y		Y		Y																																				
18881	A_23_P156907	SOBP			Y				Y	Y																	Y			Y						Y										
10184	A_23_P420196	SOCS1			Y	Y	Y	Y	Y	Y	Y	Y	Y	Y				Y		Y		Y					Y	Y	Y	Y																
14859	A_23_P128215	SOCS2				Y														Y																										
21263	A_23_P207058	SOCS3							Y	Y																	Y																			
11659	A_23_P5813	SOCS5																												Y						Y										
14293	A_23_P207981	SOCS6			Y	Y		Y	Y	Y								Y	Y	Y	Y	Y	Y	Y	Y	Y	Y	Y	Y	Y	Y	Y														
5710	A_23_P134176	SOD2	Y	Y	Y	Y		Y	Y	Y	Y	Y	Y	Y														Y		Y						Y										
8603	A_23_P254741	SOD3			Y	Y	Y	Y	Y	Y	Y	Y						Y	Y	Y	Y	Y	Y	Y	Y	Y	Y				Y		Y	Y												
10160	A_23_P25619	SOHLH2			Y	Y		Y		Y									Y	Y		Y					Y																			
21762	A_23_P211099	SON			Y	Y	Y	Y	Y	Y	Y	Y								Y		Y					Y	Y	Y	Y						Y										
8581	A_23_P202388	SORBS1							Y	Y																				Y						Y										
20527	A_23_P257562	SORBS2																		Y			Y																							
9983	A_23_P300710	SORBS3																					Y																							
15841	A_23_P352932	SORCS1			Y	Y		Y		Y										Y																										
10079	A_23_P121665	SORCS2																										Y																		
2816	A_23_P77103	SORD							Y	Y																	Y			Y						Y										
20133	A_23_P87049	SORL1			Y	Y		Y	Y	Y	Y	Y	Y	Y				Y	Y	Y		Y		Y			Y	Y	Y	Y						Y										
3729	A_23_P209607	SOS1																		Y		Y																								
17571	A_23_P343808	SOS1																									Y																			
6705	A_23_P117542	SOS2			Y	Y		Y		Y	Y	Y	Y	Y				Y	Y	Y	Y	Y		Y			Y	Y	Y	Y						Y										
12240	A_23_P118571	SOST			Y																																									
15801	A_23_P145841	SOSTDC1			Y	Y		Y	Y	Y	Y	Y	Y	Y					Y								Y			Y						Y								Y		
21891	A_23_P209712	SP100				Y														Y		Y					Y																			
10137	A_23_P209728	SP140																									Y																			
12840	A_23_P337753	SP140L	Y		Y	Y		Y	Y	Y										Y							Y			Y						Y										
9223	A_23_P104876	SPA17			Y	Y		Y		Y										Y		Y	Y				Y																			

21030	A_23_P30649	SPACA1							Y	Y																				Y						Y										
13617	A_23_P317244	SPACA3																				Y																								
17922	A_23_P38434	SPACA3			Y																						Y																			
10504	A_23_P256844	SPACA5							Y	Y																																				
16360	A_23_P34719	SPAG17			Y	Y		Y		Y									Y								Y																			
11921	A_23_P132027	SPAG4			Y	Y	Y	Y	Y	Y	Y	Y	Y	Y													Y	Y	Y	Y						Y									Y		
6657	A_23_P75147	SPAG6			Y	Y	Y	Y		Y	Y	Y																Y																		
22504	A_23_P218412	SPAG7				Y																																								
14388	A_23_P216490	SPAG8			Y	Y	Y	Y	Y	Y	Y	Y	Y	Y				Y	Y	Y				Y			Y	Y	Y	Y						Y										
13245	A_23_P356039	SPAG9							Y	Y																																				
1302	A_23_P96300	SPANXA1			Y																						Y																			
13252	A_23_P136883	SPANXB2	Y	Y	Y	Y		Y		Y								Y		Y			Y				Y	Y	Y	Y																
20659	A_23_P257882	SPANXC			Y	Y		Y		Y			Y														Y																			
1315	A_23_P113351	SPARCL1			Y																						Y																			
20581	A_23_P103897	SPATA1																												Y						Y										
17914	A_23_P346912	SPATA17			Y				Y	Y	Y	Y	Y	Y						Y			Y				Y																			
18980	A_23_P407112	SPATA18	Y		Y	Y	Y	Y	Y	Y	Y	Y								Y		Y					Y	Y	Y	Y						Y										
8150	A_23_P210554	SPATA2			Y															Y			Y				Y																			
21955	A_23_P329953	SPATA3			Y																																									
17213	A_23_P393025	SPATA4	Y	Y	Y	Y		Y	Y	Y													Y				Y	Y	Y	Y						Y										
20137	A_23_P37424	SPATA5L1			Y															Y			Y																							
14043	A_23_P327483	SPATA6				Y			Y	Y								Y				Y	Y					Y																		
13430	A_23_P48755	SPATA7			Y	Y	Y	Y	Y	Y	Y	Y	Y	Y				Y	Y	Y		Y	Y	Y			Y	Y	Y	Y						Y										
9033	A_23_P390576	SPATA9																												Y						Y										
20998	A_23_P51085	SPC25							Y	Y																																				
19412	A_23_P29630	SPCS1							Y	Y										Y																										
2526	A_23_P2143	SPCS2																		Y			Y																							
1800	A_23_P132936	SPCS3	Y	Y	Y	Y	Y	Y	Y	Y	Y	Y	Y	Y					Y	Y							Y	Y	Y	Y						Y										
3752	A_23_P40280	SPEF1																		Y			Y																							
13577	A_23_P382081	SPEF2	Y	Y	Y	Y	Y	Y	Y	Y	Y	Y	Y	Y				Y	Y	Y	Y	Y	Y	Y	Y	Y	Y	Y	Y	Y	Y	Y	Y	Y	Y	Y										
10481	A_23_P338919	SPEG							Y	Y																																				
19052	A_23_P209501	SPEG			Y	Y	Y	Y	Y	Y	Y	Y							Y	Y			Y				Y	Y	Y	Y						Y										
18989	A_23_P12054	SPEN							Y	Y																																				
16319	A_23_P338469	SPERT			Y															Y		Y	Y				Y																			
12115	A_23_P129089	SPESP1																												Y						Y										
2114	A_23_P65699	SPG11			Y	Y		Y	Y	Y	Y	Y	Y	Y						Y	Y	Y	Y				Y	Y	Y	Y						Y										
16125	A_23_P392541	SPG20							Y	Y								Y	Y	Y		Y	Y	Y						Y						Y										
20584	A_23_P147450	SPG21			Y	Y		Y		Y										Y							Y	Y	Y	Y																
5956	A_23_P217938	SPHAR			Y	Y		Y		Y																	Y																			
7544	A_23_P38106	SPHK1				Y			Y	Y	Y	Y	Y	Y				Y		Y	Y	Y	Y				Y			Y			Y			Y										
4388	A_23_P303181	SPHK2									Y		Y	Y																Y						Y										
3271	A_23_P136952	SPIN2A																					Y																							
2269	A_23_P125553	SPIN2B			Y	Y		Y	Y	Y										Y			Y																							
11708	A_23_P214079	SPINK1			Y																							Y																		
15091	A_23_P71884	SPINK4			Y	Y	Y	Y	Y	Y	Y	Y															Y	Y	Y	Y						Y										
1514	A_23_P10251	SPINK5							Y	Y	Y	Y	Y	Y																																
13479	A_23_P58706	SPINK5L3				Y												Y		Y			Y				Y						Y													
15685	A_23_P92909	SPINK6																				Y						Y		Y						Y										
19604	A_23_P213832	SPINK7			Y	Y	Y	Y		Y	Y	Y								Y	Y	Y	Y				Y																			
20347	A_23_P210503	SPINLW1			Y	Y		Y		Y								Y										Y																		
5613	A_23_P49057	SPINT1			Y				Y	Y									Y																											
5760	A_23_P27795	SPINT2																					Y										Y													
5780	A_23_P91240	SPINT3			Y	Y		Y		Y																																				
5752	A_23_P37825	SPIRE2				Y													Y	Y		Y						Y																		
5446	A_23_P433758	SPN				Y																																								
19718	A_23_P146842	SPNS1			Y	Y	Y	Y	Y	Y	Y	Y						Y	Y	Y	Y	Y	Y	Y	Y	Y	Y			Y	Y	Y				Y										
6438	A_23_P100963	SPNS3			Y	Y		Y	Y	Y																																				
13385	A_23_P431390	SPOCD1																		Y			Y																							
2457	A_23_P81598	SPOCK1																		Y																										
3848	A_23_P161280	SPOCK2	Y	Y	Y	Y	Y	Y		Y	Y	Y							Y								Y																			
10550	A_23_P95690	SPOCK3							Y	Y																				Y						Y										
17072	A_23_P10733	SPON1																		Y								Y																		
18640	A_23_P121533	SPON2							Y	Y										Y																										
19873	A_23_P107256	SPOP			Y																																									
9753	A_23_P79681	SPOPL							Y	Y	Y	Y	Y	Y																Y						Y										
C14348.5	A_23_P7313	SPP1			Y	Y		Y	Y	Y	Y	Y	Y	Y					Y				Y				Y			Y						Y										
829	A_23_P90829	SPP2			Y																																									
14284	A_23_P79067	SPPL2B			Y	Y		Y		Y										Y		Y	Y				Y																			
3033	A_23_P68208	SPR	Y	Y	Y																									Y						Y										
388	A_23_P54460	SPRED1																		Y																										
21791	A_23_P348208	SPRR1A			Y	Y	Y	Y	Y	Y	Y	Y								Y		Y	Y				Y																			
5777	A_23_P126089	SPRR2C				Y																								Y						Y										
14193	A_23_P148949	SPRR2E			Y															Y			Y				Y																			
6142	A_23_P62709	SPRR3																			Y																									
7332	A_23_P422018	SPRR4	Y	Y	Y																						Y																			
21983	A_23_P311010	SPRY3																				Y																								
11001	A_23_P65129	SPRYD3			Y													Y	Y	Y			Y	Y			Y																			

1938	A_23_P200096	SPSB1			Y																						Y																			
13771	A_23_P330532	SPSB3			Y	Y		Y		Y								Y	Y	Y		Y		Y																					
4974	A_23_P258381	SPSB4							Y	Y													Y																							
12568	A_23_P63158	SPTA1																Y																												
19948	A_23_P20832	SPTAN1																Y																												
15299	A_23_P65506	SPTB																		Y																										
1358	A_23_P102521	SPTBN1																Y		Y	Y	Y	Y				Y																			
19838	A_23_P98282	SPTBN2			Y																		Y				Y	Y	Y	Y																
21919	A_23_P256652	SPTBN4			Y	Y		Y	Y	Y										Y		Y	Y				Y																			
21491	A_23_P152084	SPTBN5																Y				Y								Y						Y										
9469	A_23_P210778	SPTLC3			Y		Y				Y																Y																			
21506	A_23_P146284	SQLE																Y																												
4448	A_23_P81401	SQSTM1			Y	Y		Y	Y	Y								Y	Y	Y	Y	Y	Y	Y	Y	Y	Y			Y	Y	Y				Y										
7905	A_23_P29452	SR140				Y			Y	Y										Y			Y																							
14849	A_23_P257578	SRA1			Y																																									
10877	A_23_P39517	SRBD1			Y	Y		Y		Y									Y	Y		Y																								
21307	A_23_P30223	SRD5A1			Y	Y	Y	Y	Y	Y	Y	Y	Y	Y	Y			Y	Y								Y	Y	Y	Y						Y										
20761	A_23_P121783	SRD5A3							Y	Y																																				
2173	A_23_P162446	SRGAP1			Y				Y	Y																																				
97	A_23_P200829	SRGAP2			Y																																									
11850	A_23_P109837	SRGAP3			Y	Y		Y	Y	Y										Y		Y	Y																							
976	A_23_P86653	SRGN	Y	Y	Y	Y		Y		Y																		Y																		
20301	A_23_P59718	SRI			Y	Y		Y	Y	Y									Y	Y								Y																		
6361	A_23_P74269	SRM			Y	Y	Y	Y	Y	Y	Y	Y	Y	Y				Y	Y	Y	Y	Y	Y	Y	Y	Y	Y				Y													Y		
2306	A_23_P14876	SRP14			Y				Y	Y	Y	Y	Y	Y													Y			Y						Y										
20536	A_23_P81612	SRP19																		Y																										
9678	A_23_P162938	SRP54			Y														Y				Y				Y	Y	Y	Y																
17444	A_23_P118353	SRP68			Y	Y		Y		Y									Y	Y	Y	Y					Y																			
128	A_23_P45928	SRP9			Y				Y	Y										Y			Y																							
9771	A_23_P19543	SRPK1																		Y																										
19448	A_23_P215594	SRPK2																					Y																							
4201	A_23_P125772	SRPK3							Y	Y								Y	Y	Y			Y	Y				Y																		
18922	A_23_P86943	SRPR			Y	Y		Y		Y									Y								Y																			
495	A_23_P80773	SRPRB			Y													Y	Y	Y				Y			Y	Y	Y	Y						Y										
8810	A_23_P96383	SRPX			Y																									Y						Y										
3683	A_23_P141248	SRR	Y																	Y													Y													
7041	A_23_P323166	SRRM2			Y	Y	Y	Y		Y	Y	Y										Y					Y	Y	Y	Y																
18318	A_23_P152381	SRRM2	Y		Y		Y		Y	Y	Y	Y	Y	Y					Y	Y							Y																			
6721	A_23_P110901	SRrp35							Y	Y	Y	Y	Y	Y																Y						Y										
1530	A_23_P141738	SS18			Y	Y	Y	Y		Y	Y	Y															Y																			
13685	A_23_P423926	SS18L1			Y	Y	Y	Y	Y	Y	Y	Y								Y							Y																			
4097	A_23_P166698	SS18L2																Y																												
20012	A_23_P33791	SSBP2				Y			Y	Y								Y	Y																											
10142	A_23_P500333	SSBP3																Y		Y																										
19246	A_23_P39329	SSBP4																				Y																								
16679	A_23_P102060	SSFA2			Y	Y	Y	Y		Y	Y	Y							Y	Y			Y				Y																			
6863	A_23_P99057	SSH1			Y	Y		Y		Y																																				
10168	A_23_P429560	SSH1																		Y			Y																							
14397	A_23_P150147	SSH3																				Y																								
17531	A_23_P159476	SSNA1			Y	Y		Y		Y								Y	Y	Y		Y	Y	Y			Y			Y						Y										
18188	A_23_P148095	SSNA1			Y	Y		Y		Y								Y	Y	Y		Y	Y	Y			Y			Y						Y										
15633	A_23_P203920	SSPN				Y													Y																											
3562	A_23_P126474	SSR2							Y	Y																																				
18123	A_23_P155226	SSR3			Y	Y		Y		Y																	Y																			
13586	A_23_P259172	SSR4			Y				Y	Y									Y								Y																			
19249	A_23_P36078	SSRP1							Y	Y								Y	Y	Y		Y	Y	Y			Y																			
18467	A_23_P252817	SST																				Y																								
953	A_23_P48523	SSTR1			Y																									Y						Y										
19168	A_23_P318800	SSTR2																Y										Y																		
16512	A_23_P68910	SSTR3			Y				Y	Y										Y			Y				Y			Y						Y										
7418	A_23_P77545	SSTR5																Y																												
6875	A_23_P63289	SSU72			Y	Y	Y	Y		Y	Y	Y								Y							Y	Y	Y	Y																
604	A_23_P137128	SSX1			Y	Y		Y		Y																	Y																			
11531	A_23_P201376	SSX2IP				Y			Y	Y										Y	Y		Y																							
2419	A_23_P252025	SSX3			Y																						Y																			
7974	A_23_P84367	SSX5																												Y						Y										
5010	A_23_P374233	SSX7			Y																						Y																			
22300	A_23_P47282	ST14			Y	Y	Y	Y	Y	Y	Y	Y	Y	Y				Y	Y	Y		Y		Y			Y	Y	Y	Y						Y										
350	A_23_P31893	ST3GAL1																		Y																										
1143	A_23_P77623	ST3GAL2			Y		Y		Y	Y	Y	Y	Y	Y				Y	Y	Y	Y	Y	Y	Y	Y	Y	Y			Y	Y	Y				Y										
4151	A_23_P64138	ST3GAL4																		Y																										
6239	A_23_P136573	ST3GAL5																			Y																									
C8894	A_23_P250800	ST3GAL6																												Y						Y										
1295	A_23_P132640	ST6GAL1			Y																						Y																			
2586	A_23_P131455	ST6GAL2																									Y																			
1074	A_23_P54968	ST6GALNAC1				Y																						Y																		
2290	A_23_P49499	ST6GALNAC2			Y	Y	Y	Y	Y	Y	Y	Y	Y	Y													Y			Y						Y										

20791	A_23_P94736	ST6GALNAC4				Y					Y		Y	Y					Y	Y		Y																							
15928	A_23_P33093	ST6GALNAC5							Y	Y																				Y						Y										
18780	A_23_P315892	ST6GALNAC6																		Y	Y	Y																								
396	A_23_P354704	ST8SIA1			Y				Y	Y																	Y																			
10032	A_23_P78389	ST8SIA3																Y																												
5784	A_23_P41693	ST8SIA4	Y	Y																								Y		Y						Y										
21212	A_23_P379789	ST8SIA5			Y	Y		Y	Y	Y										Y		Y					Y																			
22459	A_23_P118930	ST8SIA5																												Y						Y										
15107	A_23_P32499	STAB1	Y												Y				Y	Y			Y																							
12874	A_23_P162607	STAB2				Y			Y	Y																				Y						Y										
22037	A_23_P121059	STAC							Y	Y																																				
13492	A_23_P152535	STAC2			Y																																									
6738	A_23_P10947	STAC3							Y	Y								Y			Y		Y																							
4585	A_23_P357365	STAG1			Y	Y		Y	Y	Y										Y			Y																							
6750	A_23_P95489	STAG1			Y	Y		Y	Y	Y										Y			Y					Y																		
7664	A_23_P93818	STAG3L1																										Y																		
10068	A_23_P111477	STAG3L4			Y	Y	Y	Y	Y	Y	Y	Y								Y		Y	Y				Y	Y	Y	Y						Y										
5	A_23_P28555	STAMBP			Y																																									
6770	A_23_P7185	STAP1	Y	Y	Y				Y	Y										Y							Y																			
2769	A_23_P382188	STAP2			Y				Y	Y																	Y			Y						Y										
11931	A_23_P36345	STARD10							Y	Y	Y	Y	Y	Y													Y			Y						Y										
14181	A_23_P342727	STARD13			Y													Y	Y	Y		Y		Y			Y			Y						Y										
10100	A_23_P118451	STARD3			Y	Y		Y	Y	Y									Y	Y		Y	Y																							
17413	A_23_P327519	STARD4							Y	Y																	Y			Y						Y										
5380	A_23_P253745	STARD8							Y	Y																																				
13358	A_23_P387630	STARD8																					Y							Y	Y	Y	Y	Y	Y	Y										
16728	A_23_P57059	STAU1				Y												Y																												
16987	A_23_P33153	STAU2			Y	Y	Y	Y	Y	Y	Y	Y								Y							Y	Y	Y	Y																
14951	A_23_P168944	STC1																	Y																											
12972	A_23_P110685	STC2	Y	Y	Y	Y		Y	Y	Y									Y				Y				Y	Y	Y	Y						Y										
9189	A_23_P428260	STEAP2	Y		Y	Y		Y	Y	Y	Y	Y	Y	Y				Y	Y	Y		Y	Y	Y			Y	Y	Y	Y						Y										
11616	A_23_P20053	STEAP2			Y	Y		Y	Y	Y	Y	Y	Y	Y				Y	Y	Y				Y						Y						Y										
7731	A_23_P255231	STEAP4				Y																																								
18883	A_23_P53162	STIM1				Y																								Y						Y										
391	A_23_P81048	STIM2			Y																						Y																			
1534	A_23_P113078	STIP1																		Y																										
5256	A_23_P124470	STIP1			Y	Y	Y	Y		Y	Y	Y						Y	Y	Y	Y	Y	Y	Y	Y	Y	Y				Y															
19588	A_23_P30453	STK10			Y	Y	Y	Y	Y	Y	Y	Y	Y	Y				Y	Y	Y	Y	Y	Y	Y	Y	Y	Y				Y															
1755	A_23_P16483	STK11																	Y	Y		Y	Y																							
307	A_23_P82554	STK17A																		Y			Y																							
6524	A_23_P154367	STK17B	Y	Y	Y	Y		Y		Y					Y												Y			Y						Y										
12131	A_23_P253177	STK24			Y														Y	Y		Y																								
17346	A_23_P252653	STK25			Y	Y	Y	Y	Y	Y	Y	Y	Y	Y				Y	Y	Y	Y	Y	Y	Y	Y	Y	Y	Y	Y	Y	Y	Y				Y										
9174	A_23_P215963	STK3																					Y																							
7420	A_23_P369854	STK31																												Y						Y										
17731	A_23_P21519	STK32B			Y	Y	Y	Y	Y	Y	Y	Y															Y																			
16310	A_23_P202469	STK32C			Y	Y	Y	Y	Y	Y	Y	Y	Y	Y				Y	Y	Y	Y	Y	Y	Y	Y	Y	Y	Y	Y	Y	Y	Y	Y			Y										
16242	A_23_P127915	STK33							Y	Y										Y		Y																								
9673	A_23_P335898	STK36	Y	Y	Y	Y	Y	Y	Y	Y	Y	Y	Y	Y				Y	Y	Y	Y	Y	Y	Y	Y	Y	Y	Y	Y	Y	Y	Y	Y			Y		Y				Y			Y	Y
21009	A_23_P90762	STK39			Y	Y	Y	Y	Y	Y	Y	Y	Y	Y						Y	Y	Y	Y				Y	Y	Y	Y						Y										
16841	A_23_P74229	STK40			Y				Y	Y									Y								Y																			
9404	A_23_P200866	STMN1	Y	Y																																										
11469	A_23_P31734	STMN4				Y																						Y																		
11385	A_23_P255263	STOM				Y														Y	Y	Y																								
18708	A_23_P48910	STOML1			Y	Y		Y		Y								Y	Y	Y		Y		Y			Y			Y						Y										
14407	A_23_P256497	STOML2																		Y																										
2969	A_23_P77010	STON2			Y																						Y																			
7644	A_23_P309837	STON2			Y	Y		Y		Y																				Y						Y										
9878	A_23_P344481	STOX1			Y	Y	Y	Y	Y	Y	Y	Y							Y	Y							Y				Y		Y	Y	Y											
9274	A_23_P251364	STOX2							Y	Y	Y	Y	Y	Y																																
3745	A_23_P65779	STRA6																					Y																							
8907	A_23_P90682	STRADB			Y													Y	Y	Y			Y	Y			Y			Y	Y	Y	Y	Y	Y	Y										
1722	A_23_P13852	STRAP				Y																																								
9388	A_23_P252155	STRBP			Y	Y		Y	Y	Y										Y	Y						Y	Y	Y	Y						Y										
2486	A_23_P334758	STRC			Y		Y				Y											Y					Y																			
14305	A_23_P163236	STRC			Y	Y		Y	Y	Y	Y	Y	Y	Y						Y							Y	Y	Y	Y						Y										
1041	A_23_P65412	STRN3																									Y																			
5560	A_23_P67399	STRN4			Y														Y								Y																			
12175	A_23_P37866	STUB1																		Y		Y																								
5544	A_23_P28084	STX10			Y																																									
13168	A_23_P360353	STX10			Y	Y	Y	Y	Y	Y	Y	Y							Y	Y	Y	Y					Y			Y						Y										
14636	A_23_P156788	STX11	Y	Y	Y	Y		Y	Y	Y																	Y	Y	Y	Y						Y										
3263	A_23_P385206	STX12			Y	Y	Y	Y		Y	Y	Y						Y		Y							Y																			
14582	A_23_P109091	STX16																			Y						Y																			
16932	A_23_P73053	STX4																		Y	Y																									
22395	A_23_P24755	STX5				Y													Y	Y		Y	Y				Y	Y	Y	Y																
2198	A_23_P97725	STX6																												Y						Y										

14899	A_23_P214923	STX7																										Y																		
15087	A_23_P303260	STX7				Y														Y		Y						Y																		
6250	A_23_P135310	STXBP1							Y	Y																				Y						Y										
6348	A_23_P130614	STXBP2			Y													Y				Y					Y																			
3412	A_23_P51996	STXBP3			Y	Y		Y		Y									Y	Y		Y					Y																			
4424	A_23_P375566	STXBP4			Y	Y		Y	Y	Y	Y	Y	Y	Y																																
10833	A_23_P82047	STXBP5																		Y		Y						Y																		
4877	A_23_P117540	STYX			Y																						Y																			
10063	A_23_P111635	STYXL1			Y	Y		Y		Y																																				
18947	A_23_P59921	SUB1																					Y																							
3012	A_23_P117157	SUCLA2																Y		Y		Y	Y																							
15930	A_23_P79542	SUCLG1																		Y			Y																							
3982	A_23_P99249	SUCLG2																					Y																							
583	A_23_P69171	SUCNR1	Y		Y	Y		Y		Y																		Y																		
9810	A_23_P162791	SUGT1				Y														Y																										
13806	A_23_P43167	SULF1																	Y																											
20256	A_23_P154605	SULF2			Y	Y		Y	Y	Y									Y	Y							Y	Y	Y	Y						Y										
6191	A_23_P118167	SULT1A4																		Y																										
16766	A_23_P218275	SULT1A4				Y																						Y																		
2750	A_23_P302470	SULT1B1			Y	Y		Y		Y																																				
4146	A_23_P121622	SULT1B1			Y	Y		Y		Y									Y			Y	Y				Y																			
13154	A_23_P51002	SULT1C2																Y	Y																											
20754	A_23_P67381	SULT2A1																Y																												
6418	A_23_P107981	SULT2B1																	Y																											
5585	A_23_P131354	SULT6B1																										Y		Y						Y										
10381	A_23_P69242	SUMF1	Y	Y	Y	Y	Y	Y	Y	Y	Y	Y								Y							Y																			
21063	A_23_P123265	SUMF2			Y	Y		Y		Y								Y	Y	Y		Y		Y			Y																			
8121	A_23_P5821	SUMO1																		Y																										
9360	A_23_P102937	SUMO3	Y	Y																																										
5090	A_23_P150862	SUOX																Y																												
17877	A_23_P151625	SUPT16H			Y	Y		Y	Y	Y																																				
7979	A_23_P27835	SUPT5H			Y	Y		Y		Y								Y		Y			Y				Y																			
9242	A_23_P141479	SUPT6H			Y	Y		Y		Y												Y																								
14238	A_23_P210091	SUPT7L			Y	Y		Y	Y	Y										Y		Y						Y																		
940	A_23_P20648	SURF1							Y	Y										Y		Y								Y						Y										
5805	A_23_P32052	SURF4	Y	Y	Y	Y	Y	Y	Y	Y	Y	Y	Y	Y				Y	Y	Y	Y	Y	Y	Y	Y	Y	Y	Y	Y	Y	Y	Y	Y	Y	Y	Y				Y						
10636	A_23_P216738	SURF6			Y	Y		Y		Y								Y	Y	Y	Y	Y	Y	Y	Y	Y	Y	Y	Y	Y	Y	Y						Y								
1188	A_23_P314100	SUSD2			Y																																									
2442	A_23_P80232	SUSD2			Y				Y	Y																	Y			Y						Y										
10701	A_23_P401076	SUSD3	Y	Y	Y	Y		Y		Y	Y	Y	Y	Y				Y		Y			Y				Y			Y						Y						Y				
12710	A_23_P422193	SUV39H1			Y													Y	Y	Y		Y		Y																						
8532	A_23_P202392	SUV39H2																									Y																			
1255	A_23_P217968	SUV420H1							Y	Y																				Y						Y										
12885	A_23_P430040	SUV420H2																		Y										Y						Y										
7670	A_23_P23728	SV2A																		Y																										
415	A_23_P100022	SV2B																Y	Y	Y		Y	Y	Y				Y																		
21327	A_23_P116533	SWAP70																	Y	Y			Y																							
8519	A_23_P115805	SYCE1																									Y																			
18618	A_23_P131063	SYCN			Y				Y	Y																																				
5839	A_23_P210678	SYCP2			Y	Y	Y	Y	Y	Y	Y	Y						Y		Y			Y					Y																		
16652	A_23_P839	SYDE2																										Y																		
18297	A_23_P45756	SYF2																		Y			Y																							
7231	A_23_P9255	SYK	Y												Y								Y																							
6419	A_23_P500824	SYN1																		Y			Y																							
9435	A_23_P33847	SYN1																		Y		Y	Y				Y			Y						Y										
17374	A_23_P109811	SYN2							Y	Y																		Y		Y						Y										
5960	A_23_P80295	SYN3			Y	Y	Y	Y		Y	Y	Y															Y	Y	Y	Y																
2534	A_23_P96965	SYNC			Y	Y	Y	Y	Y	Y	Y	Y	Y	Y				Y	Y	Y	Y	Y	Y	Y	Y	Y	Y	Y	Y	Y	Y	Y	Y	Y	Y	Y										
3515	A_23_P500861	SYNE1			Y	Y		Y		Y												Y																								
13819	A_23_P211528	SYNGR1			Y	Y		Y	Y	Y													Y				Y	Y	Y	Y																
14657	A_23_P130203	SYNGR2			Y	Y		Y	Y	Y			Y					Y	Y	Y		Y	Y	Y			Y			Y						Y										
6775	A_23_P88913	SYNGR3																			Y									Y						Y										
16500	A_23_P164927	SYNGR4																		Y		Y	Y																							
15065	A_23_P316974	SYNJ2	Y	Y	Y	Y	Y	Y	Y	Y	Y	Y	Y	Y					Y	Y		Y	Y				Y	Y	Y	Y						Y										
5552	A_23_P65357	SYNJ2BP							Y	Y	Y	Y	Y	Y				Y	Y			Y																								
14324	A_23_P3323	SYNM				Y			Y	Y																				Y						Y										
15543	A_23_P344531	SYNPO			Y	Y	Y	Y		Y	Y	Y							Y								Y			Y						Y										
9212	A_23_P12719	SYNPO2L			Y	Y		Y		Y																																				
13299	A_23_P355517	SYNPO2L			Y															Y		Y	Y				Y																			
9539	A_23_P136935	SYP			Y				Y	Y													Y				Y																			
18638	A_23_P93881	SYPL1																		Y	Y		Y																							
2622	A_23_P36795	SYT1	Y	Y	Y	Y		Y	Y	Y	Y	Y	Y	Y													Y	Y	Y	Y						Y										
8770	A_23_P12018	SYT11																		Y		Y	Y																							
16236	A_23_P424645	SYT12							Y	Y																				Y						Y										
14245	A_23_P161659	SYT13			Y	Y	Y	Y	Y	Y	Y	Y	Y	Y						Y			Y				Y	Y	Y	Y						Y										
14736	A_23_P163697	SYT17			Y	Y		Y	Y	Y	Y	Y	Y	Y													Y			Y						Y										
422	A_23_P55917	SYT3			Y	Y		Y	Y	Y										Y	Y		Y				Y	Y	Y	Y						Y										

2708	A_23_P208031	SYT4																													Y						Y											
12318	A_23_P78851	SYT5			Y	Y		Y		Y											Y			Y					Y																				
17415	A_23_P398947	SYT6	Y	Y	Y																								Y																		
9603	A_23_P36187	SYT8				Y																																									
7188	A_23_P306310	SYT9			Y				Y	Y																	Y																				
16663	A_23_P53193	SYTL2	Y	Y	Y	Y		Y	Y	Y	Y	Y	Y	Y					Y	Y		Y	Y				Y	Y	Y	Y						Y											
15656	A_23_P168188	SYTL3	Y	Y																						Y																		
14975	A_23_P11136	SYTL4			Y		Y				Y								Y									Y																				
4664	A_23_P317082	SYTL5																										Y																	
14364	A_23_P138826	SYVN1			Y		Y				Y									Y	Y									Y			Y						Y											
1889	A_23_P93531	TAAR1			Y																																										
21081	A_23_P59331	TAAR3				Y														Y									Y			Y						Y											
9805	A_23_P328679	TAAR6				Y																																									
22130	A_23_P8269	TAAR8																									Y	Y	Y	Y						Y											
12667	A_23_P215283	TAC1			Y																																										
20143	A_23_P2283	TAC3	Y	Y	Y																						Y																		
4233	A_23_P60047	TACC1																		Y	Y		Y																									
5584	A_23_P127186	TACC2			Y				Y	Y																																				
1206	A_23_P212844	TACC3			Y	Y		Y		Y								Y	Y	Y		Y	Y	Y			Y																					
6363	A_23_P83720	TACR1																					Y					Y																	
1652	A_23_P24135	TACR2																													Y						Y											
18635	A_23_P110299	TACR3																Y																											
7460	A_23_P74344	TADA1L			Y	Y		Y	Y	Y										Y		Y	Y																								
832	A_23_P11237	TAF1																													Y						Y											
20444	A_23_P203466	TAF10																		Y											Y						Y											
19262	A_23_P74663	TAF1A																		Y																									
14251	A_23_P165891	TAF1B																	Y	Y		Y					Y																		
7233	A_23_P64029	TAF1D																		Y			Y																						
7367	A_23_P419850	TAF1L				Y																								Y						Y											
11683	A_23_P80062	TAF4			Y				Y	Y																	Y																				
19423	A_23_P95718	TAF4B			Y				Y	Y																																				
15462	A_23_P122876	TAF6																Y	Y	Y		Y		Y			Y																					
1318	A_23_P47535	TAF6L																	Y	Y		Y	Y					Y		Y						Y											
14909	A_23_P81248	TAF7																		Y							Y																		
13770	A_23_P41604	TAF9			Y	Y	Y	Y	Y	Y	Y	Y							Y	Y		Y	Y				Y	Y	Y	Y																
18666	A_23_P21734	TAF9																		Y			Y																						
11724	A_23_P254790	TAF9B			Y	Y		Y	Y	Y	Y	Y	Y	Y													Y																		
1616	A_23_P253145	TAGAP	Y	Y	Y				Y	Y					Y					Y							Y																		
8431	A_23_P87013	TAGLN																Y		Y			Y																						
18779	A_23_P74138	TAGLN2			Y	Y	Y	Y	Y	Y	Y	Y	Y	Y				Y	Y	Y		Y	Y	Y			Y	Y	Y	Y						Y											
18403	A_23_P80817	TAGLN3																Y		Y	Y		Y																					
5280	A_23_P44986	TALDO1			Y		Y				Y															Y			Y						Y											
22138	A_23_P218346	TANC2																									Y																		
11837	A_23_P154306	TANK																		Y																									
20912	A_23_P218269	TAOK2			Y	Y	Y	Y		Y	Y	Y						Y	Y	Y	Y	Y	Y	Y	Y	Y	Y			Y	Y	Y				Y											
6840	A_23_P59005	TAP1			Y	Y	Y	Y	Y	Y	Y	Y	Y														Y	Y	Y	Y						Y								Y		
2429	A_23_P259573	TAPBP			Y	Y	Y	Y		Y	Y	Y							Y									Y	Y	Y	Y																
14054	A_23_P36698	TAPBPL			Y	Y		Y	Y	Y																	Y																		
357	A_23_P301372	TAPT1			Y	Y	Y	Y	Y	Y	Y	Y	Y	Y				Y	Y	Y		Y	Y	Y			Y	Y	Y	Y						Y											
13574	A_23_P52058	TARBP1			Y															Y	Y	Y	Y				Y																		
716	A_23_P403955	TARDBP			Y	Y	Y	Y	Y	Y	Y	Y	Y	Y				Y	Y	Y		Y		Y			Y	Y	Y	Y						Y								Y		
6342	A_23_P200641	TARDBP			Y	Y	Y	Y	Y	Y	Y	Y	Y	Y					Y	Y		Y	Y				Y	Y	Y	Y																
22542	A_23_P59543	TARP			Y	Y		Y	Y	Y								Y		Y	Y		Y				Y	Y	Y	Y						Y											
8719	A_23_P70168	TARS																				Y																							
8484	A_23_P551	TARS2	Y	Y																																									
1338	A_23_P160890	TAS1R1			Y				Y	Y										Y							Y																		
10868	A_23_P335957	TAS1R2			Y																		Y				Y																		
492	A_23_P74684	TAS1R3			Y																						Y																		
18704	A_23_P402123	TAS2R1																Y										Y																	
15816	A_23_P64888	TAS2R10			Y	Y		Y	Y	Y										Y		Y	Y				Y																		
20360	A_23_P99080	TAS2R13																		Y		Y																							
9035	A_23_P105495	TAS2R14	Y	Y	Y													Y	Y																										
10283	A_23_P59825	TAS2R16			Y				Y	Y																	Y			Y						Y											
2571	A_23_P359746	TAS2R38																Y																											
9597	A_23_P20101	TAS2R4			Y													Y				Y																							
8102	A_23_P339652	TAS2R40																				Y																							
15320	A_23_P371254	TAS2R43																Y																											
1829	A_23_P416191	TAS2R44			Y				Y	Y																	Y			Y						Y											
19239	A_23_P303851	TAS2R45																										Y																	
10931	A_23_P340308	TAS2R48			Y	Y		Y		Y					Y					Y		Y																							
9308	A_23_P382794	TAS2R49																		Y																									
18939	A_23_P367013	TAS2R60			Y	Y		Y	Y	Y										Y		Y	Y				Y			Y						Y											
2551	A_23_P48022	TAS2R8			Y	Y		Y		Y																	Y			Y						Y											
9139	A_23_P336506	TAS2R9				Y																																									
405	A_23_P120557	TASP1			Y	Y		Y		Y										Y			Y																						
C13079.7	A_23_P254974	TATDN1				Y																																									
3328	A_23_P57823	TATDN2																		Y			Y																						

2834	A_23_P215360	TAX1BP1							Y	Y	Y	Y	Y	Y					Y	Y		Y																							
16913	A_23_P38468	TAX1BP3			Y	Y		Y		Y									Y	Y		Y					Y			Y						Y										
4682	A_23_P62371	TAZ			Y	Y		Y		Y								Y	Y	Y	Y	Y	Y	Y	Y	Y	Y			Y	Y	Y	Y			Y										
7055	A_23_P73023	TBC1D1			Y	Y	Y	Y	Y	Y	Y	Y	Y	Y				Y	Y	Y	Y	Y	Y	Y	Y	Y	Y			Y	Y	Y				Y				Y						
7929	A_23_P166421	TBC1D10A			Y	Y		Y		Y								Y	Y	Y		Y	Y	Y				Y		Y						Y										
7940	A_23_P22382	TBC1D10B																		Y		Y	Y																							
6600	A_23_P24215	TBC1D12			Y	Y	Y	Y	Y	Y	Y	Y	Y	Y					Y	Y		Y	Y				Y	Y	Y	Y						Y										
9629	A_23_P155826	TBC1D14																Y																												
7651	A_23_P139558	TBC1D15																	Y																											
20575	A_23_P207786	TBC1D16			Y																																									
11933	A_23_P130731	TBC1D17																		Y			Y																							
9633	A_23_P136199	TBC1D19			Y	Y		Y	Y	Y	Y	Y	Y	Y						Y	Y	Y	Y																							
5417	A_23_P28843	TBC1D20							Y	Y																				Y						Y										
6807	A_23_P408205	TBC1D21			Y																						Y																			
8391	A_23_P109593	TBC1D22A			Y	Y		Y	Y	Y								Y		Y	Y	Y	Y				Y			Y						Y										
12947	A_23_P58980	TBC1D22B			Y	Y		Y	Y	Y																	Y																			
1434	A_23_P212720	TBC1D23																	Y	Y			Y																							
9380	A_23_P164383	TBC1D29			Y																									Y						Y										
7500	A_23_P396666	TBC1D2B			Y										Y			Y	Y	Y		Y	Y	Y			Y																			
3737	A_23_P78068	TBC1D3																												Y						Y										
11790	A_23_P111273	TBC1D7			Y				Y	Y								Y		Y										Y						Y										
12370	A_23_P253281	TBC1D8			Y															Y	Y		Y				Y																			
20280	A_23_P114172	TBC1D8B																	Y		Y		Y																							
17750	A_23_P41487	TBC1D9	Y	Y	Y	Y	Y	Y	Y	Y	Y	Y	Y	Y				Y	Y	Y	Y	Y	Y	Y	Y	Y	Y	Y	Y	Y	Y	Y				Y		Y				Y			Y	Y
21286	A_23_P251611	TBC1D9B			Y																						Y																			
18495	A_23_P56254	TBCB			Y	Y	Y	Y	Y	Y	Y	Y						Y	Y	Y		Y		Y			Y																			
10711	A_23_P100602	TBCD			Y	Y		Y		Y								Y	Y	Y		Y	Y	Y			Y																			
17077	A_23_P410017	TBCEL			Y													Y	Y	Y	Y	Y	Y	Y	Y	Y	Y				Y															
13808	A_23_P44768	TBK1			Y	Y		Y		Y										Y								Y																		
9377	A_23_P130169	TBKBP1			Y		Y				Y		Y	Y						Y			Y				Y			Y						Y										
2868	A_23_P259930	TBL1X							Y	Y																				Y						Y										
5342	A_23_P114466	TBL1Y							Y	Y																																				
18321	A_23_P134395	TBL2																Y																												
2453	A_23_P106717	TBL3			Y															Y							Y			Y						Y										
17370	A_23_P19313	TBP																		Y	Y	Y	Y																							
18855	A_23_P98463	TBRG1			Y														Y	Y																										
3773	A_23_P211345	TBX1																												Y						Y										
7621	A_23_P62981	TBX15							Y	Y											Y									Y						Y										
3026	A_23_P134041	TBX18			Y														Y	Y		Y																								
11171	A_23_P137705	TBX19							Y	Y	Y	Y	Y	Y																Y						Y										
11728	A_23_P164451	TBX2																		Y	Y		Y																							
14202	A_23_P141555	TBX21	Y	Y	Y				Y	Y																	Y																			
20390	A_23_P159676	TBX22																	Y																											
17991	A_23_P383819	TBX3	Y	Y	Y	Y	Y	Y	Y	Y	Y	Y	Y	Y													Y	Y	Y	Y						Y										
21633	A_23_P204100	TBX3	Y	Y	Y	Y	Y	Y	Y	Y	Y	Y	Y	Y						Y			Y				Y	Y	Y	Y						Y										
8036	A_23_P431225	TBX6							Y	Y																																				
11530	A_23_P90357	TBXA2R																		Y	Y		Y																							
21280	A_23_P71197	TBXAS1			Y	Y		Y		Y										Y																										
4564	A_23_P88439	TC2N							Y	Y	Y	Y	Y	Y						Y		Y																								
7491	A_23_P413075	TC2N			Y						Y		Y	Y																																
2405	A_23_P156996	tcag7.1196																Y	Y	Y				Y																						
5688	A_23_P82245	tcag7.1260			Y	Y	Y	Y	Y	Y	Y	Y	Y	Y													Y	Y	Y	Y						Y										
12356	A_23_P107051	TCAP			Y				Y	Y																		Y																		
20874	A_23_P147635	TCEA2	Y	Y	Y																						Y	Y	Y	Y																
6327	A_23_P34375	TCEA3																									Y																			
270	A_23_P73801	TCEAL1			Y	Y		Y	Y	Y										Y			Y				Y	Y	Y	Y						Y										
10250	A_23_P85140	TCEAL2			Y				Y	Y	Y	Y	Y	Y					Y	Y			Y				Y			Y						Y										
7018	A_23_P259166	TCEAL4			Y	Y	Y	Y	Y	Y	Y	Y	Y	Y						Y			Y				Y	Y	Y	Y						Y										
1320	A_23_P62399	TCEAL5	Y	Y	Y	Y	Y	Y		Y	Y	Y	Y	Y						Y			Y				Y	Y	Y	Y						Y										
14995	A_23_P11331	TCEAL8			Y	Y		Y		Y																																				
19587	A_23_P74097	TCEB3			Y	Y		Y		Y									Y	Y		Y					Y																			
11077	A_23_P315910	TCEB3C																									Y			Y						Y										
15456	A_23_P133365	TCERG1			Y	Y		Y	Y	Y																				Y						Y										
18786	A_23_P336624	TCHH																			Y																									
11571	A_23_P151159	TCHP																Y																												
20873	A_23_P75369	TCIRG1				Y														Y																										
8068	A_23_P357717	TCL1A	Y		Y	Y		Y		Y									Y								Y																			
12434	A_23_P363196	TCL6																		Y			Y																							
17659	A_23_P64372	TCN1																Y																												
2082	A_23_P40605	TCN2	Y	Y	Y	Y	Y	Y	Y	Y	Y	Y	Y	Y				Y	Y	Y	Y	Y	Y	Y	Y	Y	Y	Y	Y	Y	Y	Y	Y	Y	Y											
2045	A_23_P310317	TCOF1																Y	Y	Y	Y			Y																						
9909	A_23_P350555	TCP10L			Y																																									
11489	A_23_P419107	TCP11L2			Y														Y				Y				Y																			
2516	A_23_P58005	TCTA			Y				Y	Y									Y	Y							Y			Y						Y										
1752	A_23_P76402	TCTN1	Y	Y	Y	Y		Y	Y	Y	Y	Y	Y	Y				Y	Y	Y		Y		Y			Y	Y	Y	Y						Y										
12816	A_23_P64712	TCTN2			Y	Y		Y	Y	Y									Y	Y			Y				Y						Y													
5633	A_23_P373695	TCTN3	Y	Y	Y	Y		Y	Y	Y	Y	Y	Y	Y					Y	Y		Y					Y	Y	Y	Y						Y										

16450	A_23_P204579	TDG				Y																																							
5698	A_23_P366376	TDGF1			Y																																								
15213	A_23_P337778	TDH																		Y		Y																									
7074	A_23_P80974	TDO2	Y	Y	Y		Y		Y	Y	Y	Y										Y					Y																			
14139	A_23_P97835	TDRD1																		Y																										
17753	A_23_P2711	TDRD3			Y	Y	Y	Y	Y	Y	Y	Y						Y	Y	Y		Y		Y			Y	Y	Y	Y						Y										
6282	A_23_P123672	TDRD7			Y	Y	Y	Y		Y	Y	Y						Y	Y	Y		Y	Y	Y			Y																			
10696	A_23_P46351	TDRKH																Y	Y																											
12625	A_23_P119344	TEAD2			Y				Y	Y	Y	Y	Y	Y				Y	Y	Y		Y		Y			Y	Y	Y	Y						Y										
9392	A_23_P94795	TEAD4							Y	Y								Y	Y	Y		Y	Y	Y			Y			Y						Y										
13340	A_23_P32758	TEAD4							Y	Y								Y	Y	Y		Y	Y	Y			Y			Y						Y										
12864	A_23_P104851	TECTA			Y															Y		Y	Y																							
9854	A_23_P97826	TECTB			Y	Y		Y	Y	Y								Y	Y	Y		Y		Y			Y	Y	Y	Y						Y										
9184	A_23_P374699	TEK				Y																																							
4453	A_23_P15677	TEKT1			Y	Y		Y		Y													Y					Y																		
21490	A_23_P45955	TEKT2																		Y										Y						Y										
16824	A_23_P323290	TEKT4			Y	Y		Y	Y	Y										Y		Y					Y	Y	Y	Y						Y										
20	A_23_P343357	TEKT5			Y	Y		Y		Y																	Y	Y	Y	Y																
5356	A_23_P206237	TELO2																Y	Y	Y		Y	Y	Y																						
4310	A_23_P151297	TENC1																Y	Y	Y		Y	Y	Y																						
4510	A_23_P65481	TEP1																										Y																		
8869	A_23_P216149	TERF1			Y	Y		Y		Y																																				
3831	A_23_P417200	TERF2			Y														Y								Y																			
20537	A_23_P129704	TERF2IP																												Y						Y										
18084	A_23_P110851	TERT																				Y																								
9351	A_23_P111672	TES	Y	Y	Y																	Y																								
3789	A_23_P76538	TESC																					Y																							
15568	A_23_P169178	TESK1																Y	Y	Y		Y	Y	Y																						
7785	A_23_P23394	TESK2							Y	Y	Y	Y	Y	Y																Y						Y										
4290	A_23_P135938	TESSP5																												Y						Y										
9813	A_23_P112412	TEX10							Y	Y										Y		Y								Y						Y										
11350	A_23_P96501	TEX11			Y				Y	Y																	Y																			
22348	A_23_P123307	TEX15				Y																																							
20156	A_23_P4036	TEX2							Y	Y																																			
22137	A_23_P251545	TEX261	Y	Y	Y	Y		Y	Y	Y	Y	Y	Y	Y				Y	Y								Y	Y	Y	Y						Y										
3502	A_23_P41005	TEX264			Y	Y		Y		Y									Y	Y																										
16879	A_23_P257936	TEX28							Y	Y																																			
4914	A_23_P212500	TF							Y	Y																																			
13598	A_23_P69372	TF																	Y																											
2679	A_23_P68759	TFF1			Y	Y		Y	Y	Y	Y	Y	Y	Y													Y	Y	Y	Y						Y								Y		
11164	A_23_P57364	TFF2																Y	Y	Y		Y		Y																						
2811	A_23_P393099	TFF3							Y	Y	Y	Y	Y	Y						Y										Y						Y										
12400	A_23_P257296	TFF3				Y			Y	Y	Y	Y	Y	Y						Y							Y			Y						Y										
10762	A_23_P17095	TFPI																					Y																							
282	A_23_P71103	TFPI2																									Y	Y	Y	Y						Y										
18292	A_23_P393620	TFPI2																Y									Y	Y	Y	Y						Y										
15193	A_23_P27573	TFPT			Y															Y							Y																			
881	A_23_P122896	TFR2							Y	Y																		Y		Y						Y										
16218	A_23_P65217	TGDS																		Y																										
9714	A_23_P209842	TGFA			Y															Y							Y																			
12974	A_23_P377284	TGFA			Y	Y		Y	Y	Y	Y	Y	Y	Y													Y	Y	Y	Y						Y										
7350	A_23_P412335	TGFB1			Y														Y	Y	Y	Y	Y																							
20333	A_23_P141055	TGFB1I1			Y				Y	Y								Y	Y	Y	Y	Y	Y	Y	Y	Y					Y		Y													
12805	A_23_P11959	TGFB2																	Y																											
17955	A_23_P88404	TGFB3																										Y																		
12298	A_23_P156327	TGFBI							Y	Y									Y																											
9572	A_23_P123943	TGFBR1																Y			Y																									
17214	A_23_P337242	TGFBR2																		Y			Y																							
19004	A_23_P120146	TGFBRAP1			Y													Y		Y																										
8507	A_23_P500074	TGIF1				Y																																							
18904	A_23_P65617	TGM1			Y				Y	Y	Y	Y	Y	Y																															
11119	A_23_P120393	TGM2			Y	Y	Y	Y		Y	Y	Y						Y	Y	Y	Y	Y	Y	Y	Y	Y	Y	Y	Y	Y	Y	Y	Y					Y				Y			Y	
3399	A_23_P57118	TGM3			Y														Y																											
12005	A_23_P129291	TGM5				Y																																							
22102	A_23_P117896	TGM7				Y																																							
17195	A_23_P60899	TGS1																			Y																									
4506	A_23_P95891	TH			Y	Y		Y	Y	Y									Y	Y								Y		Y						Y										
9677	A_23_P258633	TH			Y																																								
18940	A_23_P210649	TH1L							Y	Y									Y								Y																			
12936	A_23_P134814	THAP1																		Y																										
1081	A_23_P106391	THAP10			Y																						Y																			
8666	A_23_P109410	THAP7			Y													Y	Y	Y		Y		Y			Y																			
17565	A_23_P253652	THBS2																	Y																											
20214	A_23_P62021	THBS2																	Y																											
1602	A_23_P201047	THBS3																					Y																							
19555	A_23_P250362	THBS4																		Y			Y																							
2810	A_23_P131074	THEG																				Y																								

274	A_23_P31116	THEM2				Y			Y	Y																																				
19918	A_23_P121956	THG1L			Y	Y		Y		Y																			Y																			
15951	A_23_P127002	THNSL1																					Y																							
13472	A_23_P259201	THNSL2			Y		Y		Y	Y	Y	Y						Y		Y	Y	Y	Y				Y			Y			Y			Y										
875	A_23_P78372	THOC1				Y																																								
3236	A_23_P11051	THOC2																			Y																									
3203	A_23_P152984	THOC4			Y	Y		Y	Y	Y								Y	Y	Y	Y	Y	Y	Y	Y	Y	Y	Y	Y	Y	Y	Y				Y				Y						
9354	A_23_P109462	THOC5			Y	Y	Y	Y	Y	Y	Y	Y	Y	Y				Y	Y	Y		Y	Y	Y			Y	Y	Y	Y																
8679	A_23_P37949	THOC6																Y	Y	Y	Y	Y	Y	Y	Y	Y	Y				Y															
1236	A_23_P132705	THOC7																		Y																										
6453	A_23_P28057	THOP1			Y	Y		Y		Y										Y							Y																			
5762	A_23_P105212	THRSP							Y	Y																				Y						Y										
12598	A_23_P363954	THRSP			Y				Y	Y																				Y						Y										
9342	A_23_P14184	THSD1			Y	Y	Y	Y	Y	Y	Y	Y						Y	Y	Y		Y		Y			Y	Y	Y	Y						Y										
10109	A_23_P65717	THSD4																Y																												
10334	A_23_P148249	THSD4	Y	Y	Y	Y		Y	Y	Y	Y	Y	Y	Y				Y	Y	Y		Y	Y	Y			Y	Y	Y	Y	Y	Y	Y	Y	Y	Y		Y				Y			Y	Y
6092	A_23_P118330	THUMPD1			Y	Y		Y	Y	Y	Y	Y	Y	Y					Y	Y							Y	Y	Y	Y						Y										
13467	A_23_P131348	THUMPD2							Y	Y													Y							Y						Y										
358	A_23_P36364	THY1																		Y			Y																							
18453	A_23_P47756	THY1			Y		Y				Y							Y		Y		Y	Y				Y																			
16172	A_23_P24633	THYN1																Y		Y																										
18712	A_23_P90839	TIA1				Y														Y		Y	Y																							
15251	A_23_P161488	TIAL1			Y																																									
8624	A_23_P252426	TIAM2							Y	Y																																				
10412	A_23_P90311	TICAM1			Y	Y		Y		Y																	Y																			
21890	A_23_P376102	TICAM1			Y																						Y																			
14708	A_23_P126422	TIE1			Y	Y		Y	Y	Y								Y	Y	Y	Y	Y	Y	Y	Y	Y	Y	Y	Y	Y	Y	Y	Y	Y	Y											
4402	A_23_P304420	TIGD2																			Y																									
12450	A_23_P415663	TIGD3																	Y																											
15226	A_23_P370684	TIGD4																					Y				Y																			
3436	A_23_P20434	TIGD5			Y	Y		Y		Y								Y	Y								Y	Y	Y	Y																
21077	A_23_P213966	TIGD6			Y	Y	Y	Y	Y	Y	Y	Y								Y	Y	Y	Y				Y	Y	Y	Y						Y						Y				
12620	A_23_P129577	TIGD7																Y		Y			Y																							
15736	A_23_P80529	TIGIT	Y	Y	Y				Y	Y								Y									Y																			
12839	A_23_P7503	TIMD4	Y	Y	Y		Y		Y	Y	Y	Y			Y			Y									Y																			
13821	A_23_P53276	TIMELESS			Y																																									
10876	A_23_P33886	TIMM17B			Y	Y	Y	Y		Y	Y	Y						Y	Y	Y		Y	Y	Y			Y																			
3615	A_23_P55468	TIMM22				Y			Y	Y																	Y	Y	Y	Y						Y										
14989	A_23_P119214	TIMM44			Y	Y	Y	Y	Y	Y	Y	Y	Y	Y				Y	Y	Y	Y	Y		Y			Y	Y	Y	Y						Y										
12135	A_23_P358417	TIMM8A	Y	Y	Y	Y		Y	Y	Y								Y	Y	Y		Y		Y			Y	Y	Y	Y						Y										
19260	A_23_P217637	TIMM8A			Y	Y		Y	Y	Y																	Y	Y	Y	Y						Y										
11869	A_23_P98382	TIMM8B			Y																						Y																			
8887	A_23_P76918	TIMM9			Y	Y		Y		Y										Y		Y					Y																			
4196	A_23_P399078	TIMP3																Y																												
6080	A_23_P69208	TIMP4			Y	Y	Y	Y		Y	Y	Y								Y							Y	Y	Y	Y						Y										
11394	A_23_P85585	TINAGL1																												Y						Y										
888	A_23_P77020	TINF2			Y																						Y																			
12042	A_23_P167438	TINP1			Y	Y		Y	Y	Y										Y			Y				Y	Y	Y	Y																
3042	A_23_P143845	TIPARP			Y	Y		Y	Y	Y	Y	Y	Y	Y				Y		Y							Y	Y	Y	Y						Y										
16496	A_23_P327426	TIPRL	Y	Y																																										
21627	A_23_P81811	TJAP1			Y				Y	Y								Y	Y	Y			Y	Y			Y	Y	Y	Y						Y										
8040	A_23_P205828	TJP1																					Y																							
11145	A_23_P9293	TJP2																		Y																										
16625	A_23_P108157	TJP3							Y	Y									Y																											
21680	A_23_P107421	TK1			Y	Y	Y	Y		Y	Y	Y	Y	Y				Y		Y							Y	Y	Y	Y						Y										
19113	A_23_P92082	TKT			Y	Y	Y	Y	Y	Y	Y	Y						Y	Y	Y	Y	Y	Y	Y	Y	Y	Y	Y	Y	Y	Y	Y								Y						
12771	A_23_P259901	TKTL1				Y														Y																										
20317	A_23_P18662	TKTL2																		Y		Y																								
16060	A_23_P420417	TLCD1							Y	Y									Y																											
7528	A_23_P135239	TLE1			Y																																									
19479	A_23_P94493	TLE4							Y	Y																																				
7040	A_23_P426501	TLE6	Y	Y	Y															Y			Y				Y																			
17924	A_23_P39684	TLK1																					Y																							
3930	A_23_P436155	TLL2																			Y																									
4154	A_23_P9170	TLN1			Y													Y		Y			Y							Y						Y										
7819	A_23_P250651	TLN2																Y	Y			Y																								
20461	A_23_P10873	TLR1	Y	Y																																										
11499	A_23_P92499	TLR2	Y	Y																																										
13582	A_23_P29922	TLR3																	Y	Y																										
17096	A_23_P85903	TLR5																	Y																											
6874	A_23_P85240	TLR7													Y																															
17008	A_23_P62436	TLR7	Y		Y	Y	Y	Y	Y	Y	Y	Y			Y												Y	Y	Y	Y						Y										
17928	A_23_P73837	TLR8	Y	Y	Y		Y		Y	Y	Y	Y			Y																															
7661	A_23_P132654	TLR9											Y						Y																											
9726	A_23_P24028	TLX1																Y	Y	Y		Y	Y	Y			Y	Y	Y	Y																
13112	A_23_P70219	TLX3			Y	Y	Y	Y		Y	Y	Y	Y	Y						Y			Y				Y	Y	Y	Y						Y										
1955	A_23_P148959	TM2D1																		Y			Y				Y	Y	Y	Y																

19997	A_23_P10927	TM2D2			Y						Y		Y	Y																																
19408	A_23_P140515	TM2D3							Y	Y																				Y						Y										
1725	A_23_P114008	TM4SF20																										Y		Y						Y										
15960	A_23_P252432	TM4SF4			Y																						Y																			
3298	A_23_P27107	TM4SF5			Y						Y		Y	Y													Y																			
5873	A_23_P142403	TM6SF2							Y	Y																		Y		Y						Y										
21601	A_23_P127426	TM7SF2							Y	Y																				Y						Y										
18422	A_23_P47863	TM7SF3			Y				Y	Y																																				
4542	A_23_P151586	TM9SF1	Y		Y						Y		Y	Y													Y																			
11175	A_23_P210872	TM9SF4			Y	Y	Y	Y	Y	Y	Y	Y	Y	Y													Y																	Y		
1932	A_23_P79331	TMBIM1			Y	Y	Y	Y	Y	Y	Y	Y	Y	Y				Y	Y	Y	Y	Y	Y	Y	Y	Y	Y	Y	Y	Y	Y	Y				Y										
494	A_23_P13694	TMBIM4							Y	Y	Y	Y	Y	Y																																
16264	A_23_P204702	TMBIM6			Y																																									
20345	A_23_P305527	TMC2			Y	Y		Y	Y	Y								Y									Y	Y	Y	Y						Y										
10589	A_23_P258783	TMC3			Y																						Y			Y						Y										
4051	A_23_P15101	TMC5	Y	Y					Y	Y	Y	Y	Y	Y				Y	Y	Y	Y	Y	Y	Y	Y	Y	Y	Y	Y	Y	Y	Y				Y		Y				Y			Y	Y
3704	A_23_P101013	TMC6			Y	Y		Y	Y	Y									Y	Y		Y	Y				Y			Y						Y										
2054	A_23_P152991	TMC8			Y	Y		Y		Y																		Y		Y						Y										
18329	A_23_P170986	TMCC1			Y	Y		Y		Y									Y	Y		Y	Y				Y																			
7217	A_23_P322530	TMCC2																	Y			Y																								
4574	A_23_P87853	TMCC3	Y		Y				Y	Y								Y	Y								Y																			
7345	A_23_P46084	TMCO1																		Y			Y				Y																			
19519	A_23_P205188	TMCO3							Y	Y																	Y			Y						Y										
2665	A_23_P158276	TMCO4																		Y																										
16070	A_23_P65857	TMCO5A			Y	Y		Y	Y	Y																	Y	Y	Y	Y						Y										
3892	A_23_P78694	TMED1			Y	Y	Y	Y	Y	Y	Y	Y	Y							Y							Y	Y	Y	Y						Y										
22285	A_23_P36448	TMED2			Y	Y		Y		Y								Y		Y	Y	Y					Y						Y													
7413	A_23_P26173	TMED3			Y	Y		Y	Y	Y										Y		Y						Y																		
18886	A_23_P86002	TMED5	Y	Y	Y	Y		Y		Y																	Y																			
22440	A_23_P70127	TMED9			Y	Y		Y		Y										Y		Y																								
2100	A_23_P135194	TMEFF1			Y	Y		Y	Y	Y																	Y			Y						Y										
14855	A_23_P125387	TMEFF2				Y																					Y																			
17795	A_23_P15516	TMEM101			Y																																									
16111	A_23_P334892	TMEM102			Y	Y	Y	Y	Y	Y	Y	Y	Y	Y				Y	Y	Y		Y	Y	Y			Y	Y	Y	Y						Y										
21902	A_23_P435513	TMEM106A																		Y	Y		Y																							
10829	A_23_P8522	TMEM106B							Y	Y									Y	Y	Y	Y	Y																							
7677	A_23_P48175	TMEM106C			Y	Y	Y	Y	Y	Y	Y	Y																																		
8656	A_23_P118790	TMEM107			Y													Y		Y							Y																			
22260	A_23_P254688	TMEM108							Y	Y																	Y			Y						Y										
19200	A_23_P203367	TMEM109			Y	Y	Y	Y	Y	Y	Y	Y	Y	Y				Y	Y	Y	Y	Y	Y	Y	Y	Y	Y				Y															
12244	A_23_P107036	TMEM11																										Y																		
12635	A_23_P69221	TMEM111							Y	Y																																				
7805	A_23_P6866	TMEM115																		Y		Y	Y																							
15545	A_23_P105619	TMEM116							Y	Y	Y	Y	Y	Y						Y							Y																			
19202	A_23_P2573	TMEM117			Y				Y	Y									Y	Y							Y																			
12181	A_23_P259632	TMEM120A			Y	Y		Y		Y																	Y																			
13705	A_23_P61987	TMEM121			Y				Y	Y																	Y																			
15143	A_23_P202964	TMEM123			Y	Y		Y	Y	Y	Y	Y	Y	Y				Y		Y		Y					Y			Y						Y						Y				
9680	A_23_P115022	TMEM125											Y								Y																									
22406	A_23_P100155	TMEM127			Y		Y				Y		Y	Y				Y		Y			Y				Y			Y						Y										
11988	A_23_P29927	TMEM128			Y															Y			Y				Y																			
17630	A_23_P150325	TMEM133							Y	Y									Y	Y		Y	Y																							
20717	A_23_P161523	TMEM134			Y	Y		Y	Y	Y										Y			Y				Y			Y						Y										
8338	A_23_P203751	TMEM135							Y	Y	Y	Y	Y	Y						Y			Y				Y																			
20265	A_23_P329890	TMEM136			Y																		Y																							
14358	A_23_P24723	TMEM138	Y		Y	Y	Y	Y		Y	Y	Y															Y	Y	Y	Y																
20126	A_23_P42908	TMEM139							Y	Y			Y																																	
3183	A_23_P31176	TMEM140			Y																		Y																							
21682	A_23_P133120	TMEM144																										Y																		
3442	A_23_P79043	TMEM147			Y	Y		Y		Y																	Y																			
6071	A_23_P142424	TMEM149	Y	Y	Y		Y		Y	Y	Y	Y	Y	Y					Y	Y							Y	Y	Y	Y																
6806	A_23_P42080	TMEM14A							Y	Y																				Y						Y										
11278	A_23_P250974	TMEM14B																		Y																										
17844	A_23_P111288	TMEM14C							Y	Y										Y																										
14621	A_23_P5641	TMEM150							Y	Y																																				
16175	A_23_P354217	TMEM151A																					Y					Y																		
10401	A_23_P92330	TMEM156							Y	Y			Y															Y																		
16519	A_23_P369899	TMEM158			Y				Y	Y	Y	Y	Y	Y																																
21566	A_23_P206899	TMEM159			Y	Y		Y		Y									Y	Y		Y					Y	Y	Y	Y																
21995	A_23_P165171	TMEM161A			Y	Y	Y	Y	Y	Y	Y	Y	Y	Y				Y	Y	Y	Y	Y	Y	Y	Y	Y	Y	Y	Y	Y	Y	Y				Y										
3319	A_23_P156355	TMEM161B			Y	Y	Y	Y	Y	Y	Y	Y	Y	Y				Y	Y	Y	Y	Y	Y	Y	Y	Y	Y	Y	Y	Y	Y	Y	Y			Y										
11788	A_23_P39550	TMEM163	Y		Y																																									
8547	A_23_P351903	TMEM167A	Y	Y	Y																																									
20033	A_23_P156335	TMEM167A			Y	Y	Y	Y	Y	Y	Y	Y	Y	Y					Y	Y		Y					Y	Y	Y	Y						Y										
22343	A_23_P167735	TMEM167A	Y	Y																																										
13936	A_23_P8693	TMEM168																		Y	Y		Y																							
3258	A_23_P100517	TMEM170A			Y	Y		Y	Y	Y																	Y	Y	Y	Y						Y										

18749	A_23_P61371	TMEM173											Y						Y																											
22000	A_23_P344594	TMEM174																		Y			Y				Y																		
4408	A_23_P58148	TMEM175			Y	Y		Y	Y	Y								Y	Y	Y	Y	Y	Y	Y	Y	Y	Y				Y														
11586	A_23_P252082	TMEM176A	Y		Y	Y		Y		Y										Y			Y					Y																	
14523	A_23_P157007	TMEM176B	Y	Y	Y													Y	Y	Y				Y				Y																	
16887	A_23_P142738	TMEM178			Y				Y	Y																	Y			Y						Y										
22007	A_23_P79599	TMEM18																				Y																								
8978	A_23_P337729	TMEM180			Y		Y				Y							Y		Y			Y				Y			Y						Y										
17024	A_23_P202164	TMEM180			Y													Y	Y	Y		Y		Y			Y																			
8926	A_23_P149664	TMEM183B	Y	Y	Y	Y		Y		Y								Y	Y	Y		Y		Y			Y	Y	Y	Y																
4363	A_23_P17870	TMEM184B																												Y						Y										
10755	A_23_P18736	TMEM184C	Y	Y	Y		Y				Y		Y	Y									Y				Y																			
12799	A_23_P255601	TMEM185A																Y		Y																										
13044	A_23_P119788	TMEM185B				Y												Y	Y	Y		Y		Y																						
20846	A_23_P166336	TMEM191A																Y		Y																										
16716	A_23_P136116	TMEM195																									Y			Y						Y										
10146	A_23_P27048	TMEM199			Y				Y	Y																	Y																			
20818	A_23_P94552	TMEM2																	Y																											
16180	A_23_P390032	TMEM20			Y				Y	Y										Y							Y																			
1536	A_23_P391906	TMEM200A													Y																															
3785	A_23_P136238	TMEM203																Y	Y	Y		Y	Y	Y																						
1160	A_23_P37685	TMEM204																		Y																										
11973	A_23_P90099	TMEM205	Y	Y					Y	Y																	Y	Y	Y	Y						Y										
4902	A_23_P717	TMEM206																												Y						Y										
16095	A_23_P206369	TMEM208			Y	Y	Y	Y	Y	Y	Y	Y																Y		Y						Y								Y		
5151	A_23_P143074	TMEM214			Y	Y	Y	Y	Y	Y	Y	Y						Y	Y	Y			Y	Y			Y	Y	Y	Y						Y										
21342	A_23_P203389	TMEM216																Y			Y	Y																								
6141	A_23_P77562	TMEM219				Y																																								
2971	A_23_P109655	TMEM22																					Y							Y						Y										
6899	A_23_P4069	TMEM220																	Y																											
11609	A_23_P97442	TMEM222			Y	Y		Y		Y																	Y																			
15457	A_23_P203115	TMEM25	Y	Y	Y	Y		Y	Y	Y	Y	Y	Y	Y	Y			Y	Y	Y	Y	Y	Y	Y	Y	Y	Y	Y	Y	Y	Y	Y	Y			Y								Y		
9769	A_23_P382236	TMEM26	Y	Y	Y	Y	Y	Y	Y	Y	Y	Y	Y	Y				Y	Y		Y	Y	Y				Y	Y	Y	Y	Y	Y	Y	Y	Y	Y										
13583	A_23_P33984	TMEM27																		Y			Y																							
19374	A_23_P70290	TMEM30A																												Y						Y										
15350	A_23_P213069	TMEM33			Y																																									
4364	A_23_P45324	TMEM35			Y						Y		Y	Y				Y	Y								Y	Y	Y	Y						Y										
21954	A_23_P67799	TMEM37			Y	Y		Y	Y	Y								Y		Y	Y	Y	Y																							
22514	A_23_P101392	TMEM38A																									Y																			
1504	A_23_P60259	TMEM38B	Y	Y	Y										Y			Y		Y	Y	Y	Y				Y	Y	Y	Y																
2115	A_23_P84334	TMEM39A																Y	Y	Y	Y	Y	Y	Y	Y	Y					Y															
21678	A_23_P95571	TMEM39A							Y	Y								Y	Y	Y	Y	Y	Y	Y	Y	Y					Y															
13403	A_23_P34496	TMEM39B																		Y		Y	Y				Y																			
10031	A_23_P29551	TMEM40			Y																							Y																		
3743	A_23_P80831	TMEM41A			Y	Y	Y	Y	Y	Y	Y	Y															Y																			
2688	A_23_P428840	TMEM44			Y	Y		Y	Y	Y																	Y			Y						Y										
356	A_23_P1682	TMEM45B							Y	Y	Y	Y	Y	Y					Y	Y		Y								Y						Y										
4822	A_23_P11175	TMEM47																Y																												
6637	A_23_P129935	TMEM49							Y	Y								Y			Y																									
6021	A_23_P2446	TMEM5			Y	Y		Y	Y	Y	Y	Y	Y	Y				Y	Y	Y	Y			Y			Y																			
15302	A_23_P57304	TMEM50B				Y																																								
5759	A_23_P429627	TMEM52			Y	Y		Y	Y	Y	Y	Y	Y	Y					Y	Y		Y	Y				Y	Y	Y	Y						Y										
17757	A_23_P85853	TMEM53																	Y	Y			Y																							
10702	A_23_P62768	TMEM54																Y																												
21988	A_23_P48610	TMEM55B			Y	Y	Y	Y	Y	Y	Y	Y						Y	Y	Y	Y	Y	Y	Y	Y	Y	Y	Y	Y	Y	Y	Y				Y										
15982	A_23_P170290	TMEM57																												Y						Y										
5487	A_23_P103282	TMEM59			Y															Y			Y				Y																			
18395	A_23_P309155	TMEM59L																				Y	Y																							
6385	A_23_P49041	TMEM62			Y				Y	Y	Y	Y	Y	Y													Y																			
19304	A_23_P200489	TMEM63A			Y	Y		Y	Y	Y												Y					Y																			
3325	A_23_P167856	TMEM63B																	Y	Y			Y																							
9548	A_23_P255812	TMEM66							Y	Y																																				
17889	A_23_P429581	TMEM67			Y	Y		Y		Y										Y							Y																			
21514	A_23_P104151	TMEM69			Y															Y			Y																							
19962	A_23_P253963	TMEM70	Y	Y	Y	Y		Y		Y										Y								Y																		
3351	A_23_P390135	TMEM74																									Y																			
19071	A_23_P74550	TMEM77																Y	Y	Y				Y																						
17645	A_23_P140907	TMEM8			Y	Y	Y	Y	Y	Y	Y	Y						Y	Y	Y	Y	Y	Y	Y	Y	Y	Y	Y	Y	Y	Y	Y	Y	Y	Y	Y										
7778	A_23_P159589	TMEM80																	Y	Y																										
8767	A_23_P332960	TMEM80																		Y		Y																								
1689	A_23_P46690	TMEM81																				Y																								
19749	A_23_P106481	TMEM85			Y																																									
6739	A_23_P108058	TMEM86B																		Y																										
5827	A_23_P88710	TMEM87A							Y	Y	Y	Y	Y	Y					Y	Y		Y																								
1509	A_23_P91076	TMEM87B			Y	Y	Y	Y	Y	Y	Y	Y	Y	Y				Y	Y	Y	Y	Y	Y	Y	Y	Y	Y	Y	Y	Y	Y	Y				Y		Y				Y			Y	
7441	A_23_P303150	TMEM87B			Y				Y	Y	Y	Y	Y	Y						Y			Y				Y			Y						Y										
18532	A_23_P77860	TMEM88							Y	Y																																				

21688	A_23_P114952	TMEM9																	Y			Y																								
1918	A_23_P78134	TMEM93			Y	Y		Y		Y																		Y																		
13766	A_23_P27035	TMEM98							Y	Y	Y	Y	Y	Y				Y	Y	Y				Y			Y			Y						Y										
8583	A_23_P162087	TMEM9B							Y	Y								Y	Y	Y				Y			Y																			
14555	A_23_P143867	TMF1			Y													Y	Y	Y		Y	Y	Y			Y																			
3345	A_23_P401594	TMIE			Y														Y								Y																			
21653	A_23_P56091	TMIGD2				Y														Y			Y					Y																		
7814	A_23_P217546	TMLHE																Y		Y																										
7923	A_23_P112281	TMOD1																					Y																							
1980	A_23_P48803	TMOD2			Y																	Y																								
6936	A_23_P126603	TMOD4			Y	Y		Y		Y								Y			Y	Y																								
181	A_23_P325040	TMPO							Y	Y																																				
1604	A_23_P18748	TMPRSS11E			Y				Y	Y	Y	Y	Y	Y													Y																			
19884	A_23_P203150	TMPRSS13	Y	Y	Y	Y		Y	Y	Y								Y	Y	Y				Y			Y	Y	Y	Y						Y										
16672	A_23_P211273	TMPRSS3																Y	Y																											
17080	A_23_P127608	TMPRSS4																Y										Y		Y						Y										
1950	A_23_P52797	TMPRSS5							Y	Y	Y	Y	Y	Y																Y						Y										
7493	A_23_P332942	TMPRSS6			Y																									Y						Y										
18905	A_23_P69414	TMPRSS7				Y																																								
12751	A_23_P68102	TMSB10							Y	Y																																				
11975	A_23_P171388	TMSB4Y				Y																						Y																		
10025	A_23_P203957	TMTC1			Y																						Y																			
34	A_23_P382705	TMTC2							Y	Y										Y										Y						Y										
10413	A_23_P48264	TMTC3																										Y																		
20238	A_23_P65230	TMTC4			Y				Y	Y	Y	Y	Y	Y																Y						Y										
12178	A_23_P31195	TMUB1			Y	Y		Y		Y								Y	Y	Y		Y	Y	Y			Y																			
10590	A_23_P36647	TMUB2																Y	Y	Y	Y	Y		Y																						
17999	A_23_P157865	TNC																Y									Y																			
2487	A_23_P168071	TNF			Y																							Y		Y						Y										
13078	A_23_P376488	TNF			Y	Y		Y		Y																																				
13444	A_23_P38446	TNFAIP1				Y			Y	Y																	Y	Y	Y	Y						Y										
17376	A_23_P421423	TNFAIP2													Y															Y						Y										
6633	A_23_P259116	TNFAIP8	Y	Y																																										
3671	A_23_P428875	TNFAIP8L1			Y	Y	Y	Y	Y	Y	Y	Y						Y	Y	Y		Y	Y	Y			Y																	Y		
19258	A_23_P46355	TNFAIP8L2	Y		Y																		Y				Y																			
11118	A_23_P255653	TNFRSF10A				Y																																								
10045	A_23_P124625	TNFRSF10B							Y	Y																		Y		Y						Y										
1862	A_23_P95417	TNFRSF10D			Y	Y	Y	Y		Y	Y	Y															Y	Y	Y	Y						Y										
20780	A_23_P390518	TNFRSF11A			Y				Y	Y																				Y						Y										
C11210.6	A_23_P71530	TNFRSF11B			Y														Y	Y							Y			Y						Y										
1978	A_23_P49338	TNFRSF12A			Y	Y		Y	Y	Y	Y	Y	Y	Y				Y		Y		Y						Y																		
7380	A_23_P91764	TNFRSF13C							Y	Y																																				
11835	A_23_P126908	TNFRSF14																Y																												
1367	A_23_P37736	TNFRSF17																									Y																			
16900	A_23_P12215	TNFRSF18				Y													Y	Y		Y																								
3665	A_23_P140057	TNFRSF19	Y		Y													Y									Y	Y	Y	Y																
13527	A_23_P139715	TNFRSF1A																					Y																							
21417	A_23_P96940	TNFRSF1B	Y		Y	Y	Y	Y	Y	Y	Y	Y						Y	Y	Y		Y	Y	Y			Y																			
1899	A_23_P42065	TNFRSF21				Y			Y	Y																				Y						Y										
7093	A_23_P30666	TNFRSF21				Y			Y	Y	Y	Y	Y	Y				Y		Y		Y	Y							Y						Y						Y				
188	A_23_P126844	TNFRSF25																					Y																							
7801	A_23_P112970	TNFRSF4			Y				Y	Y													Y																							
21480	A_23_P218646	TNFRSF6B			Y	Y	Y	Y	Y	Y	Y	Y	Y	Y				Y	Y	Y		Y	Y	Y			Y			Y						Y										
9552	A_23_P500614	TNFRSF8	Y																				Y																							
16755	A_23_P51936	TNFRSF9			Y	Y	Y	Y		Y	Y	Y								Y							Y	Y	Y	Y																
5189	A_23_P121253	TNFSF10																									Y																			
21035	A_23_P99386	TNFSF11			Y																																									
7052	A_23_P141233	TNFSF12			Y	Y	Y	Y		Y	Y	Y						Y	Y	Y		Y	Y	Y			Y	Y	Y	Y																
10022	A_23_P152620	TNFSF13			Y	Y	Y	Y		Y	Y	Y															Y																			
4149	A_23_P14174	TNFSF13B	Y	Y	Y										Y			Y				Y					Y																			
4279	A_23_P94753	TNFSF15			Y													Y	Y	Y				Y			Y			Y						Y										
6765	A_23_P126834	TNFSF4																				Y																								
2342	A_23_P169257	TNFSF8			Y																																									
7864	A_23_P67224	TNFSF9																		Y			Y																							
5774	A_23_P258415	TNIP2			Y	Y		Y	Y	Y								Y	Y	Y	Y	Y	Y	Y	Y	Y	Y	Y	Y	Y	Y	Y				Y										
12461	A_23_P386478	TNIP3	Y	Y	Y	Y		Y	Y	Y																		Y		Y						Y										
15706	A_23_P212997	TNIP3	Y	Y	Y													Y																												
17159	A_23_P107064	TNK1				Y												Y	Y			Y						Y																		
10522	A_23_P259846	TNK2																Y	Y	Y		Y		Y																						
12413	A_23_P324931	TNK2			Y	Y		Y		Y																																				
9138	A_23_P323252	TNKS	Y	Y	Y	Y	Y	Y		Y	Y	Y						Y		Y							Y	Y	Y	Y														Y		
17717	A_23_P418597	TNKS1BP1			Y													Y		Y	Y	Y	Y				Y																			
39	A_23_P24784	TNNI2				Y																					Y																			
6219	A_23_P56050	TNNT1																												Y						Y										
3005	A_23_P127824	TNNT3			Y				Y	Y																	Y																			
13937	A_23_P129382	TNP2																									Y																			
20234	A_23_P326785	TNP2			Y	Y	Y	Y	Y	Y	Y	Y	Y	Y													Y	Y	Y	Y						Y										

1572	A_23_P156101	TNPO1				Y			Y	Y																																			
19692	A_23_P42563	TNPO3																Y				Y																							
10660	A_23_P23561	TNRC4			Y	Y		Y	Y	Y																	Y			Y						Y									
20668	A_23_P337849	TNRC4			Y													Y	Y	Y			Y	Y			Y	Y	Y	Y						Y									
21054	A_23_P349310	TNRC6A			Y	Y		Y		Y																																			
8103	A_23_P392501	TNRC6C			Y	Y		Y	Y	Y								Y		Y							Y	Y	Y	Y						Y									
21672	A_23_P351724	TNS1			Y				Y	Y	Y	Y	Y	Y						Y	Y		Y				Y			Y						Y									
15866	A_23_P156708	TNXB																					Y																						
5687	A_23_P164179	TOB1							Y	Y																				Y						Y									
20375	A_23_P75903	TOLLIP			Y	Y		Y		Y	Y	Y	Y	Y							Y						Y	Y	Y	Y															
3701	A_23_P103082	TOM1			Y	Y	Y	Y	Y	Y	Y	Y	Y	Y				Y	Y	Y	Y	Y	Y	Y	Y	Y	Y				Y														
8692	A_23_P118493	TOM1L1			Y	Y	Y	Y	Y	Y	Y	Y	Y	Y						Y		Y					Y	Y	Y	Y						Y								Y	
437	A_23_P337155	TOM1L2							Y	Y																				Y						Y									
3050	A_23_P141180	TOM1L2	Y	Y					Y	Y	Y	Y	Y	Y																Y						Y									
19975	A_23_P258544	TOMM20			Y																						Y			Y						Y									
3532	A_23_P80336	TOMM22			Y	Y	Y	Y	Y	Y	Y	Y	Y	Y				Y	Y	Y	Y	Y	Y	Y	Y	Y	Y	Y	Y	Y	Y	Y				Y									
18259	A_23_P57033	TOMM34			Y													Y		Y		Y	Y				Y																		
13619	A_23_P153266	TOMM40			Y	Y	Y	Y		Y	Y	Y						Y	Y	Y	Y	Y	Y	Y	Y	Y	Y			Y	Y	Y				Y		Y				Y			Y
22239	A_23_P843	TOMM40L													Y			Y		Y	Y																								
12220	A_23_P93844	TOMM7			Y																						Y																		
8720	A_23_P305507	TOP1							Y	Y	Y	Y	Y	Y																Y						Y									
5258	A_23_P216241	TOP1MT																			Y																								
10548	A_23_P121095	TOP2B				Y														Y																									
20363	A_23_P80213	TOP3B			Y	Y	Y	Y		Y	Y	Y	Y	Y				Y	Y	Y	Y	Y	Y	Y	Y	Y	Y	Y	Y	Y	Y	Y				Y									
13828	A_23_P159390	TOPBP1							Y	Y																																			
17988	A_23_P32217	TOPORS			Y	Y	Y	Y	Y	Y	Y	Y							Y	Y							Y	Y	Y	Y															
803	A_23_P20851	TOR1A			Y	Y		Y		Y								Y		Y		Y					Y	Y	Y	Y						Y									
16463	A_23_P336644	TOR1AIP2																		Y																									
16827	A_23_P60534	TOR2A							Y	Y										Y	Y																								
596	A_23_P115316	TOR3A			Y	Y		Y		Y									Y																										
18252	A_23_P154566	TOX2							Y	Y																				Y						Y									
3591	A_23_P54681	TOX3			Y				Y	Y	Y	Y	Y	Y				Y		Y		Y	Y				Y			Y			Y			Y									
5874	A_23_P404134	TOX4	Y	Y	Y	Y	Y	Y	Y	Y	Y	Y	Y	Y				Y	Y	Y	Y	Y	Y	Y	Y	Y	Y	Y	Y	Y	Y	Y				Y				Y					
11464	A_23_P128847	TOX4			Y															Y			Y				Y						Y												
1330	A_23_P26807	TP53			Y		Y				Y									Y	Y		Y				Y																		
10259	A_23_P88703	TP53BP1			Y	Y	Y	Y	Y	Y	Y	Y	Y	Y						Y							Y	Y	Y	Y															
9011	A_23_P12523	TP53BP2			Y				Y	Y								Y		Y			Y							Y						Y									
1189	A_23_P150281	TP53I11			Y													Y	Y	Y	Y	Y	Y	Y	Y	Y	Y				Y														
10603	A_23_P368028	TP53I11			Y													Y	Y	Y	Y	Y		Y			Y																		
17179	A_23_P27060	TP53I13			Y	Y		Y		Y										Y		Y	Y				Y																		
19275	A_23_P5392	TP53I3																		Y																									
6500	A_23_P168882	TP53INP1				Y			Y	Y	Y	Y	Y	Y				Y	Y			Y								Y						Y									
9738	A_23_P109059	TP53RK																										Y																	
5926	A_23_P145895	TP53TG1							Y	Y	Y	Y	Y	Y				Y		Y	Y	Y								Y						Y						Y			
2010	A_23_P37994	TP53TG3																	Y																										
12646	A_23_P120428	TP53TG5																Y																											
2849	A_23_P327380	TP63																									Y																		
16436	A_23_P59261	TPBG	Y	Y	Y		Y				Y																Y																		
5572	A_23_P98151	TPCN2																		Y																									
2892	A_23_P131764	TPD52L2			Y	Y	Y	Y	Y	Y	Y	Y	Y	Y				Y	Y	Y	Y	Y	Y	Y	Y	Y	Y	Y	Y	Y	Y	Y				Y									
19520	A_23_P20549	TPD52L3				Y													Y																										
4299	A_23_P150595	TPH1				Y																																							
7140	A_23_P316543	TPH2							Y	Y																																			
14553	A_23_P53554	TPI1			Y				Y	Y	Y	Y	Y	Y				Y		Y		Y						Y		Y						Y									
8303	A_23_P145957	TPK1													Y																														
1984	A_23_P216501	TPM2																					Y																						
15201	A_23_P209918	TPO			Y																																								
9830	A_23_P58675	TPPP				Y																																							
8618	A_23_P421621	TPPP2																												Y						Y									
12262	A_23_P117462	TPPP2			Y	Y		Y	Y	Y	Y	Y	Y	Y					Y	Y		Y	Y				Y			Y						Y									
5859	A_23_P45875	TPR			Y	Y		Y		Y																	Y	Y	Y	Y															
16527	A_23_P5757	TPRKB																		Y																									
16183	A_23_P54546	TPSAB1							Y	Y																																			
22060	A_23_P26294	TPSG1			Y	Y		Y		Y										Y		Y	Y				Y																		
8908	A_23_P53797	TPT1			Y	Y	Y	Y	Y	Y	Y	Y	Y	Y													Y	Y	Y	Y						Y								Y	
16011	A_23_P345746	TPTE			Y	Y		Y	Y	Y								Y		Y	Y	Y					Y			Y						Y									
18309	A_23_P68610	TPX2			Y	Y		Y		Y								Y	Y	Y		Y		Y			Y			Y						Y									
972	A_23_P258798	TRA@	Y		Y				Y	Y								Y									Y																		
16792	A_23_P258504	TRA@	Y		Y				Y	Y					Y				Y								Y																		
1975	A_23_P166553	TRABD			Y	Y		Y	Y	Y										Y		Y	Y																						
14008	A_23_P54649	TRADD			Y	Y	Y	Y	Y	Y	Y	Y	Y	Y					Y	Y			Y				Y	Y	Y	Y						Y									
14321	A_23_P216970	TRAF1			Y																																								
7511	A_23_P169327	TRAF2																Y	Y	Y		Y	Y	Y																					
20331	A_23_P5352	TRAF3IP1				Y																						Y																	
5425	A_23_P110879	TRAF3IP2			Y	Y		Y	Y	Y								Y	Y	Y		Y		Y			Y	Y	Y	Y															
11442	A_23_P323761	TRAF3IP3	Y	Y	Y														Y								Y																		
16981	A_23_P201731	TRAF5			Y	Y	Y	Y	Y	Y	Y	Y	Y	Y				Y	Y	Y	Y	Y	Y	Y	Y	Y	Y	Y	Y	Y	Y	Y				Y									

14252	A_23_P75921	TRAF6			Y														Y	Y							Y																			
12151	A_23_P206477	TRAF7			Y	Y		Y		Y								Y	Y	Y		Y		Y			Y																			
7984	A_23_P128536	TRAFD1																		Y																										
8362	A_23_P147723	TRAIP																Y				Y																								
8086	A_23_P132536	TRAK1			Y	Y		Y	Y	Y								Y	Y	Y	Y	Y	Y	Y	Y	Y	Y				Y															
10874	A_23_P209426	TRAK2																Y	Y	Y		Y		Y			Y																			
21423	A_23_P112135	TRAM1	Y	Y	Y																																									
9727	A_23_P372974	TRAM1L1			Y	Y	Y	Y	Y	Y	Y	Y	Y	Y				Y	Y	Y		Y	Y	Y			Y	Y	Y	Y						Y								Y		
20999	A_23_P58937	TRAM2																					Y																							
11318	A_23_P125343	TRAPPC1				Y																																								
21165	A_23_P308147	TRAPPC1			Y	Y		Y		Y	Y	Y	Y	Y																																
4439	A_23_P16035	TRAPPC2																		Y	Y	Y																								
11461	A_23_P73661	TRAPPC2			Y	Y		Y		Y																																				
3631	A_23_P206268	TRAPPC2L				Y																																								
2410	A_23_P200535	TRAPPC3			Y	Y		Y	Y	Y										Y		Y						Y																		
10386	A_23_P127615	TRAPPC4			Y				Y	Y	Y	Y	Y	Y					Y	Y	Y	Y	Y				Y			Y						Y										
18350	A_23_P329202	TRAPPC6A							Y	Y																																				
7409	A_23_P317194	TRAPPC6B																					Y																							
12453	A_23_P151556	TRAPPC6B																					Y																							
2923	A_23_P257684	TRAPPC9																Y		Y																										
20899	A_23_P352861	TRBV5-4																Y												Y						Y										
8939	A_23_P115636	TRDMT1							Y	Y													Y							Y						Y										
2925	A_23_P430753	TREH			Y																	Y					Y																			
11660	A_23_P19333	TREM1				Y			Y	Y	Y	Y	Y	Y																Y						Y										
21152	A_23_P167941	TREM2	Y		Y														Y	Y							Y																			
2006	A_23_P156550	TREML1				Y			Y	Y								Y		Y																										
2589	A_23_P371904	TREML1			Y																																									
14151	A_23_P7932	TREML2	Y																																											
20875	A_23_P59018	TRERF1	Y	Y	Y	Y		Y	Y	Y																	Y	Y	Y	Y						Y										
14051	A_23_P219223	TREX2			Y														Y											Y						Y										
22123	A_23_P132760	TRH			Y																						Y																			
14824	A_23_P322003	TRHR			Y																						Y																			
13947	A_23_P123503	TRIB1				Y																																								
275	A_23_P90689	TRIB2			Y	Y	Y	Y		Y	Y	Y															Y	Y	Y	Y																
9164	A_23_P214578	TRIM10																				Y																								
2424	A_23_P115124	TRIM11																	Y	Y		Y																								
2719	A_23_P99360	TRIM13			Y	Y	Y	Y	Y	Y	Y	Y						Y	Y	Y		Y		Y			Y	Y	Y	Y						Y										
2732	A_23_P87973	TRIM13			Y	Y		Y		Y								Y	Y	Y		Y		Y			Y																			
7583	A_23_P214554	TRIM15																												Y						Y										
9588	A_23_P129903	TRIM16L				Y												Y	Y	Y		Y		Y			Y																			
6433	A_23_P422661	TRIM17																Y		Y	Y		Y																							
3925	A_23_P213141	TRIM2							Y	Y																				Y						Y										
17377	A_23_P203498	TRIM22	Y	Y																																										
6680	A_23_P167384	TRIM23	Y		Y	Y	Y	Y	Y	Y	Y	Y						Y		Y			Y				Y	Y	Y	Y						Y										
17204	A_23_P93629	TRIM24																									Y																			
14703	A_23_P15326	TRIM25	Y	Y	Y		Y		Y	Y	Y	Y	Y	Y					Y	Y							Y			Y						Y										
10141	A_23_P214587	TRIM26			Y													Y	Y	Y	Y	Y	Y	Y	Y	Y	Y				Y															
6819	A_23_P89882	TRIM28			Y													Y	Y	Y		Y	Y	Y			Y																			
1897	A_23_P203267	TRIM29			Y	Y		Y	Y	Y	Y	Y	Y	Y													Y	Y	Y	Y						Y										
6987	A_23_P150619	TRIM3																									Y																			
20351	A_23_P112311	TRIM32							Y	Y																				Y						Y										
18596	A_23_P23526	TRIM33				Y																																								
9958	A_23_P124190	TRIM34			Y																																									
20114	A_23_P502553	TRIM35				Y												Y									Y	Y	Y	Y																
18557	A_23_P93236	TRIM38			Y	Y		Y	Y	Y																		Y																		
18580	A_23_P168048	TRIM39			Y													Y				Y																								
1660	A_23_P255630	TRIM4			Y													Y		Y	Y		Y				Y						Y	Y												
4857	A_23_P500601	TRIM4			Y				Y	Y								Y		Y	Y		Y				Y	Y	Y	Y			Y	Y		Y										
8089	A_23_P402778	TRIM40			Y	Y	Y	Y		Y	Y	Y															Y			Y						Y										
16826	A_23_P167521	TRIM41																Y	Y	Y			Y	Y																						
5679	A_23_P17173	TRIM43			Y																																									
12945	A_23_P139297	TRIM44			Y	Y		Y	Y	Y																	Y	Y	Y	Y																
8330	A_23_P160518	TRIM45	Y	Y	Y	Y	Y	Y	Y	Y	Y	Y	Y	Y				Y	Y	Y	Y	Y	Y	Y	Y	Y	Y	Y	Y	Y	Y	Y	Y	Y	Y	Y										
12252	A_23_P26696	TRIM47	Y	Y	Y	Y		Y	Y	Y	Y	Y	Y	Y					Y	Y		Y	Y				Y	Y	Y	Y						Y										
1061	A_23_P64306	TRIM48																Y			Y																									
7996	A_23_P1575	TRIM49			Y	Y		Y	Y	Y								Y																												
3078	A_23_P203505	TRIM5			Y															Y			Y																							
12213	A_23_P58579	TRIM52	Y	Y	Y	Y	Y	Y	Y	Y	Y	Y	Y	Y				Y	Y	Y		Y	Y	Y			Y	Y	Y	Y	Y	Y	Y	Y	Y	Y										
7943	A_23_P210247	TRIM54			Y															Y			Y																							
5750	A_23_P123402	TRIM55																		Y																										
13050	A_23_P314052	TRIM58			Y	Y		Y	Y	Y																																				
21380	A_23_P407718	TRIM59			Y				Y	Y																		Y		Y						Y										
10404	A_23_P33675	TRIM6				Y													Y	Y							Y	Y	Y	Y						Y										
18835	A_23_P112602	TRIM62							Y	Y	Y	Y	Y	Y																																
2062	A_23_P114983	TRIM63			Y	Y		Y		Y	Y	Y	Y	Y													Y	Y	Y	Y						Y										
3027	A_23_P49489	TRIM65			Y	Y		Y	Y	Y									Y	Y		Y	Y				Y			Y						Y										
18843	A_23_P48826	TRIM69			Y																						Y																			

7557	A_23_P30315	TRIM7				Y																																								
18044	A_23_P46837	TRIM8			Y																						Y																			
18916	A_23_P425880	TRIO																					Y																							
2720	A_23_P17854	TRIOBP			Y													Y	Y	Y		Y	Y	Y																						
15225	A_23_P50349	TRIP10			Y	Y	Y	Y	Y	Y	Y	Y	Y	Y				Y	Y	Y	Y	Y	Y	Y	Y	Y	Y	Y	Y	Y	Y	Y	Y			Y										
12679	A_23_P106241	TRIP11			Y	Y	Y	Y	Y	Y	Y	Y	Y	Y				Y	Y	Y		Y	Y	Y																						
3472	A_23_P147984	TRIP12																		Y																										
19527	A_23_P167607	TRIP13	Y	Y														Y																												
3824	A_23_P252128	TRIP6																			Y																									
12565	A_23_P12062	TRIT1			Y	Y		Y	Y	Y										Y			Y																							
9440	A_23_P16682	TRMT1																Y	Y																											
12784	A_23_P134191	TRMT11			Y	Y		Y	Y	Y										Y		Y	Y							Y						Y										
643	A_23_P120784	TRMT2A			Y															Y			Y																							
13218	A_23_P73604	TRMT2B																Y	Y																											
15207	A_23_P210747	TRMT6							Y	Y										Y																										
593	A_23_P151497	TRMT61A			Y	Y	Y	Y	Y	Y	Y	Y	Y	Y				Y	Y	Y		Y	Y	Y			Y	Y	Y	Y						Y										
16901	A_23_P259823	TRMT61B																		Y			Y																							
12550	A_23_P80389	TRMU			Y	Y	Y	Y	Y	Y	Y	Y						Y		Y		Y					Y	Y	Y	Y						Y										
19488	A_23_P372331	TRNT1																		Y		Y	Y																							
5087	A_23_P217228	TRO							Y	Y																				Y						Y										
21320	A_23_P150935	TROAP																		Y																										
11631	A_23_P94255	TRPA1			Y																						Y																			
5580	A_23_P18046	TRPC1																Y	Y																											
851	A_23_P41455	TRPC3																Y	Y	Y		Y		Y																						
694	A_23_P105873	TRPC4																									Y																			
6238	A_23_P154796	TRPC4AP																Y		Y	Y	Y																								
5741	A_23_P92972	TRPC7																Y		Y	Y	Y																								
14744	A_23_P129225	TRPM1							Y	Y																																				
19722	A_23_P40477	TRPM2				Y														Y							Y	Y	Y	Y																
19078	A_23_P153527	TRPM4							Y	Y																				Y						Y										
11049	A_23_P87279	TRPM5			Y	Y		Y	Y	Y	Y	Y	Y	Y													Y	Y	Y	Y						Y										
16945	A_23_P88470	TRPM7																	Y																											
22387	A_23_P403991	TRPM8							Y	Y								Y																												
15264	A_23_P134755	TRPS1	Y	Y	Y	Y	Y	Y	Y	Y	Y	Y	Y	Y				Y	Y	Y		Y	Y	Y			Y	Y	Y	Y						Y										
11628	A_23_P98244	TRPT1							Y	Y												Y								Y						Y										
960	A_23_P218389	TRPV1			Y				Y	Y										Y							Y																			
4418	A_23_P375281	TRPV1	Y	Y	Y	Y	Y	Y	Y	Y	Y	Y	Y	Y				Y	Y	Y		Y	Y	Y			Y	Y	Y	Y						Y								Y		
6509	A_23_P500244	TRPV1	Y	Y	Y	Y	Y	Y	Y	Y	Y	Y	Y	Y				Y	Y	Y		Y	Y	Y			Y	Y	Y	Y						Y						Y				
11368	A_23_P207911	TRPV2																			Y		Y							Y						Y										
16876	A_23_P139767	TRPV4			Y				Y	Y	Y	Y	Y	Y				Y	Y	Y		Y		Y			Y			Y						Y										
18938	A_23_P93973	TRPV5			Y	Y		Y		Y													Y				Y	Y	Y	Y																
12498	A_23_P71170	TRPV6			Y																						Y																			
19752	A_23_P63908	TRUB1			Y	Y		Y	Y	Y										Y							Y																			
11936	A_23_P82558	TRYX3				Y																						Y																		
11308	A_23_P94600	TSC1				Y																Y						Y																		
21963	A_23_P66110	TSC2																Y	Y	Y		Y		Y																						
14360	A_23_P217688	TSC22D3			Y															Y							Y																			
14918	A_23_P250865	TSC22D4			Y	Y	Y	Y	Y	Y	Y	Y	Y	Y				Y	Y	Y	Y	Y	Y	Y	Y	Y	Y			Y	Y	Y				Y										
14654	A_23_P104022	TSEN15																		Y			Y																							
15318	A_23_P92012	TSEN2			Y	Y	Y	Y	Y	Y	Y	Y						Y		Y		Y	Y				Y	Y	Y	Y																
11070	A_23_P130626	TSEN34				Y												Y	Y	Y				Y																						
14810	A_23_P147277	TSEN54			Y	Y		Y	Y	Y	Y	Y	Y	Y				Y	Y	Y			Y	Y			Y																			
20266	A_23_P64650	TSFM			Y																																									
18276	A_23_P75839	TSG101			Y														Y	Y		Y	Y				Y																			
7281	A_23_P17103	TSGA10							Y	Y	Y	Y	Y	Y				Y	Y	Y		Y		Y			Y	Y	Y	Y																
20269	A_23_P250694	TSGA13			Y						Y		Y	Y																																
2386	A_23_P215078	TSGA14			Y				Y	Y										Y							Y																			
5341	A_23_P12128	TSHB			Y																																									
7104	A_23_P27373	TSHZ1			Y	Y		Y	Y	Y	Y	Y	Y	Y														Y		Y						Y										
20088	A_23_P143247	TSHZ2																		Y			Y																							
21593	A_23_P121986	TSLP																												Y						Y										
11146	A_23_P28105	TSN																Y		Y		Y								Y						Y										
9454	A_23_P319520	TSNARE1																		Y																										
2851	A_23_P51572	TSNAX			Y	Y	Y	Y		Y	Y	Y	Y	Y						Y			Y				Y																			
8111	A_23_P129425	TSNAXIP1				Y																					Y	Y	Y	Y						Y										
12937	A_23_P160167	TSPAN1							Y	Y	Y	Y	Y	Y																Y						Y										
17224	A_23_P9923	TSPAN10			Y	Y		Y	Y	Y										Y		Y	Y																							
14356	A_23_P76136	TSPAN11																					Y							Y						Y										
2887	A_23_P168610	TSPAN13			Y				Y	Y	Y	Y	Y	Y													Y																			
6944	A_23_P202080	TSPAN14			Y													Y	Y	Y	Y	Y	Y	Y	Y	Y	Y			Y	Y	Y				Y										
21525	A_23_P149957	TSPAN15																		Y																										
1273	A_23_P69948	TSPAN17			Y	Y	Y	Y	Y	Y	Y	Y						Y	Y	Y		Y	Y	Y			Y	Y	Y	Y						Y										
22013	A_23_P1655	TSPAN18																		Y			Y				Y																			
19911	A_23_P14804	TSPAN3			Y	Y		Y	Y	Y	Y	Y	Y	Y				Y	Y	Y			Y	Y						Y						Y										
1665	A_23_P392928	TSPAN33	Y	Y	Y	Y		Y	Y	Y	Y	Y	Y	Y					Y	Y	Y	Y	Y				Y	Y	Y	Y						Y										
22092	A_23_P60865	TSPAN4																		Y	Y		Y																							
1824	A_23_P323930	TSPAN5			Y																						Y	Y	Y	Y																

10425	A_23_P61886	TSPAN5			Y	Y		Y		Y																	Y																			
462	A_23_P171143	TSPAN6																		Y																										
19734	A_23_P36528	TSPAN8				Y			Y	Y																		Y																		
17371	A_23_P151127	TSPAN9			Y	Y		Y	Y	Y			Y														Y	Y	Y	Y						Y										
21897	A_23_P162508	TSPAN9			Y	Y		Y		Y										Y							Y																			
10846	A_23_P109571	TSPO			Y	Y	Y	Y	Y	Y	Y	Y	Y	Y				Y		Y		Y					Y	Y	Y	Y						Y										
13394	A_23_P251391	TSPY2	Y	Y	Y										Y												Y																			
22161	A_23_P61702	TSPYL2																Y																												
11458	A_23_P110882	TSPYL4			Y	Y	Y	Y	Y	Y	Y	Y	Y	Y					Y	Y		Y	Y				Y	Y	Y	Y						Y										
22415	A_23_P417951	TSPYL5			Y				Y	Y	Y	Y	Y	Y													Y			Y						Y										
22423	A_23_P91035	TSPYL6							Y	Y																																				
16712	A_23_P414269	TSR2			Y													Y		Y	Y	Y					Y																			
5953	A_23_P29248	TST				Y																								Y						Y										
13052	A_23_P94301	TSTA3			Y	Y		Y	Y	Y									Y	Y		Y					Y			Y						Y										
21970	A_23_P24535	TTC12			Y	Y	Y	Y	Y	Y	Y	Y	Y	Y				Y		Y	Y	Y	Y				Y			Y						Y										
8264	A_23_P103864	TTC13							Y	Y													Y																							
1971	A_23_P212515	TTC14			Y	Y		Y	Y	Y								Y	Y			Y						Y		Y						Y										
20751	A_23_P326931	TTC18			Y	Y	Y	Y	Y	Y	Y	Y	Y	Y				Y	Y	Y		Y	Y	Y			Y	Y	Y	Y						Y										
6481	A_23_P50008	TTC19	Y	Y	Y	Y	Y	Y	Y	Y	Y	Y	Y	Y				Y	Y	Y	Y	Y	Y	Y	Y	Y	Y	Y	Y	Y	Y	Y						Y				Y			Y	Y
12810	A_23_P428248	TTC21A							Y	Y	Y	Y	Y	Y														Y		Y						Y										
1769	A_23_P5422	TTC21B							Y	Y																				Y						Y										
12084	A_23_P73150	TTC25			Y	Y	Y	Y	Y	Y	Y	Y	Y	Y					Y	Y		Y					Y	Y	Y	Y	Y	Y	Y	Y	Y	Y										
18357	A_23_P257668	TTC26			Y				Y	Y	Y	Y	Y	Y						Y		Y	Y				Y				Y		Y	Y	Y											
21737	A_23_P131227	TTC27			Y	Y		Y	Y	Y										Y	Y																									
18610	A_23_P29185	TTC28																	Y	Y		Y																								
7492	A_23_P92517	TTC29																Y									Y																			
2209	A_23_P120703	TTC3				Y			Y	Y	Y	Y	Y	Y						Y								Y		Y						Y										
4898	A_23_P131611	TTC30A			Y						Y		Y	Y					Y	Y	Y	Y	Y				Y																			
19516	A_23_P408913	TTC30B			Y	Y	Y	Y		Y	Y	Y	Y	Y						Y	Y	Y	Y				Y	Y	Y	Y											Y					
2985	A_23_P119857	TTC32																									Y																			
C10627.6	A_23_P61854	TTC37			Y	Y	Y	Y	Y	Y	Y	Y								Y		Y					Y	Y	Y	Y																
19808	A_23_P254198	TTC38			Y	Y		Y	Y	Y										Y		Y	Y				Y	Y	Y	Y																
15722	A_23_P160214	TTC39A							Y	Y	Y	Y	Y	Y																Y						Y										
16698	A_23_P390097	TTC39B																Y									Y																			
9301	A_23_P401098	TTC39C																Y		Y			Y										Y													
21395	A_23_P224	TTC4																			Y																									
16916	A_23_P88280	TTC5																Y	Y	Y	Y	Y		Y																						
7156	A_23_P53987	TTC6			Y	Y		Y	Y	Y	Y	Y	Y	Y									Y				Y	Y	Y	Y						Y										
2277	A_23_P136335	TTC7A			Y	Y	Y	Y	Y	Y	Y	Y	Y	Y				Y	Y	Y	Y	Y	Y	Y	Y	Y	Y	Y	Y	Y	Y	Y	Y	Y	Y	Y										
8571	A_23_P25974	TTC7B	Y						Y	Y			Y		Y															Y						Y										
14135	A_23_P350666	TTC8	Y	Y	Y	Y	Y	Y	Y	Y	Y	Y	Y	Y				Y	Y	Y		Y	Y	Y			Y	Y	Y	Y	Y	Y	Y	Y	Y	Y				Y						
7003	A_23_P254031	TTF1				Y																																								
15046	A_23_P97161	TTF2																		Y																										
18156	A_23_P259586	TTK							Y	Y																																				
16379	A_23_P253208	TTLL13			Y																																									
3118	A_23_P142698	TTLL4			Y				Y	Y	Y	Y	Y	Y				Y																												
8474	A_23_P151756	TTLL5	Y	Y	Y	Y	Y	Y	Y	Y	Y	Y							Y	Y		Y	Y				Y	Y	Y	Y						Y										
12750	A_23_P164222	TTLL6			Y	Y		Y		Y																	Y	Y	Y	Y																
21097	A_23_P332348	TTPA																Y												Y						Y										
8385	A_23_P253561	TTPAL			Y				Y	Y								Y		Y							Y																			
8773	A_23_P130333	TTR	Y	Y	Y		Y				Y		Y	Y													Y			Y						Y										
9299	A_23_P251232	TTTY14			Y	Y		Y	Y	Y	Y	Y	Y	Y														Y		Y						Y										
9828	A_23_P171409	TTTY6																			Y																									
2272	A_23_P45592	TTTY8																									Y			Y						Y										
22328	A_23_P251868	TTTY9A																	Y																											
441	A_23_P50815	TTYH1			Y				Y	Y									Y	Y							Y	Y	Y	Y						Y										
20898	A_23_P66432	TTYH2			Y				Y	Y								Y	Y	Y	Y			Y			Y			Y						Y										
20335	A_23_P500892	TUB							Y	Y																				Y						Y										
21248	A_23_P53110	TUB				Y																						Y																		
19589	A_23_P139547	TUBA1A			Y													Y	Y	Y	Y	Y	Y	Y	Y	Y	Y				Y															
1764	A_23_P430572	TUBA1B			Y															Y																										
5520	A_23_P128155	TUBA1C			Y	Y		Y	Y	Y																		Y		Y						Y										
3495	A_23_P128597	TUBA3C			Y	Y	Y	Y		Y	Y	Y	Y	Y				Y	Y	Y	Y	Y	Y	Y	Y	Y	Y				Y		Y													
11497	A_23_P56736	TUBA3D			Y				Y	Y																	Y			Y						Y										
6624	A_23_P84448	TUBA4A			Y	Y	Y	Y	Y	Y	Y	Y	Y	Y					Y	Y		Y	Y				Y	Y	Y	Y						Y										
9275	A_23_P154068	TUBA4A			Y				Y	Y								Y		Y		Y	Y				Y																			
19077	A_23_P102109	TUBA4A	Y	Y	Y	Y	Y	Y	Y	Y	Y	Y	Y	Y						Y							Y	Y	Y	Y						Y										
1615	A_23_P81914	TUBB			Y				Y	Y								Y	Y	Y	Y	Y	Y	Y	Y	Y	Y			Y	Y	Y	Y			Y										
20288	A_23_P19291	TUBB2A			Y																																									
9134	A_23_P312863	TUBB2C			Y													Y	Y	Y		Y		Y			Y																			
9712	A_23_P77489	TUBB3																	Y			Y	Y																							
6139	A_23_P50344	TUBB4			Y				Y	Y								Y	Y	Y	Y	Y	Y	Y	Y	Y					Y		Y													
14463	A_23_P133165	TUBB4Q																	Y																											
22166	A_23_P254271	TUBB6	Y	Y	Y				Y	Y	Y	Y	Y	Y				Y					Y							Y			Y			Y								Y		
4006	A_23_P26895	TUBD1																									Y	Y	Y	Y																
663	A_23_P145053	TUBE1																		Y																										
13737	A_23_P152768	TUBG1			Y	Y		Y		Y								Y	Y	Y	Y	Y		Y			Y																			

19375	A_23_P78014	TUBG2																Y		Y	Y																									
6594	A_23_P127150	TUBGCP2			Y	Y		Y		Y								Y	Y	Y	Y	Y	Y	Y	Y	Y	Y			Y	Y	Y				Y										
9882	A_23_P152038	TUBGCP5																					Y																							
2943	A_23_P6544	TUBGCP6																					Y																							
9304	A_23_P9582	TUFM			Y	Y		Y		Y								Y	Y	Y	Y	Y	Y	Y	Y	Y	Y				Y															
16257	A_23_P45074	TUFM			Y	Y	Y	Y		Y	Y	Y							Y	Y	Y	Y	Y				Y																			
19605	A_23_P251209	TUFM			Y	Y		Y		Y								Y	Y	Y	Y	Y	Y	Y	Y	Y	Y				Y															
13246	A_23_P160763	TUFT1			Y																																									
19827	A_23_P371824	TUFT1			Y				Y	Y											Y						Y																			
2790	A_23_P320026	TULP1			Y	Y	Y	Y	Y	Y	Y	Y	Y	Y						Y			Y				Y			Y						Y										
7567	A_23_P252227	TULP1			Y	Y	Y	Y		Y	Y	Y							Y				Y				Y																			
16324	A_23_P55957	TULP2																										Y																		
9812	A_23_P116980	TULP3			Y				Y	Y																	Y																			
990	A_23_P156802	TULP4																					Y																							
20459	A_23_P382045	TULP4			Y	Y		Y	Y	Y	Y	Y	Y	Y					Y																									Y		
4618	A_23_P251562	TUSC2	Y	Y	Y	Y		Y	Y	Y	Y	Y	Y	Y				Y		Y							Y	Y	Y	Y						Y										
15745	A_23_P9006	TUSC3							Y	Y	Y	Y	Y	Y					Y	Y										Y						Y										
15781	A_23_P18267	TUSC4	Y	Y					Y	Y																																				
5032	A_23_P53011	TUT1							Y	Y																				Y						Y										
918	A_23_P48166	TWF1				Y														Y								Y		Y						Y										
303	A_23_P121264	TWF2			Y	Y	Y	Y	Y	Y	Y	Y						Y	Y	Y	Y	Y	Y	Y	Y	Y	Y	Y	Y	Y	Y	Y				Y										
22232	A_23_P21324	TWIST2																		Y																										
4624	A_23_P82474	TWISTNB				Y																																								
11501	A_23_P27256	TWSG1							Y	Y																																				
14138	A_23_P51361	TXLNA																		Y		Y																								
7178	A_23_P434398	TXLNB																	Y	Y																										
11781	A_23_P60248	TXN			Y																																									
3234	A_23_P68899	TXN2			Y	Y	Y	Y	Y	Y	Y	Y						Y	Y	Y		Y	Y	Y			Y	Y	Y	Y						Y										
10688	A_23_P50062	TXNDC10			Y	Y		Y	Y	Y									Y	Y		Y						Y																		
20064	A_23_P14935	TXNDC11			Y				Y	Y													Y							Y						Y										
4213	A_23_P200199	TXNDC12			Y	Y		Y		Y																				Y						Y										
10400	A_23_P166100	TXNDC13			Y															Y																										
21823	A_23_P27091	TXNDC17																										Y																		
14292	A_23_P380092	TXNDC4	Y	Y	Y	Y		Y		Y																	Y			Y						Y										
19303	A_23_P168229	TXNDC5			Y				Y	Y									Y								Y																			
6539	A_23_P212672	TXNDC6			Y				Y	Y																	Y																			
2543	A_23_P4522	TXNL1																		Y																										
6386	A_23_P95972	TXNL4B			Y	Y		Y		Y	Y	Y	Y	Y					Y	Y	Y	Y	Y				Y																			
19130	A_23_P102988	TXNRD2			Y													Y	Y	Y		Y		Y			Y																			
6958	A_23_P153311	TYK2																		Y		Y	Y																							
11648	A_23_P91802	TYMP			Y	Y		Y	Y	Y									Y	Y		Y	Y				Y			Y						Y										
8041	A_23_P50096	TYMS																Y		Y	Y	Y																								
7780	A_23_P54517	TYRO3			Y													Y		Y																										
14187	A_23_P27994	TYROBP	Y		Y				Y	Y	Y	Y	Y	Y				Y		Y			Y				Y			Y						Y										
16001	A_23_P94403	TYRP1	Y	Y	Y	Y	Y	Y		Y	Y	Y	Y	Y				Y	Y	Y		Y	Y	Y			Y	Y	Y	Y						Y						Y				
13484	A_23_P423472	TYSND1																												Y						Y										
6061	A_23_P215198	TYW1			Y		Y				Y		Y	Y				Y		Y	Y	Y	Y				Y						Y													
12898	A_23_P103180	TYW3																Y	Y	Y		Y		Y																						
3572	A_23_P6303	U2AF1																		Y																										
17567	A_23_P326296	U2AF1L4			Y	Y	Y	Y	Y	Y	Y	Y	Y	Y				Y	Y								Y	Y	Y	Y																
4062	A_23_P27597	U2AF2			Y														Y	Y							Y																			
7064	A_23_P160460	UAP1																Y		Y																										
8404	A_23_P135164	UAP1L1			Y													Y	Y	Y	Y	Y	Y	Y	Y	Y	Y	Y	Y	Y	Y	Y														
6315	A_23_P137205	UBA1			Y													Y		Y		Y					Y																			
16056	A_23_P211840	UBA3																		Y			Y																							
22448	A_23_P18304	UBA5			Y	Y		Y		Y										Y		Y	Y																							
20939	A_23_P218905	UBA6				Y																																								
12546	A_23_P43994	UBA7			Y	Y		Y		Y								Y				Y					Y			Y						Y										
16992	A_23_P60324	UBAC1			Y	Y		Y		Y										Y	Y	Y	Y				Y																			
13179	A_23_P139998	UBAC2			Y	Y		Y		Y									Y	Y		Y	Y																							
22312	A_23_P123866	UBAP1																	Y								Y																			
19636	A_23_P6293	UBASH3A			Y													Y	Y	Y				Y			Y																			
16097	A_23_P203273	UBASH3B				Y														Y	Y	Y	Y																							
17238	A_23_P329740	UBC			Y				Y	Y	Y	Y	Y	Y														Y		Y						Y										
1366	A_23_P81898	UBD							Y	Y																	Y																			
11057	A_23_P167589	UBE2B																									Y																			
C10623.3	A_23_P143207	UBE2C			Y													Y		Y		Y					Y	Y	Y	Y						Y										
12306	A_23_P46855	UBE2D1			Y																																									
12275	A_23_P58622	UBE2D2				Y																Y																								
21302	A_23_P305677	UBE2D2			Y	Y		Y	Y	Y									Y	Y																										
18344	A_23_P58294	UBE2D3			Y																						Y			Y						Y										
237	A_23_P374767	UBE2D4							Y	Y																																				
582	A_23_P59752	UBE2D4			Y	Y		Y	Y	Y	Y	Y	Y	Y													Y	Y	Y	Y						Y										
8779	A_23_P367071	UBE2DNL																												Y						Y										
21726	A_23_P10807	UBE2E2	Y	Y	Y	Y		Y	Y	Y	Y	Y	Y	Y				Y		Y			Y				Y	Y	Y	Y						Y										
10585	A_23_P11192	UBE2E3			Y				Y	Y	Y	Y	Y	Y								Y	Y				Y	Y	Y	Y						Y										
14677	A_23_P27066	UBE2G1																Y		Y			Y																							

6394	A_23_P211179	UBE2G2			Y														Y	Y	Y	Y		Y	Y	Y																							
11826	A_23_P215096	UBE2H																	Y	Y			Y																								
11425	A_23_P152107	UBE2I			Y																Y		Y																									
3019	A_23_P214187	UBE2J1	Y	Y	Y	Y		Y	Y	Y									Y								Y	Y	Y	Y															
9144	A_23_P217881	UBE2J2			Y	Y	Y	Y		Y	Y	Y						Y	Y	Y		Y	Y	Y			Y																		
9132	A_23_P166353	UBE2L3							Y	Y																																			
9450	A_23_P75741	UBE2L6			Y		Y				Y		Y													Y																		
9516	A_23_P141853	UBE2M			Y	Y		Y		Y						Y	Y	Y	Y	Y	Y	Y	Y	Y	Y				Y														
6093	A_23_P86034	UBE2Q1																Y	Y	Y		Y		Y																					
11740	A_23_P55769	UBE2S			Y	Y		Y	Y	Y	Y	Y	Y	Y				Y	Y	Y	Y	Y	Y	Y	Y	Y	Y			Y	Y	Y				Y				Y					
93	A_23_P379661	UBE2U			Y	Y		Y		Y																		Y																	
12866	A_23_P48790	UBE3A			Y	Y		Y	Y	Y	Y	Y	Y	Y				Y	Y	Y		Y		Y			Y	Y	Y	Y						Y									
11143	A_23_P99183	UBE3B			Y	Y	Y	Y	Y	Y	Y	Y	Y	Y				Y	Y	Y				Y			Y				Y		Y	Y	Y										
4860	A_23_P59481	UBE3C			Y													Y			Y	Y				Y																		
10181	A_23_P203139	UBE4A			Y		Y				Y									Y		Y	Y																							
6107	A_23_P140029	UBL3	Y	Y					Y	Y																	Y	Y	Y	Y						Y									
2673	A_23_P62361	UBL4A			Y	Y		Y		Y									Y	Y	Y	Y	Y				Y																		
21984	A_23_P335988	UBL4A			Y	Y	Y	Y		Y	Y	Y							Y	Y		Y	Y				Y																		
11977	A_23_P117777	UBL7			Y	Y	Y	Y	Y	Y	Y	Y						Y	Y	Y	Y	Y	Y	Y	Y	Y	Y				Y		Y												
14885	A_23_P400344	UBLCP1				Y																					Y																		
21072	A_23_P218717	UBOX5			Y	Y		Y	Y	Y	Y	Y	Y	Y						Y							Y	Y	Y	Y						Y									
22217	A_23_P250904	UBQLN1																		Y		Y	Y																						
6852	A_23_P98830	UBQLN3			Y	Y		Y	Y	Y										Y		Y	Y				Y			Y						Y									
3473	A_23_P201051	UBQLN4			Y	Y		Y		Y								Y	Y	Y		Y	Y	Y			Y																		
9689	A_23_P328621	UBQLNL	Y		Y	Y		Y	Y	Y	Y	Y	Y	Y					Y	Y		Y					Y	Y	Y	Y						Y									
11828	A_23_P152066	UBR1			Y	Y		Y	Y	Y	Y	Y	Y	Y				Y	Y	Y		Y	Y	Y			Y	Y	Y	Y						Y									
12465	A_23_P362636	UBR2																		Y		Y																							
14304	A_23_P5405	UBR3																		Y	Y		Y																						
21519	A_23_P86164	UBR4																		Y																									
18181	A_23_P8825	UBR5																Y	Y	Y		Y	Y	Y																					
14168	A_23_P161501	UBTD1			Y	Y	Y	Y	Y	Y	Y	Y	Y	Y				Y		Y	Y	Y	Y				Y			Y						Y									
12739	A_23_P19061	UBTD2			Y	Y		Y		Y										Y																									
16777	A_23_P98605	UBXN1																				Y	Y																						
10208	A_23_P85666	UBXN10			Y	Y		Y	Y	Y	Y	Y	Y	Y				Y	Y	Y		Y	Y	Y			Y	Y	Y	Y						Y							Y		
10783	A_23_P131240	UBXN2A							Y	Y										Y																									
3659	A_23_P409512	UBXN4				Y	Y				Y							Y	Y	Y	Y	Y	Y	Y	Y	Y	Y					Y														
1457	A_23_P386905	UBXN6			Y	Y	Y	Y	Y	Y	Y	Y								Y		Y	Y				Y			Y						Y									
4475	A_23_P16443	UBXN6																Y																											
21379	A_23_P157465	UBXN8			Y																																								
19256	A_23_P132956	UCHL1							Y	Y	Y	Y	Y	Y	Y			Y	Y	Y		Y	Y	Y						Y						Y									
14036	A_23_P416036	UCHL5IP			Y													Y	Y	Y				Y																					
7477	A_23_P216402	UCK1			Y													Y	Y	Y		Y	Y	Y			Y																		
21427	A_23_P487	UCK2	Y	Y	Y	Y		Y	Y	Y	Y	Y	Y	Y				Y	Y	Y		Y		Y			Y	Y	Y	Y						Y									
16267	A_23_P17326	UCKL1			Y														Y	Y	Y																								
1647	A_23_P131695	UCN	Y	Y	Y				Y	Y	Y	Y	Y	Y						Y		Y					Y			Y						Y									
9094	A_23_P155453	UCN2				Y			Y	Y													Y																						
10820	A_23_P30091	UCP1				Y																					Y																	
13507	A_23_P47704	UCP2	Y		Y				Y	Y								Y		Y		Y					Y			Y						Y									
1778	A_23_P139347	UCP3																													Y						Y									
18502	A_23_P203445	UEVLD				Y	Y				Y									Y	Y		Y	Y				Y	Y	Y	Y						Y									
3476	A_23_P166333	UFD1L			Y		Y				Y																Y																		
7982	A_23_P370080	UFM1																Y																											
17872	A_23_P41437	UFSP2				Y												Y	Y	Y			Y	Y																						
7423	A_23_P313380	UGCG			Y	Y		Y	Y	Y								Y		Y							Y	Y	Y	Y						Y									
2170	A_23_P204980	UGCGL2			Y	Y	Y	Y	Y	Y	Y	Y	Y	Y				Y	Y	Y		Y		Y			Y																		
10956	A_23_P167067	UGDH							Y	Y			Y																Y						Y									
11227	A_23_P253046	UGP2																									Y																		
12302	A_23_P60599	UGT1A6																													Y						Y									
8164	A_23_P213279	UGT2A3			Y																Y					Y																		
22286	A_23_P7342	UGT2B10																Y		Y																									
13229	A_23_P212968	UGT2B11																Y																											
4656	A_23_P386911	UGT2B4																Y																											
21190	A_23_P136671	UGT2B7																Y		Y	Y																								
11966	A_23_P61346	UGT8							Y	Y																				Y						Y									
17803	A_23_P72747	UGT8							Y	Y			Y					Y	Y			Y								Y						Y									
198	A_23_P34657	UHMK1																Y																											
3393	A_23_P145370	UHRF1BP1			Y													Y	Y	Y		Y	Y	Y			Y			Y						Y									
8686	A_23_P9145	UHRF2							Y	Y													Y																						
9461	A_23_P254712	UIMC1			Y	Y		Y		Y								Y	Y	Y		Y		Y			Y																		
15326	A_23_P156873	ULBP1			Y	Y		Y		Y									Y	Y		Y	Y				Y	Y	Y	Y						Y									
7024	A_23_P168259	ULBP2			Y	Y		Y	Y	Y								Y										Y		Y						Y									
18038	A_23_P145485	ULBP2			Y	Y		Y	Y	Y								Y	Y							Y	Y	Y	Y						Y									
21640	A_23_P134100	ULBP3			Y	Y		Y		Y								Y									Y	Y	Y	Y						Y									
12119	A_23_P72551	ULK1			Y																						Y			Y						Y									
18711	A_23_P113148	ULK1																		Y																									
21893	A_23_P55107	ULK2			Y	Y	Y	Y	Y	Y	Y	Y	Y	Y					Y	Y	Y	Y						Y	Y	Y	Y						Y									
20724	A_23_P206103	ULK3																	Y																										

7183	A_23_P158775	UMOD							Y	Y	Y	Y	Y	Y																															
7871	A_23_P6624	UMPS																		Y			Y																						
1746	A_23_P49842	UNC119			Y													Y	Y	Y		Y		Y																					
4223	A_23_P87827	UNC119B			Y	Y		Y	Y	Y								Y																											
12943	A_23_P253395	UNC13B			Y																						Y			Y						Y										
21859	A_23_P88576	UNC13C			Y	Y		Y	Y	Y										Y			Y				Y																			
12448	A_23_P163903	UNC13D	Y		Y	Y		Y	Y	Y	Y	Y	Y	Y						Y			Y				Y	Y	Y	Y						Y										
14565	A_23_P218170	UNC45A																Y		Y		Y																								
22366	A_23_P351254	UNC45A																										Y																		
4184	A_23_P49677	UNC45B			Y																						Y																			
10967	A_23_P252700	UNC50																					Y																							
665	A_23_P110557	UNC5A			Y	Y		Y	Y	Y																	Y			Y						Y										
12174	A_23_P69617	UNC5C			Y	Y		Y		Y								Y	Y			Y					Y																			
8922	A_23_P428298	UNC5CL			Y	Y		Y	Y	Y											Y						Y																			
17398	A_23_P93190	UNC5CL				Y																								Y						Y										
10671	A_23_P216118	UNC5D																									Y																			
22020	A_23_P214402	UNC93A																									Y																			
7181	A_23_P98164	UNC93B1			Y	Y	Y	Y	Y	Y	Y	Y	Y	Y				Y	Y	Y	Y	Y	Y	Y	Y	Y	Y			Y	Y	Y				Y										
346	A_23_P100141	UNKL			Y																						Y																			
15283	A_23_P162559	UNQ1887																	Y		Y																									
8251	A_23_P106913	UNQ3118			Y	Y		Y		Y																	Y	Y	Y	Y																
18390	A_23_P64280	UNQ565			Y	Y		Y		Y										Y		Y						Y																		
22543	A_23_P61386	UNQ9391				Y			Y	Y																	Y	Y	Y	Y						Y										
15519	A_23_P120822	UPB1							Y	Y																																				
520	A_23_P27977	UPF1			Y	Y	Y	Y		Y	Y	Y						Y	Y	Y	Y	Y	Y	Y	Y	Y	Y				Y															
9364	A_23_P99473	UPF3A			Y	Y		Y	Y	Y																	Y			Y						Y										
226	A_23_P45409	UPF3B				Y			Y	Y																																				
2560	A_23_P121293	UPK1B			Y	Y		Y		Y									Y									Y																		
12254	A_23_P109584	UPK3A				Y												Y	Y	Y	Y	Y	Y	Y	Y	Y	Y				Y		Y	Y	Y											
10870	A_23_P351275	UPP1	Y	Y	Y	Y	Y	Y	Y	Y	Y	Y	Y	Y						Y	Y		Y				Y	Y	Y	Y	Y	Y	Y	Y	Y	Y				Y						
15585	A_23_P71989	UPP1							Y	Y																				Y						Y										
20529	A_23_P147805	UPP1				Y			Y	Y	Y	Y	Y	Y						Y			Y					Y																		
3830	A_23_P210947	UQCC																		Y		Y	Y																							
16579	A_23_P56314	UQCR							Y	Y								Y		Y		Y																								
20586	A_23_P132675	UQCRC1			Y	Y		Y		Y								Y	Y	Y	Y	Y		Y			Y																			
9434	A_23_P118002	UQCRC2			Y																						Y																			
8462	A_23_P153586	UQCRFS1			Y	Y		Y		Y																																				
18278	A_23_P213718	UQCRQ							Y	Y																																				
5082	A_23_P57293	URB1																	Y																											
10268	A_23_P425790	URG4			Y	Y		Y		Y								Y		Y	Y	Y	Y																							
7397	A_23_P35609	UROS			Y															Y																										
6012	A_23_P73128	USHBP1																		Y			Y																							
16957	A_23_P61732	USHBP1																Y	Y																											
14809	A_23_P171366	USP11																Y		Y	Y	Y																								
7538	A_23_P204269	USP15																										Y																		
3109	A_23_P257911	USP16				Y														Y								Y																		
21837	A_23_P258860	USP17																									Y																			
19703	A_23_P132159	USP18			Y													Y									Y																			
13995	A_23_P217028	USP20			Y				Y	Y										Y			Y																							
5188	A_23_P73992	USP24				Y														Y																										
20572	A_23_P256676	USP25																		Y																										
18721	A_23_P159695	USP27X			Y															Y			Y																							
13156	A_23_P206612	USP31																Y		Y																										
1351	A_23_P107154	USP32			Y																						Y			Y						Y										
12786	A_23_P106	USP33																		Y							Y																			
10346	A_23_P28676	USP34				Y																						Y		Y						Y										
4271	A_23_P116602	USP35			Y	Y	Y	Y	Y	Y	Y	Y										Y					Y	Y	Y	Y																
12386	A_23_P67918	USP37				Y			Y	Y																																				
15894	A_23_P44734	USP38			Y	Y		Y	Y	Y										Y							Y	Y	Y	Y																
17167	A_23_P154476	USP40			Y														Y	Y		Y	Y				Y																			
6120	A_23_P82394	USP42			Y	Y		Y		Y																		Y																		
21714	A_23_P152696	USP43																					Y																							
22164	A_23_P72770	USP44			Y				Y	Y								Y									Y																			
11526	A_23_P134008	USP45			Y	Y		Y	Y	Y										Y			Y																							
9565	A_23_P81180	USP46			Y	Y	Y	Y	Y	Y	Y	Y	Y	Y													Y	Y	Y	Y						Y										
7042	A_23_P148204	USP47			Y	Y		Y	Y	Y									Y	Y								Y																		
12792	A_23_P159195	USP47			Y	Y		Y	Y	Y									Y	Y		Y						Y																		
15932	A_23_P200551	USP48			Y	Y	Y	Y		Y	Y	Y								Y	Y		Y				Y																			
17082	A_23_P64954	USP5			Y	Y	Y	Y		Y	Y	Y						Y	Y	Y	Y	Y		Y			Y															Y				
1332	A_23_P355536	USP54							Y	Y										Y	Y	Y																								
1631	A_23_P63769	USP54																									Y																			
8759	A_23_P207666	USP6			Y	Y		Y		Y																	Y																			
5252	A_23_P138426	USP6NL																												Y						Y										
21604	A_23_P49481	USP7			Y																																									
12117	A_23_P129053	USP8			Y	Y		Y	Y	Y	Y	Y	Y	Y					Y	Y							Y	Y	Y	Y						Y										
4895	A_23_P217409	USP9X			Y	Y		Y		Y									Y	Y		Y						Y																		
14098	A_23_P137228	USP9Y																Y		Y		Y																								

5936	A_23_P128635	USPL1			Y	Y		Y		Y													Y				Y																			
21697	A_23_P314145	UST							Y	Y								Y	Y	Y				Y				Y																	
15271	A_23_P213441	UTP15	Y	Y	Y	Y		Y	Y	Y	Y	Y	Y	Y					Y	Y							Y	Y	Y	Y						Y										
20579	A_23_P162525	UTP20			Y																																									
1949	A_23_P92424	UTP3			Y	Y		Y		Y								Y		Y							Y																			
6444	A_23_P214772	UTRN				Y															Y																									
13083	A_23_P318840	UTRN			Y	Y		Y		Y									Y	Y		Y					Y	Y	Y	Y						Y										
19373	A_23_P146885	UTS2R			Y	Y		Y	Y	Y										Y		Y	Y				Y																			
1193	A_23_P217304	UTX																		Y		Y	Y																							
18424	A_23_P67829	UXS1							Y	Y																				Y						Y										
5306	A_23_P66190	VAC14			Y	Y		Y		Y								Y	Y	Y		Y		Y			Y			Y						Y										
6652	A_23_P77590	VAC14			Y	Y	Y	Y	Y	Y	Y	Y	Y	Y				Y	Y				Y				Y	Y	Y	Y						Y										
12472	A_23_P164341	VAMP2				Y														Y			Y																							
10915	A_23_P61569	VAMP4																					Y																							
14257	A_23_P52046	VAMP4																		Y			Y																							
13939	A_23_P39837	VAMP5	Y	Y	Y	Y	Y	Y	Y	Y	Y	Y	Y	Y													Y	Y	Y	Y																
5869	A_23_P28434	VAMP8							Y	Y																																				
19640	A_23_P369316	VANGL1			Y																						Y																			
7608	A_23_P207957	VAPA			Y	Y	Y	Y	Y	Y	Y	Y	Y	Y						Y							Y			Y						Y										
379	A_23_P258714	VARS			Y	Y	Y	Y	Y	Y	Y	Y						Y	Y	Y	Y	Y	Y	Y	Y	Y	Y				Y															
16644	A_23_P111112	VARS2				Y																																								
7487	A_23_P76998	VASH1																					Y																							
7412	A_23_P129695	VASN			Y	Y		Y	Y	Y								Y	Y	Y	Y	Y	Y	Y	Y	Y	Y	Y	Y	Y	Y	Y	Y			Y		Y								
18948	A_23_P119102	VASP			Y															Y	Y	Y	Y																							
2370	A_23_P400449	VAT1L	Y	Y	Y																						Y			Y						Y										
15293	A_23_P38959	VAV1	Y		Y	Y	Y	Y	Y	Y	Y	Y	Y	Y	Y				Y	Y			Y				Y																			
14530	A_23_P146468	VAV2																Y	Y	Y	Y	Y		Y																						
7234	A_23_P201551	VAV3			Y	Y		Y	Y	Y	Y	Y	Y	Y				Y	Y	Y			Y	Y			Y	Y	Y	Y						Y										
6724	A_23_P104541	VAX1	Y	Y					Y	Y																																				
5946	A_23_P34345	VCAM1	Y	Y																																										
14671	A_23_P144959	VCAN				Y													Y			Y						Y																		
22200	A_23_P202283	VCL																Y		Y			Y																							
13264	A_23_P83045	VCP									Y		Y	Y																																
4705	A_23_P256445	VCPIP1							Y	Y																																				
21876	A_23_P252572	VCX3B			Y								Y																																	
8242	A_23_P11390	VCY			Y						Y		Y	Y																Y						Y										
2392	A_23_P60069	VDAC3			Y																																									
1135	A_23_P387668	VEGFA			Y				Y	Y								Y		Y		Y																								
15367	A_23_P81805	VEGFA							Y	Y										Y																										
6242	A_23_P380208	VEPH1																									Y																			
17143	A_23_P404064	VEZF1																										Y																		
18401	A_23_P206981	VEZF1			Y															Y							Y			Y						Y										
19696	A_23_P204608	VEZT			Y	Y	Y	Y	Y	Y	Y	Y						Y	Y	Y		Y	Y	Y			Y	Y	Y	Y																
16284	A_23_P352905	VGF			Y	Y		Y		Y								Y	Y	Y		Y		Y			Y	Y	Y	Y																
10329	A_23_P253123	VGLL1			Y	Y		Y	Y	Y	Y	Y	Y	Y														Y		Y						Y										
4852	A_23_P410709	VGLL2																												Y						Y										
20236	A_23_P132763	VGLL3																	Y																											
18747	A_23_P132595	VGLL4				Y			Y	Y																				Y						Y										
17563	A_23_P63096	VHLL							Y	Y																																				
21850	A_23_P95770	VIPR1																									Y			Y						Y										
22075	A_23_P212016	VIPR1			Y													Y					Y				Y	Y	Y	Y																
2853	A_23_P145978	VIPR2							Y	Y										Y		Y	Y																							
13233	A_23_P68539	VISA				Y			Y	Y																																				
18796	A_23_P100486	VKORC1			Y													Y		Y	Y	Y	Y				Y																			
2618	A_23_P413815	VKORC1L1			Y	Y		Y	Y	Y									Y								Y	Y	Y	Y						Y										
14799	A_23_P43476	VLDLR																	Y				Y																							
22290	A_23_P96542	VMA21	Y	Y	Y	Y		Y	Y	Y	Y	Y	Y	Y						Y							Y	Y	Y	Y						Y								Y		
6349	A_23_P55356	VMO1				Y																																								
9362	A_23_P78493	VN1R1			Y	Y		Y		Y																																				
4576	A_23_P402279	VN1R2			Y													Y																												
7168	A_23_P78656	VN1R4	Y		Y																						Y	Y	Y	Y																
12358	A_23_P255345	VNN1			Y																						Y																			
20684	A_23_P122724	VNN2			Y										Y												Y																			
17677	A_23_P212595	VPRBP	Y	Y	Y	Y		Y		Y																	Y	Y	Y	Y						Y										
22424	A_23_P166371	VPREB3			Y	Y		Y		Y										Y			Y				Y			Y						Y										
13418	A_23_P150403	VPS11																		Y																										
3041	A_23_P217098	VPS13A			Y	Y	Y	Y	Y	Y	Y	Y	Y	Y				Y	Y	Y		Y	Y	Y			Y	Y	Y	Y												Y				
20099	A_23_P82969	VPS13B																									Y			Y						Y										
19602	A_23_P206228	VPS13C			Y	Y		Y	Y	Y			Y							Y							Y	Y	Y	Y						Y										
19772	A_23_P251216	VPS13D			Y	Y		Y		Y										Y		Y						Y																		
12979	A_23_P374732	VPS18																					Y																							
13387	A_23_P206194	VPS18			Y															Y	Y	Y	Y																							
372	A_23_P66599	VPS25				Y			Y	Y								Y		Y	Y	Y																								
20633	A_23_P75257	VPS26A			Y																																									
13795	A_23_P203323	VPS26B																		Y		Y																								
6664	A_23_P128388	VPS29							Y	Y																																				
5062	A_23_P366328	VPS37A			Y														Y																											

11720	A_23_P150876	VPS37B			Y				Y	Y								Y		Y							Y			Y						Y										
899	A_23_P44228	VPS37C			Y				Y	Y																																				
18185	A_23_P47517	VPS37C			Y				Y	Y																																				
10889	A_23_P215318	VPS41			Y	Y		Y	Y	Y										Y								Y		Y						Y										
9020	A_23_P23719	VPS45			Y	Y		Y		Y																																				
8321	A_23_P329772	VPS4A			Y	Y	Y	Y	Y	Y	Y	Y							Y	Y		Y	Y				Y																			
17345	A_23_P70583	VPS52																Y																												
7433	A_23_P131737	VPS54			Y	Y		Y	Y	Y										Y		Y	Y																							
5384	A_23_P115215	VPS72																Y	Y			Y					Y																			
3798	A_23_P119992	VRK2																		Y			Y																							
3893	A_23_P107933	VRK3			Y				Y	Y								Y	Y	Y		Y		Y																						
3433	A_23_P36018	VSIG2			Y																																									
21335	A_23_P217269	VSIG4	Y	Y	Y	Y		Y		Y	Y	Y	Y	Y					Y	Y							Y	Y	Y	Y						Y										
4477	A_23_P55836	VSTM1				Y																					Y	Y	Y	Y																
19540	A_23_P157025	VSTM2A			Y																																									
22064	A_23_P79807	VSTM2L			Y																						Y																			
2007	A_23_P14284	VTI1B																									Y																			
6994	A_23_P86089	VWA1			Y	Y		Y		Y																																				
2080	A_23_P129397	VWA3A																												Y						Y										
4630	A_23_P423989	VWA3B																									Y																			
18463	A_23_P98458	VWA5A			Y	Y		Y		Y									Y	Y		Y	Y																							
20093	A_23_P80867	VWA5B2																					Y																							
17822	A_23_P157338	VWC2																Y	Y	Y		Y		Y																						
14072	A_23_P52986	VWCE																					Y																							
14131	A_23_P105562	VWF																					Y																							
4065	A_23_P65651	WARS	Y	Y	Y	Y	Y	Y	Y	Y	Y	Y	Y	Y					Y	Y	Y		Y				Y	Y	Y	Y						Y								Y		
2041	A_23_P74380	WARS2			Y	Y		Y	Y	Y										Y	Y		Y				Y						Y													
12412	A_23_P96331	WAS			Y	Y		Y	Y	Y																	Y	Y	Y	Y						Y										
4607	A_23_P168306	WASF1									Y		Y	Y																																
13684	A_23_P201309	WASF2																				Y																								
2616	A_23_P11742	WASH2P			Y	Y		Y		Y																				Y						Y										
20055	A_23_P134609	WASL																									Y																			
1027	A_23_P252808	WBP1							Y	Y																																				
13408	A_23_P105519	WBP11			Y	Y		Y		Y																																				
1775	A_23_P3900	WBP2			Y													Y	Y	Y				Y			Y																			
8589	A_23_P410128	WBP2NL	Y	Y		Y																Y								Y						Y										
17560	A_23_P140043	WBP4			Y	Y		Y		Y								Y	Y	Y		Y		Y																						
7131	A_23_P351804	WBSCR16				Y																						Y																		
17109	A_23_P169656	WBSCR16																Y	Y	Y		Y	Y	Y																						
16833	A_23_P425963	WBSCR19			Y	Y		Y		Y																		Y																		
3157	A_23_P71014	WBSCR22			Y	Y		Y		Y								Y	Y	Y		Y		Y			Y	Y	Y	Y																
14107	A_23_P123018	WBSCR23			Y	Y		Y	Y	Y																		Y																		
22062	A_23_P381016	WBSCR27																Y	Y																											
10058	A_23_P56390	WDFY1																		Y			Y																							
10310	A_23_P502797	WDFY1																										Y																		
21502	A_23_P218108	WDFY2			Y	Y		Y	Y	Y																	Y			Y						Y										
17587	A_23_P213001	WDR1	Y		Y	Y		Y		Y								Y		Y								Y																		
17229	A_23_P136916	WDR13			Y	Y	Y	Y	Y	Y	Y	Y								Y	Y	Y	Y				Y																			
447	A_23_P5115	WDR18			Y	Y		Y	Y	Y								Y	Y	Y		Y	Y	Y			Y			Y						Y										
702	A_23_P205256	WDR20							Y	Y																																				
5320	A_23_P26476	WDR24			Y	Y		Y	Y	Y								Y	Y	Y		Y	Y	Y																						
18225	A_23_P84202	WDR25																Y	Y	Y		Y	Y	Y																						
8848	A_23_P85604	WDR26			Y	Y	Y	Y	Y	Y	Y	Y	Y	Y				Y	Y	Y		Y		Y			Y																			
9647	A_23_P217059	WDR32				Y			Y	Y													Y					Y																		
4950	A_23_P209788	WDR33				Y												Y	Y	Y		Y	Y	Y																						
7980	A_23_P414420	WDR33							Y	Y									Y				Y																							
13281	A_23_P20823	WDR34																		Y																										
21687	A_23_P108463	WDR35			Y	Y	Y	Y	Y	Y	Y	Y						Y	Y	Y		Y	Y	Y			Y	Y	Y	Y																
12843	A_23_P110598	WDR36			Y																						Y	Y	Y	Y																
978	A_23_P211302	WDR4			Y	Y		Y		Y									Y								Y																			
7767	A_23_P255260	WDR40A																Y																												
16620	A_23_P11095	WDR40B																	Y																											
11045	A_23_P259521	WDR41																										Y																		
9914	A_23_P200146	WDR42A							Y	Y																		Y																		
7856	A_23_P251716	WDR45				Y			Y	Y										Y							Y	Y	Y	Y																
13099	A_23_P207026	WDR45L			Y	Y		Y		Y																	Y																			
1991	A_23_P23748	WDR47																		Y																										
12028	A_23_P32558	WDR5			Y	Y		Y	Y	Y									Y	Y							Y	Y	Y	Y						Y										
8711	A_23_P212284	WDR51A			Y	Y		Y		Y								Y	Y								Y																			
17548	A_23_P105535	WDR51B			Y	Y		Y		Y								Y	Y	Y		Y	Y	Y			Y	Y	Y	Y																
1993	A_23_P403869	WDR52			Y	Y	Y	Y	Y	Y	Y	Y	Y	Y						Y			Y				Y	Y	Y	Y						Y										
1619	A_23_P68068	WDR54			Y	Y	Y	Y	Y	Y	Y	Y	Y	Y													Y	Y	Y	Y						Y										
4130	A_23_P121855	WDR55							Y	Y																	Y			Y						Y										
11083	A_23_P206856	WDR59							Y	Y																																				
5063	A_23_P95553	WDR6	Y	Y	Y	Y		Y	Y	Y	Y	Y	Y	Y													Y	Y	Y	Y						Y										
18112	A_23_P83671	WDR6	Y	Y	Y	Y		Y	Y	Y	Y	Y	Y	Y													Y	Y	Y	Y						Y										
19543	A_23_P157361	WDR60			Y															Y			Y				Y			Y						Y										

9704	A_23_P49015	WDR61			Y	Y	Y	Y	Y	Y	Y	Y															Y																			
13226	A_23_P339705	WDR62			Y	Y		Y		Y								Y	Y	Y	Y	Y		Y																						
5967	A_23_P399116	WDR64																Y																												
5931	A_23_P363275	WDR66			Y						Y		Y	Y													Y	Y	Y	Y						Y										
9782	A_23_P26836	WDR68																	Y	Y			Y																							
17516	A_23_P422268	WDR68			Y	Y	Y	Y	Y	Y	Y	Y	Y	Y					Y	Y	Y	Y	Y				Y	Y	Y	Y						Y										
22508	A_23_P28466	WDR69																	Y																											
1419	A_23_P164528	WDR7			Y	Y	Y	Y	Y	Y	Y	Y						Y	Y	Y		Y	Y	Y			Y	Y	Y	Y	Y	Y	Y	Y	Y											
589	A_23_P30474	WDR70																			Y		Y				Y																			
3246	A_23_P99917	WDR73	Y		Y	Y		Y	Y	Y	Y	Y	Y	Y						Y		Y	Y				Y			Y						Y										
10680	A_23_P36157	WDR74			Y	Y	Y	Y		Y	Y	Y						Y	Y	Y		Y	Y	Y			Y	Y	Y	Y						Y										
14623	A_23_P427718	WDR75				Y			Y	Y										Y		Y						Y																		
19131	A_23_P129075	WDR76																Y	Y								Y																			
15195	A_23_P115149	WDR77																									Y																			
21060	A_23_P200670	WDR78																		Y										Y						Y										
1601	A_23_P109877	WDR82																	Y	Y	Y		Y																							
3481	A_23_P163047	WDR89				Y			Y	Y								Y	Y	Y		Y		Y			Y																			
20750	A_23_P411833	WDR90			Y	Y		Y	Y	Y	Y	Y	Y	Y													Y	Y	Y	Y						Y										
16743	A_23_P348867	WDR92																Y																												
19880	A_23_P108657	WDSUB1																					Y																							
11836	A_23_P86054	WDTC1	Y	Y	Y																																									
2068	A_23_P127926	WEE1	Y		Y	Y	Y	Y	Y	Y	Y	Y						Y	Y	Y		Y	Y	Y			Y	Y	Y	Y																
10288	A_23_P106611	WFDC1			Y				Y	Y																	Y																			
8505	A_23_P500472	WFDC10B			Y	Y		Y		Y												Y					Y	Y	Y	Y																
12145	A_23_P68436	WFDC12			Y														Y	Y		Y	Y				Y																			
19671	A_23_P218675	WFDC2			Y	Y		Y		Y			Y		Y						Y						Y	Y	Y	Y						Y										
19727	A_23_P120435	WFDC3			Y	Y		Y		Y																		Y																		
17801	A_23_P102634	WFDC6																			Y																									
9106	A_23_P121499	WFS1							Y	Y	Y	Y	Y	Y													Y			Y						Y										
2770	A_23_P213205	WHSC1			Y	Y		Y		Y	Y	Y	Y	Y						Y							Y																			
20934	A_23_P7301	WHSC1			Y	Y		Y		Y																	Y	Y	Y	Y																
2396	A_23_P330612	WIPF1	Y	Y	Y	Y	Y	Y	Y	Y	Y	Y	Y	Y					Y	Y			Y				Y																			
13946	A_23_P251436	WIPF1			Y																						Y																			
5871	A_23_P366454	WIPF2			Y	Y		Y	Y	Y	Y	Y	Y	Y													Y	Y	Y	Y						Y										
17538	A_23_P136323	WIPF2																			Y																									
10922	A_23_P141394	WIPI1							Y	Y																																				
21809	A_23_P354694	WISP1													Y																															
954	A_23_P102611	WISP2																												Y						Y										
12674	A_23_P81676	WISP3																									Y																			
11691	A_23_P337689	WIT1																Y																												
1047	A_23_P419661	WNK1			Y				Y	Y																	Y																			
3786	A_23_P250701	WNK1			Y																						Y																			
1380	A_23_P219139	WNK2																				Y																								
22384	A_23_P410473	WNK2				Y																Y																								
12691	A_23_P324482	WNK3																Y	Y	Y				Y			Y																			
22447	A_23_P38334	WNK4	Y	Y	Y	Y	Y	Y	Y	Y	Y	Y	Y	Y													Y	Y	Y	Y						Y										
4925	A_23_P411157	WNT1	Y																																											
3932	A_23_P102113	WNT10A			Y	Y	Y	Y	Y	Y	Y	Y							Y				Y																							
3931	A_23_P36337	WNT11							Y	Y																																				
7605	A_23_P134601	WNT16																										Y																		
41	A_23_P59813	WNT2													Y																															
2289	A_23_P130158	WNT3			Y				Y	Y	Y	Y	Y	Y													Y																			
9701	A_23_P23468	WNT3A			Y	Y		Y	Y	Y													Y																							
9842	A_23_P385695	WNT3A			Y	Y		Y		Y								Y	Y	Y		Y	Y	Y			Y																			
14375	A_23_P11787	WNT4	Y		Y	Y		Y	Y	Y												Y	Y				Y																			
18441	A_23_P211926	WNT5A	Y																Y	Y	Y		Y				Y																			
16083	A_23_P53588	WNT5B			Y				Y	Y	Y	Y	Y	Y									Y																							
19332	A_23_P119916	WNT6			Y	Y		Y	Y	Y	Y	Y	Y	Y						Y							Y			Y						Y										
8263	A_23_P320054	WNT7B																Y																												
16156	A_23_P136155	WNT7B			Y																																									
17527	A_23_P58854	WNT8A			Y																																									
8119	A_23_P378329	WNT9A				Y			Y	Y								Y	Y	Y		Y		Y				Y																		
8709	A_23_P89588	WNT9B																										Y		Y						Y										
9511	A_23_P80122	WRB			Y																		Y																							
C8627.3	A_23_P250813	WRN				Y														Y								Y																		
1300	A_23_P414296	WRNIP1							Y	Y																																				
1408	A_23_P4353	WSB1				Y																					Y	Y	Y	Y																
11978	A_23_P147238	WSB2			Y													Y	Y	Y	Y	Y	Y	Y	Y	Y	Y				Y															
8995	A_23_P34176	WWC3							Y	Y									Y																											
10523	A_23_P345650	WWC3																									Y																			
14524	A_23_P83368	WWOX				Y																																								
11917	A_23_P146990	WWP1			Y	Y		Y	Y	Y	Y	Y	Y	Y				Y	Y	Y		Y	Y	Y			Y	Y	Y	Y						Y										
19890	A_23_P206680	WWP2			Y	Y	Y	Y	Y	Y	Y	Y	Y	Y					Y	Y		Y	Y				Y	Y	Y	Y																
22390	A_23_P357147	WWP2			Y				Y	Y	Y	Y	Y	Y				Y	Y	Y	Y	Y	Y	Y	Y	Y	Y			Y	Y	Y				Y										
1392	A_23_P29763	WWTR1							Y	Y																				Y						Y										
9939	A_23_P142013	XAB2				Y																																								
8626	A_23_P114343	XAGE3																									Y																			

2746	A_23_P301497	XAGE5			Y															Y	Y	Y	Y				Y																			
4488	A_23_P200752	XCL1			Y	Y		Y	Y	Y									Y	Y		Y	Y				Y	Y	Y	Y						Y										
9153	A_23_P51532	XCL2	Y																																											
20419	A_23_P321431	XCL2	Y	Y	Y		Y				Y							Y		Y							Y	Y	Y	Y						Y										
17390	A_23_P72487	XG	Y	Y	Y				Y	Y																	Y																			
5928	A_23_P22459	XIAP																												Y						Y										
5970	A_23_P108746	XIRP2																			Y																									
17936	A_23_P45304	XK	Y	Y					Y	Y								Y									Y																			
1969	A_23_P420431	XKR3	Y		Y													Y									Y																			
21729	A_23_P34183	XKRY2							Y	Y																	Y			Y						Y										
2889	A_23_P60283	XPA			Y	Y	Y	Y	Y	Y	Y	Y	Y	Y				Y	Y	Y		Y	Y	Y			Y	Y	Y	Y						Y										
3731	A_23_P92008	XPC																Y		Y	Y	Y																								
9623	A_23_P40078	XPO1				Y																																								
7944	A_23_P63534	XPR1																Y	Y																											
209	A_23_P153687	XRCC1			Y																																									
6312	A_23_P48470	XRCC3																	Y																											
1804	A_23_P79738	XRCC5			Y	Y		Y	Y	Y										Y							Y	Y	Y	Y						Y										
6031	A_23_P120950	XRCC6			Y	Y	Y	Y		Y	Y	Y								Y		Y						Y																		
845	A_23_P53363	XRCC6BP1				Y																																								
1448	A_23_P132820	XRN1																				Y																								
1977	A_23_P349321	XYLT1																Y				Y																								
6988	A_23_P15582	XYLT2			Y																						Y			Y						Y										
16762	A_23_P104762	YAP1			Y																																									
13912	A_23_P85570	YARS			Y	Y		Y		Y										Y		Y	Y				Y			Y						Y										
20446	A_23_P379546	YARS			Y	Y	Y	Y	Y	Y	Y	Y						Y	Y	Y	Y	Y	Y	Y	Y	Y	Y	Y	Y	Y	Y	Y	Y			Y										
4742	A_23_P34766	YBX1	Y	Y	Y										Y			Y	Y	Y	Y	Y	Y	Y	Y	Y	Y			Y	Y	Y				Y		Y								
12576	A_23_P49865	YBX2			Y	Y		Y	Y	Y								Y	Y	Y				Y			Y	Y	Y	Y																
16121	A_23_P69437	YEATS2							Y	Y																				Y						Y										
4640	A_23_P87591	YEATS4			Y	Y		Y		Y										Y			Y																							
15419	A_23_P47226	YIF1A			Y																																									
11992	A_23_P153625	YIF1B			Y																						Y																			
178	A_23_P200239	YIPF1	Y	Y					Y	Y											Y									Y						Y										
6682	A_23_P107894	YIPF2			Y	Y	Y	Y	Y	Y	Y	Y								Y							Y	Y	Y	Y																
20867	A_23_P18806	YIPF5			Y																																									
2808	A_23_P431319	YIPF6				Y																	Y				Y																			
19809	A_23_P134527	YKT6	Y	Y	Y	Y		Y	Y	Y	Y	Y	Y	Y					Y	Y							Y	Y	Y	Y						Y										
14535	A_23_P3128	YLPM1			Y	Y		Y	Y	Y	Y	Y	Y	Y													Y	Y	Y	Y						Y						Y				
21454	A_23_P104201	YME1L1	Y	Y																																										
15388	A_23_P359699	YPEL1																Y		Y																										
18969	A_23_P40548	YPEL1			Y																																									
11932	A_23_P15104	YPEL3																					Y																							
3358	A_23_P300220	YPEL4																									Y																			
959	A_23_P108835	YPEL5																	Y																											
17200	A_23_P41732	YTHDC2			Y	Y	Y	Y	Y	Y	Y	Y						Y	Y	Y		Y	Y	Y			Y	Y	Y	Y																
12112	A_23_P5912	YTHDF1			Y	Y		Y		Y																	Y																			
9543	A_23_P103414	YTHDF2				Y														Y	Y		Y																							
3344	A_23_P103070	YWHAH			Y	Y		Y	Y	Y								Y		Y		Y	Y				Y																			
17089	A_23_P131723	YWHAQ	Y	Y																																										
6415	A_23_P103662	YY1AP1			Y	Y		Y		Y																	Y	Y	Y	Y																
4968	A_23_P316064	ZACN							Y	Y									Y																											
6037	A_23_P375476	ZADH2			Y										Y																															
18599	A_23_P38774	ZADH2			Y	Y	Y	Y	Y	Y	Y	Y								Y							Y																			
10882	A_23_P210180	ZAK	Y		Y	Y	Y	Y	Y	Y	Y	Y						Y		Y			Y				Y	Y	Y	Y						Y										
10693	A_23_P322619	ZAN			Y	Y		Y	Y	Y																	Y	Y	Y	Y						Y										
C12233.8	A_23_P39682	ZAP70																	Y																											
11162	A_23_P217508	ZBED1																		Y	Y	Y	Y																							
1954	A_23_P113793	ZBED2	Y	Y	Y				Y	Y																	Y																			
18189	A_23_P155148	ZBED4			Y	Y		Y		Y																	Y	Y	Y	Y						Y										
8143	A_23_P98483	ZBED5			Y	Y	Y	Y	Y	Y	Y	Y	Y	Y				Y	Y	Y	Y	Y	Y	Y	Y	Y	Y	Y	Y	Y	Y	Y														
3285	A_23_P259141	ZBP1			Y				Y	Y			Y					Y	Y	Y				Y			Y						Y	Y												
12984	A_23_P99699	ZBTB1			Y	Y	Y	Y		Y	Y	Y						Y	Y	Y		Y		Y																						
2801	A_23_P385114	ZBTB10			Y																																									
285	A_23_P109774	ZBTB11																		Y		Y					Y																			
2106	A_23_P654	ZBTB17																		Y																										
268	A_23_P82108	ZBTB2				Y			Y	Y																				Y						Y										
12247	A_23_P40866	ZBTB20			Y				Y	Y									Y	Y		Y					Y			Y						Y										
567	A_23_P111188	ZBTB22			Y	Y		Y		Y																	Y	Y	Y	Y																
17710	A_23_P134147	ZBTB24				Y															Y																									
15319	A_23_P48626	ZBTB25			Y	Y	Y	Y		Y	Y	Y						Y	Y	Y		Y	Y	Y			Y	Y	Y	Y																
13160	A_23_P71827	ZBTB26																					Y					Y																		
1373	A_23_P116400	ZBTB3				Y			Y	Y											Y									Y						Y										
19817	A_23_P430181	ZBTB3	Y	Y	Y																																									
18657	A_23_P131028	ZBTB32	Y	Y	Y		Y		Y	Y	Y	Y								Y							Y			Y						Y								Y		
16882	A_23_P96483	ZBTB33																	Y																											
355	A_23_P137504	ZBTB37																												Y						Y										
12995	A_23_P212025	ZBTB38																	Y																											
21694	A_23_P380076	ZBTB38			Y															Y		Y	Y				Y																			

17826	A_23_P100653	ZBTB4	Y	Y	Y		Y		Y	Y	Y	Y						Y	Y	Y		Y		Y			Y			Y						Y										
14936	A_23_P160136	ZBTB40			Y				Y	Y	Y	Y	Y	Y													Y			Y						Y								Y		
17266	A_23_P11533	ZBTB40																			Y																									
19220	A_23_P387523	ZBTB40	Y	Y	Y	Y	Y	Y	Y	Y	Y	Y	Y	Y				Y	Y	Y		Y	Y	Y			Y	Y	Y	Y	Y	Y	Y	Y	Y	Y										
13339	A_23_P54019	ZBTB42			Y																						Y			Y						Y										
19363	A_23_P135315	ZBTB43				Y																						Y		Y						Y										
10440	A_23_P202945	ZBTB44																		Y			Y																							
17020	A_23_P149506	ZBTB48																		Y																										
15221	A_23_P216479	ZBTB5			Y				Y	Y									Y	Y	Y	Y	Y							Y						Y										
3795	A_23_P27827	ZBTB7A																Y		Y	Y	Y	Y																							
2595	A_23_P11729	ZBTB7B			Y	Y		Y	Y	Y																	Y	Y	Y	Y																
9931	A_23_P352365	ZBTB8			Y	Y	Y	Y	Y	Y	Y	Y	Y	Y				Y		Y			Y				Y	Y	Y	Y						Y										
14233	A_23_P58967	ZC3H11A	Y	Y	Y	Y	Y	Y	Y	Y	Y	Y	Y	Y				Y	Y	Y	Y	Y	Y	Y	Y	Y	Y	Y	Y	Y	Y	Y				Y										
18753	A_23_P204836	ZC3H13																	Y																											
11620	A_23_P54230	ZC3H14				Y			Y	Y																		Y		Y						Y										
8245	A_23_P3502	ZC3H18			Y	Y	Y	Y		Y	Y	Y						Y	Y	Y	Y	Y	Y	Y	Y	Y	Y				Y															
2984	A_23_P9036	ZC3H3			Y	Y	Y	Y	Y	Y	Y	Y						Y	Y	Y		Y	Y	Y			Y	Y	Y	Y						Y								Y		
13866	A_23_P101580	ZC3H4				Y																																								
12725	A_23_P26336	ZC3H7A			Y	Y	Y	Y	Y	Y	Y	Y	Y	Y				Y		Y	Y	Y	Y				Y	Y	Y	Y			Y			Y										
11268	A_23_P108871	ZC3H8				Y														Y		Y																								
18289	A_23_P31532	ZC3HAV1L																									Y																			
22218	A_23_P62188	ZC4H2																									Y			Y						Y										
12594	A_23_P34433	ZCCHC11																		Y		Y																								
10127	A_23_P100420	ZCCHC14			Y																																									
4026	A_23_P66958	ZCCHC2			Y																																									
10952	A_23_P55564	ZCCHC2																		Y										Y						Y										
12938	A_23_P335449	ZCCHC24			Y	Y		Y	Y	Y																	Y	Y	Y	Y						Y										
14842	A_23_P210686	ZCCHC3																				Y																								
13191	A_23_P123727	ZCCHC6																	Y	Y		Y	Y																							
9344	A_23_P124855	ZCCHC7			Y														Y									Y																		
22319	A_23_P64669	ZCCHC8			Y	Y	Y	Y	Y	Y	Y	Y						Y	Y	Y	Y	Y	Y	Y	Y	Y	Y	Y	Y	Y	Y	Y														
11897	A_23_P151221	ZCRB1			Y	Y		Y	Y	Y																	Y	Y	Y	Y						Y										
19958	A_23_P66038	ZDHHC1																										Y																		
21557	A_23_P256008	ZDHHC11	Y	Y	Y				Y	Y	Y	Y	Y	Y				Y	Y	Y		Y	Y	Y			Y	Y	Y	Y	Y	Y	Y	Y	Y	Y										
5722	A_23_P158007	ZDHHC12			Y	Y	Y	Y	Y	Y	Y	Y	Y	Y				Y	Y	Y	Y	Y	Y	Y	Y	Y	Y			Y	Y	Y				Y										
12142	A_23_P250619	ZDHHC14																					Y				Y	Y	Y	Y																
22549	A_23_P314191	ZDHHC17			Y	Y	Y	Y	Y	Y	Y	Y							Y	Y		Y					Y	Y	Y	Y																
19337	A_23_P51851	ZDHHC18			Y	Y		Y	Y	Y	Y	Y	Y	Y				Y		Y		Y	Y				Y			Y						Y										
20520	A_23_P9086	ZDHHC2																			Y							Y		Y						Y										
16409	A_23_P99397	ZDHHC20			Y	Y		Y		Y								Y		Y		Y					Y	Y	Y	Y																
17277	A_23_P302125	ZDHHC21			Y	Y	Y	Y	Y	Y	Y	Y	Y	Y																																
5066	A_23_P350689	ZDHHC23																		Y																										
14011	A_23_P212734	ZDHHC3			Y				Y	Y								Y	Y	Y		Y	Y	Y			Y																			
10806	A_23_P98532	ZDHHC5																												Y						Y										
17582	A_23_P161177	ZDHHC6			Y	Y		Y	Y	Y										Y								Y																		
17118	A_23_P129389	ZDHHC7			Y	Y		Y		Y			Y							Y	Y	Y	Y				Y																			
2515	A_23_P148057	ZDHHC9			Y	Y		Y		Y								Y									Y																			
12488	A_23_P202010	ZEB1				Y																						Y		Y						Y										
16889	A_23_P142560	ZEB2																												Y						Y										
22515	A_23_P146631	ZER1			Y				Y	Y								Y	Y	Y	Y	Y	Y	Y	Y	Y	Y			Y	Y	Y				Y				Y						
2165	A_23_P336992	ZFAND2A			Y	Y		Y		Y																		Y																		
11393	A_23_P131299	ZFAND2B			Y	Y	Y	Y	Y	Y	Y	Y						Y	Y	Y		Y	Y	Y			Y	Y	Y	Y						Y										
8635	A_23_P122375	ZFAND3																			Y																									
7275	A_23_P71752	ZFAND5																Y	Y	Y		Y	Y	Y																						
10985	A_23_P140605	ZFAND6																									Y			Y						Y										
12820	A_23_P150950	ZFC3H1			Y	Y		Y	Y	Y								Y	Y									Y																		
21328	A_23_P332908	ZFC3H1																										Y		Y						Y										
3449	A_23_P118266	ZFHX3			Y	Y		Y	Y	Y	Y	Y	Y	Y					Y								Y	Y	Y	Y						Y										
19757	A_23_P43095	ZFHX4			Y	Y		Y	Y	Y									Y									Y		Y						Y										
15990	A_23_P3856	ZFP1			Y				Y	Y									Y	Y		Y					Y																			
4814	A_23_P77310	ZFP106			Y	Y	Y	Y	Y	Y	Y	Y	Y	Y				Y		Y		Y					Y	Y	Y	Y						Y										
7164	A_23_P107724	ZFP112			Y	Y	Y	Y	Y	Y	Y	Y	Y	Y				Y	Y	Y	Y	Y	Y	Y	Y	Y	Y	Y	Y	Y	Y	Y				Y		Y				Y			Y	Y
10925	A_23_P107673	ZFP28																Y																												
8146	A_23_P99540	ZFP36L1																									Y	Y	Y	Y						Y										
17604	A_23_P254025	ZFP37							Y	Y																		Y		Y						Y										
661	A_23_P69877	ZFP62			Y	Y	Y	Y	Y	Y	Y	Y	Y	Y					Y	Y		Y	Y				Y	Y	Y	Y																
20224	A_23_P120467	ZFP64																		Y																										
19500	A_23_P416813	ZFP82							Y	Y																				Y						Y										
6745	A_23_P203346	ZFP91				Y																																								
17129	A_23_P98232	ZFPL1			Y	Y	Y	Y	Y	Y	Y	Y	Y	Y				Y	Y	Y	Y	Y	Y	Y	Y	Y	Y	Y	Y	Y	Y	Y	Y			Y				Y						
14974	A_23_P54575	ZFPM1																		Y		Y																								
15418	A_23_P168909	ZFPM2				Y													Y																											
20563	A_23_P41818	ZFR																					Y																							
995	A_23_P125639	ZFX			Y														Y	Y			Y																							
4106	A_23_P62465	ZFY	Y	Y	Y	Y		Y		Y	Y	Y	Y	Y				Y	Y	Y		Y	Y	Y			Y				Y		Y	Y	Y											
15693	A_23_P128956	ZFYVE1			Y														Y								Y																			
7818	A_23_P7543	ZFYVE16																	Y	Y		Y						Y																		

19576	A_23_P65930	ZFYVE19							Y	Y																																		
12312	A_23_P315872	ZFYVE20			Y																																							
18851	A_23_P14273	ZFYVE21							Y	Y																	Y																			
6313	A_23_P323924	ZFYVE27	Y	Y	Y	Y		Y	Y	Y								Y		Y							Y	Y	Y	Y																
13926	A_23_P45163	ZFYVE9																Y	Y	Y				Y			Y																			
6350	A_23_P345666	ZGPAT			Y															Y	Y	Y	Y				Y																			
11203	A_23_P218654	ZGPAT																	Y																											
19840	A_23_P166563	ZIC1																				Y																								
7777	A_23_P327910	ZIC3			Y																						Y																			
423	A_23_P211711	ZIC4																										Y																		
13969	A_23_P53826	ZIC5				Y																																								
6519	A_23_P94067	ZKSCAN1			Y	Y		Y	Y	Y																		Y		Y						Y										
3974	A_23_P168019	ZKSCAN3			Y	Y		Y		Y																																				
11015	A_23_P133868	ZKSCAN4			Y				Y	Y												Y					Y			Y						Y										
11661	A_23_P259661	ZKSCAN5			Y	Y		Y	Y	Y									Y	Y		Y	Y				Y																			
10437	A_23_P125749	ZMAT1				Y			Y	Y	Y	Y	Y	Y																Y						Y										
19428	A_23_P346301	ZMAT2																		Y		Y					Y			Y						Y										
18305	A_23_P113283	ZMAT3																			Y																									
22346	A_23_P44474	ZMAT4	Y		Y														Y								Y			Y						Y										
197	A_23_P132248	ZMAT5			Y	Y	Y	Y	Y	Y	Y	Y	Y	Y				Y	Y	Y	Y	Y		Y			Y	Y	Y	Y						Y										
12629	A_23_P138435	ZMIZ1			Y	Y	Y	Y	Y	Y	Y	Y						Y	Y	Y		Y		Y			Y	Y	Y	Y						Y										
18033	A_23_P111745	ZMIZ2			Y	Y		Y		Y																																				
15334	A_23_P161091	ZMYM1				Y														Y																										
1062	A_23_P99404	ZMYM2	Y		Y	Y	Y	Y	Y	Y	Y	Y	Y	Y				Y		Y	Y		Y				Y	Y	Y	Y						Y										
5772	A_23_P137073	ZMYM3																Y		Y		Y																								
7861	A_23_P104145	ZMYM4			Y	Y	Y	Y	Y	Y	Y	Y	Y	Y					Y	Y			Y				Y	Y	Y	Y						Y										
11037	A_23_P65208	ZMYM5																					Y																							
18754	A_23_P201841	ZMYM6				Y														Y								Y		Y						Y										
18912	A_23_P29663	ZMYND10	Y	Y	Y	Y	Y	Y	Y	Y	Y	Y	Y	Y													Y	Y	Y	Y			Y			Y										
21421	A_23_P89570	ZMYND15				Y																																								
15873	A_23_P305173	ZMYND19			Y	Y		Y		Y								Y	Y	Y		Y	Y	Y			Y	Y	Y	Y						Y										
18809	A_23_P166000	ZMYND8																		Y		Y																								
14038	A_23_P203829	ZNF10			Y	Y	Y	Y	Y	Y	Y	Y	Y	Y				Y	Y	Y		Y	Y	Y			Y	Y	Y	Y						Y										
16351	A_23_P45087	ZNF107			Y	Y		Y	Y	Y	Y	Y	Y	Y				Y	Y	Y		Y	Y	Y			Y	Y	Y	Y	Y	Y	Y	Y	Y	Y										
19286	A_23_P79107	ZNF107				Y			Y	Y										Y		Y	Y					Y		Y						Y										
12925	A_23_P215806	ZNF117				Y			Y	Y									Y	Y		Y	Y					Y		Y						Y										
9205	A_23_P31333	ZNF12			Y	Y	Y	Y	Y	Y	Y	Y							Y	Y							Y	Y	Y	Y						Y										
6602	A_23_P89963	ZNF121			Y																																									
14529	A_23_P52176	ZNF124			Y																						Y																			
4928	A_23_P133315	ZNF131																		Y			Y																							
16597	A_23_P154730	ZNF133			Y	Y		Y		Y																																				
5792	A_23_P325661	ZNF134			Y	Y	Y	Y		Y	Y	Y						Y	Y	Y		Y		Y			Y																			
8498	A_23_P141866	ZNF134																										Y																		
13714	A_23_P348227	ZNF135			Y	Y	Y	Y	Y	Y	Y	Y															Y	Y	Y	Y						Y										
8361	A_23_P67312	ZNF136	Y	Y	Y	Y		Y	Y	Y								Y	Y	Y		Y		Y			Y	Y	Y	Y						Y										
21249	A_23_P147115	ZNF136			Y	Y		Y	Y	Y										Y			Y				Y																			
19440	A_23_P208238	ZNF137				Y	Y		Y	Y	Y	Y								Y		Y						Y		Y						Y							Y			
14373	A_23_P59855	ZNF138							Y	Y										Y	Y	Y	Y					Y																		
19048	A_23_P101811	ZNF14			Y	Y		Y	Y	Y										Y								Y																		
15240	A_23_P150841	ZNF140																		Y																										
17969	A_23_P81207	ZNF141			Y	Y		Y	Y	Y										Y			Y				Y	Y	Y	Y						Y										
21160	A_23_P388867	ZNF141							Y	Y										Y		Y																								
14641	A_23_P203564	ZNF143			Y	Y		Y		Y									Y	Y		Y																								
18086	A_23_P108022	ZNF146																	Y	Y							Y																			
21721	A_23_P139408	ZNF148			Y	Y		Y	Y	Y									Y	Y		Y	Y				Y	Y	Y	Y																
7408	A_23_P101716	ZNF155			Y	Y	Y	Y	Y	Y	Y	Y	Y	Y						Y							Y	Y	Y	Y						Y										
16865	A_23_P168864	ZNF16																Y																												
18675	A_23_P396804	ZNF160			Y	Y	Y	Y	Y	Y	Y	Y	Y	Y				Y	Y	Y		Y	Y	Y			Y	Y	Y	Y						Y										
16465	A_23_P253921	ZNF167			Y				Y	Y																	Y			Y						Y										
1742	A_23_P49327	ZNF174																									Y																			
10355	A_23_P15983	ZNF175																		Y																										
15695	A_23_P164706	ZNF177	Y		Y	Y		Y	Y	Y	Y	Y	Y	Y	Y			Y	Y	Y	Y	Y	Y	Y	Y	Y	Y	Y	Y	Y	Y	Y				Y				Y						
2773	A_23_P141302	ZNF18	Y	Y	Y	Y		Y	Y	Y								Y	Y	Y		Y	Y	Y			Y				Y		Y	Y	Y											
18099	A_23_P89919	ZNF180			Y	Y	Y	Y	Y	Y	Y	Y	Y	Y				Y		Y		Y					Y	Y	Y	Y						Y										
12838	A_23_P50735	ZNF181				Y			Y	Y										Y																										
15919	A_23_P156615	ZNF184			Y															Y		Y																								
14646	A_23_P11025	ZNF185																			Y	Y																								
3567	A_23_P145247	ZNF187			Y	Y	Y	Y	Y	Y	Y	Y								Y		Y	Y				Y	Y	Y	Y																
52	A_23_P216869	ZNF189																		Y																										
6502	A_23_P500900	ZNF19																		Y	Y		Y																							
4840	A_23_P122464	ZNF193			Y	Y	Y	Y	Y	Y	Y	Y	Y	Y													Y	Y	Y	Y						Y										
C11617.2	A_23_P252748	ZNF195			Y	Y		Y	Y	Y								Y	Y	Y		Y	Y	Y			Y	Y	Y	Y						Y										
7761	A_23_P69055	ZNF197			Y	Y	Y	Y	Y	Y	Y	Y	Y	Y						Y							Y	Y	Y	Y						Y										
20313	A_23_P16242	ZNF20				Y													Y	Y		Y	Y					Y																		
13051	A_23_P152356	ZNF200																		Y																										
8475	A_23_P127652	ZNF202				Y														Y			Y																							
2730	A_23_P3748	ZNF205																		Y			Y																							

18639	A_23_P165242	ZNF208			Y	Y		Y	Y	Y										Y		Y	Y					Y																			
10946	A_23_P130474	ZNF211			Y	Y	Y	Y	Y	Y	Y	Y	Y	Y				Y	Y	Y		Y	Y	Y			Y	Y	Y	Y						Y										
15699	A_23_P163747	ZNF213							Y	Y								Y	Y	Y		Y	Y	Y			Y																			
2603	A_23_P127839	ZNF214	Y		Y	Y	Y	Y	Y	Y	Y	Y	Y	Y						Y		Y	Y				Y	Y	Y	Y	Y	Y	Y	Y	Y	Y										
3870	A_23_P53057	ZNF215				Y												Y		Y		Y																								
9462	A_23_P210608	ZNF217			Y				Y	Y																	Y																			
18736	A_23_P431360	ZNF219	Y	Y	Y	Y	Y	Y	Y	Y	Y	Y						Y									Y	Y	Y	Y						Y						Y				
16738	A_23_P384512	ZNF22																										Y																		
11696	A_23_P90320	ZNF221			Y																						Y	Y	Y	Y						Y										
13911	A_23_P218516	ZNF222																Y		Y		Y																								
15341	A_23_P108124	ZNF223				Y			Y	Y										Y		Y					Y	Y	Y	Y																
348	A_23_P10885	ZNF224			Y						Y		Y	Y				Y									Y			Y						Y										
508	A_23_P135885	ZNF224			Y	Y		Y	Y	Y	Y	Y	Y	Y				Y	Y	Y		Y	Y	Y			Y	Y	Y	Y						Y										
12638	A_23_P22286	ZNF224			Y	Y		Y	Y	Y	Y	Y	Y	Y				Y		Y		Y					Y			Y						Y										
10569	A_23_P164674	ZNF225			Y	Y		Y	Y	Y	Y	Y	Y	Y						Y							Y	Y	Y	Y						Y										
1895	A_23_P218434	ZNF226			Y															Y																										
10083	A_23_P371005	ZNF227			Y	Y	Y	Y	Y	Y	Y	Y	Y	Y						Y	Y		Y				Y	Y	Y	Y						Y										
18870	A_23_P130509	ZNF229							Y	Y																		Y																		
1720	A_23_P89070	ZNF23																		Y		Y																								
7691	A_23_P125043	ZNF230			Y	Y	Y	Y	Y	Y	Y	Y								Y	Y	Y					Y	Y	Y	Y																
8941	A_23_P4294	ZNF232							Y	Y										Y																										
18461	A_23_P153286	ZNF234			Y	Y	Y	Y	Y	Y	Y	Y								Y								Y																		
5743	A_23_P55605	ZNF236	Y		Y	Y	Y	Y	Y	Y	Y	Y	Y	Y					Y	Y	Y	Y	Y				Y			Y						Y										
1435	A_23_P86731	ZNF239			Y	Y	Y	Y	Y	Y	Y	Y															Y	Y	Y	Y						Y										
10781	A_23_P208049	ZNF24	Y	Y	Y	Y		Y	Y	Y	Y	Y	Y	Y					Y	Y							Y	Y	Y	Y						Y										
15703	A_23_P381577	ZNF25																	Y	Y			Y																							
20477	A_23_P165214	ZNF253																		Y		Y																								
15211	A_23_P324459	ZNF254			Y	Y		Y	Y	Y	Y	Y	Y	Y				Y	Y	Y	Y	Y	Y	Y	Y	Y	Y	Y	Y	Y	Y	Y				Y										
18944	A_23_P16029	ZNF256			Y	Y	Y	Y		Y	Y	Y						Y	Y	Y		Y		Y				Y																		
8215	A_23_P16636	ZNF257				Y			Y	Y	Y	Y	Y	Y				Y		Y			Y					Y		Y						Y										
17047	A_23_P64232	ZNF259			Y	Y		Y	Y	Y	Y	Y	Y	Y						Y							Y	Y	Y	Y						Y										
6660	A_23_P128060	ZNF26			Y	Y		Y	Y	Y										Y		Y					Y			Y						Y										
12468	A_23_P140978	ZNF263																			Y							Y																		
19328	A_23_P107764	ZNF266																	Y																											
C12093.8	A_23_P255592	ZNF268			Y	Y		Y	Y	Y			Y						Y	Y		Y						Y																		
6483	A_23_P147199	ZNF271			Y	Y		Y	Y	Y	Y	Y	Y	Y					Y	Y							Y	Y	Y	Y						Y										
8007	A_23_P158579	ZNF271			Y	Y		Y		Y										Y							Y	Y	Y	Y																
8976	A_23_P251962	ZNF273																					Y																							
9850	A_23_P217135	ZNF275							Y	Y																																				
17759	A_23_P163630	ZNF276			Y	Y	Y	Y	Y	Y	Y	Y	Y	Y				Y	Y	Y	Y	Y	Y	Y	Y	Y	Y	Y	Y	Y	Y	Y				Y										
19459	A_23_P42783	ZNF277																		Y																										
9870	A_23_P154972	ZNF280A																										Y																		
8616	A_23_P402134	ZNF280B	Y		Y																						Y																			
7678	A_23_P14708	ZNF280D			Y	Y	Y	Y	Y	Y	Y	Y	Y	Y				Y	Y	Y		Y	Y	Y			Y	Y	Y	Y						Y										
17463	A_23_P311083	ZNF281			Y	Y	Y	Y	Y	Y	Y	Y						Y	Y	Y		Y	Y	Y			Y	Y	Y	Y																
1288	A_23_P94911	ZNF283																		Y																										
1930	A_23_P119518	ZNF284			Y				Y	Y												Y																								
2049	A_23_P40503	ZNF295			Y													Y									Y																			
11980	A_23_P16058	ZNF296																									Y																			
10458	A_23_P110692	ZNF300				Y			Y	Y																	Y			Y						Y										
18949	A_23_P209032	ZNF302			Y	Y	Y	Y	Y	Y	Y	Y								Y			Y				Y	Y	Y	Y																
18215	A_23_P208280	ZNF304			Y	Y	Y	Y	Y	Y	Y	Y	Y	Y				Y	Y	Y	Y	Y		Y			Y	Y	Y	Y						Y										
2448	A_23_P145175	ZNF318			Y	Y		Y	Y	Y										Y							Y																			
10387	A_23_P26314	ZNF319				Y			Y	Y																																				
1805	A_23_P98052	ZNF32																		Y																										
4373	A_23_P38813	ZNF320				Y			Y	Y										Y		Y						Y																		
7882	A_23_P214501	ZNF322B			Y	Y		Y		Y								Y	Y	Y		Y		Y			Y																			
2344	A_23_P85969	ZNF326				Y																																								
605	A_23_P413633	ZNF329			Y	Y	Y	Y		Y	Y	Y																																		
9585	A_23_P41359	ZNF330									Y		Y	Y																																
11701	A_23_P91254	ZNF334			Y	Y	Y	Y	Y	Y	Y	Y															Y																			
21967	A_23_P17382	ZNF335			Y													Y	Y	Y	Y	Y	Y	Y	Y	Y	Y				Y															
10913	A_23_P154758	ZNF337			Y	Y	Y	Y	Y	Y	Y	Y	Y	Y						Y		Y					Y	Y	Y	Y						Y										
15764	A_23_P17562	ZNF341																		Y																										
22441	A_23_P218706	ZNF343							Y	Y										Y																										
19112	A_23_P39163	ZNF345																		Y																										
7059	A_23_P400887	ZNF346			Y	Y		Y		Y													Y				Y			Y						Y										
7692	A_23_P208250	ZNF347			Y	Y	Y	Y	Y	Y	Y	Y								Y								Y													Y					
936	A_23_P211785	ZNF35																					Y																							
1999	A_23_P78458	ZNF350							Y	Y										Y		Y																								
9919	A_23_P320802	ZNF354A			Y	Y		Y	Y	Y										Y		Y	Y																							
20298	A_23_P94766	ZNF354B			Y	Y		Y	Y	Y									Y	Y		Y	Y				Y																			
2739	A_23_P38968	ZNF358			Y	Y		Y	Y	Y								Y	Y	Y		Y	Y	Y			Y			Y						Y										
9	A_23_P137543	ZNF362																Y																												
9715	A_23_P86610	ZNF365			Y	Y	Y	Y	Y	Y	Y	Y						Y	Y								Y																			
5117	A_23_P407096	ZNF366													Y			Y	Y	Y				Y																						
624	A_23_P16354	ZNF382																		Y																										

14319	A_23_P204355	ZNF384																		Y	Y	Y																								
14803	A_23_P365675	ZNF385A																		Y		Y	Y																						
10773	A_23_P334850	ZNF385B				Y																																							
13509	A_23_P111068	ZNF391			Y	Y		Y	Y	Y									Y	Y		Y	Y																						
12170	A_23_P157416	ZNF394																	Y				Y																						
8915	A_23_P157460	ZNF395																Y		Y		Y																							
19416	A_23_P146077	ZNF395			Y		Y				Y																Y																		
22569	A_23_P392429	ZNF396			Y				Y	Y										Y							Y			Y						Y										
9156	A_23_P208143	ZNF397			Y	Y	Y	Y	Y	Y	Y	Y	Y	Y					Y	Y							Y	Y	Y	Y						Y										
1082	A_23_P78428	ZNF397OS			Y	Y		Y	Y	Y	Y	Y	Y	Y													Y	Y	Y	Y						Y										
10509	A_23_P501214	ZNF398			Y	Y		Y		Y										Y																									
9957	A_23_P90333	ZNF404			Y	Y	Y	Y	Y	Y	Y	Y								Y							Y	Y	Y	Y						Y										
5304	A_23_P208018	ZNF407			Y	Y		Y	Y	Y												Y					Y	Y	Y	Y																
13975	A_23_P45234	ZNF41																	Y																										
10514	A_23_P54179	ZNF410				Y																Y					Y	Y	Y	Y																
19238	A_23_P340922	ZNF414			Y	Y	Y	Y	Y	Y	Y	Y							Y	Y		Y	Y				Y	Y	Y	Y																
9162	A_23_P89871	ZNF415			Y	Y	Y	Y	Y	Y	Y	Y	Y	Y				Y	Y	Y		Y	Y	Y			Y	Y	Y	Y						Y										
14295	A_23_P404595	ZNF417																	Y	Y		Y	Y																						
10501	A_23_P27424	ZNF418			Y	Y		Y		Y												Y																							
22133	A_23_P164636	ZNF419			Y	Y		Y	Y	Y								Y	Y	Y		Y		Y																					
4514	A_23_P380945	ZNF420				Y														Y								Y																	
2898	A_23_P88880	ZNF423																		Y			Y																						
19043	A_23_P168410	ZNF425			Y													Y	Y				Y				Y																		
22112	A_23_P101351	ZNF426			Y	Y		Y	Y	Y								Y	Y	Y		Y	Y	Y			Y	Y	Y	Y						Y										
4861	A_23_P95736	ZNF429				Y			Y	Y	Y	Y	Y	Y						Y		Y	Y					Y		Y						Y										
15934	A_23_P5240	ZNF43				Y			Y	Y										Y		Y	Y					Y		Y						Y										
22182	A_23_P56304	ZNF430				Y			Y	Y										Y		Y	Y					Y		Y						Y										
2638	A_23_P39438	ZNF431				Y			Y	Y										Y			Y					Y																	
14830	A_23_P27649	ZNF433			Y														Y	Y		Y	Y																						
14087	A_23_P218283	ZNF434			Y																						Y	Y	Y	Y																
21671	A_23_P51202	ZNF436							Y	Y	Y	Y	Y	Y					Y	Y										Y						Y										
20164	A_23_P161156	ZNF438																			Y																								
6764	A_23_P32924	ZNF44			Y	Y	Y	Y	Y	Y	Y	Y	Y	Y				Y	Y	Y	Y	Y	Y	Y	Y	Y	Y	Y	Y	Y	Y	Y	Y	Y	Y	Y										
15975	A_23_P119279	ZNF44																			Y						Y																		
18477	A_23_P55911	ZNF440			Y	Y		Y	Y	Y									Y	Y		Y	Y					Y																	
14032	A_23_P316582	ZNF441				Y																																							
1009	A_23_P101476	ZNF442			Y	Y		Y	Y	Y										Y			Y							Y						Y										
19318	A_23_P67284	ZNF443			Y	Y		Y	Y	Y									Y	Y	Y	Y	Y					Y		Y						Y										
21375	A_23_P90088	ZNF443			Y	Y		Y	Y	Y									Y	Y		Y	Y					Y		Y						Y										
17990	A_23_P40785	ZNF445																Y	Y																										
8082	A_23_P101277	ZNF446			Y													Y	Y	Y	Y	Y	Y	Y	Y	Y	Y				Y		Y	Y	Y											
8180	A_23_P309865	ZNF449			Y	Y	Y	Y	Y	Y	Y	Y	Y	Y					Y	Y		Y	Y				Y	Y	Y	Y						Y										
20715	A_23_P101721	ZNF45			Y	Y	Y	Y	Y	Y	Y	Y	Y	Y						Y	Y		Y				Y	Y	Y	Y						Y										
22297	A_23_P426472	ZNF45			Y	Y	Y	Y	Y	Y	Y	Y								Y							Y	Y	Y	Y						Y										
21716	A_23_P356122	ZNF451				Y														Y																									
10086	A_23_P144746	ZNF454							Y	Y										Y																									
5790	A_23_P67424	ZNF461				Y			Y	Y										Y		Y	Y																						
14756	A_23_P60499	ZNF462							Y	Y	Y	Y	Y	Y																															
701	A_23_P59470	ZNF467							Y	Y																	Y			Y						Y										
1620	A_23_P415643	ZNF48	Y		Y	Y		Y		Y																	Y																		
20601	A_23_P208222	ZNF480																									Y																		
16389	A_23_P90508	ZNF486				Y			Y	Y			Y							Y								Y		Y						Y										
18757	A_23_P23966	ZNF488	Y	Y	Y	Y	Y	Y	Y	Y	Y	Y	Y	Y						Y							Y			Y						Y										
5551	A_23_P50405	ZNF490																		Y	Y		Y							Y						Y										
8899	A_23_P410725	ZNF491																				Y																							
5007	A_23_P153855	ZNF492	Y		Y	Y		Y	Y	Y	Y	Y	Y	Y				Y	Y	Y		Y	Y	Y				Y		Y						Y										
728	A_23_P319053	ZNF493																										Y																	
9024	A_23_P354942	ZNF493			Y	Y		Y	Y	Y	Y	Y	Y	Y						Y		Y						Y		Y						Y										
10756	A_23_P104177	ZNF496																				Y	Y																						
10605	A_23_P27414	ZNF497																	Y	Y		Y																							
9018	A_23_P309246	ZNF498				Y																																							
5160	A_23_P92300	ZNF501							Y	Y																	Y			Y						Y										
18994	A_23_P16609	ZNF506			Y				Y	Y																	Y																		
1399	A_23_P208812	ZNF507																		Y																									
8749	A_23_P169233	ZNF510			Y	Y		Y		Y									Y	Y							Y			Y						Y										
1376	A_23_P378488	ZNF511			Y	Y		Y	Y	Y								Y	Y	Y	Y	Y	Y	Y	Y	Y	Y				Y														
4807	A_23_P28550	ZNF512																	Y	Y	Y	Y	Y							Y						Y										
18815	A_23_P391164	ZNF512							Y	Y																				Y						Y										
21840	A_23_P28730	ZNF512B			Y															Y							Y																		
6894	A_23_P142671	ZNF513			Y														Y	Y		Y																							
6326	A_23_P210074	ZNF514			Y	Y		Y		Y										Y			Y				Y	Y	Y	Y						Y										
10989	A_23_P55606	ZNF516			Y	Y		Y		Y																	Y																		
19620	A_23_P328766	ZNF519			Y				Y	Y													Y				Y			Y						Y										
20263	A_23_P159027	ZNF521			Y				Y	Y										Y										Y						Y										
657	A_23_P376735	ZNF524																		Y																									
22081	A_23_P50259	ZNF526																	Y																										
12584	A_23_P101932	ZNF527							Y	Y									Y	Y								Y																	

11293	A_23_P56022	ZNF529			Y	Y	Y	Y		Y	Y	Y	Y	Y				Y	Y	Y		Y	Y	Y			Y	Y	Y	Y						Y										
20919	A_23_P433676	ZNF529			Y				Y	Y										Y							Y			Y						Y										
1341	A_23_P118937	ZNF532																					Y																							
1513	A_23_P346421	ZNF532																	Y	Y																										
8008	A_23_P130811	ZNF536																												Y						Y										
1398	A_23_P141856	ZNF544	Y				Y				Y							Y					Y																							
718	A_23_P130876	ZNF546			Y																		Y				Y																			
13215	A_23_P328786	ZNF546							Y	Y										Y																										
15883	A_23_P333592	ZNF547			Y				Y	Y								Y	Y	Y			Y	Y						Y						Y										
17025	A_23_P4637	ZNF548			Y	Y	Y	Y	Y	Y	Y	Y	Y	Y				Y	Y	Y		Y	Y	Y			Y	Y	Y	Y						Y										
17497	A_23_P399146	ZNF549				Y														Y																										
3179	A_23_P354827	ZNF550				Y																																								
5489	A_23_P38824	ZNF552	Y	Y	Y	Y	Y	Y	Y	Y	Y	Y	Y	Y				Y		Y	Y	Y	Y				Y	Y	Y	Y						Y										
9473	A_23_P343250	ZNF554	Y																Y																											
15009	A_23_P16652	ZNF555				Y												Y	Y	Y		Y	Y	Y																						
1562	A_23_P39456	ZNF556			Y	Y		Y		Y									Y	Y			Y							Y						Y										
18792	A_23_P90023	ZNF557				Y														Y																										
20752	A_23_P28015	ZNF558			Y	Y		Y	Y	Y								Y	Y	Y		Y	Y	Y																						
17580	A_23_P4710	ZNF559							Y	Y					Y			Y	Y	Y		Y	Y	Y																						
10265	A_23_P368773	ZNF560																Y	Y	Y			Y	Y																						
12827	A_23_P433107	ZNF561			Y	Y	Y	Y		Y	Y	Y						Y		Y		Y																								
7067	A_23_P95924	ZNF562	Y		Y	Y		Y	Y	Y									Y	Y			Y					Y		Y						Y										
12580	A_23_P107881	ZNF563				Y														Y		Y	Y					Y																		
6474	A_23_P55880	ZNF564																		Y																										
1051	A_23_P101615	ZNF565																		Y			Y				Y																			
6685	A_23_P354894	ZNF567			Y	Y	Y	Y		Y	Y	Y								Y		Y																								
14380	A_23_P39263	ZNF57																		Y																										
16611	A_23_P108342	ZNF571																		Y																										
3451	A_23_P339079	ZNF573			Y	Y	Y	Y	Y	Y	Y	Y	Y	Y					Y	Y		Y					Y	Y	Y	Y						Y						Y				
19883	A_23_P89931	ZNF574			Y	Y		Y		Y								Y	Y	Y				Y			Y																			
16820	A_23_P208198	ZNF577				Y			Y	Y								Y	Y	Y		Y		Y						Y						Y										
19679	A_23_P339604	ZNF578				Y														Y								Y																		
12372	A_23_P416221	ZNF579			Y																						Y																			
20646	A_23_P164797	ZNF580	Y	Y																																										
19466	A_23_P4793	ZNF581																		Y																										
16266	A_23_P395464	ZNF582			Y													Y	Y	Y		Y		Y			Y																			
18893	A_23_P67432	ZNF583							Y	Y																																				
10192	A_23_P414964	ZNF584			Y		Y				Y		Y	Y				Y	Y		Y	Y					Y																			
6406	A_23_P90223	ZNF585B				Y	Y		Y	Y	Y	Y																Y																		
12053	A_23_P101281	ZNF587	Y	Y	Y	Y	Y	Y	Y	Y	Y	Y	Y	Y				Y	Y	Y	Y	Y	Y	Y	Y	Y	Y	Y	Y	Y	Y	Y				Y								Y		
16966	A_23_P110031	ZNF589			Y	Y		Y	Y	Y																		Y		Y						Y										
15122	A_23_P140496	ZNF592			Y															Y			Y																							
9604	A_23_P34741	ZNF593				Y																																								
17679	A_23_P164299	ZNF594																										Y																		
4327	A_23_P155939	ZNF595																		Y		Y	Y																							
14687	A_23_P82758	ZNF596				Y	Y		Y	Y	Y	Y								Y		Y						Y																		
19050	A_23_P3753	ZNF597			Y																																									
11969	A_23_P100292	ZNF598			Y	Y		Y		Y									Y	Y																										
21760	A_23_P407684	ZNF598			Y	Y	Y	Y	Y	Y	Y	Y						Y	Y	Y	Y	Y	Y	Y	Y	Y	Y	Y	Y	Y	Y	Y						Y				Y			Y	
15972	A_23_P16008	ZNF600				Y			Y	Y										Y		Y	Y					Y																		
6284	A_23_P4626	ZNF606			Y	Y	Y	Y	Y	Y	Y	Y	Y	Y				Y	Y	Y	Y	Y	Y	Y	Y	Y	Y	Y	Y	Y	Y	Y				Y										
4527	A_23_P27810	ZNF607			Y	Y		Y	Y	Y								Y	Y	Y		Y	Y	Y			Y	Y	Y	Y						Y										
13142	A_23_P169978	ZNF608			Y																									Y						Y										
8619	A_23_P416751	ZNF610			Y	Y	Y	Y	Y	Y	Y	Y								Y		Y	Y					Y																		
19968	A_23_P4604	ZNF611			Y	Y	Y	Y	Y	Y	Y	Y	Y	Y						Y								Y		Y						Y							Y			
22333	A_23_P208172	ZNF613																												Y						Y										
14867	A_23_P101253	ZNF614			Y	Y	Y	Y		Y	Y	Y						Y	Y	Y		Y	Y	Y				Y																		
18414	A_23_P355993	ZNF614																Y		Y																										
4540	A_23_P67072	ZNF615				Y																																								
13283	A_23_P153226	ZNF616			Y	Y		Y	Y	Y								Y	Y	Y		Y	Y	Y																						
16807	A_23_P43675	ZNF618			Y																																									
9577	A_23_P156209	ZNF622																										Y																		
768	A_23_P258931	ZNF623			Y													Y		Y							Y																			
9047	A_23_P153037	ZNF624			Y	Y		Y	Y	Y									Y	Y								Y		Y						Y										
11539	A_23_P4850	ZNF625			Y	Y		Y	Y	Y										Y								Y		Y						Y										
7724	A_23_P379142	ZNF626																		Y		Y	Y																							
747	A_23_P135730	ZNF627			Y	Y		Y		Y									Y	Y	Y	Y	Y				Y																			
4274	A_23_P163816	ZNF629			Y	Y		Y	Y	Y	Y	Y	Y	Y						Y							Y	Y	Y	Y						Y								Y		
21874	A_23_P22173	ZNF638			Y	Y		Y	Y	Y									Y	Y		Y	Y				Y	Y	Y	Y						Y										
17445	A_23_P29592	ZNF639			Y				Y	Y																																				
10505	A_23_P64785	ZNF641			Y	Y		Y		Y																	Y	Y	Y	Y																
19186	A_23_P160200	ZNF643				Y														Y																										
16648	A_23_P358857	ZNF644																	Y																											
21880	A_23_P200772	ZNF644																									Y																			
932	A_23_P163826	ZNF646	Y	Y	Y				Y	Y																				Y						Y										
11033	A_23_P208208	ZNF649				Y														Y		Y						Y																		
4712	A_23_P55256	ZNF652									Y		Y	Y				Y																												

17202	A_23_P325676	ZNF653																		Y		Y																								
9948	A_23_P147801	ZNF654				Y			Y	Y								Y	Y	Y		Y	Y	Y				Y		Y						Y										
18247	A_23_P215819	ZNF655																	Y																											
54	A_23_P419206	ZNF658B																		Y																										
17578	A_23_P31938	ZNF658B																		Y																										
1606	A_23_P359654	ZNF664			Y	Y	Y	Y	Y	Y	Y	Y	Y	Y				Y	Y	Y		Y		Y			Y	Y	Y	Y						Y										
20480	A_23_P101443	ZNF665			Y																						Y																			
10839	A_23_P101623	ZNF667			Y				Y	Y										Y										Y						Y										
20399	A_23_P66289	ZNF668			Y	Y		Y		Y									Y	Y	Y	Y	Y				Y	Y	Y	Y						Y										
20172	A_23_P74981	ZNF670																		Y		Y																								
15786	A_23_P50217	ZNF671			Y				Y	Y										Y		Y	Y				Y																			
13214	A_23_P217153	ZNF673			Y				Y	Y								Y	Y	Y		Y	Y	Y			Y			Y						Y										
5185	A_23_P339687	ZNF675				Y			Y	Y			Y							Y		Y	Y					Y		Y						Y										
15881	A_23_P67251	ZNF677																	Y																											
12916	A_23_P422900	ZNF678				Y			Y	Y	Y	Y	Y	Y					Y	Y		Y	Y					Y		Y						Y										
5393	A_23_P337923	ZNF680			Y	Y		Y	Y	Y			Y					Y	Y	Y		Y	Y	Y			Y	Y	Y	Y																
244	A_23_P375517	ZNF681				Y			Y	Y			Y							Y		Y	Y					Y		Y						Y										
7894	A_23_P28012	ZNF682																	Y	Y																										
12207	A_23_P11598	ZNF684																		Y			Y																							
7077	A_23_P331813	ZNF687																	Y	Y		Y	Y																							
12570	A_23_P97221	ZNF691																									Y																			
16510	A_23_P170978	ZNF692			Y																Y																									
18527	A_23_P60101	ZNF696																Y	Y	Y		Y		Y																						
12958	A_23_P255805	ZNF7							Y	Y																																				
19116	A_23_P27638	ZNF700			Y	Y		Y	Y	Y									Y	Y		Y	Y					Y		Y						Y										
5361	A_23_P130444	ZNF701																		Y		Y																								
9913	A_23_P112045	ZNF703							Y	Y										Y	Y									Y						Y										
6768	A_23_P252704	ZNF706			Y	Y		Y		Y																		Y																		
7902	A_23_P411261	ZNF707																Y	Y	Y		Y	Y	Y				Y		Y						Y										
19160	A_23_P83714	ZNF707			Y	Y		Y	Y	Y								Y	Y	Y		Y		Y			Y	Y	Y	Y						Y										
9374	A_23_P119068	ZNF71				Y															Y									Y						Y										
12438	A_23_P345674	ZNF71																		Y										Y						Y										
4193	A_23_P54421	ZNF710			Y	Y		Y	Y	Y																																				
21438	A_23_P217297	ZNF711				Y																																								
18977	A_23_P50834	ZNF714				Y			Y	Y	Y	Y	Y	Y						Y		Y	Y					Y		Y						Y										
6118	A_23_P124438	ZNF718				Y												Y	Y	Y		Y	Y	Y				Y																		
1152	A_23_P28042	ZNF737				Y			Y	Y									Y	Y		Y						Y		Y						Y										
1233	A_23_P17739	ZNF74			Y		Y		Y	Y	Y	Y						Y	Y	Y		Y	Y	Y			Y															Y				
4359	A_23_P31212	ZNF746																		Y																										
15452	A_23_P129587	ZNF75A																Y		Y			Y																							
14520	A_23_P8133	ZNF76																		Y																										
5964	A_23_P153470	ZNF763				Y																																								
3229	A_23_P382175	ZNF765			Y																																									
21620	A_23_P15218	ZNF768			Y	Y		Y		Y								Y	Y	Y	Y	Y	Y	Y	Y	Y	Y				Y															
386	A_23_P351232	ZNF775																Y	Y	Y		Y	Y	Y																						
19141	A_23_P378499	ZNF776			Y	Y	Y	Y	Y	Y	Y	Y	Y	Y				Y	Y	Y		Y	Y	Y			Y	Y	Y	Y																
14653	A_23_P19802	ZNF777																	Y																											
18148	A_23_P56104	ZNF780A				Y			Y	Y										Y								Y		Y						Y										
16265	A_23_P419786	ZNF781				Y																						Y																		
4876	A_23_P78831	ZNF787			Y	Y		Y		Y								Y	Y	Y	Y	Y	Y	Y	Y	Y	Y			Y	Y	Y				Y										
989	A_23_P78825	ZNF790																												Y						Y										
7837	A_23_P50418	ZNF791			Y	Y	Y	Y	Y	Y	Y	Y						Y	Y	Y		Y	Y	Y			Y	Y	Y	Y						Y										
4380	A_23_P67618	ZNF792			Y				Y	Y																				Y						Y										
3909	A_23_P130466	ZNF8			Y													Y		Y							Y																			
9686	A_23_P256526	ZNF80			Y				Y	Y																	Y																			
15536	A_23_P419192	ZNF800																										Y		Y						Y										
3353	A_23_P50198	ZNF816A				Y			Y	Y										Y								Y																		
19073	A_23_P77668	ZNF821				Y												Y		Y		Y																								
15833	A_23_P39050	ZNF823			Y	Y		Y	Y	Y										Y		Y	Y							Y						Y										
14420	A_23_P89139	ZNF83			Y	Y	Y	Y		Y	Y	Y	Y	Y						Y		Y	Y				Y	Y	Y	Y						Y										
19561	A_23_P387071	ZNF830			Y															Y																										
9137	A_23_P2867	ZNF839			Y	Y		Y		Y													Y								Y		Y	Y	Y											
21724	A_23_P105276	ZNF84			Y	Y	Y	Y	Y	Y	Y	Y							Y	Y		Y																								
20476	A_23_P27404	ZNF841				Y	Y				Y								Y	Y		Y	Y					Y																		
5623	A_23_P67702	ZNF85				Y			Y	Y	Y	Y	Y	Y						Y		Y	Y					Y		Y						Y										
8011	A_23_P8408	ZNF862			Y	Y		Y		Y								Y	Y	Y				Y			Y	Y	Y	Y						Y										
3881	A_23_P209146	ZNF91			Y	Y		Y	Y	Y	Y	Y	Y	Y						Y		Y					Y	Y	Y	Y						Y							Y			
6266	A_23_P501080	ZNF92			Y	Y		Y	Y	Y	Y	Y	Y	Y				Y	Y	Y		Y	Y	Y			Y	Y	Y	Y						Y										
14791	A_23_P396570	ZNF99			Y	Y		Y		Y										Y			Y							Y						Y										
7279	A_23_P383435	ZNHIT3																					Y																							
16387	A_23_P201396	ZNHIT6							Y	Y																				Y						Y										
5502	A_23_P8038	ZNRD1																Y			Y																									
16890	A_23_P325010	ZNRF2							Y	Y	Y	Y	Y	Y																																
7218	A_23_P250422	ZNRF3																												Y						Y										
11375	A_23_P1912	ZP1				Y												Y		Y			Y				Y																			
1254	A_23_P134419	ZP3			Y	Y		Y		Y								Y	Y	Y		Y	Y	Y			Y	Y	Y	Y																
8355	A_23_P77853	ZPBP2			Y																																									

8953	A_23_P86706	ZRANB1				Y			Y	Y																																					
10193	A_23_P85519	ZRANB2				Y																						Y																		
18015	A_23_P253909	ZRANB3																									Y																			
6111	A_23_P93015	ZRSR2																			Y							Y																		
18439	A_23_P85053	ZRSR2																		Y	Y		Y																							
12165	A_23_P15135	ZSCAN10				Y														Y		Y	Y																							
2545	A_23_P259094	ZSCAN16			Y	Y		Y		Y										Y																										
7922	A_23_P417371	ZSCAN2																	Y	Y								Y																		
15254	A_23_P205885	ZSCAN2			Y				Y	Y																																				
22117	A_23_P23102	ZSCAN20			Y				Y	Y																				Y						Y										
11274	A_23_P111864	ZSCAN21																												Y						Y										
7400	A_23_P100092	ZSCAN29			Y															Y		Y	Y				Y																			
4518	A_23_P5979	ZSWIM1							Y	Y																				Y						Y										
20370	A_23_P164421	ZSWIM7			Y													Y	Y	Y	Y		Y	Y	Y		Y						Y													
18420	A_23_P112673	ZWILCH																Y																												
6996	A_23_P254887	ZYX			Y	Y	Y	Y	Y	Y	Y	Y						Y	Y	Y	Y	Y	Y	Y	Y	Y	Y	Y	Y	Y	Y	Y	Y	Y	Y	Y										
10601	A_23_P130107	ZZEF1																				Y																								
C9421.5	A_23_P11507	ZZZ3				Y														Y			Y				Y	Y	Y	Y																
57	A_23_P202572																										Y																			
67	A_23_P102536																													Y						Y										
88	A_23_P113263																													Y						Y										
107	A_23_P45382																													Y						Y										
108	A_23_P115943		Y	Y	Y																						Y																			
125	A_23_P136843				Y	Y	Y	Y	Y	Y	Y	Y						Y		Y		Y	Y				Y	Y	Y	Y						Y										
148	A_23_P210592				Y	Y		Y	Y	Y																																				
169	A_23_P15440																			Y			Y																							
184	A_23_P204541																	Y	Y	Y	Y	Y		Y				Y																		
192	A_23_P131915				Y	Y		Y	Y	Y	Y	Y	Y	Y						Y							Y	Y	Y	Y						Y										
221	A_23_P13303				Y																						Y																			
223	A_23_P151491				Y				Y	Y								Y		Y	Y						Y																			
233	A_23_P95311																					Y								Y						Y										
252	A_23_P129503																			Y		Y	Y																							
256	A_23_P254532																		Y																											
258	A_23_P39746				Y																																									
298	A_23_P96191		Y		Y	Y		Y	Y	Y									Y	Y							Y																			
337	A_23_P21585		Y		Y				Y	Y										Y							Y			Y						Y										
339	A_23_P214710				Y																						Y																			
374	A_23_P5435								Y	Y			Y																																	
376	A_23_P7262		Y	Y	Y	Y		Y		Y																																				
385	A_23_P35546								Y	Y																																				
411	A_23_P11520																										Y	Y	Y	Y																
420	A_23_P379501																											Y																		
571	A_23_P131836		Y	Y	Y	Y	Y	Y	Y	Y	Y	Y										Y					Y																Y		
592	A_23_P170217																					Y																								
616	A_23_P136556																				Y																									
640	A_23_P203089																	Y																												
675	A_23_P111898																	Y				Y																								
677	A_23_P203702					Y																																								
680	A_23_P94779				Y				Y	Y	Y	Y	Y	Y							Y						Y																			
703	A_23_P9552																	Y																												
730	A_23_P159183				Y																Y																									
778	A_23_P45260				Y	Y		Y		Y								Y	Y	Y		Y		Y			Y	Y	Y	Y						Y										
831	A_23_P91583				Y	Y		Y		Y																	Y																			
841	A_23_P53946				Y	Y	Y	Y	Y	Y	Y	Y						Y	Y	Y		Y		Y			Y	Y	Y	Y						Y							Y		
879	A_23_P46499				Y																																									
982	A_23_P125365																										Y																			
1024	A_23_P72897		Y		Y	Y		Y		Y																				Y						Y										
1076	A_23_P30870				Y													Y	Y	Y		Y	Y	Y			Y																			
1168	A_23_P80551																										Y																			
1197	A_23_P32583		Y	Y	Y	Y		Y		Y																	Y	Y	Y	Y																
1201	A_23_P96173				Y																						Y			Y						Y										
1222	A_23_P135123								Y	Y																																				
1268	A_23_P31550								Y	Y																																				
1289	A_23_P113549					Y													Y	Y			Y							Y						Y										
1292	A_23_P253863								Y	Y																				Y						Y										
1326	A_23_P71661																													Y						Y										
1346	A_23_P70069				Y	Y		Y	Y	Y	Y	Y	Y	Y				Y	Y	Y		Y		Y				Y		Y						Y										
1357	A_23_P83760		Y		Y	Y	Y	Y	Y	Y	Y	Y						Y	Y	Y		Y		Y			Y	Y	Y	Y	Y	Y	Y	Y	Y											
1362	A_23_P113172																	Y	Y																											
1384	A_23_P83653				Y																																									
1449	A_23_P258930				Y																																									
1478	A_23_P202610																	Y																												
1520	A_23_P113196																			Y																										
1527	A_23_P44946																													Y						Y										
1580	A_23_P61937																	Y	Y																											
1625	A_23_P112577				Y																						Y																			
1663	A_23_P95876				Y	Y		Y	Y	Y	Y	Y	Y	Y						Y							Y	Y	Y	Y						Y										

1679	NA					Y				Y	Y								Y										Y		Y						Y										
1691	A_23_P203630					Y	Y	Y	Y	Y	Y	Y	Y	Y	Y													Y	Y	Y	Y						Y								Y		
1713	A_23_P58091					Y	Y		Y	Y	Y									Y	Y	Y		Y	Y	Y			Y			Y						Y										
1733	A_23_P41561									Y	Y																	Y			Y						Y										
1810	A_23_P135654									Y	Y																				Y						Y										
1820	A_23_P35655																						Y																										
1845	A_23_P32489						Y													Y																												
1851	A_23_P171198					Y	Y		Y		Y										Y	Y		Y																							
1858	A_23_P103051					Y	Y		Y		Y																																					
1871	A_23_P43663			Y	Y	Y	Y		Y		Y										Y								Y		Y						Y										
1876	A_23_P107432			Y	Y	Y	Y	Y	Y	Y	Y	Y	Y			Y			Y		Y		Y	Y				Y	Y	Y	Y	Y	Y	Y	Y	Y	Y										
1903	A_23_P108394																			Y	Y		Y	Y				Y			Y						Y										
1904	A_23_P49424						Y																								Y																		
1906	A_23_P152752					Y														Y		Y							Y																		
1958	A_23_P321220					Y				Y	Y																	Y																		
1995	A_23_P435624									Y	Y																	Y	Y	Y	Y						Y										
2004	A_23_P83990					Y	Y	Y	Y	Y	Y	Y	Y								Y		Y					Y	Y	Y	Y						Y										
2011	A_23_P208582					Y												Y	Y	Y	Y	Y	Y	Y	Y	Y	Y				Y															
2029	NA					Y	Y	Y	Y	Y	Y	Y	Y	Y	Y													Y	Y	Y	Y																	
2046	A_23_P28493			Y		Y										Y					Y																									
2047	A_23_P259531									Y	Y	Y	Y	Y	Y													Y			Y						Y										
2124	A_23_P35369					Y																							Y	Y	Y	Y																	
2127	A_23_P136693					Y	Y	Y	Y	Y	Y	Y	Y							Y	Y							Y	Y	Y	Y						Y										
2179	A_23_P111766					Y										Y			Y	Y								Y																		
2186	A_23_P259040					Y																																								
2217	A_23_P152603																							Y																									
2317	A_23_P157186					Y																							Y																		
2339	A_23_P51966																									Y																							
2343	A_23_P70077					Y				Y	Y																	Y			Y						Y										
2358	A_23_P87299					Y																							Y																		
2378	A_23_P251469									Y	Y																				Y						Y										
2406	A_23_P169960					Y	Y		Y	Y	Y									Y	Y	Y		Y	Y	Y			Y	Y	Y	Y						Y										
2444	A_23_P152446																							Y																									
2455	A_23_P3340					Y	Y		Y	Y	Y																				Y						Y										
2471	A_23_P159163			Y		Y	Y		Y	Y	Y	Y	Y	Y	Y					Y	Y							Y																		
2483	A_23_P256911																				Y	Y																											
2493	A_23_P86671						Y																																							
2500	A_23_P5699						Y											Y	Y																											
2536	A_23_P102607					Y	Y		Y	Y	Y																	Y	Y	Y	Y						Y										
2553	A_23_P117480					Y	Y		Y	Y	Y										Y			Y				Y	Y	Y	Y						Y										
2559	A_23_P140884																									Y																							
2709	A_23_P101121						Y																Y																								
2712	A_23_P139104													Y						Y	Y	Y		Y	Y	Y																							
2717	A_23_P169788						Y																																							
2765	A_23_P10656					Y	Y		Y		Y																	Y	Y	Y	Y																	
2772	A_23_P9732					Y				Y	Y																	Y	Y	Y	Y																	
2831	A_23_P44663					Y																								Y																		
2833	NA					Y	Y	Y	Y	Y	Y	Y	Y	Y	Y													Y	Y	Y	Y						Y										
2911	A_23_P16874					Y	Y	Y	Y	Y	Y	Y	Y															Y																		
2948	A_23_P251002																													Y																		
2972	NA					Y	Y	Y	Y	Y	Y	Y	Y	Y	Y													Y	Y	Y	Y						Y										
3055	A_23_P216536									Y	Y										Y																										
3056	A_23_P16408									Y	Y									Y	Y			Y																								
3068	A_23_P109720									Y	Y																																			
3122	A_23_P200843					Y				Y	Y	Y	Y	Y	Y													Y			Y						Y										
3131	A_23_P84659						Y			Y	Y																																			
3151	A_23_P208275					Y				Y	Y	Y	Y	Y	Y									Y							Y						Y										
3192	A_23_P253168					Y	Y		Y		Y																	Y	Y	Y	Y																	
3214	A_23_P376471					Y				Y	Y																																			
3217	A_23_P21249			Y	Y	Y	Y	Y	Y	Y	Y	Y	Y						Y	Y	Y		Y		Y			Y	Y	Y	Y	Y	Y	Y	Y	Y	Y										
3283	A_23_P159438			Y	Y	Y	Y	Y	Y	Y	Y	Y	Y	Y	Y	Y			Y	Y	Y				Y			Y	Y	Y	Y			Y	Y													
3291	A_23_P124699					Y																																								
3336	A_23_P159208											Y		Y	Y						Y							Y																		
3352	A_23_P252796					Y	Y		Y		Y							Y		Y			Y				Y			Y						Y										
3360	A_23_P144677					Y	Y	Y	Y	Y	Y	Y	Y	Y	Y					Y	Y		Y					Y	Y	Y	Y						Y										
3401	A_23_P206841					Y	Y		Y		Y							Y								Y																		
3417	A_23_P72890					Y	Y	Y	Y	Y	Y	Y	Y															Y																		
3458	A_23_P140780																																Y						Y										
3462	A_23_P58072																						Y																										
3475	A_23_P143824																									Y																							
3482	A_23_P212180			Y	Y	Y	Y		Y		Y	Y	Y	Y	Y						Y							Y	Y	Y	Y						Y										
3531	A_23_P84966					Y																																								
3538	A_23_P124264			Y	Y	Y				Y	Y																	Y																		
3573	A_23_P165921						Y																							Y	Y	Y	Y																	
3579	A_23_P58928					Y																																								
3582	A_23_P104970									Y	Y																																			
3586	A_23_P21266					Y															Y			Y																							

3621	A_23_P81780				Y				Y	Y	Y	Y	Y	Y													Y			Y							Y														
3642	A_23_P434178				Y	Y		Y		Y											Y			Y				Y																				
3725	A_23_P72518				Y	Y	Y	Y	Y	Y	Y	Y	Y	Y				Y	Y	Y	Y	Y	Y	Y	Y	Y	Y	Y	Y	Y	Y	Y				Y										
3734	A_23_P5512				Y																																									
3762	A_23_P147564				Y																						Y																				
3771	A_23_P127297				Y																						Y	Y	Y	Y																		
3784	A_23_P22086				Y	Y		Y		Y										Y							Y			Y							Y										
3840	A_23_P204218				Y	Y		Y		Y									Y		Y		Y																									
3853	A_23_P113294				Y	Y		Y		Y										Y		Y						Y	Y	Y	Y							Y										
3867	A_23_P104393																										Y																				
3882	A_23_P113605																											Y																			
3883	A_23_P124823					Y																							Y							Y										
3889	A_23_P64048																	Y								Y																				
3922	A_23_P136020					Y			Y	Y																	Y		Y							Y										
3945	A_23_P31348																		Y			Y			Y																				
3979	A_23_P7451								Y	Y										Y																											
4010	A_23_P148526				Y	Y	Y	Y		Y	Y	Y										Y	Y				Y	Y	Y	Y							Y										
4013	A_23_P23110																										Y																				
4014	A_23_P21260			Y		Y	Y	Y	Y	Y	Y	Y	Y	Y	Y					Y	Y								Y	Y	Y	Y							Y										
4023	A_23_P82461																				Y																									
4055	A_23_P143360								Y	Y	Y	Y	Y	Y							Y		Y								Y			Y			Y										
4128	A_23_P117214																													Y							Y										
4161	A_23_P154011																	Y	Y		Y	Y																							
4179	A_23_P155551								Y	Y																Y			Y							Y										
4215	A_23_P5479				Y																																									
4230	A_23_P115616			Y	Y	Y	Y		Y		Y	Y	Y	Y	Y																Y		Y							Y						Y				
4232	A_23_P169717					Y																						Y																			
4323	A_23_P119864																													Y							Y										
4338	A_23_P61192								Y	Y																Y			Y							Y										
4343	A_23_P11895																	Y								Y																				
4366	A_23_P123656																	Y												Y							Y										
4370	A_23_P253264																	Y			Y								Y							Y										
4371	A_23_P147855																		Y	Y	Y																									
4398	A_23_P254967					Y			Y	Y											Y		Y																						
4446	A_23_P116523				Y	Y		Y		Y										Y		Y	Y				Y	Y	Y	Y																
4460	A_23_P105160																				Y						Y	Y	Y	Y																
4487	A_23_P170800				Y	Y		Y		Y																	Y			Y							Y										
4526	A_23_P44326																										Y																				
4568	A_23_P205670								Y	Y														Y					Y		Y							Y										
4582	A_23_P64962					Y												Y	Y	Y		Y		Y				Y		Y							Y										
4648	A_23_P212294				Y	Y		Y		Y										Y			Y																						
4662	A_23_P136680																										Y																				
4693	A_23_P102150																											Y		Y							Y										
4730	A_23_P4462				Y	Y	Y	Y	Y	Y	Y	Y	Y	Y				Y	Y	Y		Y	Y	Y			Y	Y	Y	Y							Y										
4790	A_23_P136753								Y	Y										Y																											
4823	A_23_P110655				Y	Y		Y	Y	Y																		Y		Y							Y										
4832	A_23_P36722																Y	Y	Y		Y	Y	Y			Y																				
4849	A_23_P133087																				Y																							
4882	A_23_P112332																					Y																						
4917	A_23_P43917			Y	Y	Y	Y	Y	Y	Y	Y	Y	Y	Y	Y	Y			Y	Y	Y				Y			Y	Y	Y	Y																
4975	A_23_P44053			Y	Y	Y	Y	Y	Y	Y	Y	Y	Y						Y	Y	Y				Y			Y	Y	Y	Y																
4978	A_23_P82191				Y																						Y																				
5000	A_23_P160399																				Y																							
5045	A_23_P119976																Y																												
5079	A_23_P258121					Y																																								
5148	NA				Y	Y	Y	Y	Y	Y	Y	Y	Y	Y				Y	Y	Y	Y		Y	Y	Y		Y	Y	Y	Y			Y			Y										
5165	A_23_P47980																													Y							Y										
5173	A_23_P206084																				Y																									
5294	A_23_P103951																	Y			Y																							
5345	A_23_P163541																										Y																				
5372	A_23_P16306				Y	Y	Y	Y		Y	Y	Y						Y	Y	Y	Y	Y	Y	Y	Y	Y	Y				Y																
5458	A_23_P217596				Y	Y		Y		Y																																			
5540	A_23_P148437				Y	Y	Y	Y	Y	Y	Y	Y	Y	Y				Y		Y		Y	Y				Y	Y	Y	Y							Y										
5553	A_23_P301328				Y	Y		Y		Y										Y						Y																				
5564	A_23_P71680					Y			Y	Y																	Y		Y							Y										
5598	A_23_P5171				Y	Y		Y	Y	Y										Y	Y		Y	Y				Y																				
5631	A_23_P11902				Y	Y		Y	Y	Y											Y		Y	Y					Y																			
5649	A_23_P36084																				Y																							
5689	A_23_P84721																		Y																									
5692	A_23_P9280				Y	Y	Y	Y	Y	Y	Y	Y								Y	Y	Y		Y	Y	Y			Y	Y	Y	Y							Y								Y		
5831	A_23_P150741					Y			Y	Y																																					
5838	A_23_P10564			Y	Y	Y	Y	Y	Y		Y	Y	Y	Y	Y				Y	Y		Y						Y	Y	Y	Y																
5913	A_23_P74398																	Y																										
5975	A_23_P60754								Y	Y									Y	Y						Y	Y	Y	Y							Y										
6018	A_23_P10060				Y														Y	Y			Y			Y																				
6022	A_23_P381489								Y	Y																																			
6028	A_23_P159024								Y	Y																																			

6065	A_23_P161257																						Y																							
6140	A_23_P1894				Y															Y			Y																							
6201	A_23_P65953				Y																																									
6212	A_23_P110752																				Y																									
6227	A_23_P48430																				Y							Y																		
6296	A_23_P150960		Y	Y	Y	Y		Y	Y	Y																	Y	Y	Y	Y						Y										
6309	A_23_P88554																			Y																										
6310	A_23_P158380				Y		Y				Y																Y																			
6314	A_23_P72271		Y	Y	Y	Y		Y	Y	Y									Y	Y							Y			Y						Y										
6340	NA				Y	Y		Y	Y	Y	Y	Y	Y	Y													Y	Y	Y	Y						Y										
6346	A_23_P92579																						Y																							
6403	A_23_P122323								Y	Y																																				
6449	A_23_P200456																													Y						Y										
6492	A_23_P107048																											Y																		
6505	A_23_P95619				Y				Y	Y	Y	Y	Y	Y									Y				Y	Y	Y	Y						Y										
6518	NA				Y	Y		Y	Y	Y	Y	Y	Y	Y													Y			Y						Y								Y		
6546	A_23_P130096					Y																					Y			Y						Y										
6566	A_23_P167624		Y	Y	Y	Y		Y		Y									Y								Y	Y	Y	Y																
6574	A_23_P48051								Y	Y																																				
6596	A_23_P318943				Y	Y		Y		Y																	Y	Y	Y	Y																
6615	NA				Y	Y	Y	Y	Y	Y	Y	Y	Y	Y				Y	Y	Y	Y	Y	Y	Y	Y	Y	Y	Y	Y	Y	Y	Y	Y	Y	Y	Y										
6732	A_23_P9707					Y																																								
6742	A_23_P124442				Y	Y		Y	Y	Y	Y	Y	Y	Y				Y	Y	Y		Y		Y			Y																			
6794	NA				Y	Y		Y	Y	Y	Y	Y	Y	Y													Y	Y	Y	Y						Y										
6901	A_23_P128911					Y																																								
6918	A_23_P258174					Y			Y	Y																		Y																		
6962	A_23_P135383					Y																						Y																		
7037	A_23_P211319				Y	Y		Y		Y										Y																										
7054	A_23_P29472				Y	Y		Y		Y																																				
7070	A_23_P75830				Y																						Y	Y	Y	Y						Y										
7080	A_23_P21907																						Y																							
7101	A_23_P219099				Y																																									
7133	A_23_P107007																		Y											Y						Y										
7143	A_23_P113952																													Y						Y										
7167	A_23_P169319		Y	Y	Y	Y	Y	Y	Y	Y	Y	Y	Y	Y				Y	Y			Y	Y				Y	Y	Y	Y						Y								Y		
7185	A_23_P104250																										Y			Y						Y										
7189	A_23_P72400		Y	Y	Y	Y	Y	Y	Y	Y	Y	Y	Y	Y				Y	Y	Y		Y		Y			Y	Y	Y	Y	Y	Y	Y	Y	Y	Y										
7196	A_23_P207235				Y													Y	Y								Y																			
7213	A_23_P80788				Y																	Y																								
7232	A_23_P349184				Y	Y		Y	Y	Y										Y		Y						Y																		
7246	A_23_P85120				Y																						Y			Y						Y										
7268	A_23_P93871																			Y			Y																							
7277	A_23_P255111				Y													Y	Y	Y			Y	Y																						
7319	A_23_P33642																													Y						Y										
7386	A_23_P120082																										Y			Y						Y										
7402	A_23_P417731				Y																						Y																			
7431	A_23_P49564				Y	Y	Y	Y	Y	Y	Y	Y	Y	Y				Y	Y	Y	Y		Y	Y	Y		Y	Y	Y	Y	Y	Y	Y	Y	Y	Y										
7440	NA				Y	Y	Y	Y	Y	Y	Y	Y	Y	Y													Y	Y	Y	Y						Y										
7442	A_23_P30415					Y																																								
7488	A_23_P135946								Y	Y																																				
7565	A_23_P170387																			Y			Y																							
7573	A_23_P33521																													Y						Y										
7585	A_23_P31931								Y	Y																																				
7619	A_23_P250323																											Y																		
7628	A_23_P86360		Y	Y																																										
7657	A_23_P32821								Y	Y																				Y						Y										
7690	A_23_P252501				Y																																									
7708	A_23_P141785				Y	Y	Y	Y		Y	Y	Y						Y										Y																		
7741	A_23_P2378				Y	Y		Y	Y	Y																		Y		Y						Y										
7759	A_23_P42680								Y	Y																																				
7809	A_23_P166508				Y	Y		Y	Y	Y	Y	Y	Y	Y					Y			Y					Y	Y	Y	Y						Y										
7811	A_23_P123189																													Y						Y										
7812	A_23_P87041																													Y						Y										
7842	A_23_P259512																						Y																							
7849	A_23_P209066																	Y		Y	Y		Y								Y		Y	Y	Y											
7865	NA				Y	Y		Y	Y	Y	Y	Y	Y	Y													Y	Y	Y	Y						Y										
7875	A_23_P211661																	Y																												
7896	A_23_P21234																	Y	Y											Y						Y										
7898	A_23_P9997		Y	Y	Y	Y		Y	Y	Y									Y	Y							Y																			
7911	A_23_P113383				Y	Y	Y	Y		Y	Y	Y						Y	Y		Y	Y																								
7915	A_23_P251953					Y																						Y																		
7926	A_23_P37317				Y				Y	Y																	Y																			
7964	A_23_P332782				Y	Y		Y	Y	Y										Y	Y	Y	Y				Y																			
7998	A_23_P26311																	Y	Y			Y								Y						Y										
8006	A_23_P209270																			Y																										
8009	A_23_P211899		Y		Y	Y	Y	Y	Y	Y	Y	Y	Y	Y				Y	Y	Y	Y	Y	Y	Y	Y	Y	Y	Y	Y	Y	Y	Y	Y	Y	Y					Y						
8016	A_23_P384965																	Y																												

8022	A_23_P216071		Y		Y	Y	Y	Y	Y	Y	Y	Y	Y	Y						Y			Y				Y	Y	Y	Y						Y										
8048	A_23_P118370				Y																																									
8081	A_23_P138343																													Y						Y										
8091	A_23_P214008					Y																																								
8112	A_23_P41564				Y																						Y			Y						Y										
8113	A_23_P135063				Y				Y	Y								Y									Y			Y						Y										
8125	A_23_P390458					Y																								Y						Y										
8135	A_23_P75197				Y																									Y						Y										
8159	A_23_P171165					Y																						Y		Y						Y										
8161	A_23_P12826																			Y	Y																									
8174	A_23_P124177					Y																																								
8181	A_23_P120165					Y																																								
8191	A_23_P13236		Y	Y	Y	Y	Y	Y	Y	Y	Y	Y																Y																		
8289	A_23_P52783																										Y																			
8309	A_23_P43744		Y	Y	Y													Y	Y								Y																			
8319	A_23_P47546				Y	Y		Y	Y	Y									Y	Y		Y	Y				Y	Y	Y	Y						Y										
8326	NA				Y	Y		Y	Y	Y	Y	Y	Y	Y						Y								Y		Y						Y										
8354	A_23_P149791																		Y																											
8365	A_23_P70998				Y	Y	Y	Y	Y	Y	Y	Y							Y	Y		Y	Y				Y	Y	Y	Y						Y										
8417	A_23_P123517				Y	Y		Y		Y										Y			Y																							
8428	A_23_P250842				Y																						Y																			
8443	A_23_P171007																						Y					Y																		
8456	A_23_P251196																		Y	Y		Y	Y																							
8458	A_23_P58137		Y	Y					Y	Y																																				
8460	NA				Y	Y		Y	Y	Y	Y	Y	Y	Y					Y								Y	Y	Y	Y						Y								Y		
8481	A_23_P42991				Y	Y		Y	Y	Y									Y	Y		Y	Y					Y																		
8491	A_23_P377191				Y																						Y			Y						Y										
8495	A_23_P54406																			Y			Y																							
8541	A_23_P153983					Y			Y	Y																		Y		Y						Y										
8544	A_23_P313588				Y															Y																										
8555	A_23_P36845																				Y																									
8556	A_23_P256366				Y																Y		Y																							
8567	A_23_P9814		Y	Y	Y	Y		Y	Y	Y									Y	Y							Y			Y						Y										
8576	A_23_P313605		Y	Y	Y	Y		Y	Y	Y																	Y																			
8606	A_23_P145952																											Y																		
8607	A_23_P307895								Y	Y									Y																											
8641	A_23_P259429								Y	Y																																				
8663	A_23_P33429		Y	Y	Y								Y																	Y						Y										
8682	NA				Y	Y	Y	Y	Y	Y	Y	Y	Y	Y				Y	Y	Y			Y	Y			Y	Y	Y	Y						Y										
8744	A_23_P216335														Y																															
8754	A_23_P75129				Y																																									
8769	A_23_P75780		Y	Y	Y	Y	Y	Y	Y	Y	Y	Y	Y	Y				Y	Y	Y			Y	Y			Y	Y	Y	Y						Y										
8823	A_23_P62779		Y	Y														Y												Y						Y										
8837	A_23_P206135																					Y																								
8841	A_23_P32684				Y	Y		Y	Y	Y	Y	Y	Y	Y														Y		Y						Y										
8853	A_23_P166586																			Y			Y																							
8866	A_23_P40225		Y		Y		Y				Y									Y			Y				Y	Y	Y	Y																
8916	A_23_P166947																													Y						Y										
8936	A_23_P154997					Y																																								
8950	A_23_P159488								Y	Y																																				
8960	A_23_P136721				Y	Y	Y	Y	Y	Y	Y	Y	Y	Y													Y																			
8999	A_23_P17152																			Y			Y																							
9069	A_23_P10987				Y													Y	Y	Y	Y	Y	Y	Y	Y	Y	Y				Y		Y													
9088	A_23_P255070																	Y	Y	Y				Y						Y						Y										
9098	A_23_P393794																	Y	Y	Y				Y																						
9105	A_23_P7512		Y	Y	Y	Y		Y		Y																	Y	Y	Y	Y						Y										
9128	A_23_P145445					Y																																								
9146	A_23_P73392		Y		Y														Y	Y							Y																			
9180	A_23_P28927																			Y			Y				Y																			
9192	A_23_P23705				Y	Y		Y	Y	Y	Y	Y	Y	Y					Y	Y	Y	Y	Y					Y		Y						Y										
9213	A_23_P205019																				Y																									
9264	A_23_P68958				Y				Y	Y																		Y																		
9302	A_23_P79049					Y																								Y						Y										
9319	A_23_P46660																		Y																											
9329	A_23_P120730																						Y																							
9358	A_23_P123754		Y		Y	Y		Y		Y					Y					Y																										
9410	A_23_P376752					Y																																								
9420	A_23_P104540				Y	Y	Y	Y	Y	Y	Y	Y	Y	Y				Y		Y	Y	Y	Y				Y	Y	Y	Y			Y			Y										
9483	A_23_P33494		Y	Y	Y	Y		Y	Y	Y	Y	Y	Y	Y													Y	Y	Y	Y						Y										
9496	A_23_P428992																										Y																			
9501	A_23_P84284																	Y	Y	Y				Y			Y			Y						Y										
9506	A_23_P122364																													Y						Y										
9549	NA				Y	Y	Y	Y	Y	Y	Y	Y	Y	Y				Y	Y	Y	Y	Y	Y	Y	Y	Y	Y	Y	Y	Y	Y	Y	Y	Y	Y	Y										
9628	A_23_P82538				Y																		Y				Y																			
9658	A_23_P84268		Y	Y	Y	Y	Y	Y	Y	Y	Y	Y	Y	Y													Y	Y	Y	Y						Y								Y		
9683	A_23_P112709																	Y																												
9698	A_23_P111424		Y	Y	Y	Y		Y		Y								Y	Y								Y	Y	Y	Y						Y										

9721	A_23_P144249																										Y																			
9748	A_23_P435390		Y		Y		Y		Y	Y	Y	Y	Y	Y				Y	Y	Y				Y			Y																			
9750	A_23_P31883		Y	Y	Y	Y	Y	Y		Y	Y	Y						Y	Y	Y				Y			Y	Y	Y	Y						Y						Y				
9755	A_23_P159805																					Y																							
9783	A_23_P95486																	Y																											
9816	A_23_P210470				Y																						Y		Y						Y										
9817	A_23_P112861																		Y			Y																							
9855	NA				Y	Y		Y	Y	Y	Y	Y	Y	Y													Y	Y	Y	Y						Y										
9863	A_23_P203228				Y															Y			Y				Y																			
9952	A_23_P305138				Y						Y		Y	Y													Y	Y	Y	Y						Y										
9979	A_23_P15018				Y													Y		Y		Y	Y																							
10002	A_23_P99821					Y			Y	Y																																				
10015	A_23_P33075				Y	Y	Y	Y		Y	Y	Y							Y	Y		Y					Y			Y						Y								Y		
10033	A_23_P348015					Y			Y	Y	Y	Y	Y	Y														Y		Y						Y										
10053	A_23_P38628																										Y																		
10062	A_23_P50645																		Y			Y																							
10074	A_23_P204225				Y				Y	Y								Y	Y	Y	Y	Y		Y																						
10096	A_23_P30150							Y	Y																		Y		Y						Y										
10111	A_23_P73383																Y																												
10151	A_23_P36205							Y	Y																				Y						Y										
10174	A_23_P80487					Y			Y	Y	Y	Y	Y	Y				Y	Y	Y				Y						Y						Y										
10175	A_23_P250019		Y	Y	Y				Y	Y	Y	Y	Y	Y													Y	Y	Y	Y						Y										
10195	A_23_P89369				Y	Y		Y		Y								Y	Y	Y	Y	Y	Y	Y	Y	Y	Y				Y															
10276	A_23_P63441				Y																						Y			Y						Y										
10292	A_23_P102812																		Y													Y	Y												
10297	A_23_P369758		Y	Y	Y	Y		Y		Y																		Y		Y						Y										
10336	A_23_P95940																	Y	Y								Y																		
10338	A_23_P52951					Y			Y	Y																																				
10341	A_23_P113047																										Y																		
10396	A_23_P403209		Y	Y	Y	Y	Y	Y	Y	Y	Y	Y	Y	Y				Y	Y	Y	Y	Y	Y	Y	Y	Y	Y	Y	Y	Y	Y	Y	Y	Y	Y	Y		Y				Y			Y	Y
10405	A_23_P84539																		Y								Y																		
10463	A_23_P13375							Y	Y																																				
10482	A_23_P32950																												Y						Y										
10485	A_23_P253631																		Y							Y																			
10507	A_23_P167408																	Y																											
10526	A_23_P135809				Y														Y	Y			Y				Y																			
10584	A_23_P147404					Y			Y	Y								Y																												
10624	A_23_P129405																		Y	Y	Y	Y																							
10627	A_23_P21306		Y	Y	Y				Y	Y																	Y																			
10653	A_23_P103743				Y								Y							Y							Y																			
10654	A_23_P24605																									Y																			
10655	A_23_P136603																			Y																									
10664	A_23_P113904				Y	Y		Y	Y	Y	Y	Y	Y	Y					Y	Y							Y	Y	Y	Y						Y										
10670	A_23_P110353		Y	Y	Y													Y	Y	Y		Y		Y			Y																			
10764	A_23_P44431							Y	Y	Y	Y	Y	Y													Y			Y						Y										
10812	A_23_P78244																	Y								Y																			
10841	A_23_P136857				Y																																									
10863	A_23_P85755				Y																									Y						Y										
10865	A_23_P213986				Y	Y		Y		Y										Y																										
10885	A_23_P402036																		Y																										
10934	A_23_P209303																		Y																										
10943	A_23_P125851					Y					Y		Y	Y					Y	Y		Y																								
10963	A_23_P51082				Y																						Y																			
10975	A_23_P162105																										Y																		
10997	A_23_P142859																					Y																							
11010	A_23_P50320				Y	Y		Y	Y	Y																		Y		Y						Y										
11014	A_23_P56851				Y	Y		Y	Y	Y	Y	Y	Y	Y													Y	Y	Y	Y						Y										
11031	A_23_P10331																									Y																			
11039	A_23_P218214				Y	Y		Y	Y	Y	Y	Y	Y	Y														Y		Y						Y										
11048	A_23_P88012																			Y		Y										Y													
11066	NA				Y	Y	Y	Y	Y	Y	Y	Y	Y	Y				Y		Y	Y	Y	Y				Y	Y	Y	Y			Y			Y										
11078	A_23_P44235				Y				Y	Y																																				
11081	A_23_P166485							Y	Y	Y	Y	Y	Y																Y						Y										
11084	A_23_P72473		Y	Y	Y	Y		Y	Y	Y					Y			Y	Y	Y				Y			Y	Y	Y	Y																
11096	A_23_P123838				Y													Y		Y																										
11101	A_23_P354208																					Y																							
11137	A_23_P170830				Y														Y	Y							Y																			
11149	A_23_P84475				Y															Y			Y																							
11166	A_23_P1847				Y				Y	Y																	Y			Y						Y										
11207	A_23_P14047				Y														Y	Y							Y																			
11218	A_23_P61559					Y																						Y		Y						Y										
11244	A_23_P47445				Y	Y		Y		Y								Y		Y			Y				Y																			
11248	A_23_P140614				Y																						Y			Y						Y										
11357	A_23_P392897																					Y																							
11400	A_23_P118134		Y																									Y																		
11403	A_23_P156611				Y																									Y						Y										
11434	A_23_P26658				Y													Y									Y																			

11437	A_23_P74562				Y																																									
11444	A_23_P214344																														Y						Y										
11473	A_23_P46523																												Y																	
11479	A_23_P209882				Y													Y	Y	Y		Y	Y	Y			Y	Y	Y	Y						Y										
11511	A_23_P213653					Y														Y			Y																							
11512	A_23_P95499				Y	Y	Y	Y	Y	Y	Y	Y	Y	Y				Y	Y	Y		Y	Y	Y			Y			Y						Y										
11521	A_23_P30055				Y																						Y			Y						Y										
11550	A_23_P101494		Y	Y	Y															Y			Y				Y						Y													
11574	A_23_P124703																		Y																											
11585	A_23_P10488				Y																																									
11591	NA				Y				Y	Y	Y	Y	Y	Y																Y						Y										
11608	A_23_P166713				Y	Y		Y		Y	Y	Y	Y	Y													Y			Y						Y										
11613	A_23_P17444				Y		Y		Y	Y	Y	Y						Y	Y	Y				Y			Y			Y						Y										
11615	A_23_P52621																											Y																		
11670	A_23_P206120																				Y									Y						Y										
11709	A_23_P258144				Y																						Y																			
11723	A_23_P75358				Y	Y		Y		Y																	Y																			
11733	NA				Y	Y		Y	Y	Y	Y	Y	Y	Y													Y			Y						Y										
11753	A_23_P44458					Y																																								
11756	A_23_P7912				Y																	Y								Y						Y										
11761	A_23_P33511																			Y																										
11776	A_23_P122478					Y																																								
11825	A_23_P86779					Y			Y	Y																				Y						Y										
11831	A_23_P113891				Y	Y		Y	Y	Y	Y	Y	Y	Y						Y		Y					Y	Y	Y	Y						Y										
11842	A_23_P138725																													Y						Y										
11852	A_23_P106412				Y	Y		Y		Y												Y					Y																			
11859	A_23_P407750																										Y																			
11860	A_23_P120644				Y															Y										Y						Y										
11863	A_23_P216581				Y															Y		Y																								
11934	A_23_P328497																										Y																			
11947	A_23_P96033				Y	Y	Y	Y	Y	Y	Y	Y															Y	Y	Y	Y						Y										
11961	A_23_P113056		Y	Y	Y		Y		Y	Y	Y	Y						Y	Y	Y				Y			Y																			
11987	A_23_P164368				Y	Y		Y	Y	Y	Y	Y	Y	Y													Y	Y	Y	Y						Y										
12026	A_23_P116110																			Y																										
12081	A_23_P212649				Y	Y		Y	Y	Y								Y	Y	Y		Y	Y	Y						Y						Y										
12088	A_23_P125551								Y	Y																		Y																		
12160	A_23_P130224				Y																																									
12173	A_23_P16315				Y	Y	Y	Y		Y	Y	Y	Y	Y					Y	Y	Y	Y	Y				Y	Y	Y	Y												Y				
12200	A_23_P137837				Y	Y		Y	Y	Y								Y		Y		Y					Y																			
12209	A_23_P163717				Y														Y	Y			Y				Y																			
12271	A_23_P254330					Y			Y	Y																		Y		Y						Y										
12278	A_23_P124078				Y	Y		Y	Y	Y																	Y	Y	Y	Y						Y										
12337	A_23_P97218		Y	Y	Y	Y		Y	Y	Y	Y	Y	Y	Y						Y			Y				Y			Y						Y										
12355	A_23_P79152				Y				Y	Y	Y	Y	Y	Y						Y	Y						Y			Y						Y										
12385	A_23_P205083				Y	Y		Y		Y	Y	Y	Y	Y						Y		Y	Y				Y	Y	Y	Y																
12406	A_23_P166502				Y				Y	Y								Y	Y	Y	Y	Y		Y			Y																			
12407	A_23_P52517																			Y																										
12424	A_23_P143285				Y	Y	Y	Y	Y	Y	Y	Y	Y	Y														Y		Y						Y										
12439	A_23_P162646				Y	Y	Y	Y		Y	Y	Y						Y	Y	Y	Y	Y	Y	Y	Y	Y	Y	Y	Y	Y	Y	Y	Y			Y								Y		
12500	A_23_P256923		Y		Y				Y	Y																	Y																			
12525	A_23_P108534				Y	Y		Y		Y								Y	Y	Y		Y		Y			Y	Y	Y	Y						Y										
12529	A_23_P206884																	Y		Y		Y																								
12558	A_23_P208719		Y	Y	Y						Y		Y	Y													Y			Y						Y										
12559	A_23_P73328		Y	Y	Y	Y	Y	Y	Y	Y	Y	Y	Y	Y				Y	Y	Y		Y		Y			Y	Y	Y	Y																
12581	A_23_P255137				Y	Y	Y	Y		Y	Y	Y											Y				Y			Y						Y										
12662	A_23_P136023		Y	Y	Y	Y	Y	Y	Y	Y	Y	Y						Y	Y	Y		Y		Y			Y	Y	Y	Y																
12698	A_23_P102384				Y				Y	Y																				Y						Y										
12707	A_23_P43982				Y	Y		Y	Y	Y																	Y			Y						Y										
12743	A_23_P322691																				Y	Y																								
12763	A_23_P84679		Y	Y	Y	Y	Y	Y	Y	Y	Y	Y	Y	Y				Y	Y	Y				Y			Y	Y	Y	Y																
12765	A_23_P115902		Y	Y	Y	Y	Y	Y	Y	Y	Y	Y	Y	Y				Y		Y		Y					Y	Y	Y	Y						Y										
12788	NA				Y	Y	Y	Y	Y	Y	Y	Y	Y	Y				Y		Y	Y	Y	Y				Y	Y	Y	Y			Y			Y										
12811	A_23_P256307				Y	Y		Y	Y	Y									Y	Y	Y	Y	Y				Y	Y	Y	Y																
12813	A_23_P43630					Y			Y	Y																				Y						Y										
12825	A_23_P158068																					Y																								
12832	A_23_P216388																			Y		Y																								
12907	A_23_P47653																										Y																			
12919	A_23_P71548					Y																																								
12927	A_23_P149638				Y															Y							Y																			
12933	A_23_P44536																											Y																		
12944	A_23_P144317					Y							Y																																	
13024	A_23_P99657				Y		Y		Y	Y	Y	Y											Y				Y			Y						Y										
13046	A_23_P256487		Y	Y	Y				Y	Y																	Y																			
13063	A_23_P119006																					Y																								
13102	A_23_P61738																		Y	Y								Y																		
13108	A_23_P21401		Y	Y	Y		Y		Y	Y	Y	Y						Y									Y	Y	Y	Y																
13126	NA		Y	Y	Y	Y	Y	Y	Y	Y	Y	Y	Y	Y													Y	Y	Y	Y						Y										

13139	A_23_P62684				Y	Y		Y	Y	Y	Y	Y	Y	Y														Y		Y						Y										
13143	A_23_P16394				Y	Y		Y	Y	Y	Y	Y	Y	Y						Y								Y																		
13147	A_23_P418431					Y																																								
13158	A_23_P133257																			Y																										
13176	A_23_P210191		Y	Y	Y	Y	Y	Y	Y	Y	Y	Y							Y	Y							Y	Y	Y	Y																
13219	A_23_P342585										Y		Y	Y													Y			Y						Y										
13267	NA				Y	Y	Y	Y	Y	Y	Y	Y	Y	Y				Y	Y	Y				Y			Y	Y	Y	Y						Y										
13269	A_23_P39277				Y															Y							Y			Y						Y										
13337	A_23_P436145																	Y	Y	Y				Y																						
13411	A_23_P85874		Y		Y																						Y																			
13474	NA				Y	Y	Y	Y	Y	Y	Y	Y	Y	Y					Y								Y	Y	Y	Y						Y								Y		
13478	A_23_P158524		Y	Y	Y	Y	Y	Y	Y	Y	Y	Y						Y	Y	Y				Y			Y	Y	Y	Y																
13536	A_23_P56450				Y	Y		Y	Y	Y	Y	Y	Y	Y													Y	Y	Y	Y						Y								Y		
13585	A_23_P214432																											Y																		
13590	A_23_P33037																		Y																											
13592	A_23_P210158				Y	Y	Y	Y		Y	Y	Y						Y	Y			Y					Y																			
13604	A_23_P22052																										Y																			
13605	A_23_P54593																											Y																		
13622	A_23_P108105				Y	Y		Y		Y	Y	Y	Y	Y						Y							Y	Y	Y	Y						Y										
13657	A_23_P28307				Y	Y		Y		Y																																				
13680	A_23_P70614																			Y	Y		Y																							
13710	A_23_P124977																		Y	Y							Y																			
13719	A_23_P154621				Y	Y	Y	Y	Y	Y	Y	Y	Y	Y				Y	Y	Y				Y			Y	Y	Y	Y						Y										
13729	A_23_P162183					Y																																								
13733	NA				Y	Y		Y	Y	Y									Y	Y	Y	Y					Y	Y	Y	Y						Y										
13738	A_23_P259892				Y	Y		Y	Y	Y	Y	Y	Y	Y						Y							Y			Y						Y										
13744	A_23_P82800																													Y						Y										
13751	A_23_P251513				Y														Y			Y																								
13805	A_23_P253821								Y	Y																																				
13809	A_23_P74508				Y	Y	Y	Y		Y	Y	Y	Y	Y				Y	Y	Y	Y	Y	Y	Y	Y	Y	Y				Y		Y	Y	Y											
13838	A_23_P146679				Y																						Y																			
13845	A_23_P84646				Y	Y		Y		Y	Y	Y	Y	Y						Y										Y						Y										
13859	A_23_P218762		Y	Y	Y	Y	Y	Y	Y	Y	Y	Y	Y	Y	Y			Y	Y	Y				Y			Y	Y	Y	Y														Y		
13881	A_23_P84016					Y			Y	Y													Y							Y						Y										
13910	A_23_P161706					Y													Y	Y		Y	Y					Y																		
13917	A_23_P119407					Y												Y																												
13972	A_23_P155582				Y	Y		Y	Y	Y																		Y																		
13978	A_23_P392529		Y	Y	Y	Y	Y	Y	Y	Y	Y	Y	Y	Y													Y	Y	Y	Y						Y								Y		
13989	A_23_P5626				Y													Y		Y	Y	Y	Y				Y						Y													
14048	A_23_P86461																						Y																							
14158	A_23_P85109					Y																																								
14174	A_23_P158417		Y	Y	Y	Y	Y	Y	Y	Y	Y	Y															Y	Y	Y	Y						Y										
14217	A_23_P25534																	Y	Y	Y		Y	Y	Y																						
14260	A_23_P259237					Y																																								
14263	NA				Y								Y																	Y						Y										
14268	A_23_P205471					Y												Y		Y		Y																								
14334	A_23_P64310					Y																Y						Y																		
14395	A_23_P22639				Y																		Y				Y	Y	Y	Y						Y										
14398	A_23_P135797																													Y						Y										
14444	A_23_P131036		Y		Y																						Y																			
14458	A_23_P43932				Y	Y		Y		Y																	Y	Y	Y	Y																
14465	A_23_P101300										Y		Y	Y																Y						Y										
14472	A_23_P32795		Y	Y	Y		Y		Y	Y	Y	Y						Y	Y	Y				Y			Y	Y	Y	Y																
14503	A_23_P206526				Y														Y	Y		Y					Y																			
14537	A_23_P164156		Y						Y	Y																				Y						Y										
14572	A_23_P125301				Y	Y	Y	Y	Y	Y	Y	Y							Y	Y		Y					Y	Y	Y	Y						Y										
14615	A_23_P149615				Y	Y	Y	Y	Y	Y	Y	Y	Y	Y				Y	Y	Y	Y	Y	Y	Y	Y	Y	Y	Y	Y	Y	Y	Y														
14655	A_23_P63484					Y																																								
14669	A_23_P29215								Y	Y																	Y																			
14696	A_23_P433571		Y	Y	Y	Y	Y	Y	Y	Y	Y	Y						Y									Y																			
14843	A_23_P258114				Y	Y	Y	Y		Y	Y	Y	Y	Y					Y	Y		Y	Y				Y																			
14877	A_23_P147224		Y	Y	Y	Y	Y	Y	Y	Y	Y	Y						Y	Y	Y				Y			Y	Y	Y	Y																
14922	A_23_P111525				Y	Y	Y	Y	Y	Y	Y	Y	Y	Y				Y	Y	Y		Y	Y	Y			Y	Y	Y	Y						Y										
14927	NA				Y	Y	Y	Y	Y	Y	Y	Y	Y	Y					Y								Y	Y	Y	Y						Y										
14934	A_23_P57174				Y	Y	Y	Y	Y	Y	Y	Y	Y	Y						Y			Y				Y	Y	Y	Y						Y						Y				
14973	A_23_P324890										Y		Y	Y								Y	Y																							
14999	A_23_P135403				Y	Y	Y	Y		Y	Y	Y																Y																		
15001	A_23_P203961				Y														Y	Y																										
15008	A_23_P54983				Y																						Y																			
15024	A_23_P135568								Y	Y	Y	Y	Y	Y				Y		Y	Y	Y	Y							Y			Y			Y										
15030	A_23_P61071		Y	Y	Y	Y	Y	Y	Y	Y	Y	Y							Y	Y							Y																			
15039	A_23_P22603				Y																	Y	Y																							
15052	A_23_P78318																	Y	Y	Y				Y						Y						Y										
15055	A_23_P30346																										Y																			
15063	A_23_P207091																			Y		Y	Y																							
15071	A_23_P142597								Y	Y																																				
15082	A_23_P80583																			Y																										

15094	A_23_P151956		Y	Y	Y	Y	Y	Y	Y	Y	Y	Y	Y	Y						Y							Y	Y	Y	Y						Y										
15117	A_23_P66371								Y	Y																																				
15123	A_23_P124160					Y														Y								Y																		
15129	A_23_P44514																					Y	Y																							
15133	A_23_P69266																					Y																								
15146	A_23_P147735								Y	Y																				Y						Y										
15171	A_23_P47220				Y	Y		Y	Y	Y																		Y		Y						Y										
15185	A_23_P45144																						Y																							
15203	A_23_P258809					Y																																								
15210	A_23_P13535																			Y		Y	Y																							
15229	A_23_P32547				Y	Y		Y	Y	Y	Y	Y	Y	Y													Y	Y	Y	Y						Y								Y		
15255	A_23_P2032				Y	Y		Y	Y	Y								Y	Y	Y		Y	Y	Y				Y																		
15277	A_23_P22313																						Y																							
15284	A_23_P158605		Y		Y				Y	Y																	Y																			
15311	A_23_P38978		Y	Y	Y	Y	Y	Y		Y	Y	Y	Y	Y				Y	Y	Y		Y	Y	Y			Y	Y	Y	Y						Y										
15356	A_23_P84169																						Y																							
15374	A_23_P61623				Y																																									
15392	A_23_P212131					Y	Y		Y	Y	Y	Y																Y		Y						Y										
15449	A_23_P72252		Y	Y	Y		Y		Y	Y	Y	Y						Y									Y	Y	Y	Y																
15478	A_23_P106559		Y		Y	Y		Y	Y	Y										Y			Y				Y	Y	Y	Y						Y										
15533	A_23_P252360																						Y																							
15621	A_23_P88067																													Y						Y										
15651	A_23_P70241					Y														Y								Y																		
15653	A_23_P43649								Y	Y								Y		Y										Y						Y										
15668	A_23_P113101				Y																																									
15788	A_23_P112794				Y													Y	Y	Y		Y	Y	Y																						
15808	A_23_P67135				Y	Y		Y	Y	Y								Y	Y	Y		Y	Y	Y			Y																			
15854	A_23_P103651																										Y																			
15869	A_23_P147334																										Y																			
15870	A_23_P168456																										Y																			
15886	A_23_P106814				Y				Y	Y	Y	Y	Y	Y													Y	Y	Y	Y						Y										
15920	A_23_P148081				Y	Y		Y	Y	Y										Y							Y	Y	Y	Y						Y										
15963	A_23_P113453				Y	Y		Y	Y	Y	Y	Y	Y	Y																Y						Y										
15976	A_23_P257445		Y	Y	Y	Y	Y	Y	Y	Y	Y	Y	Y	Y				Y	Y	Y		Y	Y	Y			Y	Y	Y	Y	Y	Y	Y	Y	Y	Y										
15983	A_23_P75719																						Y																							
16029	A_23_P11017		Y	Y	Y	Y		Y	Y	Y																		Y		Y						Y										
16046	A_23_P131445				Y													Y																												
16048	A_23_P148236		Y	Y	Y	Y	Y	Y	Y	Y	Y	Y	Y	Y				Y	Y	Y		Y		Y			Y	Y	Y	Y																
16072	A_23_P159527																				Y																									
16088	A_23_P73083		Y	Y	Y	Y		Y	Y	Y	Y	Y	Y	Y				Y	Y	Y		Y		Y			Y	Y	Y	Y																
16138	A_23_P206718				Y	Y		Y		Y													Y				Y	Y	Y	Y																
16163	A_23_P211468				Y				Y	Y									Y																											
16188	A_23_P116337				Y	Y		Y	Y	Y									Y	Y			Y				Y			Y						Y										
16254	A_23_P209251					Y												Y				Y																								
16283	A_23_P74368																			Y	Y																									
16316	A_23_P82783				Y	Y		Y		Y																	Y	Y	Y	Y						Y										
16335	A_23_P343899		Y	Y	Y				Y	Y																	Y			Y						Y										
16396	A_23_P105105				Y				Y	Y																	Y			Y						Y										
16405	A_23_P75585																						Y				Y																			
16437	A_23_P146981		Y	Y	Y	Y	Y	Y	Y	Y	Y	Y	Y	Y				Y	Y	Y	Y	Y	Y	Y	Y	Y	Y	Y	Y	Y	Y	Y	Y	Y	Y	Y										
16454	A_23_P259561		Y	Y	Y	Y	Y	Y	Y	Y	Y	Y							Y	Y							Y	Y	Y	Y																
16474	A_23_P17587																										Y			Y						Y										
16541	A_23_P207541				Y	Y	Y	Y		Y	Y	Y						Y		Y		Y					Y			Y						Y										
16563	A_23_P44886								Y	Y																																				
16580	A_23_P121320					Y																																								
16589	A_23_P19847								Y	Y																																				
16642	NA				Y	Y	Y	Y	Y	Y	Y	Y	Y	Y													Y	Y	Y	Y						Y										
16664	A_23_P84795		Y	Y	Y	Y		Y	Y	Y	Y	Y	Y	Y					Y	Y							Y																			
16684	A_23_P127809					Y																																								
16699	A_23_P80200				Y																						Y																			
16703	A_23_P49949				Y																						Y			Y						Y										
16713	A_23_P170110																				Y																									
16714	A_23_P9648				Y													Y									Y																			
16737	A_23_P95599				Y	Y	Y	Y		Y	Y	Y						Y	Y	Y		Y	Y	Y			Y	Y	Y	Y						Y										
16825	A_23_P25905				Y																																									
16839	A_23_P158897				Y																						Y																			
16846	A_23_P1707				Y	Y		Y		Y													Y				Y	Y	Y	Y																
16870	A_23_P28906				Y	Y	Y	Y	Y	Y	Y	Y	Y	Y						Y			Y				Y	Y	Y	Y						Y										
16873	A_23_P144211																			Y																										
16938	A_23_P147505				Y																																									
16950	A_23_P10605																			Y	Y		Y																							
17036	NA				Y	Y	Y	Y	Y	Y	Y	Y	Y	Y				Y	Y	Y	Y	Y	Y	Y	Y	Y	Y	Y	Y	Y	Y	Y	Y			Y										
17113	A_23_P384355		Y	Y																																										
17121	A_23_P61039		Y	Y	Y	Y	Y	Y	Y	Y	Y	Y						Y	Y	Y				Y			Y	Y	Y	Y																
17122	A_23_P120606				Y	Y		Y	Y	Y																	Y	Y	Y	Y																
17139	A_23_P112957		Y	Y	Y	Y	Y	Y	Y	Y	Y	Y						Y		Y							Y																			
17162	A_23_P217452				Y				Y	Y																	Y	Y	Y	Y						Y										

17163	A_23_P170719				Y				Y	Y			Y														Y	Y	Y	Y						Y										
17184	A_23_P99103																										Y	Y	Y	Y																
17245	A_23_P68282				Y	Y		Y		Y																																				
17251	A_23_P122600								Y	Y																																				
17252	A_23_P156767				Y																						Y																			
17262	A_23_P102183		Y	Y																																										
17279	A_23_P65843				Y	Y		Y		Y																																				
17294	A_23_P415532					Y																					Y	Y	Y	Y						Y										
17301	A_23_P136961				Y	Y		Y		Y										Y			Y				Y			Y						Y										
17348	A_23_P215696								Y	Y											Y																									
17353	A_23_P159011				Y	Y	Y	Y	Y	Y	Y	Y	Y	Y				Y	Y	Y	Y	Y	Y	Y	Y	Y	Y	Y	Y	Y	Y	Y	Y	Y	Y	Y								Y		
17362	A_23_P130187		Y		Y	Y	Y	Y	Y	Y	Y	Y	Y	Y				Y	Y	Y	Y	Y	Y	Y	Y	Y	Y	Y	Y	Y	Y	Y	Y	Y	Y	Y										
17363	A_23_P122204				Y	Y		Y	Y	Y																		Y		Y						Y										
17379	A_23_P96017				Y				Y	Y	Y	Y	Y	Y				Y		Y		Y					Y			Y						Y										
17385	A_23_P10525				Y	Y		Y		Y																	Y	Y	Y	Y																
17392	A_23_P143102				Y	Y		Y		Y													Y				Y																			
17454	A_23_P364953				Y																						Y																			
17485	A_23_P69891																		Y			Y																								
17501	A_23_P259243																													Y						Y										
17510	A_23_P50129		Y	Y																																										
17512	A_23_P381327					Y																																								
17528	A_23_P89680				Y	Y		Y		Y										Y		Y						Y		Y						Y										
17551	A_23_P84305				Y	Y		Y	Y	Y										Y	Y		Y				Y			Y						Y										
17619	A_23_P62122				Y	Y	Y	Y		Y	Y	Y							Y	Y		Y					Y																			
17627	A_23_P108614				Y	Y		Y		Y										Y			Y				Y																			
17635	A_23_P140993																	Y	Y									Y																		
17682	A_23_P18871																					Y																								
17715	NA				Y	Y	Y	Y	Y	Y	Y	Y	Y	Y													Y	Y	Y	Y						Y										
17734	A_23_P83521				Y														Y	Y																										
17749	A_23_P20372					Y														Y								Y																		
17754	A_23_P123645		Y	Y	Y	Y		Y	Y	Y								Y	Y	Y		Y	Y	Y			Y	Y	Y	Y						Y										
17769	A_23_P37527				Y															Y																										
17780	NA				Y	Y	Y	Y	Y	Y	Y	Y	Y	Y				Y	Y								Y	Y	Y	Y						Y										
17827	A_23_P208579					Y																																								
17845	A_23_P215163								Y	Y																				Y						Y										
17949	A_23_P124334					Y			Y	Y								Y	Y	Y				Y			Y																			
18003	A_23_P206826				Y	Y		Y		Y										Y							Y																			
18024	A_23_P140654				Y															Y							Y																			
18065	A_23_P307392																						Y																							
18085	A_23_P93709					Y																						Y		Y						Y										
18103	A_23_P78975				Y	Y		Y		Y																		Y		Y						Y										
18140	A_23_P158868		Y	Y	Y	Y	Y	Y	Y	Y	Y	Y	Y	Y				Y	Y	Y		Y		Y			Y	Y	Y	Y						Y										
18155	A_23_P147591				Y																						Y			Y						Y										
18177	A_23_P91369								Y	Y	Y	Y	Y	Y					Y	Y		Y	Y				Y	Y	Y	Y						Y										
18241	A_23_P124278																													Y						Y										
18272	A_23_P46718				Y				Y	Y			Y					Y		Y							Y			Y						Y										
18280	A_23_P32568																			Y																										
18307	A_23_P257434				Y		Y		Y	Y	Y	Y								Y																										
18313	A_23_P107383																											Y																		
18316	A_23_P72941																	Y																												
18332	A_23_P7719				Y	Y		Y	Y	Y																	Y																			
18361	NA				Y	Y	Y	Y	Y	Y	Y	Y	Y	Y					Y	Y							Y	Y	Y	Y						Y										
18382	A_23_P402745				Y				Y	Y																				Y						Y										
18413	A_23_P209459				Y	Y		Y	Y	Y																		Y		Y						Y										
18423	A_23_P16496		Y	Y	Y													Y		Y		Y					Y	Y	Y	Y																
18426	A_23_P3055																					Y																								
18434	A_23_P157835				Y	Y	Y	Y		Y	Y	Y								Y							Y	Y	Y	Y						Y										
18492	A_23_P200815				Y																																									
18509	A_23_P218951					Y																	Y							Y						Y										
18512	A_23_P89841		Y	Y	Y	Y	Y	Y	Y	Y	Y	Y	Y	Y				Y	Y	Y	Y	Y	Y	Y	Y	Y	Y	Y	Y	Y	Y	Y				Y								Y		
18608	A_23_P12628					Y																																								
18634	A_23_P24688								Y	Y																				Y						Y										
18648	A_23_P22672				Y	Y	Y	Y	Y	Y	Y	Y	Y	Y				Y		Y		Y					Y	Y	Y	Y						Y						Y				
18676	A_23_P21189				Y		Y		Y	Y	Y	Y	Y	Y				Y	Y	Y				Y			Y																			
18678	A_23_P132138					Y																																								
18697	A_23_P207049		Y		Y															Y							Y																			
18715	A_23_P134353		Y																																											
18767	A_23_P10753				Y		Y				Y								Y								Y																			
18819	A_23_P26281				Y	Y		Y	Y	Y																		Y		Y						Y										
18842	A_23_P124314																	Y	Y	Y		Y		Y																						
18857	A_23_P14403				Y	Y		Y	Y	Y	Y	Y	Y	Y													Y	Y	Y	Y						Y										
18911	A_23_P117987				Y	Y		Y		Y																	Y	Y	Y	Y																
18923	A_23_P89851				Y	Y	Y	Y	Y	Y	Y	Y	Y	Y				Y		Y		Y					Y																			
18942	A_23_P365117										Y		Y	Y																Y						Y										
18982	A_23_P21282				Y																																									
19000	A_23_P119153		Y	Y	Y	Y		Y	Y	Y	Y	Y	Y	Y													Y	Y	Y	Y																
19024	A_23_P113762								Y	Y	Y	Y	Y	Y																Y						Y										

19036	A_23_P164267		Y	Y	Y		Y		Y	Y	Y	Y	Y	Y				Y	Y	Y				Y			Y	Y	Y	Y																
19042	A_23_P105900				Y	Y		Y	Y	Y										Y		Y	Y				Y	Y	Y	Y						Y										
19056	A_23_P127771				Y	Y	Y	Y	Y	Y	Y	Y	Y	Y					Y								Y	Y	Y	Y						Y										
19070	A_23_P300563								Y	Y																																				
19114	A_23_P162891				Y	Y		Y		Y										Y			Y				Y			Y						Y										
19115	A_23_P90780				Y	Y		Y	Y	Y										Y		Y	Y				Y	Y	Y	Y	Y	Y	Y	Y	Y	Y										
19146	A_23_P2762					Y																						Y																		
19227	A_23_P165811				Y	Y		Y		Y								Y																												
19267	A_23_P37015					Y			Y	Y								Y		Y		Y	Y							Y						Y										
19271	A_23_P29411				Y	Y		Y		Y																		Y																		
19290	A_23_P201686																						Y																							
19302	A_23_P73820				Y	Y	Y	Y	Y	Y	Y	Y	Y	Y				Y	Y	Y				Y			Y	Y	Y	Y						Y								Y		
19310	A_23_P9854		Y		Y	Y	Y	Y	Y	Y	Y	Y						Y	Y	Y				Y			Y	Y	Y	Y																
19317	A_23_P32661				Y	Y	Y	Y	Y	Y	Y	Y	Y	Y					Y	Y							Y	Y	Y	Y																
19410	A_23_P364355				Y																						Y																			
19511	A_23_P24666				Y	Y		Y	Y	Y																	Y	Y	Y	Y						Y										
19559	A_23_P255403																													Y						Y										
19575	A_23_P91615				Y	Y	Y	Y	Y	Y	Y	Y	Y	Y				Y	Y	Y	Y	Y	Y	Y	Y	Y	Y	Y	Y	Y	Y	Y				Y										
19643	A_23_P134376					Y																																								
19660	A_23_P95244				Y																							Y																		
19668	A_23_P29907																										Y																			
19697	A_23_P48404								Y	Y																																				
19698	A_23_P74993																		Y																											
19728	A_23_P170925				Y	Y		Y	Y	Y	Y	Y	Y	Y						Y	Y	Y					Y																			
19756	A_23_P212626					Y																																								
19789	A_23_P169955				Y																																									
19792	A_23_P212782					Y													Y																											
19797	A_23_P136392				Y	Y	Y	Y		Y	Y	Y	Y	Y				Y		Y	Y	Y					Y	Y	Y	Y																
19814	A_23_P61766								Y	Y																																				
19816	A_23_P56872		Y	Y	Y	Y	Y	Y	Y	Y	Y	Y						Y	Y	Y				Y			Y																			
19820	A_23_P258050				Y	Y	Y	Y	Y	Y	Y	Y						Y	Y	Y	Y	Y	Y	Y	Y	Y	Y	Y	Y	Y	Y	Y	Y			Y								Y		
19870	A_23_P217580				Y																						Y																			
19871	A_23_P68137				Y	Y		Y		Y	Y	Y	Y	Y						Y								Y		Y						Y										
19887	A_23_P87421				Y	Y		Y	Y	Y								Y	Y	Y		Y	Y	Y			Y			Y						Y										
19905	A_23_P130496				Y																						Y																			
19929	A_23_P84974																	Y		Y																										
19941	A_23_P117971				Y	Y		Y	Y	Y																		Y		Y						Y										
19949	A_23_P89529				Y	Y		Y	Y	Y										Y	Y	Y	Y				Y	Y	Y	Y																
19951	A_23_P115032				Y	Y	Y	Y	Y	Y	Y	Y															Y	Y	Y	Y						Y										
19972	A_23_P144535				Y	Y	Y	Y		Y	Y	Y															Y	Y	Y	Y						Y								Y		
20003	A_23_P91131				Y	Y	Y	Y		Y	Y	Y	Y	Y													Y	Y	Y	Y						Y										
20015	A_23_P10104				Y	Y		Y	Y	Y									Y	Y		Y	Y				Y																			
20026	A_23_P111506																			Y	Y	Y																								
20027	A_23_P102822				Y																																									
20038	A_23_P64529					Y																Y						Y																		
20049	A_23_P206253																		Y	Y	Y	Y					Y																			
20075	A_23_P158913		Y	Y	Y	Y	Y	Y	Y	Y	Y	Y	Y	Y					Y	Y		Y	Y				Y	Y	Y	Y						Y										
20182	A_23_P150520				Y	Y		Y		Y										Y			Y				Y	Y	Y	Y																
20215	A_23_P112841																			Y		Y																								
20241	A_23_P10663					Y			Y	Y									Y	Y							Y	Y	Y	Y						Y										
20250	A_23_P72448								Y	Y																																				
20287	A_23_P129645				Y																																									
20290	A_23_P162024					Y																						Y																		
20320	A_23_P250089		Y		Y	Y	Y	Y	Y	Y	Y	Y							Y	Y							Y	Y	Y	Y						Y										
20324	A_23_P26072								Y	Y								Y		Y		Y																								
20358	A_23_P26745				Y	Y	Y	Y	Y	Y	Y	Y								Y		Y	Y				Y																			
20380	A_23_P215431																											Y																		
20404	A_23_P142113					Y																																								
20423	A_23_P254288				Y	Y		Y	Y	Y								Y	Y	Y		Y		Y			Y	Y	Y	Y																
20465	A_23_P253701				Y														Y	Y							Y																			
20547	A_23_P52848				Y	Y	Y	Y	Y	Y	Y	Y						Y	Y	Y		Y	Y	Y				Y		Y						Y										
20549	A_23_P86689				Y																						Y																			
20611	A_23_P254233		Y	Y	Y		Y		Y	Y	Y	Y						Y	Y								Y																			
20629	A_23_P72169				Y				Y	Y	Y	Y	Y	Y						Y		Y	Y				Y			Y						Y										
20670	A_23_P114048																											Y																		
20671	A_23_P34818																			Y																										
20672	A_23_P125033				Y								Y							Y		Y	Y				Y																			
20678	A_23_P136523				Y	Y		Y	Y	Y										Y			Y				Y	Y	Y	Y						Y										
20686	A_23_P121234								Y	Y								Y	Y	Y		Y		Y																						
20710	A_23_P302078								Y	Y			Y																	Y						Y										
20735	NA				Y				Y	Y	Y	Y	Y	Y													Y			Y						Y										
20759	A_23_P72330				Y		Y		Y	Y	Y	Y							Y								Y																			
20811	A_23_P21961																		Y																											
20841	A_23_P217727				Y	Y		Y		Y																	Y	Y	Y	Y																
20871	A_23_P122842								Y	Y																																				
20893	A_23_P133307				Y	Y	Y	Y	Y	Y	Y	Y	Y	Y					Y	Y			Y				Y	Y	Y	Y						Y										
20895	A_23_P202782																			Y			Y																							

20932	A_23_P8124																											Y																		
20964	A_23_P336565				Y													Y	Y	Y		Y	Y	Y			Y				Y		Y	Y	Y											
20976	A_23_P169587				Y	Y	Y	Y		Y	Y	Y							Y	Y		Y	Y				Y	Y	Y	Y																
21016	A_23_P72583																											Y																		
21025	A_23_P209179					Y																						Y																		
21029	A_23_P108427				Y	Y	Y	Y	Y	Y	Y	Y						Y	Y	Y		Y	Y	Y			Y	Y	Y	Y	Y	Y	Y	Y	Y	Y										
21103	A_23_P31164																	Y	Y	Y		Y		Y			Y			Y						Y										
21107	A_23_P39983																	Y	Y								Y																			
21127	A_23_P134704				Y																						Y																			
21217	A_23_P434268				Y	Y	Y	Y	Y	Y	Y	Y	Y	Y				Y	Y	Y	Y	Y		Y			Y	Y	Y	Y						Y										
21255	A_23_P113738				Y	Y	Y	Y	Y	Y	Y	Y	Y	Y				Y		Y			Y				Y			Y						Y										
21264	A_23_P28514																	Y												Y						Y										
21323	A_23_P47826																													Y						Y										
21340	A_23_P163962																	Y	Y											Y						Y										
21345	A_23_P86598				Y		Y				Y							Y									Y			Y						Y										
21357	A_23_P169668																													Y						Y										
21384	A_23_P435032				Y	Y		Y		Y										Y							Y																			
21420	A_23_P113819				Y																																									
21455	A_23_P9270																											Y		Y						Y										
21467	A_23_P84246				Y	Y		Y	Y	Y																		Y		Y						Y										
21530	A_23_P207802		Y	Y	Y														Y																											
21559	A_23_P96399																													Y						Y										
21564	A_23_P65644								Y	Y																	Y			Y						Y										
21602	A_23_P165770				Y	Y		Y	Y	Y																	Y	Y	Y	Y																
21647	A_23_P87810				Y	Y	Y	Y	Y	Y	Y	Y	Y	Y				Y	Y	Y		Y		Y			Y																			
21691	A_23_P311520																		Y	Y		Y	Y																							
21708	A_23_P340640																											Y		Y						Y										
21728	A_23_P57622					Y																																								
21772	A_23_P389250				Y				Y	Y									Y			Y																								
21812	A_23_P66364				Y																						Y																			
21822	A_23_P165819				Y	Y		Y	Y	Y																																				
21833	A_23_P130326																										Y																			
21839	A_23_P22808																	Y																												
21871	A_23_P64184		Y	Y	Y	Y		Y		Y																	Y																			
21895	A_23_P1153																										Y																			
21898	A_23_P303293																			Y	Y																									
21915	A_23_P55036		Y	Y	Y	Y		Y	Y	Y								Y		Y							Y																			
21951	A_23_P143811																										Y			Y						Y										
21990	A_23_P70492																		Y								Y			Y						Y										
22042	A_23_P95406				Y						Y		Y	Y									Y																							
22043	A_23_P147577								Y	Y																																				
22056	A_23_P59094		Y	Y																										Y						Y										
22087	A_23_P159631																				Y																									
22098	A_23_P92811								Y	Y																	Y	Y	Y	Y																
22101	A_23_P21804				Y	Y		Y	Y	Y								Y					Y					Y		Y						Y										
22171	A_23_P147485		Y	Y	Y	Y	Y	Y	Y	Y	Y	Y	Y	Y				Y	Y	Y		Y		Y			Y	Y	Y	Y						Y										
22211	A_23_P72319		Y	Y	Y	Y	Y	Y	Y	Y	Y	Y						Y	Y	Y				Y			Y	Y	Y	Y																
22223	A_23_P57836				Y	Y		Y	Y	Y								Y	Y	Y		Y	Y	Y			Y	Y	Y	Y						Y										
22227	A_23_P74203				Y				Y	Y	Y	Y	Y	Y													Y	Y	Y	Y						Y										
22231	NA				Y	Y	Y	Y	Y	Y	Y	Y	Y	Y				Y	Y	Y				Y			Y	Y	Y	Y						Y										
22248	A_23_P214273					Y														Y								Y																		
22259	A_23_P20573				Y	Y	Y	Y		Y	Y	Y						Y	Y	Y		Y	Y	Y			Y	Y	Y	Y																
22281	A_23_P81273					Y																					Y																			
22356	A_23_P203043				Y																																									
22377	A_23_P14432				Y	Y	Y	Y	Y	Y	Y	Y							Y	Y		Y					Y	Y	Y	Y																
22433	A_23_P161835																		Y																											
22474	A_23_P87931				Y													Y		Y																										
22512	A_23_P8108		Y		Y	Y	Y	Y	Y	Y	Y	Y							Y	Y		Y	Y				Y	Y	Y	Y	Y	Y	Y	Y	Y	Y										
22551	A_23_P32966								Y	Y									Y	Y		Y	Y																							
22559	A_23_P21882				Y				Y	Y	Y	Y	Y	Y								Y					Y																	Y		
C10768.8	A_23_P123234					Y														Y	Y	Y	Y																							
C11176.3	A_23_P48637		Y	Y	Y																							Y																		
C8482.2	A_23_P258668				Y																																									

Figure S5.1. The prognostic prediction of E2F1(7852), MYB(5586), MYBL1(C11830), MYBL2(10757) and XBP1(10024) in different cohorts (90A cohort, 181A cohort) by Kaplan-Meier survival analysis.
Some of analyses have missing data to cause the final sample number less than 90 or 181. 7852, 5586, C11830, 10757 and 10024 are the Agilent feature number for *E2F1*, *MYB*, *MYBL1*, *MYBL2* and *XBP1*, respectively.

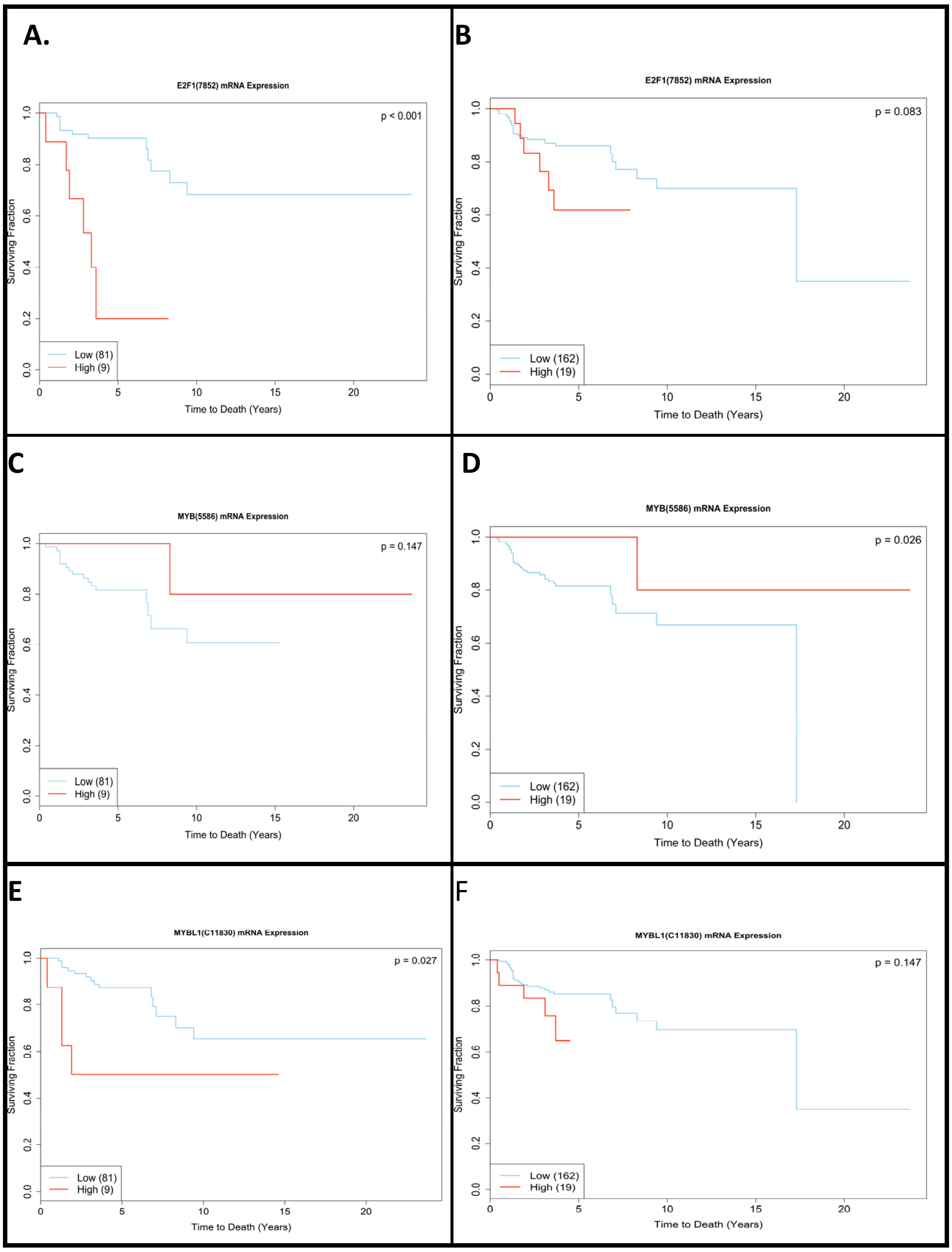

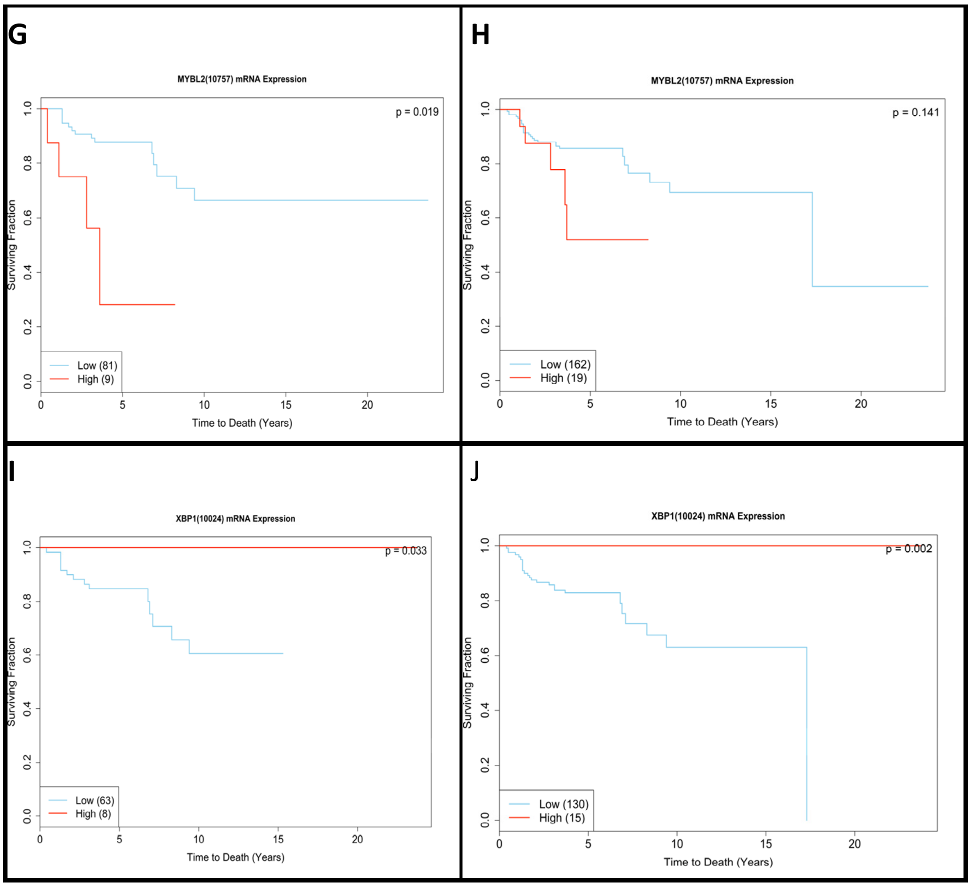
G
MYBL2(10757) mRNA Expression
p = 0.019
Surviving Fraction
Time to Death (Years)
Low (81)
High (9)
H
MYBL2(10757) mRNA Expression
p = 0.141
Surviving Fraction
Time to Death (Years)
Low (162)
High (19)
I
XBP1(10024) mRNA Expression
p = 0.033
Surviving Fraction
Time to Death (Years)
Low (63)
High (8)
J
XBP1(10024) mRNA Expression
p = 0.002
Surviving Fraction
Time to Death (Years)
Low (130)
High (15)

Figure S5.2. The prognostic prediction of ARNT2(3742), ARNT2(3187), POU2F1(947), POU2F1(11991), and SALL2(3096) in different cohorts (90A cohort, 181A cohort) by Kaplan-Meier survival analysis. 3742, 3187, 947, 11991 and 3096 are the Agilent feature number for *ARNT2*, a *ARNT2* variant, *POU2F1*, a *POU2F1* variant and *SALL2*, respectively.

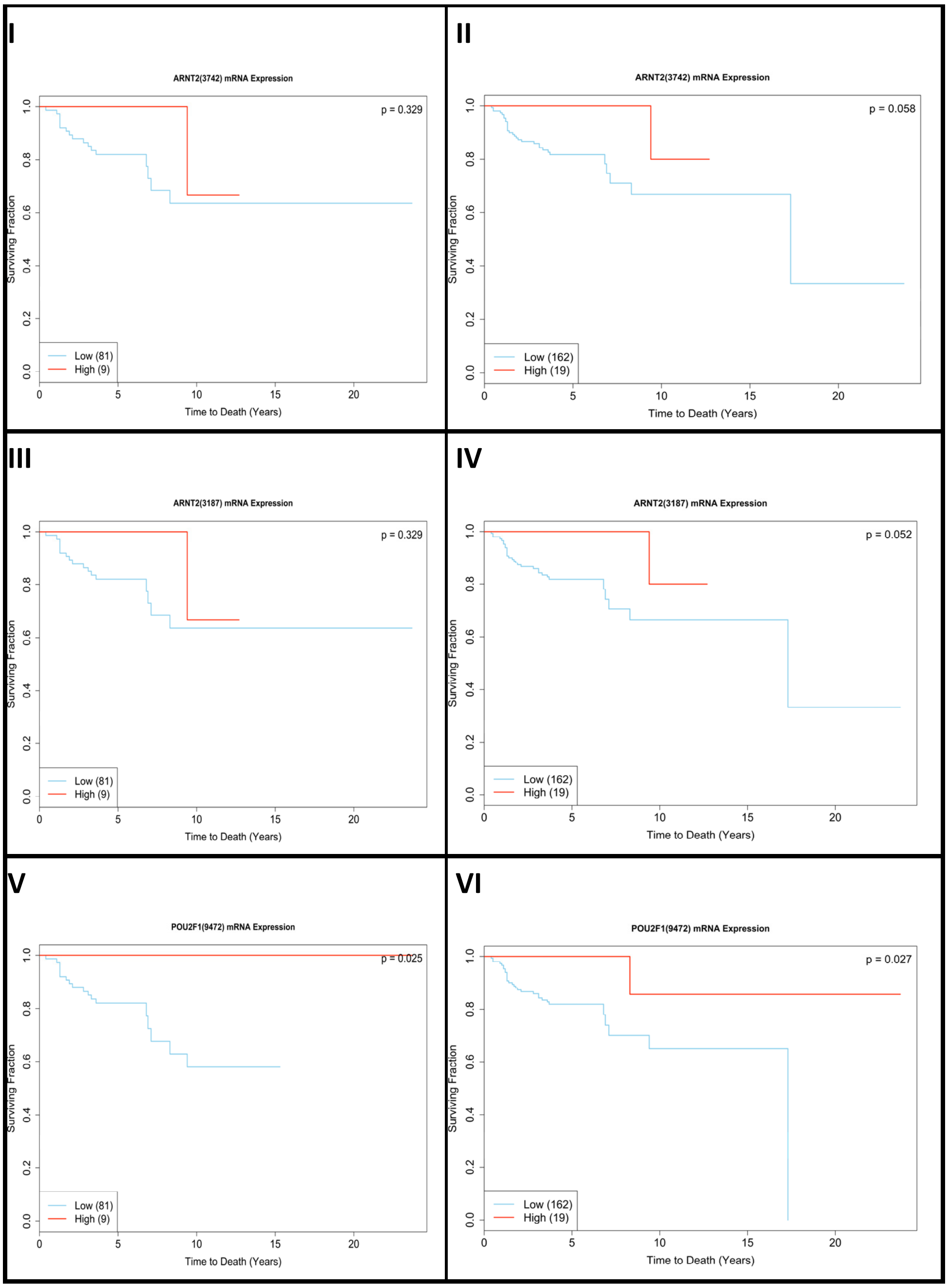

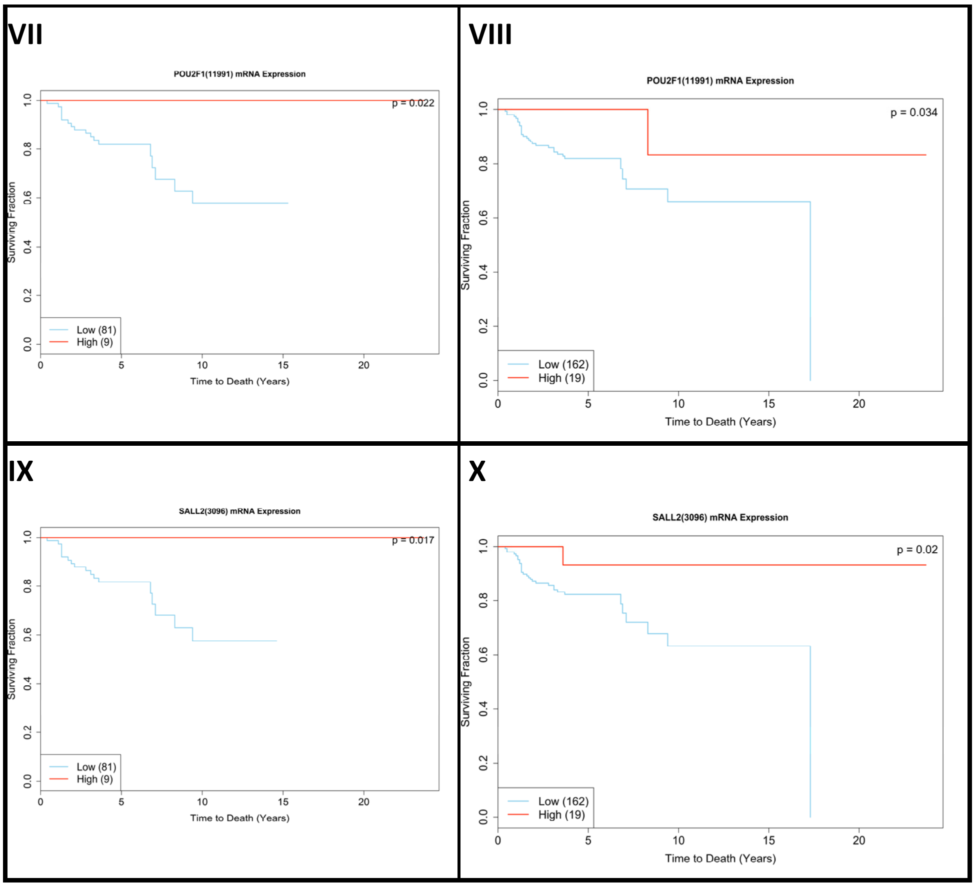
VII
POU2F1(11991) mRNA Expression
p = 0.022
Surviving Fraction
Time to Death (Years)
Low (81)
High (9)
VIII
POU2F1(11991) mRNA Expression
p = 0.034
Surviving Fraction
Time to Death (Years)
Low (162)
High (19)
IX
SALL2(3096) mRNA Expression
p = 0.017
Surviving Fraction
Time to Death (Years)
Low (81)
High (9)
X
SALL2(3096) mRNA Expression
p = 0.02
Surviving Fraction
Time to Death (Years)
Low (162)
High (19)

Table S5.1. The ANOVA results of 41 gene signature in 90A cohort. The relevant clinicopathological characteristics for MYB and/or ARNT2 are bolded. The p values of ANOVA tests to be less or equal to 0.05 are high-lighted with light blue. The gene symbols with no clinical significance are high-lighted with light yellow.

FeatureNum	GeneName	**PR**	HER	stage	LYM	**LVI**	Age	**Grade**	**TF**	**NP**	MC	**Size**	**LNM**	NTn90A
21074	ABAT	0.41	0.11	0.43	0.24	0.52	0.86	0.00	0.05	0.00	0.07	0.13	0.21	0.00
3069	ACOT7	0.25	0.22	0.35	0.61	0.40	0.30	0.24	0.73	0.44	0.07	0.01	0.27	0.00
6691	ANAPC4	0.18	0.76	1.00	0.51	0.02	0.66	0.02	0.13	0.15	0.25	0.16	0.25	0.00
2654	APOM	0.02	0.60	0.04	0.53	0.11	0.10	0.00	0.06	0.02	0.01	0.00	0.02	0.04
10396	BC009926	0.52	0.61	0.07	0.45	0.33	0.54	0.00	0.08	0.10	0.24	0.22	0.05	0.18
8570	CATSPER2	0.26	0.04	0.85	0.84	0.02	0.61	0.01	0.20	0.01	0.40	0.94	0.41	0.93
956	CCDC124	0.15	0.13	0.85	0.25	0.10	0.98	0.47	0.75	0.63	0.35	0.05	0.36	0.01
1509	CR621710	0.21	0.88	0.74	0.16	0.40	0.99	0.45	0.94	0.48	0.31	0.60	0.26	0.62
16475	DUSP7	0.96	0.27	0.92	0.22	0.07	0.76	0.05	0.39	0.24	0.02	0.52	0.15	0.01
17698	EIF5A	0.19	0.01	0.43	0.45	0.14	0.63	0.57	0.78	0.58	0.75	0.01	0.36	0.00
15762	ENST00000370040	0.69	0.16	0.11	0.48	0.01	0.58	0.00	0.02	0.01	0.34	0.32	0.09	0.03
20037	GAPDH	0.67	0.13	0.62	0.79	0.08	0.22	0.06	0.39	0.15	0.61	0.41	0.67	0.00
1039	GNAI2	0.18	0.04	0.40	0.69	0.02	0.65	0.10	0.31	0.33	0.24	0.12	0.61	0.01
21198	GNB2	0.16	0.01	0.34	0.73	0.16	0.71	0.19	0.68	0.27	0.73	0.03	0.51	0.00
714	IQCK	0.35	0.10	0.23	0.90	0.17	0.97	0.06	0.03	0.04	0.47	0.40	0.26	0.55
3891	MAF1	0.23	0.06	0.60	0.58	0.05	0.86	0.18	0.79	0.17	0.63	0.04	0.50	0.02
5918	MAP1S	0.17	0.01	0.52	0.39	0.08	0.72	0.51	0.59	0.58	0.36	0.02	0.65	0.00
18840	NFKBIL2	0.05	0.01	0.42	0.34	0.16	0.33	0.40	0.94	0.15	0.89	0.01	0.24	0.00
20917	NTN4	0.29	0.21	0.47	0.11	0.06	0.90	0.01	0.04	0.01	0.16	0.44	0.24	0.00
20295	PAH	0.67	0.21	0.96	0.90	0.00	0.40	0.01	0.14	0.14	0.76	0.97	0.72	0.00
22434	PCBP3	0.51	0.58	0.55	0.19	0.37	0.97	0.65	1.00	0.23	0.07	0.51	0.25	0.69
17776	PICK1	0.80	0.01	0.15	0.64	0.26	0.65	0.66	0.73	0.55	0.83	0.01	0.36	0.01
19411	PKMYT1	0.14	0.32	0.40	0.88	0.00	0.45	0.02	0.65	0.07	0.07	0.01	0.09	0.00
10998	PMS2CL	0.58	0.98	0.33	0.83	0.19	0.07	0.01	0.10	0.21	0.25	0.93	0.14	0.00
9472	POU2F1	0.29	0.05	0.54	0.82	0.01	0.25	0.37	0.24	0.54	0.13	0.05	0.16	0.39
11991	POU2F1	0.26	0.15	0.64	0.67	0.04	0.30	0.47	0.27	0.61	0.05	0.08	0.16	0.51
20014	PPP1R9A	0.72	0.24	0.46	0.98	0.01	0.04	0.12	0.42	0.26	0.19	0.16	0.35	0.99
4681	RAB42	0.26	0.80	0.34	0.77	0.04	0.66	0.01	0.23	0.05	0.72	0.64	0.04	0.00
3073	RANGAP1	0.28	0.02	0.49	0.27	0.24	0.89	0.24	0.73	0.53	0.78	0.01	0.17	0.00
3096	SALL2	0.29	0.54	0.07	0.04	0.01	0.37	0.01	0.01	0.01	0.09	0.14	0.00	0.00
7939	SLC25A1	0.55	0.64	0.59	0.32	0.04	0.53	0.12	0.50	0.44	0.77	0.01	0.13	0.11
9673	STK36	0.10	0.69	0.16	0.15	0.06	0.12	0.14	0.03	0.48	0.75	0.13	0.08	0.41
17750	TBC1D9	0.94	0.32	0.30	0.90	0.10	0.93	0.09	0.38	0.80	0.52	0.97	0.37	0.50
11119	TGM2	0.31	0.00	0.91	0.89	0.08	0.93	0.10	0.50	0.03	0.95	0.44	0.96	0.00
10109	THSD4	0.45	0.48	0.23	0.13	0.82	0.12	0.24	0.84	0.48	0.88	0.76	0.03	0.69
4051	TMC5	0.14	0.02	0.42	0.35	0.28	0.70	0.25	0.04	0.17	0.77	0.42	0.28	0.31
13619	TOMM40	0.39	0.03	0.56	0.55	0.06	0.88	0.29	0.62	0.25	0.72	0.01	0.46	0.00
6481	TTC19	0.08	0.45	0.68	0.73	0.20	0.73	0.00	0.03	0.12	0.11	0.43	0.22	0.00
10024	XBP1	0.98	0.09	0.10	0.84	0.03	0.58	0.01	0.62	0.00	0.82	0.11	0.45	0.01
7164	ZNF228	0.67	0.27	0.96	0.77	0.00	0.20	0.04	0.45	0.67	0.20	0.80	0.72	0.00
11969	ZNF598	0.64	0.13	0.40	0.50	0.21	0.46	0.32	0.67	0.37	0.72	0.15	0.61	0.00

Table S5.2. The ANOVA results of 41 gene signature in 181A cohort. The relevant clinicopathological characteristics for MYB and/or ARNT2 are bolded. The p values of ANOVA tests to be less or equal to 0.05 are high-lighted with light blue. The gene symbols with no clinical significance are high-lighted with light yellow.

FeatureNum	GeneName	**PR**	**HER**	stage	LYM	LVI	Age	**Grade**	**TF**	**NP**	**MC**	**Size**	LNM	NTn181A
21074	ABAT	0.00	0.50	0.57	0.08	0.26	0.15	0.00	0.00	0.00	0.00	0.75	0.29	0.02
3069	ACOT7	0.15	0.12	0.54	0.70	0.08	0.28	0.05	0.13	0.04	0.05	0.01	0.36	0.00
4259	ANAPC4	0.03	0.40	0.07	0.11	0.69	0.64	0.01	0.11	0.07	0.01	0.97	0.05	0.00
6691	ANAPC4	0.00	0.04	0.03	0.02	0.71	0.54	0.00	0.15	0.01	0.01	0.94	0.01	0.00
2654	APOM	0.00	0.19	0.53	0.20	0.17	0.64	0.00	0.00	0.00	0.00	0.14	0.07	0.00
10396	BC009926	0.00	0.02	0.75	0.08	0.15	0.41	0.00	0.00	0.00	0.00	0.69	0.31	0.23
8570	CATSPER2	0.00	0.38	0.63	0.26	0.79	0.17	0.00	0.00	0.00	0.00	0.83	0.41	0.11
956	CCDC124	0.01	0.04	0.26	0.52	0.17	0.30	0.15	0.50	0.13	0.69	0.29	0.33	0.00
1509	CR621710	0.00	0.01	0.02	0.04	0.16	0.49	0.00	0.11	0.05	0.00	0.86	0.05	0.06
16475	DUSP7	0.00	0.00	0.02	0.06	0.65	0.41	0.00	0.01	0.00	0.01	0.97	0.05	0.31
17698	EIF5A	0.02	0.00	0.59	0.20	0.53	0.80	0.19	0.94	0.22	0.55	0.26	0.42	0.00
15762	ENST00000370040	0.00	0.23	0.69	0.99	0.07	0.61	0.00	0.00	0.00	0.02	0.96	0.17	0.32
20037	GAPDH	0.25	0.01	0.31	0.12	0.80	0.72	0.01	0.21	0.01	0.21	0.86	0.18	0.00
1039	GNAI2	0.09	0.07	0.24	0.60	0.15	0.51	0.07	0.20	0.22	0.65	0.56	0.63	0.00
21198	GNB2	0.02	0.00	0.15	0.28	0.40	0.65	0.06	0.36	0.06	0.37	0.33	0.37	0.00
714	IQCK	0.01	0.00	0.25	0.04	0.34	0.65	0.00	0.01	0.00	0.00	0.32	0.31	0.07
3891	MAF1	0.04	0.03	0.16	0.83	0.11	0.98	0.09	0.81	0.11	0.48	0.33	0.38	0.00
5918	MAP1S	0.02	0.01	0.20	0.57	0.08	0.59	0.25	0.71	0.12	0.91	0.11	0.44	0.00
18840	NFKBIL2	0.15	0.01	0.60	0.80	0.30	0.42	0.27	0.92	0.16	0.47	0.40	0.56	0.00
20917	NTN4	0.00	0.00	0.20	0.00	0.69	0.11	0.00	0.00	0.00	0.03	0.52	0.14	0.00
20295	PAH	0.00	0.08	0.61	0.67	0.28	0.29	0.00	0.00	0.00	0.07	0.42	0.57	0.16
22434	PCBP3	0.07	0.30	0.10	0.01	0.07	0.40	0.11	0.63	0.10	0.16	0.51	0.07	0.65
17776	PICK1	0.32	0.04	0.97	0.53	1.00	0.36	0.51	0.85	0.33	0.65	0.37	0.82	0.00
19411	PKMYT1	0.01	0.01	0.58	0.74	0.25	0.76	0.00	0.17	0.00	0.00	0.06	0.32	0.00
10998	PMS2CL	0.01	0.19	0.19	0.01	0.05	0.56	0.00	0.03	0.01	0.02	0.78	0.08	0.00
9472	POU2F1	0.00	0.01	0.54	0.15	0.79	0.39	0.05	0.29	0.06	0.06	0.26	0.24	0.96
11991	POU2F1	0.00	0.07	0.57	0.23	0.83	0.36	0.09	0.46	0.13	0.04	0.19	0.30	0.92
20014	PPP1R9A	0.33	0.09	0.97	0.37	0.31	0.38	0.24	0.30	0.36	0.19	0.21	0.75	0.91
4681	RAB42	0.00	0.03	0.79	0.12	0.74	0.44	0.00	0.02	0.00	0.20	0.82	0.08	0.00
3073	RANGAP1	0.02	0.04	0.11	0.41	0.31	0.42	0.11	0.73	0.30	0.24	0.10	0.08	0.00
3096	SALL2	0.00	0.28	0.09	0.89	0.74	0.15	0.00	0.00	0.00	0.00	0.04	0.50	0.00
7939	SLC25A1	0.24	0.90	0.61	0.40	0.16	0.93	0.08	0.14	0.38	0.46	0.10	0.12	0.03
9673	STK36	0.00	0.18	0.29	0.58	0.65	0.75	0.00	0.00	0.00	0.00	0.08	0.77	0.10
17750	TBC1D9	0.00	0.20	0.27	0.15	0.58	0.16	0.00	0.02	0.01	0.00	0.83	0.30	0.00
11119	TGM2	0.09	0.00	0.59	0.44	0.23	0.27	0.08	0.42	0.02	0.84	0.92	0.63	0.00
10109	THSD4	0.30	0.32	0.21	0.40	0.65	0.04	0.07	0.73	0.43	0.18	0.21	0.16	0.48
4051	TMC5	0.01	0.00	0.57	0.04	0.06	0.94	0.01	0.04	0.04	0.04	0.85	0.88	0.91
13619	TOMM40	0.12	0.00	0.35	0.35	0.37	0.92	0.09	0.54	0.08	0.21	0.25	0.43	0.00
6481	TTC19	0.00	0.10	0.50	0.24	0.98	0.23	0.00	0.00	0.00	0.04	0.21	0.39	0.00
10024	XBP1	0.00	0.72	0.51	0.03	0.99	0.43	0.00	0.03	0.00	0.00	0.63	0.06	0.71
7164	ZNF228	0.16	0.58	0.09	0.27	0.15	0.10	0.01	0.39	0.02	0.26	0.60	0.31	0.00
11969	ZNF598	0.36	0.01	0.07	0.75	0.86	0.64	0.08	0.99	0.02	0.29	0.34	0.11	0.00

Table S5.3. Clinicopathological characteristics of four subcohorts of infiltrating ductal breast carcinomas.

	N= 15	N= 8	N= 16	N= 35
Characteristic	Subcohort A	Subcohort B	Subcohort C	Subcohort D
Grade 1	8	0	8	1
Grade 2	7	3	8	12
Grade 3	0	4	0	20
NA	0	1	0	2
Tubule formation 1	1	0	1	0
Tubule formation 2	9	1	9	2
Tubule formation 3	5	4	6	26
NA	0	3	0	7
Nuclear pleomorphism 1	2	0	2	1
Nuclear pleomorphism 2	12	0	13	6
Nuclear pleomorphism 3	1	5	1	21
NA	0	3	0	7
Mitotic count 1	10	2	11	7
Mitotic count 2	5	1	5	13
Mitotic count 3	0	2	0	8
NA	0	3	0	7
Lymphovascular invasion negative	7	0	7	12
Lymphovascular invasion positive	7	8	8	20
NA	1	0	1	3
No. of lymph node metastasis 0	5	0	6	15
No. of lymph node metastasis 1	8	2	8	11
No. of lymph node metastasis 2	2	0	2	0
No. of lymph node metastasis 3	0	5	0	6
NA	0	1	0	3
lymph node metastasis negative	5	1	6	16
lymph node metastasis positive	10	6	10	17
NA	0	1	0	2
Tumor size 1	8	1	9	9
Tumor size 2	7	2	7	18
Tumor size 3	0	4	0	5
Tumor size 4	0	1	0	3
stage I	3	0	4	7
stage II	10	1	10	16
stage III	2	6	2	10
stage IV	0	0	0	1
NA	0	1	0	1

Table S5.4. Univariate and multivariate analyses for survival on prognostic factors in 90A and 181A cohorts, respectively. The p values of tests in the Cox proportional hazard model to be less or equal to 0.05 are highlighted with light blue.

	90A						181A					
	Univariate Analysis		Multivariate Analysis		Multivariate Analysis		Univariate Analysis		Multivariate Analysis		Multivariate Analysis	
Prognostic factor	Hazard Ratio	P value	Hazard Ratio	P value	Hazard Ratio	P value	Hazard Ratio	P value	Hazard Ratio	P value	Hazard Ratio	P value
Grade1 vs. 2&3	1.92	0.32	0.38	1.00	0.42	0.37	2.00	0.27	0.00	1.00	0.35	0.24
LVInegative vs. positive	3.30	0.13	0.00	1.00	2.50	0.44	4.34	0.02	3.31	1.00	2.95	0.17
Size 1 vs. 2&3 &4	1.76	0.38	0.15	1.00	0.57	0.51	2.97	0.04	26425064.00	1.00	0.55	0.37
NP 1 vs. 2&3	80907453.00	1.00	0.04	1.00	92700250.00	1.00	28131056.00	1.00	2.10E+14	1.00	324566238.00	1.00
TF 1 vs. 2&3	25794474.00	1.00	6.93	1.00	0.00	1.00	25962461.00	1.00	27767548.00	1.00	0.00	1.00
LNM 1 vs. 2&3	6.82	0.06	0.22	1.00	2052168000.00	1.00	5.64	0.00	168272720.00	1.00	20.33	0.04
MC 1 vs.2&3	1.39	0.59	4137473794.00	1.00	0.80	0.77	1.77	0.22	4137473794.00	1.00	1.31	0.65
Stage 1vs, 2&3&4	2.32	0.42	16.37	1.00	2.41	1.00	7.49	0.05	0.00	1.00	286595629.00	1.00
LYM positive vs. negative	6.49	0.07	0.71	0.81	0.10	0.06	3.99	0.01	0.15	0.19	0.16	0.09
41 gene signature (subcohort A/B)	2659960467.00	1.00	43333711704.00	1.00	-	-	-	-	-	-	-	-
41 gene signature (subcohort A/nonA)	277575040.00	1.00	-	-	302767300.00	1.00	-	-	-	-	-	-
41 gene signature (subcohort C/D)	-	-	-	-	-	-	349540283.00	1.00	11024502.00	1.00	-	-
41 gene signature (subcohort C/nonC)	-	-	-	-	-	-	93278022.00	1.00	-	-	722655769.00	1.00

Figure S6.1. Heatmaps for the common gene pool in both cell cycle signal transduction pathway and the network of *MYB_ARNT2* in 90A cohort.

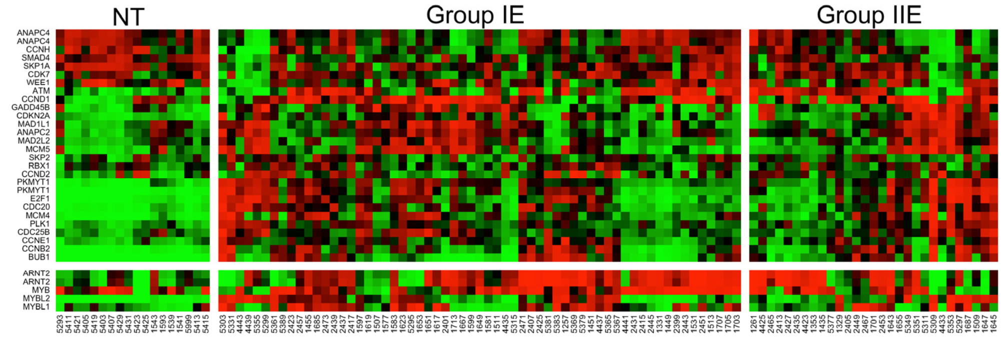

Figure S6.2. Heatmaps for the common gene pool in both VEGF signal transduction pathway and the network of *MYB_ARNT2* in 90A cohort.

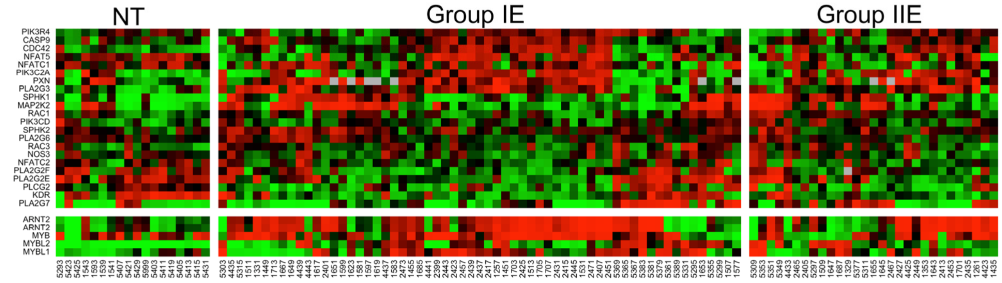

Figure S6.3. Heatmaps for the common gene pool in both p53 signal transduction pathway and the network of *MYB_ARNT2* in 90A cohort.

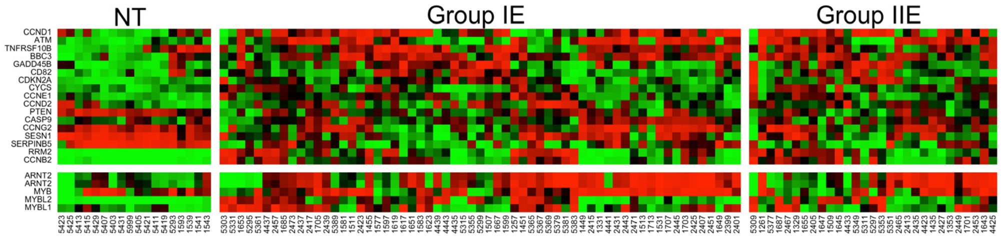

Figure S6.4. Heatmaps for the common gene pool in both ribosome signal transduction pathway and the network of *MYB_ARNT2* in 90A cohort.

Grey color within the heatmap stands for data not available after data processing.

In this case, we do not use this piece of information for further discussion due to most data not available.

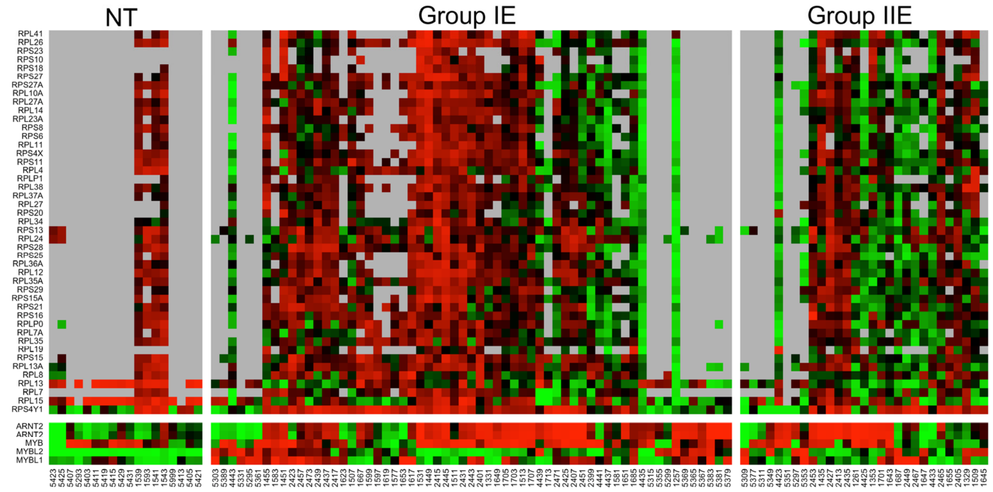

Figure S6.5. Heatmaps for the common gene pool in both PDGFRB signal transduction pathway and the network of *MYB_ARNT2* in 90A cohort.

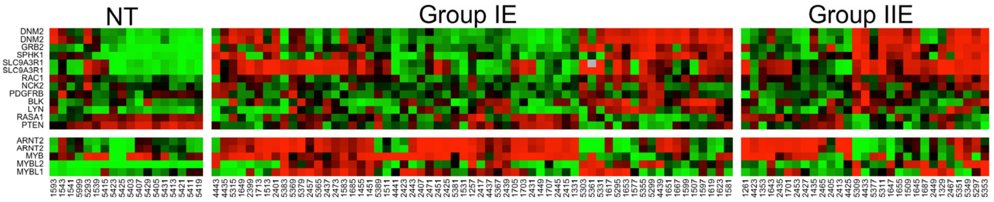

Figure S6.6. Heatmaps for the common gene pool in both ERBB2 signal transduction pathway and the network of *MYB_ARNT2* in 90A cohort.

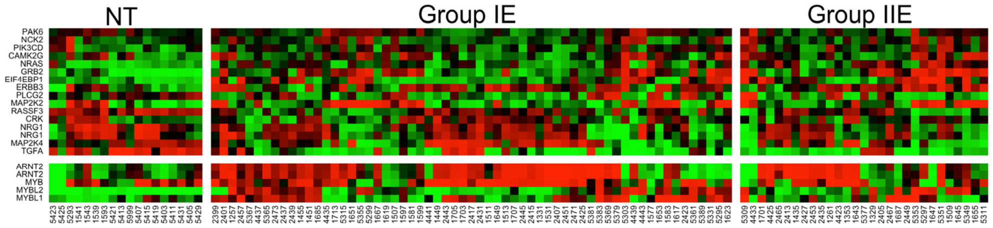

Figure S6.7. Heatmaps for the common gene pool significant for both histological grade (Grade) and the network of *MYB_ARNT2* in 90A cohort.

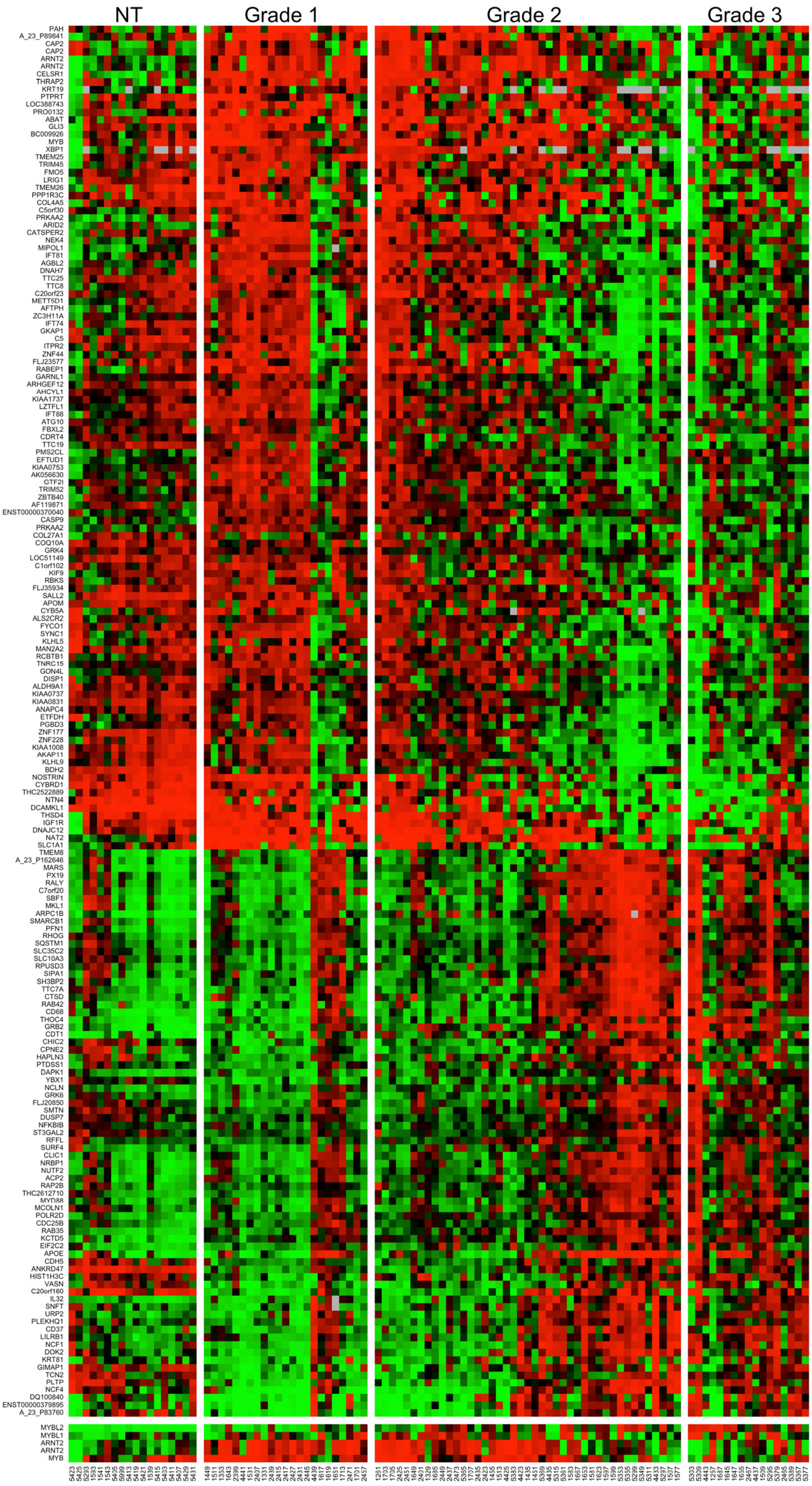
NT
Grade 1
Grade 2
Grade 3
PAH
A_23_P89841
CAP2
CAP2
ARNT2
ARNT2
CELSR1
THRAP2
KRT19
PTPRT
LOC388743
PRO0132
ABAT
GLI3
BC009926
MYB
XBP1
TMEM25
TRIM45
FMO5
LRIG1
TMEM26
PPP1R3C
COL4A5
C5orf30
PRKAA2
ARID2
CATSPER2
NEK4
MIPOL1
IFT81
AGBL2
DNAH7
TTC25
TTC8
C20orf23
METT5D1
AFTPH
ZC3H11A
IFT74
GKAP1
C5
ITPR2
ZNF44
FLJ23577
RABEP1
GARNL1
ARHGEF12
AHCYL1
KIAA1737
LZTFL1
IFT88
ATG10
FBXL2
CDRT4
TTC19
PMS2CL
EFTUD1
KIAA0753
AK056630
GTF2I
TRIM52
ZBTB40
AF119871
ENST00000370040
CASP9
PRKAA2
COL27A1
COQ10A
GRK4
LOC51149
C1orf102
KIF9
RBKS
FLJ35934
SALL2
APOM
CYB5A
ALS2CR2
FYCO1
SYNC1
KLHL5
MAN2A2
RCBTB1
TNRC15
GON4L
DISP1
ALDH9A1
KIAA0737
KIAA0831
ANAPC4
ETFDH
PGBD3
ZNF177
ZNF228
KIAA1008
AKAP11
KLHL9
BDH2
NOSTRIN
CYBRD1
THC2522889
NTN4
DCAMKL1
THSD4
IGF1R
DNAJC12
NAT2
SLC1A1
TMEM8
A_23_P162646
MARS
PX19
RALY
C7orf20
SBF1
MKL1
ARPC1B
SMARCB1
PFN1
RHOG
SQSTM1
SLC35C2
SLC10A3
RPUSD3
SIPA1
SH3BP2
TTC7A
CTSD
RAB42
CD68
THOC4
GRB2
CDT1
CHIC2
CPNE2
HAPLN3
PTDSS1
DAPK1
YBX1
NCLN
GRK6
FLJ20850
SMTN
DUSP7
NFKBIB
ST3GAL2
RFFL
SURF4
CLIC1
NRBP1
NUTF2
ACP2
RAP2B
THC2612710
MYD88
MCOLN1
POLR2D
CDC25B
RAB35
KCTD5
EIF2C2
APOE
CDH5
ANKRD47
HIST1H3C
VASN
C20orf160
IL32
SNFT
URP2
PLEKHQ1
CD37
LILRB1
NCF1
DOK2
KRT81
GIMAP1
TCN2
PLTP
NCF4
DQ100840
ENST00000379895
A_23_P83760
MYBL2
MYBL1
ARNT2
ARNT2
MYB

Figure S6.8. Heatmaps for the common gene pool significant for both lymphovascular invasion (LVI) and the network of *MYB_ARNT2* in 90A cohort.

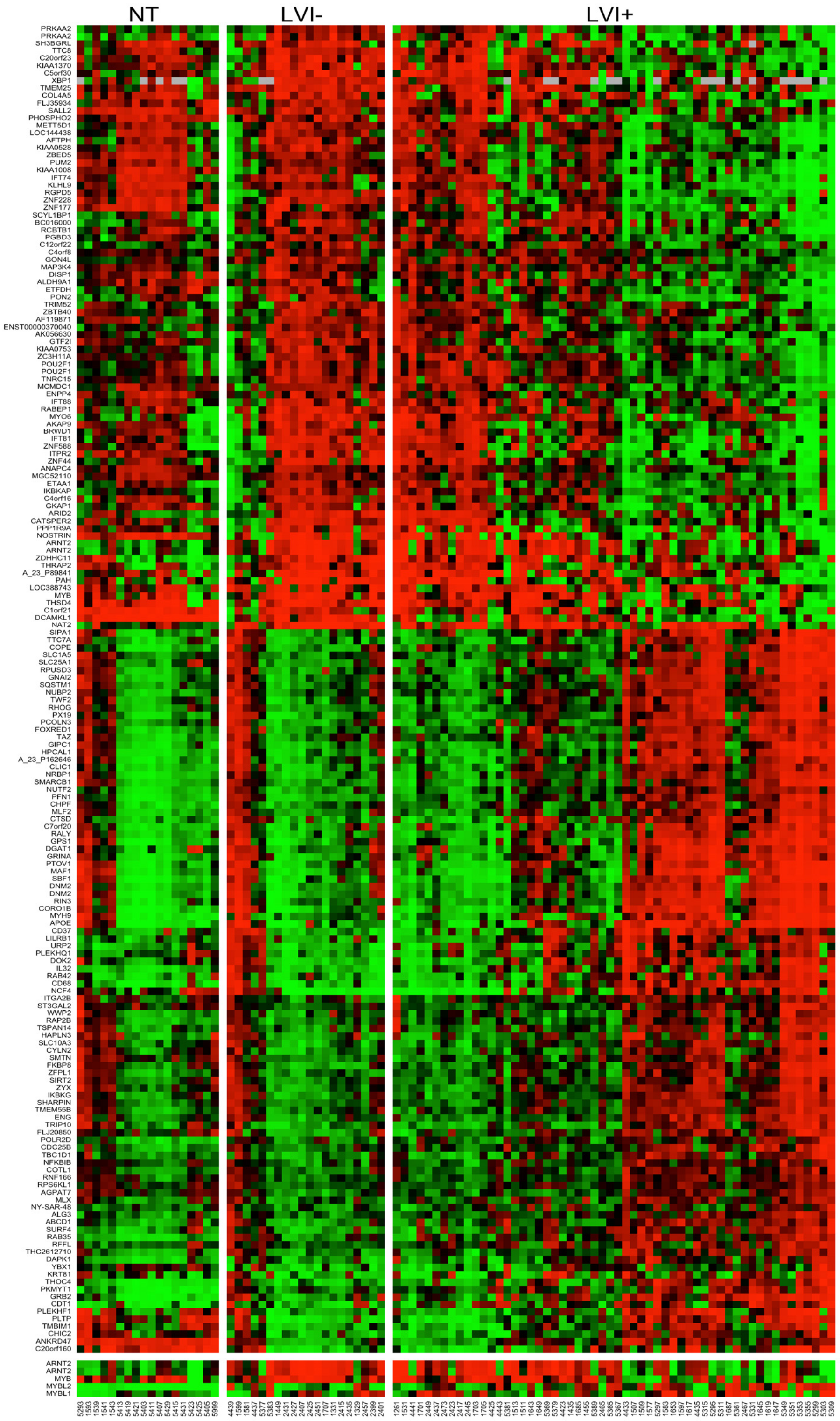

Figure S6.9. Heatmaps for the common gene pool significant for both tumor size (size) and the network of *MYB_ARNT2* in 90A cohort.

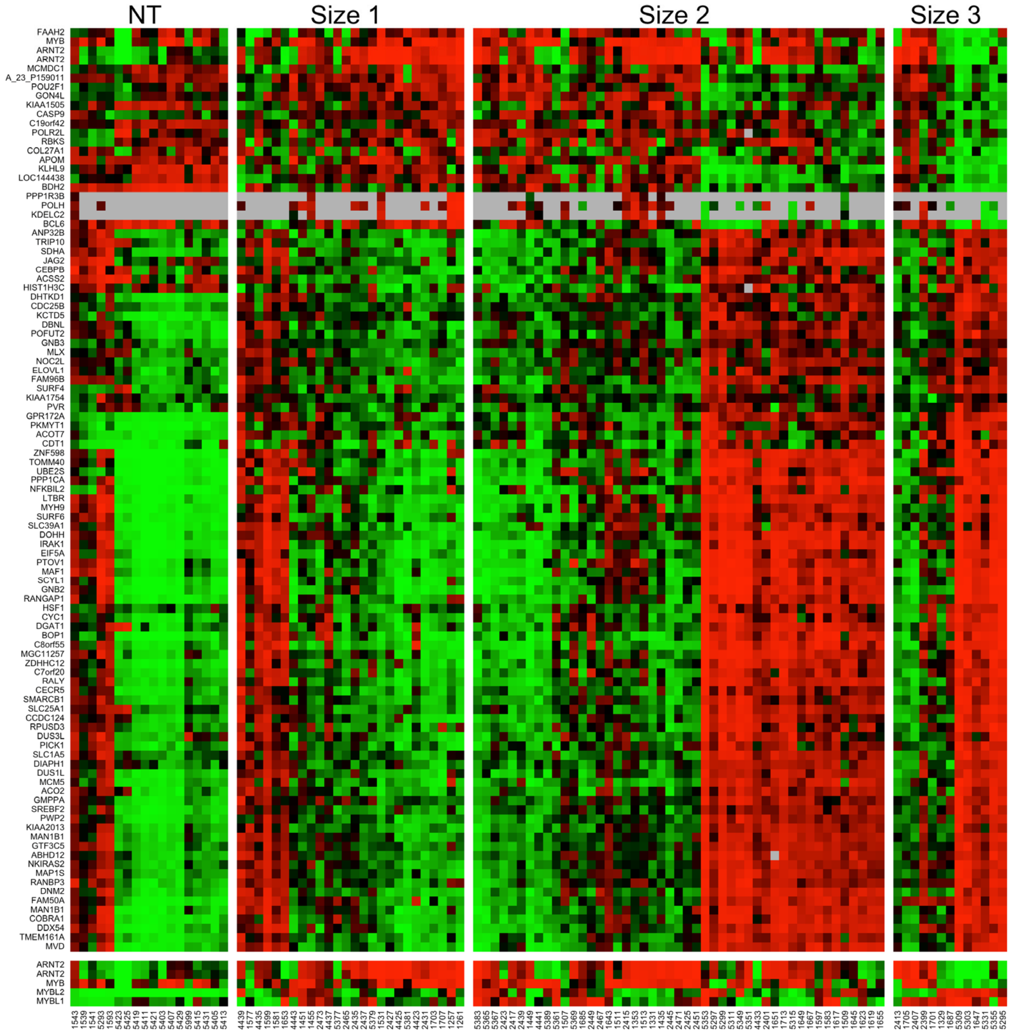

Figure S6.10. The most relevant subnetworks of *MYB*, *ARNT2* and *SALL2* for epithelial-to-mesenchymal transition (EMT) activities in 90A cohort.

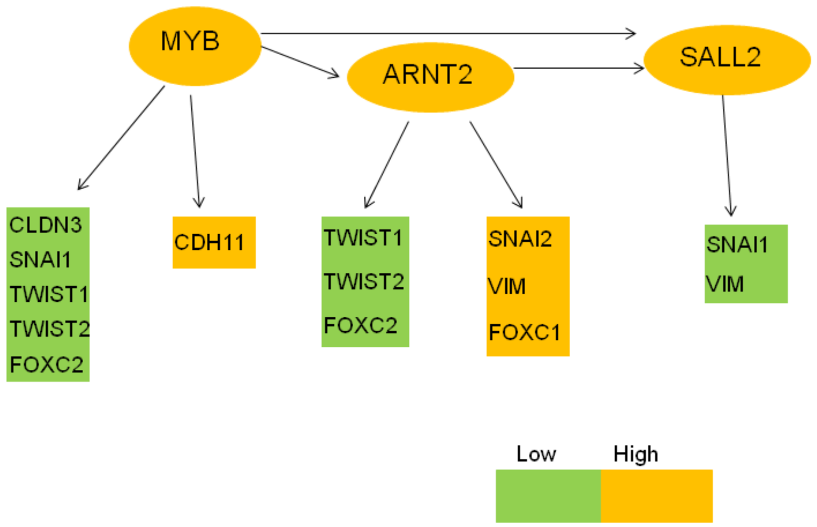

Table S6.1. . Partial results of the supervised network analysis in Tables S1.3 and S1.4 show *MYB* and *ARNT2* shared target genes (57 genes) overlapping with the downstream target genes of ARNT2/SIM1. Liu C *et al.*(2003)〔21〕 reported that ARNT2 dimerizes with SIM1 to up-regulate their downstream target genes (268 probes) *in vitro*, which are predicted to be functional in seven categories - transcription regulators, signaling components, metabolic enzymes, channels and transporters, cell adhesion and migration, miscellaneous and uncharacterized.

Cohorts	The putative target genes of *MYB* and *ARNT2* validated to be downstream target genes of ARNT2/ SIM1 〔21〕
Groups IE and IIE	NACA,HGFAC, GALNT4, GRIN1, SERPINA5, FNBP1L, SPRR1A, COL12A1, CDH3, LRRFIP1, FHIT, EIF5A, PFN1, BAT3, TERF1,TNFRSF9, CAMLG, SYT5, MRPL2, AKT1, CD86.
Group IE less Group IIE	AHR, CBFB, EYA1, SOX10, MDM4, TCF3, NR5A1, FOXH1, ARNT2, WNT7B, SMAD3, MTPN, TYROBP, TNF, EGFR,GPX3, OAZ1, CALB2, ADAM28, LCAT, LGALS9, CD86, CD52, GUCY2C, CRYGD, POLD1, APOE.
Group IIE less Group IE	HOMER1, LIFR, CCR1, GSTA3, KLK9, APOA5, SP100, NMBR, SIM1.

Figure S7.1. Mean plot analyses of mRNA levels for *POU2F1*(9472) in eight clinical categories and in three cohorts of infiltrating ductal carcinoma (IDCs), respectively. Lymphovascular invasion (LVI), nodal category (lymph node metastasis (LYM), number of nodal metastasis(LNM)), histological grade (Grade) category (nuclear pleomorphism (NP) and tubule formation (TF)) and stage were analyzed. Cohort 1 (90A) has Groups IE and IIE. Cohort 2 (91A) has ER(-) subtypes (see main text for definitions). Cohort 3(181A) has cohorts 1 and 2.

Cohort 1

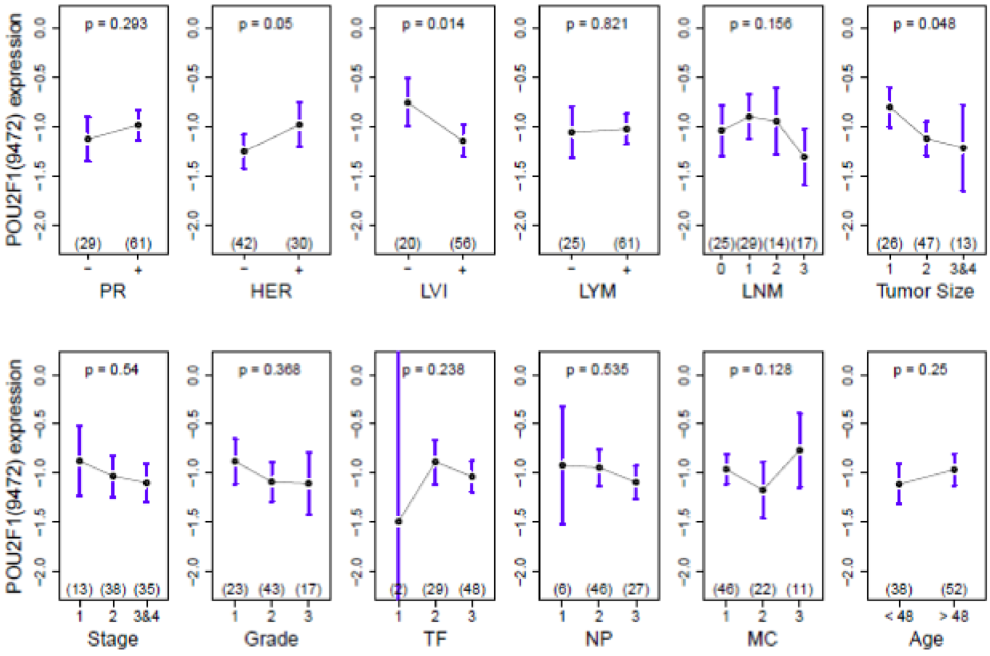

B. Cohort 2.

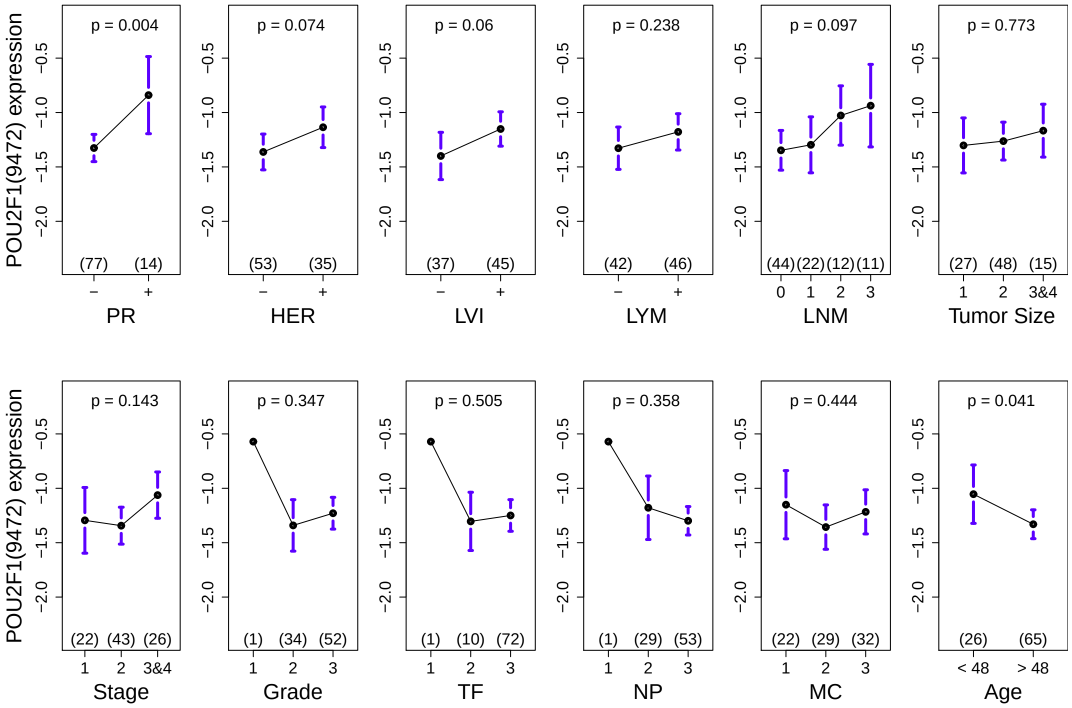

C. Cohort 3.

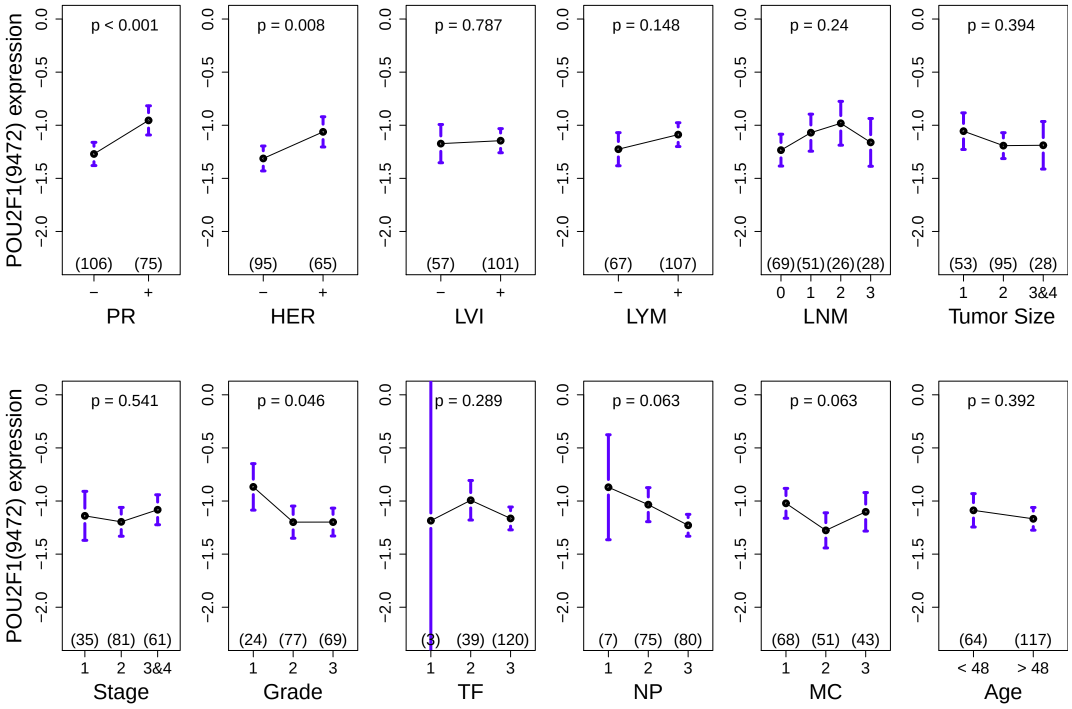

Figure S7.2. Mean plot analyses of mRNA levels for *POU2F1* (11991) in eight clinical categories and in three cohorts of infiltrating ductal carcinoma (IDCs), respectively. Lymphovascular invasion (LVI), nodal category (lymph node metastasis (LYM), number of nodal metastasis (LNM)), histological grade (Grade) category (nuclear pleomorphism (NP) and tubule formation (TF)) and stage were analyzed. Cohort 1 (90A) has Groups IE and IIE. Cohort 2 (91A) has ER(−) subtypes (see main text for definitions). Cohort 3 (181A) has cohorts 1 and 2.

A. Cohort 1.

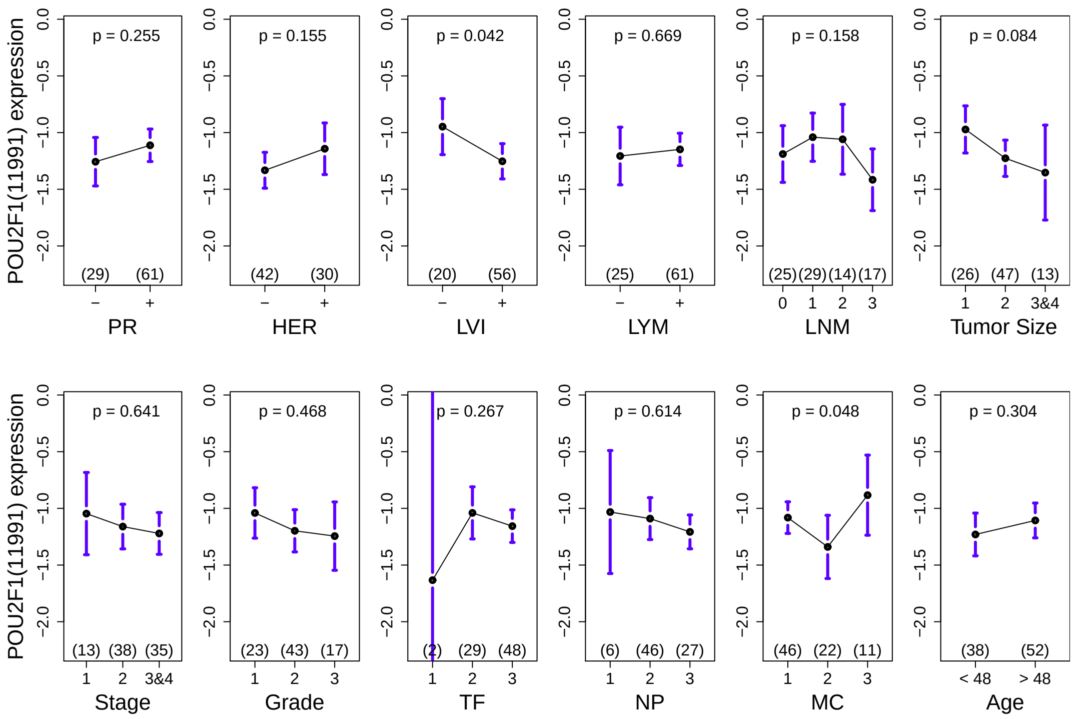

B. Cohort 2.

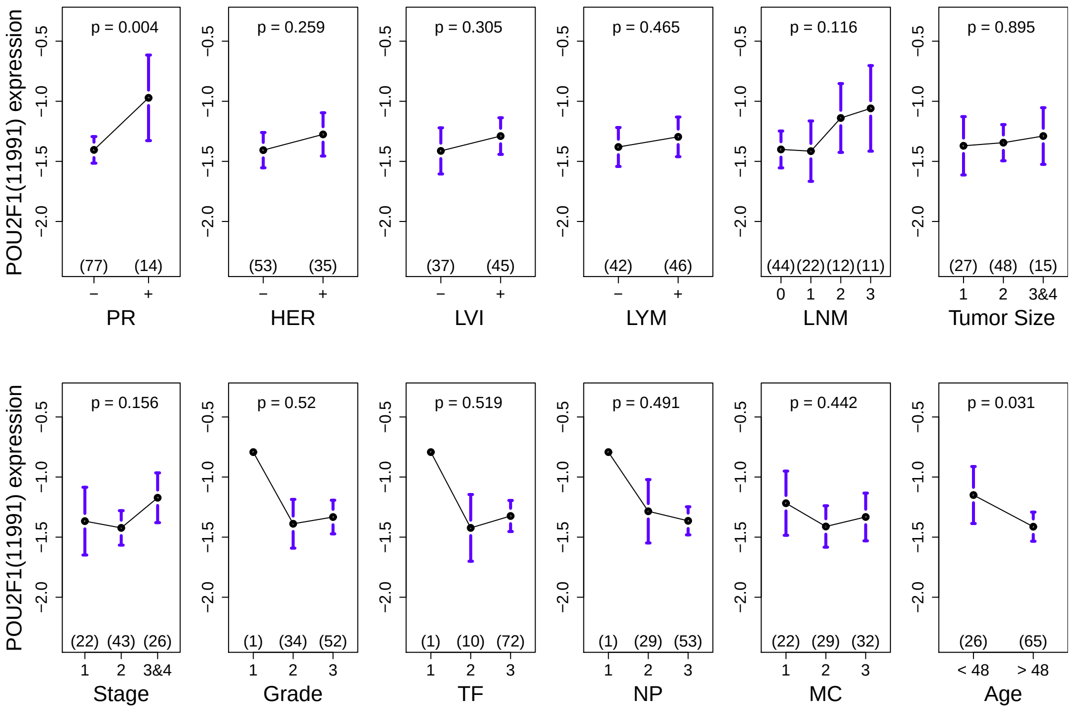

C. Cohort 3.

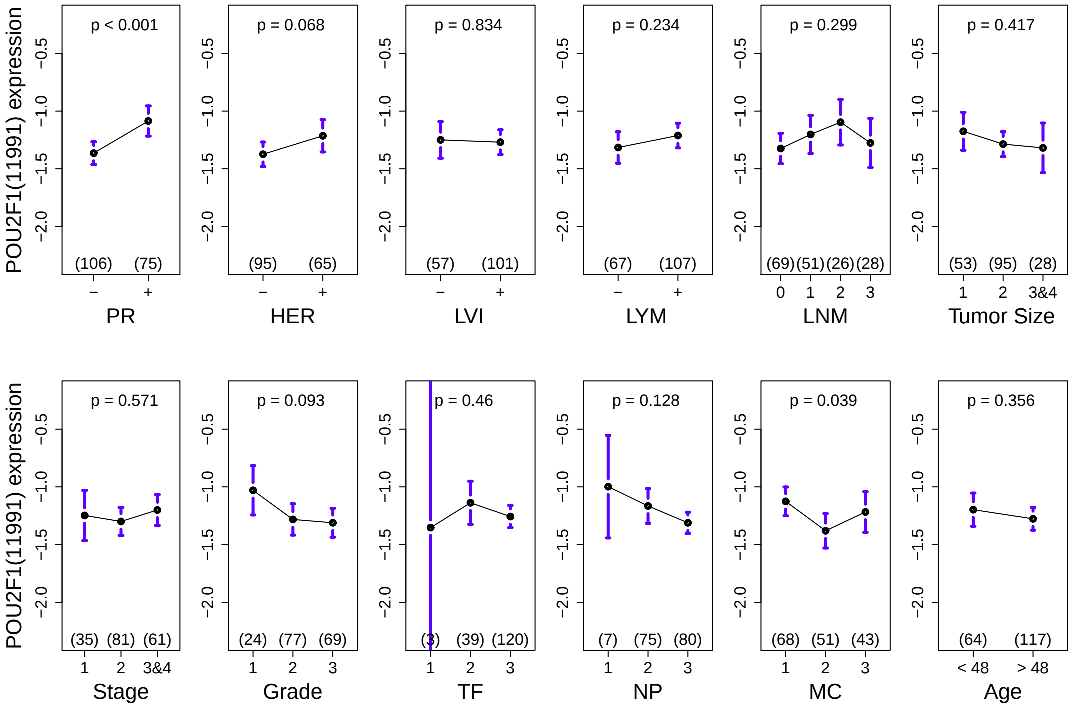

Figure S7.3. Mean plot analyses of mRNA levels for *SALL2* (3096) in eight clinical categories and in three cohorts of infiltrating ductal carcinoma (IDCs), respectively. Lymphovascular invasion (LVI), nodal category (lymph node metastasis (LYM), number of nodal metastasis (LNM)), histological grade (Grade) category (nuclear pleomorphism (NP) and tubule formation (TF)) and stage were analyzed. Cohort 1 (90A) has Groups IE and IIE. Cohort 2 (91A) has ER(−) subtypes (see main text for definitions). Cohort 3 (181A) has cohorts 1 and 2.

A. Cohort 1.

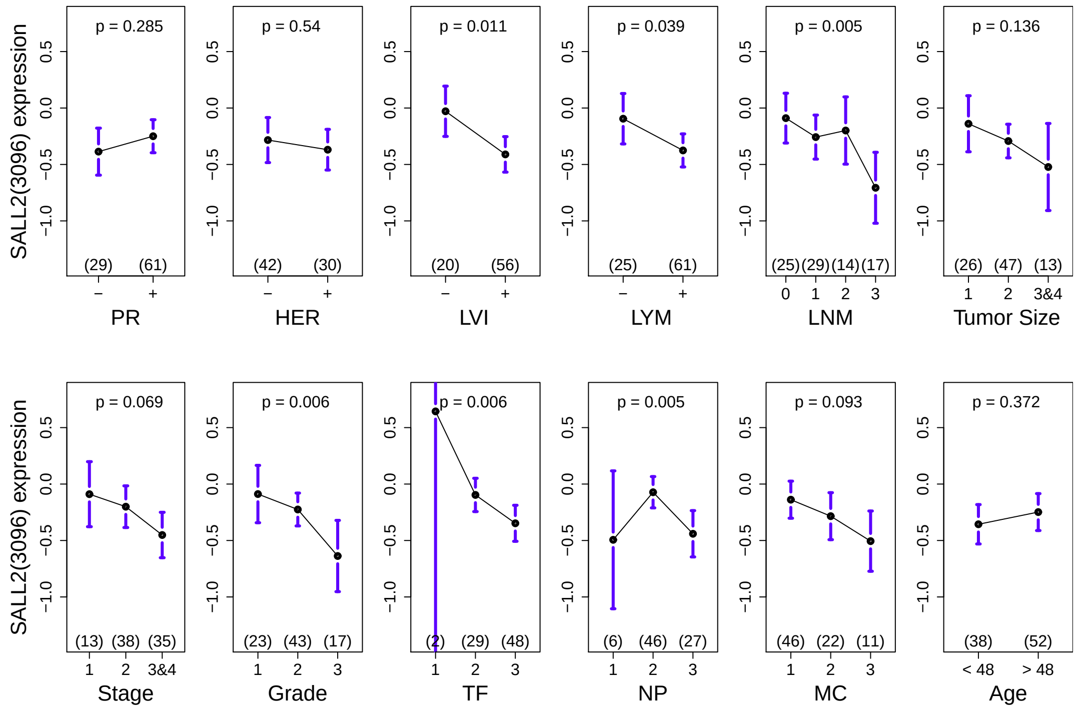

B. Cohort 2.

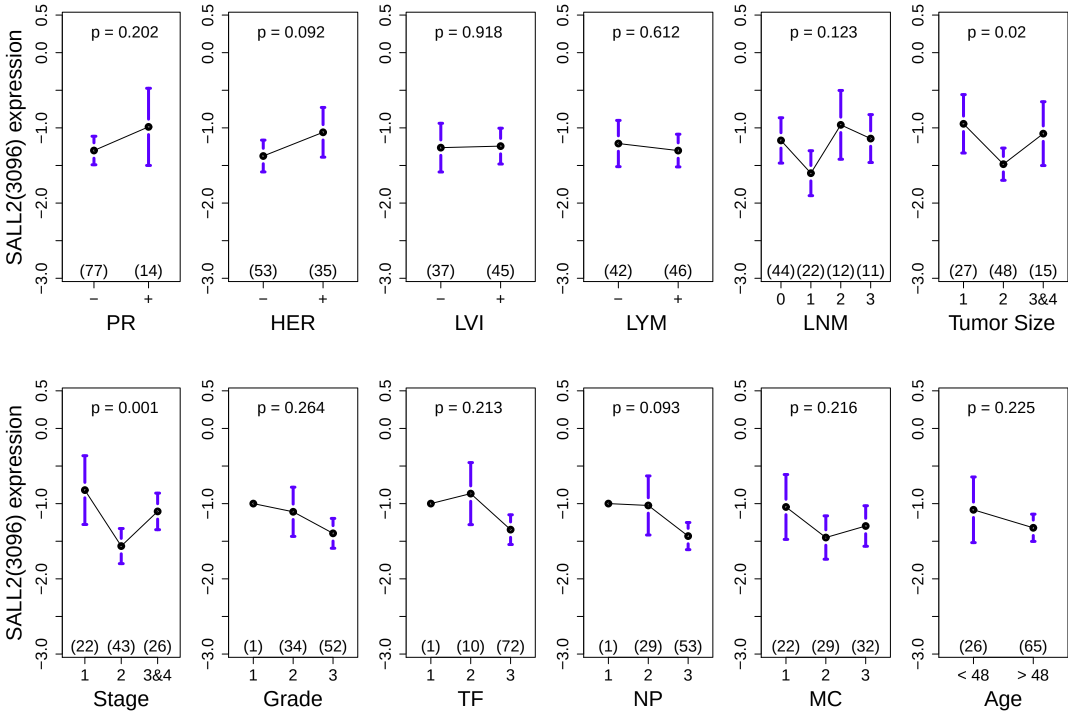

C. Cohort 3.

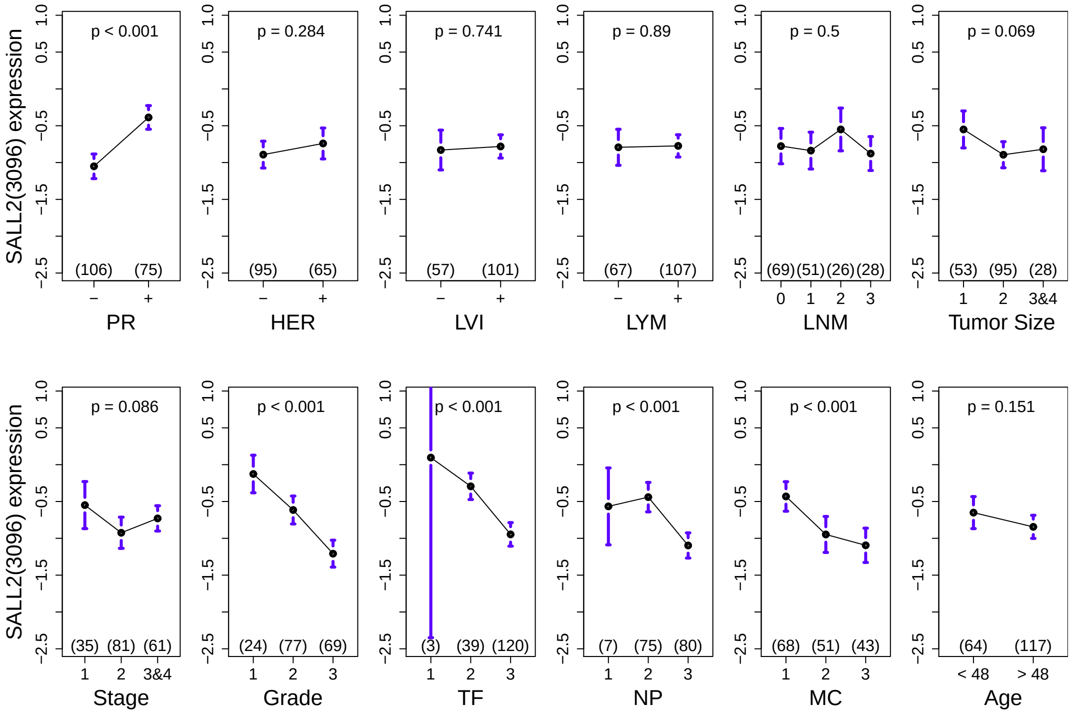

Figure S7.4. Mean plot analyses of mRNA levels for *XBP1* (10024) in eight clinical categories and in three cohorts of infiltrating ductal carcinoma (IDCs), respectively. Lymphovascular invasion (LVI), nodal category (lymph node metastasis (LYM), number of nodal metastasis (LNM)), histological grade (Grade) category (nuclear pleomorphism (NP) and tubule formation (TF)) and stage were analyzed. Cohort 1 (90A) has Groups IE and IIE. Cohort 2 (91A) has ER(−) subtypes (see main text for definitions). Cohort 3 (181A) has cohorts 1 and 2.

A. Cohort 1.

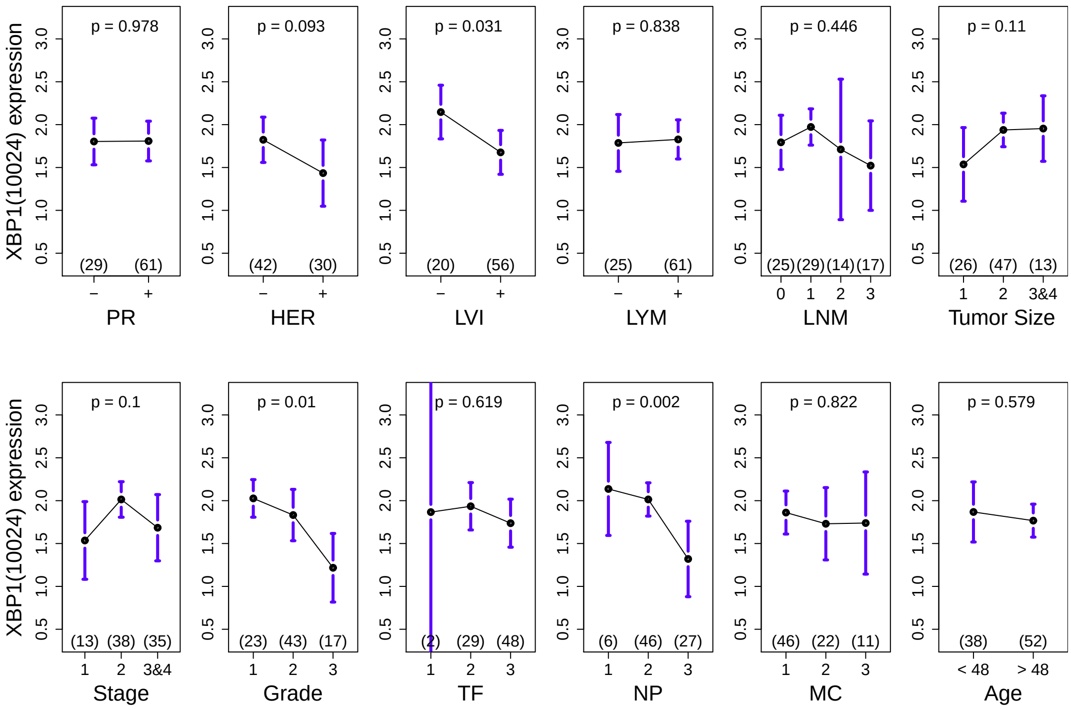

B. Cohort 2.

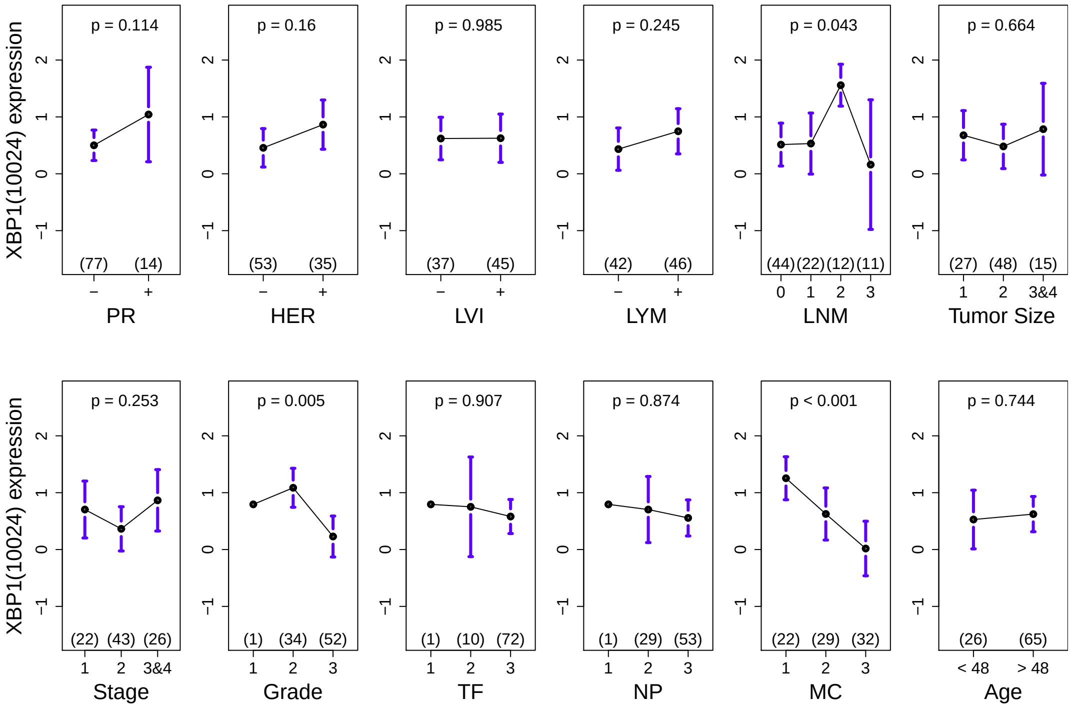

C. Cohort 3.

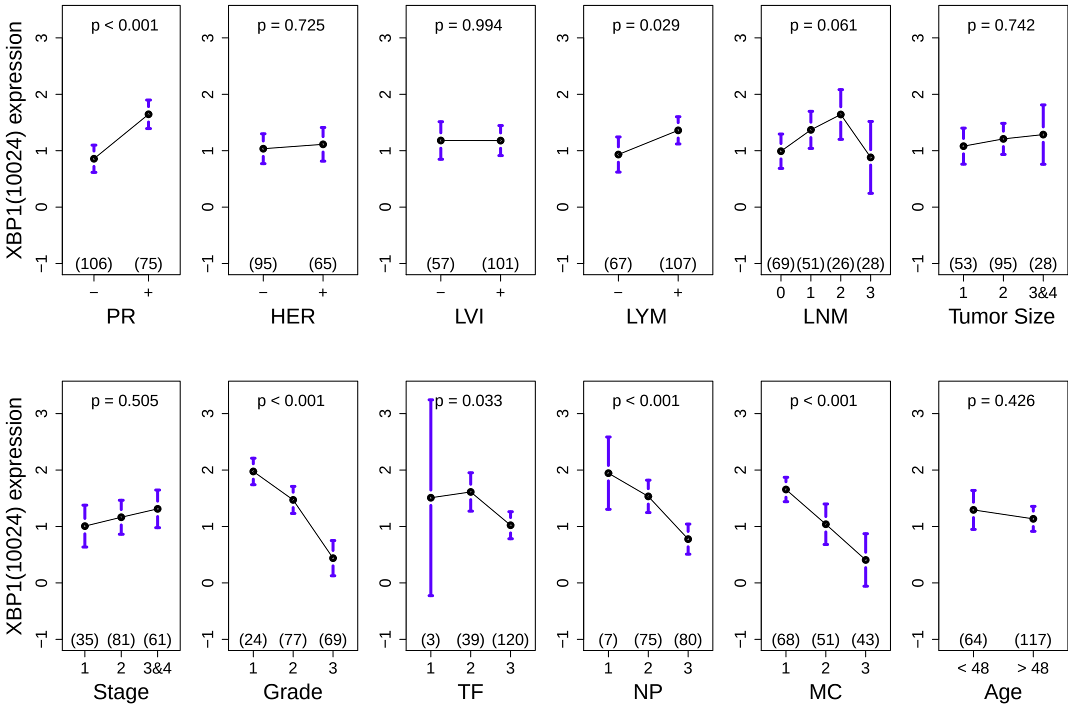

Figure S7.5. Mean plot analyses of mRNA levels for *MYBL1* (C11830) in eight clinical categories and in three cohorts of infiltrating ductal carcinoma (IDCs), respectively. Lymphovascular invasion (LVI), nodal category (lymph node metastasis (LYM), number of nodal metastasis (LNM)), histological grade (Grade) category (nuclear pleomorphism (NP) and tubule formation (TF)) and stage were analyzed. Cohort 1 (90A) has Groups IE and IIE. Cohort 2 (91A) has ER(−) subtypes (see main text for definitions). Cohort 3 (181A) has cohorts 1 and 2.

A. Cohort 1.

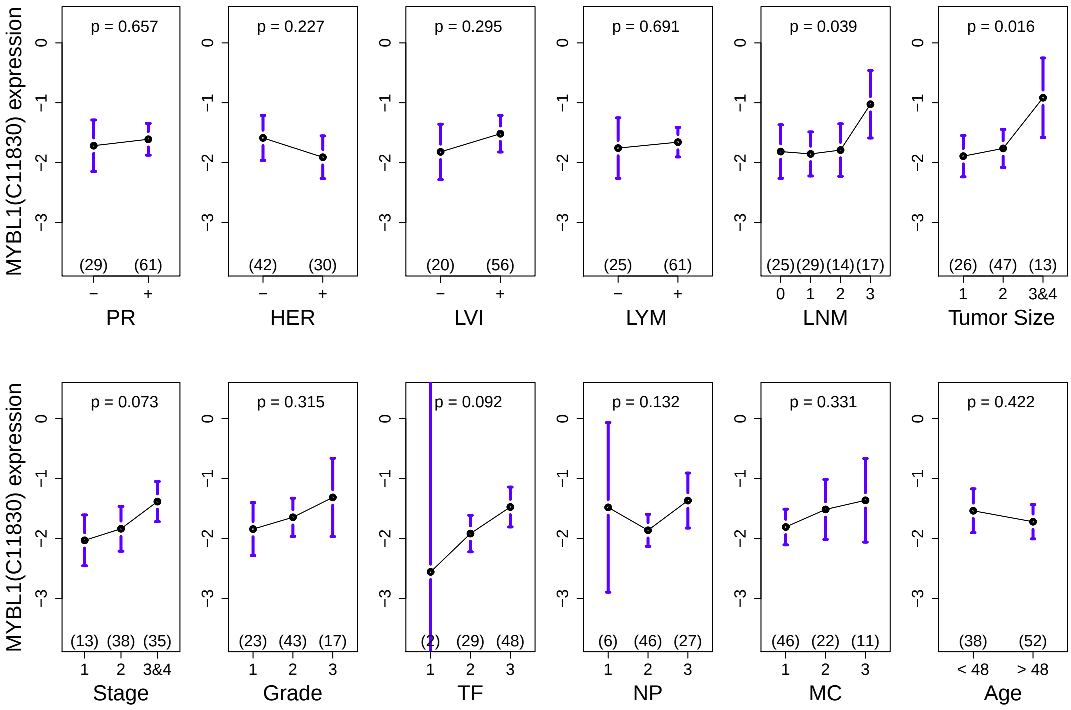

B. Cohort 2.

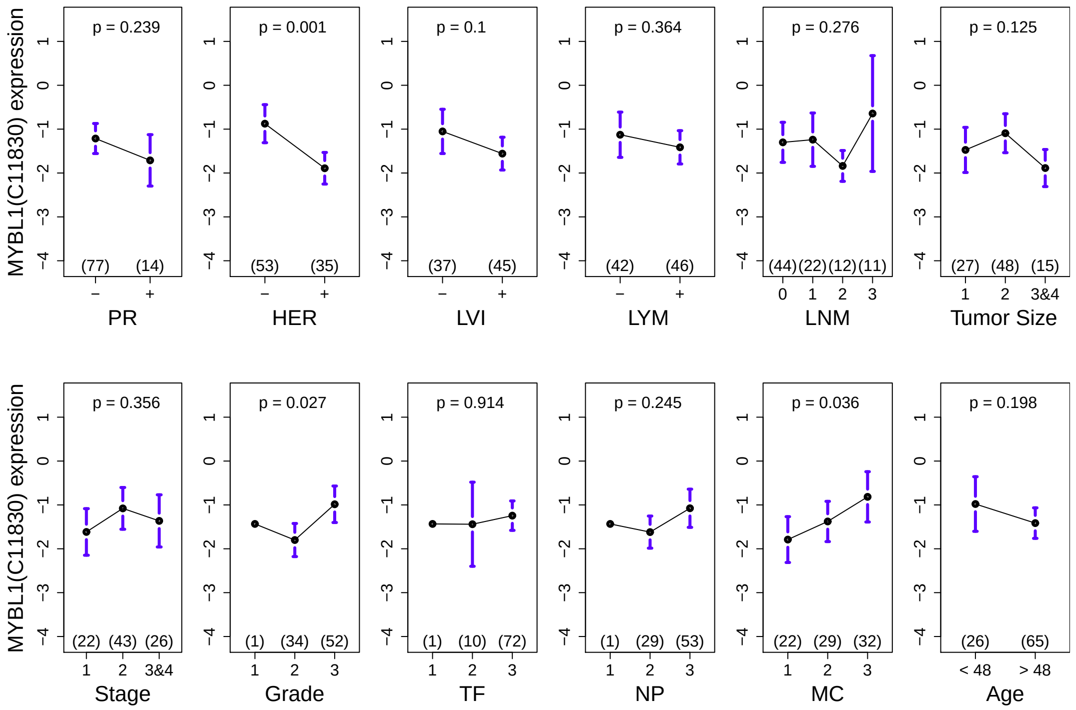

C. Cohort 3.

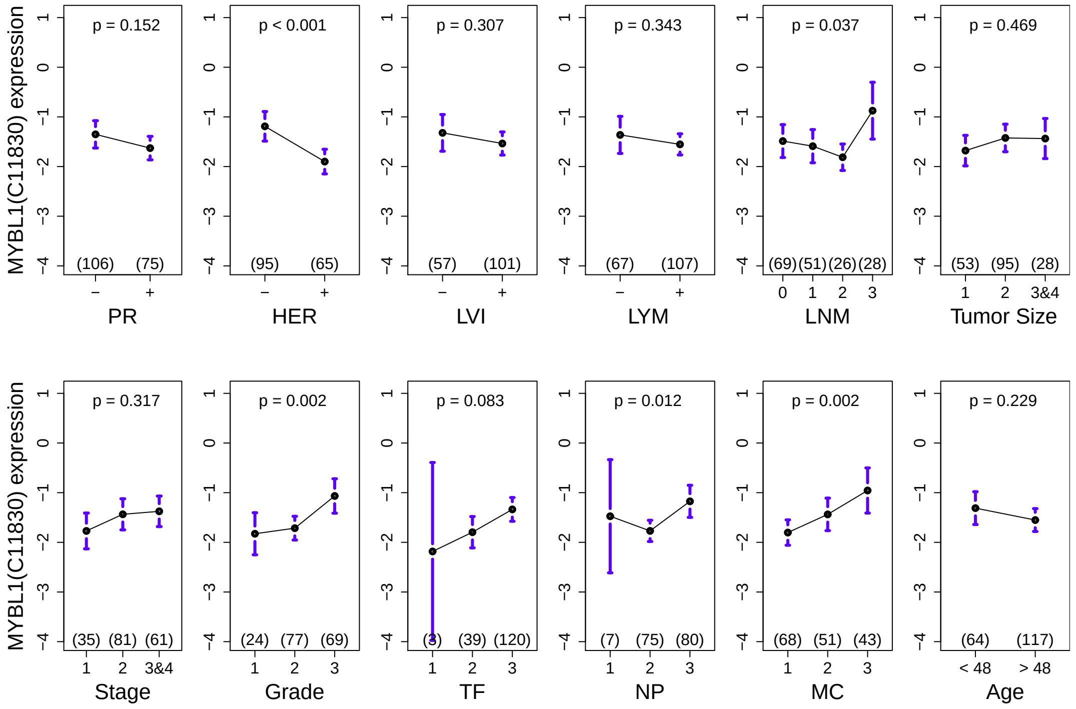

Figure S7.6. Mean plot analyses of mRNA levels for *MYBL2* (10757) in eight clinical categories and in three cohorts of infiltrating ductal carcinoma (IDCs), respectively. Lymphovascular invasion (LVI), nodal category (lymph node metastasis (LYM), number of nodal metastasis (LNM)), histological grade (Grade) category (nuclear pleomorphism (NP) and tubule formation (TF)) and stage were analyzed. Cohort 1 (90A) has Groups IE and IIE. Cohort 2 (91A) has ER(−) subtypes (see main text for definitions). Cohort 3 (181A) has cohorts 1 and 2.

A. Cohort 1.

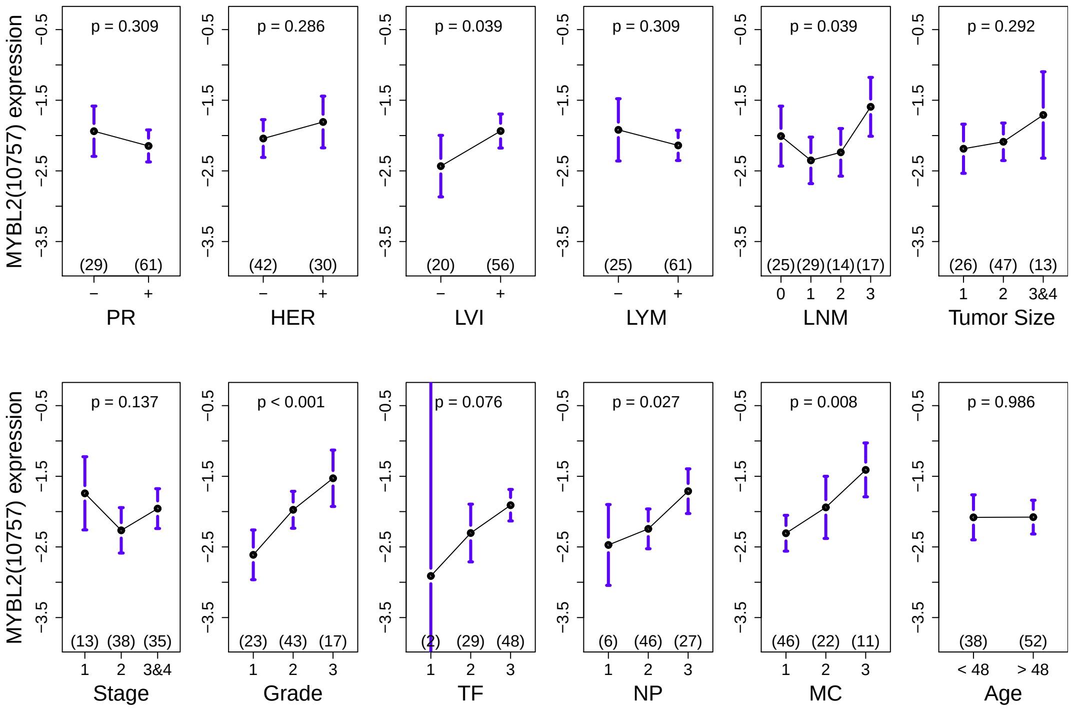

B. Cohort 2.

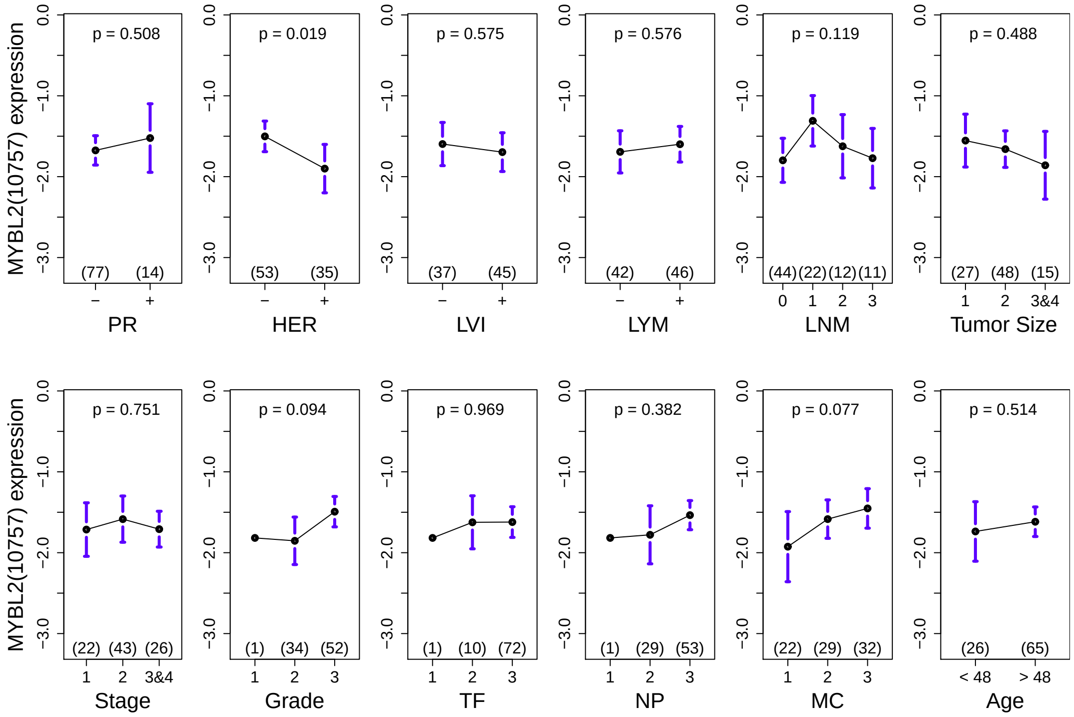

C. Cohort 3.

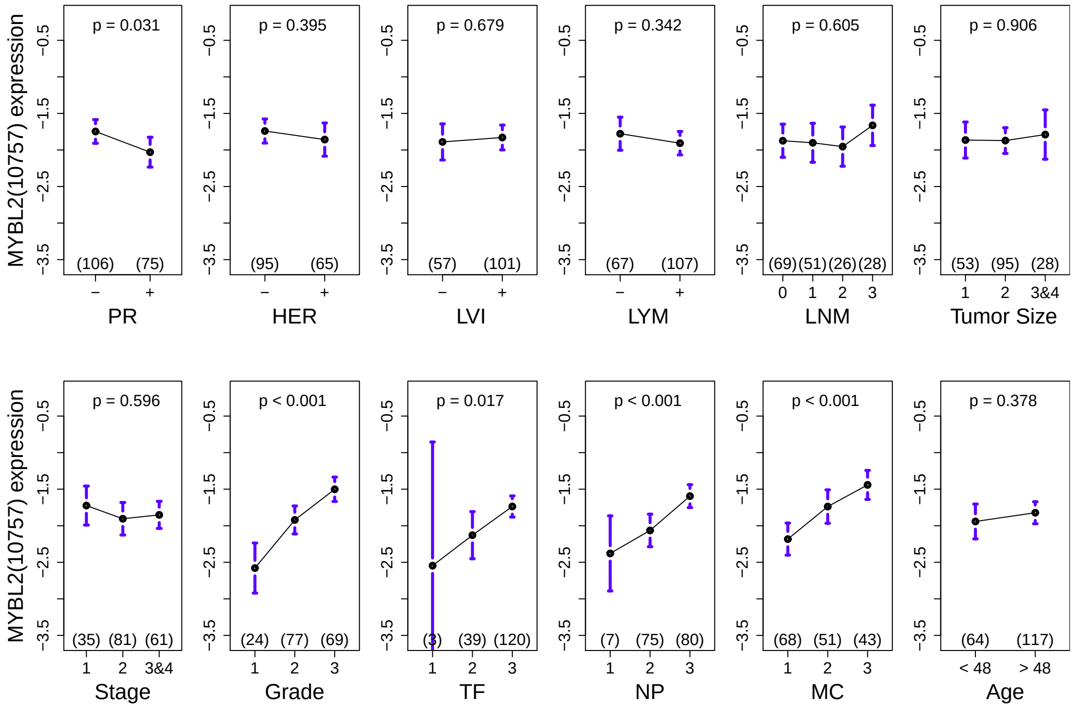

Figure S8.1. The heatmaps for the expression pattern of forty one probes in relation to the gene expression patterns of the four transcription factors (ARNT2, MYB, MYBL1 and MYBL2) in three cohorts.

18A cohort stands for non-tumor component(NT). 61A cohort stands for group IE subtype. 29A cohort stands for group IIE subtype.

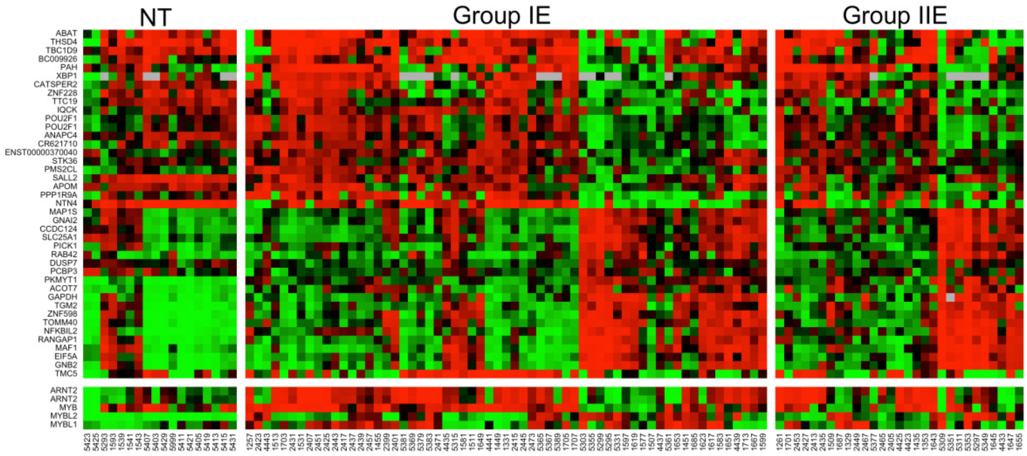

Figure S8.2. The heatmaps for the expression pattern of forty one probes in relation to the gene expression patterns of the four transcription factors (ARNT2, MYB, MYBL1 and MYBL2) in two cohorts.

25A cohort stands for non-tumor component (NT). 181A cohort stands for tumor component consisting of 90 ER(+) IDCs and 91 ER(-) IDCs.

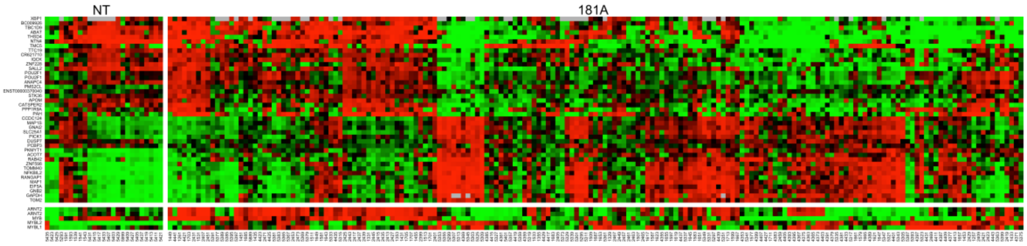

Supplement: Supplementary file 1 — There are eight supplemental files gathered to be the additional file 1. We collected the Venn diagram figures and their corresponding gene pools to form the unique table. It consists of supplemental files 1-4. The major results of survival analyses are listed in supplemental file 5. We have gathered the key results of the network analyses in supplemental file 6. It includes the clinical relevant gene profiling, the biochemical profiling and the partially validated results of the network analysis. We put the ANOVA test results of a few important transcriptions factors for showing their clinical impacts in supplemental file 7. The gene expression patterns of the 41-gene signature in two subtypes of ER(+) IDCs and 181 IDCs are shown in supplemental file 8. [file 813067.f1.pdf]
